# AEnet: a practical tool to construct the splicing-associated phenotype atlas at a single cell level

**DOI:** 10.1093/gigascience/giaf110

**Published:** 2025-09-24

**Authors:** Shang Liu, Xi Chen, Xiaohu Huang, Yuhang Wang, Waidong Huang, Pengfei Qin, Rui Li, Xuanxuan Zou, Wending Pang, Xiaoyun Huang, Shiping Liu, Yinqi Bai, Liang Wu

**Affiliations:** BGI Research, Chongqing 401329, China; Ruijin Yangtze River Delta Health Institute, Wuxi Branch of Ruijin Hospital, Ruijin Hospital, Shanghai Jiao Tong University School of Medicine, Shanghai 200025, China; State Key Laboratory of Genome and Multi-omics Technologies, BGI Research, Shenzhen 518083, China; BGI Research, Shenzhen 518083, China; BGI Research, Chongqing 401329, China; School of Biology and Biological Engineering, South China University of Technology, Guangzhou 510006, China; BGI Research, Chongqing 401329, China; School of Biology and Biological Engineering, South China University of Technology, Guangzhou 510006, China; BGI Research, Chongqing 401329, China; College of Life Sciences, University of Chinese Academy of Sciences, Beijing 101408, China; BGI Research, Chongqing 401329, China; State Key Laboratory of Genome and Multi-omics Technologies, BGI Research, Shenzhen 518083, China; BGI Research, Shenzhen 518083, China; Institute of Intelligent Medical Research (IIMR), BGI Genomics, Shenzhen 518083, China; BGI Research, Chongqing 401329, China; Department of Neurology, Hubei Provincial Clinical Research Center for Parkinson’s Disease, Xiangyang No.1 People's Hospital, Hubei University of Medicine, Xiangyang 441000, China; BGI Research, Chongqing 401329, China; School of Biology and Biological Engineering, South China University of Technology, Guangzhou 510006, China; JC School of Public Health and Primary Care, Faculty of Medicine, The Chinese University of Hong Kong, Hong Kong SAR 999077, China; State Key Laboratory of Genome and Multi-omics Technologies, BGI Research, Hangzhou 310030, China; BGI Research, Hangzhou 310030, China; BGI Research, Sanya 572025, China; BGI Research, Chongqing 401329, China; State Key Laboratory of Genome and Multi-omics Technologies, BGI Research, Shenzhen 518083, China; BGI Research, Shenzhen 518083, China; Zhongshan-BGI Precision Medical Center, Zhongshan Hospital, Fudan University, Shanghai 200032, China; Shanxi Medical University-BGI Collaborative Center for Future Medicine, Shanxi Medical University, Taiyuan 030001, China

**Keywords:** Single-cell analysis, Alternative Splicing, Clustering, Regulatory network prediction

## Abstract

Alternative splicing (AS), a crucial driver of proteomic diversity, is a fundamental source of cellular heterogeneity alongside gene expression levels. AS is closely linked to various physiological and pathological processes, including tumor progression and embryonic development. Single-cell RNA sequencing (scRNA-seq) technologies capture AS events through junction reads at cellular resolution, enabling the identification of core AS events that regulate specific cell types or states. However, single-cell sequencing technologies and their data are plagued by inherent limitations, such as shallow sequencing depth, high dropout rates, and batch effects. Furthermore, previous clustering approaches have overlooked the crucial interplay between AS and gene expression in defining distinct “cell types,” posing ongoing challenges in this field. In this study, we present a novel method called Alternative Splicing-Gene Expression Network (AEnet), which combines gene expression levels with AS patterns to profile cellular heterogeneity and define what we term “cell subpopulations.” AEnet also identifies key AS events and infers the regulatory mechanisms underlying these events. By applying AEnet to tumor cells, pan-cancer immune cells, and embryonic cells, we demonstrate enhanced cell clustering, the identification of novel AS events with potential functional importance, and the discovery of the key splicing factors involved in cell state transitions. The application of AEnet provides new insights into cellular heterogeneity and its role in both physiological and pathological processes.

## Introduction

The diversity of proteomes is an important manifestation of the complexity of organisms, and alternative splicing (AS) is one of the major factors contributing to this diversity [1, 2]. The major types of alternative splicing, including exon skipping (SE), mutually exclusive exons (MXEs), intron retention (IR), alternative 3′ splice site (A3SS), alternative 5′ splice site (A5SS), alternative last exon (ALE), alternative first exon (AFE), and multiple-exon splicing (MSE), have also been identified [3, 4]. AS plays a crucial role in various physiological and pathological processes such as embryonic development [5], aging [6], and tumor progression [7, 8]. Several key splicing factors are involved in the regulation of AS during disease progression. For instance, RBFOX2 is a master regulator for mesenchymal tissue-specific splicing [9], playing a significant role in the formation of mesenchymal-like states in tumor cells [10]. Recently, single-cell transcriptomics has become a powerful tool for analyzing profiles of AS at high resolution [11, 12]. Several recent studies have expanded our understanding of cell type–specific splicing programs. For example, Huang et al. [13] revealed that subtype-specific splicing patterns refine the classification of pituitary neuroendocrine tumors (PitNETs). Joglekar et al. [14] constructed a cross-species isoform atlas, demonstrating conserved and human-specific splicing programs linked to neurodevelopment and disease. Similarly, Lukacsovich et al. [15] showed that neurexin isoforms are cell type specific and developmentally stable in the brain, underpinning synaptic identity. Together, these studies underscore the biological relevance and regulatory specificity of AS in health and disease, motivating the development of methods like AEnet for systematic analysis of AS at a single-cell resolution [16].

Although several computational methods have been developed to analyze alternative splicing at the single-cell level, most rely on predefined cell types based on canonical gene expression clustering. These methods typically assess splicing heterogeneity through comparisons between expression-defined groups, in a manner analogous to differential gene expression analysis (Supplementary Table S1). However, cell types defined by AS can differ substantially from those defined by gene expression, which may result in incomplete detection of splicing heterogeneity. This limitation is evident in tools such as BRIE [17], Outrigger [18], and our earlier method, DESJ detection [19]. Furthermore, existing single-cell analysis tools lack the extensibility to reveal the regulatory mechanisms of alternative splicing, infer its regulatory factors, and identify functional pathways of specific isoforms—rather than merely those of differentially spliced genes (Supplementary Table S1). MARVEL [20] addresses this limitation by performing Gene Ontology (GO) enrichment analysis using the clusterProfiler R package, which can identify enriched pathways among differentially spliced genes. However, this approach only indirectly infers the potential functional relevance of AS events and does not directly assess the role of individual isoforms produced via alternative splicing, thus limiting an in-depth understanding of how alternative splicing influences cells and cell states [21, 22].

Several issues require further consideration in the design of a single-cell AS analytical pipeline. First, unlike RNA expression, which is typically quantified in absolute values, AS events are usually quantified as proportional values. The sparsity of single-cell RNA sequencing (scRNA-seq) data often introduces the “not a number (NaN)” challenge during calculations when the denominator (the total number of AS events for a given gene in a single cell) is zero [23]. Second, AS events can also be affected by batch effects, an inherent limitation of single-cell techniques [24]. Third, not all AS events contribute functionally to cellular heterogeneity [25]. Therefore, both upstream and downstream approaches that account for filtering processes should be incorporated.

To address these challenges, we present the Alternative Splicing-Gene Expression Network (AEnet) to explore core AS events and gene coexpression patterns in a network at the single-cell level. Using our algorithm, we find that both splice site preferences and gene expression contribute to cellular heterogeneity during clustering, though they exhibit dynamic interplays and varying weights across different datasets. We refer to the separated clusters as cell (sub)populations to avoid confusion with either cell types or cell states. The software has 3 major functions: first, to construct AS profiling-based clusters and separate cell subpopulations with distinct AS–gene expression networks; second, to identify key splicing factors for AS clusters (analogous to gene markers); and third, to pinpoint functional pathways involved in the regulatory mechanisms based on core subsets of AS events.

By applying the AEnet method to malignant cells with different immunotherapy responses, T-cell analysis in pan-cancer, and cell differentiation during embryonic gastrulation, we demonstrate the power of AEnet in fine-grained clustering of cells by disease or developmental states, seamlessly linking upstream regulatory factors and downstream action pathways, highlighting novel isoforms of functional importance, and constructing alternative splicing landscapes along the AS-based developmental trajectory. These findings will deepen our understanding of the role of alternative splicing in tumorigenesis and embryonic development, providing new strategies and ideas for clinical prognosis prediction, tumor immunotherapy, and congenital disease treatment.

## Results

### The overview of AEnet

**Figure 1:**
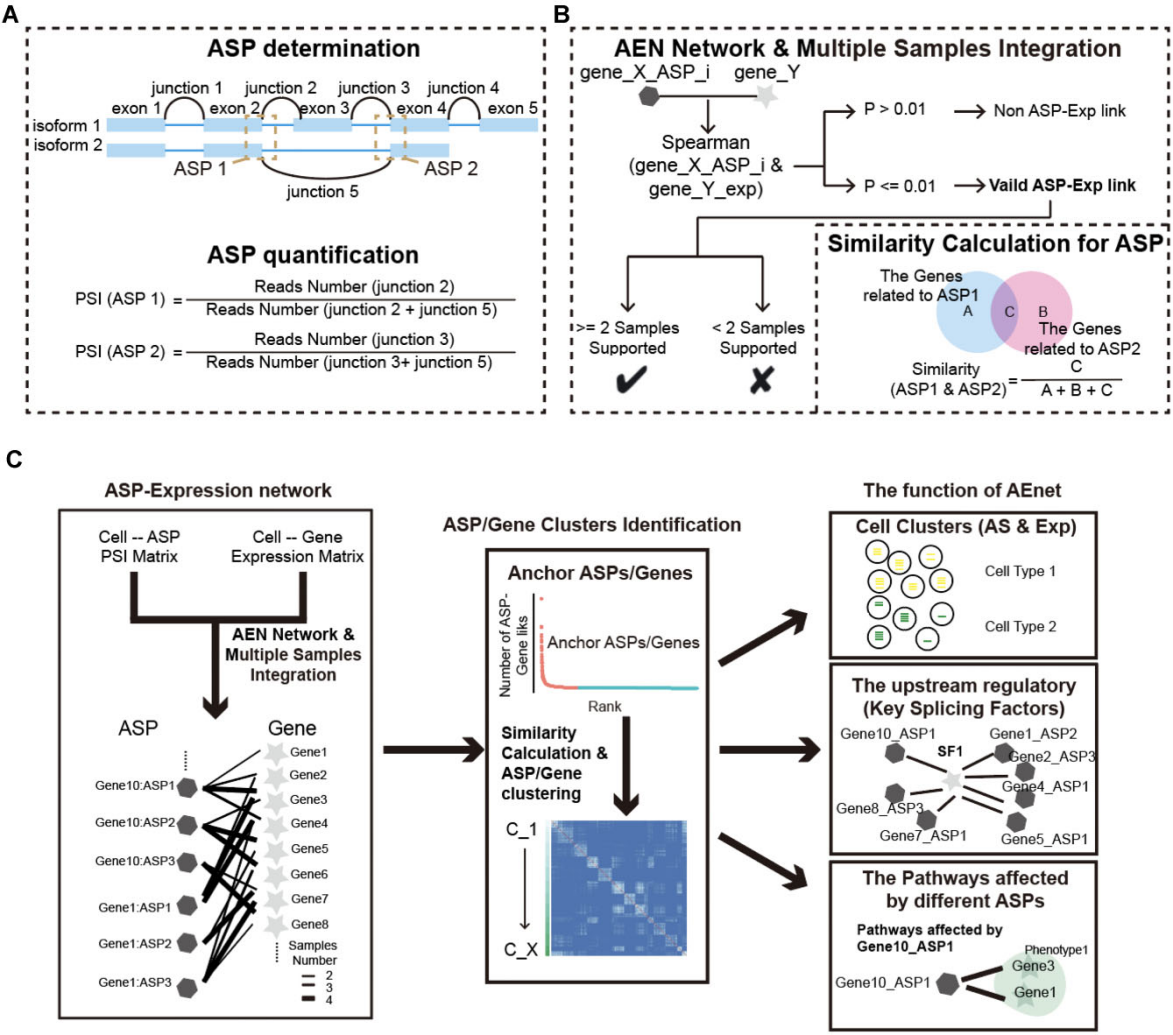
Schematic diagram of the AEnet method. (A) Schematic diagram of the identification of an alternative splicing pattern (ASP) and the calculation of PSI. (B) Schematic diagram of AEnet network construction, multiple sample integration, and ASP similarity calculation. (C) Scheme of AEnet methods.

We have developed the AEnet method that integrates alternative splicing with gene expression levels to uncover cellular splicing heterogeneity and underlying regulatory mechanisms. In brief, AEnet begins by quantifying alternative splicing patterns (ASPs) using junction reads from individual cells during data preprocessing (Fig. 1A). The percent spliced-in (PSI) for a specific AS event is defined as the proportion of junction reads curated from all detected junctions that span the same site (Fig. 1A). Notably, unlike expression values, which are always nonnegative integers, PSI is assigned as NaN (resulting from division by 0) when no junction reads are detected at a presupposed AS site in a cell (Supplementary Fig. S1A). In scRNA-seq data, the prevalent RNA dropout and shallow sequencing issues make the occurrence of NaN even more challenging within individual cells.

To mitigate this effect, for each ASP and each gene, we limit the calculation of the correlation (Spearman by default) to cells with valid PSI values and the gene’s expression level, retaining only those ASP–expression correlation links (referred to as ASP–EXP links) with significant *P* values (Fig. 1B). This filtering step ensures that only statistically robust associations are retained, as these statistically significant links indicate potential relationships between gene expression dynamics and the usage preference of specific ASPs across the cells. When multiple scRNA-seq samples are available under any experimental conditions, we retain only ASP–EXP links that share the same correlation trend and appear at a moderately higher frequency (2 by default) to exclude batch effect–induced artifacts (Supplementary Figs. S1B, C). These steps allow AEnet to overcome technical noise and capture common relationships between AS and gene expression.

We hypothesize that the interactions identified in common ASP–EXP links play either direct or indirect roles in posttranscriptional regulation and gene expression diversity. For instance, increased expression of specific splicing factors may promote or inhibit the inclusion of certain exons, thereby modifying the AS profile of target genes (Supplementary Fig. S1D) [26]. Additionally, distinct isoforms are associated with the expression dynamics of multiple genes and influence specific signaling pathways, ultimately contributing to changes in cell state (Supplementary Fig. S1E). In other cases, both alternative splicing and gene expression levels may be dysregulated, leading to aberrant gene function during stress responses or in diseases [27].

To prioritize splicing events with broad regulatory influence, AEnet ranks ASPs based on the number of associated ASP–EXP links. The top 1,500 ASPs (by default) are selected as anchor ASPs. Pairwise similarities between anchor ASPs are then computed using the Jaccard index of their associated gene sets, resulting in an ASP similarity matrix used for clustering (Supplementary Fig. S1F–G). In parallel, a gene similarity matrix is constructed to define anchor genes, which are similarly clustered into gene modules (Supplementary Fig. S1H). These clusters represent coregulated gene sets and splicing programs.

Taken together, the AEnet pipeline begins with the detection of ASPs at the cellular level, followed by the construction of ASP (of gene i)–expression (of gene j) links (ASP–EXP links), and the generation of the ASP similarity matrix at the sample level. Ultimately, it uncovers specific ASPs and coexpression/regulatory patterns at the cell population level (Fig. 1C, Supplementary Fig. S2A). The output of AEnet focuses on evaluating 3 main biological events (Fig. 1C, Supplementary Fig. S2B–D): first, the separation of cell subpopulations with distinct ASP compositions; second, the key splicing factors that influence specific ASP clusters (analogous to gene markers), identified based on a predefined list of splicing factors (Supplementary Table S2); and third, the functional pathways activated or inhibited by individual or small subsets of ASPs. These downstream analyses demonstrate AEnet’s capability in identifying cellular splicing heterogeneity and regulatory mechanisms.

### Evaluation of AEnet for comprehensive characterization of splicing heterogeneity

We next evaluated AEnet across multiple analytical steps, including ASP–EXP network construction (comprising ASP identification, ASP quantification, and the prediction of ASP and gene clusters), and cell clustering (Supplementary Fig. S3A). A total of 4 datasets were used for this evaluation, each accompanied by published annotations serving as ground truth. These datasets, including induced pluripotent stem cell (iPSC) [18], hepatocellular carcinoma (HCC) [28], and T-cell datasets [29], are described in detail in Supplementary Table S3.

We first evaluated the performance of AEnet in the ASP identification (Supplementary Fig. S3A–C). Current methods, such as BRIE, Outrigger, and MARVEL, are all fundamentally annotation-dependent and therefore unable to detect unannotated AS events. Additionally, these tools are limited in their ability to detect MSEs (Supplementary Fig. S3B, Supplementary Table S4). Since MARVEL has been compared with other methods and demonstrated to be the optimal one in its category in a previous study, we next compared the AS events detected by AEnet and MARVEL using the demo dataset of MARVEL, which includes iPSCs and iPSC-derived endoderm cells (Supplementary Fig. S3C) [30]. MARVEL identified a total of 20,509 SEs, 1,279 MXEs, 8,295 RIs, 5,163 A5SSs, 5,832 A3SSs, 5,818 AFEs, 2,072 ALEs, and 0 MSEs (Supplementary Fig. S3D). In MARVEL’s iPSC dataset, AEnet detected 722,278 additional AS patterns (63% unannotated) across 12,866 genes, with MSE detection 3.2-fold higher than MARVEL (Supplementary Fig. S3E, F). These findings highlight AEnet’s comprehensive capability to detect a wide range of AS events—including unannotated and complex patterns—except for intron retention events. Compared to our homologous method, DESJ detection, AEnet overcomes critical limitations in low-depth robustness. DESJ detection’s single-junction PSI calculation leads to 38% error rates in low-coverage scenarios (Supplementary Fig. S3G, H). In contrast, AEnet mitigates this by focusing on junction reads with shared splice sites (requiring ≥5 supporting reads) and transforming read distributions into transcript usage ratios. This strategy reduced PSI error by 47% (*P* < 0.001, Supplementary Fig. S3I). Taken together, AEnet outperforms existing methods in detecting unannotated alternative splicing events, resolving complex AS patterns, and ensuring reliable quantification across varying data depths.

Low-count ASPs may introduce random fluctuations in PSI estimates, potentially leading to inaccurate quantification, which in turn can affect the construction of ASP–EXP links and the identification of anchor ASPs (Supplementary Fig. S4A). To evaluate the impact of count thresholds on these outcomes, we systematically tested a range of minimum read count thresholds: 0, 3, 5, 7, and 9 (Supplementary Fig. S4A). Using a threshold of 0 yielded the highest number of ASP–EXP links, with ∼40% classified as “specific.” However, 99% of these specific links were supported by fewer than 2 samples, suggesting they were likely artifacts of random fluctuations rather than meaningful biological associations (Supplementary Fig. S4B, C). Similarly, the 0-read threshold also led to an inflated number of anchor ASPs, most of which were linked to low-confidence, sample-specific signals (Supplementary Fig. S4D). In contrast, applying thresholds of ≥3 substantially reduced these spurious associations. Importantly, most biologically meaningful ASP–EXP links were retained even when moderate thresholds (>0) were applied (Supplementary Fig. S4E). Based on these results, AEnet uses a default threshold of 5 supporting reads to ensure PSI robustness while minimizing noise.

While filtering low-count ASPs improves analytical reliability, we also considered the potential risk of excluding rare but biologically relevant splicing events. To address this, AEnet defines an ASP as “valid” in a sample only if it is supported by ≥5 reads across ≥20 cells. ASPs failing this criterion are excluded from downstream analysis, as their sparsity compromises the reliability of similarity estimates between splicing and expression. To assess whether this filtering excludes informative low-abundance ASPs, we stratified all ASPs into 5 categories based on their support across cells: invalid, ≤20 cells (excluded); type 1, >20–30 cells; type 2, >30–40 cells; type 3, >40–50 cells; and type 4, >50 cells. An ASP was assigned to the highest applicable category if it met the criteria in ≥3 samples (Supplementary Fig. S4F). As expected, higher-support ASPs (fewer NaNs) showed stronger ASP–EXP associations. Nonetheless, ∼30% of type 1 ASPs (i.e., relatively rare but retained) still showed significant correlations with gene expression, and 4 were identified as anchor ASPs (Supplementary Fig. S4G, H), indicating their functional relevance. In summary, our results support the use of both read count and sample support thresholds to reduce noise while preserving biological signal. AEnet remains capable of capturing meaningful but infrequent ASPs and provides user-defined thresholding to support flexible analysis tailored to specific research goals.

Finally, to assess the performance of AEnet in the identification of ASP clusters, an ASP–ASP similarity (Jaccard index) matrix was generated with increasing levels of noise to evaluate AEnet’s effectiveness in ASP cluster prediction (Supplementary Fig. S5A, B). Using a supervised hierarchical clustering method, AEnet demonstrated a high accuracy consistency score of approximately 0.9 between the background and the clusters identified, even when noise levels reached 80% (Supplementary Fig. S5C, D). These results demonstrate AEnet’s robustness in identifying ASP clusters despite noise. Furthermore, we evaluated AEnet’s performance using more realistic noise models, specifically Gaussian and Poisson noise. We simulated increasing levels of both Gaussian and Poisson noise and generated ASP–ASP similarity matrices (Jaccard index) under each noise condition to assess AEnet’s robustness in ASP clustering (Supplementary Fig. S6A–B, D–E). Using a supervised hierarchical clustering approach, AEnet consistently achieved a high accuracy score—approximately 0.9—between the ground truth and the predicted clusters, even under noise levels as high as 90% for both noise models (Supplementary Fig. S6C, F). These results demonstrate AEnet’s resilience to biologically relevant noise, further supporting its reliability in identifying splicing patterns in realistic, noisy settings.

### Benchmarking AEnet against existing methods

**Figure 2:**
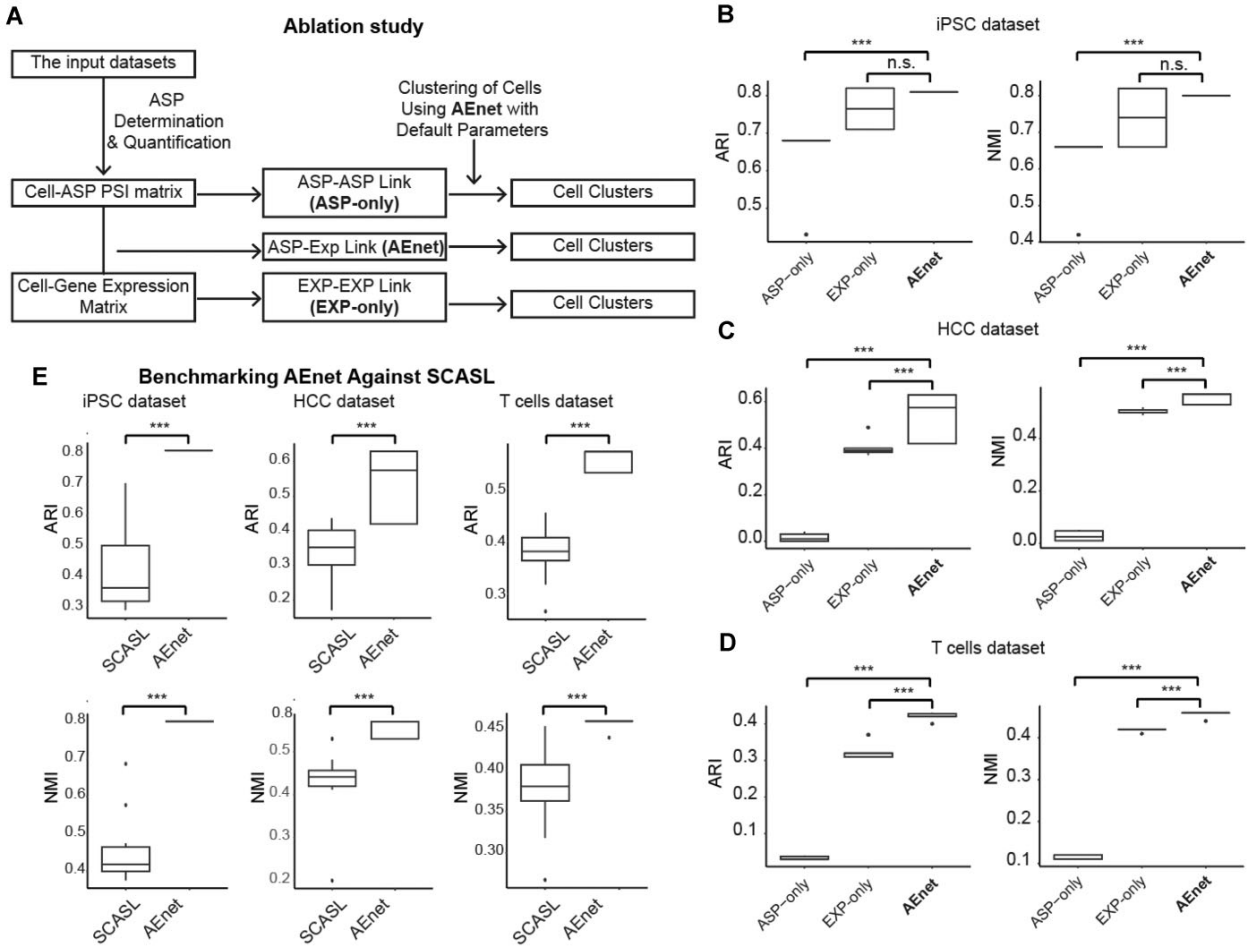
Benchmarking AEnet against existing methods. (A) Schematic diagram of the assessment pipeline for ASP-only, RNA-only, and joint clustering analyses, with all other processing steps remaining identical to those in AEnet. (B–D) Quantitative benchmarking of clustering concordance using (upper) Adjusted Rand Index (ARI) and (lower) normalized mutual information (NMI) metrics across the 3 networks. Statistical significance was assessed via 1-sided Wilcoxon rank-sum tests. (E–G) Quantitative benchmarking of clustering concordance using (left) ARI and (right) NMI metrics of AEnet and SCASL. Statistical significance was assessed via 1-sided Wilcoxon rank-sum tests. *P < 0.05, **P < 0.01, ***P < 0.001; n.s., not significant.

While the above analyses validate AEnet’s basic performance in characterizing splicing heterogeneity, its practical value in the field requires comparison with existing state-of-the-art methods. We thus benchmarked AEnet against established tools for single-cell AS analysis. We next evaluated AEnet’s performance in capturing cellular heterogeneity using the 3 benchmarking datasets. Comprehensive ablation analyses, using published cell-type annotations as ground truth, demonstrated that the joint ASP–EXP model (AEnet) consistently outperformed the standalone AS-only (Anet) and expression-only (Enet) approaches (Fig. 2A). Specifically, the median Adjusted Rand Index (ARI) scores were 0.81 (AEnet), 0.68 (Anet), and 0.77 (Enet) for the iPSC dataset; 0.58 (AEnet), 0.10 (Anet), and 0.39 (Enet) for the HCC dataset; and 0.42 (AEnet), 0.10 (Anet), and 0.32 (Enet) for the T-cell dataset (Fig. 2B–D). These results highlight that integrating ASP features with gene expression significantly enhances clustering resolution and biological interpretability. Moreover, AEnet produced the most informative low-dimensional embeddings across all datasets (Supplementary Fig. S7), accurately reconstructing the cellular architecture in iPSCs, delineating major lineages in HCC, and resolving functionally distinct T-cell subsets—tasks in which AS-only or EXP-only models performed suboptimally.

We further compared AEnet with established splicing-aware clustering methods, including SCASL [31] and scQuint [32]. Across all 3 datasets, AEnet consistently outperformed SCASL in clustering accuracy, with higher ARI scores: iPSC (0.81 vs. 0.37), T cell (0.42 vs. 0.29), and HCC (0.58 vs. 0.35) (Fig. 2E–G). In the iPSC dataset, SCASL failed to distinguish iPSCs from Neuron Progenitor Cells (NPCs), while AEnet clearly separated these populations (Supplementary Fig. S8A). In the HCC dataset, AEnet effectively resolved lymphoid, myeloid, and malignant epithelial lineages, whereas SCASL showed poor separation between immune cell types (Supplementary Fig. S8B). Similarly, in the T-cell dataset, SCASL generated overlapping clusters, failing to delineate functional T-cell subsets, in contrast to the well-separated clusters produced by AEnet (Supplementary Fig. S8C). Since scQuint primarily uses a variational autoencoder (VAE) to generate embeddings, we applied clustering to the scQuint-derived embeddings using default parameters. In the iPSC dataset—characterized by relatively simple cellular composition—AEnet and scQuint showed comparable performance. However, in the more complex T-cell and HCC datasets, scQuint produced overlapping clusters and failed to resolve key subpopulations (Supplementary Fig. S8D). Together, these results confirm that joint modeling of alternative splicing and gene expression enables AEnet to more accurately capture cellular heterogeneity compared to other splicing-based methods.

We next assessed AEnet’s performance in mitigating batch effects and identifying AS-driven cell heterogeneity. Compared to SCASL—the current leading single-cell clustering tool based on AS—AEnet showed improved robustness across multiple samples (Supplementary Fig. S9). In lung cancer datasets [33], AEnet uncovered shared AS heterogeneity across patients, whereas SCASL mainly reflected patient-specific batch effects (Supplementary Fig. S9A, B). Similarly, in the colorectal cancer (CRC) [29] and recurrent hepatocellular carcinoma (RHCC) [28] T-cell datasets, SCASL failed to detect AS heterogeneity in T cells with minimal batch effects, while AEnet successfully captured these patterns (Supplementary Fig. S9C, D). These results demonstrate AEnet’s ability to detect biologically meaningful AS heterogeneity, even in the presence of technical noise or batch variation, making it well suited for large-scale, multisample studies.

To further demonstrate AEnet’s versatility, we applied it to a widely used 10x Genomics peripheral blood mononuclear cell (PBMC) dataset (Supplementary Fig. S9E, G) [34]. Due to the limited sequencing depth of 10x platforms, AEnet detected insufficient splicing events for robust AS-based analysis. We therefore focused on alternative polyadenylation (APA), another form of isoform regulation. In this dataset, AEnet identified 190 anchor APA events and defined 7 APA-based cell clusters (Supplementary Fig. S9E). These clusters aligned well with known immune cell types, including B cells, CD4⁺ T cells, CD8⁺ T cells, dendritic cells, macrophages, mast cells, monocytes, neutrophils, natural killer (NK) cells, and regulatory T cells (Supplementary Fig. S9F). Clustering performance was quantitatively supported by a median ARI of 0.83 and normalized mutual information (NMI) of 0.78 (Supplementary Fig. S9G). These results highlight AEnet’s ability to uncover isoform-level heterogeneity in shallow-depth datasets, although we note that AS-based analyses remain more challenging in such settings due to limited read coverage.

### AEnet resolves tumor heterogeneity bias and identifies immunotherapy-nonresponsive tumor subpopulations

Due to inherent intra- and intertumor heterogeneity, grouping malignant cells solely based on either gene expression profiles or ASPs is challenging. Here, we showcase the power of AEnet in untangling the intricate ASP–EXP relationships using data from 1,286 tumor cells from 6 patients with lung cancer with varying responses to immunotherapy, classified as normal (N), residual disease (RD), and progressive disease (PD) after therapy [33].

**Figure 3:**
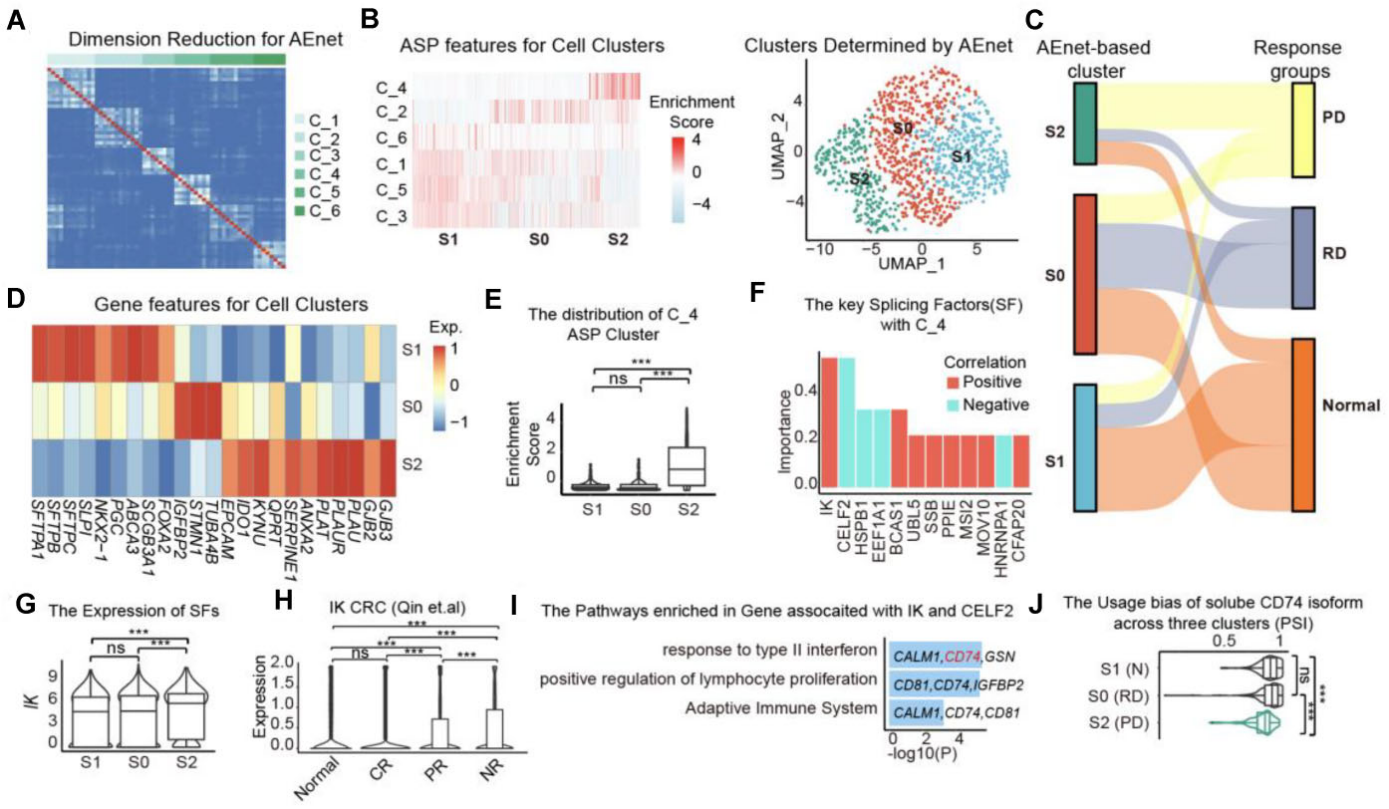
AEnet decreases the bias caused by tumor heterogeneity and uncovers mechanistic insights in immunotherapy response. (A) The heatmap shows the ASP clusters from dimension reduction of AEnet. (B) The heatmap displays the enrichment score of ASP clusters across cell clusters determined by AEnet (left). UMAP displays clustering of cell types determined by AEnet (right). (C) The Sankey plot shows the overlapping of cells between response groups and clusters determined by AEnet. (D) The heatmap displays the expression of marker genes across cell clusters determined by AEnet. (E) The distribution of C_4 enrichment scores across cell clusters determined by AEnet. (F) The barplot displays the importance of splicing factors in the formation of C_4 ASP clusters. The color represents the correlation relationship between splicing factors and C_4 ASP clusters. (G) The expression of IK across cell clusters. Statistical analysis was performed using Student’s *t* test. (H) The expression of IK within cells at different response groups. Statistical analysis was performed using Student’s *t* test. (I) The top 3 pathways enriched for the genes with alternative splicing patterns in C_4 ASP clusters. (J) The alternative splicing patterns of CD74 and the PSI distribution across 3 clusters. Statistical analysis was performed using Student’s *t* test. *P < 0.05, **P < 0.01, ***P < 0.001.

Based on the AEnet algorithm, the ASP similarity matrix revealed a distinct separation into 6 ASP clusters, which resulted in 3 cell subpopulations (Fig. 3A, B). The AEnet-defined clusters exhibited biased distribution across response groups, with PD dominated by S2, RD primarily comprising S0 cells, and the N group containing the majority of S1 cells (Fig. 3C). The marker genes of S1 (N) were enriched in alveolar signatures, including *AQP4, SFTPB/C/D, NKX2-1*, and *FOXA2* [35, 36], while S2 (PD) was associated with elevated expression of prothrombin activation genes (*PLAT, PLAUR*), gap-junction proteins (*GJB2/3/*5), and the well-known epithelial-mesenchymal transition (EMT) marker *EPCAM* (Fig. 3D) [37–39].

From the perspective of ASP clusters, C_4 (ASP cluster 4) was notably co-occurring with S2 while being excluded from S1 (Fig. 3E). The top-ranked hub genes in this cluster were IK and CELF2, which exhibited opposite expression trends between normal and PD cells (Fig. 3F, G). CELF2 is a crucial splicing factor, and its downregulation has been reported to promote tumor progression in both pancreatic and breast cancers [40, 41]. The role of IK remains unclear; however, we found it to be upregulated in a CRC cohort as responses to immunotherapy deteriorated (Fig. 3H) [42].

In addition, C_4 was enriched in inflammation-associated pathways, including the response to type II interferon, positive regulation of lymphocyte proliferation, and the adaptive immune system (Fig. 3I). Among the key genes related to inflammatory responses, CD74, the HLA-DR antigen-associated invariant chain, is reported to exhibit dual oncogenic and tumor-suppressive roles depending on the cancer type and specific microenvironment. In this lung cancer dataset, we found that ASPs in CD74 were exclusively dominated by the isoforms CD74-201 and CD74-202. CD74-202 is reported as the soluble form, which suppressed melanoma cell growth and induced apoptosis under IFN-γ stimulatory conditions [43, 44]. The significantly differentiated ratio of CD74-202 to CD74-201 could directly indicate posttherapy responses in different cell groups (Fig. 3J).

### AEnet reveals cellular splicing heterogeneity and its key splicing events in tumor-infiltrating T cells across various cancer types

The design of the AEnet algorithm largely bypasses batch effect issues in scRNA-seq analyses, making it particularly suitable for pan-cancer studies. To explore the cross-tissue capacity of this “AS-Expression Network” concept, we curated tumor-infiltrating lymphocyte T cells from 4 cancer types: liver cancer (HCC) [45], CRC [29], lung cancer (LUAD) [33], and RHCC [28].

**Figure 4:**
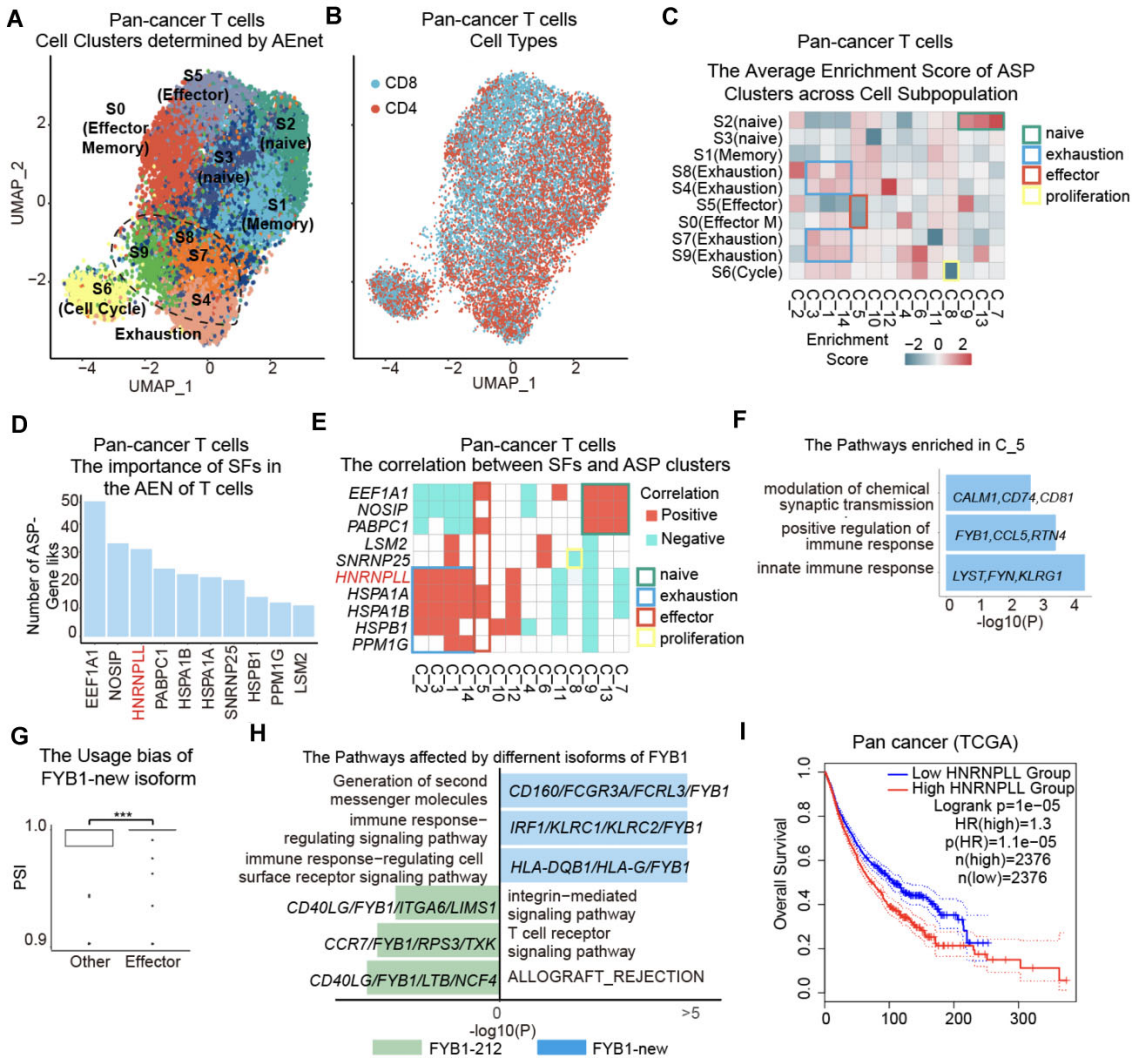
Application of the AEnet to pan-cancer tumor-infiltrating T-cell single-cell data. (A, B) UMAP displays clustering of cell types determined by AEnet (A) and lineage (B). (C) The heatmap displays the enrichment score of ASP clusters across cell clusters determined by AS. (D) The barplot displays the importance of splicing factors in the formation of the AEN network of the pan-cancer T cells. (E) The relationship between ASP clusters and key splicing factors. (F). The top 3 pathways enriched for the genes with alternative splicing patterns in C_5 ASP clusters. (G) The PSI distribution of the FYB1-new isoform within FYB1 across effector and other T cells. Statistical analysis was performed using Student’s *t* test. (H) The pathways enriched in the gene sets with different isoforms of FYB1. (I) Kaplan–Meier analysis shows the overall survival of patients characterized by low (blue) or high (red) HNRNPLL in The Cancer Genome Atlas cohort. Statistical analysis was performed using the log-rank test. *P < 0.05, **P < 0.01, ***P < 0.001.

By selecting the top 1,223 ASP events from a total of 86,616 valid ASP–EXP links (supported by at least 2 samples across a minimum of 2 datasets), we identified 14 ASP clusters (referred to as C1–C14) (Supplementary Fig. S10A–C). We subsequently generated 10 cell subpopulations (referred to as S0–S9) based on distinct compositions of ASP cluster-wise signatures across the 4 T-cell datasets (Fig. 4A, Supplementary Fig. S10D). Among these populations, S2 and S3 expressed naive T-cell markers (*IL7R, CCR7, LEF1*); S1 was characterized by memory T-cell markers (*CD52, ANXA1, CREM*); S0 and S5 exhibited effector T markers (*NKG7, GZMA/B*); S4, S7, S8, and S9 represented the exhausted state of T cells (*PDCD1, CTLA4, HAVCR2*); and S6 corresponded to the proliferative state (*MKI67, TOP2A*) (Supplementary Fig. S10E) [46]. Strikingly, AEnet did not partition T cells into canonical CD4^+^ and CD8^+^ subtypes, suggesting that alternative splicing primarily contributes to the transition of cell states rather than defining cell lineage (Fig. 4B).

Cross-cluster interactions were observed in our data (Supplementary Fig. S10C). To further explore these relationships, we organized ASP clusters based on pairwise similarity and identified 3 major regions, each comprising clusters with higher intraregion similarity than interregion similarity (Supplementary Fig. S10F). Region 1 includes clusters C_7 and C_9, which exhibit the highest mutual similarity and are both predominantly enriched in naive T cells. Interestingly, C_7 also shares similarity with C_5 and C_10, while C_9 is more closely related to C_4 and C_11. This suggests that C_7 and C_9 may represent bifurcating points leading to 2 naive T-cell differentiation trajectories—one toward memory T cells (C_5 and C_10) and the other toward effector T cells (C_4 and C_11) (Supplementary Fig. S10H–J). Supporting this, C_7 is enriched during the naive-to-memory transition, while C_9 is enriched along the naive-to-effector axis (Supplementary Fig. S10G). Region 2 comprises clusters C_11, C_4, and C_14, which show strong intercluster similarity. C_4 acts as a central node, connecting C_11 and C_14, suggesting a potential progression from effector T cells (C_11) to effector memory T cells (C_4), and eventually to exhausted T cells (C_14). Notably, C_11 also shares similarity with C_9, and C_14 with C_1—both associated with exhausted states—indicating a continuous exhaustion trajectory (Supplementary Fig. S10H). Region 3 implies an alternative exhaustion pathway, involving clusters C_5, C_10, and C_12. Here, C_10 links C_5 (memory/naive-enriched) and C_12 (exhausted-enriched), forming a sequence akin to that in region 2. Similarity between C_5 and C_7, as well as between C_12 and C_1, further supports a parallel differentiation path from naive/memory T cells to exhaustion (Supplementary Fig. S10I). In summary, while rigid clustering provides discrete groupings, our similarity-based regional analysis reveals underlying transitions and trajectories among ASP-defined clusters. These findings highlight the dynamic continuum of T-cell state transitions and underscore the value of complementary methods in capturing intermediate cell states that may be overlooked by strict partitioning.

When focusing on ASP clusters, the exclusive deficiency of C_5 in effector T cells (S0 and S5) and C_8 in proliferating T cells (S6) were 2 notable signatures demonstrating the correlation between specific ASPs and cell population heterogeneity (Fig. 4C). Among the top 10 common splicing factors, the C_5 ASP cluster positively correlated with EEF1A1 and PABPC1, which were partially aligned with ASPs primarily occurring in naive T cells (clusters C_7, C_9, and C_13) (Fig. 4D, E). In contrast, C_8 was negatively correlated with small nuclear ribonucleoprotein 25 (SNRNP25), a splicing factor involved in spliceosome assembly and function, intron excision, and exon ligation [47]. The upregulation of SNRNP25 (reflected as a double-negative correlation) in proliferating T cells served as a clear marker, identifying this cell type solely through splicing factors (Supplementary Fig. S10K).

C_5 was also correlated with HSPA1A/B, splicing factors contributing to exhausted T cells (C_1, C_2, C_3, C_12, and C_14). To explore the functional implications of C_5, we performed pathway enrichment analysis on its hub genes (Fig. 4F). This revealed a previously unannotated splicing variant of FYB1 (referred to as FYB_new), which utilizes chr5_39,217,636 as the end of the first exon of FYB1—a site not documented in the GRCh38.p14 reference genome. This splicing variant is associated with pathways involved in second-messenger generation, immune response–regulating signaling, and cell surface receptor signaling, all critical processes for achieving effector cell status (Fig. 4G, H). In contrast, FYB1-212 was associated with pathways linked to naive and memory T cells.

Among the top splicing factors, HNRNPLL ranked third and exhibited a strong positive correlation with ASP clusters associated with exhausted T cells. It was also linked to unfavorable clinical prognosis (Fig. 4I). Genes regulated by HNRNPLL were enriched in thymic T-cell selection, axon guidance, and leukocyte activation (Supplementary Fig. S10L). Among the hub genes, CD3D, the most canonical T-cell marker, exhibited 2 isoforms with distinct PSI distributions between exhausted and other T cells (Supplementary Fig. S10M). Exhausted T cells preferentially spliced into isoform CD3D-202, which is linked to the PD-1 signaling pathway, CD28 family-mediated costimulation, and TCR signaling, while other T cells primarily utilized CD3D-201 for differentiation (Supplementary Fig. S10N).

Collectively, we demonstrate the capability of the AEnet algorithm for integrated scRNA-seq data analysis without the need for batch corrections. Our algorithm facilitates bioinformatic data mining for isoform usage preferences and even the discovery of novel isoforms of functional importance, such as new ASPs in the FYB gene for effector T cells.

### AEnet uncovers transitional cell states and key splicing factors in embryonic gastrulation

During embryogenesis, alternative splicing is a key mechanism that fine-tunes developmental pathways and controls cell fate decisions. Here, we apply AEnet to interrogate a scRNA-seq dataset of gastrulation-stage human embryos from the Human Developmental Biology Resource, elucidating how the AS process enables precise regulation of gene expression at this stage. The reference dataset comprises 1,195 cells (665 caudal, 340 rostral, and 190 yolk sac cells), with a median of 4,000 genes detected per cell [48].

**Figure 5:**
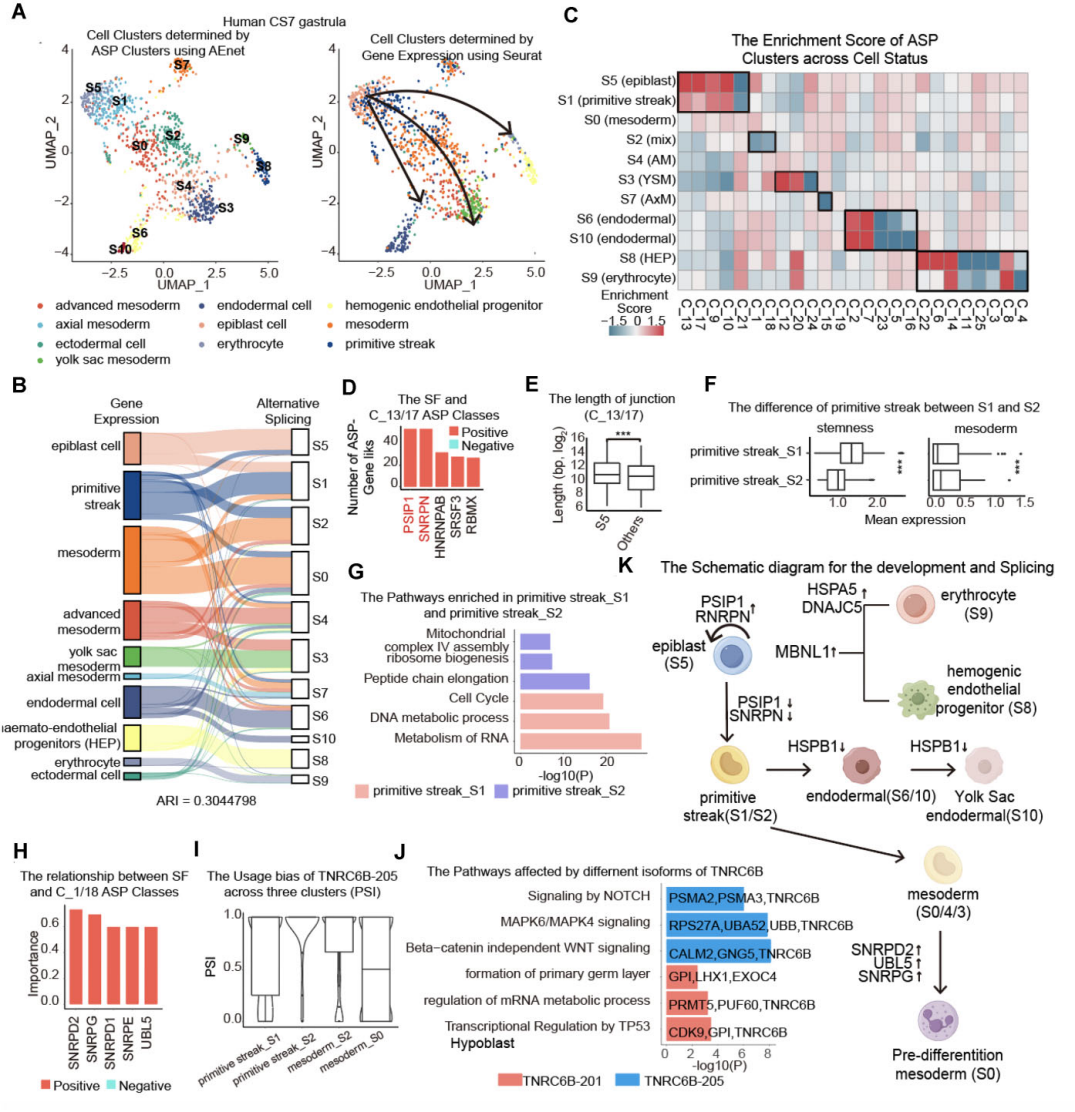
AEnet uncovers transitional cell states and key splicing factors in embryonic gastrulation. (A) UMAP displays clustering of cells by cell type determined by alternative splicing (left) and gene expression (right). (B) The overlapping of cells between clusters determined by alternative splicing and gene expression. (C) The heatmap displays the enrichment score of ASP clusters across cell clusters determined by AS. (D) The barplot displays the importance of splicing factors in the formation of C_13/17 ASP clusters. The color indicates the relationship between SF and ASP clusters. (E) The length of junction that is differentially used in different cell types. Statistical analysis was performed using Student’s *t* test. (F) The boxplot displays the expression of stemness and mesoderm markers across primitive streak cell subsets determined by AS and expression. Statistical analysis was performed using Student’s *t* test. (G) The pathways enriched in the upregulated genes in the primitive streak cell subsets determined by AS and expression. (H) The barplot displays the importance of splicing factors in the formation of C_1/18 ASP clusters. The color indicates the relationship between SF and ASP clusters. (I) The alternative splicing patterns of TNRC6B and the PSI distribution. (J) The pathways enriched in the gene sets with different isoforms of TNRC6B. (K) The illustration depicts the developmental trajectory and highlights the top-ranked key splicing factors based on their relative importance. *P < 0.05, **P < 0.01, ***P < 0.001.

Following the standard AEnet workflow, we first identified a total of 1,604 ASP events, 25 ASP clusters, and 11 cell populations (Fig. 5A, Supplementary Fig.  S11A–C). The sequential differentiation trajectory from epiblast cells (cell subpopulation 5, S5) to the primitive streak (S1), followed by the transition to endodermal cells (S6/S10) or mesoderm (S2/S0), ultimately leading to axial mesoderm (S7), was clearly discernible (Fig. 5A, right) [49]. Notably, the ASPs are assumed to be highly distinct among different cell types during embryogenesis. As a result, AEnet factorized the cell populations in a manner similar to those clustered based solely on RNA profilings (Fig. 5B, ARI = 0.304).

Epiblast cells (S5) are classic pluripotent stem cells, derived from the inner cell mass of the blastocyst and capable of differentiating into the 3 germ layers. For the highly correlated ASP clusters C_13 and C_17, PSIP1 and SNRPN were identified as key regulatory factors that maintain cell stemness (Fig. 5C, D, Supplementary Fig. S11D, E). HNRNPAB and SRSF3 were associated with splicing decisions that resulted in longer junctions spanning genomic loci, observed more frequently in epiblast cells than in cells with reduced stemness (Fig. 5E). We verified this intriguing finding in an independent iPSC dataset (Supplementary Fig. S11F) [18].

Some subpopulations defined by AEnet were in a transitional stage. For example, S2 shares RNA profiling similarities with both S1 and S0 (Fig. 5B). This cell population expressed relatively lower stemness signatures and higher mesodermal features compared to the primitive streak (S1) (Fig. 5F). The enriched pathways also indicated that S2 was an intermediate cell state between the primitive streak (S1) and mesoderm (S0) (Fig. 5G). We subsequently compared the ASP cluster compositions between S2 and S0, focusing on 2 of the most distinct ASP clusters, C_1 and C_18, for downstream analysis (Fig. 5C). As part of the routine analysis, we identified the top splicing factors and hub gene–enriched pathways. SNRPD2, a core component of the spliceosome, emerged as a pivotal factor distinguishing these 2 mesoderm subtypes (Fig. 5H, Supplementary Fig.  S11G). Related to the genes in enriched pathways, TNRC6B displayed distinct isoform distributions across cell clusters (Fig. 5I, Supplementary Fig. S11H). TNRC6B-205 was enriched in S2 and linked to the classical WNT, Notch, and MAPK signaling pathways. In contrast, TNRC6B-201 was prevalent in other cell types and associated with primary germ layer formation and other processes (Fig. 5J).

Moreover, we unveiled that ASP clusters C_2, C_5, C_7, C_16, and C_23 were pivotal for endoderm differentiation, with HSPB1 emerging as a core negative regulator of their formation. Clusters C_12, C_20, and C_24, crucial for yolk sac mesoderm development, were positively regulated by HSPB1 (Supplementary Fig. S11I, J). Lastly, ASP events in hepatic and erythrocyte development (C_3, C_4, C_6, C_8, C_11, C_14, C_22, and C_25) were governed by MBNL1 and HSPA5, both highly expressed in these cell types (Supplementary Fig. S11I, J).

By exploring the alternative splicing landscapes in this human embryonic data (Fig. 5K), we not only clarified phenotypic subtleties along the AS-based developmental trajectory but also illuminated the complexity of AS mechanisms underlying cell differentiation and embryonic development, successfully demonstrating the capabilities of the AEnet algorithm.

## Discussion

The discovery of cellular heterogeneity can be enhanced by multiple modalities derived from single-cell genomics technologies. For example, SHARE-seq provides insights into cell identity by jointly detecting gene expression and chromatin regulation [50], while CITE-seq simultaneously studies the transcriptome and protein expression, accounting for posttranscriptional and translational modifications to enable fine-grained detection of cell populations [51]. However, generating such data remains laboratory-intensive, involving complex, multistep workflows that require careful optimization and the use of specialized reagents and equipment. In fact, alternative splicing events represent another inherent modality that is often present in common scRNA-seq data [52, 53]. These events can be cost-efficiently detected using plate-based platforms, like Fluidigm C1 and Smart-seq [54, 55], or droplet-based platforms with higher sequencing depth [22, 56, 57].

The development of AEnet introduces a novel framework that integrates single-cell alternative splicing dynamics with gene expression profiles to construct regulatory networks reflective of cellular heterogeneity. By applying AEnet to 3 distinct single-cell datasets, we found that the interplay between AS and gene expression is dynamic in the process of generating cell populations. For tumor patient samples with varying immunotherapy responses and pan-cancer T-cell analysis, the integrated clustering results reveal distinct cell states within the RNA-based subtypes. In contrast, during cell differentiation in embryonic gastrulation, the clustering results more closely resemble the major cell types. This suggests that AS events contribute differently across tissues and conditions in distinguishing cell clusters, with a particularly important role during the fine-grained clustering process. Therefore, previous methods that first rely on RNA-based clustering and then apply AS-based differential analysis as a separate downstream step are not optimal for capturing cellular heterogeneity using AS events.

A central advantage of the core advantages of AEnet lies in its ability to “modularize” complex gene expression data by grouping AS patterns, thereby minimizing noise from RNA expression profiling, which is often referred to as batch effects in scRNA-seq analysis. This modular approach also mitigates AS-based biases related to missing values, tumor heterogeneity, and patient-specific factors, while enabling the identification of novel cell subpopulations and intermediate states that may remain obscured using traditional methods.

It is important to note that AEnet is primarily designed for unsupervised discovery of splicing heterogeneity and regulatory mechanisms within a single biological context (e.g., a specific sample or condition), rather than for direct differential splicing comparisons across clusters or conditions (e.g., disease vs. healthy states). Its strengths lie in integrating alternative splicing and gene expression to characterize intracondition splicing programs, identify regulatory modules, and predict affected pathways—capabilities enabled by its modular approach to minimize noise and capture context-enriched features. Notably, AEnet can also predict key regulatory components such as splicing factors (SFs) that drive observed splicing patterns, providing mechanistic insights into posttranscriptional regulation. For users seeking groupwise differential splicing analysis, AEnet’s outputs (e.g., anchor ASPs and regulatory modules) can be integrated with dedicated tools like MARVEL or MAJIQ-SC, which specialize in statistical comparisons across predefined groups. A detailed functionality comparison of these methods is provided in Supplementary Table S1, aiding users in selecting tools aligned with their research objectives.

Looking forward, advances in sequencing technologies, particularly long-read platforms, will further enhance the resolution and accuracy of AS detection at the single cell level [14, 58]. This advancement will significantly broaden the use of our method as a conventional analysis tool. However, even with well-designed sequencing, barcode errors in current single-cell long-read sequencing still significantly affect data quality, leading to the misassignment of AS events to unrelated cells [59]. We look forward to the rapid development of high-precision, high-throughput long-read sequencing technologies to advance research in the single-cell AS field and the development of our software in the near future.

## Methods

### Dataset preprocessing prior to AEnet for alternative splicing

Dataset preprocessing prior to AEnet involved a series of essential steps aimed at constructing the cell-junction count matrix and the annotation file for junctions, using the established DESJ detection pipeline [19]. Initially, alignment software such as STAR was employed to obtain the coverage information of junctions in each cell [60]. To ensure the reliability of the data, the junctions with a minimum of Rm reads in Cellm cells were retained, with default values set as Cellm = 10 and Rm = 4. Additionally, all junctions were annotated to determine their primary gene sources. Specifically, we selectively retained junctions that were exclusively associated with a single gene to ensure accuracy of the alignment. Furthermore, a count matrix was generated, reflecting the read numbers of junctions in each cell. Subsequently, the count matrix was normalized through dividing the counts by the unique mapped read number for each cell, resulting in the generation of the counts per million (CPM) matrix. The diligent execution of these preprocessing steps ensured the integrity and reliability of the dataset, allowing for robust analysis within the AEnet framework.

### Detailed procedure for AEnet

The AEnet method consists of 6 major steps: ASP identification and quantification, AEN network and multiple sample integration, anchor ASP identification, ASP cluster identification, cell clustering, and regulatory mechanism prediction. The following sections describe each step in detail.

#### ASP identification and quantification

Exon–exon junctions with the same starting point or endpoint were defined as ASPs. The PSI value of the ASPs in cells was measured by comparing the coverage of 1 junction to the 2 junctions derived from the ASP. Subsequently, we integrated all cells and all ASPs into a cell–ASP PSI matrix.

#### AEN network and multiple samples integration

We integrated the alternative splicing event scoring matrix and gene expression matrix of all cells in each sample to infer the potential relationship between ASPs and gene expression. For each sample, AEnet calculated the significance of the correlation between ASP PSI values and gene expression using Spearman’s rank correlation. Significant ASP PSI–gene expression links (*P* < 0.01) were then used to construct an alternative splicing–gene expression association network (AEN) for each sample. The edge width of the links in the network was determined using the Spearman correlation coefficient, which indicates both the strength and the direction (positive or negative) of the correlation. To ensure robustness, we extracted ASP–EXP links that exhibited consistent trends across multiple samples (defined as more than 2 samples). These links were then integrated into a multisample AEN network. In this pan-sample AEN network, the width of the edges corresponds to the number of samples supporting each ASP–EXP link, while the color indicates the direction of the correlation (positive or negative). This approach effectively mitigated batch effects and strengthened the reliability of the analysis.

#### Anchor ASP/gene identification

We calculated the degree strength of the ASP in the AEN network and selected the top ASPs based on their ranking. The degree of ASPs was determined by the number of links to the ASP in the AEN network. A higher degree indicates a stronger association between the ASP and gene expression dynamics in the dataset. The top 1,500 ASPs (by default) were selected for downstream analyses, provided that each ASP was associated with more than 15 positive and 15 negative links. To reduce data complexity and extract key information, we calculate the Jaccard metric of ASP–EXP links (where genes linked to the ASP are treated as sets) to represent their similarity for each pair of ASPs, thereby constructing a similarity matrix of anchor ASPs. Analogously, anchor genes were defined as those associated with ASPs from more than 30 distinct genes. For these anchor genes, a similar Jaccard similarity metric was calculated based on their associated ASPs, enabling the construction of a gene-level similarity matrix.

#### ASP/gene cluster identification

Using hierarchical clustering methods, we divided the ASPs into ASP clusters according to their similarity, ensuring that ASPs in the same clusters were associated with similar gene expression sets. The number of clusters is set to 25 by default. Clusters are filtered by ensuring that at least 10% of ASP pairs within each cluster exhibit a similarity score higher than 0.1. Clusters with fewer than 10 ASPs are merged with the most similar clusters, provided that the similarity between clusters is above 0.1. The similarity between 2 ASP clusters is defined as the proportion of ASP pairs with a similarity score greater than 0.1, where 1 ASP in the pair belongs to 1 cluster and the other ASP belongs to the other cluster. An analogous clustering procedure was applied to anchor genes to identify gene clusters based on shared ASP associations.

#### Cell clustering analysis

To further understand the cell heterogeneity at the level of alternative splicing, we performed cell clustering analysis based on alternative splicing and gene expression. Specifically, we first calculated the enrichment scores of each ASP cluster and each gene cluster for individual cells, resulting in a cell-by-cluster enrichment score matrix. The enrichment score of an ASP cluster in a given cell was defined as the average PSI value of all ASPs within that cluster. Similarly, the enrichment score of a gene cluster in a given cell was defined as the average expression level of all genes in that cluster. Next, the cell–ASP and gene cluster enrichment score matrix was normalized using the scale function. Finally, the normalized matrix was used as input for the FindNeighbors and FindClusters functions of Seurat to detect cell clusters [61, 62]. Notably, cell clustering can be performed based on either cell–ASP cluster enrichment score matrix or cell–gene cluster matrix alone. Finally, cell subpopulations were delineated using a dual-modality approach that integrates ASPs with gene expression data.

#### Regulatory mechanism inference

To identify key splicing factors, we integrated the AEN network with a predefined list of splicing factors and a specific set of ASP events [63]. This allowed us to construct a subnetwork of ASP splicing factors, facilitating the exploration of the regulatory relationships between alternative splicing events and splicing factors. The higher the degree strength of the splicing factor, the more it indicated a close relationship with the occurrence of these ASP events. The regulatory direction of splicing factors on the given set of ASPs was determined based on the proportion of positive or negative correlations. Specifically, if 75% or more of the splicing factor–ASP pairs were positive (or negative), the splicing factor was classified as positively (or negatively) regulating the ASP sets.

We also identified key pathways associated with different ASP patterns. For each ASP event, gene sets that were positively and negatively correlated with the event were identified based on the AEN network. Functional enrichment analysis was then performed on both the ASP’s gene and the positively (or negatively) correlated gene sets. The pathway to which the ASP gene belongs is considered the most likely pathway affected by the ASP change.

### Performance evaluation of AEnet

To evaluate AEnet’s performance in inferring ASPs relative to MARVEL—the currently most comprehensive method—we applied a demo dataset of MARVEL, comprising iPSCs and iPSC-derived endoderm cells [30]. ASP patterns were identified using the asp function of AEnet, while MARVEL was run with default parameters for direct comparison. To systematically evaluate the impact of count thresholds on ASP detection, we tested a range of minimum read count cutoffs: 0, 3, 5, 7, and 9. In parallel, to assess potential biases introduced by filtering against rare but biologically relevant splicing events, we stratified all ASPs into 5 categories based on the number of supporting cells: invalid, ≤20 cells (excluded); type 1, >20–30 cells; type 2, >30–40 cells; type 3, >40–50 cells; and type 4, >50 cells. Subsequent analyses evaluated the influence of each ASP type on link robustness and biological relevance.

To evaluate AEnet’s ability to identify ASP clusters, a simulated ASP similarity matrix was generated, with ASPs represented along both rows and columns, and similarity values (measured by the Jaccard index) as matrix elements. To assess robustness under varying noise conditions, different types of noise were introduced: uniform noise via the noiseInjector.unif function from the GROAN package, Gaussian noise using the rtruncnorm function, and Poisson noise using the poisson_discrete function from the truncnorm package. The number of ground-truth ASP clusters was set to 3. AEnet’s clustering performance was quantified by a consistency score, defined as the Jaccard index between the predicted ASP clusters and the simulated ground-truth clusters.

### Benchmarking AEnet against other methods

We next focused on evaluating the clustering performance of AEnet using published datasets with established cell-type annotations as ground truth, employing ARI and NMI as evaluation metrics. Three independent datasets—iPSC [18], HCC [28], and T-cell datasets [29]—were used for benchmarking, with detailed information provided in Supplementary Table S3. To assess the contribution of different data modalities, we conducted ablation analyses by providing AEnet with the expression matrix alone, the ASP PSI matrix alone, or both. These inputs were used to construct the full ASP–EXP network, as well as ASP-only and EXP-only networks, followed by cell clustering based on each configuration.

To compare the performance of AEnet with existing splicing-based clustering methods, we evaluated SCASL and scQuint across 3 benchmark datasets. The cell–junction count matrix was used as input for both SCASL and scQuint, with all methods run using their respective default parameters. Notably, scQuint was used solely for low-dimensional embedding, whereas SCASL provided both embeddings and clustering outputs. To further assess AEnet’s robustness to batch effects, we analyzed data from 3 patients in the lung cancer dataset and 4 patients from the CRC and RHCC cohorts. We then examined the distribution of cells in the low-dimensional embedding space to evaluate the extent of batch mixing.

To further demonstrate the versatility of AEnet, we applied it to a widely used 10x Genomics PBMC dataset [34]. Due to the limited sequencing depth of 10x data, AEnet was unable to detect a sufficient number of AS events for downstream analysis. Therefore, we focused instead on APA events, which also reflect isoform-level regulatory dynamics. APA usage was quantified using the scAPAtrap [64] and movAPA [65] package, and the resulting cell-level relative usage of distal (RUD) polyadenylation site values, together with gene expression data, were used as input to AEnet with default parameters to evaluate its performance on 10x datasets.

### The lung cancer cells data processing

We downloaded the scRNA-seq raw reads of the human lung cancer dataset from the NCBI database under accession code PRJNA591860 [33]. This dataset contained 1,286 normal and cancer cells from 6 patients before initiating systemic targeted therapy (TKI naive [TN]), at the residual disease (RD) state, which includes samples taken at any response groups during treatment with targeted therapy while the tumor was regressing or stable by clinical imaging (RD), and upon subsequent progressive disease, as determined by clinical imaging, at which point the tumors showed acquired drug resistance (progression [PD]). The human genome (version GRCh38) was used as the reference genome for alignment with STAR (v2.5.3) [60]. We used an existing pipeline to create the junction count matrix. We first merged all the output of the SJ.out.tab files from the STAR aligner. Next, we conducted the dataset preprocessing prior to the AEnet step, described as before. Finally, we got the cell-junction CPM matrix and the junction annotation files for the dataset.

Then, we used the outcome from the above step as the input to the AEnet pipeline. First, the CPM matrix was input to the asp function of AEnet with default parameters, and the ASPs for each gene were identified. Second, the correlation network between ASPs and gene was constructed using the asp function with default parameters based on the expression matrix and the junction CPM matrix. Next, we further refined the AEN network by retaining only the connection pairs with at least 4 samples supported, using the merge_cor function with default parameters. Additionally, the ASPs with positive or negative links of more than 20 were retained, and 70 key ASPs were identified. These key ASPs were then clustered with hierarchy clustering, leading to the identification of 6 ASP clusters based on their similarity, using the junction_clustering function with default parameters. Subsequently, we calculated the enrichment score of each ASP cluster for the cells and performed cluster analysis using the enrichment score matrix of the cell–ASP clusters, categorizing the cells into 3 groups using asp_score and cell_clus function with a resolution of 0.2. To further identify the key splicing factors involved in the formation of the C_4 ASP clusters, we used the key_sf function to detect the key SF. Additionally, we also grouped cells into different populations based on gene expression. The gene count matrix was normalized using log1p normalization. Next, the top 3,000 highly variable genes were selected to perform principal component analysis (PCA). Subsequently, 20 dimensions of principal components were used to perform Louvain clustering and UMAP-based visualization.

The SCASL package was applied to the junction count matrix of tumor cells to perform cell clustering, using default parameters for filtering, normalization, imputation, and clustering [31]. The Seurat [61, 62] package was applied to the expression matrix of tumor cells to perform normalization, dimensionality reduction, and clustering: (i) the data were normalized using the “LogNormalize” function; (2) the top 2,000 highly variable genes were detected with the “FindVariableFeatures” function and selected, with the batch effects across different samples corrected by the “FindIntegrationAnchors” and “IntegrateData” functions; (iii) a k-nearest neighbors–based graph in the 20 PCA space was constructed and refined by cell–cell weights using the “FindNeighbors” function; (iv) representative results from graph-based clustering were obtained using the “FindClusters” function with a resolution of 0.3; and (v) the top 20 PCAs were used to perform UMAP for visualization of the cells.

### The pan-cancer T-cell data processing

We downloaded the scRNA-seq raw reads of human T cells in the fasta format from the EGA database (EGAS00001002072 for HCC, EGAS00001002791 for CRC), NCBI database (PRJNA591860 for LUAD), and CNSA database (CNP0000650 for RHCC). The corresponding gene expression matrix was downloaded from the GEO database (GSE98638 for HCC, GSE108989 for CRC) and CNSA database (CNP0000650 for RHCC). The human genome (version GRCH38) was used as the reference genome for alignment with STAR (v2.5.3). We used an existing pipeline to create the junction count matrix. We first merged all the output of the SJ.out.tab files from the STAR aligner. Next, we conducted the dataset preprocessing prior to the AEnet step, described as before. Finally, we got the cell-junction CPM matrix and the junction annotation files for each dataset.

Then, we used the outcome from the above step as the input to the AEnet pipeline. First, the CPM matrix was input to the asp function of AEnet with default parameters, and the ASPs for each gene were identified for each dataset. Second, the correlation network between ASPs and gene was constructed using the asp function with default parameters based on the expression matrix and the junction CPM matrix for each dataset. Next, we further refined the AEN network by retaining only the connection pairs with at least 2 samples supported for each dataset, using the merge_cor function with default parameters. Additionally, the ASP–EXP links supported by at least 2 datasets were retained for the downstream analysis. As a result, 1,223 key ASPs were identified based on their ranking and absolute number of degrees (top 2,000 with a degree number greater than 10). These key ASPs were then clustered, leading to the identification of 14 ASP clusters based on the similarity of their associated phenotypes through the junction_clustering function with default parameters. Subsequently, we calculated the enrichment score of each ASP cluster for the cells of the 4 datasets and performed cluster analysis using the enrichment score matrix of the cell–ASP clusters, categorizing the cells into 10 groups using the cell_clus function with a resolution of 0.9. To further identify the key splicing factors involved in the formation of the 14 ASP clusters, we used the key_sf function to detect the key SF for each ASP cluster. AEnet was also used to detect key gene sets affected by ASPs from the same gene. These gene sets were then used to identify enriched pathways associated with different alternative splicing patterns of the same gene, such as CD3D and FYB1. The SCASL package was applied to the junction count matrix of T cells in multiple datasets to perform cell clustering, using default parameters for filtering, normalization, imputation, and clustering [31].

### Gastrulating human embryo cell data processing

We downloaded the scRNA-seq raw reads of the human embryo dataset from ArrayExpress under accession code E-MTAB-9388. This dataset contained 1,195 cells of the human embryo on embryonic day 16 assigned into 10 clusters. The human genome (version GRCH38) was used as the reference genome for alignment with STAR (v2.5.3). We used an existing pipeline to create the junction count matrix. We first merged all the output of the SJ.out.tab files from the STAR aligner. Next, we conducted the dataset preprocessing prior to the AEnet step, described as before. Finally, we got the cell-junction CPM matrix and the junction annotation files for the dataset.

Then, we used the result from the above step as the input to the AEnet pipeline. First, the CPM matrix was input to the asp function of AEnet with default parameters, and the ASPs for each gene were identified. Second, the correlation network between ASPs and gene was constructed using the asp function with default parameters based on the expression matrix and the junction CPM matrix. Next, we further refined the AEN network by retaining only the connection pairs with *P* values less than 1e-4. As a result, 1,604 key ASPs were identified based on their ranking and absolute number of degrees (top 2,000 with a degree number greater than 50). These key ASPs were then clustered, leading to the identification of 25 ASP clusters based on the similarity of their associated phenotypes. Subsequently, we calculated the enrichment scores of each ASP class within the cells and performed cluster analysis using the enrichment score matrix of the cell–ASP clusters, categorizing the cells into 11 groups using the cell_clus function with a resolution of 0.1. To further identify the key splicing factors involved in the formation of the 25 ASP clusters, we used the key_sf function to detect the key SF for each ASP class. AEnet was also used to detect key gene sets affected by ASPs from the same gene. These gene sets were then used to identify enriched pathways associated with different alternative splicing patterns of the same gene, such as TNRC6B.

### Pathway enrichment analysis

Metascape (RRID:SCR_016620) was used to characterize the biological functions of the differentially expressed genes of cells in different statuses [66]. The differentially expressed genes between different cell types were uploaded into the Metascape for the pathway analysis with default setting.

### Survival analysis

GEPIA2 (RRID:SCR_026154) was used to analyze the survival status of HNRNPLL [67]. HNRNPLL was uploaded into the GEPIA2, and the reference datasets were the whole datasets from The Cancer Genome Atlas.

## Availability of Source Code and Requirements

Project name: AEnet

Project homepage: https://github.com/liushang17/AEN

Operating system(s): Platform independent

Programming language: R

License: GPL-3.0 license

RRID:SCR_027285

A version of record snapshot of the GitHub repository has been archived in the Software Heritage [68].

## Supplementary Material

giaf110_Supplemental_Files

giaf110_Authors_Response_To_Reviewer_Comments_original_submission

giaf110_Authors_Response_To_Reviewer_Comments_Revision_1

giaf110_GIGA-D-25-00064_Original_Submission

giaf110_GIGA-D-25-00064_Revision_1

giaf110_GIGA-D-25-00064_Revision_2

giaf110_Reviewer_1_Report_Original_Submission Xin Shao -- 3/12/2025

giaf110_Reviewer_1_Report_Revision_1 Xin Shao -- 7/15/2025

giaf110_Reviewer_2_Report_Original_Submission Sean Wen -- 3/14/2025

giaf110_Reviewer_2_Report_Revision_1 Sean Wen -- 7/15/2025

giaf110_Reviewer_3_Report_Original_Submission Cyrillus Tan -- 3/18/2025

giaf110_Reviewer_3_Report_Revision_1 Cyrillus Tan -- 7/14/2025

## Data Availability

The gastrulating human embryo dataset is available in the ArrayExpress database under accession number E-MTAB-9388. scRNA-seq raw reads of human T cells in fastq format are from the EGA database (EGAS00001002072 for HCC, EGAS00001002791 for CRC), NCBI database (PRJNA591860 for LUAD), and CNSA database (CNP0000650 for RHCC). The corresponding gene expression matrix was downloaded from the GEO database (GSE98638 for HCC, GSE108989 for CRC) and CNSA database (CNP0000650 for RHCC). The additional data files are hosted in Zenodo [69].

## Additional Files

**Supplementary Fig. S1**. The key difficulties in cell splicing heterogeneity analysis. (A) The NaN value problem and its source. (B) Schematic diagram illustrating the application of AEnet to multiple samples. (C) Systematic criteria for detecting batch-vulnerable ASP–EXP links across heterogeneous samples and experimental conditions. (D) Schematic diagram illustrating the effect of splicing factors on the selection of splicing patterns. (E) Schematic diagram showing how different ASPs of the same gene impact distinct biological pathways. (F) The selection of anchor ASPs according to the number of ASP–gene links of the ASPs. (G) The identification of ASP clusters. (H) The selection of anchor genes according to the number of ASP–gene links of the genes.

**Supplementary Fig. S2**. The three major functions of AEnet. (A) Schematic overview of the AEnet pipeline. Inputs (normal): cell–junction count matrix, cell–gene expression matrix, and a predefined list of splicing factors. Key steps are highlighted in bold. Outputs (in bold italic): cell clusters, predicted key splicing factors, and signaling pathways regulated by specific alternative splicing patterns (ASPs). (B) Schematic diagram of the analysis of cellular splicing heterogeneity. (C) Identification of the most critical splicing factors associated with the ASP set. (D) Schematic diagram of pathway inference associated with different patterns of ASP.

**Supplementary Fig. S3**. The evaluation of AEnet. (A) Schematic overview of the pipeline for the comprehensive evaluation of AEnet, organized into 4 steps: (i) ASP determination and quantification (compared with MARVEL), (ii) AEN network construction and multisample integration (evaluation of different count thresholds), (iii) ASP/gene cluster identification (assessment under varying noise levels), and (iv) cell clustering (ablation analysis and comparison with SCASL and scQuint). (B) The common types of alternative splicing. (C) The comparison between AEnet and MARVEL in the detection of ASPs. (D) The number of alternative splicing events detected by MARVEL. (E) The number of alternative splicing events detected by AEnet as well as the comparison with MARVEL. (F) The demo example of FHOD3 for the rare patterns detected by AEnet. (G) Schematic illustration of PSI calculation in DESJ detection. (H) Impact of low gene expression on the accuracy of DESJ detection: a case study using genes without alternative splicing events. (I) Schematic diagram of the calculation of PSI for the alternative splicing pattern (ASP).

**Supplementary Fig. S4**. Evaluation of different count thresholds on AEnet performance. (A) Schematic overview of the benchmarking strategy for evaluating different count thresholds on the construction of ASP–EXP link and anchor ASPs. (B, C) Quantification of ASP–EXP links across varying count thresholds, with color coding denoting link specificity in the HCC dataset (B) and the T-cell dataset (C). (D, E) Patient-level validation rates for specific ASP−EXP links at different expression count cutoffs in the HCC dataset (D) and the T-cell dataset (E). (F, G) Quantification of anchor ASPs across varying count thresholds, with color coding denoting link specificity in the HCC dataset (F) and the T-cell dataset (G). (H, I) The similarity of anchor ASPs across different expression count cutoffs in the HCC dataset (H) and the T-cell dataset (I). (J) Schematic diagram of the identification of ASP types for 1 sample or 1 dataset. (K) Quantitative analysis of ASP subtypes categorized by the presence (blue) or absence (gray) of ASP–EXP links in the HCC dataset. (L) Frequency distribution of anchor ASPs stratified by ASP subtype classification in the HCC dataset. (M) Quantitative analysis of ASP subtypes categorized by the presence (blue) or absence (gray) of ASP–EXP links in the T-cell dataset. (N) Frequency distribution of anchor ASPs stratified by ASP subtype classification in the T-cell dataset.

**Supplementary Fig. S5**. The evaluation of ASP cluster prediction of AEnet with random noise. (A, B) Simulated ASP–ASP similarity matrix (B) was created with increasing noise (A). (C) Boxplots present the Jaccard index of ASP clusters between the background and AEnet. (D) ASP clusters identified by AEnet in the simulated datasets.

**Supplementary Fig. S6**. The evaluation of ASP cluster prediction of AEnet with Gaussian noise and Poisson noise. (A, B) Simulated ASP–ASP similarity matrix (B) was created with increasing Gaussian noise (A). (C) Boxplots present the Jaccard index of ASP clusters between the background and AEnet with the increasing Gaussian noise. (D, E) Simulated ASP–ASP similarity matrix (E) was created with increasing Poisson noise (D). (F) Boxplots present the Jaccard index of ASP clusters between the background and AEnet with the Poisson noise.

**Supplementary Fig. S7**. Ablation analysis for AEnet. (A–C) Uniform Manifold Approximation and Projection (UMAP) visualizations showing the published cell-type annotations (right) and the predicted clusters (left) derived from the ASP–EXP (AEnet) network (top), expression-only (EXP) network (middle), and ASP-only network (bottom) for (A) full-length iPSC and (B) HCC single-cell RNA-seq datasets. (C) UMAP visualizations showing the published cell-type annotations (bottom) and the predicted clusters (upper) derived from the ASP–EXP (AEnet) network (left), expression-only (EXP) network (middle), and ASP-only network (right) for the T-cell datasets. Clustering solutions shown represent those with the highest Adjusted Rand Index (ARI).

**Supplementary Fig. S8**. Benchmarking AEnet with SCAL and scQuint. (A–C) UMAP visualizations showing the published cell-type annotations (right) and the predicted clusters (left) derived from AEnet (upper) and SCASL (bottom) for (A) full-length iPSC, (B) HCC single-cell RNA-seq, and (C) T-cell datasets. Clustering solutions shown represent those with the highest Adjusted Rand Index (ARI). (D) UMAP visualizations demonstrate the relative performance of scQuint (right) in the full-length iPSC dataset, T-cell sequencing data, and HCC single-cell RNA-seq data.

**Supplementary Fig. S9**. Performance comparison of AEnet and SCASL. (A, B) UMAP shows the clustering of cell types determined by AEnet (A) and SCASL (B) (left) and patient clustering (right) for epithelial cells from multiple patients. (C, D) UMAP shows the clustering of cell types determined by AEnet (C) and SCASL (D) (left) and patient clustering (right) for T cells from the colorectal cancer (CRC) and recurrent hepatocellular carcinoma (RHCC) datasets. (E) Heatmap showing APA classes derived from dimensionality reduction using AEnet. (F) UMAP visualization of cell clustering based on alternative polyadenylation profiles. (G) Quantitative benchmarking of clustering performance using ARI and NMI, evaluated against the ground-truth annotations of the PBMC dataset.

**Supplementary Fig. S10**. AEnet reveals cellular splicing heterogeneity and its key splicing events in tumor-infiltrating T cells across various cancer types. (A) The barplot displays the selection of high-quality links in the AEnet of pan-cancer T cells. (B) The selection of key alternative splicing patterns. (C) The heatmap shows the ASP cluster from dimension reduction of AEnet. (D) UMAP displays cell sources. (E) The heatmap displays the expression of marker genes across cell clusters determined by AS. (F) Heatmap of ASP clusters ordered by similarity, illustrating intercluster relationships within the pan-cancer T-cell dataset. (G–J) Distribution of enrichment scores for selected ASP clusters. (K) The expression of key splicing factors across clusters determined by alternative splicing. (L) The pathways enriched in genes with ASP associated with HNRNPLL. (M) The PSI distribution of the CD3D-201 isoform within CD3D within exhaustion and other T cells. Statistical analysis was performed using Student’s *t* test. (N) The pathways enriched in the gene sets with different isoforms of CD3D.

**Supplementary Fig. S11**. AEnet reveals intermediate cell status during gastrulation. (A) The selection of key alternative splicing patterns. (B) The heatmap shows the ASP clusters from dimension reduction of AEnet. (C) The heatmap displays the expression of developmental markers across cell clusters determined by AS. (D) The heatmap displays the enrichment score of ASP clusters across cell clusters determined by AS. (E) The expression of SNRPN and PSIP1 across the cell clusters determined by AS. (F) The length of junction that is differentially used in different cell types from Song et al. Statistical analysis was performed using Student’s *t* test. (G) The expression of SNRPD2 across the cell clusters determined by AS. (H) The pathways enriched in the genes within C_1 and C_18, which shows correlation with the expression of SNRPD2. (I, J) The heatmap displays the relationship between ASP clusters and key splicing factors (I), as well as the expression of key splicing factors (J). **P* < 0.05, ***P* < 0.01, ****P* < 0.001.

**Supplementary Table S1**. Summary of the function for methods.

**Supplementary Table S2**. Splicing factor list.

**Supplementary Table S3**. The datasets used to evaluate the performance of AEnet.

**Supplementary Table S4**. Summary of the 5 methods.

## Abbreviations

A3SS: alternative 3′ splice site; A5SS: alternative 5′ splice site; AEN: alternative splicing–gene expression association network; AEnet: Alternative Splicing-Gene Expression Network; AFE: alternative first exon; ALE: alternative last exon; APA: alternative polyadenylation; ARI: Adjusted Rand Index; AS: alternative splicing; ASP: alternative splicing pattern; CPM: counts per million; CRC: colorectal cancer; EMT: epithelial-mesenchymal transition; GO: Gene Ontology; HCC: hepatocellular carcinoma; iPSC: induced pluripotent stem cell; IR: intron retention; LUAD: lung cancer; MSE: multiple-exon splicing; MXE: mutually exclusive exon; N: normal; NaN: not a number; NK: natural killer; NMI: normalized mutual information; NMI: normalized mutual information; PBMC: peripheral blood mononuclear cell; PD: progressive disease; PitNET: pituitary neuroendocrine tumor; PSI: percent spliced-in; RD: residual disease; RHCC: recurrent hepatocellular carcinoma; RUD: relative usage of distal; scRNA-seq: single-cell RNA sequencing; SE: exon skipping; SF: splicing factor; VAE: variational autoencoder.
