# Supplementary material for: AEnet: a practical tool to construct the splicing-associated phenotype atlas at a single cell level
Source: Gigascience. 2025 Sep 24;14:giaf110. doi: 10.1093/gigascience/giaf110 (PMC12457822; doi:10.1093/gigascience/giaf110)
Supplement: giaf110_Supplemental_Files [file giaf110_supplemental_files.zip › FIG.S1-S6.pdf]

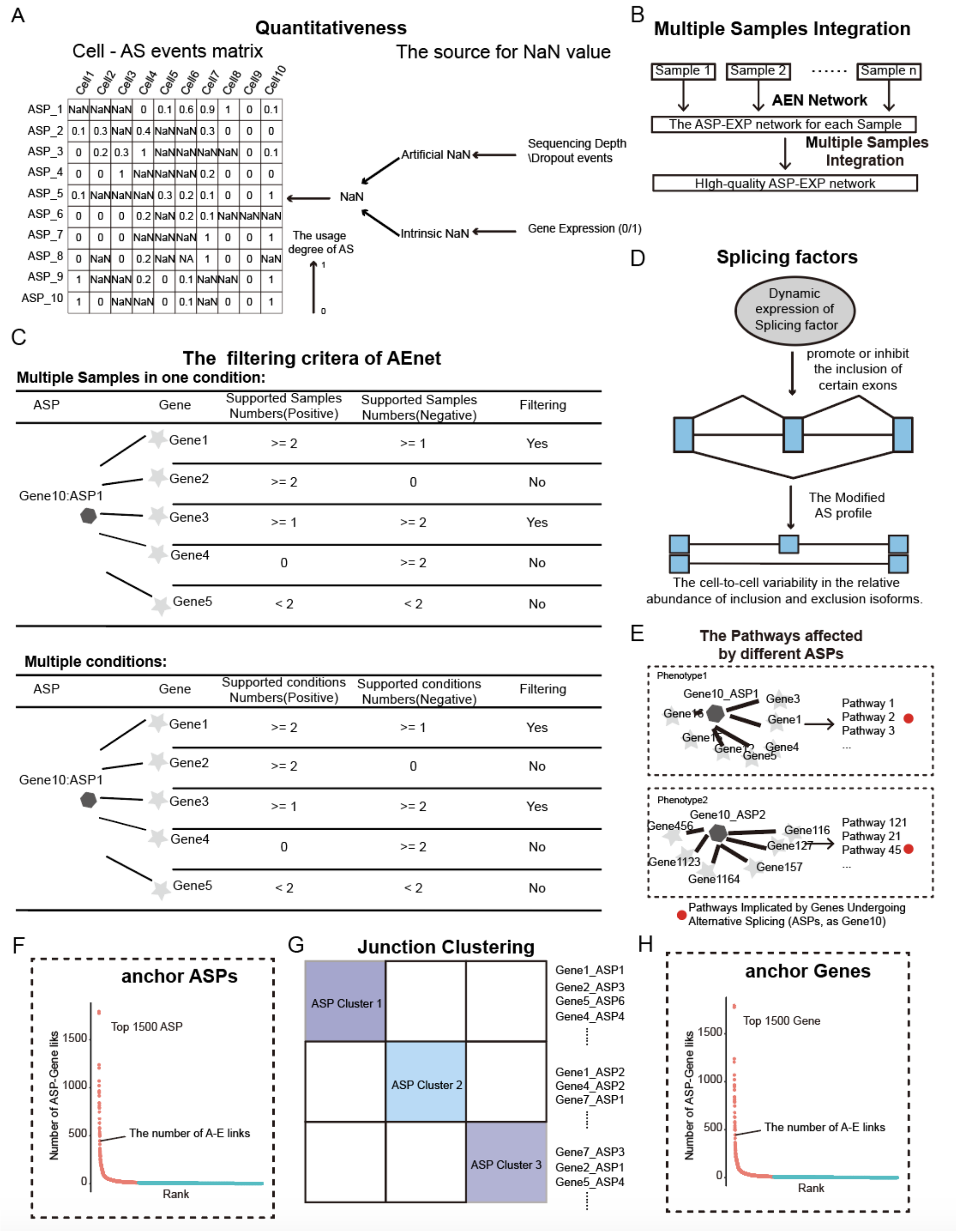

Figure S1. The key difficulties in cell splicing heterogeneity analysis. A. The NaN value problem and its source. **B.** Schematic diagram illustrating the application of AEnet to multiple samples. **C.** Systematic criteria for detecting batch-vulnerable ASP-EXP links across heterogeneous samples and experimental conditions. **D.** Schematic diagram illustrating the effect of splicing factors on the selection of splicing patterns. **E.** Schematic diagram showing how different ASPs of the same gene impact distinct biological pathways. **F.** The selection of anchor ASPs according to the number of ASP-

Gene links of the ASPs. **G.** The identification of ASP clusters. **H.** The selection of anchor Genes according to the number of ASP-Gene links of the genes.

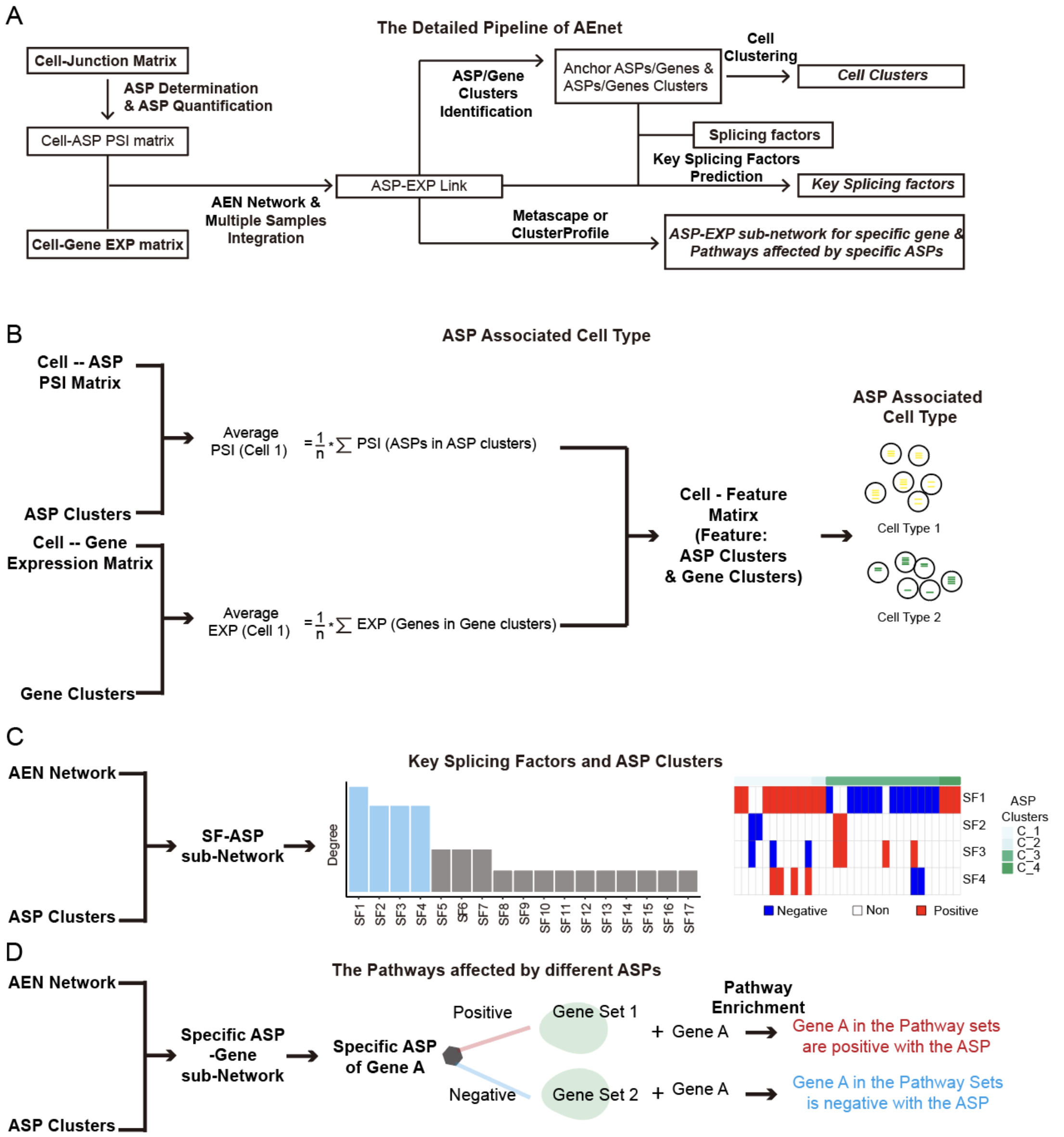

Figure S2. The three major functions of AEnet. A. Schematic overview of the AEnet pipeline. Inputs (Normal): Cell–Junction Count Matrix, Cell–Gene Expression Matrix, and a predefined list of splicing factors. Key steps are highlighted in bold. Outputs (in bold Italic): Cell clusters, predicted key splicing factors, and signaling pathways regulated by specific alternative splicing patterns (ASPs).**B.** Schematic diagram of the analysis of cellular splicing heterogeneity. **C.** Identification of the most critical splicing factors associated with the ASP Set. **D.** Schematic diagram of pathway inference associated with different patterns of ASP.

Evaluation of AEnet for Comprehensive Characterization of Splicing Heterogeneity

We next evaluated AEnet across multiple analytical steps, including ASP–EXP network construction (comprising ASP identification, ASP quantification, and the prediction of ASP and gene clusters), and cell clustering **(Fig. S3A)**. A total of four datasets were used for this evaluation, each accompanied by published annotations serving as ground truth. These datasets, including iPSC[18], HCC[28], and T cell datasets[29], are described in detail in **Table S3**.

We first evaluated the performance of AEnet in the ASP identification **(Fig. S3A-C)**. Current methods, such as BRIE, Outrigger, and MARVEL, are all fundamentally annotation-dependent and therefore unable to detect unannotated AS events. Additionally, these tools are limited in their ability to detect MSE **(Fig. S3B, Table S4)**. Since MARVEL has been compared with other methods and demonstrated to be the optimal one in its category in a previous study, we next compared the AS events detected by AEnet and MARVEL using the demo dataset of MARVEL, which includes induced pluripotent stem cells (iPSCs) and iPSC-derived endoderm cells **(Fig. S3C)**[30]. MARVEL identified a total of 20,509 SE, 1,279 MXE, 8,295 RI, 5,163 A5SS, 5,832 A3SS, 5,818 AFE, 2,072 ALE, and 0 MSE **(Fig. S3D)**. In MARVEL's iPSC dataset, AEnet detected 722,278 additional AS patterns (63% unannotated) across 12,866 genes, with MSE detection 3.2-fold higher than MARVEL **(Fig. S3E-F).** These findings highlight AEnet's comprehensive capability to detect a wide range of AS events—including unannotated and complex patterns—except for intron retention events. Compared to our homologous method DESJ-detection, AEnet overcomes critical limitations in low-depth robustness. DESJ-detection's single-junction PSI calculation leads to 38% error rates in low-coverage scenarios **(Fig. S3G-H)**. In contrast, AEnet mitigates this by focusing on junction reads with shared splice sites (requiring ≥5 supporting reads) and transforming read distributions into transcript usage ratios. This strategy reduced PSI error by 47% ($p<0.001$, **Fig. S3I**). Taken together, AEnet outperforms existing methods in detecting unannotated alternative splicing events, resolving complex AS patterns, and ensuring reliable quantification across varying data depths.

Low-count ASPs may introduce random fluctuations in PSI estimates, potentially leading to inaccurate quantification, which in turn can affect the construction of ASP-Exp links and the identification of anchor ASPs **(Fig. S4A)**. To evaluate the impact of count thresholds on these outcomes, we systematically tested a range of minimum read count thresholds: 0, 3, 5, 7, and 9 **(Fig. S4A)**. Using a threshold of 0 yielded the highest number of ASP-Exp links, with ~40% classified as "specific." However, 99% of these specific links were supported by fewer than two samples, suggesting they were likely artifacts of random fluctuations rather than meaningful biological associations

(Fig. S4B-C). Similarly, the 0-read threshold also led to an inflated number of anchor ASPs, most of which were linked to low-confidence, sample-specific signals **(Fig. S4D)**. In contrast, applying thresholds of ≥3 substantially reduced these spurious associations. Importantly, most biologically meaningful ASP-Exp links were retained even when moderate thresholds (>0) were applied **(Fig. S4E).** Based on these results, AEnet uses a default threshold of five supporting reads to ensure PSI robustness while minimizing noise.

While filtering low-count ASPs improves analytical reliability, we also considered the potential risk of excluding rare but biologically relevant splicing events. To address this, AEnet defines an ASP as "valid" in a sample only if it is supported by ≥5 reads across ≥20 cells. ASPs failing this criterion are excluded from downstream analysis, as their sparsity compromises the reliability of similarity estimates between splicing and expression. To assess whether this filtering excludes informative low-abundance ASPs, we stratified all ASPs into five categories based on their support across cells: Invalid: ≤20 cells (excluded); Type 1: >20–30 cells; Type 2: >30–40 cells; Type 3: >40–50 cells; Type 4: >50 cells. An ASP was assigned to the highest applicable category if it met the criteria in ≥3 samples **(Fig. S4F)**. As expected, higher-support ASPs (fewer NaNs) showed stronger ASP-Exp associations. Nonetheless, ~30% of Type 1 ASPs (i.e., relatively rare but retained) still showed significant correlations with gene expression, and four were identified as anchor ASPs **(Fig. S4G-H)**, indicating their functional relevance. In summary, our results support the use of both read count and sample support thresholds to reduce noise while preserving biological signal. AEnet remains capable of capturing meaningful but infrequent ASPs and provides user-defined thresholding to support flexible analysis tailored to specific research goals.

Finally, to assess the performance of AEnet in the identification of ASP clusters, an ASPs-ASPs similarity (Jaccard index) matrix was generated with increasing levels of noise to evaluate AEnet's effectiveness in ASP clusters prediction, **(Fig. S5A-B)**. Using a supervised hierarchical clustering method, AEnet demonstrated a high accuracy consistency score of approximately 0.9 between the background and the clusters identified, even when noise levels reached 80% **(Fig. S5C-D)**. These results demonstrate AEnet's robustness in identifying ASP clusters despite noise. Furthermore, we evaluated AEnet's performance using more realistic noise models, specifically Gaussian and Poisson noise. We simulated increasing levels of both Gaussian and Poisson noise and generated ASP–ASP similarity matrices (Jaccard index) under each noise condition to assess AEnet's robustness in ASP clustering **(Fig.**

S6A-B, D-E). Using a supervised hierarchical clustering approach, AEnet consistently achieved a high accuracy score—approximately 0.9—between the ground truth and the predicted clusters, even under noise levels as high as 90% for both noise models **(Fig. S6C, F)**. These results demonstrate AEnet's resilience to biologically relevant noise, further supporting its reliability in identifying splicing patterns in realistic, noisy settings.

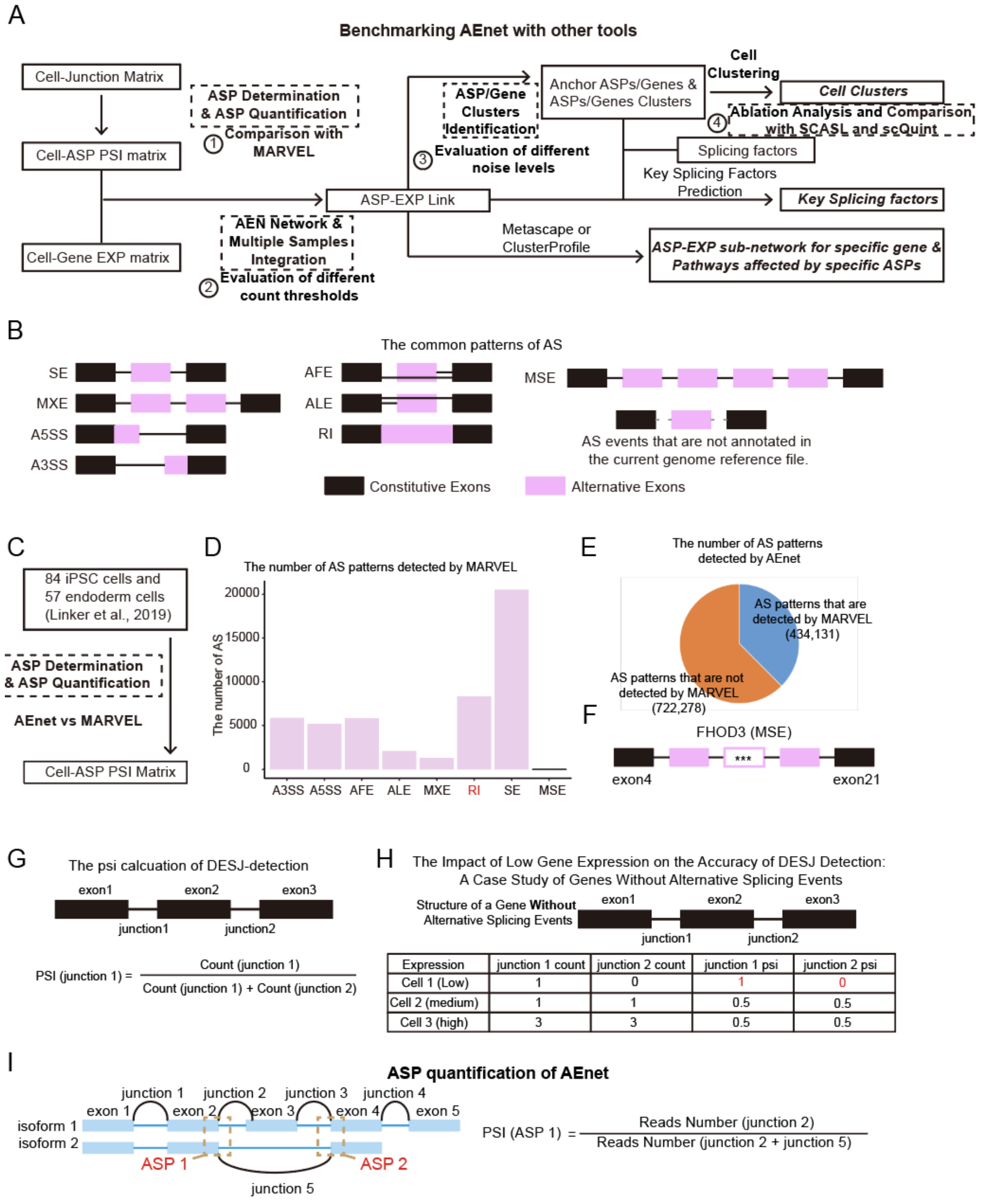

Expression	junction 1 count	junction 2 count	junction 1 psi	junction 2 psi
Cell 1 (Low)	1	0	1	0
Cell 2 (medium)	1	1	0.5	0.5
Cell 3 (high)	3	3	0.5	0.5

Figure S3. The evaluation of AEnet. A. Schematic overview of the pipeline for the comprehensive evaluation of AEnet, organized into four steps: (1) ASP determination

and quantification (compared with MARVEL), (2) AEN network construction and multi-sample integration (evaluation of different count thresholds), (3) ASP/gene cluster identification (assessment under varying noise levels), and (4) cell clustering (ablation analysis and comparison with SCASL and scQuint). **B.** The common types of Alternative splicing. **C.** The comparison between AEnet and MARVEL in the detection of ASPs. **D.** The number of alternative splicing events detected by MARVEL. **E.** The number of alternative splicing events detected by AEnet as well as the comparison with MARVEL. **F.** The demo example of FHOD3 for the rare patterns detected by AEnet. **G.** Schematic illustration of PSI calculation in DESJ-detection. **H.** Impact of Low Gene Expression on the Accuracy of DESJ Detection: A Case Study Using Genes Without Alternative Splicing Events. **I.** Schematic Diagram of the calculation of PSI for Alternative Splicing Pattern (ASP).

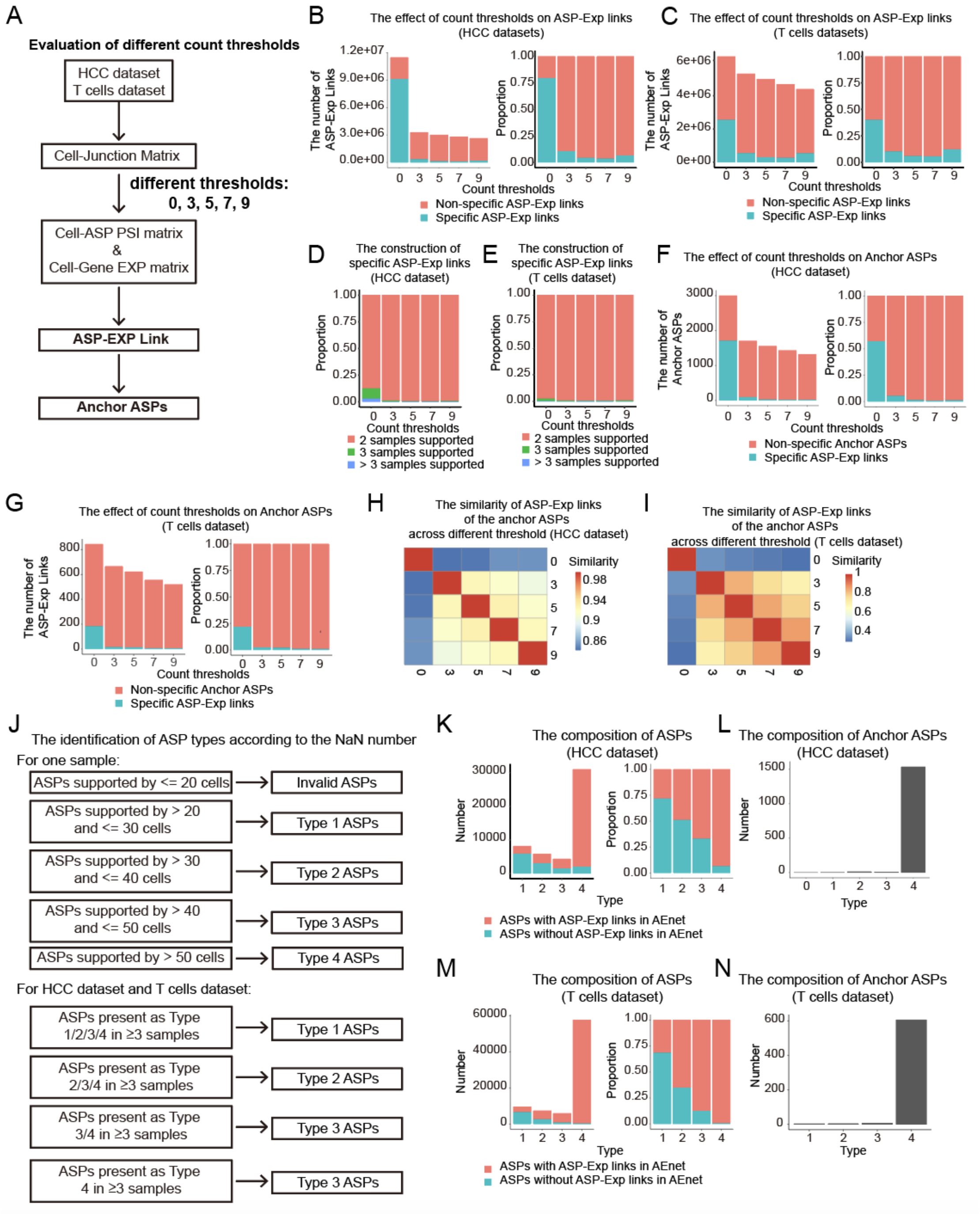

Figure S4. Evaluation of different count thresholds on AEnet performance. A. Schematic overview of benchmarking strategy for evaluating different count thresholds on the construction of ASP-EXP Link and Anchor ASPs. **B-C.** Quantification of ASP-EXP links across varying count thresholds, with color coding denoting link specificity in the HCC dataset (B) and the T cell dataset (C).**D-E.** Patient-level validation rates for specific ASP-EXP links at different expression count cutoffs in the HCC dataset (D) and the T cell dataset (E). **F-G.** Quantification of anchor ASPs across varying count thresholds, with color coding denoting link specificity in the HCC dataset (F) and the T cell dataset (G). **H-I.** The similarity of anchor ASPs across different expression count

cutoffs in the HCC dataset (H) and the T cell dataset (I). **J.** Schematic Diagram of the identification of ASP types for one sample or one dataset. **K.** Quantitative analysis of ASP subtypes categorized by presence (blue) or absence (gray) of ASP-EXP links in the HCC dataset. **L.** Frequency distribution of anchor ASPs stratified by ASP subtype classification in the HCC dataset. **M.** Quantitative analysis of ASP subtypes categorized by presence (blue) or absence (gray) of ASP-EXP links in the T cells dataset. **N.** Frequency distribution of anchor ASPs stratified by ASP subtype classification in the T cells dataset.

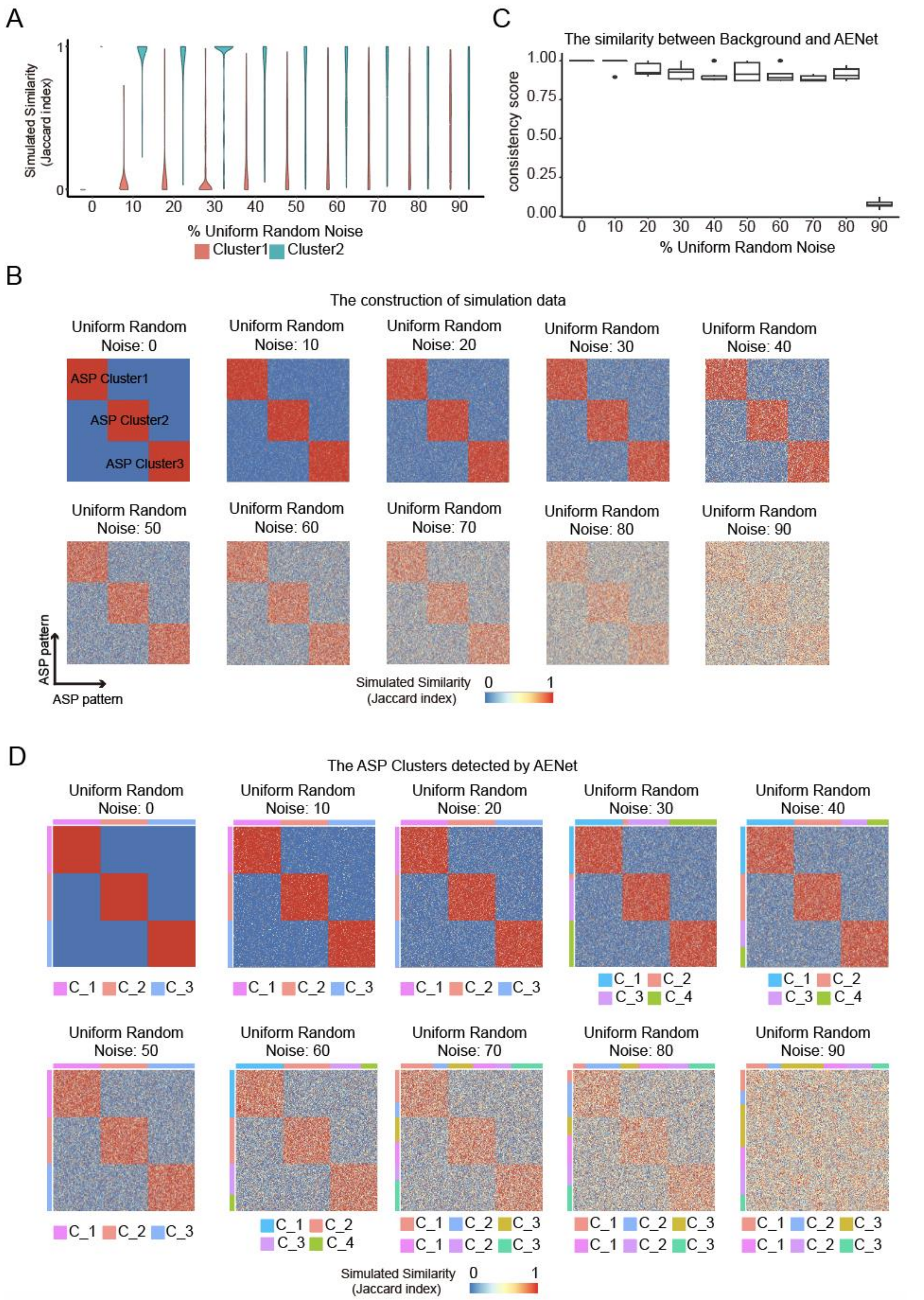

Figure S5. The evaluation of ASP clusters prediction of AEnet with random noise. **A-B.** Simulated ASPs-ASPs similarity matrix (B) was created with increasing noise (A). **C.** Box plots present the Jaccard index of ASP clusters between the background and AENet. **D.** ASP clusters identified by AENet in the simulated datasets.

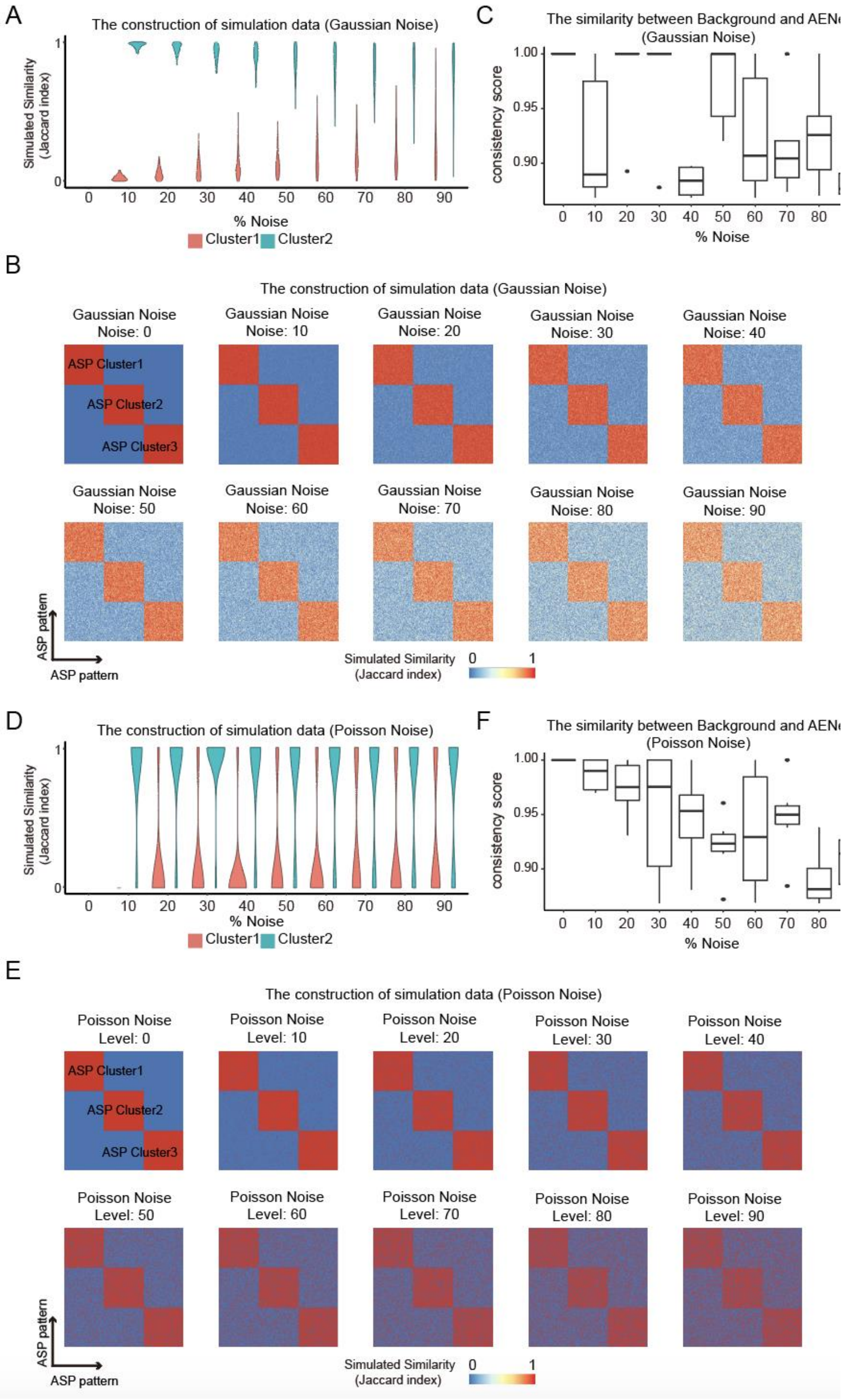

Figure S6. The evaluation of ASP clusters prediction of AEnet with gaussian noise and Poisson noise. A-B. Simulated ASPs-ASPs similarity matrix (B) was created with increasing gaussian noise (A). **C.** Box plots present theJaccard index of

ASP clusters between the background and AENet with the increasing gaussian noise. **D-E.** Simulated ASPs-ASPs similarity matrix (E) was created with increasing poisson noise (D). **F.** Box plots present the Jaccard index of ASP clusters between the background and AENet with the Poisson noise.

Benchmarking AEnet Against Existing Methods

While the above analyses validate AEnet's basic performance in characterizing splicing heterogeneity, its practical value in the field requires comparison with existing state-of-the-art methods. We thus benchmarked AEnet against established tools for single-cell AS analysis. We next evaluated AEnet's performance in capturing cellular heterogeneity using the three benchmarking datasets. Comprehensive ablation analyses, using published cell type annotations as ground truth, demonstrated that the joint ASP-EXP model (AEnet) consistently outperformed the standalone AS-only (Anet) and expression-only (Enet) approaches **(Fig. 2A)**. Specifically, the median ARI scores were 0.81 (AEnet), 0.68 (Anet), and 0.77 (Enet) for the iPSC dataset; 0.58 (AEnet), 0.10 (Anet), and 0.39 (Enet) for the HCC dataset; and 0.42 (AEnet), 0.10 (Anet), and 0.32 (Enet) for the T cell dataset **(Fig. 2B–D)**. These results highlight that integrating ASP features with gene expression significantly enhances clustering resolution and biological interpretability. Moreover, AEnet produced the most informative low-dimensional embeddings across all datasets **(Fig. S7),** accurately reconstructing the cellular architecture in iPSCs, delineating major lineages in HCC, and resolving functionally distinct T cell subsets—tasks in which AS-only or EXP-only models performed suboptimally.

We further compared AEnet with established splicing-aware clustering methods, including SCASL [31] and scQuint [32]. Across all three datasets, AEnet consistently outperformed SCASL in clustering accuracy, with higher ARI scores: iPSC (0.81 vs. 0.37), T cell (0.42 vs. 0.29), and HCC (0.58 vs. 0.35) **(Fig. 2E–G)**. In the iPSC dataset, SCASL failed to distinguish iPSCs from NPCs, while AEnet clearly separated these populations **(Fig. S8A)**. In the HCC dataset, AEnet effectively resolved lymphoid, myeloid, and malignant epithelial lineages, whereas SCASL showed poor separation between immune cell types **(Fig. S8B)**. Similarly, in the T cell dataset, SCASL generated overlapping clusters, failing to delineate functional T cell subsets, in contrast to the well-separated clusters produced by AEnet **(Fig. S8C)**. Since scQuint primarily uses a variational autoencoder (VAE) to generate embeddings, we applied clustering to the scQuint-derived embeddings using default parameters. In the iPSC dataset—characterized by relatively simple cellular composition—AEnet and scQuint showed comparable performance. However, in the more complex T cell and HCC datasets, scQuint produced overlapping clusters and failed to resolve key subpopulations **(Fig. S8D)**. Together, these results confirm that joint modeling of alternative splicing and gene expression enables AEnet to more accurately capture cellular heterogeneity compared to other splicing-based methods.
