# Supplementary material for: AEnet: a practical tool to construct the splicing-associated phenotype atlas at a single cell level
Source: Gigascience. 2025 Sep 24;14:giaf110. doi: 10.1093/gigascience/giaf110 (PMC12457822; doi:10.1093/gigascience/giaf110)
Supplement: giaf110_Supplemental_Files [file giaf110_supplemental_files.zip › FIG.S10.pdf]

584

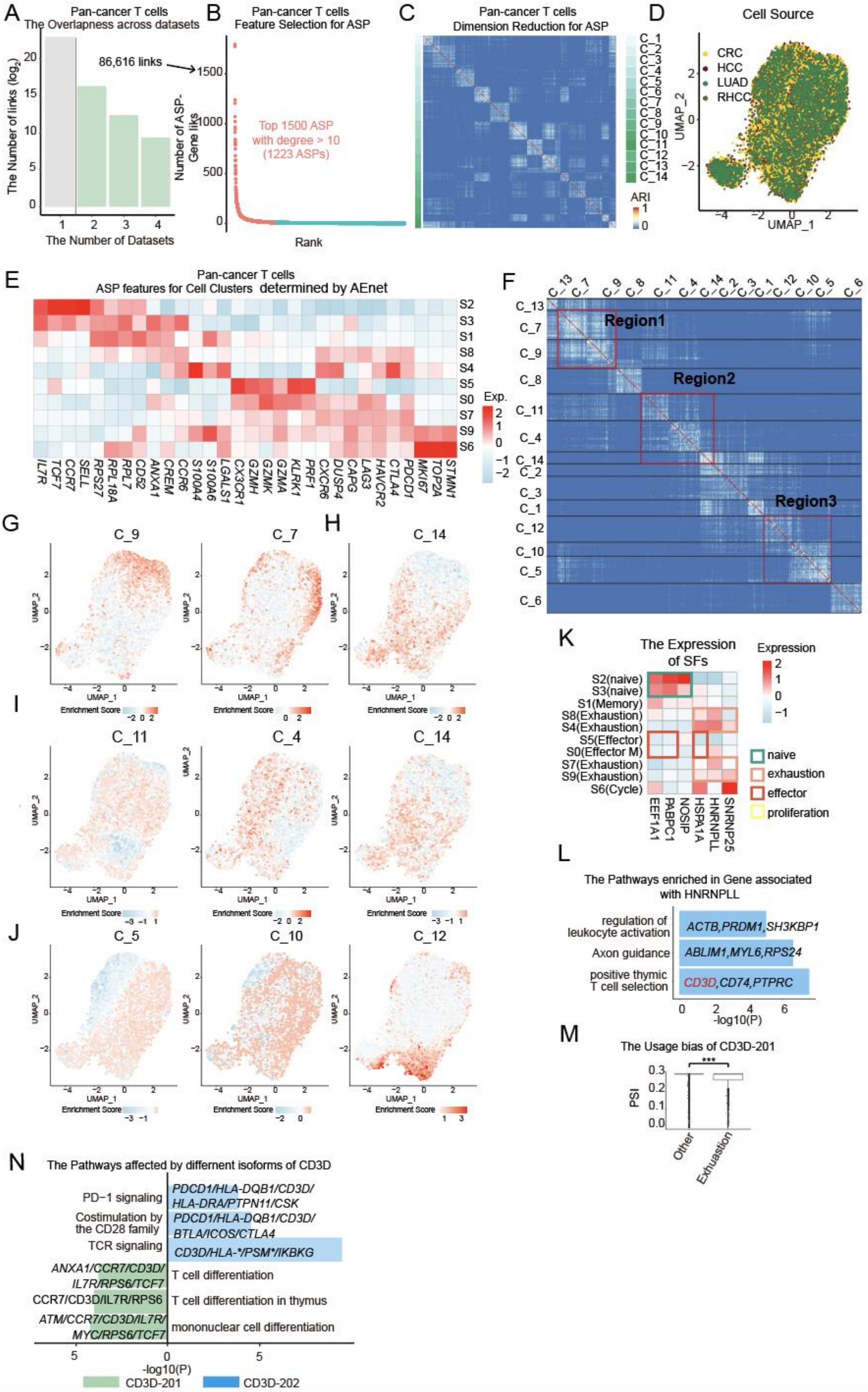

A
Pan-cancer T cells
The Overlapness across datasets
86,616 links
The Number of links (log2)
The Number of Datasets
B
Pan-cancer T cells
Feature Selection for ASP
Top 1500 ASP
with degree > 10
(1223 ASPs)
Number of ASP-Gene liks
Rank
C
Pan-cancer T cells
Dimension Reduction for ASP
ARI
D
Cell Source
CRC
HCC
LUAD
RHCC
UMAP_1
UMAP_2
E
Pan-cancer T cells
ASP features for Cell Clusters determined by AEnet
Exp.
F
Region1
Region2
Region3
G
H
I
J
Enrichment Score
K
The Expression of SFs
Expression
S2(naive)
S3(naive)
S1(Memory)
S8(Exhaustion)
S4(Exhaustion)
S5(Effector)
S0(Effector M)
S7(Exhaustion)
S9(Exhaustion)
S6(Cycle)
EEF1A1
PABPC1
NOSIP
HSPA1A
HNRNPLL
SNRNP25
naive
exhaustion
effector
proliferation
L
The Pathways enriched in Gene associated with HNRNPLL
regulation of leukocyte activation
ACTB,PRDM1,SH3KBP1
Axon guidance
ABLIM1,MYL6,RPS24
positive thymic T cell selection
CD3D,CD74,PTPRC
-log10(P)
M
The Usage bias of CD3D-201
PSI
Other
Exhaustion
N
The Pathways affected by different isoforms of CD3D
PD−1 signaling
PDCD1/HLA-DQB1/CD3D/HLA-DRA/PTPN11/CSK
Costimulation by the CD28 family
PDCD1/HLA-DQB1/CD3D/BTLA/ICOS/CTLA4
TCR signaling
CD3D/HLA-*/PSM*/IKBKG
ANXA1/CCR7/CD3D/IL7R/RPS6/TCF7
T cell differentiation
CCR7/CD3D/IL7R/RPS6
T cell differentiation in thymus
ATM/CCR7/CD3D/IL7R/MYC/RPS6/TCF7
mononuclear cell differentiation
-log10(P)
CD3D-201
CD3D-202

Figure S10. AEnet reveals cellular splicing heterogeneity and its key splicing events in tumor-infiltrating T cells across various cancer types. A. The barplot displays the selection of high quality links in the AEnet of Pan-cancer T cells. **B.** The selection of key alternative splicing patterns. **C.** The heatmap shows the ASP cluster from dimension reduction of AEnet. **D.** UMAP displays cell sources. **E.** The heatmap displays the expression of marker genes across cell clusters determined by AS. **F.** Heatmap of ASP clusters ordered by similarity, illustrating inter-cluster relationships within the pan-cancer T cell dataset. **G-J.** Distribution of enrichment scores for selected ASP clusters. **K.** The expression of key splicing factors across clusters determined by alternative splicing. **L.** The pathways enriched in genes with ASP associated with HNRNPLL. **M.** The PSI distribution of CD3D-201 isoform within CD3D within exhaustion and other T cells. Statistical analysis was performed using the Student's t-test. **N.** The pathways enriched in the gene sets with different isoforms of CD3D.

AEnet Uncovers Transitional Cell States and Key Splicing Factors in Embryonic Gastrulation

During embryogenesis, alternative splicing is a key mechanism that fine-tunes developmental pathways and controls cell fate decisions. Here, we apply AEnet to interrogate a scRNA-seq dataset of gastrulation-stage human embryos from the Human Developmental Biology Resource, elucidating how the AS process enables precise regulation of gene expression at this stage. The reference dataset comprises 1,195 cells (665 caudal, 340 rostral, and 190 yolk sac cells), with a median of 4,000 genes detected per cell[48].

Following the standard AEnet workflow, we first identified a total of 1,604 ASP events, 25 ASP clusters, and 11 cell populations (**Fig. 5A and S11A-C**). The sequential differentiation trajectory from epiblast cells (cell subpopulation 5, S5) to the primitive streak (S1), followed by the transition to endodermal cells (S6/S10) or mesoderm (S2/S0), ultimately leading to axial mesoderm (S7), was clearly discernible (**Fig. 5A, right panel**) [49]. Notably, the ASPs are assumed to be highly distinct among different cell types during embryogenesis. As a result, AEnet factorized the cell populations in a manner similar to those clustered based solely on RNA profilings (**Fig. 5B**, ARI = 0.304).

Epiblast cells (S5) are classic pluripotent stem cells, derived from the inner cell mass of the blastocyst and capable of differentiating into the three germ layers. For the highly correlated ASP clusters C_13 and C_17, PSIP1 and SNRPN were identified as key regulatory factors that maintain cell stemness (**Fig. 5C-D, S11D-E**). HNRNPAB and SRSF3 were associated with splicing decisions that resulted in longer junctions spanning genomic loci, observed more frequently in epiblast cells than in cells with reduced stemness (**Fig. 5E**). We verified this intriguing finding in an independent iPSC dataset (**Fig. S11F**)[18].
