# Supplementary material for: AEnet: a practical tool to construct the splicing-associated phenotype atlas at a single cell level
Source: Gigascience. 2025 Sep 24;14:giaf110. doi: 10.1093/gigascience/giaf110 (PMC12457822; doi:10.1093/gigascience/giaf110)
Supplement: giaf110_Supplemental_Files [file giaf110_supplemental_files.zip › FIG.S11.pdf]

patterns of TNRC6B and the PSI distribution. **J.** The pathways enriched in the gene sets with different isoforms of TNRC6B. **K.** The illustration depicts the developmental trajectory and highlights the top-ranked key splicing factors based on their relative importance. * $p < 0.05$, ** $p < 0.01$, *** $p < 0.001$.

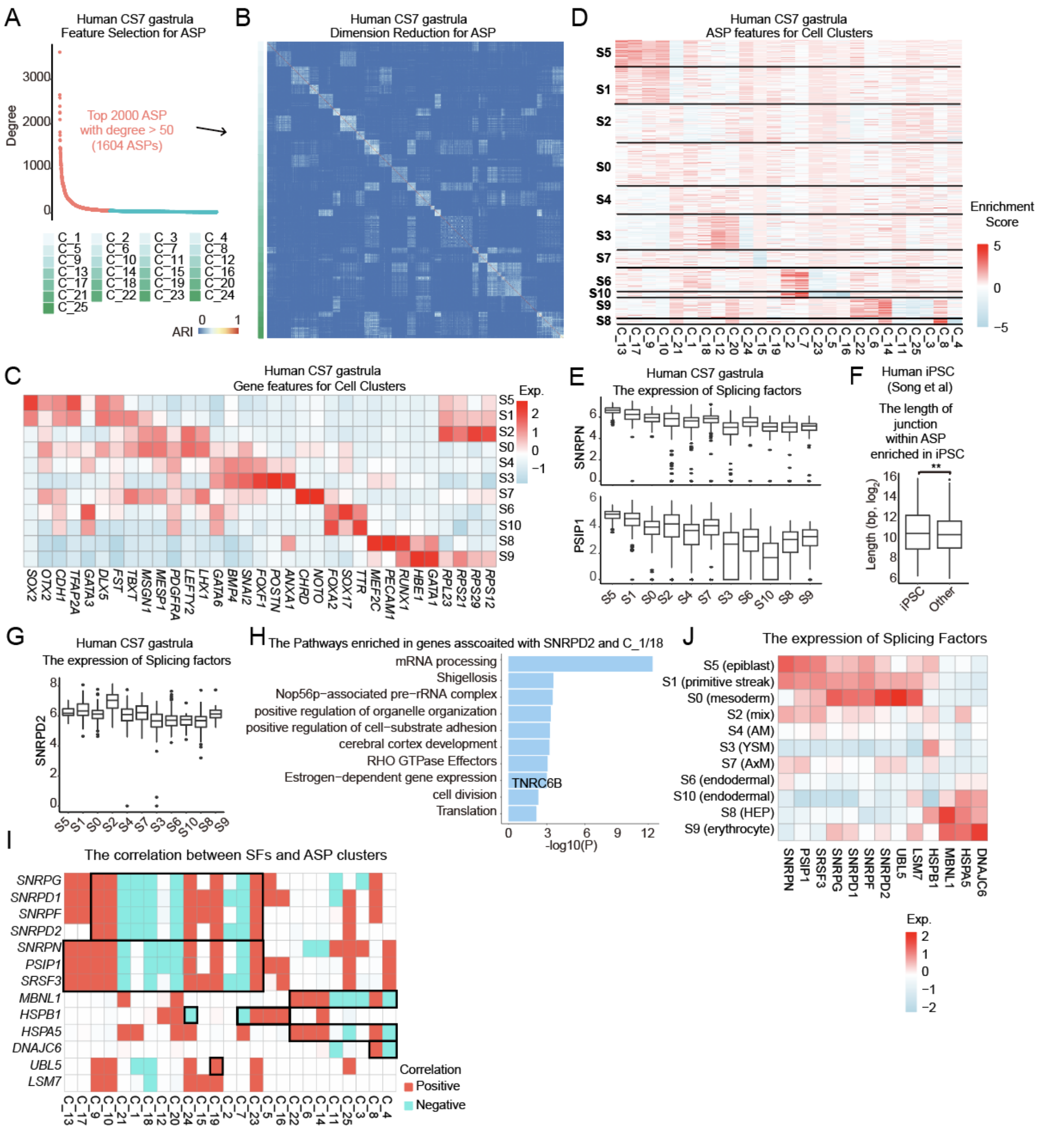

Figure S11. AEnet reveals intermediate cell status during gastrulation. A. The selection of key alternative splicing patterns. **B.** The heatmap shows the ASP clusters from dimension reduction of AEnet. **C.** The heatmap displays the expression of developmental markers across cell clusters determined by AS. **D.** The heatmap displays the enrichment score of ASP clusters across cell clusters determined by AS. **E.** The expression of SNRPN and PSIP1 across the cell clusters determined by AS. **F.** The length of junction that is differentially used in different cell types from Song et al. Statistical analysis was performed using the Student's t-test. **G.** The expression of SNRPD2 across the cell clusters determined by AS. **H.** The pathways enriched in the
