# Supplementary material for: AEnet: a practical tool to construct the splicing-associated phenotype atlas at a single cell level
Source: Gigascience. 2025 Sep 24;14:giaf110. doi: 10.1093/gigascience/giaf110 (PMC12457822; doi:10.1093/gigascience/giaf110)
Supplement: giaf110_Supplemental_Files [file giaf110_supplemental_files.zip › FIG.S7-S9.pdf]

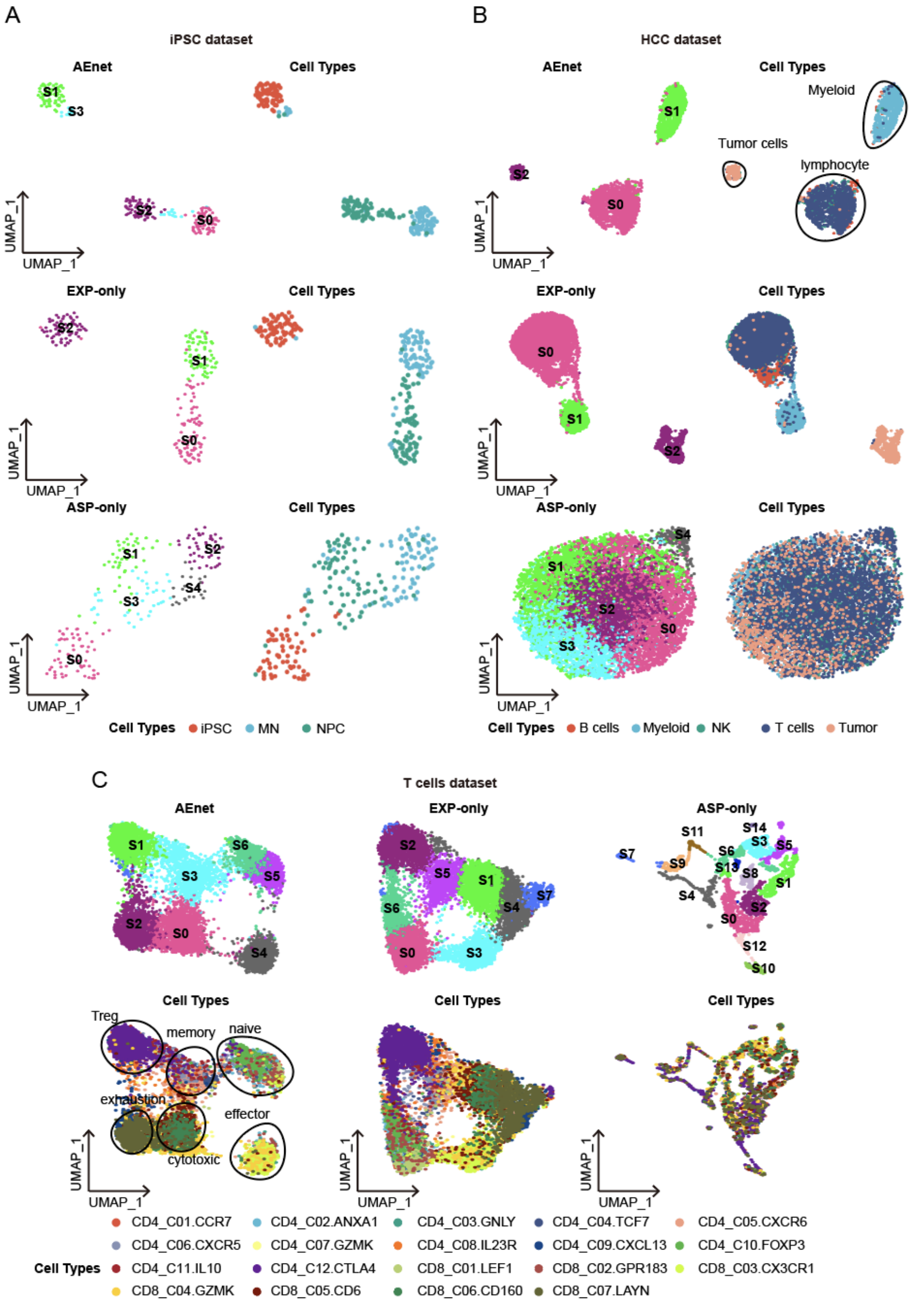

Figure S7. Ablation analysis for AEnet. A-C. Uniform Manifold Approximation and Projection (UMAP) visualizations showing the published cell type annotations (right) and the predicted clusters (left) derived from the ASP-EXP (AEnet) network (top), expression-only (EXP) network (middle), and ASP-only network (bottom) for (A) full-length iPSC and (B) hepatocellular carcinoma (HCC) single-cell RNA-seq datasets. (C) UMAP visualizations showing the published cell type annotations (bottom) and the predicted clusters (upper) derived from the ASP-EXP (AEnet) network (left), expression-only (EXP) network (middle), and ASP-only network (right) for the T cells datasets. Clustering solutions shown represent those with the highest adjusted Rand index (ARI).

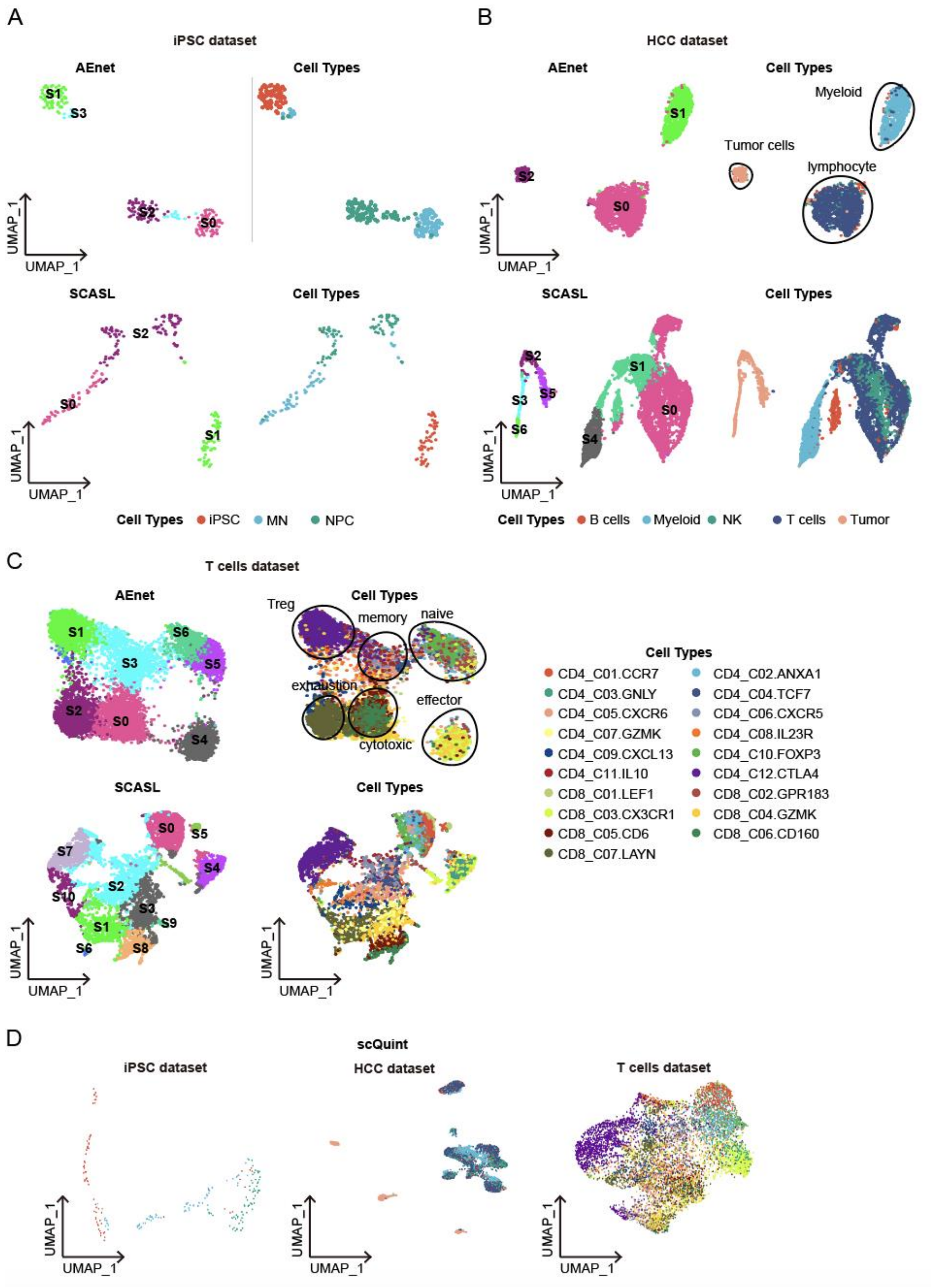

Figure S8. Benchmarking AEnet with SCAL and scQuint. A-C. UMAP visualizations showing the published cell type annotations (right) and the predicted clusters (left) derived from AEnet (upper) and SCASL (bottom) for: (A) full-length iPSC (B) hepatocellular carcinoma (HCC) single-cell RNA-seq datasets, and the (C) T cell dataset. Clustering solutions shown represent those with the highest adjusted Rand index (ARI). **D.** UMAP visualizations demonstrate the relative performance of scQuint (right) in: full-length iPSC dataset, T cells sequencing data, and hepatocellular carcinoma (HCC) single-cell RNA-seq data.

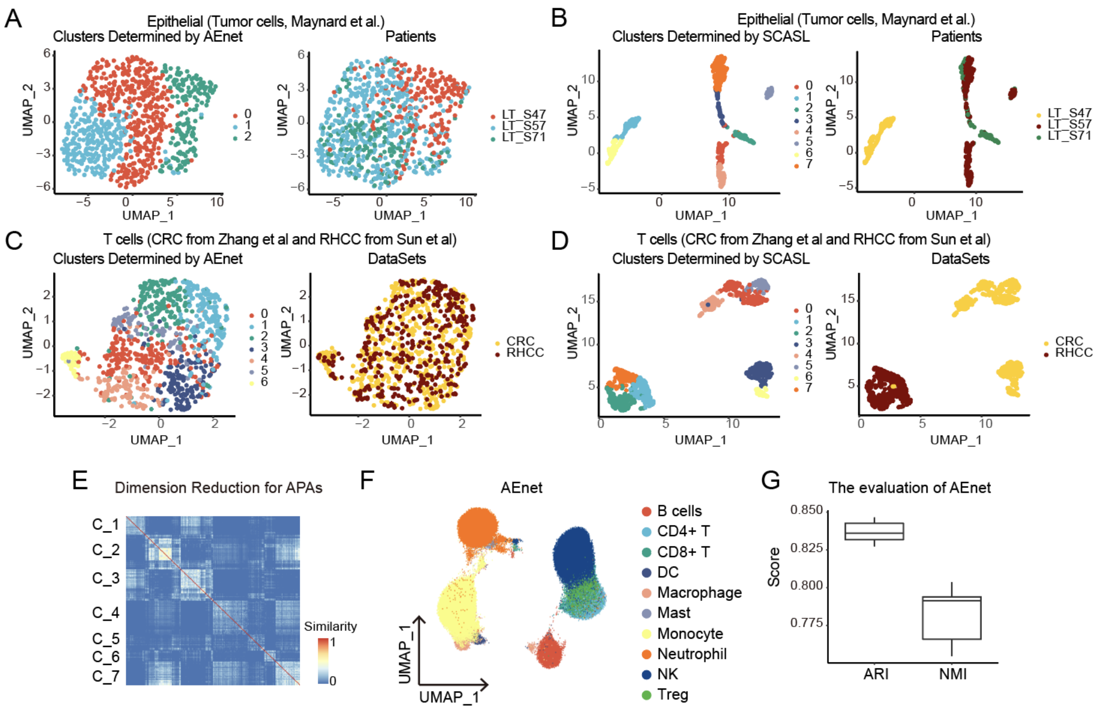

Figure S9. Performance comparison of AEnet and SCASL. A-B. UMAP shows the clustering of cell types determined by AEnet **(A)** and SCASL **(B)** (left panel) and patient clustering (right panel) for epithelial cells from multiple patients. **C-D.** UMAP shows the clustering of cell types determined by AEnet **(C)** and SCASL **(D)** (left panel) and patient clustering (right panel) for T cells from the CRC (Colorectal cancer) and RHCC (Recurrent Hepatocellular carcinoma) datasets. **E.** Heatmap showing APA classes derived from dimensionality reduction using AEnet. **F.** UMAP visualization of cell clustering based on alternative polyadenylation profiles. **G.** Quantitative benchmarking of clustering performance using ARI and NMI, evaluated against the ground truth annotations of the PBMC dataset.

AEnet resolves tumor heterogeneity bias and identifies immunotherapy-nonresponsive tumor subpopulations

Due to inherent intra- and inter-tumor heterogeneity, grouping malignant cells solely based on either gene expression profiles or ASPs is challenging. Here, we showcase the power of AEnet in untangling the intricate ASP-EXP relationships using data from 1,286 tumor cells from six lung cancer patients with varying responses to immunotherapy, classified as normal (N), residual disease (RD), and progressive disease (PD) after therapy [33].

Based on the AEnet algorithm, the ASP similarity matrix revealed a distinct separation into six ASP clusters, which resulted in three cell subpopulations (**Fig. 3A-B**). The AEnet-defined clusters exhibited biased distribution across response groups, with PD dominated by S2, RD primarily comprising S0 cells, and N group containing the
