# Supplementary material for: AEnet: a practical tool to construct the splicing-associated phenotype atlas at a single cell level
Source: Gigascience. 2025 Sep 24;14:giaf110. doi: 10.1093/gigascience/giaf110 (PMC12457822; doi:10.1093/gigascience/giaf110)
Supplement: giaf110_GIGA-D-25-00064_Original_Submission [file giaf110_giga-d-25-00064_original_submission.pdf]

GigaScience

AEnet: a practical tool to construct the splicing associated phenotype atlas at single cell level

--Manuscript Draft--

**Manuscript Number:**	GIGA-D-25-00064
**Full Title:**	AEnet: a practical tool to construct the splicing associated phenotype atlas at single cell level
**Article Type:**	Research
**Funding Information:**	GuangDong Basic and Applied Basic Research Foundation (2021A1515110832) — Miss Liang Wu
	Shenzhen Key Laboratory of Single-Cell Omics (ZDSYS20190902093613831) — Miss Liang Wu
	Shenzhen Science and Technology Program (LCYX20220620105200001) — Miss Liang Wu
	Key Technologies Research and Development Program of Anhui Province (2021YFC2501900) — Miss Liang Wu
	Shenzhen Science and Technology Program (JCYJ20240813150001003) — Dr Xi Chen
**Abstract:**	Alternative splicing (AS), a crucial driver of proteomic diversity, is a fundamental source of cellular heterogeneity alongside gene expression levels. AS is closely linked to various physiological and pathological processes, including tumor progression and embryonic development. Single-cell RNA sequencing (scRNA-seq) technologies capture AS events through junction reads at cellular resolution, enabling the identification of core AS events that regulate specific cell types or states. However, single-cell sequencing technology and its data are plagued by inherent limitations such as shallow sequencing depth, high dropout rates, and batch effects. Furthermore, previous clustering approaches have overlooked the crucial interplay between alternative splicing (AS) and gene expression in defining distinct 'cell types,' posing ongoing challenges in this field. In this study, we present a novel method called the AEnet, which combines gene expression levels with AS patterns to profile cellular heterogeneity and define what we term 'cell subpopulations'. AEnet also identifies key AS events and infers the regulatory mechanisms underlying these events. By applying AEnet to tumor cells, pan-cancer immune cells, and embryonic cells, we demonstrate enhanced cell clustering, the identification of novel AS events with potential functional importance, and the discovery of the key splicing factors involved in cell state transformation. The application of AEnet provides new insights into cellular heterogeneity and its role in both physiological and pathological processes.
**Corresponding Author:**	Liang Wu<br><br>CHINA
**Corresponding Author Secondary Information:**	
**Corresponding Author's Institution:**	
**Corresponding Author's Secondary Institution:**	
**First Author:**	Liang Wu
**First Author Secondary Information:**	
**Order of Authors:**	Liang Wu
	Shang Liu

Powered by Editorial Manager® and ProduXion Manager® from Aries Systems Corporation

	Xi Chen
	Yinqi Bai
	Shiping Liu
	Xiaohu Huang
	Yuhang Wang
	Waidong Huang
	Pengfei Qin
	Rui Li
	Xuanxuan Zou
	Wending Pang
**Order of Authors Secondary Information:**	
**Additional Information:**	
**Question**	**Response**
Are you submitting this manuscript to a special series or article collection?	No
**Experimental design and statistics**<br><br>Full details of the experimental design and statistical methods used should be given in the Methods section, as detailed in our Minimum Standards Reporting Checklist. Information essential to interpreting the data presented should be made available in the figure legends.<br><br>Have you included all the information requested in your manuscript?	Yes
**Resources**<br><br>A description of all resources used, including antibodies, cell lines, animals and software tools, with enough information to allow them to be uniquely identified, should be included in the Methods section. Authors are strongly encouraged to cite Research Resource Identifiers (RRIDs) for antibodies, model organisms and tools, where possible.<br><br>Have you included the information requested as detailed in our Minimum Standards Reporting Checklist?	Yes

**Availability of data and materials**<br><br>All datasets and code on which the conclusions of the paper rely must be either included in your submission or deposited in publicly available repositories (where available and ethically appropriate), referencing such data using a unique identifier in the references and in the "Availability of Data and Materials" section of your manuscript.<br><br>Have you have met the above requirement as detailed in our Minimum Standards Reporting Checklist?	Yes
GigaScience has policies and guidelines in place for the use of generative AI-writing tools such as ChatGPT. If you have used such writing tools to assist with writing the manuscript this must be declared and cited in the text. Authors should not list AI-writing tools and other AI-assisted technologies as an author or co-author and should acknowledge that they are fully responsible for text generated or refined by AI-writing tools.<p><br><br>A summary of use (particularly in the introduction or among methods) needs to be included at the end of the paper, and the outputs should also be included as a supplementary file hosted in GigaDB or other open repositories. Please read our guidelines for more information. <p><br><br>By submitting to GigaScience, you are aware of the journal's AI-writing tools policy, and if you have declared use of such tools below, you have acknowledged this where appropriate in your manuscript and have made a summary of use and outputs available. <p><br><b>AI-assisted writing tools have been used in the preparation of this manuscript?	No

Powered by Editorial Manager® and ProduXion Manager® from Aries Systems Corporation

A manuscript containing both the content and figures

Click here to access/download;Manuscript;AEN Manuscript_1001.pdf

AEnet: a practical tool to construct the splicing associated phenotype atlas at single cell level

Shang Liu[1*], Xi Chen[1,2,7,*], Xiaohu Huang[1,2,3], Yuhang Wang[1,2,3], Waidong Huang[1,2,4], Pengfei Qin[1,2,7], Rui Li[2], Xuanxuan Zou[1,2], Wending Pang[1,2,3], Shiping Liu[8,9,#], Yinqi Bai[10,#], Liang Wu[1,2,5,6,7#]

1. BGI Research, Chongqing 401329, China
2. BGI Research, Shenzhen 518083, China
3. School of Biology and Biological Engineering, South China University of Technology, Guangzhou, China.
4. College of Life Sciences, University of Chinese Academy of Sciences, Beijing, China
5. Zhongshan-BGI Precision Medical Center, Zhongshan Hospital, Fudan University, Shanghai, China.
6. Shanxi Medical University-BGI Collaborative Center for Future Medicine, Shanxi Medical University, Taiyuan 030001, China
7. State Key Laboratory of Genome and Multi-omics Technologies, BGI Research, Shenzhen 518083, China
8. BGI Research, Hangzhou 310030, China
9. State Key Laboratory of Genome and Multi-omics Technologies, BGI Research, Hangzhou 310030, China
10. BGI Research, Sanya 572025, China

* These authors contributed equally

# Correspondence: wuliang@genomics.cn; baiyinqi@genomics.cn; liushiping@genomics.cn

Abstract

Alternative splicing (AS), a crucial driver of proteomic diversity, is a fundamental source of cellular heterogeneity alongside gene expression levels. AS is closely linked to various physiological and pathological processes, including tumor progression and embryonic development. Single-cell RNA sequencing (scRNA-seq) technologies capture AS events through junction reads at cellular resolution, enabling the identification of core AS events that regulate specific cell types or states. However, single-cell sequencing technology and its data are plagued by inherent limitations such as shallow sequencing depth, high dropout rates, and batch effects. Furthermore, previous clustering approaches have overlooked the crucial interplay between alternative splicing (AS) and gene expression in defining distinct 'cell types,' posing ongoing challenges in this field. In this study, we present a novel method called the AEnet, which combines gene expression levels with AS patterns to profile cellular heterogeneity and define what we term 'cell subpopulations'. AEnet also identifies key AS events and infers the regulatory mechanisms underlying these events. By applying AEnet to tumor cells, pan-cancer immune cells, and embryonic cells, we demonstrate enhanced cell clustering, the identification of novel AS events with potential functional importance, and the discovery of the key splicing factors involved in cell state transformation. The application of AEnet provides new insights into cellular heterogeneity and its role in both physiological and pathological processes.

Introduction

The diversity of proteomes is an important manifestation of the complexity of organisms, and alternative splicing (AS) is one of the major factors contributing to this diversity [1,2]. AS plays a crucial role in various physiological and pathological processes such as embryonic development [3], aging [4], and tumor progression [5,6]. Several key splicing factors are involved in the regulation of AS during disease progression. For instance, RBFOX2 is a master regulator for mesenchymal tissue-specific splicing [7], playing a significant role in the formation of mesenchymal-like states in tumor cells [8]. Recently, single-cell transcriptomics has become a powerful tool for analyzing profilings of AS at high resolution [9,10]. Aviv Regev's lab used this technology to construct a full-length transcriptome map of thirty-six dendritic cells and found that IRF7 underwent differential AS events at the single-cell level, revealing changes in alternative splicing that were previously obscured at the bulk-level [11].

Although there are several bioinformatic approaches to studying single-cell AS profiling, most are based on canonical RNA-based clustering into cell types and then comparing AS heterogeneity, similar to differential gene expression analysis, between these predefined cell types. Since AS events can also reflect inherent splice site preferences across different cell states, these methods can sometimes lead to inflated false negatives, as cell states become confounders in the RNA-based classification of cell types. This is exemplified by BRIE [12], Outrigger [13], MARVEL [14], and our earlier attempt, DESJ-detection [15]. Furthermore, existing single-cell analysis tools lack the extensibility to reveal the regulatory mechanisms of alternative splicing, infer their regulatory factors, and identify functional pathways, thus limiting an in-depth understanding of how alternative splicing influences cell cells and cell states [16,17].

Several issues require further consideration in the design of a single-cell AS analytical pipeline. First, unlike RNA expression, which is typically quantified in absolute values, AS events are usually quantified as proportional values. The sparsity of scRNA-seq data often introduces the "NaN" (not a number) challenge during calculations when the denominator (the total number of AS events for a given gene in a single cell) is zero. Second, AS events can also be affected by batch effects, an inherent limitation of single-cell techniques. Third, not all AS events contribute functionally to cellular heterogeneity. Therefore, both upstream and downstream approaches that account for filtering processes should be incorporated.

To address these challenges, we present the Alternative splicing-gene Expression Network (AEnet, https://github.com/liushang17/AEnet) to explore core AS events and gene co-expression patterns in a network at the single-cell level. Using our algorithm, we find that both splice site preferences and gene expressions contribute to cellular heterogeneity during clustering, though they exhibit dynamic interplays and varying weights across different datasets. We refer to the separated clusters as cell (sub)populations to avoid confusion with either cell types or cell states. The software has three major functions: first, to construct AS profiling-based clusters and separate cell subpopulations with distinct AS-gene expression networks; second, to identify key splicing factors for AS clusters (analogous to gene markers); and third, to pinpoint functional pathways involved in the regulatory mechanisms based on core subsets of AS events.

By applying the AEnet method to malignant cells with different immunotherapy responses, T cell analysis in pan-cancer, and cell differentiation during embryonic gastrulation, we demonstrate the power of AEnet in fine-grained clustering of cells by disease or developmental states, seamlessly linking upstream regulatory factors and downstream action pathways, highlighting novel isoforms of functional importance, and constructing alternative splicing landscapes along the AS-based developmental trajectory. These findings will deepen our understanding of the role of alternative splicing in tumorigenesis and embryonic development, providing new strategies and

ideas for clinical prognosis prediction, tumor immunotherapy, and congenital disease treatment.

Results

The overview of AEnet

We have developed the AEnet method that integrates alternative splicing with gene expression levels to uncover cellular splicing heterogeneity and underlying regulatory mechanisms. In brief, AEnet begins by quantifying alternative splicing patterns (ASP) using junction reads from individual cells during data preprocessing (**Fig. 1A**). The percent spliced-in (PSI) for a specific AS event is defined as the proportion of junction reads curated from all detected junctions that span the same site (**Fig. 1A, bottom panel**). Notably, unlike expression values, which are always nonnegative integers, PSI is assigned as NaN (resulting from division by 0) when no junction reads are detected at a presupposed AS site in a cell (**Fig. S1A**). In scRNA-seq data, the prevalent RNA dropout and shallow sequencing issues make the occurrence of NaN even more challenging within individual cells.

To mitigate this effect, for each ASP and each gene, we limit the calculation of the correlation (Spearman by default) to cells with valid PSI values and the gene's expression level, retaining only those ASP-expression correlation links (referred to as ASP-EXP links) with significant P values (**Fig. 1B**). These statistically significant links indicate potential relationships between gene expression dynamics and the usage preference of specific ASPs across the cells. When multiple scRNA-seq samples are available under any experimental conditions, we retain only ASP-EXP links that share the same correlation trend and appear at a moderately higher frequency (2 by default) to exclude batch effect-induced artifacts (**Fig. 1B**). These steps allow AEN to overcome technical noise and capture common relationships between AS and gene expression.

We hypothesize that the interactions identified in common ASP-EXP links play either direct or indirect roles in post-transcriptional regulation and gene expression diversity. For instance, increased expression of specific splicing factors may promote or inhibit the inclusion of certain exons, thereby modifying the AS profile of target genes (**Fig. S1B**)[18]. In other cases, both alternative splicing and gene expression levels may be dysregulated, leading to aberrant gene function during stress responses or in diseases[19]. We then rank the most frequent ASPs (n = 1500, by default) by summing the common ASP-EXP links associated with each ASP, defining them as anchor ASPs (**Fig. S1C**). For each pair of anchor ASPs, we calculate the Jaccard metric of ASP-EXP links (where genes linked to the ASP are treated as sets) to represent their similarity, thereby constructing a similarity matrix of anchor ASPs for downstream clustering (**Fig. 1B**). The clustering process aims to separate the ASPs into distinct

groups, each representing a group of AS events correlated with a set of co-expressed or co-regulated genes (**Fig. S1D**). We then construct a gene graph for each ASP cluster, where the genes are nodes and the number of ASPs represents the weight of the edges. Hub genes are defined as the network-connected genes that pass the edge threshold (surpassing half the number of ASPs, by default) (**Fig. S1E**). These genes are used to infer the cell states or functional programs that are highly associated with specific ASP clusters through gene set or pathway enrichment analyses.

Taken together, the AEnet pipeline begins with the detection of alternative splicing patterns (ASPs) at the cellular level, then constructs ASP (of gene i)–expression (of gene j) links (ASP-EXP links), and defines the ASP similarity matrix at the sample level. Ultimately, it uncovers specific ASPs and co-expression/regulatory patterns at the cell population level (**Fig. 1C**). The output of AEnet focuses on evaluating three main biological events (**Fig. 1C, S1F-H**): first, the separation of cell subpopulations with distinct ASP compositions; second, the key splicing factors that influence specific ASP clusters (analogous to gene markers); and third, the functional pathways activated or inhibited by individual or small subsets of ASPs. These downstream analyses demonstrate AEnet's capability in identifying cellular splicing heterogeneity and regulatory mechanisms.

Finally, to assess the performance of AENet, we focused on the core steps involved in its identification of ASP clusters. An ASPs-ASPs similarity (Jaccard index) matrix was generated with increasing levels of noise to evaluate AENet's effectiveness in ASP clusters prediction, **(Fig. S2A-B)**. Using a supervised hierarchical clustering method, AENet demonstrated a high accuracy consistency score of approximately 0.9 between the background and the clusters identified, even when noise levels reached 80% **(Fig. S2C-D)**. These results demonstrate AENet's robustness in identifying ASP clusters despite noise.

Additionally, compared to SCASL, the current main single-cell clustering method based on AS, AENet effectively mitigated batch effects and revealed cell AS heterogeneity **(Fig. S3)**. In lung cancer cell datasets, AENet was able to uncover common AS heterogeneity across multiple patients, whereas SCASL primarily identified AS heterogeneity driven by batch effects of patients **(Fig. S3A-B)**. In the CRC (colorectal cancer) and RHCC (recurrent hepatocellular carcinoma) T cell datasets, SCASL failed to reveal AS heterogeneity in T cells with minimal batch effects compared to tumor cells, whereas AENet successfully captured the AS heterogeneity **(Fig. S3C-D)**. In summary, AENet provides a reliable tool for ASP cluster identification, even in the presence of noise and batch effects, making it a valuable resource for large-scale, multi-sample analyses in complex biological systems.

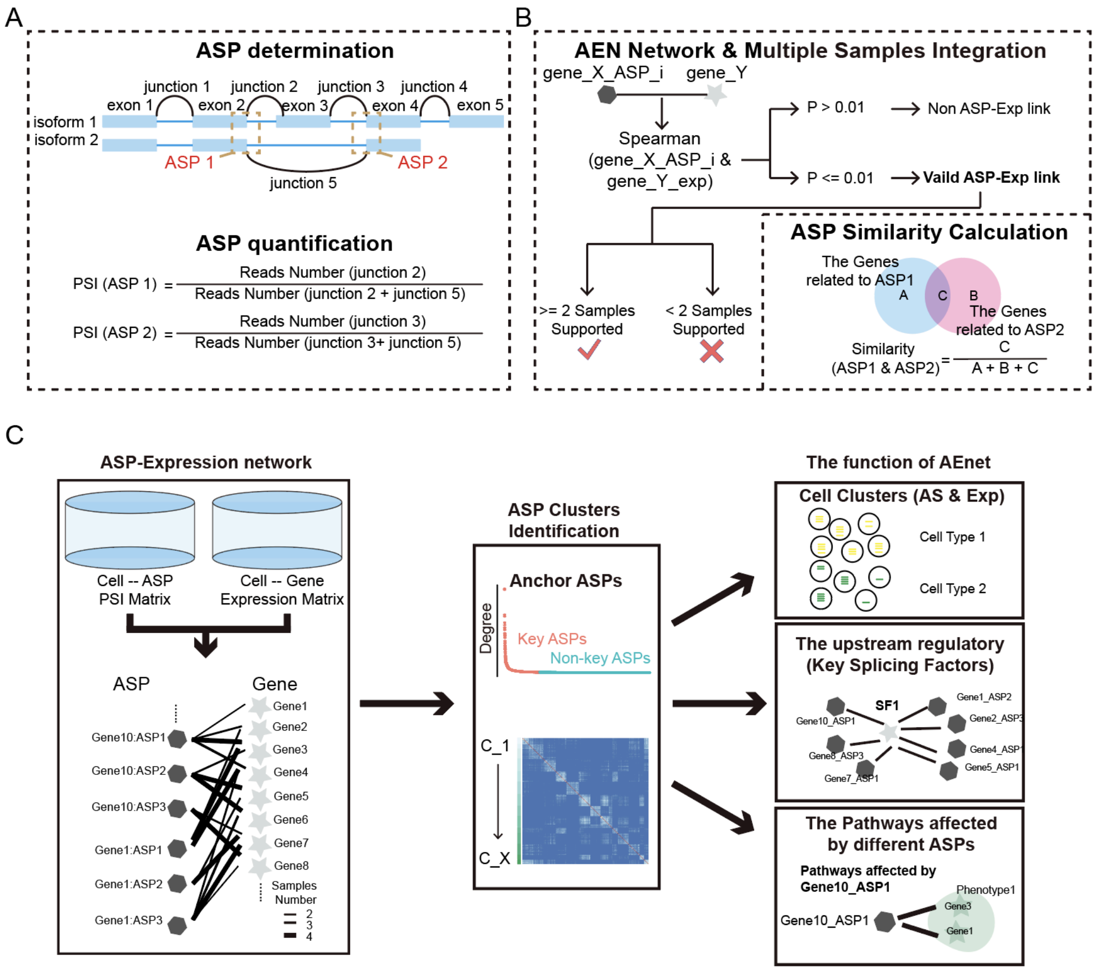

Figure 1. Schematic Diagram of the AEnet Method. A. Schematic Diagram of the identification of Alternative Splicing Pattern (ASP) and the calculation of PSI. **B.** Schematic Diagram of AEN Network Construction, Multiple Samples Integration, and ASP Similarity Calculation. **C.** Scheme of AEnet methods.

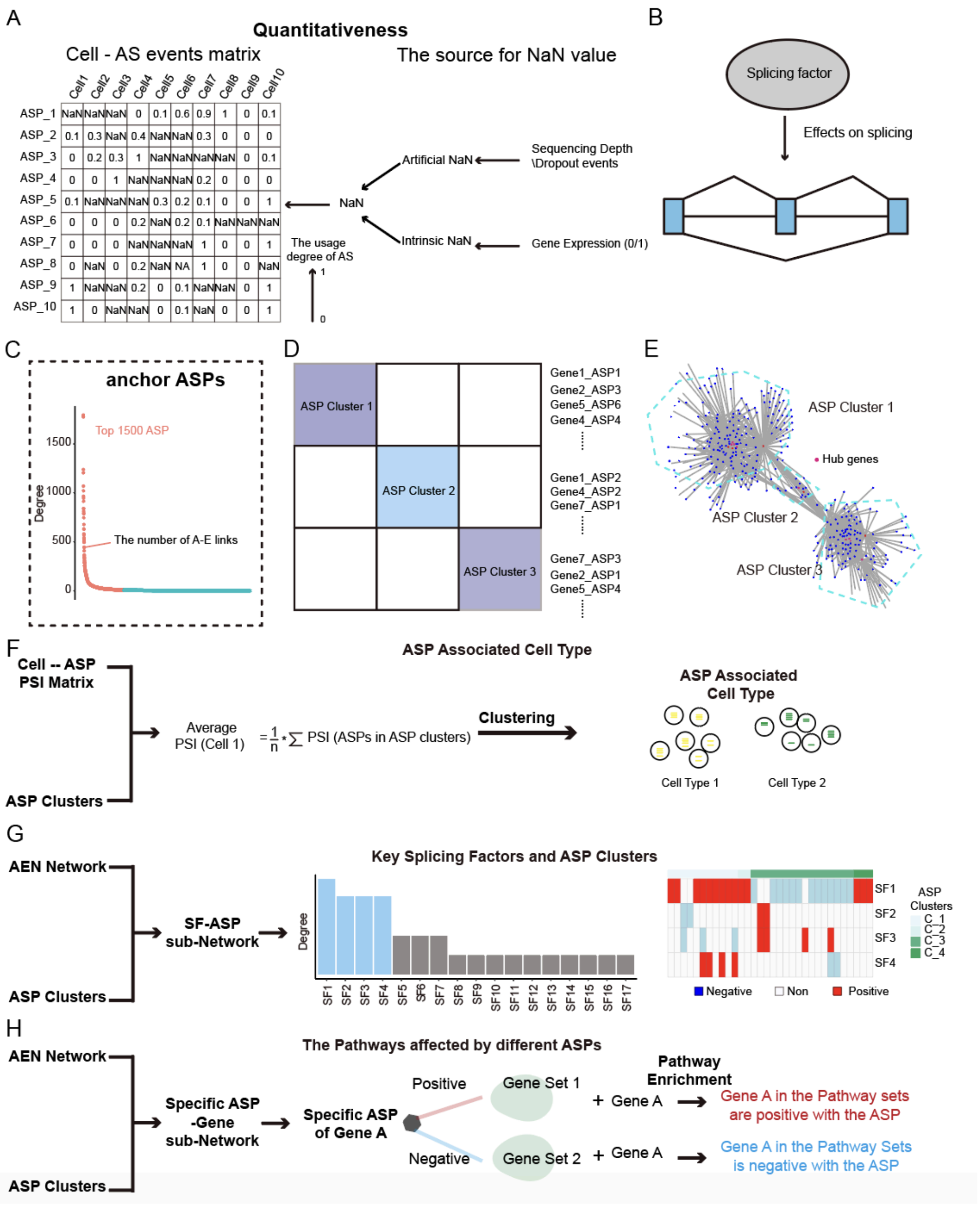

Figure S1. The key difficulties in cell splicing heterogeneity analysis. A. The NaN value problem and its source. **B.** Schematic diagram illustrating the effect of splicing factors on the selection of splicing patterns. **C.** The selection of anchor ASPs. **D-E.** The identification of ASP clusters (D) and Hub genes of ASP clusters (E). **F.** Schematic Diagram of the Analysis of Cell Splicing Heterogeneity. **G.** Identification of the Most Critical Splicing Factors Associated with the ASP Set. **H.** Schematic Diagram of Pathway Inference Associated with Different patterns of ASP.

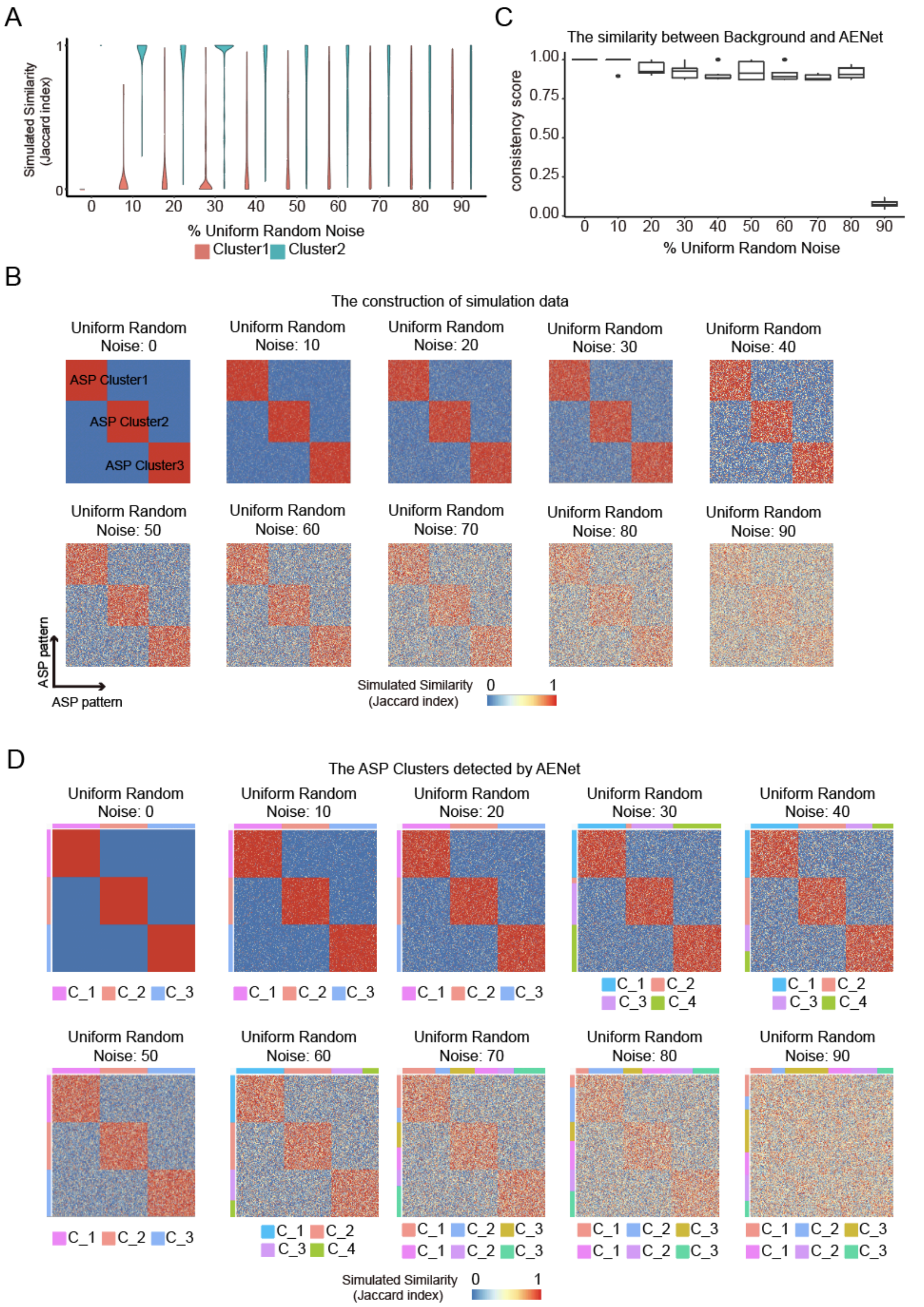

Figure S2. The evaluation of ASP clusters prediction of AEnet. A-B. Simulated ASPs-ASPs similarity matrix **(B)** was created with increasing noise **(A)**. **C.** Box plots present the jaccard index of ASP clusters between the background and AENet. **D.** ASP clusters identified by AENet in the simulated datasets.

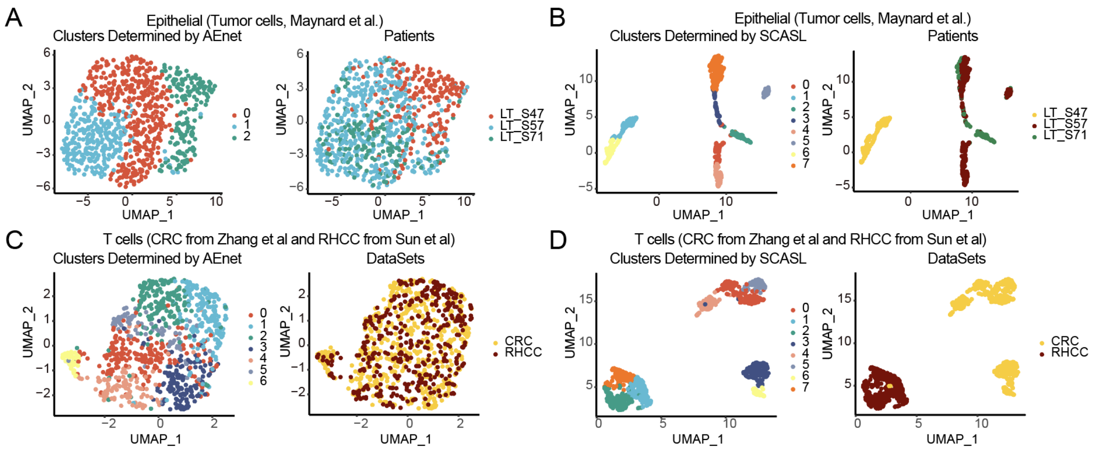

Figure S3. Performance comparison of AEnet and SCASL. A-B. UMAP shows the clustering of cell types determined by AEnet **(A)** and SCASL **(B)** (left panel) and patient clustering (right panel) for epithelial cells from multiple patients. **C-D.** UMAP shows the clustering of cell types determined by AEnet **(C)** and SCASL **(D)** (left panel) and patient clustering (right panel) for T cells from the CRC (Colorectal cancer) and RHCC (Recurrent Hepatocellular carcinoma) datasets.

AEnet reduces bias from tumor heterogeneity and identifies key subpopulations and splicing events involved in immunotherapy response.

Due to inherent intra- and inter-tumor heterogeneity, grouping malignant cells solely based on either gene expression profiles or ASPs is challenging. Here, we showcase the power of AEnet in untangling the intricate ASP-EXP relationships using data from 1,286 tumor cells from six lung cancer patients with varying responses to immunotherapy, classified as normal (N), residual disease (RD), and progressive disease (PD) after therapy [20].

Based on the AEnet algorithm, the ASP similarity matrix revealed a distinct separation into six ASP clusters, which resulted in three cell subpopulations (**Fig. 2A-B**). These identified cell populations were not solely determined by gene expression profiles, ASPs, or individual patient sources. Additionally, compared to SCASL, AENet effectively mitigated batch effects (**Fig. S4A-B**). The clusters determined by AEnet represented a transitional therapy-response trajectory from PD (cell subpopulation 2, denoted as S2), to RD (S0), to normal response (S1), progressing from left to right along the UMAP_1 axis (**Fig. 2C and S4C-D**). The marker genes of S1 (N) were enriched in alveolar signatures, including *AQP4*, *SFTPB/C/D*, *NKX2-1*, and *FOXA2* [21,22], while S2 (PD) was associated with elevated expression of prothrombin activation genes (*PLAT*, *PLAUR*), gap-junction proteins

(*GJB2/3/5*), and the well-known EMT (epithelial-mesenchymal transition) marker *EPCAM* (**Fig. 2D**) [23–25].

From the perspective of ASP clusters, C_4 (ASP cluster 4) was notably co-occurring with S2 while being excluded from S1 (**Fig. 2E**). The top-ranked hub genes in this cluster were IK and CELF2, which exhibited opposite expression trends between normal and PD cells (**Fig. 2F-G**). CELF2 is a crucial splicing factor, and its downregulation has been reported to promote tumor progression in both pancreatic and breast cancers [26,27]. The role of IK remains unclear; however, we found it to be upregulated in a CRC cohort as responses to immunotherapy deteriorated (**Fig. S4E**)[28].

In addition, C_4 was enriched in inflammation-associated pathways, including the response to type II interferon, positive regulation of lymphocyte proliferation, and the adaptive immune system (**Fig. 2F**). Among the key genes related to inflammatory responses, CD74, the HLA-DR antigens-associated invariant chain, is reported to exhibit dual oncogenic and tumor-suppressive roles depending on the cancer type and specific microenvironment. In this lung cancer dataset, we found that ASPs in CD74 were exclusively dominated by the isoforms CD74-201 and CD74-202. CD74-202 is reported as the soluble form, which suppressed melanoma cell growth and induced apoptosis under IFN-γ stimulatory conditions [29,30]. The significantly differentiated ratio of CD74-202 to CD74-201 could directly indicate post-therapy responses in different cell groups (**Fig. 2G**).

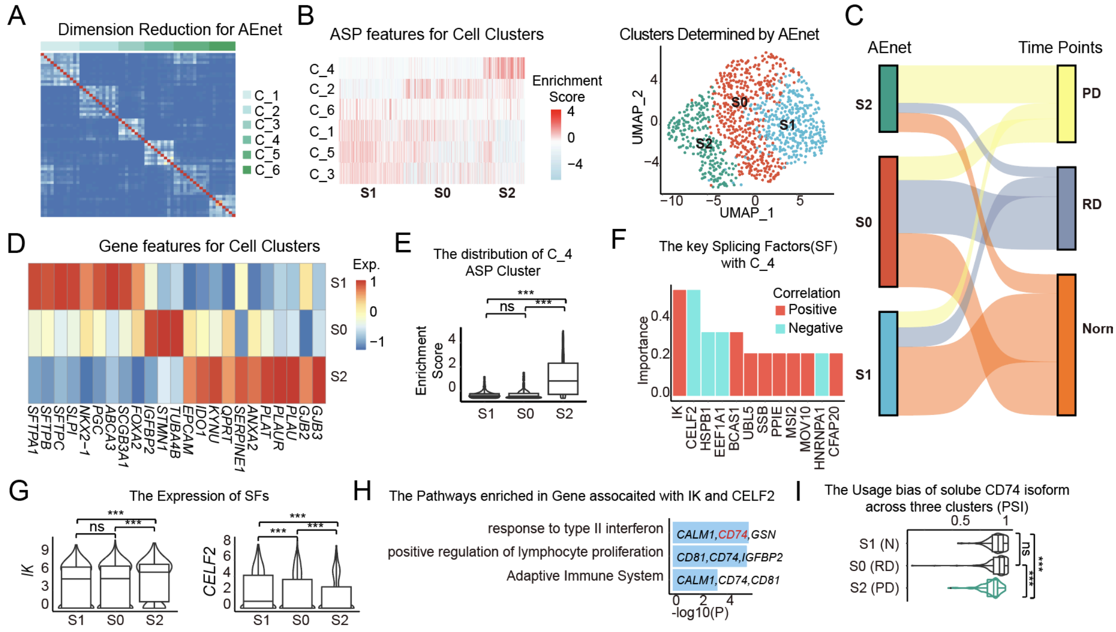

Figure 2. AEnet decreases the bias caused by tumor heterogeneity and uncover mechanistic insights in immunotherapy response. A. The heatmap shows the ASP clusters from dimension reduction of AEnet. **B.** The heatmap displays

the enrichment score of ASP clusters across cell clusters determined by AEnet (left panel). UMAP displays clustering of cell types determined by AEnet (right panel). **C.** The Sankey plot shows the overlapping of cells between Time Points and Clusters determined by AEnet. **D.** The heatmap displays the expression of marker genes across cell clusters determined by AEnet. **E.** The distribution of C_4 enrichment scores across cell clusters determined by AEnet. **F.** The barplot displays the importance of splicing factors in the formation of C_4 ASP clusters. The color represents the correlation relationship between splicing factors and C_4 ASP clusters. **G.** The expression of IK and CELF2 across cell clusters. Statistical analysis was performed using the Student's t-test. **H.** The top 3 pathways enriched for the genes with alternative splicing patterns in C_4 ASP clusters. **I.** The alternative splicing patterns of CD74 and the PSI distribution across 3 clusters. Statistical analysis was performed using the Student's t-test. * $p < 0.05$, ** $p < 0.01$, *** $p < 0.001$.

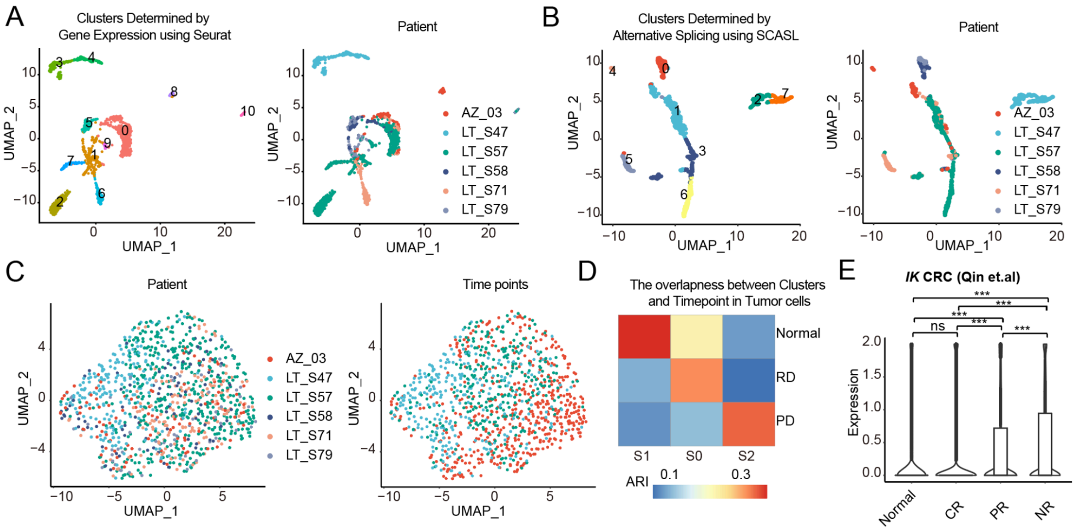

Figure S4. AEnet reduces bias from tumor heterogeneity and identifies key splicing factors involved in immunotherapy response. A. UMAP displays clustering of cell types determined by Gene Expression (left panel) and patients (right panel). **B.** UMAP displays clustering of cell types determined by Alternative splicing by SCASL (left panel) and patients (right panel). **C.** UMAP of AEnet displays cell source and time points (right panel). **D.** The heatmap shows the overlapping of cells between Time Points and Clusters determined by AEnet. **E.** The expression of IK within cells at different timepoints. Statistical analysis was performed using the Student's t-test. * $p < 0.05$, ** $p < 0.01$, *** $p < 0.001$.

AEnet reveals cellular splicing heterogeneity and its key splicing events in tumor-infiltrating T cells across various cancer types

The design of the AEnet algorithm largely bypasses batch effect issues in scRNA-seq analyses, making it particularly suitable for pan-cancer studies. To explore the cross-tissue capacity of this 'AS-Expression Network' concept, we curated tumor-infiltrating lymphocyte T cells from four cancer types: liver cancer (HCC) [31], colorectal cancer (CRC) [32], lung cancer (LUAD) [20], and recurrent liver cancer (RHCC) [33].

By selecting the top 1,223 ASP events from a total of 86,616 valid ASP-EXP links (supported by at least 2 samples across a minimum of 2 datasets), we identified 14 ASP clusters (referred to as C1–C14) (**Fig. S5A-C**). We subsequently generated 10 cell subpopulations (referred to as S0–S9) based on distinct compositions of ASP cluster-wise signatures across the 4 T cells datasets (**Fig. 3A and S5D**). Consistent with the clustering observed in tumor cells, AENet effectively mitigated the batch effect, whereas SCASL did not (**Fig. S5E**). Among these populations, S2 and S3 expressed naïve T cell markers (*IL7R, CCR7, LEF1*), S1 was characterized by memory T cell markers (*CD52, ANXA1, CREM*), S0 and S5 exhibited effector T markers (*NKG7, GZMA/B*), S4, S7, S8, and S9 represented the exhausted state of T cells (*PDCD1, CTLA4, HAVCR2*), while S6 corresponded to the proliferative state (*MKI67, TOP2A*) (**Fig. S5F**) [34]. Strikingly, AEnet did not partition T cells into canonical $CD4^+$ and $CD8^+$ subtypes, suggesting that alternative splicing primarily contributes to the transition of cell states rather than defining cell lineage (**Fig. 3B**).

When focusing on ASP clusters, the exclusive deficiency of C_5 in effector T cells (S0 and S5) and C_8 in proliferating T cells (S6) were two notable signatures demonstrating the correlation between specific ASPs and cell population heterogeneity (**Fig. 3C**). Among the top 10 common splicing factors, the C_5 ASP cluster positively correlated with EEF1A1 and PABPC1, which were partially aligned with ASPs primarily occurring in naïve T cells (clusters C_7, C_9, and C_13) (**Fig. 3D-E**). Additionally, C_5 was correlated with HSPA1A/B, splicing factors contributing to exhausted T cells (C_1, C_2, C_3, C_12, and C_14). Effector T cells tend to adopt splicing patterns opposite to those of the C_5 ASP cluster, which suggests C_5 ASP class emerged as a pivotal determinant for effector T cell formation (**Fig. 3C**). We then explored the enriched pathways associated with hub genes in C_5 to identify potential factors that distinguish effector T cells from other T cell types (**Fig. 3F**). Surprisingly, effector T cells favored a previously undefined FYB pattern (referred to as FYB_new), using chr5_39217636 as the end of the first exon of FYB1—a site not documented in the GRCh38.p14 reference genome. This splicing variant is associated with pathways involved in second messenger generation, immune

response-regulating signaling, and cell surface receptor signaling, all critical processes for achieving effector cell status (**Fig. 3G-H**). In contrast, FYB1-212 was associated with pathways linked to naïve and memory T cells. Regarding the other ASP cluster, C_8 was negatively correlated with SNRNP25 among the top 10 factors (**Fig. 3E**). Small Nuclear Ribonucleoprotein 25 (SNRNP25) is known for its role in spliceosome assembly and function, facilitating the accurate removal of introns from pre-mRNA and the joining of exons to form mature mRNA[35]. Its upregulation (reflected as a double negative correlation) in proliferating T cells served as a clear marker, identifying this cell type solely through splicing factors **(Fig. S5G)**.

Of particular interest, HNRNPLL, the third-ranked splicing factor (**Fig. 3D**), was highly positively correlated with exhausted T-associated ASP clusters and linked to poor prognosis (**Fig. 3E, 3I**). Genes regulated by HNRNPLL were enriched in thymic T cell selection, axon guidance, and leukocyte activation (**Fig. S5H**). Among the hub genes, CD3D, the most canonical T cell marker, exhibited two isoforms with distinct PSI distributions between exhausted and other T cells (**Fig. S5I**). Exhausted T cells preferentially spliced into isoform CD3D-202, which is linked to the PD-1 signaling pathway, CD28 family-mediated costimulation, and TCR signaling, while other T cells primarily utilized CD3D-201 for differentiation (**Fig. S5J**).

Collectively, we demonstrate the capability of the AEnet algorithm for integrated scRNA-seq data analysis without the need for batch corrections. Our algorithm facilitates bioinformatic data mining for isoform usage preferences, and even the discovery of novel isoforms of functional importance, such as new ASPs in the FYB gene for effector T cells.

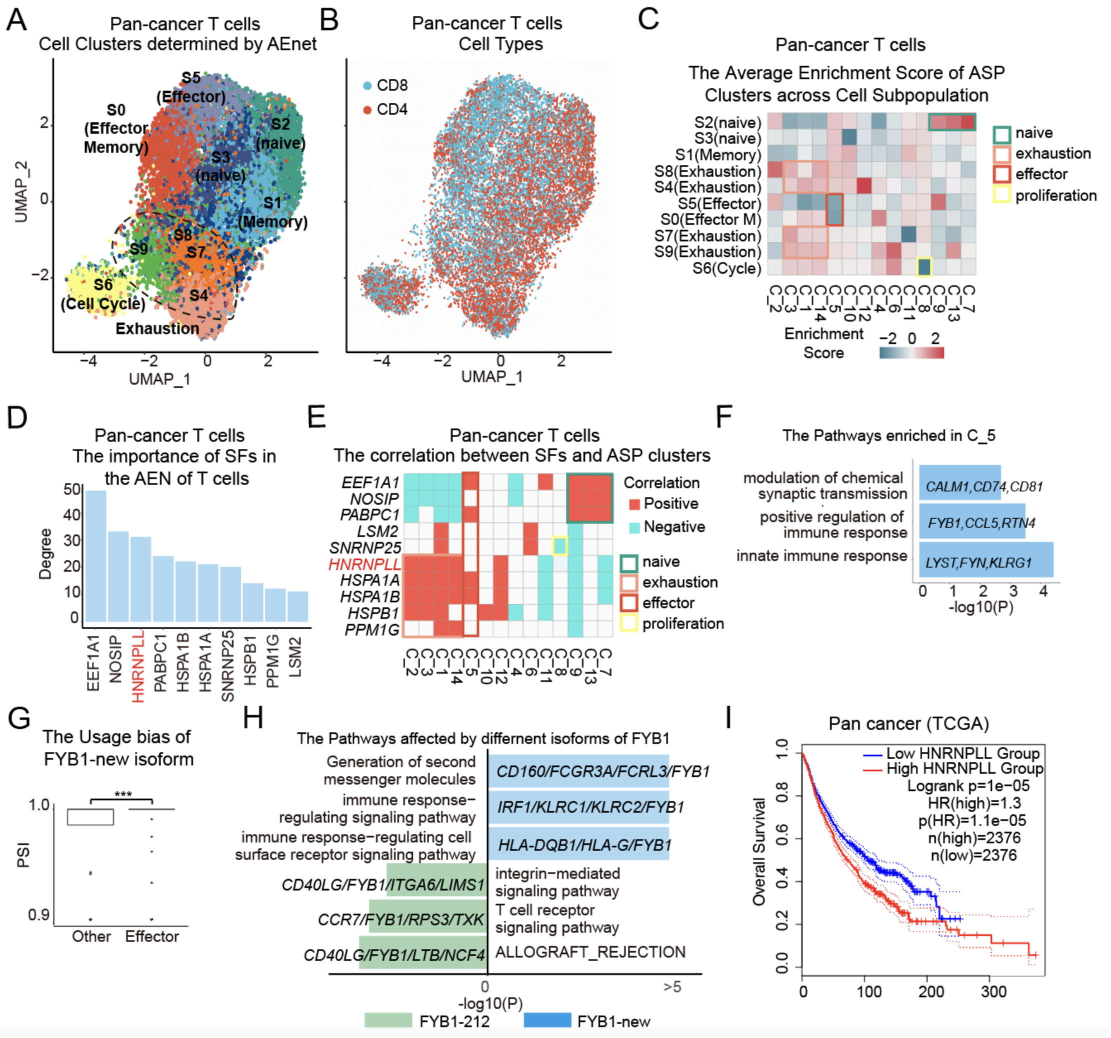

Figure 3. Application of the AEnet to Pan-Cancer Tumor-Infiltrating Lymphocyte T Cell Single-Cell Data. A-B. UMAP displays clustering of cell types determined by AEnet (A) and lineage (B). **C.** The heatmap displays the enrichment score of ASP clusters across cell clusters determined by AS. **D.** The barplot displays the importance of splicing factors in the formation of the AEN network of the pan-cancer T cells. **E.** The relationship between ASP clusters and key splicing factors. **F.** The top 3 pathways enriched for the genes with alternative splicing patterns in C_5 ASP clusters. **G.** The PSI distribution of FYB1-new isoform within FYB1 across effector and other T cells. Statistical analysis was performed using the Student's t-test. **H.** The pathways enriched in the gene sets with different isoforms of FYB1. **I.** Kaplan-Meier analysis shows the overall survival of patients characterized by low (blue) or high (red) HNRNPLL in the TCGA cohort. Statistical analysis was performed using the log-rank test. * $p < 0.05$, ** $p < 0.01$, *** $p < 0.001$.

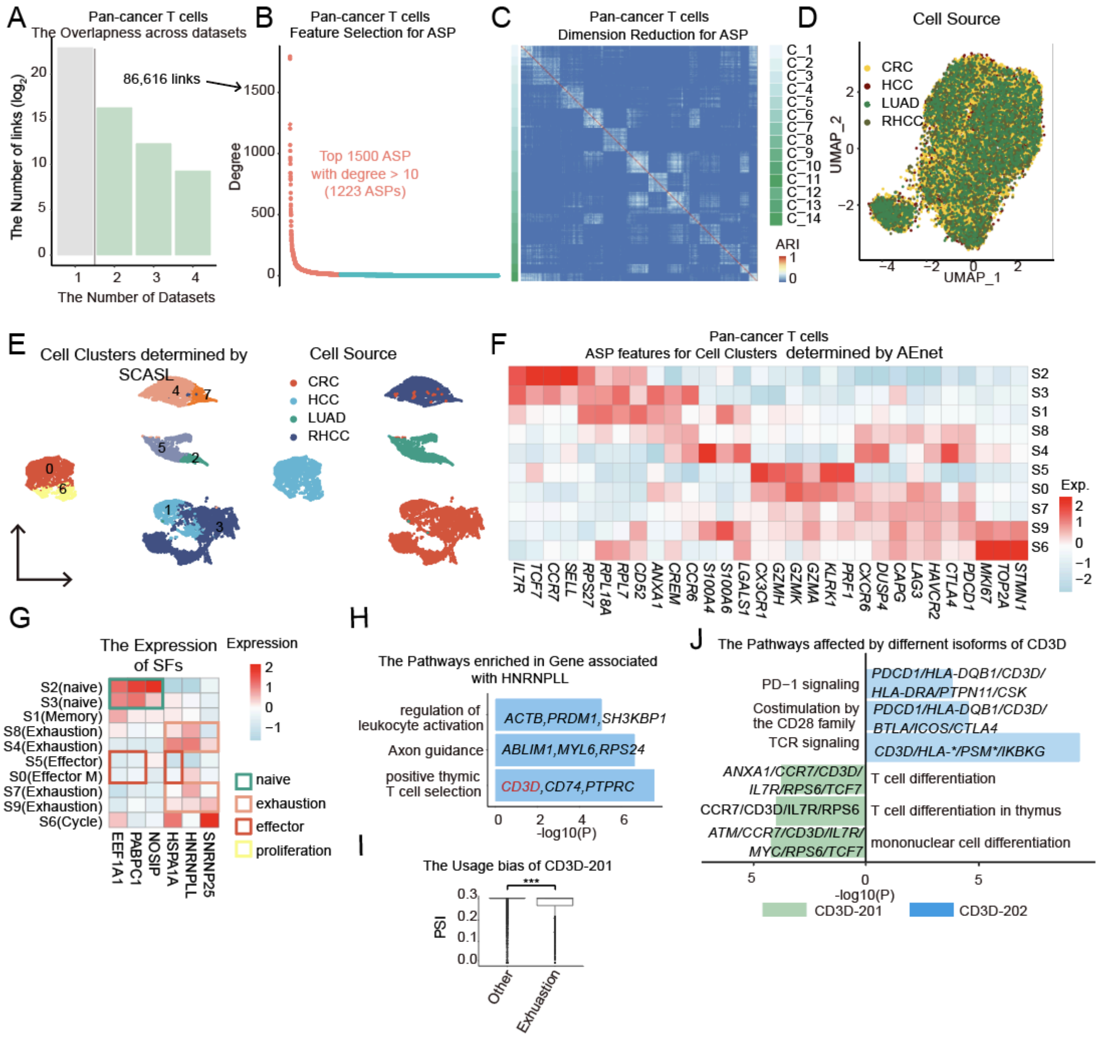

Figure S5. AEnet reveals cellular splicing heterogeneity and its key splicing events in tumor-infiltrating T cells across various cancer types. A. The barplot displays the selection of high quality links in the AEnet of Pan-cancer T cells. **B.** The selection of key alternative splicing patterns. **C.** The heatmap shows the ASP cluster from dimension reduction of AEnet. **D.** UMAP displays cell sources. **E.** UMAP displays clustering of cell types determined by Alternative splicing by SCASL (left panel) and cell sources (right panel). **F.** The heatmap displays the expression of marker genes across cell clusters determined by AS. **G.** The expression of key splicing factors across clusters determined by alternative splicing. **H.** The pathways enriched in genes with ASP associated with HNRNPLL. **I.** The PSI distribution of CD3D-201 isoform within CD3D within exhaustion and other T cells. Statistical analysis was performed using the Student's t-test. **J.** The pathways enriched in the gene sets with different isoforms of CD3D.

AEnet Uncovers Transitional Cell States and Key Splicing Factors in Embryonic Gastrulation

During embryogenesis, alternative splicing is a key mechanism that fine-tunes developmental pathways and controls cell fate decisions. Here, we apply AEnet to interrogate a scRNA-seq dataset of gastrulation-stage human embryos from the Human Developmental Biology Resource, elucidating how the AS process enables precise regulation of gene expression at this stage. The reference dataset comprises 1,195 cells (665 caudal, 340 rostral, and 190 yolk sac cells), with a median of 4,000 genes detected per cell[36].

Analogous to the previous analysis process, we first identified a total of 1,604 ASP events, 25 ASP clusters, and 11 cell populations (**Fig. 4A and S6A-C**). Notably, the ASPs are assumed to be highly distinct among different cell types during embryogenesis. Therefore AEnet factorized the cell populations in a manner similar to those clustered based solely on RNA profilings (**Fig. 4B**, ARI = 0.304). The sequential differentiation trajectory from epiblast cells (cell subpopulation 5, S5) to the primitive streak (S1), followed by the transition to endodermal cells (S6/S10) or mesoderm (S2/S0), ultimately leading to axial mesoderm (S7), was clearly discernible (**Fig. 4A, right panel**) [37].

Epiblast cells (S5) are classic pluripotent stem cells, derived from the inner cell mass of the blastocyst and capable of differentiating into the three germ layers. For the highly correlated ASP clusters C_13 and C_17, PSIP1 and SNRPN were identified as key regulatory factors that maintain cell stemness (**Fig. 4C-D, S6D-E**). HNRNPAB and SRSF3 were associated with splicing decisions that resulted in longer junctions spanning genomic loci, observed more frequently in epiblast cells than in cells with reduced stemness (**Fig. 4E**). We verified this intriguing finding in an independent iPSC dataset (**Fig. S6F**)[13].

Some populations defined by AEnet were in a transitional stage. For example, S2 shares RNA profiling similarities with both S1 and S0 (**Fig. 4B**). This cell population expressed relatively lower stemness signatures and higher mesodermal features compared to the primitive streak (S1) (**Fig. 4F**). The enriched pathways also indicated that S2 was an intermediate cell state between the primitive streak (S1) and mesoderm (S0) (**Fig. 4G**). We subsequently compared the ASP cluster compositions between S2 and S0, focusing on two of the most distinct ASP clusters, C_1 and C_18, for downstream analysis (**Fig. 4C**). As part of the routine analysis, we identified the top splicing factors and hub-gene-enriched pathways. SNRPD2, a core component of the spliceosome, emerged as a pivotal factor distinguishing these two mesoderm subtypes (**Fig. 4H and S6G**). Related to the genes in enriched pathways, TNRC6B displayed distinct isoform distributions across cell clusters (**Fig. 4I and S6H**). TNRC6B-205 was enriched in S2 and linked to the classical WNT, Notch, and

MAPK signaling pathways. In contrast, TNRC6B-201 was prevalent in other cell types and associated with primary germ layer formation and other processes (**Fig. 4J**).

Moreover, we unveiled that ASP clusters C_2, C_5, C_7, C_16, and C_23 were pivotal for endoderm differentiation, with HSPB1 emerging as a core negative regulator of their formation. Clusters C_12, C_20, and C_24, crucial for yolk sac mesoderm development, were positively regulated by HSPB1 **(Fig. S6I-J)**. Lastly, ASP events in HEP and erythrocyte development (C_3, C_4, C_6, C_8, C_11, C_14, C_22, and C_25) were governed by MBNL1 and HSPA5, both highly expressed in these cell types (**Fig. S6I-J**).

By exploring the alternative splicing landscapes in this human embryonic data (**Fig. 4K**), we not only clarified phenotypic subtleties along the AS-based developmental trajectory but also illuminated the complexity of AS mechanisms underlying cell differentiation and embryonic development, successfully demonstrating the capabilities of the AEnet algorithm.

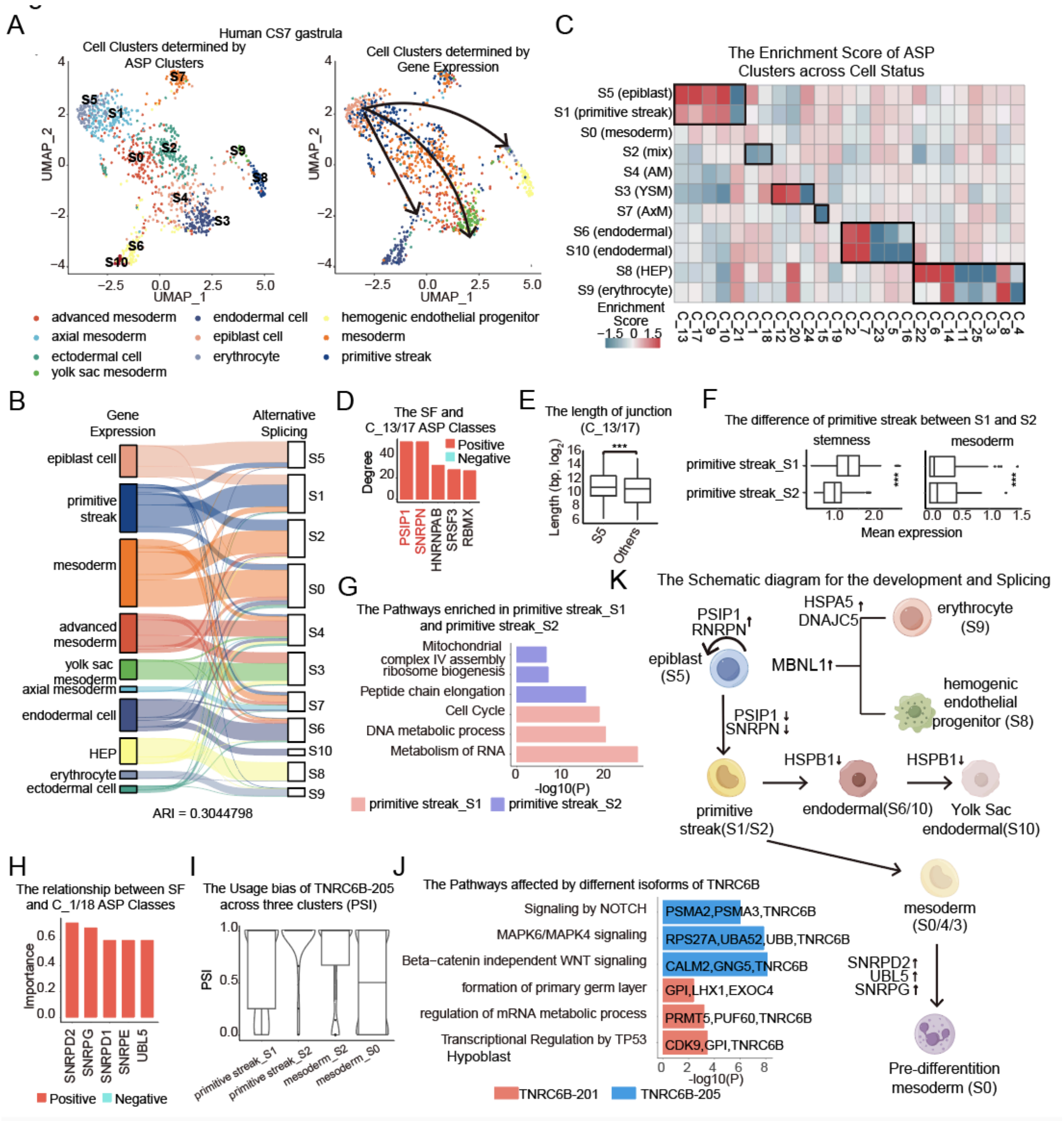

Figure 4. AEnet Uncovers Transitional Cell States and Key Splicing Factors in Embryonic Gastrulation. A. UMAP displays clustering of cells by cell type determined by Alternative Splicing (left panel), and gene expression (right panel). **B.** The overlapping of cells between clusters determined by alternative splicing and gene expression. **C.** The heatmap displays the enrichment score of ASP classes across cell clusters determined by AS. **D.** The barplot displays the importance of splicing factors in the formation of C_13/17 ASP classes. The color indicates the relationship between SF and ASP classes. **E.** The length of junction that differentially usage in different cell types. Statistical analysis was performed using the Student's t-test. **F.** The boxplot displays the expression of stemness and mesoderm markers across primitive streak cell subsets determined by AS and expression. Statistical analysis was performed using the Student's t-test. **G.** The pathways enriched in the upregulated genes in the primitive streak cell subsets determined by AS and expression. **H.** The barplot displays the importance of splicing factors in the formation

of C_1/18 ASP classes. The color indicates the relationship between SF and ASP classes. **I.** The alternative splicing patterns of TNRC6B and the PSI distribution. **J.** The pathways enriched in the gene sets with different isoforms of TNRC6B. **K.** The illustration depicts the developmental trajectory and highlights the top-ranked key splicing factors based on their relative importance. * $p < 0.05$, ** $p < 0.01$, *** $p < 0.001$.

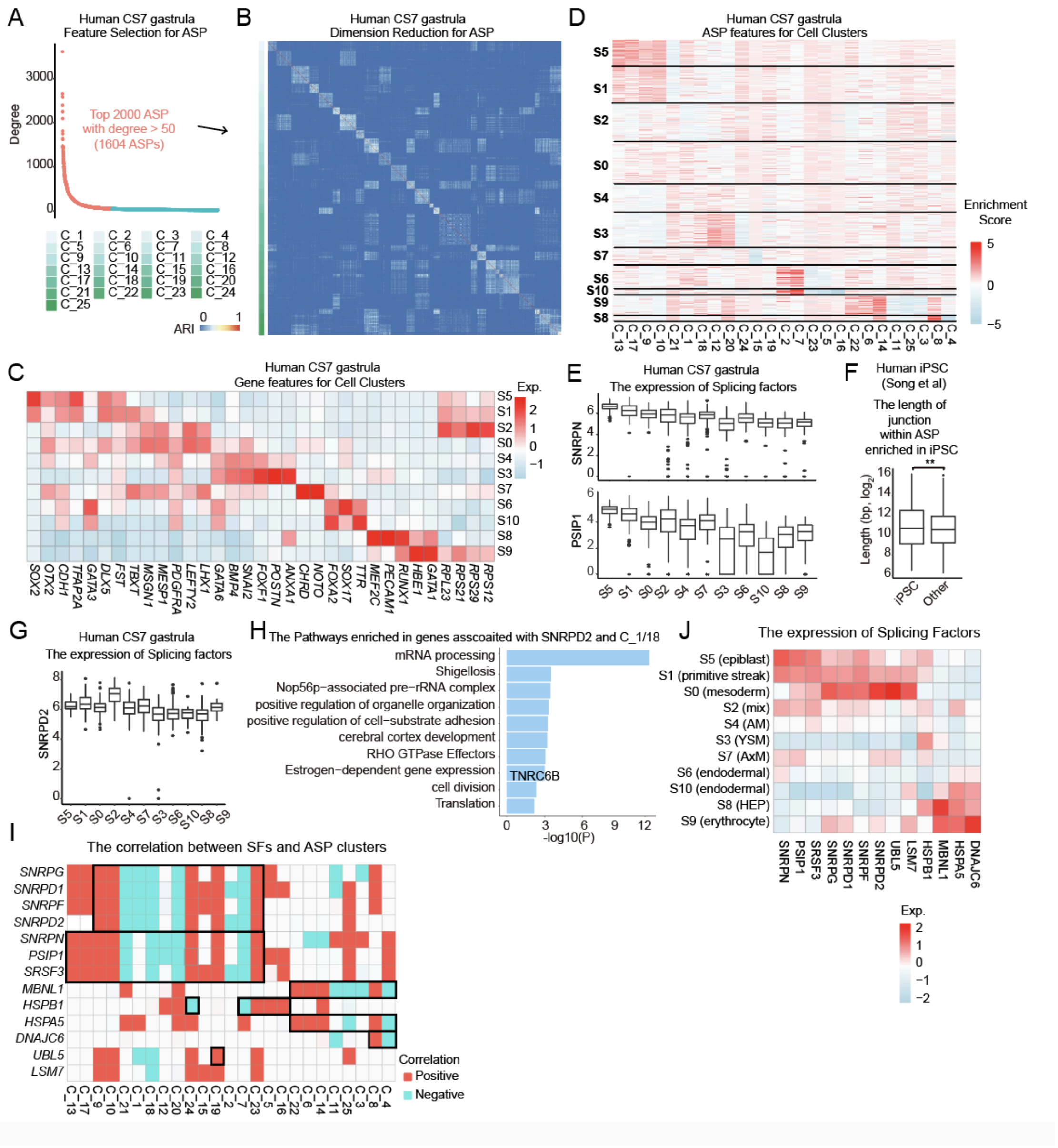

Figure S6. AEnet reveals intermediate cell status during gastrulation. A. The selection of key alternative splicing patterns. **B.** The heatmap shows the ASP classes from dimension reduction of AEnet. **C.** The heatmap displays the expression of developmental markers across cell clusters determined by AS. **D.** The heatmap displays the enrichment score of ASP classes across cell clusters determined by AS. **E.** The expression of SNRPN and PSIP1 across the cell clusters determined by AS. **F.** The length of junction that differentially usage in different cell types from Song et

al. Statistical analysis was performed using the Student's t-test. **G.** The expression of SNRPD2 across the cell clusters determined by AS. **H.** The pathways enriched in the genes within C_1 and C_18, which shows correlation with the expression of SNRPD2. **I-J.** The heatmap displays the relationship between ASP classes and key splicing factors (I), as well as the expression of key splicing factor **(J)**. * $p < 0.05$, ** $p < 0.01$, *** $p < 0.001$.

Discussion

The discovery of cellular heterogeneity can be enhanced by multiple modalities derived from single-cell genomics technologies. For example, SHARE-seq provides insights into cell identity by jointly detecting gene expression and chromatin regulation [38], while CITE-seq simultaneously studies the transcriptome and protein expression, accounting for post-transcriptional and translational modifications to enable fine-grained detection of cell populations [39]. However, generating such data remains laboratory-intensive, involving complex, multi-step workflows that require careful optimization and the use of specialized reagents and equipment. In fact, alternative splicing events represent another inherent modality that is often present in common scRNA-seq data [40,41]. These events can be cost-efficiently detected using plate-based platforms like Fluidigm C1 and Smart-seq [42,43], or droplet-based platforms with higher sequencing depth [17,44,45].

The introduction of AEnet represents a groundbreaking attempt to integrate single-cell alternative splicing dynamics and gene expression profiling into a network to explore cellular heterogeneity, accounting for post-transcriptional regulatory mechanisms. By applying AEnet to three distinct single-cell datasets, we found that the interplay between AS and gene expression is dynamic when generating cell populations. For tumor patient samples with varying immunotherapy responses and pan-cancer T cell analysis, the integrated clustering results reveal distinct cell states within the RNA-based subtypes. In contrast, during cell differentiation in embryonic gastrulation, the clustering results more closely resemble the major cell types. This suggests that AS events contribute differently across tissues and conditions in distinguishing cell clusters, with a particularly important role during the fine-grained clustering process. Therefore, previous methods that first rely on RNA-based clustering and then apply AS-based differential analysis as a separate downstream step are not optimal for capturing cellular heterogeneity using AS events.

One of the core advantages of AEnet lies in its ability to "modularize" complex gene expression data by grouping AS patterns, thereby minimizing noise from RNA expression profiling, which is often referred to as batch effects in scRNA-seq analysis. This modular approach also mitigates AS-based biases related to missing values, tumor heterogeneity, and patient-specific factors, while enabling the

identification of novel cell subpopulations and intermediate states that may remain obscured using traditional methods.

With the progression of sequencing technologies, particularly the advent of long-read sequencing, alternative splicing events can be identified more conveniently and accurately [46,47]. This advancement will significantly broaden the use of our method as a conventional analysis tool. However, even with well-designed sequencing, barcode errors in current single-cell long-read sequencing still significantly affect data quality, leading to the misassignment of AS events to unrelated cells [48]. We look forward to the rapid development of high-precision, high-throughput long-read sequencing technologies to advance research in the single-cell AS field and the development of our software in the near future.

Methods

Dataset preprocessing prior to AEnet for alternative splicing

Dataset preprocessing prior to AEN involved a series of essential steps aimed at constructing the cell-junction count matrix and the annotation file for junctions, by the established DESJ-detection pipeline [15]. Initially, alignment software such as STAR was employed to obtain the coverage information of junctions in each cell [49]. To ensure the reliability of the data, the junctions with a minimum of Rm reads in Cellm cells were retained, with default values set as Cellm = 10 and Rm = 4. Additionally, all junctions were annotated to determine their primary gene sources. Specifically, we selectively retained junctions that were exclusively associated with a single gene to ensure accuracy of the alignment. Furthermore, a count matrix was generated, reflecting the read numbers of junctions in each cell. Subsequently, the count matrix was normalized through dividing the counts by the unique mapped reads number for each cell, resulting in the generation of the CPM (counts per million) matrix. The diligent execution of these preprocessing steps ensured the integrity and reliability of the dataset, allowing for robust analysis within the AEN framework.

Detailed procedure for AEnet

The AEnet method consists of multiple main steps: ASP identification and quantification, AEN network and multiple samples integration, anchor ASP identification, ASP clusters identification, cell clustering, and regulatory mechanism prediction. In this section, we will introduce each step in detail.

ASP Identification and Quantitative

Exon-exon junctions with the same starting point or endpoint were defined as alternative splicing patterns (ASP). The percent spliced In (PSI) value of the ASPs in cells was measured by comparing the coverage of one junction to the two junctions derived from the ASP. Subsequently, we integrated all cells and all ASPs into a cell-ASP PSI matrix.

AEN Network and multiple samples integration

We integrated the alternative splicing event scoring matrix and gene expression matrix of all cells in each sample, to infer the potential relationship between ASPs and gene expression. For each sample, AEnet calculated the significance of the correlation between ASP PSI values and gene expression using Spearman's rank correlation. Significant ASP PSI - Gene Expression links ($p < 0.01$) were then used to construct an alternative splicing-gene expression association network (AEN) for each sample. The edge width of the links in the network was determined using the

Spearman correlation coefficient, which indicates both the strength and the direction (positive or negative) of the correlation. To ensure robustness, we extracted ASP-EXP links that exhibited consistent trends across multiple samples (defined as more than two samples). These links were then integrated into a multi-sample AEN network. In this pan-sample AEN network, the width of the edges corresponds to the number of samples supporting each ASP-EXP link, while the color indicates the direction of the correlation (positive or negative). This approach effectively mitigated batch effects and strengthened the reliability of the analysis.

Anchor ASPs identification

We calculated the degree strength of the ASP in the AEN network and selected the top ASPs based on their ranking. The degree of ASPs was determined by the number of links to the ASP in the AEN network. A higher degree indicates a stronger association between the ASP and gene expression dynamics in the dataset. The top 1500 ASPs (default) were chosen for the downstream analysis. To reduce data complexity and extract key information, we calculate the Jaccard metric of ASP-EXP links (where genes linked to the ASP are treated as sets) to represent their similarity for each pair of ASPs, thereby constructing a similarity matrix of anchor ASPs.

ASP clusters identification

Using hierarchical clustering methods, we divided the ASPs into ASP clusters according to their similarity, ensuring that ASPs in the same clusters were associated with similar gene expression sets. The number of clusters is defaultly set as 25. Clusters are filtered by ensuring that at least 10% of ASP pairs within each cluster exhibit a similarity score higher than 0.1. Clusters with fewer than 10 ASPs are merged with the most similar clusters, provided that the similarity between clusters is above 0.1. The similarity between two ASP clusters is defined as the proportion of ASP pairs with a similarity score greater than 0.1, where one ASP in the pair belongs to one cluster and the other ASP belongs to the other cluster.

Cell Clustering Analysis

To further understand the cell heterogeneity at the level of alternative splicing, we performed cell clustering analysis based on alternative splicing. First, we calculated the enrichment score of each ASP cluster in individual cells, constructing a cell-ASP cluster enrichment score matrix. The enrichment score of an ASP cluster in a given cell was defined as the average PSI value of all ASPs within the cluster for that cell. Next, the cell-ASP cluster enrichment score matrix was normalized using the scale function. Finally, the normalized matrix was used as input for the FindNeighbors and FindClusters functions of Seurat to detect cell clusters [50,51].

Regulatory Mechanism Inference

To identify key splicing factors, we integrated the AEN network with a predefined list of splicing factors and a specific set of ASP events [52]. This allowed us to construct a sub-network of ASP-splicing factors, facilitating the exploration of the regulatory relationships between alternative splicing events and splicing factors. The higher the degree strength of the splicing factor, the more it indicated a close relationship with the occurrence of these ASP events. The regulatory direction of splicing factors on the given set of ASPs was determined based on the proportion of positive or negative correlations. Specifically, if 75% or more of the splicing factor–ASP pairs were positive (or negative), the splicing factor was classified as positively (or negatively) regulating the ASP sets.

We also identified key pathways associated with different ASP patterns. For each ASP event, gene sets that were positively and negatively correlated with the event were identified based on the AEN network. Functional enrichment analysis was then performed on both the ASP' gene and the positively (or negatively) correlated gene sets. The pathway to which the ASP gene belongs is considered the most likely pathway affected by the ASP change.

Performance evaluation

To assess the capability of AENet in identifying ASP clusters, a simulated ASP similarity matrix was generated, with ASPs as both the columns and rows, and the similarity (Jaccard index) as the matrix elements. Increasing levels of noise were introduced using the 'noiseInjector.unif' function in GROAN package. The number of ASP clusters was set to 3. The consistency score of AENet performance was defined as the Jaccard index between the clusters identified by AENet and the background clusters. For the performance comparison between AENet and SCASL, we extracted data from three patients in the lung cancer dataset and four patients from the CRC and RHCC cohorts.

The lung cancer cells data processing

We downloaded the scRNA-seq raw reads of the human lung cancer dataset from the NCBI database under accession code PRJNA591860[20]. This dataset contained 1,286 normal and cancer cells from six patients before initiating systemic targeted therapy (TKI naive [TN]), at the residual disease (RD) state, which includes samples taken at any time during treatment with targeted therapy while the tumor was regressing or stable by clinical imaging (RD), and upon subsequent progressive disease as determined by clinical imaging, at which point the tumors showed acquired drug resistance (progression [PD]). The human genome (version GRCH38) was used as the reference genome for alignment with STAR (v2.5.3)[49]. We used an

existing pipeline to create the junction count matrix. We first merged all the output of the SJ.out.tab files from the STAR aligner. Next, we conducted the dataset pre-processing prior to the AEnet step, described as before. Finally, We get the cell-junction CPM matrix, and the junction annotation files for the dataset.

Then, we used the outcome from the above step as the input to the AEnet pipeline. Firstly, the CPM matrix was input to the asp function of AEnet with default parameters, and the ASPs for each gene is identified. Secondly, the correlation network between ASPs and Gene is constructed using the asp function with default parameters based on the expression matrix and the junction CPM matrix. Next, we further refined the AEN network by retaining only the connection pairs with at least 4 samples supported, using the merge_cor function with default parameters. Additionally, the ASPs with positive or negative links all more than 20 are retained and 70 key ASPs were identified. These key ASPs were then clustered with hierarchy clustering, leading to the identification of 6 ASP clusters based on their similarity, using the junction_clustering function with default parameters. Subsequently, we calculated the enrichment score of each ASP cluster for the cells and performed cluster analysis using the enrichment score matrix of the cell-ASP clusters, categorizing the cells into 3 groups using asp_score and cell_clus function with resolution as 0.2. To further identify the key splicing factors involved in the formation of the C_4 ASP classes, we used the key_sf function to detect the key SF. Additionally, we also group cells into different populations based on gene expression. The gene count matrix was normalized using log1p normalization. Next, the top 3000 highly variable genes were selected to perform principal component analysis. Subsequently, 20 dimensions of principal components were used to perform Louvain clustering and Uniform Manifold Approximation and Projection (UMAP)-based visualization.

The SCASL package was applied to the junction count matrix of tumor cells to perform cell clustering, using default parameters for filtering, normalization, imputation, and clustering [53]. The Seurat [50,51] package was applied to the expression matrix of tumor cells to perform normalization, dimensionality reduction and clustering: (1) the data was normalized using the “LogNormalize” function; (2) the top 2000 highly variable genes were detected with the “FindVariableFeatures” function and selected, the batch effects across different samples were corrected by the “FindIntegrationAnchors” and “IntegrateData” functions; (3) a KNN-based graph in the 20 PCA space was constructed and refined by cell-cell weights using the “FindNeighbors” function; (4) representative results from graph-based clustering were obtained using the “FindClusters” function with a resolution of 0.3; (5) the top 20 PCAs were used to perform UMAP for visualization of the cells.

The pan-cancer T cells data data processing

We downloaded the scRNA-seq raw reads of human T cells in the fasta format from the EGD database (EGAS00001002072 for HCC, EGAS00001002791 for CRC), NCBI database (PRJNA591860 for LUAD), and CNSA database (CNP0000650 for RHCC). The corresponding gene expression matrix was downloaded from the GEO database (GSE98638 for HCC, GSE108989 for CRC) and CNSA database (CNP0000650 for RHCC). The human genome (version GRCH38) was used as the reference genome for alignment with STAR (v2.5.3). We used an existing pipeline to create the junction count matrix. We first merged all the output of the SJ.out.tab files from the STAR aligner. Next, we conducted the dataset pre-processing prior to the AEnet step, described as before. Finally, We get the cell-junction CPM matrix, and the junction annotation files for each dataset.

Then, we used the outcome from the above step as the input to the AEnet pipeline. Firstly, the CPM matrix was input to the asp function of AEnet with default parameters, and the ASPs for each gene is identified for each dataset. Secondly, the correlation network between ASPs and Gene is constructed using the asp function with default parameters based on the expression matrix and the junction CPM matrix for each dataset. Next, we further refined the AEN network by retaining only the connection pairs with at least 2 samples supported for each dataset, using the merge_cor function with default parameters. Additionally, The ASP-EXP links supported by at least 2 datasets were retained for the downstream analysis. As a result, 1,223 key ASPs were identified based on their ranking and absolute number of degrees (top 2,000 with a degree number greater than 10). These key ASPs were then clustered, leading to the identification of 14 ASP clusters based on the similarity of their associated phenotypes through junction_clustering function with default parameters. Subsequently, we calculated the enrichment score of each ASP cluster for the cells of the four datasets and performed cluster analysis using the enrichment score matrix of the cell-ASP clusters, categorizing the cells into 10 groups using cell_clus function with resolution as 0.9. To further identify the key splicing factors involved in the formation of the 14 ASP clusters, we used the key_sf function to detect the key SF for each ASP cluster. AEnet was also used to detect key gene sets affected by ASPs from the same gene. These gene sets were then used to identify enriched pathways associated with different alternative splicing patterns of the same gene, such as CD3D and FYB1. The SCASL package was applied to the junction count matrix of T cells in multiple datasets to perform cell clustering, using default parameters for filtering, normalization, imputation, and clustering [53].

The gastrulating human embryo cells data processing

We downloaded the scRNA-seq raw reads of the human embryo dataset from ArrayExpress under accession code E-MTAB-9388. The processed data also was

downloaded from http://www.human-gastrula.ne. This dataset contained 1195 cells of the human embryo at embryonic day 16 assigned into 10 clusters. The human genome (version GRCH38) was used as the reference genome for alignment with STAR (v2.5.3). We used an existing pipeline to create the junction count matrix. We first merged all the output of the SJ.out.tab files from the STAR aligner. Next, we conducted the dataset pre-processing prior to the AEnet step, described as before. Finally, We get the cell-junction CPM matrix, and the junction annotation files for the dataset.

Then, we used the outcome from the above step as the input to the AEnet pipeline. Firstly, the CPM matrix was input to the asp function of AEnet with default parameters, and the ASPs for each gene is identified. Secondly, the correlation network between ASPs and Gene is constructed using the asp function with default parameters based on the expression matrix and the junction CPM matrix. Next, we further refined the AEN network by retaining only the connection pairs with p-values less than 1e-4. As a result, 1,604 key ASPs were identified based on their ranking and absolute number of degrees (top 2,000 with a degree number greater than 50). These key ASPs were then clustered, leading to the identification of 25 ASP clusters based on the similarity of their associated phenotypes. Subsequently, we calculated the enrichment of each ASP class within the cells and performed cluster analysis using the enrichment score matrix of the cell-ASP classes, categorizing the cells into 11 groups using the cell_clus function with resolution as 0.1. To further identify the key splicing factors involved in the formation of the 25 ASP classes, we used the key_sf function to detect the key SF for each ASP class. AEnet was also used to detect key gene sets affected by ASPs from the same gene. These gene sets were then used to identify enriched pathways associated with different alternative splicing patterns of the same gene, such as TNRC6B.

Pathway enrichment analysis

Metascape (http://metascape.org) was used to characterize the biological functions of the DEGs of cells in different status[54]. The differentially expressed genes between different cell types were uploaded into the Metascape for the pathway analysis with default setting.

Survival Analysis

GEPIA2 (http://gepia2.cancer-pku.cn/#survival) was used to detect the survival status of HNRNPLL[55]. HNRNPLL was uploaded into the GEPIA2 and the reference datasets were the whole TCGA datasets.

Acknowledgements

We sincerely thank the support provided by China National GeneBank. This study was supported by GuangDong Basic and Applied Basic Research Foundation (2021A1515110832), Shenzhen Key Laboratory of Single-Cell Omics (ZDSYS20190902093613831), Shenzhen Science and Technology Program (LCYX20220620105200001), the National Key R&D Program of China (2021YFC2501900), and Shenzhen Science and Technology Program (JCYJ20240813150001003).

Data availability

The gastrulating human embryo dataset is available in the ArrayExpress database under accession number E-MTAB-938824. scRNA-seq raw reads of human T cells in fastq format from the EGD database (EGAS00001002072 for HCC, EGAS00001002791 for CRC), NCBI database (PRJNA591860 for LUAD), and CNSA databse (CNP0000650 for RHCC). The corresponding gene expression matrix was downloaded from the GEO database (GSE98638 for HCC, GSE108989 for CRC) and CNSA database (CNP0000650 for RHCC).

Code availability

AEnet is written in R. The source code is available on Github at https://github.com/liushang17/AEN.

Author contributions

S.L., X.C, Y.B., L.W. and S.L. conceived the study, designed and performed research, contributed new analytical tools, analyzed data and wrote the manuscript. S.L. also developed the software, performed experiments and developed the metrics. X.C, X.H., Y.B., Y.W., W.H., P.Q., R.L., W.P., and X.Z. discussed the results and contributed to the writing. X.C, helped procure and interpret the datasets. L.W., Y.B., X.C, and S.L. supervised research and contributed to the writing. L.W. supervised the research and the entire project.

Competing interests

The authors declare no competing interests.

Declaration of generative AI and AI-assisted technologies in the writing process

During the preparation of this work, the authors used ChatGPT 4.0 (GPT-4) in order to facilitate the process of proofreading the contents of the draft[56]. After using this tool/service, the authors reviewed and edited the content as needed and took full responsibility for the content of the publication.

Reference

1. Liu, Y., Gonzàlez-Porta, M., Santos, S., Brazma, A., Marioni, J.C., Aebersold, R., Venkitaraman, A.R., and Wickramasinghe, V.O. (2017). Impact of alternative splicing on the human proteome. Cell Rep. *20*, 1229–1241.

2. Öther-Gee Pohl, S., and Myant, K.B. (2022). Alternative RNA splicing in tumour heterogeneity, plasticity and therapy. Dis. Model. Mech. *15*. https://doi.org/10.1242/dmm.049233.

3. Raj, B., and Blencowe, B.J. (2015). Alternative splicing in the mammalian nervous system: Recent insights into mechanisms and functional roles. Neuron *87*, 14–27.

4. Bhadra, M., Howell, P., Dutta, S., Heintz, C., and Mair, W.B. (2020). Alternative splicing in aging and longevity. Hum. Genet. *139*, 357–369.

5. Martinez, N.M., and Lynch, K.W. (2013). Control of alternative splicing in immune responses: many regulators, many predictions, much still to learn. Immunol. Rev. *253*, 216–236.

6. Martín, E., Vivori, C., Rogalska, M., Herrero-Vicente, J., and Valcárcel, J. (2021). Alternative splicing regulation of cell-cycle genes by SPF45/SR140/CHERP complex controls cell proliferation. RNA *27*, 1557–1576.

7. Braeutigam, C., Rago, L., Rolke, A., Waldmeier, L., Christofori, G., and Winter, J. (2014). The RNA-binding protein Rbfox2: an essential regulator of EMT-driven alternative splicing and a mediator of cellular invasion. Oncogene *33*, 1082–1092.

8. Jbara, A., Lin, K.-T., Stossel, C., Siegfried, Z., Shqerat, H., Amar-Schwartz, A., Elyada, E., Mogilevsky, M., Raitses-Gurevich, M., Johnson, J.L., et al. (2023). RBFOX2 modulates a metastatic signature of alternative splicing in pancreatic cancer. Nature *617*, 147–153.

9. Olivieri, J.E., Dehghannasiri, R., and Salzman, J. (2022). The SpliZ generalizes “percent spliced in” to reveal regulated splicing at single-cell resolution. Nat. Methods *19*, 307–310.

10. Li, Z., Zhang, B., Chan, J.J., Tabatabaeian, H., Tong, Q.Y., Chew, X.H., Fan, X., Driguez, P., Chan, C., Cheong, F., et al. (2024). An isoform-resolution transcriptomic atlas of colorectal cancer from long-read single-cell sequencing.

Cell Genom. *4*, 100641.

11. Shalek, A.K., Satija, R., Adiconis, X., Gertner, R.S., Gaublomme, J.T., Raychowdhury, R., Schwartz, S., Yosef, N., Malboeuf, C., Lu, D., et al. (2013). Single-cell transcriptomics reveals bimodality in expression and splicing in immune cells. Nature *498*, 236–240.

12. Huang, Y., and Sanguinetti, G. (2017). BRIE: transcriptome-wide splicing quantification in single cells. Genome Biol. *18*. https://doi.org/10.1186/s13059-017-1248-5.

13. Song, Y., Botvinnik, O.B., Lovci, M.T., Kakaradov, B., Liu, P., Xu, J.L., and Yeo, G.W. (2017). Single-cell alternative splicing analysis with expedition reveals splicing dynamics during neuron differentiation. Mol. Cell *67*, 148–161.e5.

14. Wen, W.X., Mead, A.J., and Thongjuea, S. (2023). MARVEL: an integrated alternative splicing analysis platform for single-cell RNA sequencing data. Nucleic Acids Res. *51*, e29.

15. Liu, S., Zhou, B., Wu, L., Sun, Y., Chen, J., and Liu, S. (2021). Single-cell differential splicing analysis reveals high heterogeneity of liver tumor-infiltrating T cells. Sci. Rep. *11*, 5325.

16. Zhang, Q., Ai, Y., and Abdel-Wahab, O. (2024). Molecular impact of mutations in RNA splicing factors in cancer. Mol. Cell *84*, 3667–3680.

17. Capitanchik, C., Wilkins, O.G., Wagner, N., Gagneur, J., and Ule, J. (2024). From computational models of the splicing code to regulatory mechanisms and therapeutic implications. Nat. Rev. Genet. https://doi.org/10.1038/s41576-024-00774-2.

18. Dvinge, H., Kim, E., Abdel-Wahab, O., and Bradley, R.K. (2016). RNA splicing factors as oncoproteins and tumour suppressors. Nat. Rev. Cancer *16*, 413–430.

19. Bradley, R.K., and Anczuków, O. (2023). RNA splicing dysregulation and the hallmarks of cancer. Nat. Rev. Cancer *23*, 135–155.

20. Maynard, A., McCoach, C.E., Rotow, J.K., Harris, L., Haderk, F., Kerr, D.L., Yu, E.A., Schenk, E.L., Tan, W., Zee, A., et al. (2020). Therapy-induced evolution of human lung cancer revealed by single-cell RNA sequencing. Cell *182*, 1232–1251.e22.

21. Travaglini, K.J., Nabhan, A.N., Penland, L., Sinha, R., Gillich, A., Sit, R.V., Chang, S., Conley, S.D., Mori, Y., Seita, J., et al. (2020). A molecular cell atlas of the human lung from single-cell RNA sequencing. Nature *587*, 619–625.

22. Sikkema, L., Ramírez-Suástegui, C., Strobl, D.C., Gillett, T.E., Zappia, L., Madissoon, E., Markov, N.S., Zaragosi, L.-E., Ji, Y., Ansari, M., et al. (2023). An integrated cell atlas of the lung in health and disease. Nat. Med. *29*, 1563–1577.

23. Yang, J., Antin, P., Berx, G., Blanpain, C., Brabletz, T., Bronner, M., Campbell, K., Cano, A., Casanova, J., Christofori, G., et al. (2020). Guidelines and definitions for research on epithelial-mesenchymal transition. Nat. Rev. Mol. Cell Biol. *21*, 341–352.

24. Beyer, E.C., and Berthoud, V.M. (2018). Gap junction gene and protein families: Connexins, innexins, and pannexins. Biochim. Biophys. Acta Biomembr. *1860*, 5–8.

25. Narayanaswamy, P.B., Baral, T.K., Haller, H., Dumler, I., Acharya, K., and Kiyan, Y. (2017). Transcriptomic pathway analysis of urokinase receptor silenced breast cancer cells: a microarray study. Oncotarget *8*, 101572–101590.

26. Piqué, L., Martinez de Paz, A., Piñeyro, D., Martínez-Cardús, A., Castro de Moura, M., Llinàs-Arias, P., Setien, F., Gomez-Miragaya, J., Gonzalez-Suarez, E., Sigurdsson, S., et al. (2019). Epigenetic inactivation of the splicing RNA-binding protein CELF2 in human breast cancer. Oncogene *38*, 7106–7112.

27. Lai, S., Wang, Y., Li, T., Dong, Y., Lin, Y., Wang, L., Weng, S., Zhang, X., and Lin, C. (2022). N6-methyladenosine-mediated CELF2 regulates CD44 alternative splicing affecting tumorigenesis via ERAD pathway in pancreatic cancer. Cell Biosci. *12*, 125.

28. Qin, P., Chen, H., Wang, Y., Huang, L., Huang, K., Xiao, G., Han, C., Hu, J., Lin, D., Wan, X., et al. (2023). Cancer-associated fibroblasts undergoing neoadjuvant chemotherapy suppress rectal cancer revealed by single-cell and spatial transcriptomics. Cell Rep. Med. *4*, 101231.

29. Fukuda, Y., Bustos, M.A., Cho, S.-N., Roszik, J., Ryu, S., Lopez, V.M., Burks, J.K., Lee, J.E., Grimm, E.A., Hoon, D.S.B., et al. (2022). Interplay between soluble CD74 and macrophage-migration inhibitory factor drives tumor growth and influences patient survival in melanoma. Cell Death Dis. *13*. https://doi.org/10.1038/s41419-022-04552-y.

30. Leng, L., Metz, C.N., Fang, Y., Xu, J., Donnelly, S., Baugh, J., Delohery, T., Chen, Y., Mitchell, R.A., and Bucala, R. (2003). MIF signal transduction initiated by binding to CD74. J. Exp. Med. *197*, 1467–1476.

31. Zheng, C., Zheng, L., Yoo, J.-K., Guo, H., Zhang, Y., Guo, X., Kang, B., Hu, R., Huang, J.Y., Zhang, Q., et al. (2017). Landscape of infiltrating T cells in liver cancer revealed by single-cell sequencing. Cell *169*, 1342–1356.e16.

32. Zhang, L., Yu, X., Zheng, L., Zhang, Y., Li, Y., Fang, Q., Gao, R., Kang, B., Zhang, Q., Huang, J.Y., et al. (2018). Lineage tracking reveals dynamic relationships of T cells in colorectal cancer. Nature *564*, 268–272.

33. Sun, Y., Wu, L., Zhong, Y., Zhou, K., Hou, Y., Wang, Z., Zhang, Z., Xie, J., Wang, C., Chen, D., et al. (2021). Single-cell landscape of the ecosystem in early-relapse hepatocellular carcinoma. Cell *184*, 404–421.e16.

34. Zheng, L., Qin, S., Si, W., Wang, A., Xing, B., Gao, R., Ren, X., Wang, L., Wu, X., Zhang, J., et al. (2021). Pan-cancer single-cell landscape of tumor-infiltrating T cells. Science *374*, abe6474.

35. Wilkinson, M.E., Charenton, C., and Nagai, K. (2020). RNA splicing by the spliceosome. Annu. Rev. Biochem. *89*, 359–388.

36. Tyser, R.C.V., Mahammadov, E., Nakanoh, S., Vallier, L., Scialdone, A., and Srinivas, S. (2021). Single-cell transcriptomic characterization of a gastrulating human embryo. Nature *600*, 285–289.

37. Zhai, J., Xiao, Z., Wang, Y., and Wang, H. (2022). Human embryonic development: from peri-implantation to gastrulation. Trends Cell Biol. *32*, 18–29.

38. Ma, S., Zhang, B., LaFave, L.M., Earl, A.S., Chiang, Z., Hu, Y., Ding, J., Brack, A., Kartha, V.K., Tay, T., et al. (2020). Chromatin potential identified by shared single-cell profiling of RNA and chromatin. Cell *183*, 1103–1116.e20.

39. Stoeckius, M., Hafemeister, C., Stephenson, W., Houck-Loomis, B., Chattopadhyay, P.K., Swerdlow, H., Satija, R., and Smibert, P. (2017). Simultaneous epitope and transcriptome measurement in single cells. Nat. Methods *14*, 865–868.

40. Kahles, A., Lehmann, K.-V., Toussaint, N.C., Hüser, M., Stark, S.G., Sachsenberg, T., Stegle, O., Kohlbacher, O., Sander, C., Cancer Genome Atlas Research Network, et al. (2018). Comprehensive analysis of alternative splicing across tumors from 8,705 patients. Cancer Cell *34*, 211–224.e6.

41. Cortés-López, M., Chamely, P., Hawkins, A.G., Stanley, R.F., Swett, A.D., Ganesan, S., Mouhieddine, T.H., Dai, X., Kluegel, L., Chen, C., et al. (2023). Single-cell multi-omics defines the cell-type-specific impact of splicing aberrations in human hematopoietic clonal outgrowths. Cell Stem Cell *30*, 1262–1281.e8.

42. Picelli, S., Björklund, Å.K., Faridani, O.R., Sagasser, S., Winberg, G., and Sandberg, R. (2013). Smart-seq2 for sensitive full-length transcriptome profiling in single cells. Nat. Methods *10*, 1096–1098.

43. Picelli, S., Faridani, O.R., Björklund, A.K., Winberg, G., Sagasser, S., and Sandberg, R. (2014). Full-length RNA-seq from single cells using Smart-seq2. Nat. Protoc. *9*, 171–181.

44. Liao, Y., Liu, Z., Zhang, Y., Lu, P., Wen, L., and Tang, F. (2023). High-throughput and high-sensitivity full-length single-cell RNA-seq analysis on third-generation sequencing platform. Cell Discov. *9*, 5.

45. Shiau, C.-K., Lu, L., Kieser, R., Fukumura, K., Pan, T., Lin, H.-Y., Yang, J., Tong, E.L., Lee, G., Yan, Y., et al. (2023). High throughput single cell long-read sequencing analyses of same-cell genotypes and phenotypes in human tumors. Nat. Commun. *14*, 4124.

46. Joglekar, A., Hu, W., Zhang, B., Narykov, O., Diekhans, M., Marrocco, J., Balacco, J., Ndhlovu, L.C., Milner, T.A., Fedrigo, O., et al. (2024). Single-cell long-read sequencing-based mapping reveals specialized splicing patterns in developing and adult mouse and human brain. Nat. Neurosci. *27*, 1051–1063.

47. Kumari, P., Kaur, M., Dindhoria, K., Ashford, B., Amarasinghe, S.L., and Thind, A.S. (2024). Advances in long-read single-cell transcriptomics. Hum. Genet. *143*, 1005–1020.

48. Weile, J., Ferra, G., Boyle, G., Pendyala, S., Amorosi, C., Yeh, C.-L., Cote, A.G., Kishore, N., Tabet, D., van Loggerenberg, W., et al. (2024). Pacybara: accurate long-read sequencing for barcoded mutagenized allelic libraries. Bioinformatics *40*. https://doi.org/10.1093/bioinformatics/btae182.

49. Dobin, A., Davis, C.A., Schlesinger, F., Drenkow, J., Zaleski, C., Jha, S., Batut,

P., Chaisson, M., and Gingeras, T.R. (2013). STAR: ultrafast universal RNA-seq aligner. Bioinformatics *29*, 15–21.

50. Hao, Y., Hao, S., Andersen-Nissen, E., Mauck, W.M., 3rd, Zheng, S., Butler, A., Lee, M.J., Wilk, A.J., Darby, C., Zager, M., et al. (2021). Integrated analysis of multimodal single-cell data. Cell *184*, 3573–3587.e29.

51. Stuart, T., Butler, A., Hoffman, P., Hafemeister, C., Papalexi, E., Mauck, W.M., 3rd, Hao, Y., Stoeckius, M., Smibert, P., and Satija, R. (2019). Comprehensive integration of single-cell data. Cell *177*, 1888–1902.e21.

52. Seiler, M., Peng, S., Agrawal, A.A., Palacino, J., Teng, T., Zhu, P., Smith, P.G., Cancer Genome Atlas Research Network, Buonamici, S., and Yu, L. (2018). Somatic mutational landscape of splicing factor genes and their functional consequences across 33 cancer types. Cell Rep. *23*, 282–296.e4.

53. Xiang, X., He, Y., Zhang, Z., and Yang, X. (2024). Interrogations of single-cell RNA splicing landscapes with SCASL define new cell identities with physiological relevance. Nat. Commun. *15*. https://doi.org/10.1038/s41467-024-46480-9.

54. Zhou, Y., Zhou, B., Pache, L., Chang, M., Khodabakhshi, A.H., Tanaseichuk, O., Benner, C., and Chanda, S.K. (2019). Metascape provides a biologist-oriented resource for the analysis of systems-level datasets. Nat. Commun. *10*, 1523.

55. Tang, Z., Kang, B., Li, C., Chen, T., and Zhang, Z. (2019). GEPIA2: an enhanced web server for large-scale expression profiling and interactive analysis. Nucleic Acids Res. *47*, W556–W560.

56. Bai, Y., Kosonocky, C.W., and Wang, J.Z. (2024). How our authors are using AI tools in manuscript writing. Patterns (N. Y.) *5*, 101075.

Cover letter Click here to access/download;Personal Cover;Cover Letter.pdf

Dear Scott and Hongling,

On behalf of all coauthors, I am writing to submit the manuscript "**AEnet: a practical tool to construct the splicing associated phenotype atlas at single cell level"** for your consideration as an Article in *GigaScience.*

Alternative splicing (AS) is a key contributor to proteome diversity and cellular heterogeneity. Exploring AS's impact at the single-cell level holds great promise for revealing cellular splicing diversity and understanding AS-gene interplay regulatory mechanisms. However, previous clustering approaches have overlooked the crucial interplay between AS preference and gene expression in defining distinct 'cell types'. Also, they lack the extensibility to reveal the regulatory mechanisms of alternative splicing, infer their regulatory factors, and identify functional pathways. Studies of AS at high resolution are plagued by several inherent limitations. First, AS events are usually quantified as proportional values. The sparsity of scRNA-seq data often introduces the "NaN" (not a number) challenge during calculations when the denominator (the total number of AS events for a given gene in a single cell) is zero. Second, AS events can also be affected by batch effects. Third, not all AS events contribute functionally to cellular heterogeneity.

Here, we present the AS-gene Expression Network (AEnet) to explore core AS events and gene co-expression patterns in a network at the single-cell level. Using our algorithm, we find that both splice site preferences and gene expressions contribute to cellular heterogeneity during clustering, though they exhibit dynamic interplays and varying weights across different datasets. AEnet has three major functions: first, to construct AS profiling-based clusters and separate cell subpopulations with distinct AS-gene expression networks; second, to identify key splicing factors for AS clusters; and third, to pinpoint functional pathways involved in

the regulatory mechanisms based on core subsets of AS events. By applying the AEnet method to malignant cells, pan-cancer T cells, and cell differentiation during gastrulation, we demonstrate the power of AEnet in fine-grained clustering of cells by disease or developmental states, linking upstream regulatory factors and downstream action pathways, highlighting novel isoforms of functional importance, and constructing AS landscapes along the AS-based developmental trajectory.

We have incorporated a performance evaluation of AENet along with a comparison to SCASL. In a simulated dataset, AENet demonstrates robust performance in identifying AS clusters with high accuracy and consistency. Notably, AENet outperforms SCASL in handling noise and complex biological systems. AENet mitigates batch effects and captures cell heterogeneity in large-scale, multi-sample datasets.

This work furthered our understanding of the interplay between AS and gene expression and how this interaction promotes cellular heterogeneity. The concept of AEnet is poised to be instrumental in the realm of single-cell omics, especially as three-generation sequencing technologies continue to advance. Thank you in advance for your consideration of our manuscript for publication in *GigaScience*.

We are looking forward to hearing from you.

Yours Sincerely,

Liang Wu, PhD
On behalf of all authors
BGI Research, Shenzhen 518083, China.
Email: wuliang@genomics.cn
