# Supplementary material for: AEnet: a practical tool to construct the splicing-associated phenotype atlas at a single cell level
Source: Gigascience. 2025 Sep 24;14:giaf110. doi: 10.1093/gigascience/giaf110 (PMC12457822; doi:10.1093/gigascience/giaf110)
Supplement: giaf110_GIGA-D-25-00064_Revision_1 [file giaf110_giga-d-25-00064_revision_1.pdf]

GigaScience

AEnet: a practical tool to construct the splicing associated phenotype atlas at single cell level

--Manuscript Draft--

**Manuscript Number:**	GIGA-D-25-00064R1
**Full Title:**	AEnet: a practical tool to construct the splicing associated phenotype atlas at single cell level
**Article Type:**	Technical Note
**Funding Information:**	GuangDong Basic and Applied Basic Research Foundation (2021A1515110832) — Miss Liang Wu<br>Shenzhen Key Laboratory of Single-Cell Omics (ZDSYS20190902093613831) — Miss Liang Wu<br>Shenzhen Science and Technology Program (LCYX20220620105200001) — Miss Liang Wu<br>Key Technologies Research and Development Program of Anhui Province (2021YFC2501900) — Miss Liang Wu<br>Shenzhen Science and Technology Program (JCYJ20240813150001003) — Dr Xi Chen
**Abstract:**	Alternative splicing (AS), a crucial driver of proteomic diversity, is a fundamental source of cellular heterogeneity alongside gene expression levels. AS is closely linked to various physiological and pathological processes, including tumor progression and embryonic development. Single-cell RNA sequencing (scRNA-seq) technologies capture AS events through junction reads at cellular resolution, enabling the identification of core AS events that regulate specific cell types or states. However, single-cell sequencing technology and its data are plagued by inherent limitations such as shallow sequencing depth, high dropout rates, and batch effects. Furthermore, previous clustering approaches have overlooked the crucial interplay between alternative splicing (AS) and gene expression in defining distinct ‘cell types,’ posing ongoing challenges in this field. In this study, we present a novel method called the AEnet, which combines gene expression levels with AS patterns to profile cellular heterogeneity and define what we term ‘cell subpopulations’. AEnet also identifies key AS events and infers the regulatory mechanisms underlying these events. By applying AEnet to tumor cells, pan-cancer immune cells, and embryonic cells, we demonstrate enhanced cell clustering, the identification of novel AS events with potential functional importance, and the discovery of the key splicing factors involved in cell state transformation. The application of AEnet provides new insights into cellular heterogeneity and its role in both physiological and pathological processes.
**Corresponding Author:**	Liang Wu<br><br>CHINA
**Corresponding Author Secondary Information:**	
**Corresponding Author's Institution:**	
**Corresponding Author's Secondary Institution:**	
**First Author:**	Liang Wu
**First Author Secondary Information:**	
**Order of Authors:**	Liang Wu<br>Shang Liu

Powered by Editorial Manager® and ProduXion Manager® from Aries Systems Corporation

	Xi Chen
	Yinqi Bai
	Shiping Liu
	Xiaohu Huang
	Yuhang Wang
	Waidong Huang
	Pengfei Qin
	Rui Li
	Xuanxuan Zou
	Wending Pang
**Order of Authors Secondary Information:**	
**Response to Reviewers:**	Reviewer reports:<br><br>Reviewer #1: This paper presents AEnet, a cell clustering method based on the combined gene expression levels and AS patterns for cell-type annotation with scRNA-seq data. This idea is very interesting and I can see the great efforts made by the authors. However, as a method paper, the limited benchmarked datasets and methods make this work too simple, shallow, and not convinced. I have several concerns about the contents of this paper:<br><br>(1) Since the inference of AS patterns is the foundation for subsequent analysis and the authors proposed a new approach to infer the AS patterns from scRNA-seq data, this approach should be benchmarked with other known methods, such as BRIE, Outrigger, MARVEL, and their own earlier attempt, DESJ-detection, to demonstrate the superior accuracy or efficiency to quantify the AS patterns.<br><br>(2) The authors assumes that the combined gene expression levels and AS patterns is more accurate in identifying the cell clusters than the traditional strategies. If so, the ablation study should be performed by only considering the AS patterns or gene expression levels, to demonstrate the superior accuracy of AEnet compared to so-called Anet (AS only) or Enet (gene expression only) by replacing combined AE matrix with the AS patterns matrix or gene expression matrix and keeping the other parts of processing totally same as that in AEnet.<br><br>(3) I observe that the authors compared the performance of AEnet and SCASL (single-cell clustering method based on AS) in identifying the cell clusters (Fig. S3). But the authors focused on the performance of reducing batch effects. This is very unreasonable. Since they are both single-cell clustering methods, the evaluation index should be known ARI, NMI, and/or cell-type annotation accuracy based on the ground-truth cell labels, rather than the patient samples.<br><br>(4) Beside the ablation study, the comparison with other widely-used single-cell clustering methods should be conducted, e.g., the Seurat clustering method based on the combined AE matrix (ASP & E), and scSHC, with the known ARI and NMI indexes.<br><br>(5) In Fig. 2, the authors mentioned that AEnet can decrease the bias or batch effects. If so, the benchmarked evaluations with other known methods (such as Seurat CCA, Harmony, fastMNN, Conos, LIGER) should be performed to demonstrate the superior accuracy or efficiency to reduce the bias or batch effects.<br><br>(6) Since the AEnet is a universal method for clustering, more benchmarked datasets are needed, including the datasets obtained from different scRNA-seq technologies (Smart-seq, Microwell-seq, 10X Genomics, etc.), species, tissues, conditions, diseases (including more non-cancer diseases).<br>(7) Typo "cell cells" in "how alternative splicing influences cell cells and cell states".

Powered by Editorial Manager® and ProduXion Manager® from Aries Systems Corporation

Reviewer #2: Liu et al. developed a novel software (AEnet) for analysing alternative splicing (AS) at single-cell resolution. This software leverages both alternative splicing and gene expression profiles (ASP and EXP, respectively) to build ASP-EXP links. These links are then utilised for identifying cell clusters, cluster-specific regulatory splicing factors and isoforms, and pathway enrichment analysis. Overall, the software demonstrates sufficient novelty over existing single-cell AS tools to warrant publication. The narrative is also sensible and coherent. My only comments pertain to the benchmarking of this software and application of this software beyond the contexts reported in this manuscript, and they are aimed at improving the already high-quality of the manuscript.

Main comments:

1. In Fig. S3A (left panel) and S3C (left panel), were the cell clusters identified by AEnet biologically meaningful? For example, do the clusters demonstrate expression of lineage-specific genes that would enable cell type assignment or identification?

2. The clustering capability of AEnet has not been benchmarked against datasets in which the cell types are known (ground truth). Is AEnet able to delineate the different cell populations whose identity have been previously assigned? The myriads of published peripheral blood mononuclear cells (PBMCs) and bone marrow mononuclear cells (BMMCs) datasets consists of a wide diversity of confidently-assigned cell types suitable for this benchmarking exercise.

3. The clustering capability of AEnet was compared against SCASL and Seurat by visually inspecting the clustering profile on reduced dimension space. While this initial visual-based inspection is sensible, an objective measurement could serve as an additional method of comparison. Two such metrices for clustering assessment are adjusted rand index (ARI) and normalised mutual information (NMI).

4. The ability of AEnet to mitigate batch effect was compared against Seurat and SCASL. For Seurat specifically, the batch effect was "corrected" using the "FindIntegrationAnchors" and "IntegrateData" functions. These functions integrate multiple samples, but do not adjust for batch effect per se. To enable a fair comparison with Seurat, or other means of batch correction, more suitable approaches for adjusting batch effect should be benchmarked against. The canonical correlation analysis (CCA) and Harmony are two batch correction approaches implemented by Seurat that may be benchmarked against.

5. It would be of particular interest to benchmark AEnet against other splicing-based clustering approaches. The variational autoencoder (VAE) algorithm implemented by scQuint (PMID: 35229721) and DOLPHIN (https://apc01.safelinks.protection.outlook.com/?url=https%3A%2F%2Fwww.researchsquare.com%2Farticle%2Frs-5474597%2Fv1&data=05%7C02%7Cwuliang%40genomics.cn%7C19524fec513b46a5e91e08dd65e932ef%7C853aa2281adc4d91bb286065c1e9963d%7C0%7C0%7C638778774818200358%7CUnknown%7CTWFpbGZsb3d8eyJFbXB0eU1hcGkiOnRydWUsIlYiOiIwLjAuMDAwMCIsIlAiOiJXaW4zMiIsIkFOIjoiTWFpbCIsIldUIjoyfQ%3D%3D%7C0%7C%7C%7C&sdata=YxkFdnbgoIhPFzDFAkxTMiwh1cM8PlxZo58M7e2jgco%3D&reserved=0) have demonstrated convincing clustering ability.

6. I appreciate the detailed demonstration of AEnet on tumour-infiltrating T cells and embryonic gastrulation using AEnet, and the identification of regulatory splicing factors in these scenarios. In haematological malignancies such as myelodysplastic syndrome (MDS) and myeloproliferative neoplasm (MPN), more than half of the patients have mutations in splicing factors, namely SF3B1, SRSF2, and U2AF1, and therefore are known to have dysregulated splicing profiles. Moreover, several AS events related to these splicing factors have already been experimentally validated, for example, SEPTIN2 and MAP3K7 are mis-spliced in SFB31-mutant MDS patients. These splicing events may serve as positive controls in benchmarking exercise. Furthermore, related to comment no. 2 above, the haematopoietic compartment consists of a wide variety of cell types suitable for showcasing AEnet functionalities. Therefore, these cancer types are biologically relevant for benchmarking and for
demonstrating the application of AEnet. One such example is a dataset that consists of

Powered by Editorial Manager® and ProduXion Manager® from Aries Systems Corporation

SFB31-mutant MDS samples (PMID: 37582363).

7. The cornerstone of single-cell RNA-sequencing analysis is differential gene and splicing analysis. AEnet excels in revealing regulatory splicing factors, isoforms, and pathways within a cluster or condition. However, AEnet does not enable comparison across different clusters or conditions, e.g., disease versus healthy states. While the lack of this functionality is not a limitation in itself, this may be detailed in the Discussion to aid users in deciding which single-cell AS tools will be best suited to meet their research goals.

8. For the purpose of reproducibility and to convince prospective users, like myself, to use AEnet, please may the authors provide the codes used to generate AEnet-related figures in the manuscript. A R Markdown format is highly encouraged, if possible.

Minor comments:
9. Please may the authors provide the pre-defined list of splicing factors in a Supplementary Table.
10. In paragraph 1 of the Introduction, "… analyzing profilings of AS…" should be "…analyzing profiles of AS…".

11. In paragraph 2 of the Introduction, the authors alluded to the inflated false negatives exemplified by published single-cell AS tools. Has this limitation been demonstrated or investigated for these tools? If not, I recommend the authors to tone down this assertion.

12. In paragraph 2 of the Introduction, the authors also mentioned that current single-cell AS tools do not identify functional pathways. But MARVEL has a functionality to identify enriched functional pathways (PMID: 36631981).

13. In paragraph 3 of the Introduction, the authors listed three challenges of single-cell AS analysis. Please may the authors provide the relevant citations for each of these challenges.

Reviewer #3: In this work, Liu & Chen et al. present AEnet, a computational method for analyzing alternative splicing (AS) at the single-cell level. By integrating AS and scRNA-seq data, they identify alternative splicing pattern (ASP) clusters and use them to define cell subpopulations. The authors demonstrate the utility of their method in three biological contexts: tumor immunotherapy response, tumor-infiltrating T cells, and embryonic development. This work integrates two distinct but complementary data modalities, AS and scRNA-seq, into a single framework, improving the characterization of cellular heterogeneity. The method is well-designed and effectively applied, but several issues should be addressed before publication.
Major points:
* Retaining NaNs for zero counts is valid, but even low-count ASPs may introduce random fluctuations in PSIs. Have you tested different count thresholds? And how does it affect results? If an ASP is rare and mostly NaN, how do you decide whether to include it? Could this introduce bias against infrequent but biologically relevant splicing events?

* The cell populations identified by ASP are demonstrated to be independent of RNA-based clustering. Have you directly compared ASP-only, RNA-only, and joint clustering to assess whether they provide unique biological insights?

* The uniform noise model used in evaluations may not reflect biological noise. Have you tested more realistic noise models?

* AEnet claims to bypass the batch effects, but the mechanism should be better explained. Is this achieved because correlations are calculated within each sample and aggregated later?

* From your results (e.g., Fig. S5C), there seem to be cross-cluster interactions. Are you concerned that rigid clustering may overlook meaningful relationships between clusters?

*   Alternative splicing inherently provides positional information. Have you analyzed whether splicing events are enriched in specific genomic regions?

Minor points:
*   The criteria for defining a cell subpopulation should be stated in the main text.
*   Are the same sequencing results used for gene count matrix and ASP detection?
*   The first section provides both method description and validation/benchmarking. Consider separating them into distinct sections and expanding each more thoroughly.
*   Can AEnet handle larger single-cell datasets? How does it scale computationally?
*   Figures in the first section lack self-explanatory captions. Some (e.g., Fig. 1C, S1B) do not provide sufficient information.
*   The anchor ASP identification plots need x-axes, and the y-axes should be labeled "Number of ASP-Gene Links" instead of just "Degree."
*   The cited work from Regev's lab in the introduction is a bit old. Are there more recent references that should be included?

Point-by-point response to the referees' comments

The general response to the reviewers:
We sincerely thank all the reviewers for their time and for providing valuable comments and suggestions, which have significantly helped us improve our method. During the revision process, we have carefully addressed all the points raised, as detailed below and marked in blue.

Reviewer reports:

Reviewer #1: This paper presents AEnet, a cell clustering method based on the combined gene expression levels and AS patterns for cell-type annotation with scRNA-seq data. This idea is very interesting and I can see the great efforts made by the authors. However, as a method paper, the limited benchmarked datasets and methods make this work too simple, shallow, and not convinced. I have several concerns about the contents of this paper:

(1) Since the inference of AS patterns is the foundation for subsequent analysis and the authors proposed a new approach to infer the AS patterns from scRNA-seq data, this approach should be benchmarked with other known methods, such as BRIE, Outrigger, MARVEL, and their own earlier attempt, DESJ-detection, to demonstrate the superior accuracy or efficiency to quantify the AS patterns.

Response: Thank you for highlighting this critical point. We have rigorously benchmarked AEnet against BRIE, Outrigger, MARVEL, and our prior method DESJ-detection, demonstrating its superior accuracy in quantifying AS patterns.
Current methods (BRIE [1], Outrigger [2], and MARVEL [3]) are all fundamentally

Powered by Editorial Manager® and ProduXion Manager® from Aries Systems Corporation

annotation-dependent and therefore unable to detect unannotated AS events. Additionally, these tools are limited in their ability to detect MSE (Fig. R1A, Table R1). Since MARVEL has been compared with other methods and demonstrated to be the optimal one in its category in a previous study [3], we next compared the AS events detected by AEnet and MARVEL using the demo dataset of MARVEL, which includes induced pluripotent stem cells (iPSCs) and iPSC-derived endoderm cells [4] (Fig. R1B). MARVEL identified a total of 20,509 SE, 1,279 MXE, 8,295 RI, 5,163 A5SS, 5,832 A3SS, 5,818 AFE, 2,072 ALE, and 0 MSE (Fig. R1C). In MARVEL's iPSC dataset, AEnet detected 722,278 additional AS patterns (63% unannotated) across 12,866 genes, with MSE detection 3.2-fold higher than MARVEL (Fig. R1D-E). These findings highlight AEnet's comprehensive capability to detect a wide range of AS events—including unannotated and complex patterns—except for intron retention events.

TypeBRIEOutriggerMARVELDESJ-detectionAEnet
SE√/√√√
MXE/√√√√
RI//√//
A3SS//√√√
A5SS//√√√
AFE//√√√
ALE//√√√
MSE//√√√
Unannotated AS events///√√

Table R1. Summary of the five methods.

Figure R1. (A) The common types of Alternative splicing. (B) The comparison between AEnet and MARVEL in the detection of ASPs. (C) The number of alternative splicing events detected by MARVEL. (D) The number of alternative splicing events detected by AEnet as well as the comparison with MARVEL. (E) The demo example of FHOD3 for the rare patterns detected by AEnet.

Compared to our homologous method DESJ-detection [5], AEnet overcomes critical limitations in low-depth robustness. DESJ-detection's single-junction PSI calculation leads to 38% error rates in low-coverage scenarios (Fig. R1F-G). AEnet mitigates this by focusing on junction reads with shared splice sites (requiring ≥5 supporting reads) and standardizing read distributions to transcript usage ratios, reducing PSI error by 47% ($p<0.001$, Fig. R1H).

Taken together, AEnet outperforms existing methods in detecting unannotated alternative splicing events, resolving complex AS patterns, and ensuring reliable quantification across varying data depths. We have incorporated this suggestion into the manuscript at Page9, Line 201-223 and revised Figure S3.

Figure R1. (G) Schematic illustration of PSI calculation in DESJ-detection. (H) Impact of Low Gene Expression on the Accuracy of DESJ Detection: A Case Study Using Genes Without Alternative Splicing Events. (I) Schematic Diagram of the calculation of PSI for Alternative Splicing Pattern (ASP).

(2) The authors assumes that the combined gene expression levels and AS patterns is more accurate in identifying the cell clusters than the traditional strategies. If so, the ablation study should be performed by only considering the AS patterns or gene expression levels, to demonstrate the superior accuracy of AEnet compared to so-called Anet (AS only) or Enet (gene expression only) by replacing combined AE matrix with the AS patterns matrix or gene expression matrix and keeping the other parts of processing totally same as that in AEnet.

Response: We appreciate your insightful comment. To serve the question, three independent datasets—iPSC [2], T cell [6], and HCC [7]—were used for evaluation, and detailed information is provided in Table R2. Comprehensive ablation analyses using published cell type annotations as ground truth demonstrated that the joint ASP-EXP model (AEnet) consistently outperformed the standalone AS-only (Anet) and expression-only (Enet) approaches (Fig. R2A). Specifically, the median ARI scores were 0.81 (AEnet), 0.68 (Anet), and 0.77 (Enet) for the iPSC dataset; 0.58 (AEnet), 0.10 (Anet), and 0.39 (Enet) for the HCC dataset; and 0.42 (AEnet), 0.10 (Anet), and

Powered by Editorial Manager® and ProduXion Manager® from Aries Systems Corporation

0.32 (Enet) for the T cell dataset (Fig. R2B–D). These results demonstrate that integrating ASP features with gene expression markedly improves clustering resolution and biological interpretability. Furthermore, AEnet generated the most informative low-dimensional embeddings across all datasets (Fig. R2E–G), accurately capturing cellular architecture in iPSCs, delineating major lineages in HCC, and resolving functionally distinct T cell subsets—capabilities that were limited or absent in the AS-only and EXP-only models. Thus, AEnet consistently achieves superior performance by jointly leveraging alternative splicing and gene expression to reveal biologically meaningful cellular heterogeneity. We have incorporated this suggestion into the manuscript at Page15, Line 311-324 and revised Figure 2A-D and S7.

Table R2. Summary of the three datasets

Figure R2. (A) Schematic of the assessment pipeline for ASP-only, RNA-only, and joint clustering analyses. (B-D) Quantitative benchmarking of clustering concordance using (upper) Adjusted Rand Index (ARI) and (down) Normalized Mutual Information (NMI) metrics across the three networks. Statistical significance was assessed via one-sided Wilcoxon rank-sum tests. * $P < 0.05$, ** $P < 0.01$, *** $P < 0.001$, **** $P < 0.0001$; n.s., not significant.

Figure R2. (E–F) Uniform Manifold Approximation and Projection (UMAP) visualizations showing the published cell type annotations (right) and the predicted clusters (left) derived from the ASP-EXP (AEnet) network (top), expression-only (EXP) network (middle), and ASP-only network (bottom) for (E) full-length iPSC and (F) hepatocellular carcinoma (HCC) single-cell RNA-seq datasets. (G) UMAP visualizations showing the published cell type annotations (botton) and the predicted clusters (upper) derived from the ASP-EXP (AEnet) network (left), expression-only (EXP) network (middle), and ASP-only network (right) for the T cells datasets. Clustering solutions shown represent those with the highest adjusted Rand index (ARI).

(3) I observe that the authors compared the performance of AEnet and SCASL (single-cell clustering method based on AS) in identifying the cell clusters (Fig. S3). But the authors focused on the performance of reducing batch effects. This is very unreasonable. Since they are both single-cell clustering methods, the evaluation index should be known ARI, NMI, and/or cell-type annotation accuracy based on the ground-truth cell labels, rather than the patient samples.
Response: We appreciate the reviewer's suggestion to use ARI and NMI metrics for a more objective comparison between AEnet and SCASL[8]. We have now included a quantitative comparison using these metrics (Figure R3). The results show that AEnet achieves significantly higher ARI and NMI scores compared to SCASL, indicating its superior performance in cell clustering. This demonstrates that AEnet not only reduces batch effects but also provides more accurate cell cluster identification compared to SCASL.
Comprehensive benchmarking demonstrates that AEnet consistently outperforms SCASL in clustering accuracy, achieving higher ARI scores across all three datasets: iPSC (AEnet: 0.81 vs. SCASL: 0.37), T cell (AEnet: 0.42 vs. SCASL: 0.29), and HCC (AEnet: 0.58 vs. SCASL: 0.35) (Fig. R3A-C). Moreover, AEnet produced more informative low-dimensional embeddings across all datasets. In the iPSC dataset, SCASL failed to effectively distinguish iPSCs from NPCs, whereas AEnet accurately captured these distinctions (Fig. R3A, D). In the HCC dataset, AEnet robustly separated three major lineages—lymphocytes, myeloid cells, and malignant epithelial cells—while SCASL showed limited resolution between myeloid and lymphoid populations (Fig. R3B, E). Similarly, in the T cell dataset, SCASL generated overlapping clusters and failed to resolve distinct functional subsets, in contrast to the well-delineated subpopulations identified by AEnet (Fig. R3C, F). Collectively, these results demonstrate that integrating alternative splicing features with gene expression enables AEnet to more accurately capture cellular heterogeneity and biologically meaningful substructures, establishing it as a superior tool for single-cell clustering. We have incorporated this suggestion into the manuscript at Page15, Line 325-342 and revised Figure 2E-G and S8A-C.

Powered by Editorial Manager® and ProduXion Manager® from Aries Systems Corporation

Figure R3. (A-C) Quantitative benchmarking of clustering concordance using (left) ARI and (right) NMI metrics of AEnet and SCASL. (D–F) UMAP visualizations showing the published cell type annotations (right) and the predicted clusters (left) derived from AEnet (upper) and SCASL (botton) for: (D) full-length iPSC (E) hepatocellular carcinoma (HCC) single-cell RNA-seq datasets, and the (F) T cell dataset. Clustering solutions shown represent those with the highest adjusted Rand index (ARI). Statistical significance was assessed via one-sided Wilcoxon rank-sum tests. * P < 0.05, ** P < 0.01, *** P < 0.001, **** P < 0.0001; n.s., not significant.

(4) Beside the ablation study, the comparison with other widely-used single-cell clustering methods should be conducted, e.g., the Seurat clustering method based on the combined AE matrix (ASP & E), and scSHC, with the known ARI and NMI indexes.
Response: We fully appreciate your careful assessment of this issue. We evaluated the clustering performance of AEnet, scSHC[9], and Seurat[10] using ARI and NMI as quantitative metrics. As scSHC does not permit manual adjustment of clustering resolution or specification of the number of clusters, we applied the method using its default parameters, which yielded a single clustering result. Moreover, scSHC does not provide functionality for generating low-dimensional embeddings.
To ensure consistency across methods, the cell–junction count matrix was used as input for both scSHC and Seurat. Comprehensive benchmarking demonstrates that AEnet consistently outperforms Seurat and scSHC in clustering accuracy (Fig. R4A–C). Specifically, the median ARI scores for the iPSC dataset were 0.81 (AEnet), 0.81 (Seurat), and 0.82 (scSHC); for the HCC dataset, 0.58 (AEnet), 0.29 (Seurat), and 0.10 (scSHC); and for the T cell dataset, 0.42 (AEnet), 0.36 (Seurat), and 0.26 (scSHC) (Fig. R4A–C). Seurat produced overly complex and less interpretable embeddings in the T cell dataset, compared to AEnet (Fig. R4D-F). Additionally, in both the HCC and T cell datasets, Seurat generated several small, fragmented clusters. Notably, these mini-clusters were primarily driven by junctions from specific genes—such as ACTG1—and did not reflect genuine splicing heterogeneity (Fig. R4G). Instead, they were associated with differential gene expression, suggesting that Seurat may be suboptimal for detecting key alternative splicing events. Thus, AEnet outperforms both Seurat and scSHC in clustering accuracy across multiple datasets, offering more biologically meaningful clustering and interpretable embeddings.
We sincerely thank the reviewer for this insightful recommendation. In response, we have conducted the suggested comparative analyses. However, given that our manuscript primarily aims to introduce and validate AEnet in the context of alternative splicing analysis—and that the compared methods (Seurat and scSHC) are primarily designed for gene expression rather than splicing-based inference—we have decided not to include these results in the main text at this stage. We believe this choice helps maintain focus and coherence in the manuscript, and we greatly appreciate the reviewer’s understanding on this point.

Figure R4. (A-C) Quantitative benchmarking of clustering concordance using (left) ARI and (right) NMI metrics of AEnet and Seurat. (D–F) UMAP visualizations showing the published cell type annotations (right) and the predicted clusters (left) derived from AEnet (upper) and Seurat (botton) for: (D) full-length iPSC (E) hepatocellular carcinoma (HCC) single-cell RNA-seq datasets, and the (F) T cell dataset. Clustering solutions shown represent those with the highest adjusted Rand index (ARI). (G) The differentially junction for S24 in the T cells dataset. Statistical significance was assessed via one-sided Wilcoxon rank-sum tests. * P < 0.05, ** P < 0.01, *** P < 0.001, **** P < 0.0001; n.s., not significant.

(5) In Fig. 2, the authors mentioned that AEnet can decrease the bias or batch effects. If so, the benchmarked evaluations with other known methods (such as Seurat CCA, Harmony, fastMNN, Conos, LIGER) should be performed to demonstrate the superior accuracy or efficiency to reduce the bias or batch effects.
Response: We thank the reviewer for the insightful comment. In response, we conducted benchmarking comparisons to evaluate AEnet’s ability to mitigate batch effects. As AEnet is tailored to detect splicing heterogeneity, we focused on batch correction performance in the PSI-based framework.

Powered by Editorial Manager® and ProduXion Manager® from Aries Systems Corporation

To ensure a fair comparison, we applied two widely used batch correction methods—Seurat CCA [10] and Harmony[11]—to the cell–junction count matrix prior to PSI computation (Fig. R5A-D). However, these methods are primarily optimized for gene expression data and are not well-suited for alternative splicing analysis. In particular, they lack the sensitivity to detect key splicing events and fail to capture splicing-driven cellular heterogeneity. Consequently, AEnet outperformed both methods in identifying biologically meaningful cell heterogeneity, achieving significantly higher ARI and NMI scores (Fig. R5A-B). Moreover, CCA and Harmony tended to overcorrect, resulting in fragmented clusters driven by expression noise (e.g., ACTG1-associated mini-clusters) rather than true alternative splicing patterns (Fig. R5C-D). By contrast, AEnet successfully preserves AS-based clustering structure and captures shared splicing variation across multiple samples or conditions, compared to SCASL (Fig. R5E-H). Although methods like fastMNN[12], Conos[13], and LIGER [10] are also widely used for batch effect correction, their applicability to PSI matrices is similarly limited, and they are not designed to model splicing-specific heterogeneity. In summary, AEnet offers a splicing-aware alternative to general batch correction methods, demonstrating superior performance in preserving biologically relevant AS variation across diverse datasets. We have incorporated this suggestion into the manuscript at Page 15-16, Line 343-353 and revised Figure S9.

Figure R5. (A-B). Quantitative benchmarking of clustering concordance using (A) Adjusted Rand Index (ARI) and (B) Normalized Mutual Information (NMI) metrics across integration methods. (C) UMAP visualizations showing the published cell type annotations (botton) and the predicted clusters (upper) derived from AEnet (left), Harmony (middle), and CCA (right) for the T cells datasets. Clustering solutions shown represent those with the highest adjusted Rand index (ARI). (E) The differentially junction for S22 in the T cells dataset. (E-F) UMAP shows the clustering of cell types determined by AEnet (E) and SCASL (F) (left panel) and patient clustering (right panel) for epithelial cells from multiple patients. (G-H). UMAP shows the clustering of cell types determined by AEnet (G) and SCASL (H) (left panel) and patient clustering (right panel) for T cells from the CRC (Colorectal cancer) and RHCC (Recurrent Hepatocellular carcinoma) datasets. Statistical significance was assessed via one-sided Wilcoxon rank-sum tests * P < 0.05, ** P < 0.01, *** P < 0.001, **** P < 0.0001; n.s., not significant

(6) Since the AEnet is a universal method for clustering, more benchmarked datasets are needed, including the datasets obtained from different scRNA-seq technologies (Smart-seq, Microwell-seq, 10X Genomics, etc.), species, tissues, conditions, diseases (including more non-cancer diseases).
Response: We thank the reviewer for raising this important point regarding the generalizability of AEnet. Owing to the ultra-high sequencing depth and full-length coverage of transcripts provided by the Smart-seq2 platform, most of our alternative splicing (AS)-based analyses have been conducted using Smart-seq2 data. Nevertheless, AEnet remains effective when sequencing depth is adequate. To further demonstrate its versatility, we applied AEnet to a widely used 10X Genomics PBMC dataset (Fig. R6A) [14]. We have incorporated this suggestion into the manuscript at Page16, Line 354-366 and revised Figure S9.
Due to the limited sequencing depth and only single-end coverage for transcript (5‘ end or 3’ end) of 10X data, alternative splicing (AS)-based analyses are constrained by the low detection rate of splicing events. However, alternative polyadenylation (APA) events, which also reflect isoform-level regulation, are main AS events that could be investigated in 10X data. In this dataset, AEnet identified 190 anchor alternative polyadenylation (APA) events and delineated 7 APA-defined clusters (Fig. R6B). These clusters corresponded well to known immune cell subpopulations, including B cells, $CD4^+$ T cells, $CD8^+$ T cells, dendritic cells, macrophages, mast cells, monocytes, neutrophils, natural killer (NK) cells, and regulatory T (Treg) cells (Fig. R6C). Clustering results were consistent with established biological annotations. Quantitative benchmarking further confirmed AEnet’s robustness, achieving a median Adjusted Rand Index (ARI) of 0.83 and a Normalized Mutual Information (NMI) of 0.78 (Fig. R6D). These results demonstrate that AEnet can effectively capture the contribution of isoform usage to cellular heterogeneity based on 10x Genomics datasets.

Figure R6. (A) Schematic of the assessment pipeline for AEnet applied to the PBMC

Powered by Editorial Manager® and ProduXion Manager® from Aries Systems Corporation

dataset from 10x Genomics. (B) Heatmap showing APA classes derived from dimensionality reduction using AEnet. (C) UMAP visualization of cell clustering based on alternative polyadenylation profiles. (D) Quantitative benchmarking of clustering performance using ARI and NMI, evaluated against the ground truth annotations of the PBMC dataset.

(7) Typo "cell cells" in "how alternative splicing influences cell cells and cell states".

Response: The phrase "cell cells" was indeed a typographical error, and we have corrected to "how alternative splicing influences cell types and cell states" (Page3, Line 85) .

Reviewer #2: Liu et al. developed a novel software (AEnet) for analysing alternative splicing (AS) at single-cell resolution. This software leverages both alternative splicing and gene expression profiles (ASP and EXP, respectively) to build ASP-EXP links. These links are then utilised for identifying cell clusters, cluster-specific regulatory splicing factors and isoforms, and pathway enrichment analysis. Overall, the software demonstrates sufficient novelty over existing single-cell AS tools to warrant publication. The narrative is also sensible and coherent. My only comments pertain to the benchmarking of this software and application of this software beyond the contexts reported in this manuscript, and they are aimed at improving the already high-quality of the manuscript.

Main comments:

1. In Fig. S3A (left panel) and S3C (left panel), were the cell clusters identified by AEnet biologically meaningful? For example, do the clusters demonstrate expression of lineage-specific genes that would enable cell type assignment or identification?
Response: We are grateful for this thoughtful comment. The clusters identified by AEnet in Fig. S3A and S3C do exhibit clear biological relevance. In Fig. S3A (Fig. R7A), AEnet successfully uncovered shared AS heterogeneity across multiple patients in the lung cancer cell dataset[15]. The resulting clusters reflected a continuum of therapeutic response, progressing from progressive disease (PD; subpopulation S2), to residual disease (RD; S0), to a normal response state (S1), as visualized along the UMAP_1 axis (Fig. R7A-B). The marker genes of S1 (N) were enriched in alveolar signatures, including AQP4, SFTPB/C/D, NKX2-1, and FOXA2, while S2 (PD) was associated with elevated expression of prothrombin activation genes (PLAT, PLAUR), gap-junction proteins (GJB2/3/5), and the well-known EMT (epithelial-mesenchymal transition) marker EPCAM (Fig. R7C). This description has been incorporated into the revised manuscript (Page 21, Lines 420–427) and revised Figure 3C-D.
In the colorectal cancer (CRC) [6] and recurrent hepatocellular carcinoma (RHCC) [7]T cell datasets, AEnet also effectively captured alternative splicing-driven heterogeneity among T cell populations (Fig. R7D). Specifically, subpopulation S3 expressed naïve T cell markers (IL7R, CCR7, LEF1), S0 and S5 were enriched in memory T cell markers (CD52, ANXA1, CREM), S1 displayed effector T cell markers (NKG7, GZMA/B), S2 and S4 represented exhausted T cell states (PDCD1, CTLA4, HAVCR2), and S6 corresponded to proliferative T cells (MKI67, TOP2A) (Fig. R7E). Notably, AEnet did not stratify T cells into traditional $CD4^+$ and $CD8^+$ lineages, suggesting that alternative splicing plays a more prominent role in modulating functional state transitions rather than in defining classical T cell lineages. TThis interpretation is in the revised manuscript at Page 23, Line 475-487 and revised Figure S10E.

Figure R7. (A) UMAP shows the clustering of cell types determined by AEnet for epithelial cells from multiple patients. (B) The Sankey plot shows the overlapping of cells between Time Points and Clusters determined by AEnet. (C) The heatmap displays the expression of marker genes across cell clusters determined by AEnet for epithelial cells. (D) UMAP shows the clustering of cell types determined by AEnet for T cells from the CRC (Colorectal cancer) and RHCC (Recurrent Hepatocellular carcinoma) datasets. (E) The heatmap displays the expression of marker genes across cell clusters determined by AS.

2. The clustering capability of AEnet has not been benchmarked against datasets in which the cell types are known (ground truth). Is AEnet able to delineate the different cell populations whose identity have been previously assigned? The myriads of

Powered by Editorial Manager® and ProduXion Manager® from Aries Systems Corporation

published peripheral blood mononuclear cells (PBMCs) and bone marrow mononuclear cells (BMMCs) datasets consists of a wide diversity of confidently-assigned cell types suitable for this benchmarking exercise.
Response: Thank you for highlighting this important point. We next focused on evaluating the clustering capability of AEnet using published datasets as ground truth references with ARI and NMI as evaluation metrics. To serve the question, three independent datasets—iPSC [2], T cell [6], and HCC [7]—were used for evaluation, and detailed information is provided in Table R3.
Comprehensive ablation analyses using published cell type annotations as ground truth demonstrated that the joint ASP-EXP model (AEnet) consistently outperformed the standalone AS-only (Anet) and expression-only (Enet) approaches (Fig. R8A). Specifically, the median ARI scores were 0.81 (AEnet), 0.68 (Anet), and 0.77 (Enet) for the iPSC dataset; 0.58 (AEnet), 0.10 (Anet), and 0.39 (Enet) for the HCC dataset; and 0.42 (AEnet), 0.10 (Anet), and 0.32 (Enet) for the T cell dataset (Fig. R8B–D). These results demonstrate that integrating ASP features with gene expression markedly improves clustering resolution and biological interpretability. Furthermore, AEnet generated the most informative low-dimensional embeddings across all datasets (Fig. R8E–G), accurately capturing cellular architecture in iPSCs, delineating major lineages in HCC, and resolving functionally distinct T cell subsets—capabilities that were limited or absent in the AS-only and EXP-only models. Thus, AEnet consistently achieves superior performance by jointly leveraging alternative splicing and gene expression to reveal biologically meaningful cellular heterogeneity. We have incorporated this suggestion into the manuscript at Page15, Line 311-324 and revised Figure 2A-D and S7.
As a preliminary attempt, we analyzed a publicly available PBMC dataset (GSE132044), which includes two samples and 528 cells with an average read depth of 1,061,009. However, due to the sequencing format being paired-end 50 bp (PE50) rather than the more informative PE100, the alternative splicing (AS) signal in this dataset was limited. Specifically, only 3,202 alternative splicing patterns were detected, resulting in just 6,524 ASP-EXP links. This led to the identification of only 6 anchor ASPs and no identifiable ASP clusters, rendering this dataset unsuitable for robust evaluation of AEnet's performance (Fig. R8H). We agree with the reviewer that PBMC and BMMC datasets represent valuable benchmarking resources, and we plan to incorporate higher-depth datasets in future evaluations to further demonstrate the generalizability of AEnet.

Table R3. Summary of the three datasets

Figure R8. (A) Schematic of the assessment pipeline for ASP-only, RNA-only, and joint clustering analyses. (B-D) Quantitative benchmarking of clustering concordance using (upper) Adjusted Rand Index (ARI) and (down) Normalized Mutual Information (NMI) metrics across the three networks. Statistical significance was assessed via one-sided Wilcoxon rank-sum tests. * $P < 0.05$, ** $P < 0.01$, *** $P < 0.001$, **** $P < 0.0001$; n.s., not significant.

Figure R8. (E–F) Uniform Manifold Approximation and Projection (UMAP) visualizations showing the published cell type annotations (right) and the predicted clusters (left) derived from the ASP-EXP (AEnet) network (top), expression-only (EXP) network (middle), and ASP-only network (bottom) for (E) full-length iPSC and (F) hepatocellular carcinoma (HCC) single-cell RNA-seq datasets. (G) UMAP visualizations showing the published cell type annotations (botton) and the predicted clusters (upper) derived from the ASP-EXP (AEnet) network (left), expression-only (EXP) network (middle), and ASP-only network (right) for the T cells datasets. Clustering solutions shown represent those with the highest adjusted Rand index (ARI). (H) Detailed summary of the PBMC dataset.

3. The clustering capability of AEnet was compared against SCASL and Seurat by visually inspecting the clustering profile on reduced dimension space. While this initial visual-based inspection is sensible, an objective measurement could serve as an additional method of comparison. Two such metrices for clustering assessment are adjusted rand index (ARI) and normalised mutual information (NMI).

Powered by Editorial Manager® and ProduXion Manager® from Aries Systems Corporation

Response: Thank you for highlighting this important point. We fully agree that clustering performance should be evaluated using objective ground-truth metrics such as Adjusted Rand Index (ARI) and Normalized Mutual Information (NMI), rather than relying solely on visual inspection or improvements in batch correction. To this end, we have conducted comprehensive benchmarking analyses across three datasets—iPSC, T cell, and HCC—using published cell type annotations as ground truth.
AEnet consistently outperformed SCASL [8] in clustering accuracy across all datasets: iPSC (AEnet: 0.81 vs. SCASL: 0.37), T cell (0.42 vs. 0.29), and HCC (0.58 vs. 0.35) (Fig. R9A–C). Moreover, AEnet produced more biologically meaningful and interpretable low-dimensional embeddings. For example, in the iPSC dataset, SCASL failed to distinguish iPSCs from NPCs, while AEnet accurately resolved these subpopulations (Fig. R9A, D). In the HCC dataset, AEnet clearly separated lymphocytes, myeloid cells, and malignant epithelial cells, whereas SCASL blurred boundaries between major lineages (Fig. R9B, E). Similarly, in the T cell dataset, AEnet identified functionally distinct subsets with high fidelity, in contrast to the overlapping and poorly resolved clusters generated by SCASL (Fig. R9C, F). We have incorporated this suggestion into the manuscript at Page15, Line 325-342 and revised Figure 2E-G and S8A-C.
We also compared AEnet to Seurat [10] using ARI and NMI, applying the same cell–junction count matrix to ensure a fair comparison. AEnet consistently achieved superior clustering performance: in the iPSC dataset (AEnet: 0.81, Seurat: 0.81, scSHC: 0.82), HCC dataset (AEnet: 0.58, Seurat: 0.29, scSHC: 0.10), and T cell dataset (AEnet: 0.42, Seurat: 0.36, scSHC: 0.26) (Fig. R10A–C). While Seurat performed comparably in the iPSC dataset, it produced overly complex and fragmented embeddings in the T cell and HCC datasets (Fig. R10D–F). Notably, Seurat generated several small clusters driven by junctions from specific genes—such as ACTG1—that reflected differential gene expression rather than true splicing heterogeneity (Fig. R10G). This suggests Seurat may be suboptimal for identifying isoform-level variation.
Taken together, these results demonstrate that AEnet not only outperforms SCASL but also provides more accurate, biologically meaningful clustering than general-purpose methods like Seurat and scSHC, particularly in detecting alternative splicing-driven cellular heterogeneity.

Figure R9. (A-C) Quantitative benchmarking of clustering concordance using (left) ARI and (right) NMI metrics of AEnet and SCASL. Statistical significance was assessed via one-sided Wilcoxon rank-sum tests. (D–F) UMAP visualizations showing the published cell type annotations (right) and the predicted clusters (left) derived from AEnet (upper) and SCASL (botton) for: (D) full-length iPSC (E) hepatocellular carcinoma (HCC) single-cell RNA-seq datasets, and the (F) T cell dataset. Clustering solutions shown represent those with the highest adjusted Rand index (ARI). * $P < 0.05$, ** $P < 0.01$, *** $P < 0.001$, **** $P < 0.0001$; n.s., not significant.

Figure R10. (A-C) Quantitative benchmarking of clustering concordance using (left) ARI and (right) NMI metrics of AEnet and Seurat. Statistical significance was assessed via one-sided Wilcoxon rank-sum tests. (D–F) UMAP visualizations showing the published cell type annotations (right) and the predicted clusters (left) derived from AEnet (upper) and Seurat (botton) for: (D) full-length iPSC (E) hepatocellular carcinoma (HCC) single-cell RNA-seq datasets, and the (F) T cell dataset. Clustering solutions shown represent those with the highest adjusted Rand index (ARI). (G) The differentially junction for S24 in the T cells dataset. * $P < 0.05$, ** $P < 0.01$, *** $P < 0.001$, **** $P < 0.0001$; n.s., not significant.

4. The ability of AEnet to mitigate batch effect was compared against Seurat and SCASL. For Seurat specifically, the batch effect was "corrected" using the "FindIntegrationAnchors" and "IntegrateData" functions. These functions integrate multiple samples, but do not adjust for batch effect per se. To enable a fair comparison with Seurat, or other means of batch correction, more suitable approaches for adjusting batch effect should be benchmarked against. The canonical correlation analysis (CCA) and Harmony are two batch correction approaches implemented by Seurat that may be benchmarked against.
Response: We thank the reviewer for the insightful comment regarding the need for

more suitable batch correction baselines. In response, we performed benchmarking comparisons to assess the ability of AEnet to mitigate batch effects, particularly in the context of splicing heterogeneity. To this end, we applied commonly used batch correction methods—Seurat CCA [10] and Harmony [11]—to the cell–junction count matrix prior to PSI computation, ensuring fair comparison (Fig. R11A–C). To ensure a fair comparison, we applied two widely used batch correction methods—Seurat CCA and Harmony—to the cell–junction count matrix prior to PSI computation (Fig. R11A-D). However, these methods are primarily optimized for gene expression data and are not well-suited for alternative splicing analysis. In particular, they lack the sensitivity to detect key splicing events and fail to capture splicing-driven cellular heterogeneity. Consequently, AEnet outperformed both methods in identifying biologically meaningful cell heterogeneity, achieving significantly higher ARI and NMI scores (Fig. R11A-B). Moreover, CCA and Harmony tended to overcorrect, resulting in fragmented clusters driven by expression noise (e.g., ACTG1-associated mini-clusters) rather than true alternative splicing patterns (Fig. R11C-D). By contrast, AEnet successfully preserves AS-based clustering structure and captures shared splicing variation across multiple samples or conditions, compared to SCASL (Fig. R5E–H). In summary, AEnet offers a splicing-aware alternative to general batch correction methods, demonstrating superior performance in preserving biologically relevant AS variation across diverse datasets.We have incorporated this suggestion into the manuscript at Page 15-16, Line 343-353 and the revised Figure S9A-D.

Figure R11. (A-B). Quantitative benchmarking of clustering concordance using (A) Adjusted Rand Index (ARI) and (B) Normalized Mutual Information (NMI) metrics across integration methods. (C) UMAP visualizations showing the published cell type annotations (botton) and the predicted clusters (upper) derived from AEnet (left), Harmony (middle), and CCA (right) for the T cells datasets. Clustering solutions shown represent those with the highest adjusted Rand index (ARI). (E) The differentially junction for S22 in the T cells dataset. (E-F) UMAP shows the clustering of cell types determined by AEnet (E) and SCASL (F) (left panel) and patient clustering (right panel) for epithelial cells from multiple patients. (G-H). UMAP shows the clustering of cell types determined by AEnet (G) and SCASL (H) (left panel) and patient clustering (right panel) for T cells from the CRC (Colorectal cancer) and RHCC (Recurrent Hepatocellular carcinoma) datasets. Statistical significance was assessed via one-sided Wilcoxon rank-sum tests * P < 0.05, ** P < 0.01, *** P < 0.001, **** P < 0.0001; n.s., not significant

5. It would be of particular interest to benchmark AEnet against other splicing-based clustering approaches. The variational autoencoder (VAE) algorithm implemented by scQuint (PMID: 35229721) and DOLPHIN (https://apc01.safelinks.protection.outlook.com/?url=https%3A%2F%2Fwww.researchsquare.com%2Farticle%2Frs-5474597%2Fv1&data=05%7C02%7Cwuliang%40genomics.cn%7C19524fec513b46a5e91e08dd65e932ef%7C853aa2281adc4d91bb286065c1e9963d%7C0%7C0%7C638778774818200358%7CUnknown%7CTWFpbGZsb3d8eyJFbXB0eU1hcGkiOnRydWUsIlYiOiIwLjAuMDAwMCIsIlAiOiJXaW4zMiIsIkFOIjoiTWFpbCIsIldUIjoyfQ%3D%3D%7C0%7C%7C%7C&sdata=YxkFdnbgoIhPFzDFAkxTMiwh1cM8PlxZo58M7e2jgco%3D&reserved=0) have demonstrated convincing clustering ability.
Response: We thank the reviewer for this important suggestion. To benchmark AEnet against other splicing-based clustering methods, we evaluated its performance relative to scQuint [16] and DOLPHIN using ARI and NMI as objective metrics.
As scQuint primarily employs a variational autoencoder (VAE) for embedding, we performed clustering on the scQuint-derived embeddings using default settings, with the cell–junction count matrix as input (Fig. R12A). In the iPSC dataset, which presents relatively simple cellular composition, AEnet and scQuint achieved comparable embedding quality (Fig. R12B). However, in the more complex T cell dataset and HCC datasets, AEnet effectively resolved well-defined functional subsets and major lineage (Fig. R12C-D). In contrast, scQuint yielded overlapping clusters and failed to distinguish several key subpopulations (Fig. R12C-D). We further compared AEnet with DOLPHIN using three benchmark datasets provided by the DOLPHIN study, as BAM files required for exon count matrix construction were unavailable for our own datasets (Fig. R12E). Across all three datasets, AEnet demonstrated clustering accuracy comparable to that of DOLPHIN. Specifically, AEnet achieved an ARI of

	0.606 in the PBMC dataset, 0.78 in the PDAC dataset, and 0.43 in the Colon dataset, closely matching DOLPHIN's performance (Fig. R12F–G). In addition, AEnet produced informative low-dimensional embeddings across all three datasets, similar to DOLPHIN (Fig. R12H–J).<br>Collectively, AEnet outperforms scQuint in resolving fine-grained cellular subtypes and matches DOLPHIN's clustering accuracy across diverse benchmark datasets, demonstrating both its robustness and generalizability for splicing-aware single-cell analysis. We have incorporated this suggestion into the manuscript at Page15, Line 325-342 and revised Figure S8D.<br><br>Figure R12. (A) Schematic of the assessment pipeline for AEnet and scQuint. (B-D) Uniform Manifold Approximation and Projection (UMAP) visualizations demonstrate the relative performance of AEnet (left) and scQuint (right) in: (B) full-length iPSC dataset, (C) T ...
**Additional Information:**	
**Question**	**Response**
Are you submitting this manuscript to a special series or article collection?	No
**Experimental design and statistics**<br><br>Full details of the experimental design and statistical methods used should be given in the Methods section, as detailed in our Minimum Standards Reporting Checklist. Information essential to interpreting the data presented should be made available in the figure legends.<br><br>Have you included all the information requested in your manuscript?	Yes
**Resources**<br><br>A description of all resources used, including antibodies, cell lines, animals and software tools, with enough information to allow them to be uniquely identified, should be included in the Methods section. Authors are strongly encouraged to cite Research Resource Identifiers (RRIDs) for antibodies, model organisms and tools, where possible.<br><br>Have you included the information requested as detailed in our Minimum Standards Reporting Checklist?	Yes

**Availability of data and materials**<br><br>All datasets and code on which the conclusions of the paper rely must be either included in your submission or deposited in publicly available repositories (where available and ethically appropriate), referencing such data using a unique identifier in the references and in the “Availability of Data and Materials” section of your manuscript.<br><br>Have you have met the above requirement as detailed in our Minimum Standards Reporting Checklist?	Yes
GigaScience has policies and guidelines in place for the use of generative AI-writing tools such as ChatGPT. If you have used such writing tools to assist with writing the manuscript this must be declared and cited in the text. Authors should not list AI-writing tools and other AI-assisted technologies as an author or co-author and should acknowledge that they are fully responsible for text generated or refined by AI-writing tools.<p><br><br>A summary of use (particularly in the introduction or among methods) needs to be included at the end of the paper, and the outputs should also be included as a supplementary file hosted in GigaDB or other open repositories. Please read our guidelines for more information. <p><br><br>By submitting to GigaScience, you are aware of the journal's AI-writing tools policy, and if you have declared use of such tools below, you have acknowledged this where appropriate in your manuscript and have made a summary of use and outputs available. <p><br><b>AI-assisted writing tools have been used in the preparation of this manuscript?	No

Manuscript Click here to access/download;Manuscript;AEN Manuscript.pdf

AEnet: a practical tool to construct the splicing associated phenotype atlas at single cell level

Shang Liu[1*], Xi Chen[1,2,7,*], Xiaohu Huang[1,2,3], Yuhang Wang[1,2,3], Waidong Huang[1,2,4], Pengfei Qin[1,2,7], Rui Li[2], Xuanxuan Zou[1,2], Wending Pang[1,2,3], Shiping Liu[8,9,#], Yinqi Bai[10,#], Liang Wu[1,2,5,6,7#]

1. BGI Research, Chongqing 401329, China
2. BGI Research, Shenzhen 518083, China
3. School of Biology and Biological Engineering, South China University of Technology, Guangzhou, China.
4. College of Life Sciences, University of Chinese Academy of Sciences, Beijing, China
5. Zhongshan-BGI Precision Medical Center, Zhongshan Hospital, Fudan University, Shanghai, China.
6. Shanxi Medical University-BGI Collaborative Center for Future Medicine, Shanxi Medical University, Taiyuan 030001, China
7. State Key Laboratory of Genome and Multi-omics Technologies, BGI Research, Shenzhen 518083, China
8. BGI Research, Hangzhou 310030, China
9. State Key Laboratory of Genome and Multi-omics Technologies, BGI Research, Hangzhou 310030, China
10. BGI Research, Sanya 572025, China

* These authors contributed equally

# Correspondence: wuliang@genomics.cn; baiyinqi@genomics.cn; liushiping@genomics.cn

Abstract

Alternative splicing (AS), a crucial driver of proteomic diversity, is a fundamental source of cellular heterogeneity alongside gene expression levels. AS is closely linked to various physiological and pathological processes, including tumor progression and embryonic development. Single-cell RNA sequencing (scRNA-seq) technologies capture AS events through junction reads at cellular resolution, enabling the identification of core AS events that regulate specific cell types or states. However, single-cell sequencing technology and its data are plagued by inherent limitations such as shallow sequencing depth, high dropout rates, and batch effects. Furthermore, previous clustering approaches have overlooked the crucial interplay between alternative splicing (AS) and gene expression in defining distinct 'cell types,' posing ongoing challenges in this field. In this study, we present a novel method called the AEnet, which combines gene expression levels with AS patterns to profile cellular heterogeneity and define what we term 'cell subpopulations'. AEnet also identifies key AS events and infers the regulatory mechanisms underlying these events. By applying AEnet to tumor cells, pan-cancer immune cells, and embryonic cells, we demonstrate enhanced cell clustering, the identification of novel AS events with potential functional importance, and the discovery of the key splicing factors involved in cell state transformation. The application of AEnet provides new insights into cellular heterogeneity and its role in both physiological and pathological processes.

Introduction

The diversity of proteomes is an important manifestation of the complexity of organisms, and alternative splicing (AS) is one of the major factors contributing to this diversity [1,2]. The major types of alternative splicing include exon skipping (SE), mutually exclusive exons (MXE), intron retention (IR), alternative 3′ splice site (A3SS), alternative 5′ splice site (A5SS), alternative last exon (ALE), alternative first exon (AFE), and multiple-exon splicing (MSE) have also been identified[3,4]. AS plays a crucial role in various physiological and pathological processes such as embryonic development [5], aging [6], and tumor progression [7,8]. Several key splicing factors are involved in the regulation of AS during disease progression. For instance, RBFOX2 is a master regulator for mesenchymal tissue-specific splicing [9], playing a significant role in the formation of mesenchymal-like states in tumor cells [10]. Recently, single-cell transcriptomics has become a powerful tool for analyzing profiles of AS at high resolution [11,12]. Several recent studies have expanded our understanding of cell-type-specific splicing programs. Huang et al. revealed that

subtype-specific splicing patterns refine the classification of pituitary neuroendocrine tumors (PitNETs) [13]. Anoushka et al. constructed a cross-species isoform atlas, demonstrating conserved and human-specific splicing programs linked to neurodevelopment and disease[14]. Similarly, David et al. showed that neurexin isoforms are cell-type-specific and developmentally stable in the brain, underpinning synaptic identity [15]. Together, these studies underscore the biological relevance and regulatory specificity of AS in health and disease, motivating the development of methods like AEnet for systematic analysis of AS at single-cell resolution.[16].

Although there are several bioinformatic approaches to studying single-cell AS profiling, most are based on canonical RNA-based clustering into cell types and then comparing AS heterogeneity, similar to differential gene expression analysis, between these predefined cell types **(Table S1)**. However, cell types defined by AS can differ substantially from those defined by gene expression, which may result in incomplete detection of splicing heterogeneity. This is exemplified by BRIE [17], Outrigger [18], and our earlier attempt, DESJ-detection [19]. Furthermore, existing single-cell analysis tools lack the extensibility to reveal the regulatory mechanisms of alternative splicing, infer their regulatory factors, and identify functional pathways of specific isoforms—rather than merely those of differentially spliced genes **(Table S1)**. MARVEL [20] addresses this limitation by performing gene ontology (GO) enrichment analysis using the clusterProfiler R package, which can identify enriched pathways among differentially spliced genes. However, this approach only indirectly infers the potential functional relevance of AS events and does not directly assess the role of individual isoforms produced via alternative splicing, thus limiting an in-depth understanding of how alternative splicing influences cell cells and cell states [21,22].

Several issues require further consideration in the design of a single-cell AS analytical pipeline. First, unlike RNA expression, which is typically quantified in absolute values, AS events are usually quantified as proportional values. The sparsity of scRNA-seq data often introduces the "NaN" (not a number) challenge during calculations when the denominator (the total number of AS events for a given gene in a single cell) is zero [23]. Second, AS events can also be affected by batch effects, an inherent limitation of single-cell techniques [24]. Third, not all AS events contribute functionally to cellular heterogeneity [25]. Therefore, both upstream and downstream approaches that account for filtering processes should be incorporated.

To address these challenges, we present the Alternative splicing-gene Expression Network (AEnet, https://github.com/liushang17/AEnet) to explore core AS events and gene co-expression patterns in a network at the single-cell level. Using our algorithm, we find that both splice site preferences and gene expressions contribute to cellular heterogeneity during clustering, though they exhibit dynamic interplays and varying weights across different datasets. We refer to the separated clusters as cell (sub)populations to avoid confusion with either cell types or cell states. The software has three major functions: first, to construct AS profiling-based clusters and separate

cell subpopulations with distinct AS-gene expression networks; second, to identify key splicing factors for AS clusters (analogous to gene markers); and third, to pinpoint functional pathways involved in the regulatory mechanisms based on core subsets of AS events.

By applying the AEnet method to malignant cells with different immunotherapy responses, T cell analysis in pan-cancer, and cell differentiation during embryonic gastrulation, we demonstrate the power of AEnet in fine-grained clustering of cells by disease or developmental states, seamlessly linking upstream regulatory factors and downstream action pathways, highlighting novel isoforms of functional importance, and constructing alternative splicing landscapes along the AS-based developmental trajectory. These findings will deepen our understanding of the role of alternative splicing in tumorigenesis and embryonic development, providing new strategies and ideas for clinical prognosis prediction, tumor immunotherapy, and congenital disease treatment.

Results

The overview of AEnet

We have developed the AEnet method that integrates alternative splicing with gene expression levels to uncover cellular splicing heterogeneity and underlying regulatory mechanisms. In brief, AEnet begins by quantifying alternative splicing patterns (ASP) using junction reads from individual cells during data preprocessing (**Fig. 1A**). The percent spliced-in (PSI) for a specific AS event is defined as the proportion of junction reads curated from all detected junctions that span the same site (**Fig. 1A, bottom panel**). Notably, unlike expression values, which are always nonnegative integers, PSI is assigned as NaN (resulting from division by 0) when no junction reads are detected at a presupposed AS site in a cell (**Fig. S1A**). In scRNA-seq data, the prevalent RNA dropout and shallow sequencing issues make the occurrence of NaN even more challenging within individual cells.

To mitigate this effect, for each ASP and each gene, we limit the calculation of the correlation (Spearman by default) to cells with valid PSI values and the gene's expression level, retaining only those ASP-expression correlation links (referred to as ASP-EXP links) with significant P values (**Fig. 1B**). These statistically significant links indicate potential relationships between gene expression dynamics and the usage preference of specific ASPs across the cells. When multiple scRNA-seq samples are available under any experimental conditions, we retain only ASP-EXP links that share the same correlation trend and appear at a moderately higher frequency (2 by default) to exclude batch effect-induced artifacts (**Fig. 1B, S1B-C**). These steps allow AEN to overcome technical noise and capture common relationships between AS and gene expression.

We hypothesize that the interactions identified in common ASP-EXP links play either direct or indirect roles in post-transcriptional regulation and gene expression diversity. For instance, increased expression of specific splicing factors may promote or inhibit the inclusion of certain exons, thereby modifying the AS profile of target genes (**Fig. S1D**)[26]. Additionally, distinct isoforms are associated with the expression dynamics of multiple genes and influence specific signaling pathways, ultimately contributing to changes in cell state (**Fig. S1E**). In other cases, both alternative splicing and gene expression levels may be dysregulated, leading to aberrant gene function during stress responses or in diseases[27]. We then rank the most frequent ASPs (n = 1500, by default) by summing the common ASP-EXP links associated with each ASP, defining them as anchor ASPs, based on the final ASP-EXP network by AEnet (**Fig. S1F**). For each pair of anchor ASPs, we calculate the Jaccard metric of ASP-EXP links (where genes linked to the ASP are treated as sets) to represent their similarity, thereby constructing a similarity matrix of anchor ASPs for downstream clustering (**Fig. 1B**). The clustering process aims to separate the ASPs into distinct groups, each representing a group of AS events correlated with a set of co-expressed or co-regulated genes (**Fig. S1G**). Following a similar strategy, we constructed a gene similarity matrix to define anchor genes and perform clustering, resulting in discrete gene clusters (**Fig. S1H**). These gene clusters serve as the basis for inferring cell states or functional programs associated with specific ASP clusters.

Taken together, the AEnet pipeline begins with the detection of alternative splicing patterns (ASPs) at the cellular level, then constructs ASP (of gene i)–expression (of gene j) links (ASP-EXP links), and defines the ASP similarity matrix at the sample level. Ultimately, it uncovers specific ASPs and co-expression/regulatory patterns at the cell population level (**Fig. 1C, S2A**). The output of AEnet focuses on evaluating three main biological events (**Fig. 1C, S2B-D**): first, the separation of cell subpopulations with distinct ASP compositions; second, the key splicing factors that influence specific ASP clusters (analogous to gene markers), identified based on a predefined list of splicing factors **(Table S2)**; and third, the functional pathways activated or inhibited by individual or small subsets of ASPs. These downstream analyses demonstrate AEnet’s capability in identifying cellular splicing heterogeneity and regulatory mechanisms.

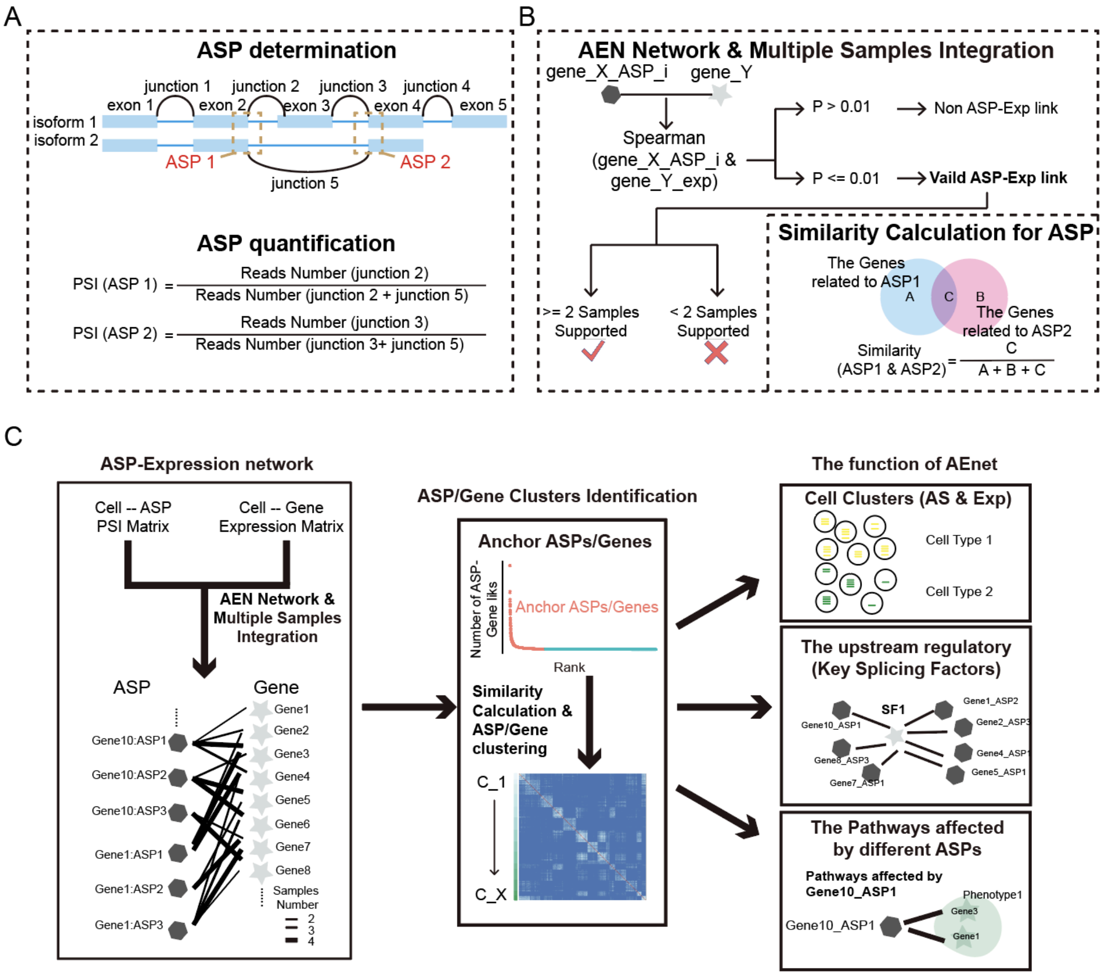

Figure 1. Schematic Diagram of the AEnet Method. A. Schematic Diagram of the identification of Alternative Splicing Pattern (ASP) and the calculation of PSI. **B.** Schematic Diagram of AEN Network Construction, Multiple Samples Integration, and ASP Similarity Calculation. **C.** Scheme of AEnet methods.

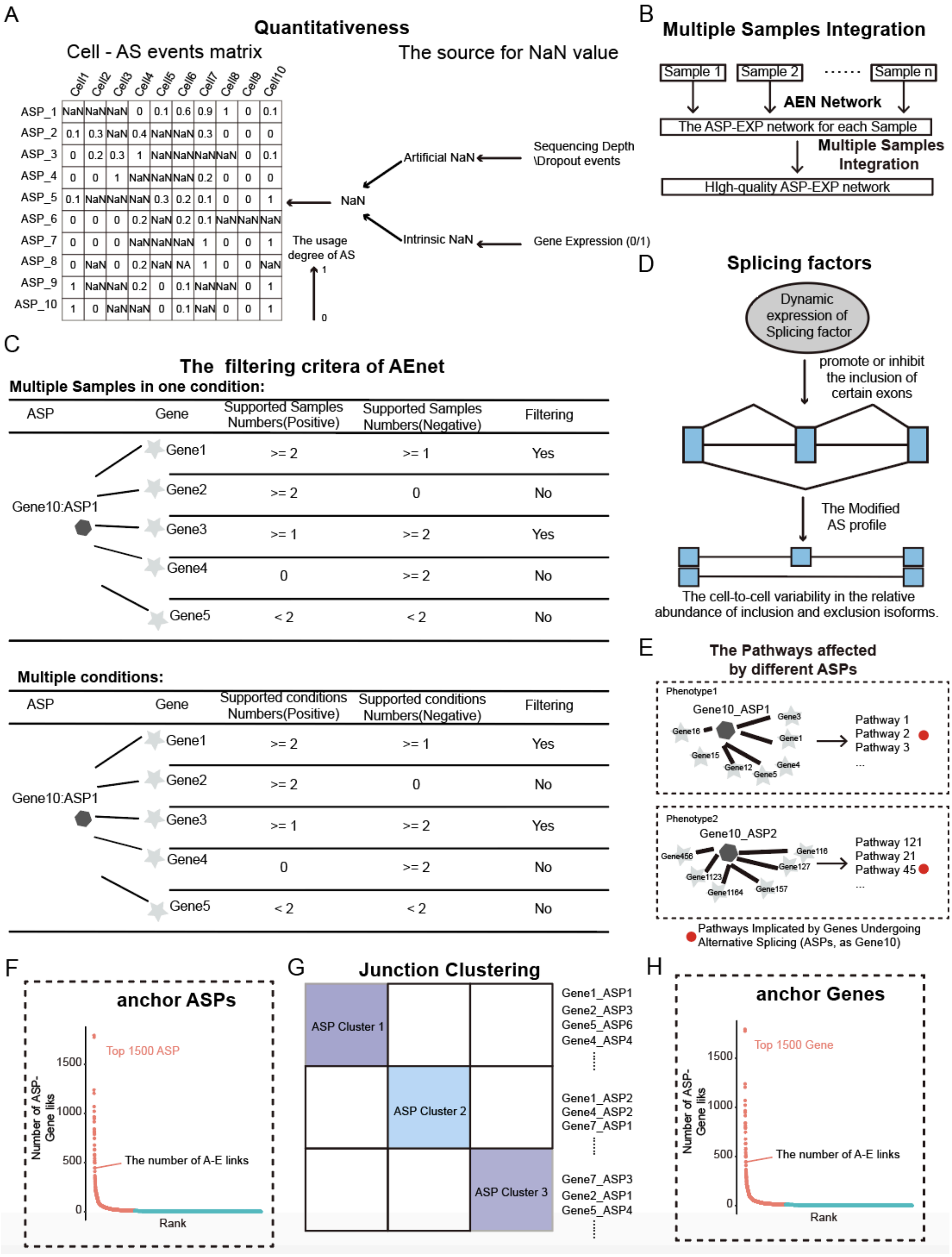

Figure S1. The key difficulties in cell splicing heterogeneity analysis. A. The NaN value problem and its source. **B.** Schematic diagram illustrating the application of AEnet to multiple samples. **C.** Systematic criteria for detecting batch-vulnerable ASP-EXP links across heterogeneous samples and experimental conditions. **D.** Schematic diagram illustrating the effect of splicing factors on the selection of splicing patterns. **E.** Schematic diagram showing how different ASPs of the same gene

impact distinct biological pathways. **F.** The selection of anchor ASPs. **G.** The identification of ASP clusters. **H.** The selection of anchor Genes.

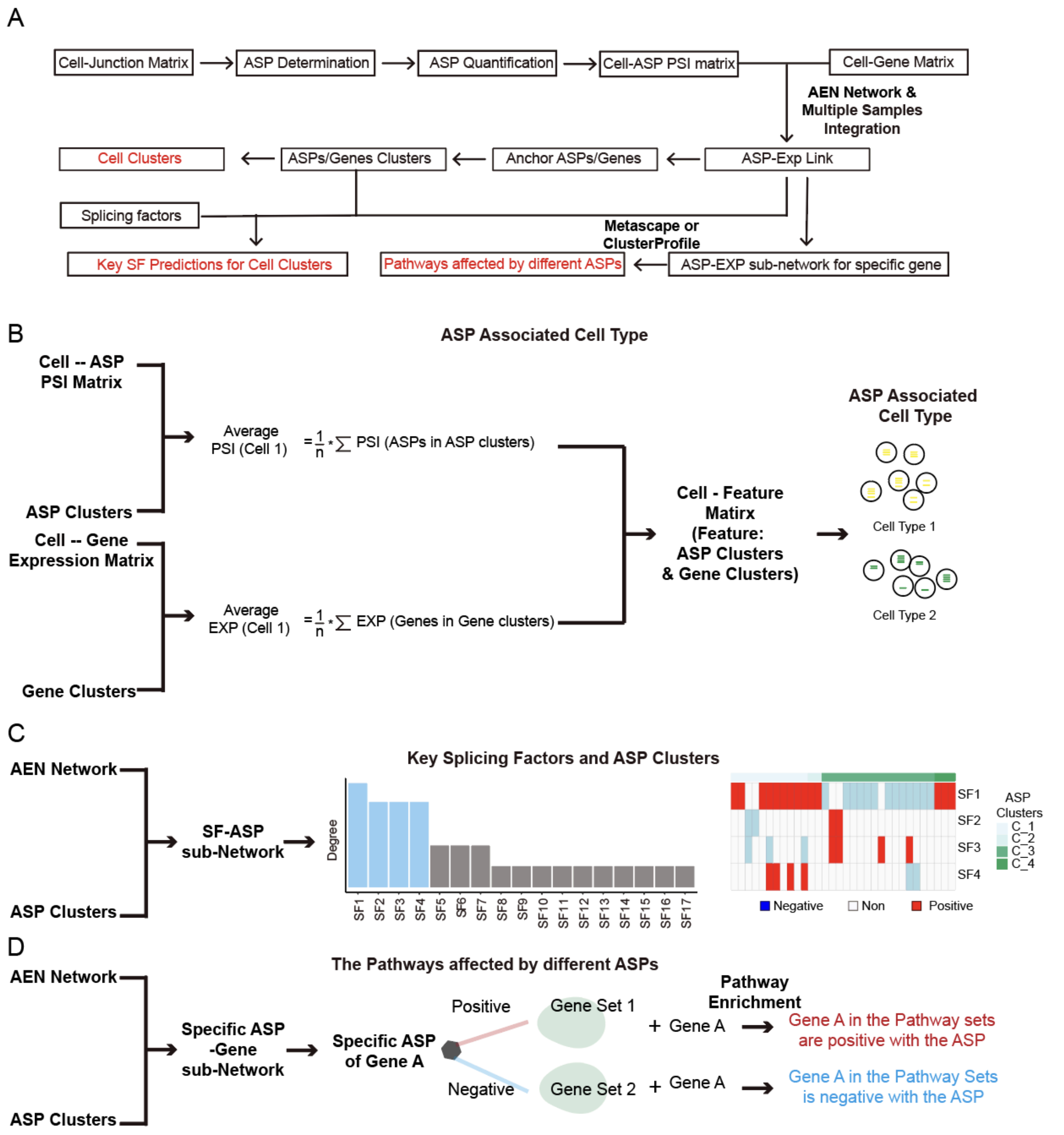

Figure S2. The Three Major Functions of AEnet. A. Schematic overview of the complete AEnet analytical pipeline. **B.** Schematic Diagram of the Analysis of Cell Splicing Heterogeneity. **C.** Identification of the Most Critical Splicing Factors Associated with the ASP Set. **D.** Schematic Diagram of Pathway Inference Associated with Different patterns of ASP.

Evaluation of AEnet for Comprehensive Characterization of Splicing Heterogeneity

We next evaluated AEnet across multiple analytical steps, including ASP–EXP network construction (comprising ASP identification, ASP quantification, and the prediction of ASP and gene clusters), and cell clustering **(Fig. S3A)**. A total of four datasets were used for this evaluation, each accompanied by published annotations serving as ground truth. These datasets, including iPSC[18], HCC[28], and T cell datasets[29], are described in detail in **Table S3**.

We first evaluate the performance of AEnet in the ASP identification **(Fig. S3A-C)**. Current methods, such as BRIE, Outrigger, and MARVEL, are all fundamentally annotation-dependent and therefore unable to detect unannotated AS events. Additionally, these tools are limited in their ability to detect MSE **(Fig. S3B, Table S4)**. Since MARVEL has been compared with other methods and demonstrated to be the optimal one in its category in a previous study, we next compared the AS events detected by AEnet and MARVEL using the demo dataset of MARVEL, which includes induced pluripotent stem cells (iPSCs) and iPSC-derived endoderm cells **(Fig. S3C)**[30]. MARVEL identified a total of 20,509 SE, 1,279 MXE, 8,295 RI, 5,163 A5SS, 5,832 A3SS, 5,818 AFE, 2,072 ALE, and 0 MSE **(Fig. S3D)**. In MARVEL's iPSC dataset, AEnet detected 722,278 additional AS patterns (63% unannotated) across 12,866 genes, with MSE detection 3.2-fold higher than MARVEL **(Fig. S3E-F).** These findings highlight AEnet's comprehensive capability to detect a wide range of AS events—including unannotated and complex patterns—except for intron retention events. Compared to our homologous method DESJ-detection, AEnet overcomes critical limitations in low-depth robustness. DESJ-detection's single-junction PSI calculation leads to 38% error rates in low-coverage scenarios **(Fig. S3G-H)**. AEnet mitigates this by focusing on junction reads with shared splice sites (requiring ≥5 supporting reads) and standardizing read distributions to transcript usage ratios, reducing PSI error by 47% ($p<0.001$, **Fig. S3I**). Taken together, AEnet outperforms existing methods in detecting unannotated alternative splicing events, resolving complex AS patterns, and ensuring reliable quantification across varying data depths.

Low-count ASPs may indeed introduce random fluctuations in PSI estimates, potentially leading to inaccurate quantification, which in turn can affect the construction of ASP-Exp links and the identification of anchor ASPs **(Fig. S4A)**. To evaluate the impact of count thresholds on these outcomes, we systematically tested a range of minimum read count thresholds: 0, 3, 5, 7, and 9 **(Fig. S4A)**. Setting the threshold to 0 yielded the highest number of ASP-Exp links, with ~40% classified as "specific." However, 99% of these specific links were supported by fewer than two samples, suggesting they were likely artifacts of random fluctuations rather than meaningful biological associations **(Fig. S4B-C)**. Similarly, the 0-read threshold also led to an inflated number of anchor ASPs, most of which were linked to

low-confidence, sample-specific signals **(Fig. S4D)**. By contrast, thresholds of ≥3 substantially reduced these spurious associations. Moreover, most biologically meaningful ASP-Exp links were retained when thresholds >0 were applied **(Fig. S4E).** Based on these results, AEnet uses a default threshold of five supporting reads to ensure PSI robustness while minimizing noise.

We also appreciate the reviewer's point about potential bias against rare but biologically relevant splicing events. In AEnet, an ASP is considered "valid" in a sample only if it is supported by ≥5 reads across ≥20 cells. ASPs failing this criterion are excluded from downstream analysis, as their sparsity compromises the reliability of similarity estimates between splicing and expression. To assess whether this filtering excludes informative low-abundance ASPs, we stratified all ASPs into five categories based on their support across cells: Invalid: ≤20 cells (excluded); Type 1: >20–30 cells; Type 2: >30–40 cells; Type 3: >40–50 cells; Type 4: >50 cells. An ASP was assigned to the highest applicable category if it met the criteria in ≥3 samples **(Fig. S4F)**. As expected, higher-support ASPs (fewer NaNs) showed stronger ASP-Exp associations. Nonetheless, ~30% of Type 1 ASPs (i.e., relatively rare but retained) still showed significant correlations with gene expression, and four were identified as anchor ASPs **(Fig. S4G-H)**, indicating their functional relevance. In summary, our results support the use of both read count and sample support thresholds to reduce noise while preserving biological signal. AEnet remains capable of capturing meaningful but infrequent ASPs and provides user-defined thresholding to support flexible analysis tailored to specific research goals.

Finally, to assess the performance of AENet in the identification of ASP clusters, an ASPs-ASPs similarity (Jaccard index) matrix was generated with increasing levels of noise to evaluate AENet's effectiveness in ASP clusters prediction, **(Fig. S5A-B)**. Using a supervised hierarchical clustering method, AENet demonstrated a high accuracy consistency score of approximately 0.9 between the background and the clusters identified, even when noise levels reached 80% **(Fig. S5C-D)**. These results demonstrate AENet's robustness in identifying ASP clusters despite noise. Furthermore, we evaluated AEnet's performance using more realistic noise models, specifically Gaussian and Poisson noise. We simulated increasing levels of both Gaussian and Poisson noise and generated ASP–ASP similarity matrices (Jaccard index) under each noise condition to assess AEnet's robustness in ASP clustering **(Fig. S6A-B, D-E)**. Using a supervised hierarchical clustering approach, AEnet consistently achieved a high accuracy score—approximately 0.9—between the ground truth and the predicted clusters, even under noise levels as high as 90% for both noise models **(Fig. S6C, F)**. These results demonstrate AEnet's resilience to biologically relevant noise, further supporting its reliability in identifying splicing patterns in realistic, noisy settings.

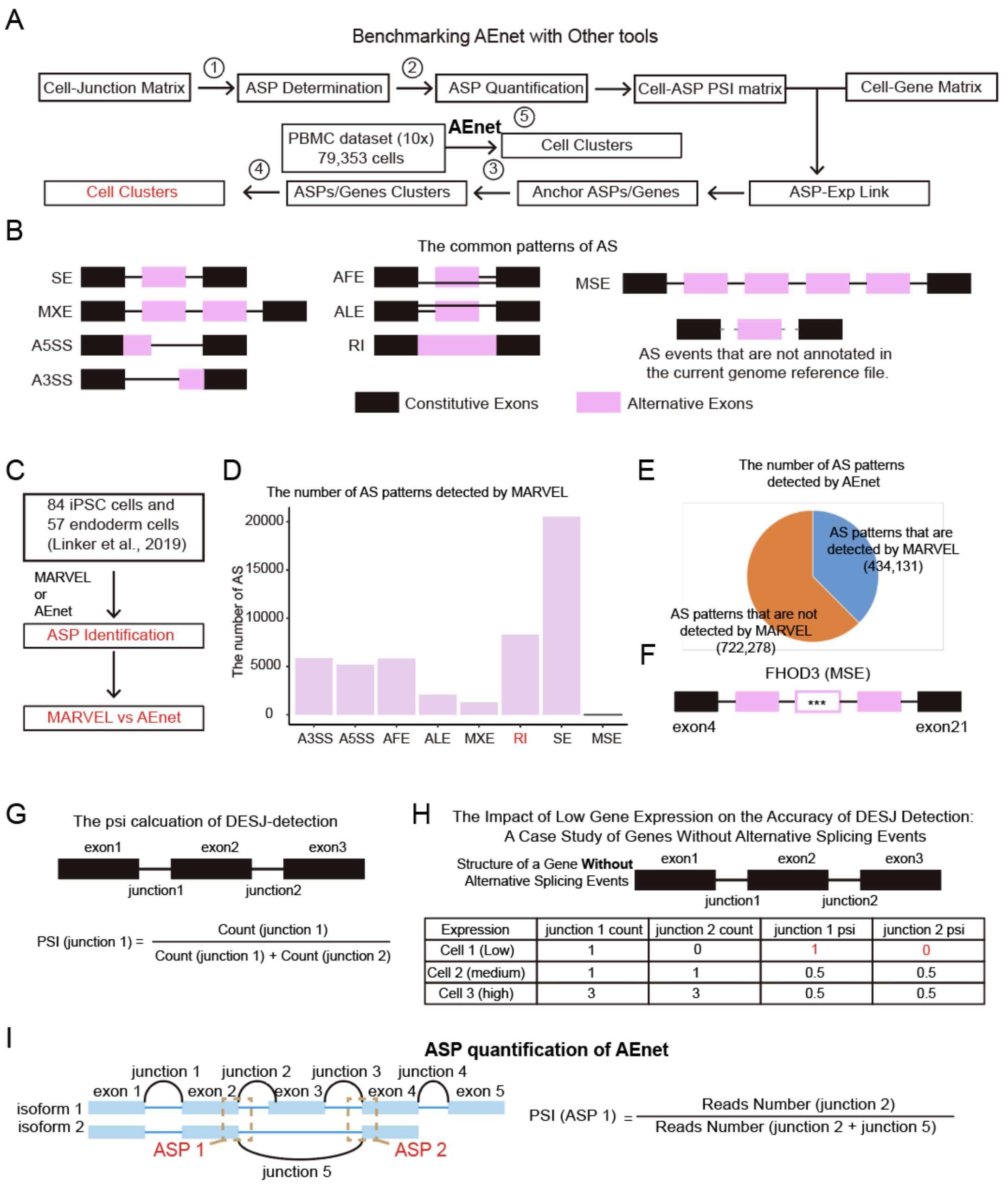

Figure S3. The evaluation of AEnet. A. Schematic overview of the pipeline for the comprehensive evaluation of AEnet at seven points, which is marked in number. **B.** The common types of Alternative splicing. **C.** The rare patterns of Alternative splicing. **D.** Schematic illustration of PSI calculation in DESJ-detection. **E.** Impact of Low Gene Expression on the Accuracy of DESJ Detection: A Case Study Using Genes Without Alternative Splicing Events. **F.** The comparison between AEnet and MARVEL in the detection of ASPs. **G.** The number of alternative splicing events detected by MARVEL. **H.** The number of alternative splicing events detected by AEnet as well as the comparison with MARVEL. **I.** The demo example of FHOD3 for the rare patterns detected by AEnet.

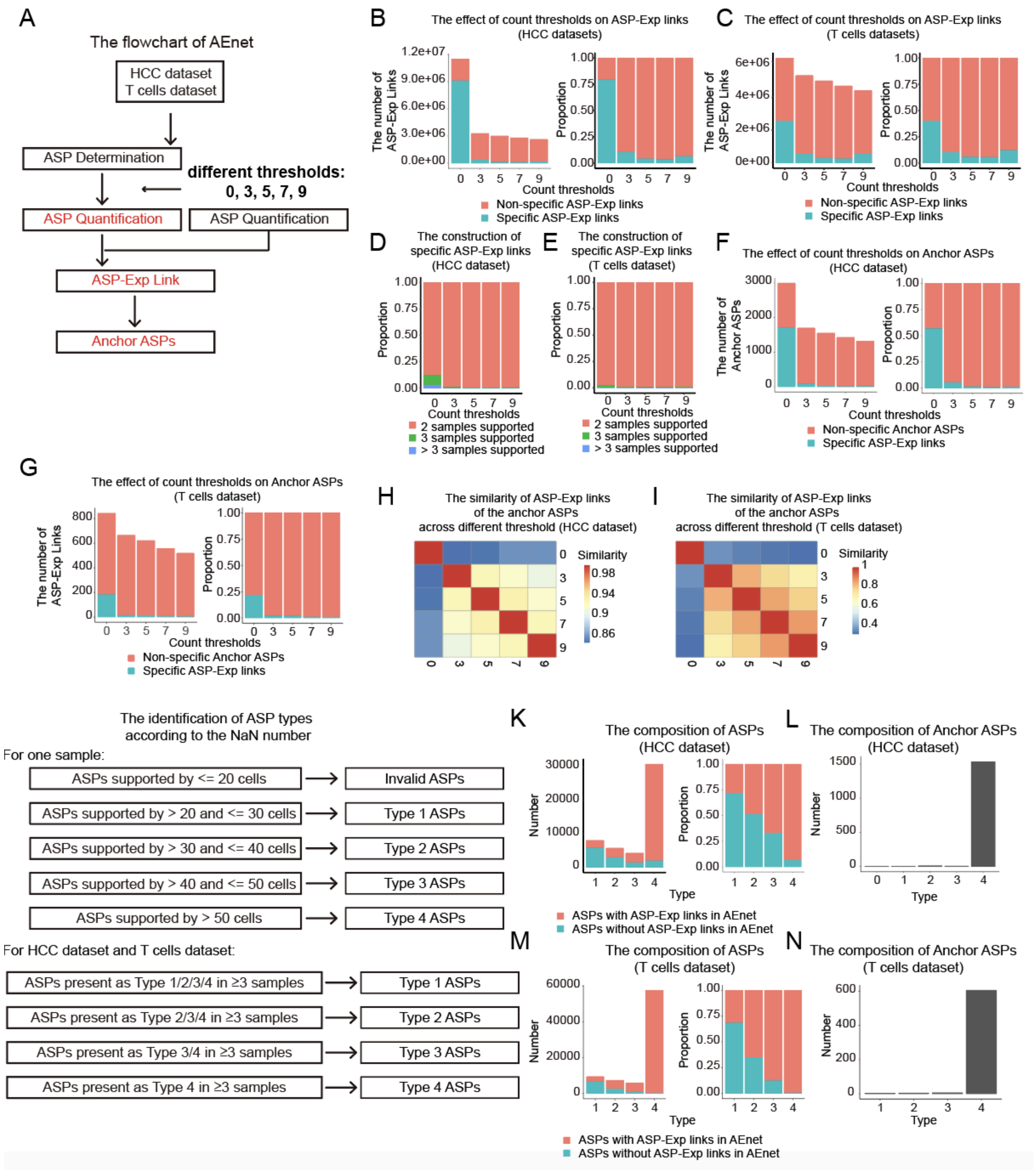

Figure S4. Evaluation of Different Count Thresholds on AEnet Performance. A. Schematic overview of benchmarking strategy for evaluating different count thresholds. **B.** Quantification of ASP-EXP links across varying count thresholds, with color coding denoting link specificity. **C.** Patient-level validation rates for specific ASP-EXP links at different expression count cutoffs. **D.** Quantification of anchor ASPs across varying count thresholds, with color coding denoting link specificity. **E.** The similarity of anchor ASPs across different expression count cutoffs. **F.** Schematic Diagram of the identification of ASP types for one sample or one dataset. **G.** Quantitative analysis of ASP subtypes categorized by presence (blue) or absence (gray) of ASP-EXP links. **H.** Frequency distribution of anchor ASPs stratified by ASP subtype classification.

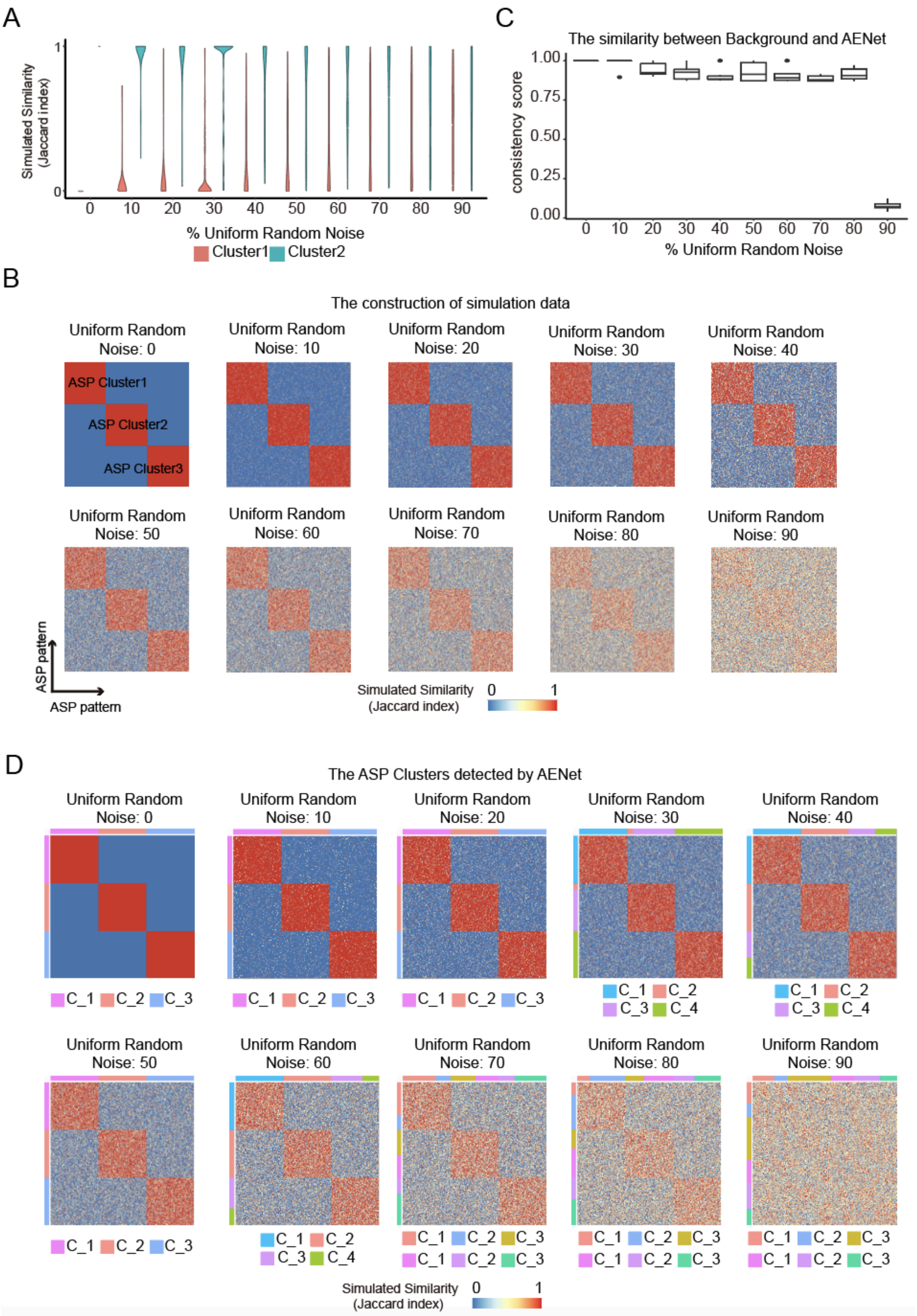

Figure S5. The evaluation of ASP clusters prediction of AEnet with random noise. A-B. Simulated ASPs-ASPs similarity matrix (B) was created with increasing noise (A). C. Box plots present the jaccard index of ASP clusters between the background and AENet. D. ASP clusters identified by AENet in the simulated datasets.

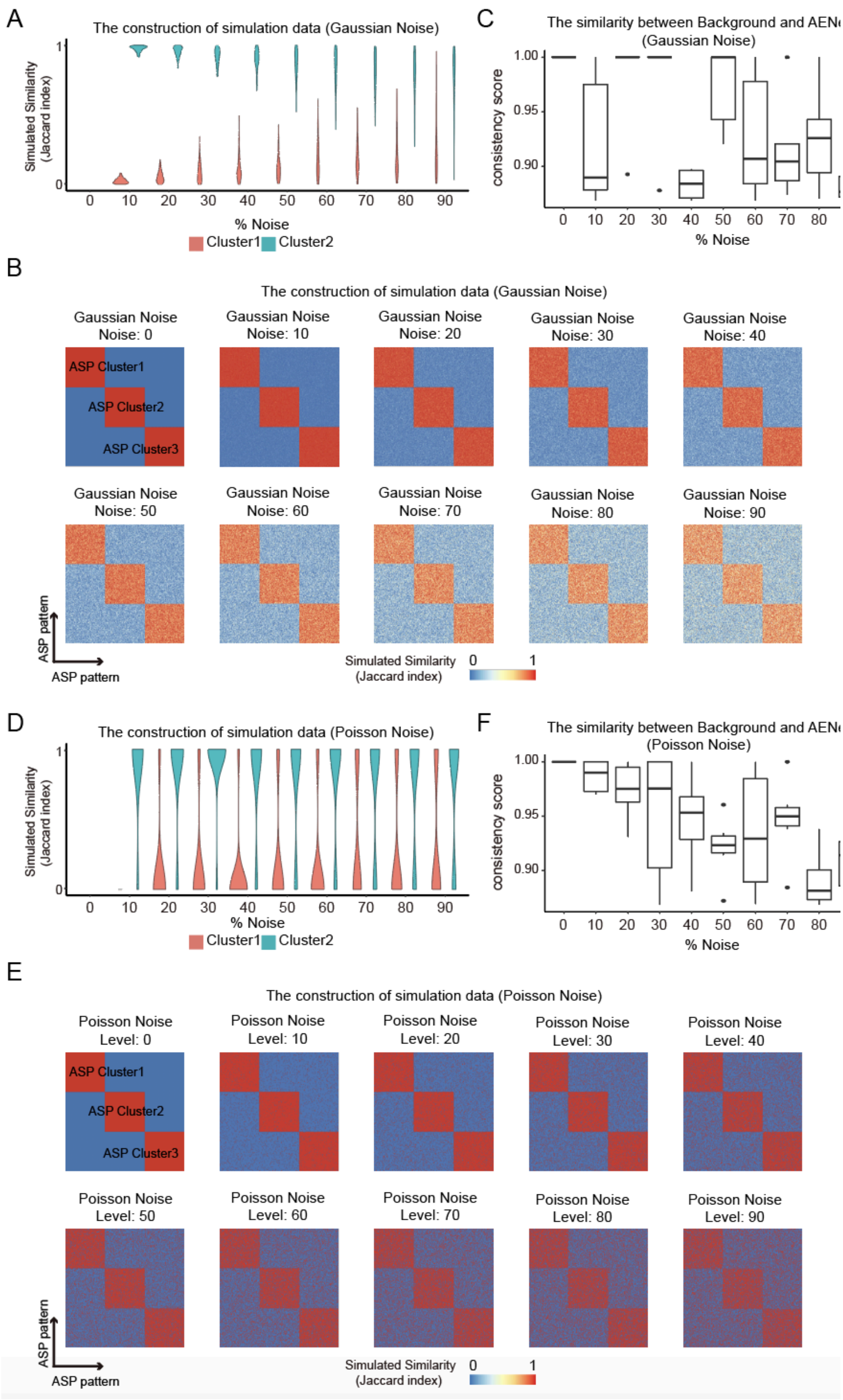

Figure S6. The evaluation of ASP clusters prediction of AEnet with gaussian noise and poisson noise. A-B. Simulated ASPs-ASPs similarity matrix (B) was created with increasing gaussian noise (A). **C.** Box plots present the jaccard index of

ASP clusters between the background and AENet with the increasing gaussian noise. **D-E.** Simulated ASPs-ASPs similarity matrix (E) was created with increasing poisson noise (D). **F.** Box plots present the jaccard index of ASP clusters between the background and AENet with the poisson noise.

Benchmarking AEnet Against Existing Methods

We next evaluated AEnet's performance in capturing cellular heterogeneity using the three benchmarking datasets. Comprehensive ablation analyses, using published cell type annotations as ground truth, demonstrated that the joint ASP-EXP model (AEnet) consistently outperformed the standalone AS-only (Anet) and expression-only (Enet) approaches **(Fig. 2A)**. Specifically, the median ARI scores were 0.81 (AEnet), 0.68 (Anet), and 0.77 (Enet) for the iPSC dataset; 0.58 (AEnet), 0.10 (Anet), and 0.39 (Enet) for the HCC dataset; and 0.42 (AEnet), 0.10 (Anet), and 0.32 (Enet) for the T cell dataset **(Fig. 2B–D)**. These results highlight that integrating ASP features with gene expression significantly enhances clustering resolution and biological interpretability. Moreover, AEnet produced the most informative low-dimensional embeddings across all datasets **(Fig. S7),** accurately reconstructing the cellular architecture in iPSCs, delineating major lineages in HCC, and resolving functionally distinct T cell subsets—tasks in which AS-only or EXP-only models performed suboptimally.

We further compared AEnet with established splicing-aware clustering methods, including SCASL [31] and scQuint [32]**(Fig. 2A)**. Across all three datasets, AEnet consistently outperformed SCASL in clustering accuracy, with higher ARI scores: iPSC (0.81 vs. 0.37), T cell (0.42 vs. 0.29), and HCC (0.58 vs. 0.35) **(Fig. 2E–G)**. In the iPSC dataset, SCASL failed to distinguish iPSCs from NPCs, while AEnet clearly separated these populations **(Fig. S8A)**. In the HCC dataset, AEnet effectively resolved lymphoid, myeloid, and malignant epithelial lineages, whereas SCASL showed poor separation between immune cell types **(Fig. S8B)**. Similarly, in the T cell dataset, SCASL generated overlapping clusters, failing to delineate functional T cell subsets, in contrast to the well-separated clusters produced by AEnet **(Fig. S8C)**. Since scQuint primarily uses a variational autoencoder (VAE) to generate embeddings, we applied clustering to the scQuint-derived embeddings using default parameters. In the iPSC dataset—characterized by relatively simple cellular composition—AEnet and scQuint showed comparable performance. However, in the more complex T cell and HCC datasets, scQuint produced overlapping clusters and failed to resolve key subpopulations **(Fig. S8D)**. Together, these results confirm that joint modeling of alternative splicing and gene expression enables AEnet to more accurately capture cellular heterogeneity compared to other splicing-based methods.

We next assessed AEnet's performance in mitigating batch effects and identifying AS-driven cell heterogeneity. Compared to SCASL—the current leading single-cell

clustering tool based on AS—AEnet showed improved robustness across multiple samples **(Fig. S9)**. In lung cancer datasets[33], AEnet uncovered shared AS heterogeneity across patients, whereas SCASL mainly reflected patient-specific batch effects **(Fig. S9A–B)**. Similarly, in the CRC [29] and RHCC[28] T cell datasets, SCASL failed to detect AS heterogeneity in T cells with minimal batch effects, while AEnet successfully captured these patterns **(Fig. S9C–D)**. These results demonstrate AEnet's ability to detect biologically meaningful AS heterogeneity even in the presence of technical noise or batch variation, making it well-suited for large-scale, multi-sample studies.

To further demonstrate AEnet's versatility, we applied it to a widely used 10x Genomics PBMC dataset **(Fig. S9E–G)** [34]. Due to the limited sequencing depth of 10x platforms, AEnet detected insufficient splicing events for robust AS-based analysis. We therefore focused on alternative polyadenylation (APA), another form of isoform regulation. In this dataset, AEnet identified 190 anchor APA events and defined seven APA-based cell clusters **(Fig. S9E).** These clusters aligned well with known immune cell types, including B cells, $CD4^+$ T cells, $CD8^+$ T cells, dendritic cells, macrophages, mast cells, monocytes, neutrophils, NK cells, and regulatory T cells **(Fig. S9F)**. Clustering performance was quantitatively supported by a median ARI of 0.83 and NMI of 0.78 **(Fig. S9G)**. These results highlight AEnet's ability to uncover isoform-level heterogeneity in shallow-depth datasets, although we note that AS-based analyses remain more challenging in such settings due to limited read coverage.

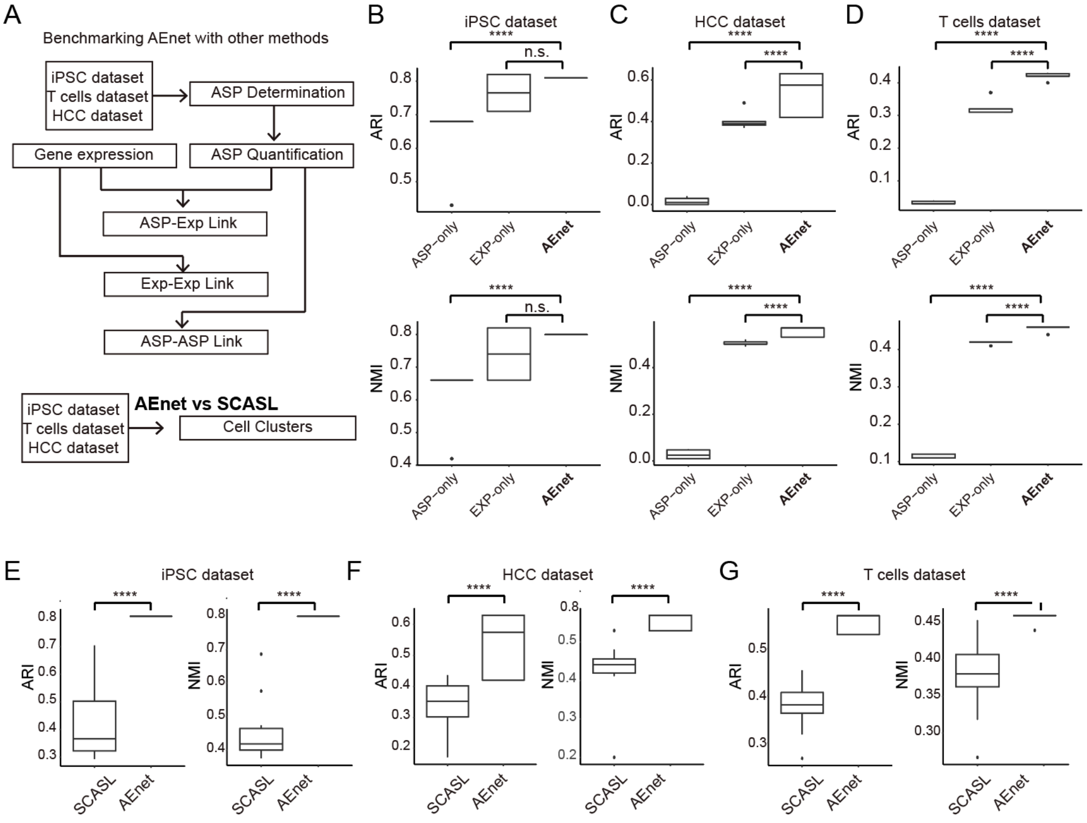

Figure 2. Benchmarking AEnet Against Existing Methods. A. Schematic of the assessment pipeline for ASP-only, RNA-only, and joint clustering analyses. **B-D.** Quantitative benchmarking of clustering concordance using (upper) Adjusted Rand Index (ARI) and (down) Normalized Mutual Information (NMI) metrics across the three networks. Statistical significance was assessed via one-sided Wilcoxon rank-sum tests. **E-G.** Quantitative benchmarking of clustering concordance using (left) ARI and (right) NMI metrics of AEnet and SCASL. Statistical significance was assessed via one-sided Wilcoxon rank-sum tests. * P < 0.05, ** P < 0.01, *** P < 0.001, **** P < 0.0001; n.s., not significant.

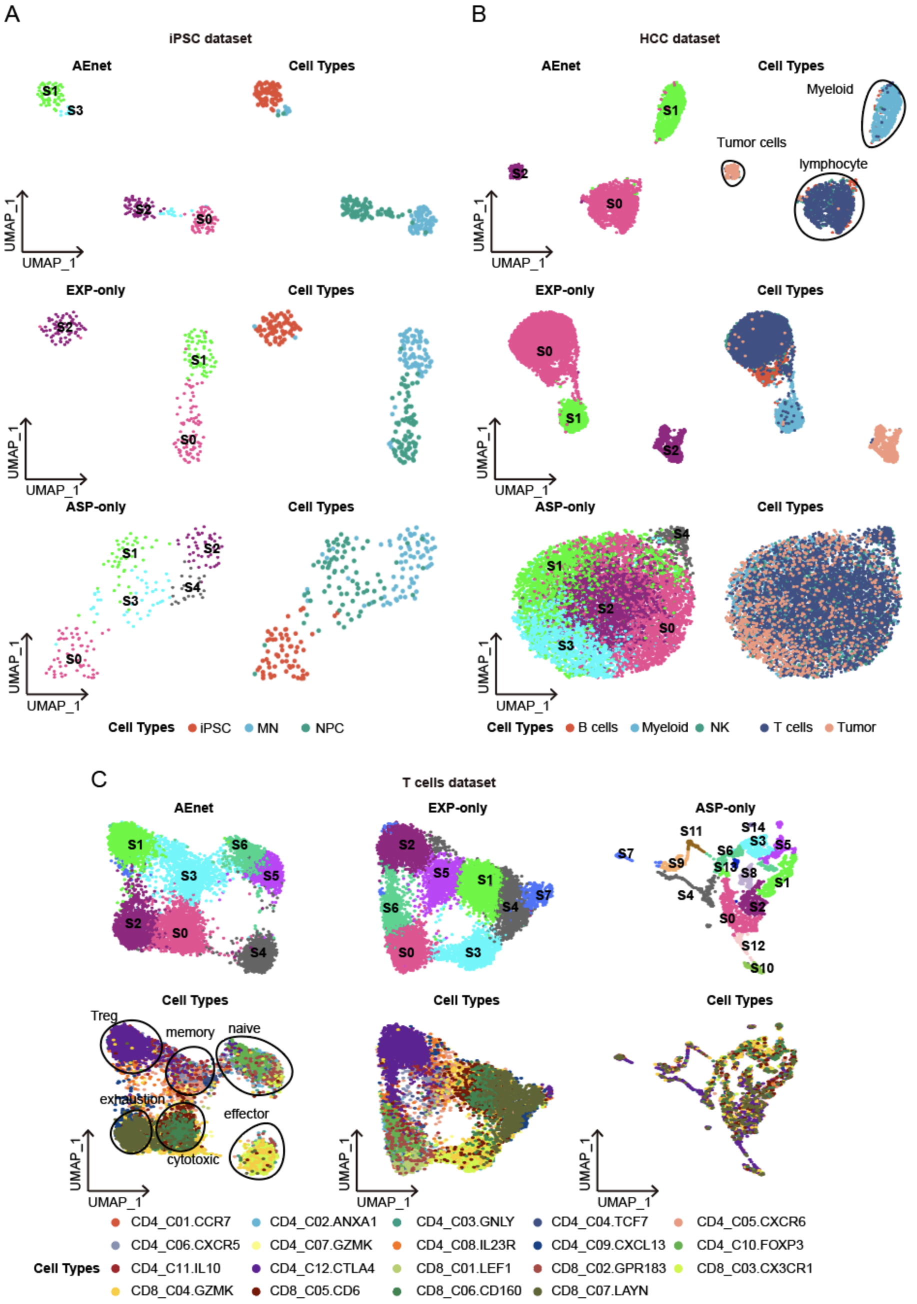

Figure S7. Ablation analysis for AEnet. A-C. Uniform Manifold Approximation and Projection (UMAP) visualizations showing the published cell type annotations (right) and the predicted clusters (left) derived from the ASP-EXP (AEnet) network (top), expression-only (EXP) network (middle), and ASP-only network (bottom) for (A) full-length iPSC and (B) hepatocellular carcinoma (HCC) single-cell RNA-seq datasets. (C) UMAP visualizations showing the published cell type annotations (botton) and the predicted clusters (upper) derived from the ASP-EXP (AEnet) network (left), expression-only (EXP) network (middle), and ASP-only network (right) for the T cells datasets. Clustering solutions shown represent those with the highest adjusted Rand index (ARI).

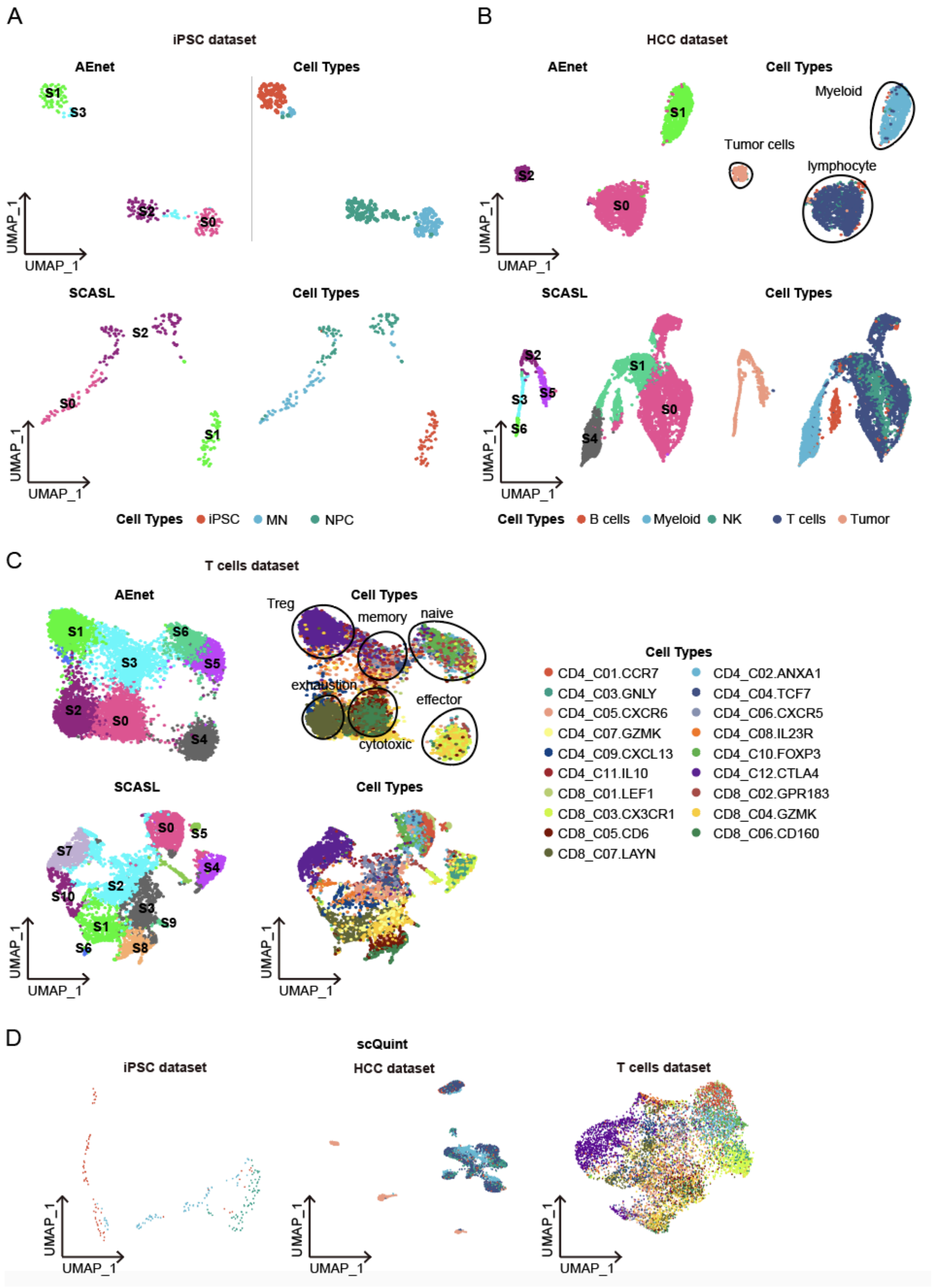

Figure S8. Benchmarking AEnet with SCAL and scQuint. A-C. UMAP visualizations showing the published cell type annotations (right) and the predicted clusters (left) derived from AEnet (upper) and SCASL (botton) for: (A) full-length iPSC (B) hepatocellular carcinoma (HCC) single-cell RNA-seq datasets, and the (C) T cell dataset. Clustering solutions shown represent those with the highest adjusted Rand index (ARI). **D.** UMAP visualizations demonstrate the relative performance of scQuint (right) in: full-length iPSC dataset, T cells sequencing data, and hepatocellular carcinoma (HCC) single-cell RNA-seq data.

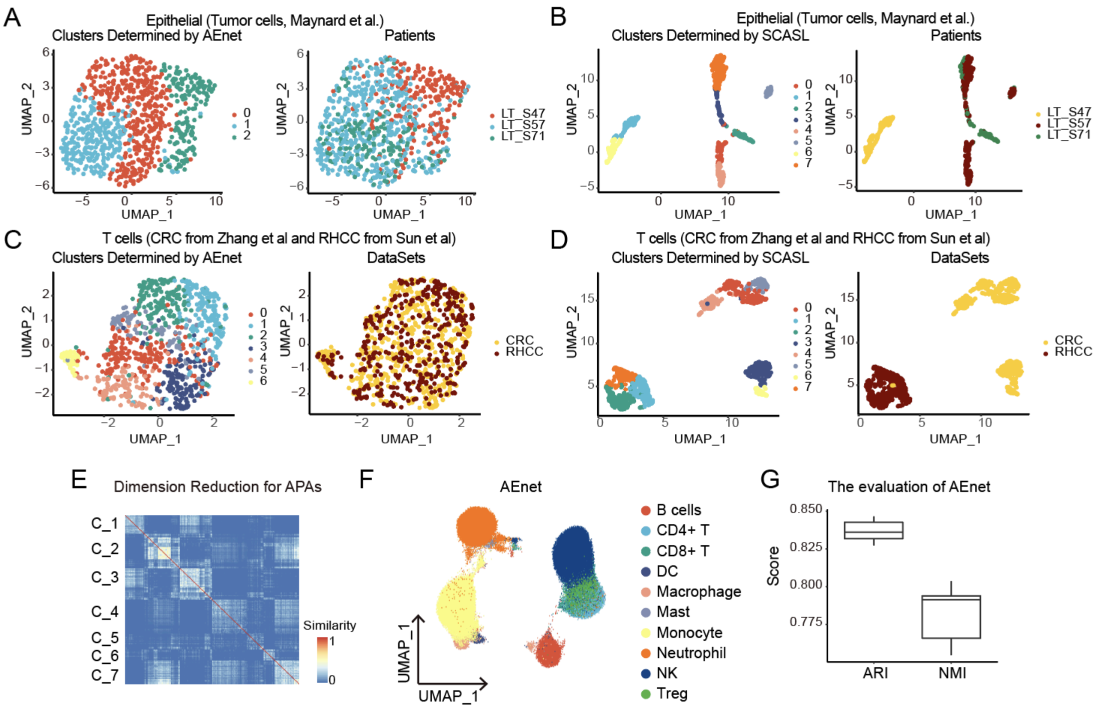

Figure S9. Performance comparison of AEnet and SCASL. A-B. UMAP shows the clustering of cell types determined by AEnet **(A)** and SCASL **(B)** (left panel) and patient clustering (right panel) for epithelial cells from multiple patients. **C-D.** UMAP shows the clustering of cell types determined by AEnet **(C)** and SCASL **(D)** (left panel) and patient clustering (right panel) for T cells from the CRC (Colorectal cancer) and RHCC (Recurrent Hepatocellular carcinoma) datasets. **E.** Heatmap showing APA classes derived from dimensionality reduction using AEnet. **F.** UMAP visualization of cell clustering based on alternative polyadenylation profiles. **G.** Quantitative benchmarking of clustering performance using ARI and NMI, evaluated against the ground truth annotations of the PBMC dataset.

AEnet reduces bias from tumor heterogeneity and identifies key subpopulations and splicing events involved in immunotherapy response.

Due to inherent intra- and inter-tumor heterogeneity, grouping malignant cells solely based on either gene expression profiles or ASPs is challenging. Here, we showcase the power of AEnet in untangling the intricate ASP-EXP relationships using data from 1,286 tumor cells from six lung cancer patients with varying responses to immunotherapy, classified as normal (N), residual disease (RD), and progressive disease (PD) after therapy [33].

Based on the AEnet algorithm, the ASP similarity matrix revealed a distinct separation into six ASP clusters, which resulted in three cell subpopulations (**Fig.**

3A-B). The clusters determined by AEnet represented a transitional therapy-response trajectory from PD (cell subpopulation 2, denoted as S2), to RD (S0), to normal response (S1), progressing from left to right along the UMAP_1 axis (**Fig. 3C**). The marker genes of S1 (N) were enriched in alveolar signatures, including *AQP4*, *SFTPB/C/D*, *NKX2-1*, and *FOXA2 [35,36]*, while S2 (PD) was associated with elevated expression of prothrombin activation genes (*PLAT*, *PLAUR*), gap-junction proteins (*GJB2/3/5*), and the well-known EMT (epithelial-mesenchymal transition) marker *EPCAM* (**Fig. 3D**) [37–39].

From the perspective of ASP clusters, C_4 (ASP cluster 4) was notably co-occurring with S2 while being excluded from S1 (**Fig. 3E**). The top-ranked hub genes in this cluster were IK and CELF2, which exhibited opposite expression trends between normal and PD cells (**Fig. 3F-G**). CELF2 is a crucial splicing factor, and its downregulation has been reported to promote tumor progression in both pancreatic and breast cancers [40,41]. The role of IK remains unclear; however, we found it to be upregulated in a CRC cohort as responses to immunotherapy deteriorated (**Fig. 3H**)[42].

In addition, C_4 was enriched in inflammation-associated pathways, including the response to type II interferon, positive regulation of lymphocyte proliferation, and the adaptive immune system (**Fig. 3I**). Among the key genes related to inflammatory responses, CD74, the HLA-DR antigens-associated invariant chain, is reported to exhibit dual oncogenic and tumor-suppressive roles depending on the cancer type and specific microenvironment. In this lung cancer dataset, we found that ASPs in CD74 were exclusively dominated by the isoforms CD74-201 and CD74-202. CD74-202 is reported as the soluble form, which suppressed melanoma cell growth and induced apoptosis under IFN-γ stimulatory conditions [43,44]. The significantly differentiated ratio of CD74-202 to CD74-201 could directly indicate post-therapy responses in different cell groups (**Fig. 3J**).

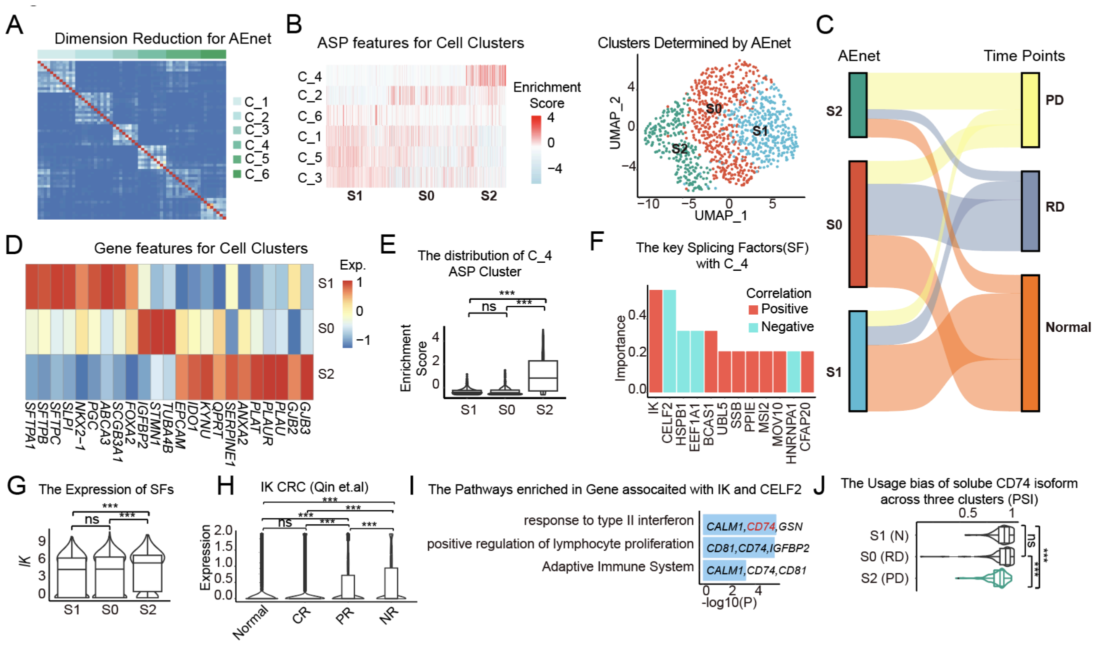

Figure 3. AEnet decreases the bias caused by tumor heterogeneity and uncover mechanistic insights in immunotherapy response. A. The heatmap shows the ASP clusters from dimension reduction of AEnet. **B.** The heatmap displays the enrichment score of ASP clusters across cell clusters determined by AEnet (left panel). UMAP displays clustering of cell types determined by AEnet (right panel). **C.** The Sankey plot shows the overlapping of cells between Time Points and Clusters determined by AEnet. **D.** The heatmap displays the expression of marker genes across cell clusters determined by AEnet. **E.** The distribution of C_4 enrichment scores across cell clusters determined by AEnet. **F.** The barplot displays the importance of splicing factors in the formation of C_4 ASP clusters. The color represents the correlation relationship between splicing factors and C_4 ASP clusters. **G.** The expression of IK across cell clusters. Statistical analysis was performed using the Student's t-test. **H.** The expression of IK within cells at different timepoints. Statistical analysis was performed using the Student's t-test. **I.** The top 3 pathways enriched for the genes with alternative splicing patterns in C_4 ASP clusters. **J.** The alternative splicing patterns of CD74 and the PSI distribution across 3 clusters. Statistical analysis was performed using the Student's t-test. * $p < 0.05$, ** $p < 0.01$, *** $p < 0.001$.

AEnet reveals cellular splicing heterogeneity and its key splicing events in tumor-infiltrating T cells across various cancer types

The design of the AEnet algorithm largely bypasses batch effect issues in scRNA-seq analyses, making it particularly suitable for pan-cancer studies. To explore the

cross-tissue capacity of this 'AS-Expression Network' concept, we curated tumor-infiltrating lymphocyte T cells from four cancer types: liver cancer (HCC) [45], colorectal cancer (CRC) [29], lung cancer (LUAD) [33], and recurrent liver cancer (RHCC) [28].

By selecting the top 1,223 ASP events from a total of 86,616 valid ASP-EXP links (supported by at least 2 samples across a minimum of 2 datasets), we identified 14 ASP clusters (referred to as C1–C14) (**Fig. S10A-C**). We subsequently generated 10 cell subpopulations (referred to as S0–S9) based on distinct compositions of ASP cluster-wise signatures across the 4 T cells datasets (**Fig. 4A and S10D**). Among these populations, S2 and S3 expressed naïve T cell markers (*IL7R, CCR7, LEF1*), S1 was characterized by memory T cell markers (*CD52, ANXA1, CREM*), S0 and S5 exhibited effector T markers (*NKG7, GZMA/B*), S4, S7, S8, and S9 represented the exhausted state of T cells (*PDCD1, CTLA4, HAVCR2*), while S6 corresponded to the proliferative state (*MKI67, TOP2A*) (**Fig. S10E**) [46]. Strikingly, AEnet did not partition T cells into canonical $CD4^+$ and $CD8^+$ subtypes, suggesting that alternative splicing primarily contributes to the transition of cell states rather than defining cell lineage (**Fig. 4B**).

Cross-cluster interactions observed in our data **(Fig. S10C)**. To further explore these relationships, we organized ASP clusters based on pairwise similarity and identified three major regions, each comprising clusters with higher intra-region similarity than inter-region similarity **(Fig. S10F)**. Region 1 includes clusters C_7 and C_9, which exhibit the highest mutual similarity and are both predominantly enriched in naïve T cells. Interestingly, C_7 also shares similarity with C_5 and C_10, while C_9 is more closely related to C_4 and C_11. This suggests that C_7 and C_9 may represent bifurcating points leading to two naïve T cell differentiation trajectories—one toward memory T cells (C_5 and C_10), and the other toward effector T cells (C_4 and C_11) **(Fig. S10H-J)**. Supporting this, C_7 is enriched during the naïve-to-memory transition, while C_9 is enriched along the naïve-to-effector axis **(Fig. S10G)**. Region 2 comprises clusters C_11, C_4, and C_14, which show strong inter-cluster similarity. C_4 acts as a central node, connecting C_11 and C_14, suggesting a potential progression from effector T cells (C_11) to effector memory T cells (C_4), and eventually to exhausted T cells (C_14). Notably, C_11 also shares similarity with C_9, and C_14 with C_1—both associated with exhausted states—indicating a continuous exhaustion trajectory **(Fig. S10H)**. Region 3 implies an alternative exhaustion pathway, involving clusters C_5, C_10, and C_12. Here, C_10 links C_5 (memory/naïve-enriched) and C_12 (exhausted-enriched), forming a sequence akin to that in Region 2. Similarity between C_5 and C_7, and between C_12 and C_1, further supports a parallel differentiation path from naïve/memory T cells to exhaustion **(Fig. S10I)**. In summary, while rigid clustering provides discrete groupings, our similarity-based regional analysis reveals underlying transitions and trajectories among ASP-defined clusters. These findings reinforce the reviewer's

observation and highlight the importance of complementary methods to uncover dynamic, intermediate cellular states that may be overlooked by strict partitioning.

When focusing on ASP clusters, the exclusive deficiency of C_5 in effector T cells (S0 and S5) and C_8 in proliferating T cells (S6) were two notable signatures demonstrating the correlation between specific ASPs and cell population heterogeneity (**Fig. 4C**). Among the top 10 common splicing factors, the C_5 ASP cluster positively correlated with EEF1A1 and PABPC1, which were partially aligned with ASPs primarily occurring in naïve T cells (clusters C_7, C_9, and C_13) (**Fig. 4D-E**). Additionally, C_5 was correlated with HSPA1A/B, splicing factors contributing to exhausted T cells (C_1, C_2, C_3, C_12, and C_14). Effector T cells tend to adopt splicing patterns opposite to those of the C_5 ASP cluster, which suggests C_5 ASP class emerged as a pivotal determinant for effector T cell formation (**Fig. 4C**). We then explored the enriched pathways associated with hub genes in C_5 to identify potential factors that distinguish effector T cells from other T cell types (**Fig. 4F**). Surprisingly, effector T cells favored a previously undefined FYB pattern (referred to as FYB_new), using chr5_39217636 as the end of the first exon of FYB1—a site not documented in the GRCh38.p14 reference genome. This splicing variant is associated with pathways involved in second messenger generation, immune response-regulating signaling, and cell surface receptor signaling, all critical processes for achieving effector cell status (**Fig. 4G-H**). In contrast, FYB1-212 was associated with pathways linked to naïve and memory T cells. Regarding the other ASP cluster, C_8 was negatively correlated with SNRNP25 among the top 10 factors (**Fig. 4E**). Small Nuclear Ribonucleoprotein 25 (SNRNP25) is known for its role in spliceosome assembly and function, facilitating the accurate removal of introns from pre-mRNA and the joining of exons to form mature mRNA[47]. Its upregulation (reflected as a double negative correlation) in proliferating T cells served as a clear marker, identifying this cell type solely through splicing factors **(Fig. S10K)**.

Of particular interest, HNRNPLL, the third-ranked splicing factor (**Fig. 4D**), was highly positively correlated with exhausted T-associated ASP clusters and linked to poor prognosis (**Fig. 4E, 4I**). Genes regulated by HNRNPLL were enriched in thymic T cell selection, axon guidance, and leukocyte activation (**Fig. S10L**). Among the hub genes, CD3D, the most canonical T cell marker, exhibited two isoforms with distinct PSI distributions between exhausted and other T cells (**Fig. S10M**). Exhausted T cells preferentially spliced into isoform CD3D-202, which is linked to the PD-1 signaling pathway, CD28 family-mediated costimulation, and TCR signaling, while other T cells primarily utilized CD3D-201 for differentiation (**Fig. S10N**).

Collectively, we demonstrate the capability of the AEnet algorithm for integrated scRNA-seq data analysis without the need for batch corrections. Our algorithm facilitates bioinformatic data mining for isoform usage preferences, and even the discovery of novel isoforms of functional importance, such as new ASPs in the FYB gene for effector T cells.

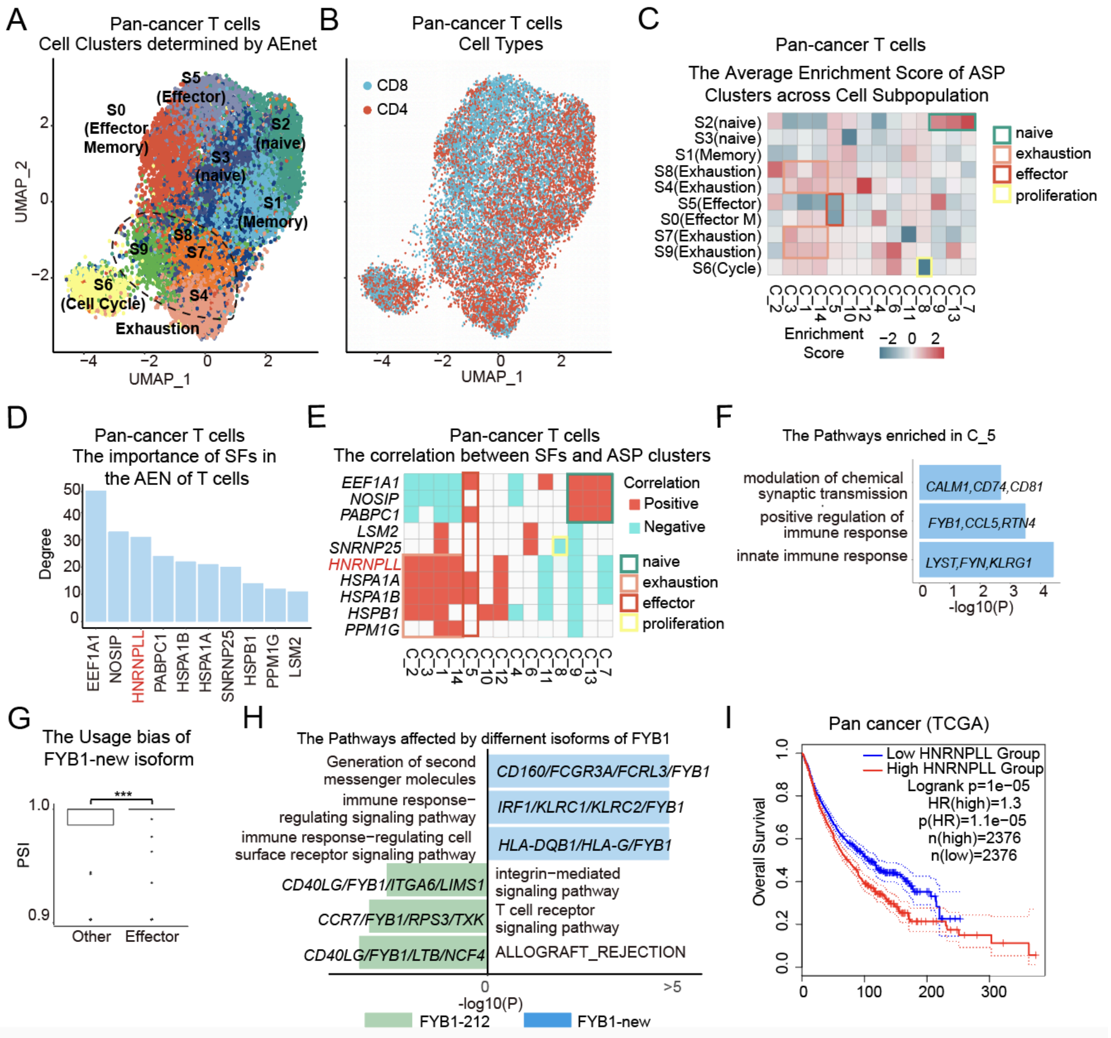

Figure 4. Application of the AEnet to Pan-Cancer Tumor-Infiltrating Lymphocyte T Cell Single-Cell Data. A-B. UMAP displays clustering of cell types determined by AEnet (A) and lineage (B). **C.** The heatmap displays the enrichment score of ASP clusters across cell clusters determined by AS. **D.** The barplot displays the importance of splicing factors in the formation of the AEN network of the pan-cancer T cells. **E.** The relationship between ASP clusters and key splicing factors. **F.** The top 3 pathways enriched for the genes with alternative splicing patterns in C_5 ASP clusters. **G.** The PSI distribution of FYB1-new isoform within FYB1 across effector and other T cells. Statistical analysis was performed using the Student's t-test. **H.** The pathways enriched in the gene sets with different isoforms of FYB1. **I.** Kaplan-Meier analysis shows the overall survival of patients characterized by low (blue) or high (red) HNRNPLL in the TCGA cohort. Statistical analysis was performed using the log-rank test. * $p < 0.05$, ** $p < 0.01$, *** $p < 0.001$.

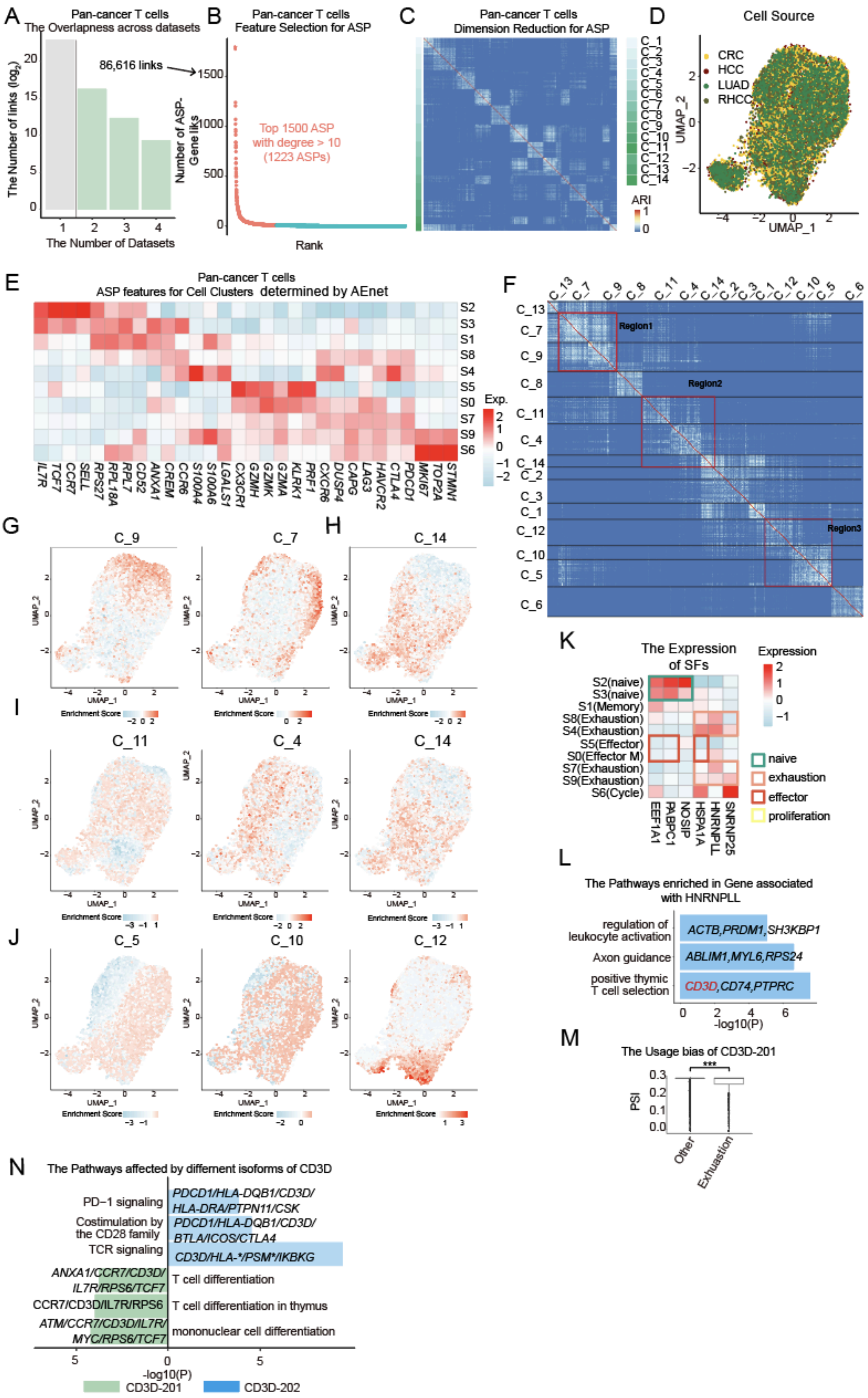
A
Pan-cancer T cells
The Overlapness across datasets
86,616 links
The Number of links (log2)
The Number of Datasets
B
Pan-cancer T cells
Feature Selection for ASP
Top 1500 ASP
with degree > 10
(1223 ASPs)
Number of ASP-Gene liks
Rank
C
Pan-cancer T cells
Dimension Reduction for ASP
ARI
D
Cell Source
CRC
HCC
LUAD
RHCC
UMAP_1
UMAP_2
E
Pan-cancer T cells
ASP features for Cell Clusters determined by AEnet
Exp.
F
Region1
Region2
Region3
G
H
I
J
Enrichment Score
K
The Expression of SFs
Expression
S2(naive)
S3(naive)
S1(Memory)
S8(Exhaustion)
S4(Exhaustion)
S5(Effector)
S0(Effector M)
S7(Exhaustion)
S9(Exhaustion)
S6(Cycle)
EEF1A1
PABPC1
NOSIP
HSPA1A
HNRNPLL
SNRNP25
naive
exhaustion
effector
proliferation
L
The Pathways enriched in Gene associated with HNRNPLL
regulation of leukocyte activation
ACTB,PRDM1,SH3KBP1
Axon guidance
ABLIM1,MYL6,RPS24
positive thymic T cell selection
CD3D,CD74,PTPRC
-log10(P)
M
The Usage bias of CD3D-201
PSI
Other
Exhaustion
N
The Pathways affected by differnent isoforms of CD3D
PD−1 signaling
PDCD1/HLA-DQB1/CD3D/HLA-DRA/PTPN11/CSK
Costimulation by the CD28 family
PDCD1/HLA-DQB1/CD3D/BTLA/ICOS/CTLA4
TCR signaling
CD3D/HLA-*/PSM*/IKBKG
ANXA1/CCR7/CD3D/IL7R/RPS6/TCF7
T cell differentiation
CCR7/CD3D/IL7R/RPS6
T cell differentiation in thymus
ATM/CCR7/CD3D/IL7R/MYC/RPS6/TCF7
mononuclear cell differentiation
-log10(P)
CD3D-201
CD3D-202

Figure S10. AEnet reveals cellular splicing heterogeneity and its key splicing events in tumor-infiltrating T cells across various cancer types. A. The barplot displays the selection of high quality links in the AEnet of Pan-cancer T cells. **B.** The selection of key alternative splicing patterns. **C.** The heatmap shows the ASP cluster from dimension reduction of AEnet. **D.** UMAP displays cell sources. **E.** The heatmap displays the expression of marker genes across cell clusters determined by AS. **F.** Heatmap of ASP clusters ordered by similarity, illustrating inter-cluster relationships within the pan-cancer T cell dataset. **G-J.** Distribution of enrichment scores for selected ASP clusters. **K.** The expression of key splicing factors across clusters determined by alternative splicing. **L.** The pathways enriched in genes with ASP associated with HNRNPLL. **M.** The PSI distribution of CD3D-201 isoform within CD3D within exhaustion and other T cells. Statistical analysis was performed using the Student's t-test. **N.** The pathways enriched in the gene sets with different isoforms of CD3D.

AEnet Uncovers Transitional Cell States and Key Splicing Factors in Embryonic Gastrulation

During embryogenesis, alternative splicing is a key mechanism that fine-tunes developmental pathways and controls cell fate decisions. Here, we apply AEnet to interrogate a scRNA-seq dataset of gastrulation-stage human embryos from the Human Developmental Biology Resource, elucidating how the AS process enables precise regulation of gene expression at this stage. The reference dataset comprises 1,195 cells (665 caudal, 340 rostral, and 190 yolk sac cells), with a median of 4,000 genes detected per cell[48].

Analogous to the previous analysis process, we first identified a total of 1,604 ASP events, 25 ASP clusters, and 11 cell populations (**Fig. 5A and S11A-C**). Notably, the ASPs are assumed to be highly distinct among different cell types during embryogenesis. Therefore AEnet factorized the cell populations in a manner similar to those clustered based solely on RNA profilings (**Fig. 5B**, ARI = 0.304). The sequential differentiation trajectory from epiblast cells (cell subpopulation 5, S5) to the primitive streak (S1), followed by the transition to endodermal cells (S6/S10) or mesoderm (S2/S0), ultimately leading to axial mesoderm (S7), was clearly discernible (**Fig. 5A, right panel**) [49].

Epiblast cells (S5) are classic pluripotent stem cells, derived from the inner cell mass of the blastocyst and capable of differentiating into the three germ layers. For the highly correlated ASP clusters C_13 and C_17, PSIP1 and SNRPN were identified as key regulatory factors that maintain cell stemness (**Fig. 5C-D, 11D-E**). HNRNPAB and SRSF3 were associated with splicing decisions that resulted in longer junctions spanning genomic loci, observed more frequently in epiblast cells than in cells with

reduced stemness (**Fig. 5E**). We verified this intriguing finding in an independent iPSC dataset (**Fig. S11F**)[18].

Some populations defined by AEnet were in a transitional stage. For example, S2 shares RNA profiling similarities with both S1 and S0 (**Fig. 5B**). This cell population expressed relatively lower stemness signatures and higher mesodermal features compared to the primitive streak (S1) (**Fig. 5F**). The enriched pathways also indicated that S2 was an intermediate cell state between the primitive streak (S1) and mesoderm (S0) (**Fig. 5G**). We subsequently compared the ASP cluster compositions between S2 and S0, focusing on two of the most distinct ASP clusters, C_1 and C_18, for downstream analysis (**Fig. 5C**). As part of the routine analysis, we identified the top splicing factors and hub-gene-enriched pathways. SNRPD2, a core component of the spliceosome, emerged as a pivotal factor distinguishing these two mesoderm subtypes (**Fig. 5H and S11G**). Related to the genes in enriched pathways, TNRC6B displayed distinct isoform distributions across cell clusters (**Fig. 5I and S11H**). TNRC6B-205 was enriched in S2 and linked to the classical WNT, Notch, and MAPK signaling pathways. In contrast, TNRC6B-201 was prevalent in other cell types and associated with primary germ layer formation and other processes (**Fig. 5J**).

Moreover, we unveiled that ASP clusters C_2, C_5, C_7, C_16, and C_23 were pivotal for endoderm differentiation, with HSPB1 emerging as a core negative regulator of their formation. Clusters C_12, C_20, and C_24, crucial for yolk sac mesoderm development, were positively regulated by HSPB1 **(Fig. S11I-J)**. Lastly, ASP events in HEP and erythrocyte development (C_3, C_4, C_6, C_8, C_11, C_14, C_22, and C_25) were governed by MBNL1 and HSPA5, both highly expressed in these cell types (**Fig. S11I-J**).

By exploring the alternative splicing landscapes in this human embryonic data (**Fig. 5K**), we not only clarified phenotypic subtleties along the AS-based developmental trajectory but also illuminated the complexity of AS mechanisms underlying cell differentiation and embryonic development, successfully demonstrating the capabilities of the AEnet algorithm.

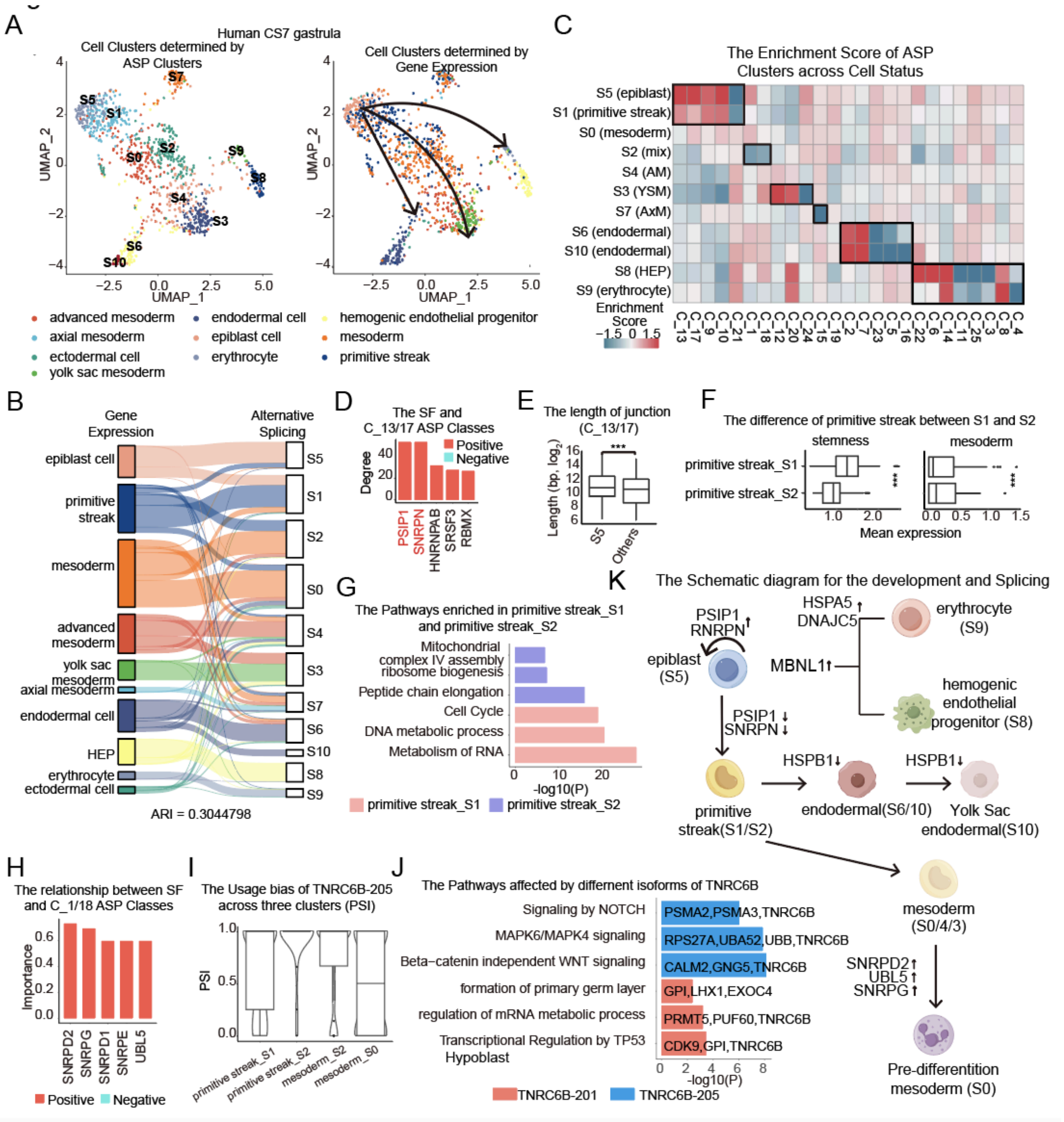

Figure 5. AEnet Uncovers Transitional Cell States and Key Splicing Factors in Embryonic Gastrulation. A. UMAP displays clustering of cells by cell type determined by Alternative Splicing (left panel), and gene expression (right panel). **B.** The overlapping of cells between clusters determined by alternative splicing and gene expression. **C.** The heatmap displays the enrichment score of ASP classes across cell clusters determined by AS. **D.** The barplot displays the importance of splicing factors in the formation of C_13/17 ASP classes. The color indicates the relationship between SF and ASP classes. **E.** The length of junction that differentially usage in different cell types. Statistical analysis was performed using the Student's t-test. **F.** The boxplot displays the expression of stemness and mesoderm markers across primitive streak cell subsets determined by AS and expression. Statistical analysis was performed using the Student's t-test. **G.** The pathways enriched in the upregulated genes in the primitive streak cell subsets determined by AS and expression. **H.** The barplot displays the importance of splicing factors in the formation

of C_1/18 ASP classes. The color indicates the relationship between SF and ASP classes. **I.** The alternative splicing patterns of TNRC6B and the PSI distribution. **J.** The pathways enriched in the gene sets with different isoforms of TNRC6B. **K.** The illustration depicts the developmental trajectory and highlights the top-ranked key splicing factors based on their relative importance. * p < 0.05, ** p < 0.01, *** p < 0.001.

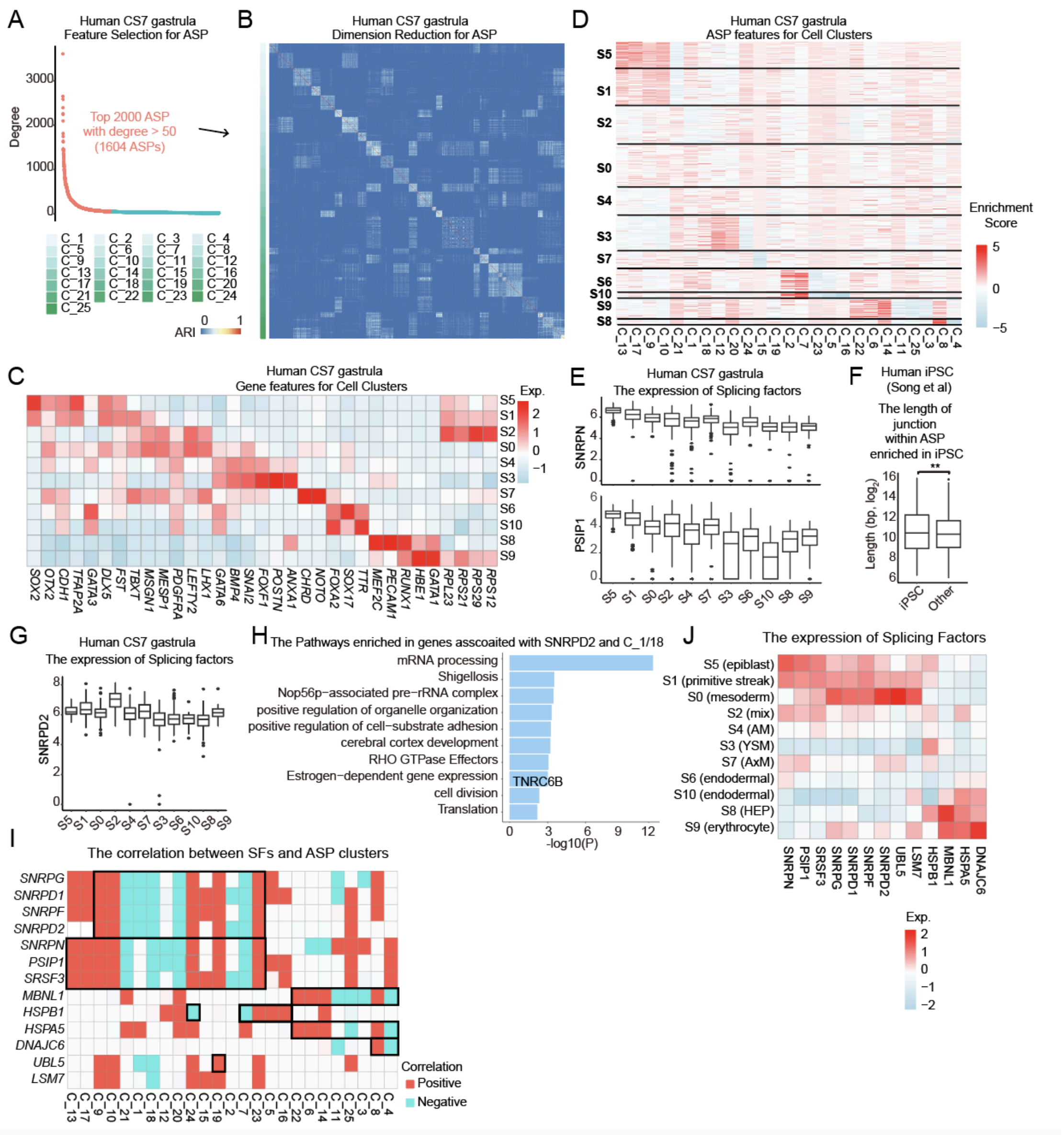

Figure S11. AEnet reveals intermediate cell status during gastrulation. A. The selection of key alternative splicing patterns. **B.** The heatmap shows the ASP classes from dimension reduction of AEnet. **C.** The heatmap displays the expression of developmental markers across cell clusters determined by AS. **D.** The heatmap displays the enrichment score of ASP classes across cell clusters determined by AS. **E.** The expression of SNRPN and PSIP1 across the cell clusters determined by AS. **F.** The length of junction that differentially usage in different cell types from Song et

al. Statistical analysis was performed using the Student's t-test. **G.** The expression of SNRPD2 across the cell clusters determined by AS. **H.** The pathways enriched in the genes within C_1 and C_18, which shows correlation with the expression of SNRPD2. **I-J.** The heatmap displays the relationship between ASP classes and key splicing factors (I), as well as the expression of key splicing factor **(J)**. * $p < 0.05$, ** $p < 0.01$, *** $p < 0.001$.

Discussion

The discovery of cellular heterogeneity can be enhanced by multiple modalities derived from single-cell genomics technologies. For example, SHARE-seq provides insights into cell identity by jointly detecting gene expression and chromatin regulation [50], while CITE-seq simultaneously studies the transcriptome and protein expression, accounting for post-transcriptional and translational modifications to enable fine-grained detection of cell populations [51]. However, generating such data remains laboratory-intensive, involving complex, multi-step workflows that require careful optimization and the use of specialized reagents and equipment. In fact, alternative splicing events represent another inherent modality that is often present in common scRNA-seq data [52,53]. These events can be cost-efficiently detected using plate-based platforms like Fluidigm C1 and Smart-seq [54,55], or droplet-based platforms with higher sequencing depth [22,56,57].

The introduction of AEnet represents a groundbreaking attempt to integrate single-cell alternative splicing dynamics and gene expression profiling into a network to explore cellular heterogeneity, accounting for post-transcriptional regulatory mechanisms. By applying AEnet to three distinct single-cell datasets, we found that the interplay between AS and gene expression is dynamic when generating cell populations. For tumor patient samples with varying immunotherapy responses and pan-cancer T cell analysis, the integrated clustering results reveal distinct cell states within the RNA-based subtypes. In contrast, during cell differentiation in embryonic gastrulation, the clustering results more closely resemble the major cell types. This suggests that AS events contribute differently across tissues and conditions in distinguishing cell clusters, with a particularly important role during the fine-grained clustering process. Therefore, previous methods that first rely on RNA-based clustering and then apply AS-based differential analysis as a separate downstream step are not optimal for capturing cellular heterogeneity using AS events.

One of the core advantages of AEnet lies in its ability to "modularize" complex gene expression data by grouping AS patterns, thereby minimizing noise from RNA expression profiling, which is often referred to as batch effects in scRNA-seq analysis. This modular approach also mitigates AS-based biases related to missing values, tumor heterogeneity, and patient-specific factors, while enabling the

identification of novel cell subpopulations and intermediate states that may remain obscured using traditional methods.

To note, AEnet is designed primarily for unsupervised discovery of splicing heterogeneity and regulatory mechanisms within a single biological context (e.g., a specific sample or condition), rather than for direct differential splicing comparisons across clusters or conditions (e.g., disease vs. healthy states). Its strengths lie in integrating alternative splicing and gene expression to characterize intra-condition splicing programs, identify regulatory modules, and predict affected pathways—capabilities enabled by its modular approach to minimize noise and capture context-enriched features. Notably, AEnet can also predict key regulatory components such as splicing factors (SFs) that drive observed splicing patterns, providing mechanistic insights into post-transcriptional regulation. For users seeking group-wise differential splicing analysis, AEnet's outputs (e.g., anchor ASPs and regulatory modules) can be integrated with dedicated tools like MARVEL or MAJIQ-SC, which specialize in statistical comparisons across predefined groups. A detailed functionality comparison of these methods is provided in Supplemental Table 1, aiding users in selecting tools aligned with their research objectives.

With the progression of sequencing technologies, particularly the advent of long-read sequencing, alternative splicing events can be identified more conveniently and accurately [14,58]. This advancement will significantly broaden the use of our method as a conventional analysis tool. However, even with well-designed sequencing, barcode errors in current single-cell long-read sequencing still significantly affect data quality, leading to the misassignment of AS events to unrelated cells [59]. We look forward to the rapid development of high-precision, high-throughput long-read sequencing technologies to advance research in the single-cell AS field and the development of our software in the near future.

Methods

Dataset preprocessing prior to AEnet for alternative splicing

Dataset preprocessing prior to AEN involved a series of essential steps aimed at constructing the cell-junction count matrix and the annotation file for junctions, by the established DESJ-detection pipeline [19]. Initially, alignment software such as STAR was employed to obtain the coverage information of junctions in each cell [60]. To ensure the reliability of the data, the junctions with a minimum of Rm reads in Cellm cells were retained, with default values set as Cellm = 10 and Rm = 4. Additionally, all junctions were annotated to determine their primary gene sources. Specifically, we selectively retained junctions that were exclusively associated with a single gene to ensure accuracy of the alignment. Furthermore, a count matrix was generated, reflecting the read numbers of junctions in each cell. Subsequently, the count matrix was normalized through dividing the counts by the unique mapped reads number for each cell, resulting in the generation of the CPM (counts per million) matrix. The diligent execution of these preprocessing steps ensured the integrity and reliability of the dataset, allowing for robust analysis within the AEN framework.

Detailed procedure for AEnet

The AEnet method consists of multiple main steps: ASP identification and quantification, AEN network and multiple samples integration, anchor ASP identification, ASP clusters identification, cell clustering, and regulatory mechanism prediction. In this section, we will introduce each step in detail.

ASP Identification and Quantitative

Exon-exon junctions with the same starting point or endpoint were defined as alternative splicing patterns (ASP). The percent spliced In (PSI) value of the ASPs in cells was measured by comparing the coverage of one junction to the two junctions derived from the ASP. Subsequently, we integrated all cells and all ASPs into a cell-ASP PSI matrix.

AEN Network and multiple samples integration

We integrated the alternative splicing event scoring matrix and gene expression matrix of all cells in each sample, to infer the potential relationship between ASPs and gene expression. For each sample, AEnet calculated the significance of the correlation between ASP PSI values and gene expression using Spearman's rank correlation. Significant ASP PSI - Gene Expression links ($p < 0.01$) were then used to construct an alternative splicing-gene expression association network (AEN) for each sample. The edge width of the links in the network was determined using the

Spearman correlation coefficient, which indicates both the strength and the direction (positive or negative) of the correlation. To ensure robustness, we extracted ASP-EXP links that exhibited consistent trends across multiple samples (defined as more than two samples). These links were then integrated into a multi-sample AEN network. In this pan-sample AEN network, the width of the edges corresponds to the number of samples supporting each ASP-EXP link, while the color indicates the direction of the correlation (positive or negative). This approach effectively mitigated batch effects and strengthened the reliability of the analysis.

Anchor ASPs/Genes identification

We calculated the degree strength of the ASP in the AEN network and selected the top ASPs based on their ranking. The degree of ASPs was determined by the number of links to the ASP in the AEN network. A higher degree indicates a stronger association between the ASP and gene expression dynamics in the dataset. The top 1,500 ASPs (by default) were selected for downstream analyses, provided that each ASP was associated with more than 15 positive and 15 negative links. To reduce data complexity and extract key information, we calculate the Jaccard metric of ASP-EXP links (where genes linked to the ASP are treated as sets) to represent their similarity for each pair of ASPs, thereby constructing a similarity matrix of anchor ASPs. Analogously, anchor genes were defined as those associated with ASPs from more than 30 distinct genes. For these anchor genes, a similar Jaccard similarity metric was calculated based on their associated ASPs, enabling the construction of a gene-level similarity matrix.

ASP/Gene clusters identification

Using hierarchical clustering methods, we divided the ASPs into ASP clusters according to their similarity, ensuring that ASPs in the same clusters were associated with similar gene expression sets. The number of clusters is defaultly set as 25. Clusters are filtered by ensuring that at least 10% of ASP pairs within each cluster exhibit a similarity score higher than 0.1. Clusters with fewer than 10 ASPs are merged with the most similar clusters, provided that the similarity between clusters is above 0.1. The similarity between two ASP clusters is defined as the proportion of ASP pairs with a similarity score greater than 0.1, where one ASP in the pair belongs to one cluster and the other ASP belongs to the other cluster. An analogous clustering procedure was applied to anchor genes to identify gene clusters based on shared ASP associations.

Cell Clustering Analysis

To further understand the cell heterogeneity at the level of alternative splicing, we performed cell clustering analysis based on alternative splicing and gene expression.

Specifically, we first calculated the enrichment scores of each ASP cluster and each gene cluster for individual cells, resulting in a cell-by-cluster enrichment score matrix. The enrichment score of an ASP cluster in a given cell was defined as the average PSI (Percent Spliced-In) value of all ASPs within that cluster. Similarly, the enrichment score of a gene cluster in a given cell was defined as the average expression level of all genes in that cluster. Next, the cell-ASP and Gene cluster enrichment score matrix was normalized using the scale function. Finally, the normalized matrix was used as input for the FindNeighbors and FindClusters functions of Seurat to detect cell clusters [61,62]. Notably, cell clustering can be performed based on either cell–ASP cluster enrichment score matrix or cell–gene cluster matrix alone. Finally, cell subpopulations were delineated using a dual-modality approach that integrates alternative splicing patterns (ASPs) with gene expression data.

Regulatory Mechanism Inference

To identify key splicing factors, we integrated the AEN network with a predefined list of splicing factors and a specific set of ASP events [63]. This allowed us to construct a sub-network of ASP-splicing factors, facilitating the exploration of the regulatory relationships between alternative splicing events and splicing factors. The higher the degree strength of the splicing factor, the more it indicated a close relationship with the occurrence of these ASP events. The regulatory direction of splicing factors on the given set of ASPs was determined based on the proportion of positive or negative correlations. Specifically, if 75% or more of the splicing factor–ASP pairs were positive (or negative), the splicing factor was classified as positively (or negatively) regulating the ASP sets.

We also identified key pathways associated with different ASP patterns. For each ASP event, gene sets that were positively and negatively correlated with the event were identified based on the AEN network. Functional enrichment analysis was then performed on both the ASP' gene and the positively (or negatively) correlated gene sets. The pathway to which the ASP gene belongs is considered the most likely pathway affected by the ASP change.

Performance evaluation of AEnet

To evaluate AEnet's performance in inferring ASP patterns in comparison with MARVEL—the currently most comprehensive method—we applied a demo dataset of MARVEL, comprising induced pluripotent stem cells (iPSCs) and iPSC-derived endoderm cells [30]. ASP patterns were identified using the asp function of AEnet, while MARVEL was run with default parameters to detect ASP patterns for comparison. To systematically evaluate the impact of count thresholds, we tested a range of minimum read count cutoffs: 0, 3, 5, 7, and 9. In parallel, to assess potential

biases introduced by filtering against rare but biologically relevant splicing events, we stratified all ASPs into five categories based on the number of supporting cells: Invalid: ≤20 cells (excluded); Type 1: >20–30 cells; Type 2: >30–40 cells; Type 3: >40–50 cells; Type 4: >50 cells. Subsequent analyses evaluated the influence of each ASP type on link robustness and biological relevance.

To evaluate AEnet's ability to identify ASP clusters, a simulated ASP similarity matrix was generated, with ASPs represented along both rows and columns, and similarity values (measured by the Jaccard index) as matrix elements. To assess robustness under varying noise conditions, different types of noise were introduced: uniform noise via the noiseInjector.unif function from the GROAN package, Gaussian noise using the rtruncnorm function, and Poisson noise using the poisson_discrete function from the truncnorm package. The number of ground-truth ASP clusters was set to three. AEnet's clustering performance was quantified by a consistency score, defined as the Jaccard index between the predicted ASP clusters and the simulated ground-truth clusters.

Benchmarking AEnet against other methods

We next focused on evaluating the clustering performance of AEnet using published datasets with established cell type annotations as ground truth, employing Adjusted Rand Index (ARI) and Normalized Mutual Information (NMI) as evaluation metrics. Three independent datasets—iPSC[18], HCC[28], and T cell datasets[29]—were used for benchmarking, with detailed information provided in **Supplementary Table S3**. To assess the contribution of different data modalities, we conducted ablation analyses by providing AEnet with either the expression matrix alone, the ASP PSI matrix alone, or both. These inputs were used to construct the full ASP-EXP network, as well as ASP-only and EXP-only networks, followed by cell clustering based on each configuration.

To compare the performance of AEnet with existing splicing-based clustering methods, we evaluated SCASL and scQuint across three benchmark datasets. The cell–junction count matrix was used as input for both SCASL and scQuint, with all methods run using their respective default parameters. Notably, scQuint was used solely for low-dimensional embedding, whereas SCASL provided both embeddings and clustering outputs. To further assess AEnet's robustness to batch effects, we analyzed data from three patients in the lung cancer dataset and four patients from the CRC and RHCC cohorts. We then examined the distribution of cells in the low-dimensional embedding space to evaluate the extent of batch mixing.

To further demonstrate the versatility of AEnet, we applied it to a widely used 10X Genomics peripheral blood mononuclear cell (PBMC) dataset [34]. Due to the limited sequencing depth of 10X data, AEnet was unable to detect a sufficient number of alternative splicing (AS) events for downstream analysis. Therefore, we focused

instead on alternative polyadenylation (APA) events, which also reflect isoform-level regulatory dynamics. APA usage was quantified using the scAPAtrap [64] and movAPA [65] package, and the resulting cell-level Relative Usage of Distal polyadenylation sites (RUD) values, together with gene expression data, were used as input to AEnet with default parameters to evaluate its performance on 10X datasets.

The lung cancer cells data processing

We downloaded the scRNA-seq raw reads of the human lung cancer dataset from the NCBI database under accession code PRJNA591860[33]. This dataset contained 1,286 normal and cancer cells from six patients before initiating systemic targeted therapy (TKI naive [TN]), at the residual disease (RD) state, which includes samples taken at any time during treatment with targeted therapy while the tumor was regressing or stable by clinical imaging (RD), and upon subsequent progressive disease as determined by clinical imaging, at which point the tumors showed acquired drug resistance (progression [PD]). The human genome (version GRCH38) was used as the reference genome for alignment with STAR (v2.5.3)[60]. We used an existing pipeline to create the junction count matrix. We first merged all the output of the SJ.out.tab files from the STAR aligner. Next, we conducted the dataset pre-processing prior to the AEnet step, described as before. Finally, We get the cell-junction CPM matrix, and the junction annotation files for the dataset.

Then, we used the outcome from the above step as the input to the AEnet pipeline. Firstly, the CPM matrix was input to the asp function of AEnet with default parameters, and the ASPs for each gene is identified. Secondly, the correlation network between ASPs and Gene is constructed using the asp function with default parameters based on the expression matrix and the junction CPM matrix. Next, we further refined the AEN network by retaining only the connection pairs with at least 4 samples supported, using the merge_cor function with default parameters. Additionally, the ASPs with positive or negative links all more than 20 are retained and 70 key ASPs were identified. These key ASPs were then clustered with hierarchy clustering, leading to the identification of 6 ASP clusters based on their similarity, using the junction_clustering function with default parameters. Subsequently, we calculated the enrichment score of each ASP cluster for the cells and performed cluster analysis using the enrichment score matrix of the cell-ASP clusters, categorizing the cells into 3 groups using asp_score and cell_clus function with resolution as 0.2. To further identify the key splicing factors involved in the formation of the C_4 ASP classes, we used the key_sf function to detect the key SF. Additionally, we also group cells into different populations based on gene expression. The gene count matrix was normalized using log1p normalization. Next, the top 3000 highly variable genes were selected to perform principal component analysis. Subsequently, 20 dimensions of principal components were used to perform Louvain

clustering and Uniform Manifold Approximation and Projection (UMAP)-based visualization.

The SCASL package was applied to the junction count matrix of tumor cells to perform cell clustering, using default parameters for filtering, normalization, imputation, and clustering [31]. The Seurat [61,62] package was applied to the expression matrix of tumor cells to perform normalization, dimensionality reduction and clustering: (1) the data was normalized using the “LogNormalize” function; (2) the top 2000 highly variable genes were detected with the “FindVariableFeatures” function and selected, the batch effects across different samples were corrected by the “FindIntegrationAnchors” and “IntegrateData” functions; (3) a KNN-based graph in the 20 PCA space was constructed and refined by cell-cell weights using the “FindNeighbors” function; (4) representative results from graph-based clustering were obtained using the “FindClusters” function with a resolution of 0.3; (5) the top 20 PCAs were used to perform UMAP for visualization of the cells.

The pan-cancer T cells data data processing

We downloaded the scRNA-seq raw reads of human T cells in the fasta format from the EGD database (EGAS00001002072 for HCC, EGAS00001002791 for CRC), NCBI database (PRJNA591860 for LUAD), and CNSA database (CNP0000650 for RHCC). The corresponding gene expression matrix was downloaded from the GEO database (GSE98638 for HCC, GSE108989 for CRC) and CNSA database (CNP0000650 for RHCC). The human genome (version GRCH38) was used as the reference genome for alignment with STAR (v2.5.3). We used an existing pipeline to create the junction count matrix. We first merged all the output of the SJ.out.tab files from the STAR aligner. Next, we conducted the dataset pre-processing prior to the AEnet step, described as before. Finally, We get the cell-junction CPM matrix, and the junction annotation files for each dataset.

Then, we used the outcome from the above step as the input to the AEnet pipeline. Firstly, the CPM matrix was input to the asp function of AEnet with default parameters, and the ASPs for each gene is identified for each dataset. Secondly, the correlation network between ASPs and Gene is constructed using the asp function with default parameters based on the expression matrix and the junction CPM matrix for each dataset. Next, we further refined the AEN network by retaining only the connection pairs with at least 2 samples supported for each dataset, using the merge_cor function with default parameters. Additionally, The ASP-EXP links supported by at least 2 datasets were retained for the downstream analysis. As a result, 1,223 key ASPs were identified based on their ranking and absolute number of degrees (top 2,000 with a degree number greater than 10). These key ASPs were then clustered, leading to the identification of 14 ASP clusters based on the similarity of their associated phenotypes through junction_clustering function with default

parameters. Subsequently, we calculated the enrichment score of each ASP cluster for the cells of the four datasets and performed cluster analysis using the enrichment score matrix of the cell-ASP clusters, categorizing the cells into 10 groups using cell_clus function with resolution as 0.9. To further identify the key splicing factors involved in the formation of the 14 ASP clusters, we used the key_sf function to detect the key SF for each ASP cluster. AEnet was also used to detect key gene sets affected by ASPs from the same gene. These gene sets were then used to identify enriched pathways associated with different alternative splicing patterns of the same gene, such as CD3D and FYB1. The SCASL package was applied to the junction count matrix of T cells in multiple datasets to perform cell clustering, using default parameters for filtering, normalization, imputation, and clustering [31].

The gastrulating human embryo cells data processing

We downloaded the scRNA-seq raw reads of the human embryo dataset from ArrayExpress under accession code E-MTAB-9388. The processed data also was downloaded from http://www.human-gastrula.ne. This dataset contained 1195 cells of the human embryo at embryonic day 16 assigned into 10 clusters. The human genome (version GRCH38) was used as the reference genome for alignment with STAR (v2.5.3). We used an existing pipeline to create the junction count matrix. We first merged all the output of the SJ.out.tab files from the STAR aligner. Next, we conducted the dataset pre-processing prior to the AEnet step, described as before. Finally, We get the cell-junction CPM matrix, and the junction annotation files for the dataset.

Then, we used the outcome from the above step as the input to the AEnet pipeline. Firstly, the CPM matrix was input to the asp function of AEnet with default parameters, and the ASPs for each gene is identified. Secondly, the correlation network between ASPs and Gene is constructed using the asp function with default parameters based on the expression matrix and the junction CPM matrix. Next, we further refined the AEN network by retaining only the connection pairs with p-values less than 1e-4. As a result, 1,604 key ASPs were identified based on their ranking and absolute number of degrees (top 2,000 with a degree number greater than 50). These key ASPs were then clustered, leading to the identification of 25 ASP clusters based on the similarity of their associated phenotypes. Subsequently, we calculated the enrichment of each ASP class within the cells and performed cluster analysis using the enrichment score matrix of the cell-ASP classes, categorizing the cells into 11 groups using the cell_clus function with resolution as 0.1. To further identify the key splicing factors involved in the formation of the 25 ASP classes, we used the key_sf function to detect the key SF for each ASP class. AEnet was also used to detect key gene sets affected by ASPs from the same gene. These gene sets were then used to identify enriched pathways associated with different alternative splicing patterns of the same gene, such as TNRC6B.

Pathway enrichment analysis

Metascape (http://metascape.org) was used to characterize the biological functions of the DEGs of cells in different status[66]. The differentially expressed genes between different cell types were uploaded into the Metascape for the pathway analysis with default setting.

Survival Analysis

GEPIA2 (http://gepia2.cancer-pku.cn/#survival) was used to detect the survival status of HNRNPLL[67]. HNRNPLL was uploaded into the GEPIA2 and the reference datasets were the whole TCGA datasets.

Acknowledgements

We sincerely thank the support provided by China National GeneBank. This study was supported by GuangDong Basic and Applied Basic Research Foundation (2021A1515110832), Shenzhen Key Laboratory of Single-Cell Omics (ZDSYS20190902093613831), Shenzhen Science and Technology Program (LCYX20220620105200001), the National Key R&D Program of China (2021YFC2501900), and Shenzhen Science and Technology Program (JCYJ20240813150001003).

Data availability

The gastrulating human embryo dataset is available in the ArrayExpress database under accession number E-MTAB-938824. scRNA-seq raw reads of human T cells in fastq format from the EGD database (EGAS00001002072 for HCC, EGAS00001002791 for CRC), NCBI database (PRJNA591860 for LUAD), and CNSA databse (CNP0000650 for RHCC). The corresponding gene expression matrix was downloaded from the GEO database (GSE98638 for HCC, GSE108989 for CRC) and CNSA database (CNP0000650 for RHCC).

Code availability

AEnet is written in R. All codes to reproduce figures presented in this paper will be publicly available through GitHub (https://github.com/liushang17/AEN) and the corresponding DOI is as follows: https://doi.org/10.5281/zenodo.14842870.

Author contributions

S.L., X.C, Y.B., L.W. and S.L. conceived the study, designed and performed research, contributed new analytical tools, analyzed data and wrote the manuscript. S.L. also developed the software, performed experiments and developed the metrics. X.C, X.H., Y.B., Y.W., W.H., P.Q., R.L., W.P., and X.Z. discussed the results and contributed to the writing. X.C, helped procure and interpret the datasets. L.W., Y.B., X.C, and S.L. supervised research and contributed to the writing. L.W. supervised the research and the entire project.

Competing interests

The authors declare no competing interests.

Declaration of generative AI and AI-assisted technologies in the writing process

During the preparation of this work, the authors used ChatGPT 4.0 (GPT-4) in order to facilitate the process of proofreading the contents of the draft[68]. After using this tool/service, the authors reviewed and edited the content as needed and took full responsibility for the content of the publication.

Reference

1. Liu Y, Gonzàlez-Porta M, Santos S, Brazma A, Marioni JC, Aebersold R, et al.. Impact of alternative splicing on the human proteome. *Cell Rep*. 20:1229–412017;

2. Öther-Gee Pohl S, Myant KB. Alternative RNA splicing in tumour heterogeneity, plasticity and therapy. *Dis Model Mech*. The Company of Biologists; 2022; doi: 10.1242/dmm.049233.

3. Wright CJ, Smith CWJ, Jiggins CD. Alternative splicing as a source of phenotypic diversity. *Nat Rev Genet*. Springer Science and Business Media LLC; 23:697–7102022;

4. Suzuki H, Aoki Y, Kameyama T, Saito T, Masuda S, Tanihata J, et al.. Endogenous multiple Exon skipping and back-splicing at the DMD mutation hotspot. *Int J Mol Sci*. MDPI AG; 17:17222016;

5. Raj B, Blencowe BJ. Alternative splicing in the mammalian nervous system: Recent insights into mechanisms and functional roles. *Neuron*. Elsevier BV; 87:14–272015;

6. Bhadra M, Howell P, Dutta S, Heintz C, Mair WB. Alternative splicing in aging and longevity. *Hum Genet*. Springer Science and Business Media LLC; 139:357–692020;

7. Martinez NM, Lynch KW. Control of alternative splicing in immune responses: many regulators, many predictions, much still to learn. *Immunol Rev*. Wiley; 253:216–362013;

8. Martín E, Vivori C, Rogalska M, Herrero-Vicente J, Valcárcel J. Alternative splicing regulation of cell-cycle genes by SPF45/SR140/CHERP complex controls cell proliferation. *RNA*. Cold Spring Harbor Laboratory; 27:1557–762021;

9. Braeutigam C, Rago L, Rolke A, Waldmeier L, Christofori G, Winter J. The RNA-binding protein Rbfox2: an essential regulator of EMT-driven alternative splicing and a mediator of cellular invasion. *Oncogene*. Springer Science and Business Media LLC; 33:1082–922014;

10. Jbara A, Lin K-T, Stossel C, Siegfried Z, Shqerat H, Amar-Schwartz A, et al.. RBFOX2 modulates a metastatic signature of alternative splicing in pancreatic cancer. *Nature*. Springer Science and Business Media LLC; 617:147–532023;

11. Olivieri JE, Dehghannasiri R, Salzman J. The SpliZ generalizes “percent spliced in” to reveal regulated splicing at single-cell resolution. *Nat Methods*. Springer Science and Business Media LLC; 19:307–102022;

12. Li Z, Zhang B, Chan JJ, Tabatabaeian H, Tong QY, Chew XH, et al.. An isoform-resolution transcriptomic atlas of colorectal cancer from long-read single-cell sequencing. *Cell Genom*. Elsevier BV; 4:1006412024;

13. Huang Y, Guo J, Han X, Zhao Y, Li X, Xing P, et al.. Splicing diversity enhances the molecular classification of pituitary neuroendocrine tumors. *Nat Commun*. Springer Science and Business Media LLC; 16:15522025;

14. Joglekar A, Hu W, Zhang B, Narykov O, Diekhans M, Marrocco J, et al.. Single-cell long-read sequencing-based mapping reveals specialized splicing patterns in developing and adult mouse and human brain. *Nat Neurosci*. Springer Science and Business Media LLC; 27:1051–632024;

15. Lukacsovich D, Winterer J, Que L, Luo W, Lukacsovich T, Földy C. Single-cell RNA-seq reveals developmental origins and ontogenetic stability of neurexin alternative splicing profiles. *Cell Rep*. Elsevier BV; 27:3752–9.e42019;

16. Shalek AK, Satija R, Adiconis X, Gertner RS, Gaublomme JT, Raychowdhury R, et al.. Single-cell transcriptomics reveals bimodality in expression and splicing in immune cells. *Nature*. Springer Science and Business Media LLC; 498:236–402013;

17. Huang Y, Sanguinetti G. BRIE: transcriptome-wide splicing quantification in single cells. *Genome Biol*. Springer Science and Business Media LLC; 2017; doi: 10.1186/s13059-017-1248-5.

18. Song Y, Botvinnik OB, Lovci MT, Kakaradov B, Liu P, Xu JL, et al.. Single-cell alternative splicing analysis with expedition reveals splicing dynamics during neuron differentiation. *Mol Cell*. Elsevier BV; 67:148–61.e52017;

19. Liu S, Zhou B, Wu L, Sun Y, Chen J, Liu S. Single-cell differential splicing analysis reveals high heterogeneity of liver tumor-infiltrating T cells. *Sci Rep*.

Springer Science and Business Media LLC; 11:53252021;

20. Wen WX, Mead AJ, Thongjuea S. MARVEL: an integrated alternative splicing analysis platform for single-cell RNA sequencing data. *Nucleic Acids Res*. Oxford University Press (OUP); 51:e292023;

21. Zhang Q, Ai Y, Abdel-Wahab O. Molecular impact of mutations in RNA splicing factors in cancer. *Mol Cell*. Elsevier BV; 84:3667–802024;

22. Capitanchik C, Wilkins OG, Wagner N, Gagneur J, Ule J. From computational models of the splicing code to regulatory mechanisms and therapeutic implications. *Nat Rev Genet*. 2024; doi: 10.1038/s41576-024-00774-2.

23. Risso D, Perraudeau F, Gribkova S, Dudoit S, Vert J-P. A general and flexible method for signal extraction from single-cell RNA-seq data. *Nat Commun*. 9:2842018;

24. Tran HTN, Ang KS, Chevrier M, Zhang X, Lee NYS, Goh M, et al.. A benchmark of batch-effect correction methods for single-cell RNA sequencing data. *Genome Biol*. Springer Science and Business Media LLC; 21:122020;

25. Wan Y, Larson DR. Splicing heterogeneity: separating signal from noise. *Genome Biol*. 19:862018;

26. Dvinge H, Kim E, Abdel-Wahab O, Bradley RK. RNA splicing factors as oncoproteins and tumour suppressors. *Nat Rev Cancer*. Springer Science and Business Media LLC; 16:413–302016;

27. Bradley RK, Anczuków O. RNA splicing dysregulation and the hallmarks of cancer. *Nat Rev Cancer*. 23:135–552023;

28. Sun Y, Wu L, Zhong Y, Zhou K, Hou Y, Wang Z, et al.. Single-cell landscape of the ecosystem in early-relapse hepatocellular carcinoma. *Cell*. Elsevier BV; 184:404–21.e162021;

29. Zhang L, Yu X, Zheng L, Zhang Y, Li Y, Fang Q, et al.. Lineage tracking reveals dynamic relationships of T cells in colorectal cancer. *Nature*. Springer Science and Business Media LLC; 564:268–722018;

30. Linker SM, Urban L, Clark SJ, Chhatriwala M, Amatya S, McCarthy DJ, et al.. Combined single-cell profiling of expression and DNA methylation reveals splicing regulation and heterogeneity. *Genome Biol*. Springer Science and Business Media LLC; 20:302019;

31. Xiang X, He Y, Zhang Z, Yang X. Interrogations of single-cell RNA splicing landscapes with SCASL define new cell identities with physiological relevance. *Nat Commun*. Springer Science and Business Media LLC; 2024; doi: 10.1038/s41467-024-46480-9.

32. Benegas G, Fischer J, Song YS. Robust and annotation-free analysis of alternative splicing across diverse cell types in mice. *Elife*. eLife Sciences Publications, Ltd; 2022; doi: 10.7554/eLife.73520.

33. Maynard A, McCoach CE, Rotow JK, Harris L, Haderk F, Kerr DL, et al.. Therapy-induced evolution of human lung cancer revealed by single-cell RNA

sequencing. *Cell*. Elsevier BV; 182:1232–51.e222020;

34. Xue R, Zhang Q, Cao Q, Kong R, Xiang X, Liu H, et al.. Liver tumour immune microenvironment subtypes and neutrophil heterogeneity. *Nature*. Springer Science and Business Media LLC; 612:141–72022;

35. Travaglini KJ, Nabhan AN, Penland L, Sinha R, Gillich A, Sit RV, et al.. A molecular cell atlas of the human lung from single-cell RNA sequencing. *Nature*. Springer Science and Business Media LLC; 587:619–252020;

36. Sikkema L, Ramírez-Suástegui C, Strobl DC, Gillett TE, Zappia L, Madissoon E, et al.. An integrated cell atlas of the lung in health and disease. *Nat Med*. 29:1563–772023;

37. Yang J, Antin P, Berx G, Blanpain C, Brabletz T, Bronner M, et al.. Guidelines and definitions for research on epithelial-mesenchymal transition. *Nat Rev Mol Cell Biol*. Springer Science and Business Media LLC; 21:341–522020;

38. Beyer EC, Berthoud VM. Gap junction gene and protein families: Connexins, innexins, and pannexins. *Biochim Biophys Acta Biomembr*. 1860:5–82018;

39. Narayanaswamy PB, Baral TK, Haller H, Dumler I, Acharya K, Kiyan Y. Transcriptomic pathway analysis of urokinase receptor silenced breast cancer cells: a microarray study. *Oncotarget*. 8:101572–902017;

40. Piqué L, Martinez de Paz A, Piñeyro D, Martínez-Cardús A, Castro de Moura M, Llinàs-Arias P, et al.. Epigenetic inactivation of the splicing RNA-binding protein CELF2 in human breast cancer. *Oncogene*. Springer Science and Business Media LLC; 38:7106–122019;

41. Lai S, Wang Y, Li T, Dong Y, Lin Y, Wang L, et al.. N6-methyladenosine-mediated CELF2 regulates CD44 alternative splicing affecting tumorigenesis via ERAD pathway in pancreatic cancer. *Cell Biosci*. 12:1252022;

42. Qin P, Chen H, Wang Y, Huang L, Huang K, Xiao G, et al.. Cancer-associated fibroblasts undergoing neoadjuvant chemotherapy suppress rectal cancer revealed by single-cell and spatial transcriptomics. *Cell Rep Med*. Elsevier BV; 4:1012312023;

43. Fukuda Y, Bustos MA, Cho S-N, Roszik J, Ryu S, Lopez VM, et al.. Interplay between soluble CD74 and macrophage-migration inhibitory factor drives tumor growth and influences patient survival in melanoma. *Cell Death Dis*. Springer Science and Business Media LLC; 2022; doi: 10.1038/s41419-022-04552-y.

44. Leng L, Metz CN, Fang Y, Xu J, Donnelly S, Baugh J, et al.. MIF signal transduction initiated by binding to CD74. *J Exp Med*. Rockefeller University Press; 197:1467–762003;

45. Zheng C, Zheng L, Yoo J-K, Guo H, Zhang Y, Guo X, et al.. Landscape of infiltrating T cells in liver cancer revealed by single-cell sequencing. *Cell*. 169:1342–56.e162017;

46. Zheng L, Qin S, Si W, Wang A, Xing B, Gao R, et al.. Pan-cancer single-cell landscape of tumor-infiltrating T cells. *Science*. American Association for the Advancement of Science (AAAS); 374:abe64742021;

47. Wilkinson ME, Charenton C, Nagai K. RNA splicing by the spliceosome. *Annu Rev Biochem*. Annual Reviews; 89:359–882020;

48. Tyser RCV, Mahammadov E, Nakanoh S, Vallier L, Scialdone A, Srinivas S. Single-cell transcriptomic characterization of a gastrulating human embryo. *Nature*. Springer Science and Business Media LLC; 600:285–92021;

49. Zhai J, Xiao Z, Wang Y, Wang H. Human embryonic development: from peri-implantation to gastrulation. *Trends Cell Biol*. Elsevier BV; 32:18–292022;

50. Ma S, Zhang B, LaFave LM, Earl AS, Chiang Z, Hu Y, et al.. Chromatin potential identified by shared single-cell profiling of RNA and chromatin. *Cell*. Elsevier BV; 183:1103–16.e202020;

51. Stoeckius M, Hafemeister C, Stephenson W, Houck-Loomis B, Chattopadhyay PK, Swerdlow H, et al.. Simultaneous epitope and transcriptome measurement in single cells. *Nat Methods*. 14:865–82017;

52. Kahles A, Lehmann K-V, Toussaint NC, Hüser M, Stark SG, Sachsenberg T, et al.. Comprehensive analysis of alternative splicing across tumors from 8,705 patients. *Cancer Cell*. 34:211–24.e62018;

53. Cortés-López M, Chamely P, Hawkins AG, Stanley RF, Swett AD, Ganesan S, et al.. Single-cell multi-omics defines the cell-type-specific impact of splicing aberrations in human hematopoietic clonal outgrowths. *Cell Stem Cell*. Elsevier BV; 30:1262–81.e82023;

54. Picelli S, Björklund ÅK, Faridani OR, Sagasser S, Winberg G, Sandberg R. Smart-seq2 for sensitive full-length transcriptome profiling in single cells. *Nat Methods*. Springer Science and Business Media LLC; 10:1096–82013;

55. Picelli S, Faridani OR, Björklund AK, Winberg G, Sagasser S, Sandberg R. Full-length RNA-seq from single cells using Smart-seq2. *Nat Protoc*. Springer Science and Business Media LLC; 9:171–812014;

56. Liao Y, Liu Z, Zhang Y, Lu P, Wen L, Tang F. High-throughput and high-sensitivity full-length single-cell RNA-seq analysis on third-generation sequencing platform. *Cell Discov*. Springer Science and Business Media LLC; 9:52023;

57. Shiau C-K, Lu L, Kieser R, Fukumura K, Pan T, Lin H-Y, et al.. High throughput single cell long-read sequencing analyses of same-cell genotypes and phenotypes in human tumors. *Nat Commun*. 14:41242023;

58. Kumari P, Kaur M, Dindhoria K, Ashford B, Amarasinghe SL, Thind AS. Advances in long-read single-cell transcriptomics. *Hum Genet*. Springer Science and Business Media LLC; 143:1005–202024;

59. Weile J, Ferra G, Boyle G, Pendyala S, Amorosi C, Yeh C-L, et al.. Pacybara: accurate long-read sequencing for barcoded mutagenized allelic libraries. *Bioinformatics*. 2024; doi: 10.1093/bioinformatics/btae182.

60. Dobin A, Davis CA, Schlesinger F, Drenkow J, Zaleski C, Jha S, et al.. STAR: ultrafast universal RNA-seq aligner. *Bioinformatics*. Oxford University Press (OUP); 29:15–212013;

61. Hao Y, Hao S, Andersen-Nissen E, Mauck WM 3rd, Zheng S, Butler A, et al.. Integrated analysis of multimodal single-cell data. *Cell*. Elsevier BV; 184:3573–87.e292021;

62. Stuart T, Butler A, Hoffman P, Hafemeister C, Papalexi E, Mauck WM 3rd, et al.. Comprehensive integration of single-cell data. *Cell*. Elsevier BV; 177:1888–902.e212019;

63. Seiler M, Peng S, Agrawal AA, Palacino J, Teng T, Zhu P, et al.. Somatic mutational landscape of splicing factor genes and their functional consequences across 33 cancer types. *Cell Rep*. 23:282–96.e42018;

64. Wu X, Liu T, Ye C, Ye W, Ji G. scAPAtrap: identification and quantification of alternative polyadenylation sites from single-cell RNA-seq data. *Brief Bioinform*. Oxford University Press (OUP); 2021; doi: 10.1093/bib/bbaa273.

65. Ye W, Liu T, Fu H, Ye C, Ji G, Wu X. movAPA: modeling and visualization of dynamics of alternative polyadenylation across biological samples. *Bioinformatics*. Oxford University Press (OUP); 37:2470–22021;

66. Zhou Y, Zhou B, Pache L, Chang M, Khodabakhshi AH, Tanaseichuk O, et al.. Metascape provides a biologist-oriented resource for the analysis of systems-level datasets. *Nat Commun*. Springer Science and Business Media LLC; 10:15232019;

67. Tang Z, Kang B, Li C, Chen T, Zhang Z. GEPIA2: an enhanced web server for large-scale expression profiling and interactive analysis. *Nucleic Acids Res*. Oxford University Press (OUP); 47:W556–602019;

68. Bai Y, Kosonocky CW, Wang JZ. How our authors are using AI tools in manuscript writing. *Patterns (N Y)*. Elsevier BV; 5:1010752024;

Click here to access/download
Supplementary Material
Supplementary Tables.xlsx

 Click here to access/download;Personal Cover;Cover Letter.pdf

Dear Hongfang,

On behalf of all co-authors, I am pleased to submit our revised manuscript entitled "**AEnet: a practical tool to construct the splicing associated phenotype atlas at single-cell level**" for consideration as an Article in GigaScience. We appreciate the reviewers' insightful comments and suggestions, which have significantly improved the quality, clarity, and impact of our work. All concerns have been addressed comprehensively in the revised manuscript and we also provide point by point response in the response letter.

Key revisions include:

1.Performed Ablation Studies: We systematically evaluated AEnet against alternative splicing (AS)-only (Anet) or gene expression-only (Enet) approaches , demonstrating the superior clustering accuracy of our integrated method using Adjusted Rand Index (ARI) and Normalized Mutual Information (NMI) metrics.

2. Expanded Benchmarking: We have rigorously benchmarked AEnet against established alternative splicing (AS) detection, AS based clustering, and batch correction methods, respectively , such as BRIE, Outrigger, MARVEL, SCASL, Seurat, scSHC, and scQuint, using diverse datasets and objective metrics like ARI and NMI. These comparisons demonstrate AEnet's superior accuracy and efficiency in quantifying alternative splicing (AS) patterns and identifying cell clusters.

3. Improved Methodological Transparency: We reorganized the manuscript to separate the Methods and Benchmarking sections, providing more detailed protocols

and a comprehensive evaluation of AEnet's performance using public and synthetic datasets. We updated the publicly available R package on GitHub (https://github.com/liushang17/AEnet), and provided demonstration datasets and R Markdown scripts to ensure full reproducibility of all figures.

AEnet integrates alternative splicing and gene expression data to construct AS profiling-based clusters, identify key splicing factors, and pinpoint functional pathways for specific ASP. By applying AEnet to various biological contexts, we demonstrate its ability to capture cellular heterogeneity, mitigate batch effects, and reveal novel isoforms of functional importance.

We believe that this revised manuscript addresses the reviewers' concerns and represents a significant contribution to the field of single-cell omics. We are confident that AEnet will be a valuable tool for researchers exploring the interplay between alternative splicing and gene expression.

Thank you for your continued consideration of our manuscript. We look forward to hearing from you soon.

Yours Sincerely,

Liang Wu, PhD

On behalf of all authors

BGI Research, Shenzhen 518083, China.

Email: wuliang@genomics.cn

 Click here to access/download;Personal Cover;Point-by-point response.pdf

Reviewer reports:

Reviewer #1: This paper presents AEnet, a cell clustering method based on the combined gene expression levels and AS patterns for cell-type annotation with scRNA-seq data. This idea is very interesting and I can see the great efforts made by the authors. However, as a method paper, the limited benchmarked datasets and methods make this work too simple, shallow, and not convinced. I have several concerns about the contents of this paper:

(1) Since the inference of AS patterns is the foundation for subsequent analysis and the authors proposed a new approach to infer the AS patterns from scRNA-seq data, this approach should be benchmarked with other known methods, such as BRIE, Outrigger, MARVEL, and their own earlier attempt, DESJ-detection, to demonstrate the superior accuracy or efficiency to quantify the AS patterns.

(2) The authors assumes that the combined gene expression levels and AS patterns is more accurate in identifying the cell clusters than the traditional strategies. If so, the ablation study should be performed by only considering the AS patterns or gene expression levels, to demonstrate the superior accuracy of AEnet compared to so-called Anet (AS only) or Enet (gene expression only) by replacing combined AE matrix with the AS patterns matrix or gene expression matrix and keeping the other parts of processing totally same as that in AEnet.

(3) I observe that the authors compared the performance of AEnet and SCASL (single-cell clustering method based on AS) in identifying the cell clusters (Fig. S3). But the authors focused on the performance of reducing batch effects. This is very unreasonable. Since they are both single-cell clustering methods, the evaluation index should be known ARI, NMI, and/or cell-type annotation accuracy based on the ground-truth cell labels, rather than the patient samples.

(4) Beside the ablation study, the comparison with other widely-used single-cell clustering methods should be conducted, e.g., the Seurat clustering method based on the combined AE matrix (ASP & E), and scSHC, with the known ARI and NMI indexes.

(5) In Fig. 2, the authors mentioned that AEnet can decrease the bias or batch effects. If so, the benchmarked evaluations with other known methods (such as Seurat CCA, Harmony, fastMNN, Conos, LIGER) should be performed to demonstrate the superior accuracy or efficiency to reduce the bias or batch effects.

(6) Since the AEnet is a universal method for clustering, more benchmarked datasets are needed, including the datasets obtained from different scRNA-seq technologies (Smart-seq, Microwell-seq, 10X Genomics, etc.), species, tissues, conditions, diseases (including more non-cancer diseases).

(7) Typo "cell cells" in "how alternative splicing influences cell cells and cell states".

Reviewer #2: Liu et al. developed a novel software (AEnet) for analysing alternative splicing (AS) at single-cell resolution. This software leverages both alternative splicing and gene expression profiles (ASP and EXP, respectively) to build ASP-EXP links. These links are then utilised for identifying cell clusters, cluster-specific regulatory splicing factors and isoforms, and pathway enrichment analysis. Overall, the software demonstrates sufficient novelty over existing single-cell AS tools to warrant publication. The narrative is also sensible and coherent. My only comments pertain to the benchmarking of this software and application of this software beyond the contexts reported in this manuscript, and they are aimed at improving the already high-quality of the manuscript.

Main comments:

1. In Fig. S3A (left panel) and S3C (left panel), were the cell clusters identified by AEnet biologically meaningful? For example, do the clusters demonstrate expression of lineage-specific genes that would enable cell type assignment or identification?

2. The clustering capability of AEnet has not been benchmarked against datasets in which the cell types are known (ground truth). Is AEnet able to delineate the different cell populations whose identity have been previously assigned? The myriads of published peripheral blood mononuclear cells (PBMCs) and bone marrow mononuclear cells (BMMCs) datasets consists of a wide diversity of confidently-assigned cell types suitable for this benchmarking exercise.

3. The clustering capability of AEnet was compared against SCASL and Seurat by visually inspecting the clustering profile on reduced dimension space. While this initial visual-based inspection is sensible, an objective measurement could serve as an additional method of comparison. Two such metrices for clustering assessment are adjusted rand index (ARI) and normalised mutual information (NMI).

4. The ability of AEnet to mitigate batch effect was compared against Seurat and SCASL. For Seurat specifically, the batch effect was "corrected" using the "FindIntegrationAnchors" and "IntegrateData" functions. These functions integrate multiple samples, but do not adjust for batch effect per se. To enable a fair comparison with Seurat, or other means of batch correction, more suitable approaches for adjusting batch effect should be benchmarked against. The canonical correlation analysis (CCA) and Harmony are two batch correction approaches implemented by Seurat that may be benchmarked against.

5. It would be of particular interest to benchmark AEnet against other splicing-based clustering approaches. The variational autoencoder (VAE) algorithm implemented by scQuint (PMID: 35229721) and DOLPHIN (https://apc01.safelinks.protection.outlook.com/?url=https%3A%2F%2Fwww.researchsquare.com%2Farticle%2Frs-5474597%2Fv1&data=05%7C02%7Cwuliang%40genomics.cn%7C19524fec513b46a5e91e08dd65e932ef%7C853aa2281adc4d91bb286065c1e9963d%7C0%7C0%7C638778774818200358%7CUnknown%7CTWFpbGZsb3d8eyJFbXB0eU1hcGkiOnRydWUsIlYiOiIwLjAuMDAwMCIsIlAiOiJXaW4zMiIsIkFOIjoiTWFpbCIsIldUIjoyfQ%3D%3D%7C0%7C%7C%7C&sdata=YxkFdnbgoIhPFzDFAkxTMiwh1cM8PlxZo58M7e2jgco%3D&reserved=0) have demonstrated convincing clustering ability.

6. I appreciate the detailed demonstration of AEnet on tumour-infiltrating T cells and embryonic gastrulation using AEnet, and the identification of regulatory splicing factors in these scenarios. In haematological malignancies such as myelodysplastic syndrome (MDS) and myeloproliferative neoplasm (MPN), more than half of the patients have mutations in splicing factors, namely SF3B1, SRSF2, and U2AF1, and therefore are known to have dysregulated splicing profiles. Moreover, several AS events related to these splicing factors have already been experimentally validated, for example, SEPTIN2 and MAP3K7 are mis-spliced in SFB31-mutant MDS patients. These splicing events may serve as positive controls in benchmarking exercise. Furthermore, related to comment no. 2 above, the haematopoietic compartment consists of a wide variety of cell types suitable for showcasing AEnet functionalities. Therefore, these cancer types are biologically relevant for benchmarking and for demonstrating the application of AEnet. One such example is a dataset that consists of SFB31-mutant MDS samples (PMID: 37582363).

7. The cornerstone of single-cell RNA-sequencing analysis is differential gene and splicing analysis. AEnet excels in revealing regulatory splicing factors, isoforms, and pathways within a cluster or condition. However, AEnet does not enable comparison across different clusters or conditions, e.g., disease versus healthy states. While the lack of this functionality is not a limitation in itself, this may be detailed in the Discussion to aid users in deciding which single-cell AS tools will be best suited to meet their research goals.

8. For the purpose of reproducibility and to convince prospective users, like myself, to use AEnet, please may the authors provide the codes used to generate AEnet-related figures in the manuscript. A R Markdown format is highly encouraged, if possible.

Minor comments:

9. Please may the authors provide the pre-defined list of splicing factors in a Supplementary Table.

10. In paragraph 1 of the Introduction, "… analyzing profilings of AS…" should be "…analyzing profiles of AS…".

11. In paragraph 2 of the Introduction, the authors alluded to the inflated false negatives exemplified by published single-cell AS tools. Has this limitation been demonstrated or investigated for these tools? If not, I recommend the authors to tone down this assertion.

12. In paragraph 2 of the Introduction, the authors also mentioned that current single-cell AS tools do not identify functional pathways. But MARVEL has a functionality to identify enriched functional pathways (PMID: 36631981).

13. In paragraph 3 of the Introduction, the authors listed three challenges of single-cell AS analysis. Please may the authors provide the relevant citations for each of these challenges.

Reviewer #3: In this work, Liu & Chen et al. present AEnet, a computational method for analyzing alternative splicing (AS) at the single-cell level. By integrating AS and scRNA-seq data, they identify alternative splicing pattern (ASP) clusters and use them to define cell subpopulations. The authors demonstrate the utility of their method in three biological contexts: tumor immunotherapy response, tumor-infiltrating T cells, and embryonic development. This work integrates two distinct but complementary data modalities, AS and scRNA-seq, into a single framework, improving the characterization of cellular heterogeneity. The method is well-designed and effectively applied, but several issues should be addressed before publication.
Major points:
* Retaining NaNs for zero counts is valid, but even low-count ASPs may introduce random fluctuations in PSIs. Have you tested different count thresholds? And how does it affect results? If an ASP is rare and mostly NaN, how do you decide whether to include it? Could this introduce bias against infrequent but biologically relevant splicing events?

* The cell populations identified by ASP are demonstrated to be independent of RNA-based clustering. Have you directly compared ASP-only, RNA-only, and joint clustering to assess whether they provide unique biological insights?

* The uniform noise model used in evaluations may not reflect biological noise. Have you tested more realistic noise models?

* AEnet claims to bypass the batch effects, but the mechanism should be better explained. Is this achieved because correlations are calculated within each sample and aggregated later?

* From your results (e.g., Fig. S5C), there seem to be cross-cluster interactions. Are you concerned that rigid clustering may overlook meaningful relationships between clusters?

* Alternative splicing inherently provides positional information. Have you analyzed whether splicing events are enriched in specific genomic regions?

Minor points:
* The criteria for defining a cell subpopulation should be stated in the main text.
* Are the same sequencing results used for gene count matrix and ASP detection?
* The first section provides both method description and validation/benchmarking. Consider separating them into distinct sections and expanding each more thoroughly.
* Can AEnet handle larger single-cell datasets? How does it scale computationally?
* Figures in the first section lack self-explanatory captions. Some (e.g., Fig. 1C, S1B) do not provide sufficient information.
* The anchor ASP identification plots need x-axes, and the y-axes should be labeled "Number of ASP-Gene Links" instead of just "Degree."
* The cited work from Regev's lab in the introduction is a bit old. Are there more recent references that should be included?

Point-by-point response to the referees' comments

The general response to the reviewers:
We sincerely thank all the reviewers for their time and for providing valuable comments and suggestions, which have significantly helped us improve our method. During the revision process, we have carefully addressed all the points raised, as detailed below and marked in blue.

Reviewer reports:

Reviewer #1: This paper presents AEnet, a cell clustering method based on the combined gene expression levels and AS patterns for cell-type annotation with scRNA-seq data. This idea is very interesting and I can see the great efforts made by the authors. However, as a method paper, the limited benchmarked datasets and methods make this work too simple, shallow, and not convinced. I have several concerns about the contents of this paper:

(1) Since the inference of AS patterns is the foundation for subsequent analysis and the authors proposed a new approach to infer the AS patterns from scRNA-seq data, this approach should be benchmarked with other known methods, such as BRIE, Outrigger, MARVEL, and their own earlier attempt, DESJ-detection, to demonstrate the superior accuracy or efficiency to quantify the AS patterns.

Response: Thank you for highlighting this critical point. We have rigorously benchmarked AEnet against BRIE, Outrigger, MARVEL, and our prior method DESJ-detection, demonstrating its superior accuracy in quantifying AS patterns. Current methods (BRIE [1], Outrigger [2], and MARVEL [3]) are all fundamentally annotation-dependent and therefore unable to detect unannotated AS events. Additionally, these tools are limited in their ability to detect MSE **(Fig. R1A, Table R1)**. Since MARVEL has been compared with other methods and demonstrated to be the optimal one in its category in a previous study [3], we next compared the AS events detected by AEnet and MARVEL using the demo dataset of MARVEL, which includes induced pluripotent stem cells (iPSCs) and iPSC-derived endoderm cells [4] **(Fig. R1B)**. MARVEL identified a total of 20,509 SE, 1,279 MXE, 8,295 RI, 5,163 A5SS, 5,832 A3SS, 5,818 AFE, 2,072 ALE, and 0 MSE **(Fig. R1C)**. In MARVEL's iPSC dataset, AEnet detected 722,278 additional AS patterns (63% unannotated) across 12,866 genes, with MSE detection 3.2-fold higher than MARVEL **(Fig. R1D-E)**. These findings highlight AEnet's comprehensive capability to detect a wide range of AS events—including unannotated and complex patterns—except for intron

retention events.

Type	BRIE	Outrigger	MARVEL	DESJ-detection	AEnet
SE	√	/	√	√	√
MXE	/	√	√	√	√
RI	/	/	√	/	/
A3SS	/	/	√	√	√
A5SS	/	/	√	√	√
AFE	/	/	√	√	√
ALE	/	/	√	√	√
MSE	/	/	√	√	√
Unannotated AS events	/	/	/	√	√

Table R1. Summary of the five methods.

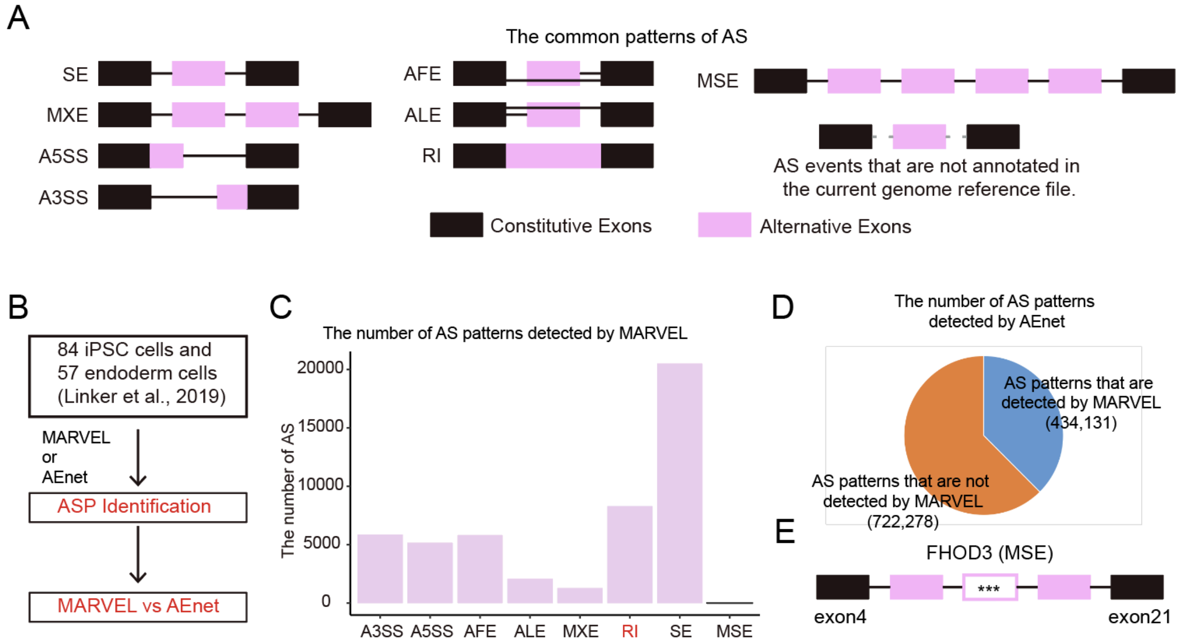

Figure R1. (A) The common types of Alternative splicing. **(B)** The comparison between AEnet and MARVEL in the detection of ASPs. **(C)** The number of alternative splicing events detected by MARVEL. **(D)** The number of alternative splicing events detected by AEnet as well as the comparison with MARVEL. **(E)** The demo example of FHOD3 for the rare patterns detected by AEnet.

Compared to our homologous method DESJ-detection [5], AEnet overcomes critical limitations in low-depth robustness. DESJ-detection's single-junction PSI calculation leads to 38% error rates in low-coverage scenarios (**Fig. R1F-G**). AEnet mitigates this by focusing on junction reads with shared splice sites (requiring ≥5 supporting reads) and standardizing read distributions to transcript usage ratios, reducing PSI error by 47% ($p<0.001$, **Fig. R1H**).

Taken together, AEnet outperforms existing methods in detecting unannotated alternative splicing events, resolving complex AS patterns, and ensuring reliable quantification across varying data depths. We have incorporated this suggestion into the manuscript at Page9, Line 201-223 and revised Figure S3.

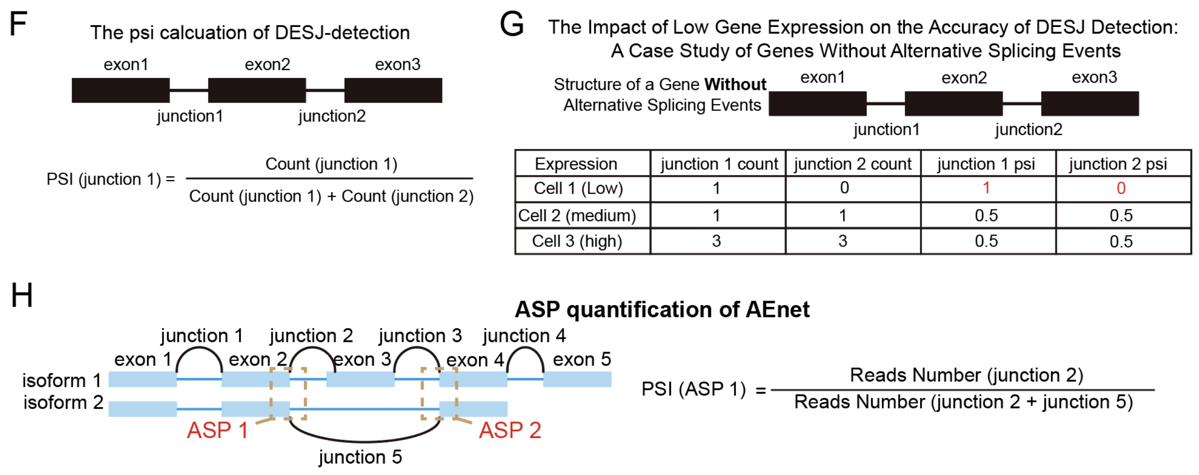

Figure R1. (G) Schematic illustration of PSI calculation in DESJ-detection. **(H)** Impact of Low Gene Expression on the Accuracy of DESJ Detection: A Case Study Using Genes Without Alternative Splicing Events. **(I)** Schematic Diagram of the calculation of PSI for Alternative Splicing Pattern (ASP).

(2) The authors assumes that the combined gene expression levels and AS patterns

is more accurate in identifying the cell clusters than the traditional strategies. If so, the ablation study should be performed by only considering the AS patterns or gene expression levels, to demonstrate the superior accuracy of AEnet compared to so-called Anet (AS only) or Enet (gene expression only) by replacing combined AE matrix with the AS patterns matrix or gene expression matrix and keeping the other parts of processing totally same as that in AEnet.

Response: We appreciate your insightful comment. To serve the question, three independent datasets—iPSC [2], T cell [6], and HCC [7]—were used for evaluation, and detailed information is provided in **Table R2**. Comprehensive ablation analyses using published cell type annotations as ground truth demonstrated that the joint ASP-EXP model (AEnet) consistently outperformed the standalone AS-only (Anet) and expression-only (Enet) approaches **(Fig. R2A)**. Specifically, the median ARI scores were 0.81 (AEnet), 0.68 (Anet), and 0.77 (Enet) for the iPSC dataset; 0.58 (AEnet), 0.10 (Anet), and 0.39 (Enet) for the HCC dataset; and 0.42 (AEnet), 0.10 (Anet), and 0.32 (Enet) for the T cell dataset **(Fig. R2B–D)**. These results demonstrate that integrating ASP features with gene expression markedly improves clustering resolution and biological interpretability. Furthermore, AEnet generated the most informative low-dimensional embeddings across all datasets **(Fig. R2E–G)**, accurately capturing cellular architecture in iPSCs, delineating major lineages in HCC, and resolving functionally distinct T cell subsets—capabilities that were limited or absent in the AS-only and EXP-only models. Thus, AEnet consistently achieves superior performance by jointly leveraging alternative splicing and gene expression to reveal biologically meaningful cellular heterogeneity. We have incorporated this suggestion into the manuscript at **Page15, Line 311-324** and revised **Figure 2A-D** and **S7**.

dataset	Sample Number	Cell Number	Average reads per cell	Platform	Cell Types
iPSC dataset2	1	191	11,932,904	Smart-seq2 (PE 100)	iPSCs (62), NPCs (69), and MNs (60)
HCC dataset	19	16,963	12,633,167	Smart-seq2 (PE 100)	Tumor cells (2737), Epi (34), Endo (99), HSC (119), pDC (47), Plasma (83), B cell (819), Myeloid (3293), Tcell (7294), Plasma (83), and NK (1947)
T cels dataset	12	8,530	1,250,000	Smart-seq2 (PE 100)	CD4_C01.CCR7 (472), CD4_C02.ANXA1 (509), CD4_C03.GNLY (170), CD4_C04.TCF7 (331), CD4_C05.CXCR6 (639), CD4_C06.CXCR5 (216), CD4_C07.GZMK (204), CD4_C08.IL23R (229), CD4_C09.CXCL13 (272), CD4_C10.FOXP3 (365), CD4_C11.IL10 (176), CD4_C12.CTLA4 (1319), CD8_C01.LEF1 (164), CD8_C02.GPR183 (155), CD8_C03.CX3CR1 (773), CD8_C04.GZMK (840), CD8_C05.CD6 (431), CD8_C06.CD160 (363), CD8_C07.LAYN (831)

Table R2. Summary of the three datasets

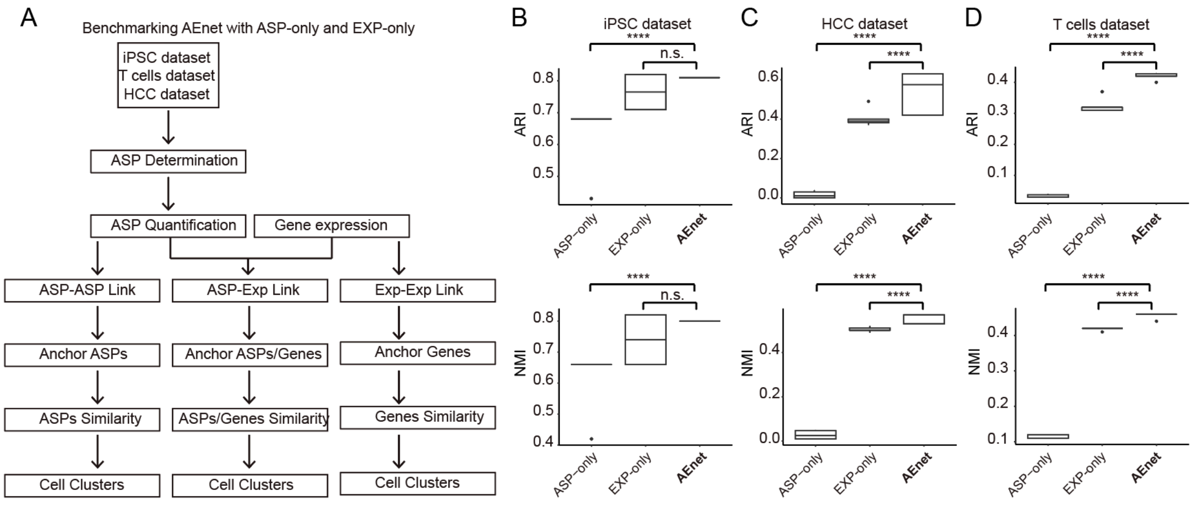

Figure R2. (A) Schematic of the assessment pipeline for ASP-only, RNA-only, and joint clustering analyses. **(B-D)** Quantitative benchmarking of clustering concordance using (upper) Adjusted Rand Index (ARI) and (down) Normalized Mutual Information (NMI) metrics across the three networks. Statistical significance was assessed via one-sided Wilcoxon rank-sum tests. * $P < 0.05$, ** $P < 0.01$, *** $P < 0.001$, **** $P < 0.0001$; n.s., not significant.

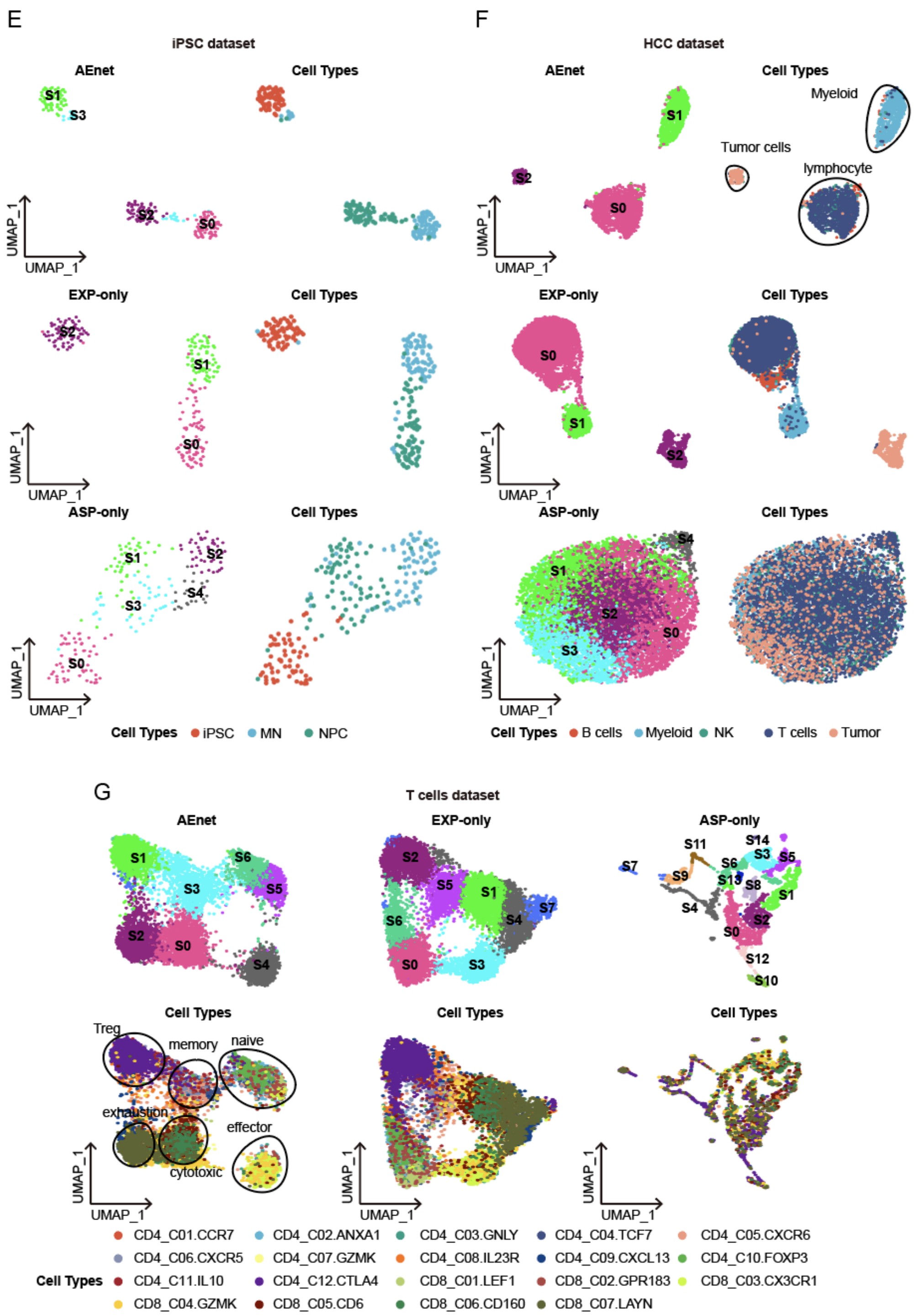

Figure R2. (E–F) Uniform Manifold Approximation and Projection (UMAP) visualizations showing the published cell type annotations (right) and the predicted clusters (left) derived from the ASP-EXP (AEnet) network (top), expression-only (EXP) network (middle), and ASP-only network (bottom) for (E) full-length iPSC and (F) hepatocellular carcinoma (HCC) single-cell RNA-seq datasets. **(G)** UMAP visualizations showing the published cell type annotations (botton) and the predicted

clusters (upper) derived from the ASP-EXP (AEnet) network (left), expression-only (EXP) network (middle), and ASP-only network (right) for the T cells datasets. Clustering solutions shown represent those with the highest adjusted Rand index (ARI).

(3) I observe that the authors compared the performance of AEnet and SCASL (single-cell clustering method based on AS) in identifying the cell clusters (Fig. S3). But the authors focused on the performance of reducing batch effects. This is very unreasonable. Since they are both single-cell clustering methods, the evaluation index should be known ARI, NMI, and/or cell-type annotation accuracy based on the ground-truth cell labels, rather than the patient samples.

Response: We appreciate the reviewer's suggestion to use ARI and NMI metrics for a more objective comparison between AEnet and SCASL[8]. We have now included a quantitative comparison using these metrics (Figure R3). The results show that AEnet achieves significantly higher ARI and NMI scores compared to SCASL, indicating its superior performance in cell clustering. This demonstrates that AEnet not only reduces batch effects but also provides more accurate cell cluster identification compared to SCASL.

Comprehensive benchmarking demonstrates that AEnet consistently outperforms SCASL in clustering accuracy, achieving higher ARI scores across all three datasets: iPSC (AEnet: 0.81 vs. SCASL: 0.37), T cell (AEnet: 0.42 vs. SCASL: 0.29), and HCC (AEnet: 0.58 vs. SCASL: 0.35) **(Fig. R3A-C).** Moreover, AEnet produced more informative low-dimensional embeddings across all datasets. In the iPSC dataset, SCASL failed to effectively distinguish iPSCs from NPCs, whereas AEnet accurately captured these distinctions **(Fig. R3A, D)**. In the HCC dataset, AEnet robustly separated three major lineages—lymphocytes, myeloid cells, and malignant epithelial cells—while SCASL showed limited resolution between myeloid and lymphoid populations **(Fig. R3B, E)**. Similarly, in the T cell dataset, SCASL generated overlapping clusters and failed to resolve distinct functional subsets, in contrast to the well-delineated subpopulations identified by AEnet **(Fig. R3C, F)**. Collectively, these results demonstrate that integrating alternative splicing features with gene expression enables AEnet to more accurately capture cellular

heterogeneity and biologically meaningful substructures, establishing it as a superior tool for single-cell clustering. We have incorporated this suggestion into the manuscript at **Page15, Line 325-342** and revised **Figure 2E-G and S8A-C**.

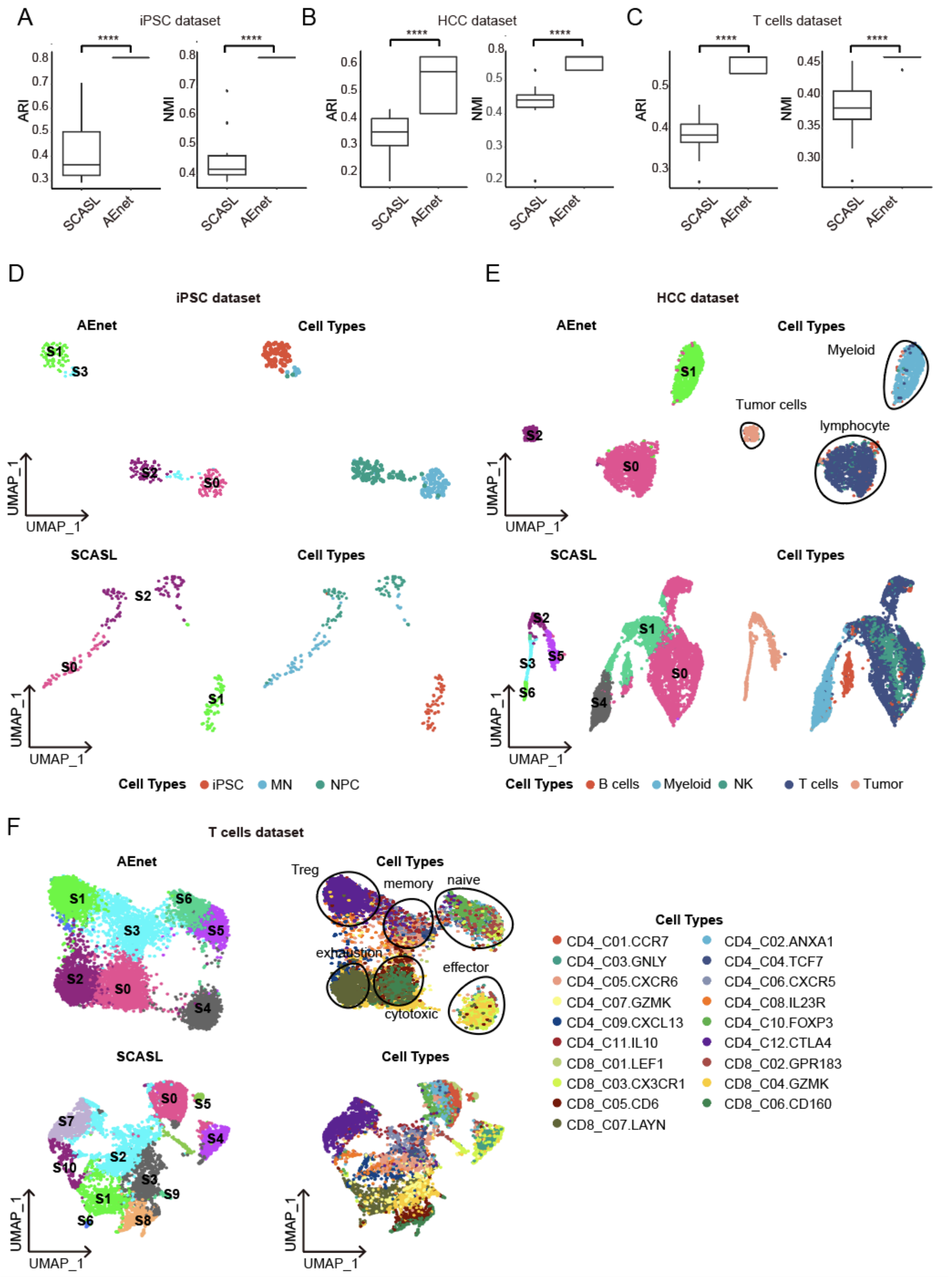

Figure R3. (A-C) Quantitative benchmarking of clustering concordance using (left) ARI and (right) NMI metrics of AEnet and SCASL. **(D–F)** UMAP visualizations showing the published cell type annotations (right) and the predicted clusters (left) derived from AEnet (upper) and SCASL (botton) for: (D) full-length iPSC (E) hepatocellular carcinoma (HCC) single-cell RNA-seq datasets, and the (F) T cell dataset. Clustering solutions shown represent those with the highest adjusted Rand index (ARI). Statistical significance was assessed via one-sided Wilcoxon rank-sum tests. * $P < 0.05$, ** $P < 0.01$, *** $P < 0.001$, **** $P < 0.0001$; n.s., not significant.

(4) Beside the ablation study, the comparison with other widely-used single-cell clustering methods should be conducted, e.g., the Seurat clustering method based on the combined AE matrix (ASP & E), and scSHC, with the known ARI and NMI indexes.

Response: We fully appreciate your careful assessment of this issue. We evaluated the clustering performance of AEnet, scSHC[9], and Seurat[10] using ARI and NMI as quantitative metrics. As scSHC does not permit manual adjustment of clustering resolution or specification of the number of clusters, we applied the method using its default parameters, which yielded a single clustering result. Moreover, scSHC does not provide functionality for generating low-dimensional embeddings.

To ensure consistency across methods, the cell–junction count matrix was used as input for both scSHC and Seurat. Comprehensive benchmarking demonstrates that AEnet consistently outperforms Seurat and scSHC in clustering accuracy **(Fig. R4A–C)**. Specifically, the median ARI scores for the iPSC dataset were 0.81 (AEnet), 0.81 (Seurat), and 0.82 (scSHC); for the HCC dataset, 0.58 (AEnet), 0.29 (Seurat), and 0.10 (scSHC); and for the T cell dataset, 0.42 (AEnet), 0.36 (Seurat), and 0.26 (scSHC) **(Fig. R4A–C).** Seurat produced overly complex and less interpretable embeddings in the T cell dataset, compared to AEnet **(Fig. R4D-F)**. Additionally, in both the HCC and T cell datasets, Seurat generated several small, fragmented clusters. Notably, these mini-clusters were primarily driven by junctions from specific genes—such as ACTG1—and did not reflect genuine splicing heterogeneity **(Fig. R4G)**. Instead, they were associated with differential gene expression, suggesting that Seurat may be suboptimal for detecting key alternative splicing events. Thus, AEnet outperforms both Seurat and scSHC in clustering accuracy across multiple datasets, offering more biologically meaningful clustering and interpretable embeddings.

We sincerely thank the reviewer for this insightful recommendation. In response, we have conducted the suggested comparative analyses. However, given that our manuscript primarily aims to introduce and validate AEnet in the context of alternative splicing analysis—and that the compared methods (Seurat and scSHC) are primarily designed for gene expression rather than splicing-based inference—we have decided not to include these results in the main text at this stage. We believe this choice helps maintain focus and coherence in the manuscript, and we greatly appreciate the reviewer's understanding on this point.

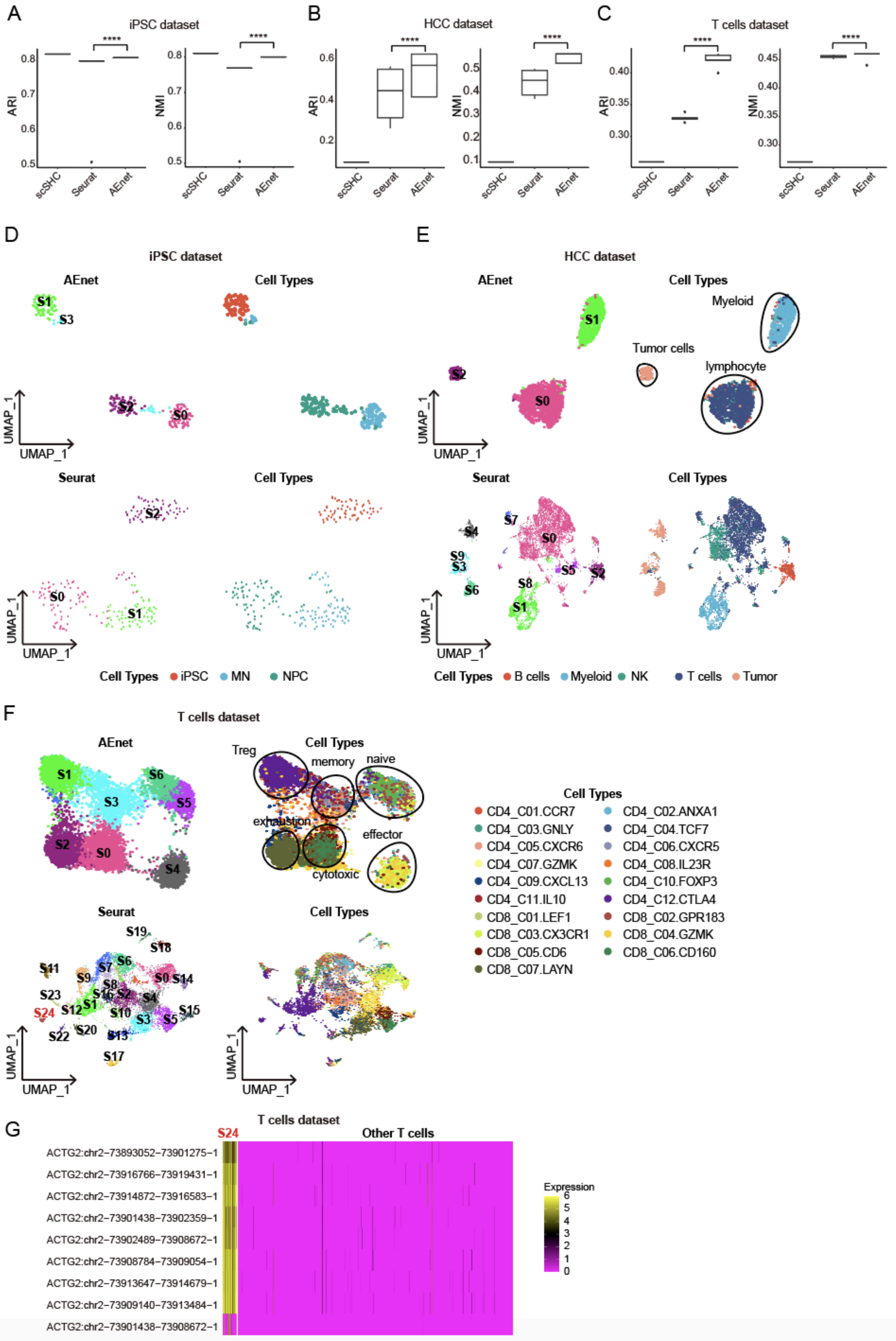
A
iPSC dataset
ARI
NMI
scSHC
Seurat
AEnet
B
HCC dataset
C
T cells dataset
D
iPSC dataset
AEnet
Cell Types
Seurat
UMAP_1
UMAP_1
Cell Types iPSC MN NPC
E
HCC dataset
Myeloid
Tumor cells
lymphocyte
Cell Types B cells Myeloid NK T cells Tumor
F
T cells dataset
Treg
memory
naive
exhaustion
effector
cytotoxic
Cell Types
CD4_C01.CCR7
CD4_C02.ANXA1
CD4_C03.GNLY
CD4_C04.TCF7
CD4_C05.CXCR6
CD4_C06.CXCR5
CD4_C07.GZMK
CD4_C08.IL23R
CD4_C09.CXCL13
CD4_C10.FOXP3
CD4_C11.IL10
CD4_C12.CTLA4
CD8_C01.LEF1
CD8_C02.GPR183
CD8_C03.CX3CR1
CD8_C04.GZMK
CD8_C05.CD6
CD8_C06.CD160
CD8_C07.LAYN
G
T cells dataset
S24
Other T cells
ACTG2:chr2−73893052−73901275−1
ACTG2:chr2−73916766−73919431−1
ACTG2:chr2−73914872−73916583−1
ACTG2:chr2−73901438−73902359−1
ACTG2:chr2−73902489−73908672−1
ACTG2:chr2−73908784−73909054−1
ACTG2:chr2−73913647−73914679−1
ACTG2:chr2−73909140−73913484−1
ACTG2:chr2−73901438−73908672−1
Expression

Figure R4. (A-C) Quantitative benchmarking of clustering concordance using (left) ARI and (right) NMI metrics of AEnet and Seurat. **(D–F)** UMAP visualizations showing the published cell type annotations (right) and the predicted clusters (left) derived from AEnet (upper) and Seurat (botton) for: (D) full-length iPSC (E) hepatocellular carcinoma (HCC) single-cell RNA-seq datasets, and the (F) T cell dataset. Clustering solutions shown represent those with the highest adjusted Rand index (ARI). **(G)** The differentially junction for S24 in the T cells dataset. Statistical significance was assessed via one-sided Wilcoxon rank-sum tests. * P < 0.05, ** P < 0.01, *** P < 0.001, **** P < 0.0001; n.s., not significant.

(5) In Fig. 2, the authors mentioned that AEnet can decrease the bias or batch effects. If so, the benchmarked evaluations with other known methods (such as Seurat CCA, Harmony, fastMNN, Conos, LIGER) should be performed to demonstrate the superior accuracy or efficiency to reduce the bias or batch effects.

Response: We thank the reviewer for the insightful comment. In response, we conducted benchmarking comparisons to evaluate AEnet's ability to mitigate batch effects. As AEnet is tailored to detect splicing heterogeneity, we focused on batch correction performance in the PSI-based framework.

To ensure a fair comparison, we applied two widely used batch correction methods—Seurat CCA [10] and Harmony[11]—to the cell–junction count matrix prior to PSI computation **(Fig. R5A-D)**. However, these methods are primarily optimized for gene expression data and are not well-suited for alternative splicing analysis. In particular, they lack the sensitivity to detect key splicing events and fail to capture splicing-driven cellular heterogeneity. Consequently, AEnet outperformed both methods in identifying biologically meaningful cell heterogeneity, achieving significantly higher ARI and NMI scores **(Fig. R5A-B)**. Moreover, CCA and Harmony tended to overcorrect, resulting in fragmented clusters driven by expression noise (e.g., ACTG1-associated mini-clusters) rather than true alternative splicing patterns **(Fig. R5C-D)**.

By contrast, AEnet successfully preserves AS-based clustering structure and captures shared splicing variation across multiple samples or conditions, compared to SCASL **(Fig. R5E-H)**. Although methods like fastMNN[12], Conos[13], and LIGER [10] are also widely used for batch effect correction, their applicability to PSI matrices is similarly limited, and they are not designed to model splicing-specific heterogeneity. In summary, AEnet offers a splicing-aware alternative to general batch

correction methods, demonstrating superior performance in preserving biologically relevant AS variation across diverse datasets. We have incorporated this suggestion into the manuscript at **Page 15-16, Line 343-353** and **revised Figure S9**.

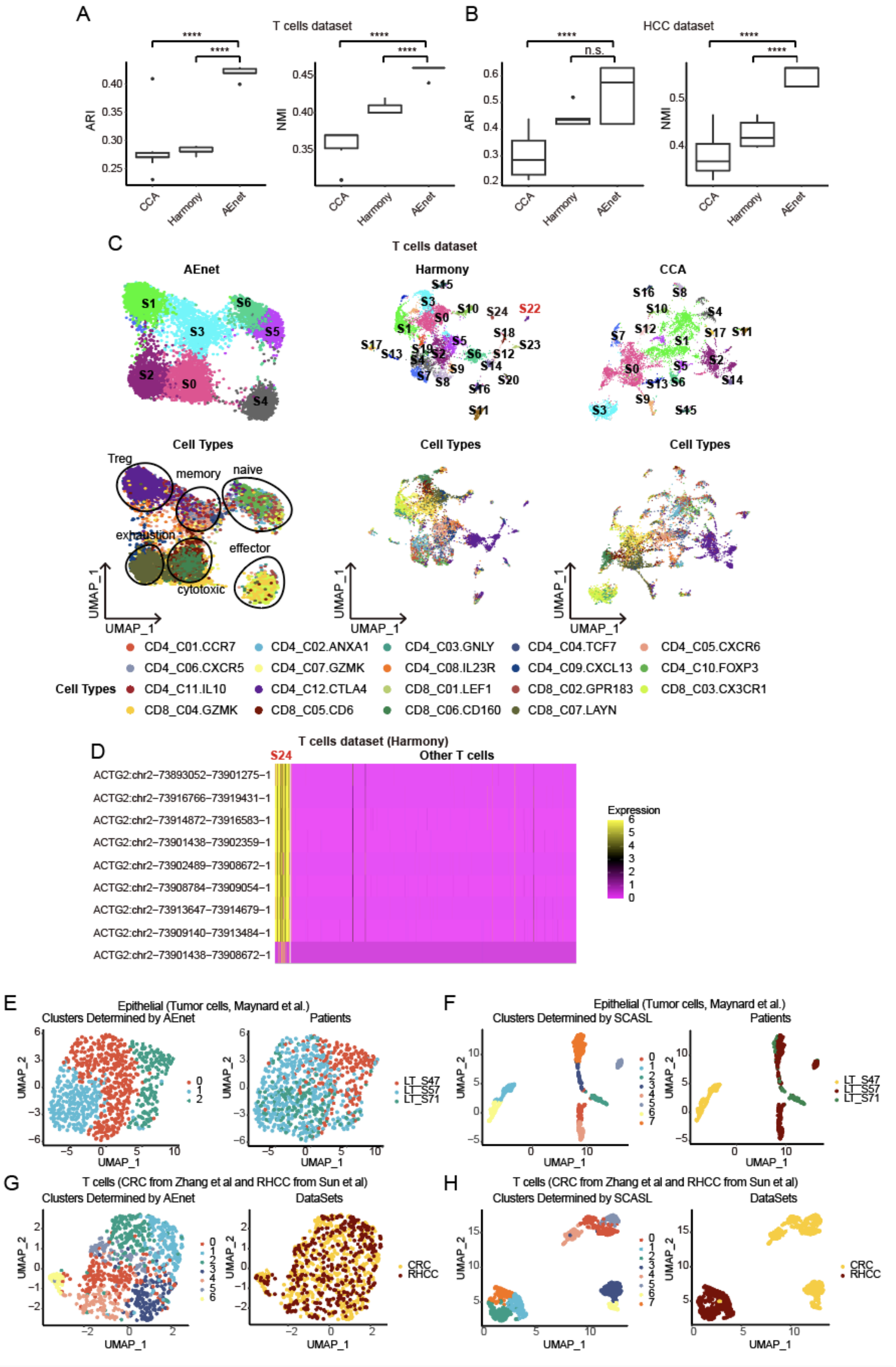

Figure R5. (A-B). Quantitative benchmarking of clustering concordance using **(A)** Adjusted Rand Index (ARI) and **(B)** Normalized Mutual Information (NMI) metrics across integration methods. **(C)** UMAP visualizations showing the published cell type annotations (botton) and the predicted clusters (upper) derived from AEnet (left), Harmony (middle), and CCA (right) for the T cells datasets. Clustering solutions shown represent those with the highest adjusted Rand index (ARI). **(E)** The differentially junction for S22 in the T cells dataset. **(E-F)** UMAP shows the clustering of cell types determined by AEnet **(E)** and SCASL **(F)** (left panel) and patient clustering (right panel) for epithelial cells from multiple patients. **(G-H).** UMAP shows the clustering of cell types determined by AEnet **(G)** and SCASL **(H)** (left panel) and patient clustering (right panel) for T cells from the CRC (Colorectal cancer) and RHCC (Recurrent Hepatocellular carcinoma) datasets. Statistical significance was assessed via one-sided Wilcoxon rank-sum tests * $P < 0.05$, ** $P < 0.01$, *** $P < 0.001$, **** $P < 0.0001$; n.s., not significant

(6) Since the AEnet is a universal method for clustering, more benchmarked datasets are needed, including the datasets obtained from different scRNA-seq technologies (Smart-seq, Microwell-seq, 10X Genomics, etc.), species, tissues, conditions, diseases (including more non-cancer diseases).

Response: We thank the reviewer for raising this important point regarding the generalizability of AEnet. Owing to the ultra-high sequencing depth and full-length coverage of transcripts provided by the Smart-seq2 platform, most of our alternative splicing (AS)-based analyses have been conducted using Smart-seq2 data. Nevertheless, AEnet remains effective when sequencing depth is adequate. To further demonstrate its versatility, we applied AEnet to a widely used 10X Genomics PBMC dataset **(Fig. R6A)** [14]. We have incorporated this suggestion into the manuscript at **Page16, Line 354-366** and revised **Figure S9**.

Due to the limited sequencing depth and only single-end coverage for transcript (5‘ end or 3’ end) of 10X data, alternative polyadenylation (APA) events, which also reflect isoform-level regulation, are main AS events that could be investigated in 10X data. In this dataset, AEnet identified 190 anchor alternative polyadenylation (APA) events and delineated 7 APA-defined clusters **(Fig. R6B)**. These clusters corresponded well to known immune cell subpopulations, including B cells, $CD4^+$ T cells, $CD8^+$ T cells, dendritic cells, macrophages, mast cells, monocytes, neutrophils, natural killer (NK) cells, and regulatory T (Treg) cells **(Fig. R6C)**. Clustering results were consistent with established biological annotations. Quantitative benchmarking further confirmed AEnet’s robustness, achieving a median Adjusted Rand Index

(ARI) of 0.83 and a Normalized Mutual Information (NMI) of 0.78 **(Fig. R6D)**. These results demonstrate that AEnet can effectively capture the contribution of isoform usage to cellular heterogeneity based on 10x Genomics datasets.

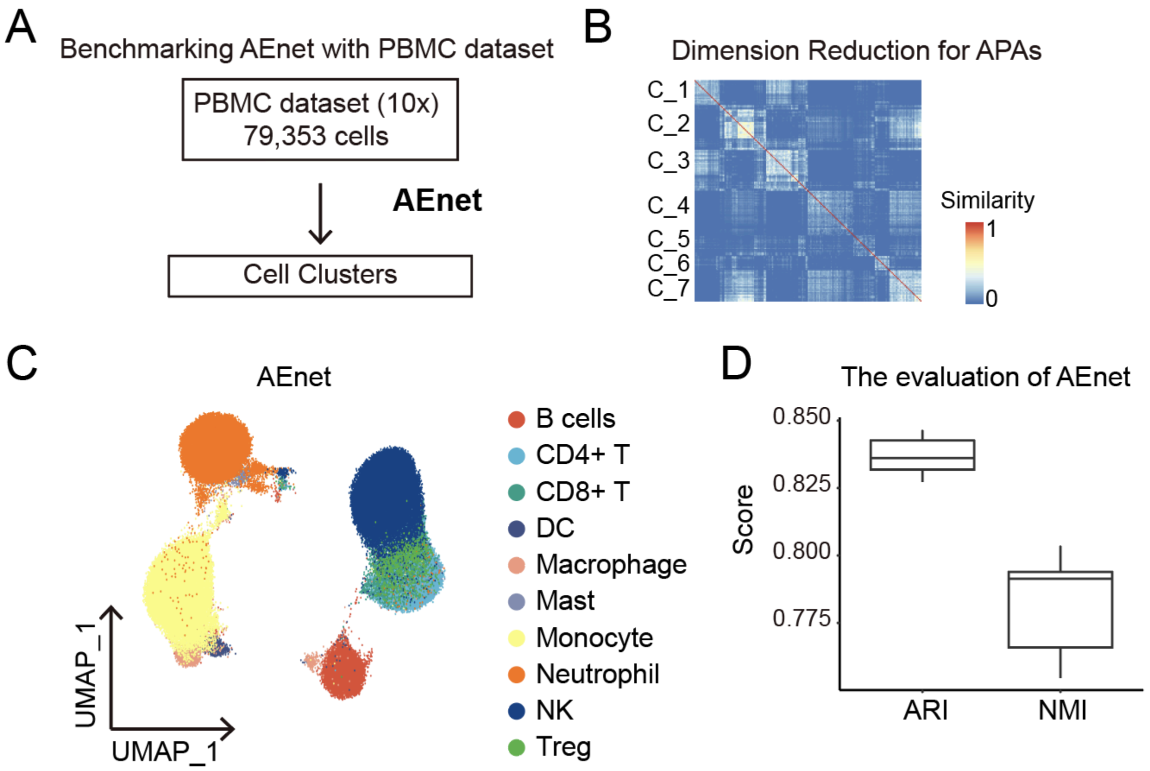

Figure R6. (A) Schematic of the assessment pipeline for AEnet applied to the PBMC dataset from 10x Genomics. **(B)** Heatmap showing APA classes derived from dimensionality reduction using AEnet. **(C)** UMAP visualization of cell clustering based on alternative polyadenylation profiles. **(D)** Quantitative benchmarking of clustering performance using ARI and NMI, evaluated against the ground truth annotations of the PBMC dataset.

(7) Typo "cell cells" in "how alternative splicing influences cell cells and cell states".

Response: The phrase "cell cells" was indeed a typographical error, and we have corrected to "how alternative splicing influences cell types and cell states" (Page3, Line 85) .

Reviewer #2: Liu et al. developed a novel software (AEnet) for analysing alternative splicing (AS) at single-cell resolution. This software leverages both alternative splicing and gene expression profiles (ASP and EXP, respectively) to build ASP-EXP links. These links are then utilised for identifying cell clusters, cluster-specific regulatory splicing factors and isoforms, and pathway enrichment analysis. Overall,

the software demonstrates sufficient novelty over existing single-cell AS tools to warrant publication. The narrative is also sensible and coherent. My only comments pertain to the benchmarking of this software and application of this software beyond the contexts reported in this manuscript, and they are aimed at improving the already high-quality of the manuscript.

Main comments:

1. In Fig. S3A (left panel) and S3C (left panel), were the cell clusters identified by AEnet biologically meaningful? For example, do the clusters demonstrate expression of lineage-specific genes that would enable cell type assignment or identification?

Response: We are grateful for this thoughtful comment. The clusters identified by AEnet in Fig. S3A and S3C do exhibit clear biological relevance. In Fig. S3A **(Fig. R7A)**, AEnet successfully uncovered shared AS heterogeneity across multiple patients in the lung cancer cell dataset[15]. The resulting clusters reflected a continuum of therapeutic response, progressing from progressive disease (PD; subpopulation S2), to residual disease (RD; S0), to a normal response state (S1), as visualized along the UMAP_1 axis **(Fig. R7A-B)**. The marker genes of S1 (N) were enriched in alveolar signatures, including AQP4, SFTPB/C/D, NKX2-1, and FOXA2, while S2 (PD) was associated with elevated expression of prothrombin activation genes (PLAT, PLAUR), gap-junction proteins (GJB2/3/5), and the well-known EMT (epithelial-mesenchymal transition) marker EPCAM **(Fig. R7C)**. This description has been incorporated into the revised manuscript (**Page 21, Lines 420–427**) and revised **Figure 3C-D.**

In the colorectal cancer (CRC) [6] and recurrent hepatocellular carcinoma (RHCC) [7]T cell datasets, AEnet also effectively captured alternative splicing-driven heterogeneity among T cell populations **(Fig. R7D)**. Specifically, subpopulation S3 expressed naïve T cell markers (IL7R, CCR7, LEF1), S0 and S5 were enriched in memory T cell markers (CD52, ANXA1, CREM), S1 displayed effector T cell markers (NKG7, GZMA/B), S2 and S4 represented exhausted T cell states (PDCD1, CTLA4, HAVCR2), and S6 corresponded to proliferative T cells (MKI67, TOP2A) **(Fig. R7E)**. Notably, AEnet did not stratify T cells into traditional $CD4^+$ and $CD8^+$ lineages, suggesting that alternative splicing plays a more prominent role in modulating functional state transitions rather than in defining classical T cell lineages. TThis

interpretation is in the revised manuscript at **Page 23, Line 475-487** and revised **Figure S10E**.

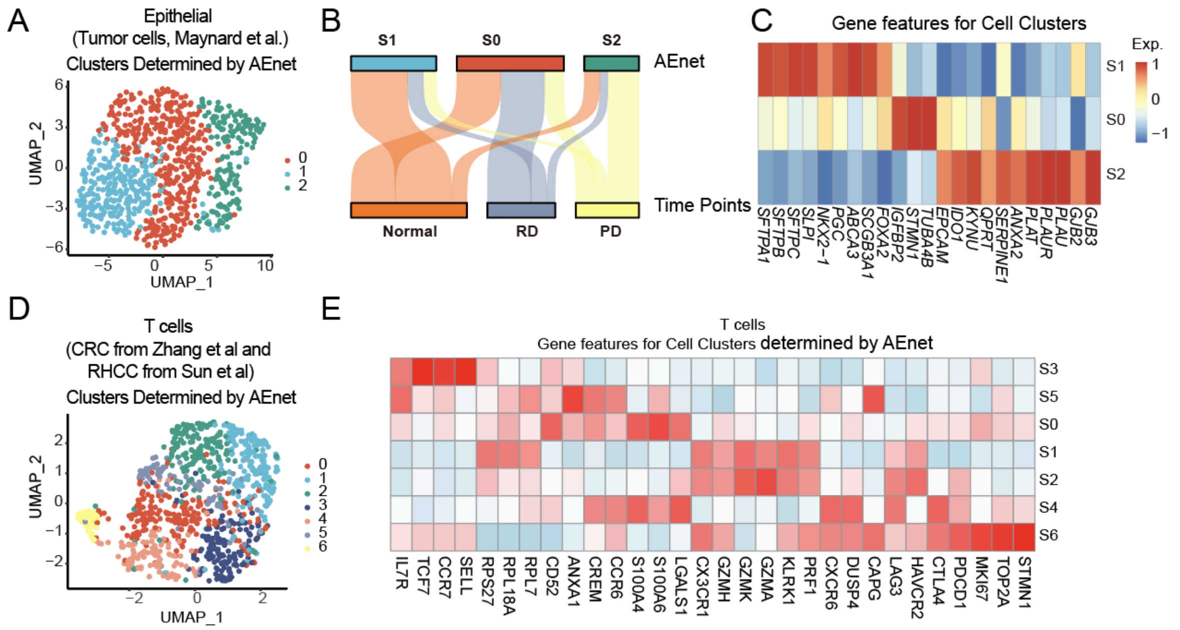

Figure R7. (A) UMAP shows the clustering of cell types determined by AEnet for epithelial cells from multiple patients. **(B)** The Sankey plot shows the overlapping of cells between Time Points and Clusters determined by AEnet. **(C)** The heatmap displays the expression of marker genes across cell clusters determined by AEnet for epithelial cells. **(D)** UMAP shows the clustering of cell types determined by AEnet for T cells from the CRC (Colorectal cancer) and RHCC (Recurrent Hepatocellular carcinoma) datasets. **(E)** The heatmap displays the expression of marker genes across cell clusters determined by AS.

2. The clustering capability of AEnet has not been benchmarked against datasets in which the cell types are known (ground truth). Is AEnet able to delineate the different cell populations whose identity have been previously assigned? The myriads of published peripheral blood mononuclear cells (PBMCs) and bone marrow mononuclear cells (BMMCs) datasets consists of a wide diversity of confidently-assigned cell types suitable for this benchmarking exercise.

Response: Thank you for highlighting this important point. We next focused on evaluating the clustering capability of AEnet using published datasets as ground truth references with ARI and NMI as evaluation metrics. To serve the question, three independent datasets—iPSC [2], T cell [6], and HCC [7]—were used for evaluation, and detailed information is provided in **Table R3**.

Comprehensive ablation analyses using published cell type annotations as ground truth demonstrated that the joint ASP-EXP model (AEnet) consistently outperformed the standalone AS-only (Anet) and expression-only (Enet) approaches **(Fig. R8A)**.

Specifically, the median ARI scores were 0.81 (AEnet), 0.68 (Anet), and 0.77 (Enet) for the iPSC dataset; 0.58 (AEnet), 0.10 (Anet), and 0.39 (Enet) for the HCC dataset; and 0.42 (AEnet), 0.10 (Anet), and 0.32 (Enet) for the T cell dataset **(Fig. R8B–D)**. These results demonstrate that integrating ASP features with gene expression markedly improves clustering resolution and biological interpretability. Furthermore, AEnet generated the most informative low-dimensional embeddings across all datasets **(Fig. R8E–G)**, accurately capturing cellular architecture in iPSCs, delineating major lineages in HCC, and resolving functionally distinct T cell subsets—capabilities that were limited or absent in the AS-only and EXP-only models. Thus, AEnet consistently achieves superior performance by jointly leveraging alternative splicing and gene expression to reveal biologically meaningful cellular heterogeneity. We have incorporated this suggestion into the manuscript at **Page15, Line 311-324 and revised Figure 2A-D and S7**.

As a preliminary attempt, we analyzed a publicly available PBMC dataset (GSE132044), which includes two samples and 528 cells with an average read depth of 1,061,009. However, due to the sequencing format being paired-end 50 bp (PE50) rather than the more informative PE100, the alternative splicing (AS) signal in this dataset was limited. Specifically, only 3,202 alternative splicing patterns were detected, resulting in just 6,524 ASP-EXP links. This led to the identification of only 6 anchor ASPs and no identifiable ASP clusters, rendering this dataset unsuitable for robust evaluation of AEnet's performance **(Fig. R8H)**. We agree with the reviewer that PBMC and BMMC datasets represent valuable benchmarking resources, and we plan to incorporate higher-depth datasets in future evaluations to further demonstrate the generalizability of AEnet.

dataset	Sample Number	Cell Number	Average reads per cell	Platform	Cell Types
iPSC dataset2	1	191	11,932,904	Smart-seq2 (PE 100)	iPSCs (62), NPCs (69), and MNs (60)
HCC dataset	19	16,963	12,633,167	Smart-seq2 (PE 100)	Tumor cells (2737), Epi (34), Endo (99), HSC (119), pDC (47), Plasma (83), B cell (819), Myeloid (3293), Tcell (7294), Plasma (83), and NK (1947)
T cels dataset	12	8,530	1,250,000	Smart-seq2 (PE 100)	CD4_C01.CCR7 (472), CD4_C02.ANXA1 (509), CD4_C03.GNLY (170), CD4_C04.TCF7 (331), CD4_C05.CXCR6 (639), CD4_C06.CXCR5 (216), CD4_C07.GZMK (204), CD4_C08.IL23R (229), CD4_C09.CXCL13 (272), CD4_C10.FOXP3 (365), CD4_C11.IL10 (176), CD4_C12.CTLA4 (1319), CD8_C01.LEF1 (164), CD8_C02.GPR183 (155), CD8_C03.CX3CR1 (773), CD8_C04.GZMK (840), CD8_C05.CD6 (431), CD8_C06.CD160 (363), CD8_C07.LAYN (831)

Table R3. Summary of the three datasets

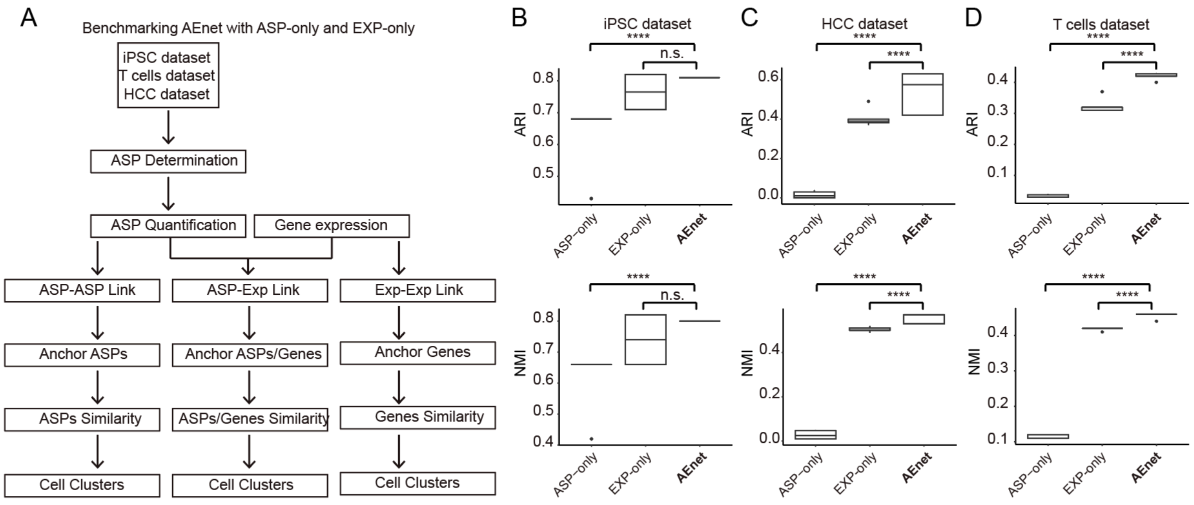

Figure R8. (A) Schematic of the assessment pipeline for ASP-only, RNA-only, and joint clustering analyses. **(B-D)** Quantitative benchmarking of clustering concordance using (upper) Adjusted Rand Index (ARI) and (down) Normalized Mutual Information (NMI) metrics across the three networks. Statistical significance was assessed via one-sided Wilcoxon rank-sum tests. * $P < 0.05$, ** $P < 0.01$, *** $P < 0.001$, **** $P < 0.0001$; n.s., not significant.

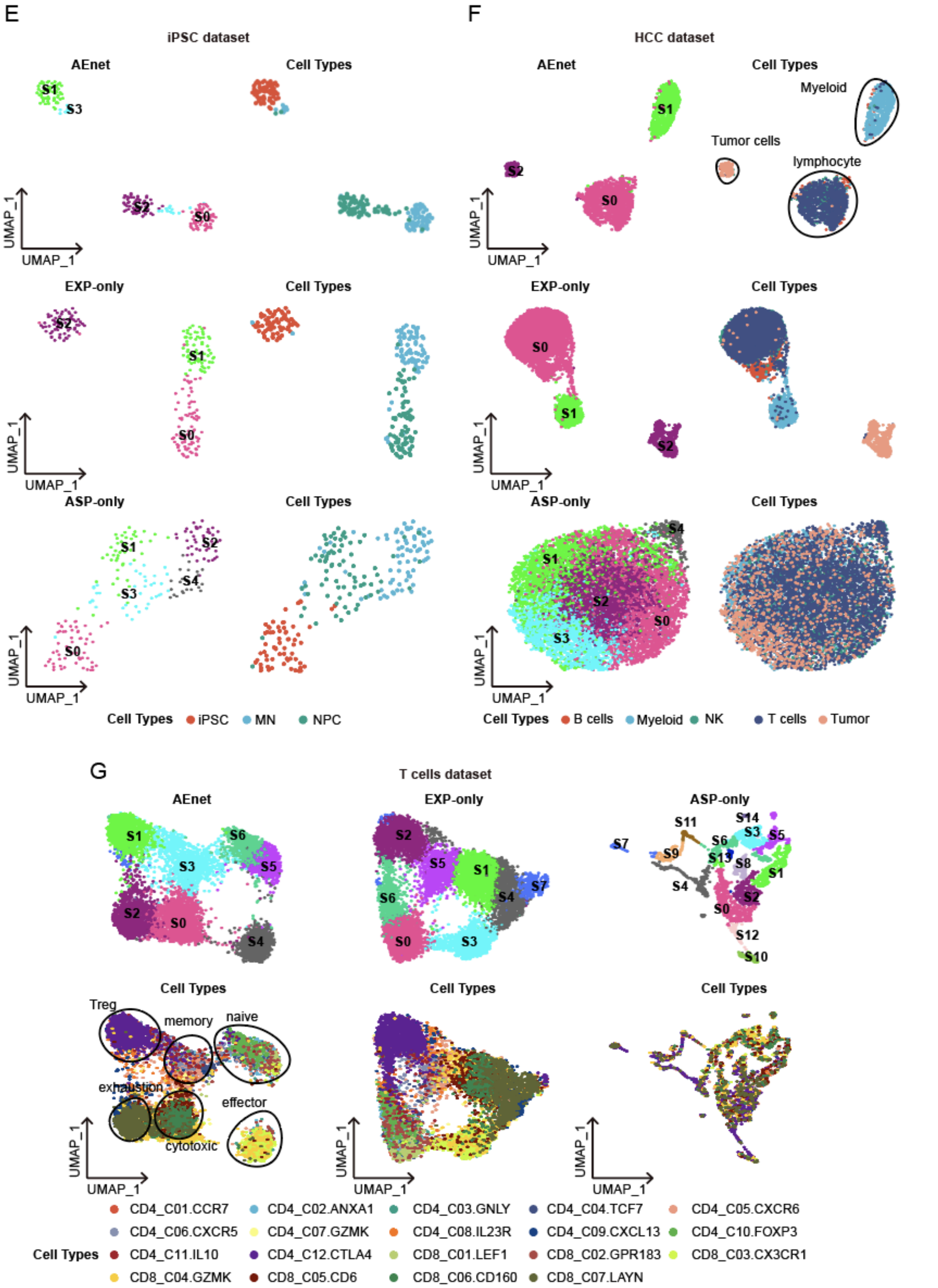

H

The description for PBMC dataset (Ding et al.)

The basic information

Sample Number	2
Cell Number	528
Average reads per cell	1,061,009
Platform	Smart-seq2 (PE 50)

The processed AS information

The number of ASP	3,202
The ASP-EXP links	6,524
The number of anchor ASPs	6
ASP Clusters	0

Figure R8. (E–F) Uniform Manifold Approximation and Projection (UMAP) visualizations showing the published cell type annotations (right) and the predicted clusters (left) derived from the ASP-EXP (AEnet) network (top), expression-only (EXP) network (middle), and ASP-only network (bottom) for (E) full-length iPSC and (F) hepatocellular carcinoma (HCC) single-cell RNA-seq datasets. **(G)** UMAP visualizations showing the published cell type annotations (botton) and the predicted clusters (upper) derived from the ASP-EXP (AEnet) network (left), expression-only (EXP) network (middle), and ASP-only network (right) for the T cells datasets. Clustering solutions shown represent those with the highest adjusted Rand index (ARI). **(H)** Detailed summary of the PBMC dataset.

3. The clustering capability of AEnet was compared against SCASL and Seurat by visually inspecting the clustering profile on reduced dimension space. While this initial visual-based inspection is sensible, an objective measurement could serve as an additional method of comparison. Two such metrices for clustering assessment are adjusted rand index (ARI) and normalised mutual information (NMI).

Response: Thank you for highlighting this important point. We fully agree that clustering performance should be evaluated using objective ground-truth metrics such as Adjusted Rand Index (ARI) and Normalized Mutual Information (NMI), rather than relying solely on visual inspection or improvements in batch correction. To this end, we have conducted comprehensive benchmarking analyses across three datasets—iPSC, T cell, and HCC—using published cell type annotations as ground truth.

AEnet consistently outperformed SCASL [8] in clustering accuracy across all datasets: iPSC (AEnet: 0.81 vs. SCASL: 0.37), T cell (0.42 vs. 0.29), and HCC (0.58 vs. 0.35) **(Fig. R9A–C)**. Moreover, AEnet produced more biologically meaningful and interpretable low-dimensional embeddings. For example, in the iPSC dataset, SCASL failed to distinguish iPSCs from NPCs, while AEnet accurately resolved these subpopulations **(Fig. R9A, D)**. In the HCC dataset, AEnet clearly separated lymphocytes, myeloid cells, and malignant epithelial cells, whereas SCASL blurred boundaries between major lineages **(Fig. R9B, E)**. Similarly, in the T cell dataset, AEnet identified functionally distinct subsets with high fidelity, in contrast to the

overlapping and poorly resolved clusters generated by SCASL **(Fig. R9C, F)**. We have incorporated this suggestion into the manuscript at **Page15, Line 325-342** and revised **Figure 2E-G** and **S8A-C**.

We also compared AEnet to Seurat [10] using ARI and NMI, applying the same cell–junction count matrix to ensure a fair comparison. AEnet consistently achieved superior clustering performance: in the iPSC dataset (AEnet: 0.81, Seurat: 0.81, scSHC: 0.82), HCC dataset (AEnet: 0.58, Seurat: 0.29, scSHC: 0.10), and T cell dataset (AEnet: 0.42, Seurat: 0.36, scSHC: 0.26) **(Fig. R10A–C)**. While Seurat performed comparably in the iPSC dataset, it produced overly complex and fragmented embeddings in the T cell and HCC datasets **(Fig. R10D–F)**. Notably, Seurat generated several small clusters driven by junctions from specific genes—such as ACTG1—that reflected differential gene expression rather than true splicing heterogeneity **(Fig. R10G)**. This suggests Seurat may be suboptimal for identifying isoform-level variation.

Taken together, these results demonstrate that AEnet not only outperforms SCASL but also provides more accurate, biologically meaningful clustering than general-purpose methods like Seurat and scSHC, particularly in detecting alternative splicing-driven cellular heterogeneity.

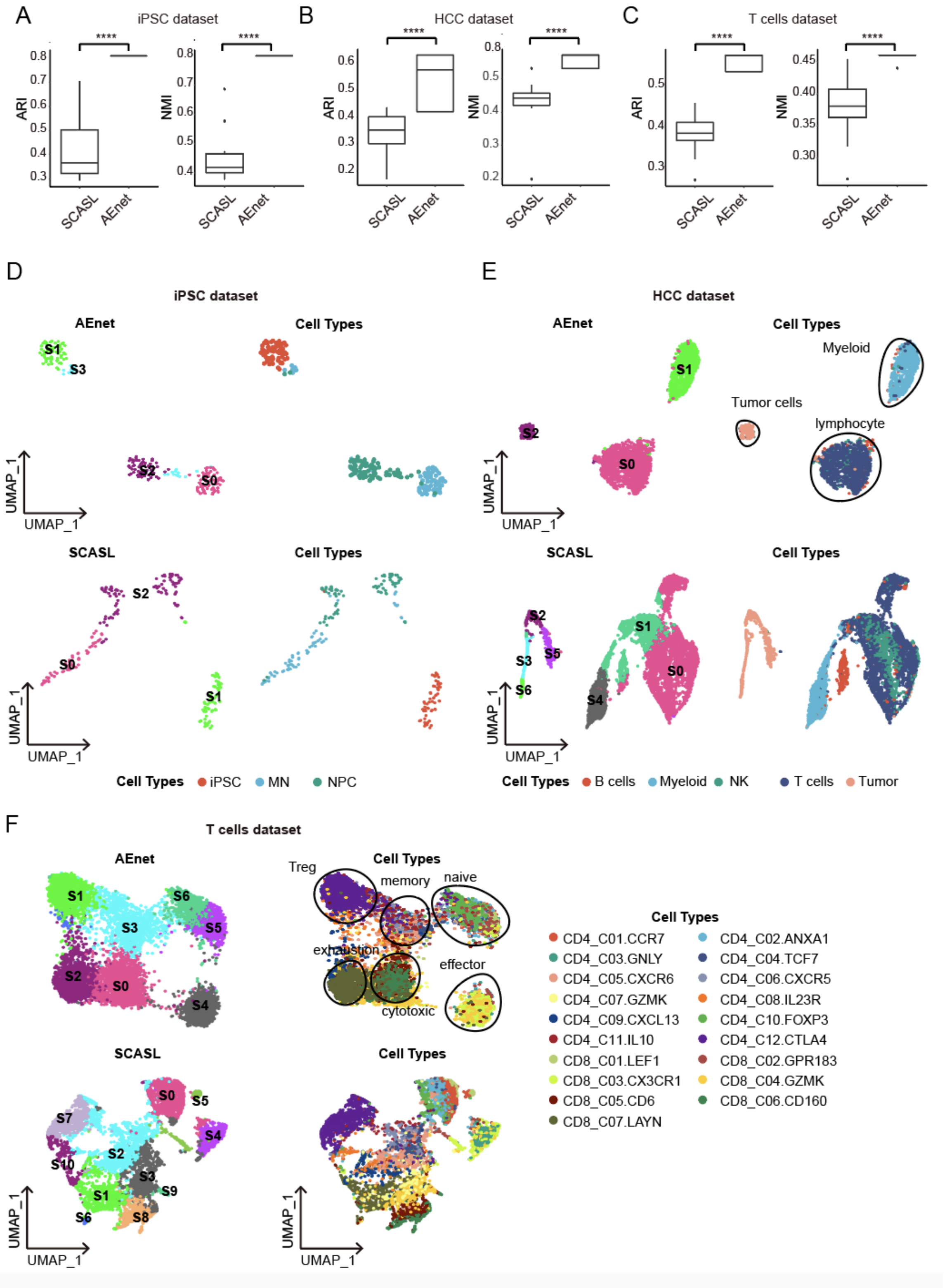

Figure R9. (A-C) Quantitative benchmarking of clustering concordance using (left) ARI and (right) NMI metrics of AEnet and SCASL. Statistical significance was assessed via one-sided Wilcoxon rank-sum tests. **(D–F)** UMAP visualizations showing the published cell type annotations (right) and the predicted clusters (left) derived from AEnet (upper) and SCASL (botton) for: (D) full-length iPSC (E) hepatocellular carcinoma (HCC) single-cell RNA-seq datasets, and the (F) T cell

dataset. Clustering solutions shown represent those with the highest adjusted Rand index (ARI). * P < 0.05, ** P < 0.01, *** P < 0.001, **** P < 0.0001; n.s., not significant.

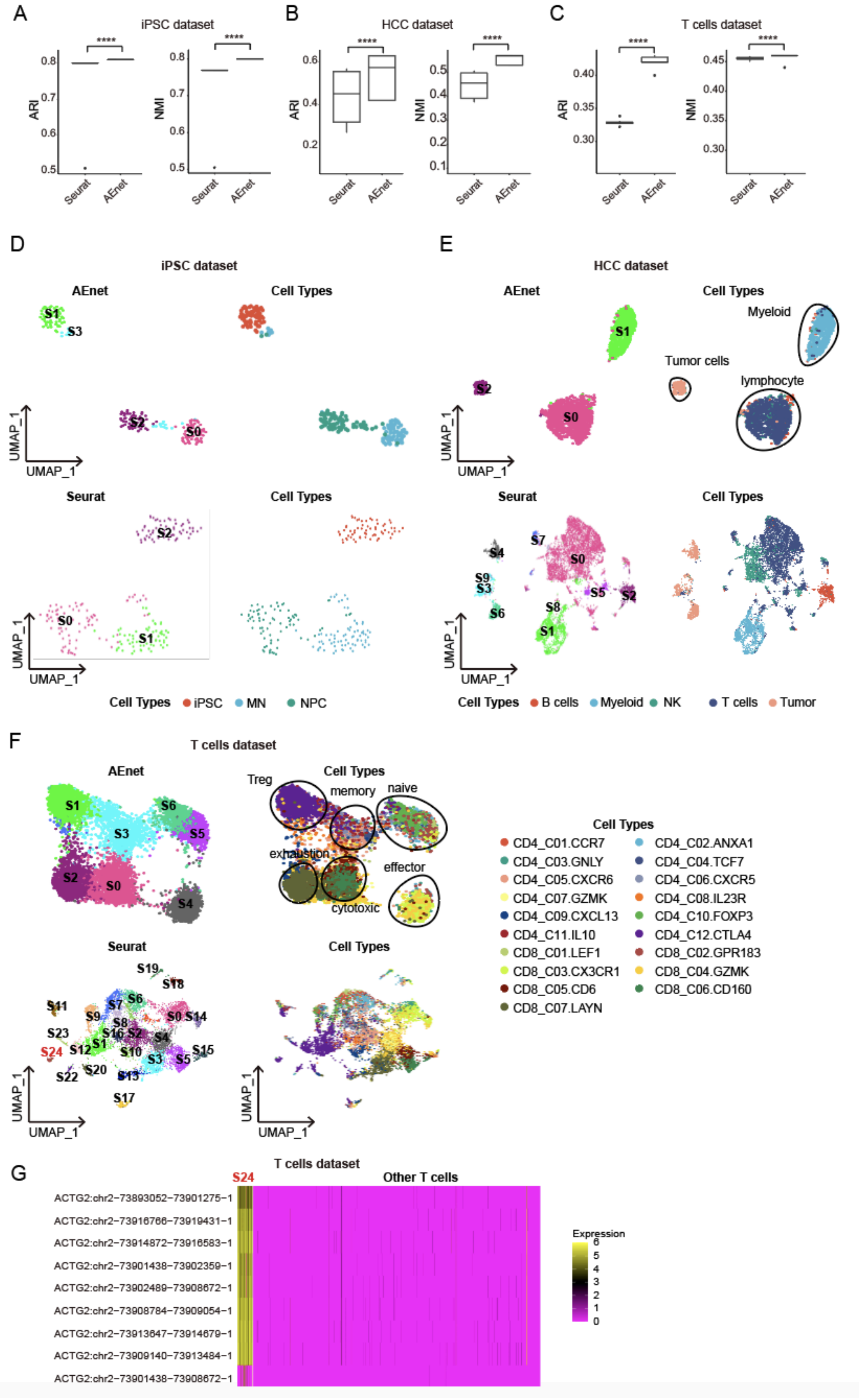

Figure R10. (A-C) Quantitative benchmarking of clustering concordance using (left) ARI and (right) NMI metrics of AEnet and Seurat. Statistical significance was assessed via one-sided Wilcoxon rank-sum tests. **(D–F)** UMAP visualizations showing the published cell type annotations (right) and the predicted clusters (left) derived from AEnet (upper) and Seurat (botton) for: (D) full-length iPSC (E) hepatocellular carcinoma (HCC) single-cell RNA-seq datasets, and the (F) T cell dataset. Clustering solutions shown represent those with the highest adjusted Rand index (ARI). **(G)** The differentially junction for S24 in the T cells dataset. * $P < 0.05$, ** $P < 0.01$, *** $P < 0.001$, **** $P < 0.0001$; n.s., not significant.

4. The ability of AEnet to mitigate batch effect was compared against Seurat and SCASL. For Seurat specifically, the batch effect was "corrected" using the "FindIntegrationAnchors" and "IntegrateData" functions. These functions integrate multiple samples, but do not adjust for batch effect per se. To enable a fair comparison with Seurat, or other means of batch correction, more suitable approaches for adjusting batch effect should be benchmarked against. The canonical correlation analysis (CCA) and Harmony are two batch correction approaches implemented by Seurat that may be benchmarked against.

Response: We thank the reviewer for the insightful comment regarding the need for more suitable batch correction baselines. In response, we performed benchmarking comparisons to assess the ability of AEnet to mitigate batch effects, particularly in the context of splicing heterogeneity. To this end, we applied commonly used batch correction methods—Seurat CCA [10] and Harmony [11]—to the cell–junction count matrix prior to PSI computation, ensuring fair comparison **(Fig. R11A–C)**. To ensure a fair comparison, we applied two widely used batch correction methods—Seurat CCA and Harmony—to the cell–junction count matrix prior to PSI computation **(Fig. R11A-D)**. However, these methods are primarily optimized for gene expression data and are not well-suited for alternative splicing analysis. In particular, they lack the sensitivity to detect key splicing events and fail to capture splicing-driven cellular heterogeneity. Consequently, AEnet outperformed both methods in identifying biologically meaningful cell heterogeneity, achieving significantly higher ARI and NMI scores **(Fig. R11A-B)**. Moreover, CCA and Harmony tended to overcorrect, resulting in fragmented clusters driven by expression noise (e.g., ACTG1-associated mini-clusters) rather than true alternative splicing patterns **(Fig. R11C-D)**. By contrast, AEnet successfully preserves AS-based clustering structure and captures shared splicing variation across multiple samples or conditions, compared to SCASL

(Fig. R5E–H). In summary, AEnet offers a splicing-aware alternative to general batch correction methods, demonstrating superior performance in preserving biologically relevant AS variation across diverse datasets.We have incorporated this suggestion into the manuscript at Page 15-16, Line 343-353 and the revised Figure S9A-D.

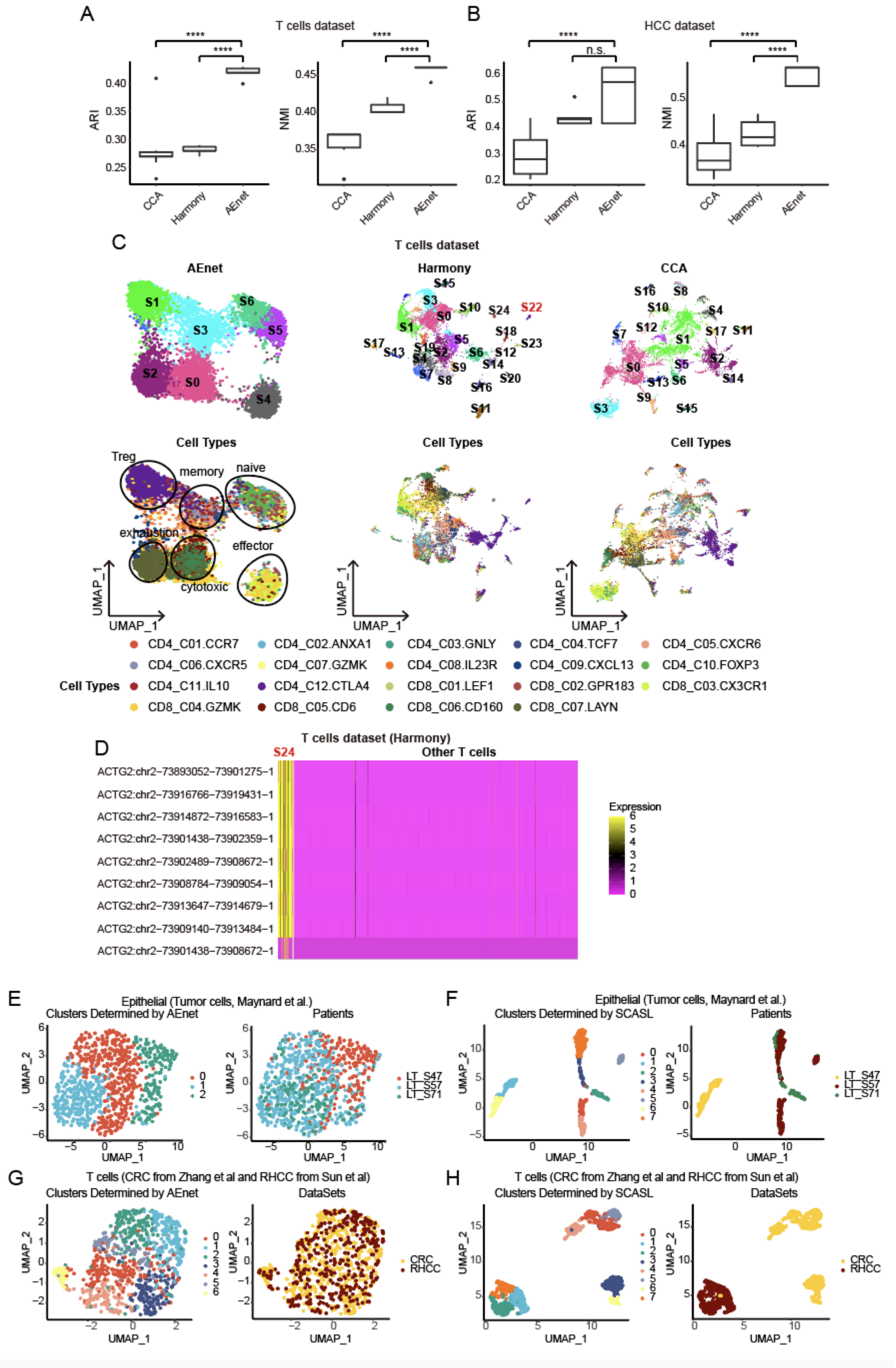

Figure R11. (A-B). Quantitative benchmarking of clustering concordance using **(A)** Adjusted Rand Index (ARI) and **(B)** Normalized Mutual Information (NMI) metrics across integration methods. **(C)** UMAP visualizations showing the published cell type annotations (botton) and the predicted clusters (upper) derived from AEnet (left), Harmony (middle), and CCA (right) for the T cells datasets. Clustering solutions shown represent those with the highest adjusted Rand index (ARI). **(E)** The differentially junction for S22 in the T cells dataset. **(E-F)** UMAP shows the clustering of cell types determined by AEnet **(E)** and SCASL **(F)** (left panel) and patient clustering (right panel) for epithelial cells from multiple patients. **(G-H).** UMAP shows the clustering of cell types determined by AEnet **(G)** and SCASL **(H)** (left panel) and patient clustering (right panel) for T cells from the CRC (Colorectal cancer) and RHCC (Recurrent Hepatocellular carcinoma) datasets. Statistical significance was assessed via one-sided Wilcoxon rank-sum tests * $P < 0.05$, ** $P < 0.01$, *** $P < 0.001$, **** $P < 0.0001$; n.s., not significant

5. It would be of particular interest to benchmark AEnet against other splicing-based clustering approaches. The variational autoencoder (VAE) algorithm implemented by scQuint (PMID: 35229721) and DOLPHIN (https://apc01.safelinks.protection.outlook.com/?url=https%3A%2F%2Fwww.researchsquare.com%2Farticle%2Frs-5474597%2Fv1&data=05%7C02%7Cwuliang%40genomics.cn%7C19524fec513b46a5e91e08dd65e932ef%7C853aa2281adc4d91bb286065c1e9963d%7C0%7C0%7C638778774818200358%7CUnknown%7CTWFpbGZsb3d8eyJFbXB0eU1hcGkiOnRydWUsIlYiOiIwLjAuMDAwMCIsIlAiOiJXaW4zMiIsIkFOIjoiTWFpbCIsIldUIjoyfQ%3D%3D%7C0%7C%7C%7C&sdata=YxkFdnbgoIhPFzDFAkxTMiwh1cM8PlxZo58M7e2jgco%3D&reserved=0) have demonstrated convincing clustering ability.

Response: We thank the reviewer for this important suggestion. To benchmark AEnet against other splicing-based clustering methods, we evaluated its performance relative to scQuint [16] and DOLPHIN using ARI and NMI as objective metrics.

As scQuint primarily employs a variational autoencoder (VAE) for embedding, we performed clustering on the scQuint-derived embeddings using default settings, with the cell–junction count matrix as input **(Fig. R12A)**. In the iPSC dataset, which presents relatively simple cellular composition, AEnet and scQuint achieved comparable embedding quality **(Fig. R12B)**. However, in the more complex T cell dataset and HCC datasets, AEnet effectively resolved well-defined functional subsets and major lineage **(Fig. R12C-D)**. In contrast, scQuint yielded overlapping

clusters and failed to distinguish several key subpopulations **(Fig. R12C-D)**. We further compared AEnet with DOLPHIN using three benchmark datasets provided by the DOLPHIN study, as BAM files required for exon count matrix construction were unavailable for our own datasets **(Fig. R12E)**. Across all three datasets, AEnet demonstrated clustering accuracy comparable to that of DOLPHIN. Specifically, AEnet achieved an ARI of 0.606 in the PBMC dataset, 0.78 in the PDAC dataset, and 0.43 in the Colon dataset, closely matching DOLPHIN's performance **(Fig. R12F–G)**. In addition, AEnet produced informative low-dimensional embeddings across all three datasets, similar to DOLPHIN **(Fig. R12H–J)**.

Collectively, AEnet outperforms scQuint in resolving fine-grained cellular subtypes and matches DOLPHIN's clustering accuracy across diverse benchmark datasets, demonstrating both its robustness and generalizability for splicing-aware single-cell analysis. We have incorporated this suggestion into the manuscript at Page15, Line 325-342 and revised Figure S8D.

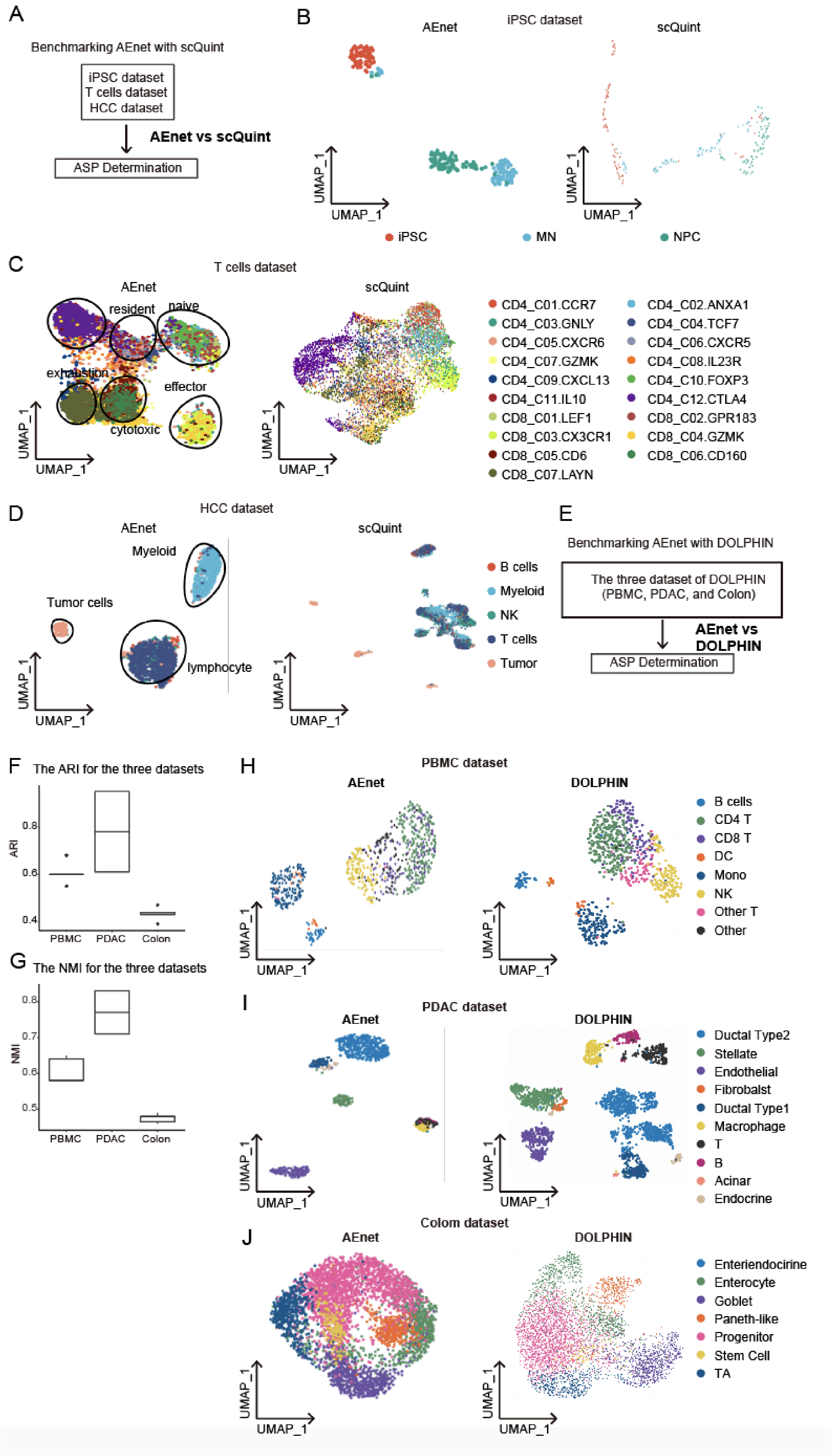
A
Benchmarking AEnet with scQuint
iPSC dataset
T cells dataset
HCC dataset
AEnet vs scQuint
ASP Determination
B
iPSC dataset
AEnet
scQuint
iPSC
MN
NPC
C
T cells dataset
AEnet
scQuint
resident
naive
exhaustion
effector
cytotoxic
CD4_C01.CCR7
CD4_C02.ANXA1
CD4_C03.GNLY
CD4_C04.TCF7
CD4_C05.CXCR6
CD4_C06.CXCR5
CD4_C07.GZMK
CD4_C08.IL23R
CD4_C09.CXCL13
CD4_C10.FOXP3
CD4_C11.IL10
CD4_C12.CTLA4
CD8_C01.LEF1
CD8_C02.GPR183
CD8_C03.CX3CR1
CD8_C04.GZMK
CD8_C05.CD6
CD8_C06.CD160
CD8_C07.LAYN
D
HCC dataset
AEnet
scQuint
Myeloid
Tumor cells
lymphocyte
B cells
Myeloid
NK
T cells
Tumor
E
Benchmarking AEnet with DOLPHIN
The three dataset of DOLPHIN (PBMC, PDAC, and Colon)
AEnet vs DOLPHIN
ASP Determination
F
The ARI for the three datasets
ARI
PBMC
PDAC
Colon
G
The NMI for the three datasets
NMI
H
PBMC dataset
AEnet
DOLPHIN
B cells
CD4 T
CD8 T
DC
Mono
NK
Other T
Other
I
PDAC dataset
Ductal Type2
Stellate
Endothelial
Fibrobalst
Ductal Type1
Macrophage
T
B
Acinar
Endocrine
J
Colom dataset
Enteriendocirine
Enterocyte
Goblet
Paneth-like
Progenitor
Stem Cell
TA
UMAP_1

Figure R12. (A) Schematic of the assessment pipeline for AEnet and scQuint. **(B-D)** Uniform Manifold Approximation and Projection (UMAP) visualizations demonstrate the relative performance of AEnet (left) and scQuint (right) in: (B) full-length iPSC dataset, (C) T cells sequencing data, and (D) hepatocellular carcinoma (HCC) single-cell RNA-seq data. **(E)** Schematic illustration of the assessment pipeline used to compare AEnet and DOLPHIN. **(F–G)** Quantitative benchmarking of clustering concordance using (E) ARI and (F) NMI metrics across the three datasets. **(H–J)** Uniform Manifold Approximation and Projection (UMAP) visualizations showing the clustering performance of AEnet (left) and DOLPHIN (right) across the three DOLPHIN benchmark datasets: PBMC (H), PDAC (I), and Colon (J).

6. I appreciate the detailed demonstration of AEnet on tumour-infiltrating T cells and embryonic gastrulation using AEnet, and the identification of regulatory splicing factors in these scenarios. In haematological malignancies such as myelodysplastic syndrome (MDS) and myeloproliferative neoplasm (MPN), more than half of the patients have mutations in splicing factors, namely SF3B1, SRSF2, and U2AF1, and therefore are known to have dysregulated splicing profiles. Moreover, several AS events related to these splicing factors have already been experimentally validated, for example, SEPTIN2 and MAP3K7 are mis-spliced in SF3B1-mutant MDS patients. These splicing events may serve as positive controls in benchmarking exercise. Furthermore, related to comment no. 2 above, the haematopoietic compartment consists of a wide variety of cell types suitable for showcasing AEnet functionalities. Therefore, these cancer types are biologically relevant for benchmarking and for demonstrating the application of AEnet. One such example is a dataset that consists of SF3B1-mutant MDS samples (PMID: 37582363).

Response: We sincerely thank the reviewer for the valuable suggestion regarding the use of SF3B1-mutant myelodysplastic syndrome (MDS) datasets (e.g., PMID: 37582363) for benchmarking AEnet. We fully agree that hematological malignancies such as MDS and myeloproliferative neoplasms (MPN), which frequently harbor mutations in core splicing factors (e.g., SF3B1, SRSF2, U2AF1), represent highly relevant biological systems for investigating alternative splicing dysregulation. Moreover, several mis-spliced events associated with these mutations—such as those in SEPTIN2 and MAP3K7—have been experimentally validated and could serve as useful positive controls for benchmarking splicing-aware models.

To preliminarily explore this direction, we applied AEnet to the dataset referenced in PMID: 37582363 [17]. AEnet identified a total of 163,921 alternative splicing patterns (ASPs), including 424 anchor ASPs and 1,620 anchor genes. The resulting clustering revealed clear correspondence with known hematopoietic subpopulations,

such as erythroid progenitors (EP), megakaryocyte-erythroid progenitors (MEP), megakaryocyte progenitors (MKP), hematopoietic stem and progenitor cells (HSPCs), dendritic cells (DCs), and mature monocytes (Mat_Mono) **(Fig. R13A)**. Quantitative benchmarking confirmed the robustness of AEnet in this context, yielding a median Adjusted Rand Index (ARI) of 0.525 and a Normalized Mutual Information (NMI) of 0.5 **(Fig. R13B)**. These findings support AEnet's ability to resolve complex cellular heterogeneity in clinically relevant, splicing-perturbed systems.

We next examined the distribution of wild-type (WT) and mutant (MUT) cells in MDS patients and found that WT cells were enriched in cluster S2, whereas MUT cells were predominantly distributed across other clusters—consistent with findings from the original study **(Fig. R13C)** [17]. Notably, the C_7 ASP cluster was specifically enriched in MUT cells **(Fig. R13D)**. The splicing factor most strongly associated with this cluster was HNRNPA1 **(Fig. R13E),** which regulates the alternative splicing of *Arhgap1*. This splicing event leads to Cdc42 activation and has been implicated in hematopoietic defects in HSPCs, contributing to the pathogenesis of MDS, as previously reported [18].

Due to time constraints and in order to maintain the manuscript's conceptual focus, we have not incorporated this analysis into the main text. However, we recognize its biological and translational importance and plan to include a more comprehensive exploration of this dataset in future versions of the study. We will also make the associated analysis scripts publicly available alongside the AEnet package to support reproducibility and further exploration by the community.

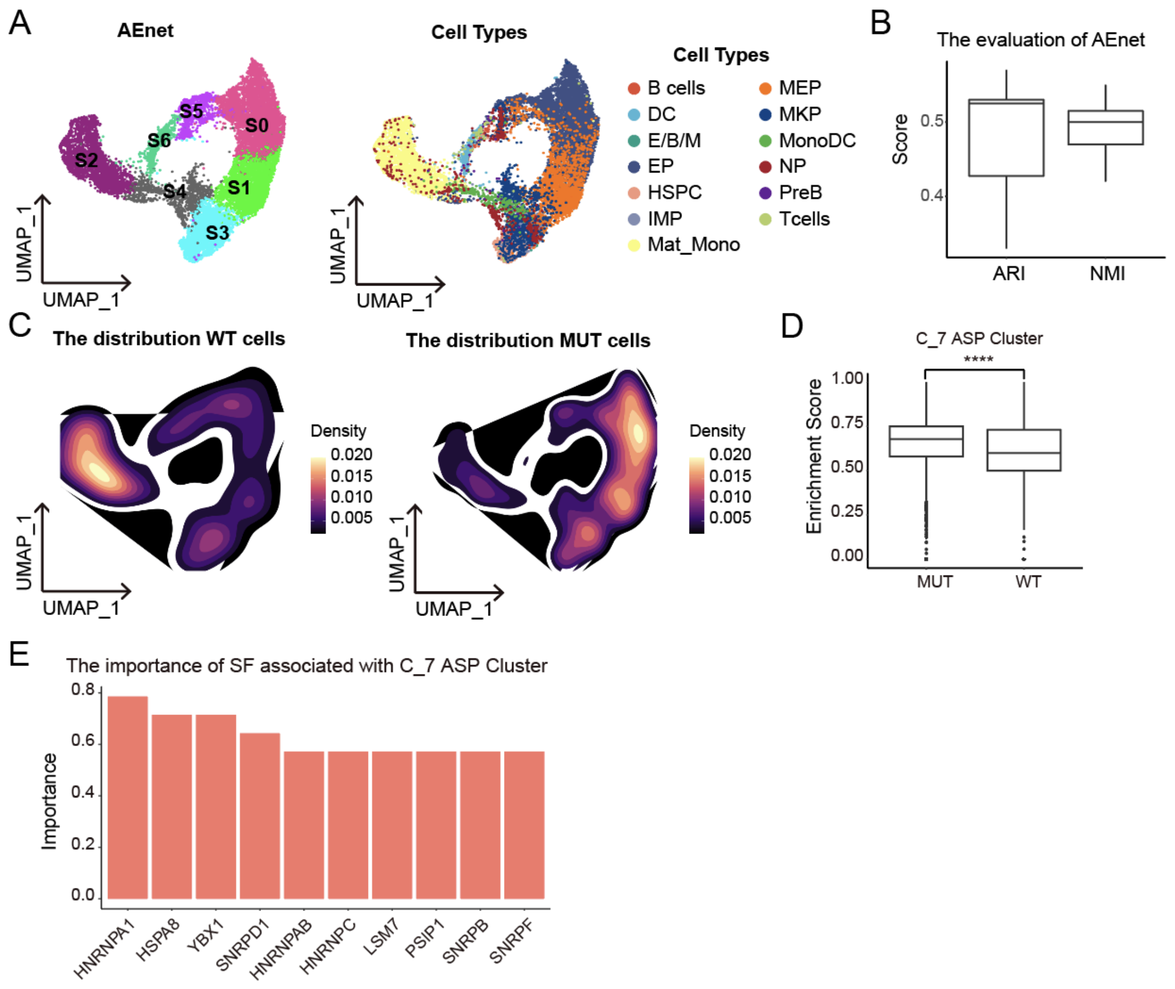

Figure R13. (A) UMAP visualizations showing the published cell type annotations (right) and the predicted clusters (left) derived from AEnet (left). Clustering solutions shown represent those with the highest adjusted Rand index (ARI). **(B)** Quantitative benchmarking of clustering performance using ARI and NMI, evaluated against the ground truth annotations of the MDS dataset. **(C)** The distribution of SF3B1 WT and MUT cells in the MDS patients. **(D)** The distribution of enrichment scores for C_7 ASP clusters. **(E)** The importance of splicing factors associated with C_7 ASP Clusters.

7. The cornerstone of single-cell RNA-sequencing analysis is differential gene and splicing analysis. AEnet excels in revealing regulatory splicing factors, isoforms, and pathways within a cluster or condition. However, AEnet does not enable comparison across different clusters or conditions, e.g., disease versus healthy states. While the lack of this functionality is not a limitation in itself, this may be detailed in the Discussion to aid users in deciding which single-cell AS tools will be best suited to meet their research goals.

Response: We thank the reviewer for this thoughtful and constructive comment. AEnet is designed to integrate alternative splicing and gene expression data to uncover regulatory splicing factors, isoforms, and pathway activities in a splicing-aware manner. Its current focus lies in intra-condition analyses—namely, characterizing splicing heterogeneity, regulatory programs, and functional consequences within a single biological context, such as a specific sample or condition.

As correctly noted by the reviewer, AEnet is not currently designed for direct differential splicing comparisons across clusters or conditions (e.g., disease vs. healthy states). This is primarily because AEnet aims to identify shared and context-enriched splicing programs across cells, rather than testing for statistical differences between predefined groups. Nevertheless, the anchor ASPs and regulatory modules identified by AEnet could serve as informative features for downstream comparison using complementary differential splicing tools (e.g., MARVEL, MAJIQ-SC), particularly when group-level comparisons are desired. A detailed comparison of these functionalities across methods is provided in Table R4.

We agree that this functional scope should be made explicit for users when choosing the most suitable tool for their research objectives. Accordingly, we have revised the Discussion to clarify that AEnet is primarily intended for unsupervised discovery of splicing heterogeneity and its regulatory mechanisms within a single condition or sample. For tasks requiring group-wise differential splicing analysis, users may consider integrating AEnet's outputs with dedicated statistical testing frameworks. We thank the reviewer again for this valuable suggestion, which has helped improve the clarity and usability of our work. We have incorporated this suggestion into the manuscript at **Page32, Line 710-724** and **Table S1**.

Softwares/Steps	SCASL	MARVEL	DESJ-detection	Expenditure (Outrigger)	BRIE	AEnet
quantitativeness	√	√	√	√	√	√

imputation	√	/	/	/	√	/
feature selection	/	/	/	/	/	√
dimension reduction	√	√	/	/	/	√
cell clustering	√	/	/	/	/	√
Differentially Splicing events Detection	√	√	√	√	√	/
Modality classification	/	√	/	√	/	/
RNA velocity	/	/	/	/	√	/
Splicing Factors prediction	/	/	/	/	/	√
Pathways affected by single splicing event prediction	/	/	/	/	/	√
Pathways enriched in differentially splicing genes	/	√	/	/	/	/
Nonsense-mediated decay prediction	/	√	/	/	/	/
RNA-sequencing platform	a	a&b	a	a	a	a

a, Plate-based sequencing; b, Droplet-based

Table R4. Summary of methods

8. For the purpose of reproducibility and to convince prospective users, like myself, to use AEnet, please may the authors provide the codes used to generate AEnet-related figures in the manuscript. A R Markdown format is highly encouraged, if possible.

Response: We sincerely thank the reviewer for this constructive suggestion. In response, we have updated our R package, which is now publicly available at: https://github.com/liushang17/AEnet. To facilitate reproducibility and help users get started, we also provide demonstration datasets corresponding to two biological systems:

- iPSC dataset: https://drive.google.com/file/d/1Qkg4De3DER4Qs5V_vP-M7GgwprwjwkLA/view?usp=drive_link
- T cell dataset: https://drive.google.com/file/d/1zuut5OlYgFeYXytU5kUZsYKbx07CKb56/view?usp=drive_link

We fully agree that providing the code used to generate manuscript figures is essential for transparency and reproducibility. To that end, we are currently preparing a set of R Markdown scripts that will reproduce the key figures in the manuscript using the demonstration datasets. These materials will be made publicly available via the GitHub repository in the near future. In the meantime, we are happy to share preliminary versions of the scripts upon request. We appreciate the reviewer's interest in using AEnet and will prioritize making these resources as accessible and user-friendly as possible.

Minor comments:

9. Please may the authors provide the pre-defined list of splicing factors in a Supplementary Table.

Response: Thanks for pointing this out. We have provided the pre-defined list of splicing factors in **Supplementary Table S4**.

10. In paragraph 1 of the Introduction, "… analyzing profilings of AS…" should be "…analyzing profiles of AS…".
Response: We thank the reviewer for their attentive reading and valuable feedback. The phrase "analyzing profilings of AS" has been corrected to "analyzing profiles of AS" in Paragraph 1 of the Introduction at **Page 2, Line 59-60**.

11. In paragraph 2 of the Introduction, the authors alluded to the inflated false negatives exemplified by published single-cell AS tools. Has this limitation been demonstrated or investigated for these tools? If not, I recommend the authors to tone down this assertion.
Response: We sincerely thank the reviewer for this correction. Upon re-evaluation, we recognize that our original statement overgeneralized the limitations of existing tools without direct benchmarking evidence. We will revise the text on **Page 3, Lines 70–75** as follows:
'Although there are several bioinformatic approaches to studying single-cell AS profiling, most are based on canonical RNA-based clustering into cell types and then comparing AS heterogeneity, similar to differential gene expression analysis, between these predefined cell types. However, cell types defined by AS can differ substantially from those defined by gene expression, which may result in incomplete detection of splicing heterogeneity.'

12. In paragraph 2 of the Introduction, the authors also mentioned that current single-cell AS tools do not identify functional pathways. But MARVEL has a functionality to identify enriched functional pathways (PMID: 36631981).
Response: We sincerely thank the reviewer for pointing out that MARVEL has a functionality to identify enriched functional pathways. We acknowledge our oversight in the original introduction and appreciate the correction. We have revised the Introduction to reflect MARVEL's capabilities in performing gene ontology (GO) enrichment analysis using the clusterProfiler R package, which can identify enriched pathways among differentially spliced genes. We have incorporated this suggestion into the manuscript at **Page3, Line 80-85**.

13. In paragraph 3 of the Introduction, the authors listed three challenges of single-cell AS analysis. Please may the authors provide the relevant citations for each of these challenges.
Response: We thank the reviewer for raising this important point. We have added relevant citations [19–21] for each challenge, respectively.

Reviewer #3: In this work, Liu & Chen et al. present AEnet, a computational method for analyzing alternative splicing (AS) at the single-cell level. By integrating AS and scRNA-seq data, they identify alternative splicing pattern (ASP) clusters and use them to define cell subpopulations. The authors demonstrate the utility of their method in three biological contexts: tumor immunotherapy response,

tumor-infiltrating T cells, and embryonic development. This work integrates two distinct but complementary data modalities, AS and scRNA-seq, into a single framework, improving the characterization of cellular heterogeneity. The method is well-designed and effectively applied, but several issues should be addressed before publication.

Major points:

* Retaining NaNs for zero counts is valid, but even low-count ASPs may introduce random fluctuations in PSIs. Have you tested different count thresholds? And how does it affect results? If an ASP is rare and mostly NaN, how do you decide whether to include it? Could this introduce bias against infrequent but biologically relevant splicing events?

Response: We thank the reviewer for this insightful question. We fully agree that low-count ASPs can introduce noise into PSI estimation, potentially leading to spurious ASP-Exp links and misidentification of anchor ASPs **(Fig. R14A)**. To systematically evaluate the impact of count thresholds, we tested a range of minimum read count cutoffs: 0, 3, 5, 7, and 9. We assessed their influence on ASP-Exp link quantity, specificity, and anchor ASP detection **(Fig. R14A)**.

Setting the threshold to 0 yielded the highest number of ASP-Exp links, with ~40% classified as "specific." However, 99% of these specific links were supported by fewer than two samples, suggesting they were likely artifacts of random fluctuations rather than meaningful biological associations **(Fig. R14B-C)**. Similarly, the 0-read threshold also led to an inflated number of anchor ASPs, most of which were linked to low-confidence, sample-specific signals **(Fig. R14D)**. By contrast, thresholds of ≥3 substantially reduced these spurious associations. Moreover, most biologically meaningful ASP-Exp links were retained when thresholds >0 were applied **(Fig. R14E)**. Based on these results, AEnet uses a default threshold of five supporting reads to ensure PSI robustness while minimizing noise.

We also appreciate the reviewer's point about potential bias against rare but biologically relevant splicing events. In AEnet, an ASP is considered "valid" in a sample only if it is supported by ≥5 reads across ≥20 cells. ASPs failing this criterion are excluded from downstream analysis, as their sparsity compromises the reliability of similarity estimates between splicing and expression. To assess whether this filtering excludes informative low-abundance ASPs, we stratified all ASPs into five categories based on their support across cells:

- Invalid: ≤20 cells (excluded)
- Type 1: >20–30 cells
- Type 2: >30–40 cells
- Type 3: >40–50 cells
- Type 4: >50 cells

An ASP was assigned to the highest applicable category if it met the criteria in ≥3 samples **(Fig. R14F)**. As expected, higher-support ASPs (fewer NaNs) showed stronger ASP-Exp associations. Nonetheless, approximately 30% of Type 1 ASPs—representing relatively rare but retained events—still showed significant correlations with gene expression, and four were identified as anchor ASPs **(Fig. R14G–H)**, underscoring their potential functional relevance, albeit at a lower frequency than higher-support types. In summary, our results support the use of both read count and sample support thresholds to reduce noise while preserving biological signal. AEnet remains capable of capturing meaningful but infrequent ASPs and provides user-defined thresholding to support flexible analysis tailored to specific research goals. We have incorporated this suggestion into the manuscript at **Page 9-10, Line 224-255 and revised Figure S4**.

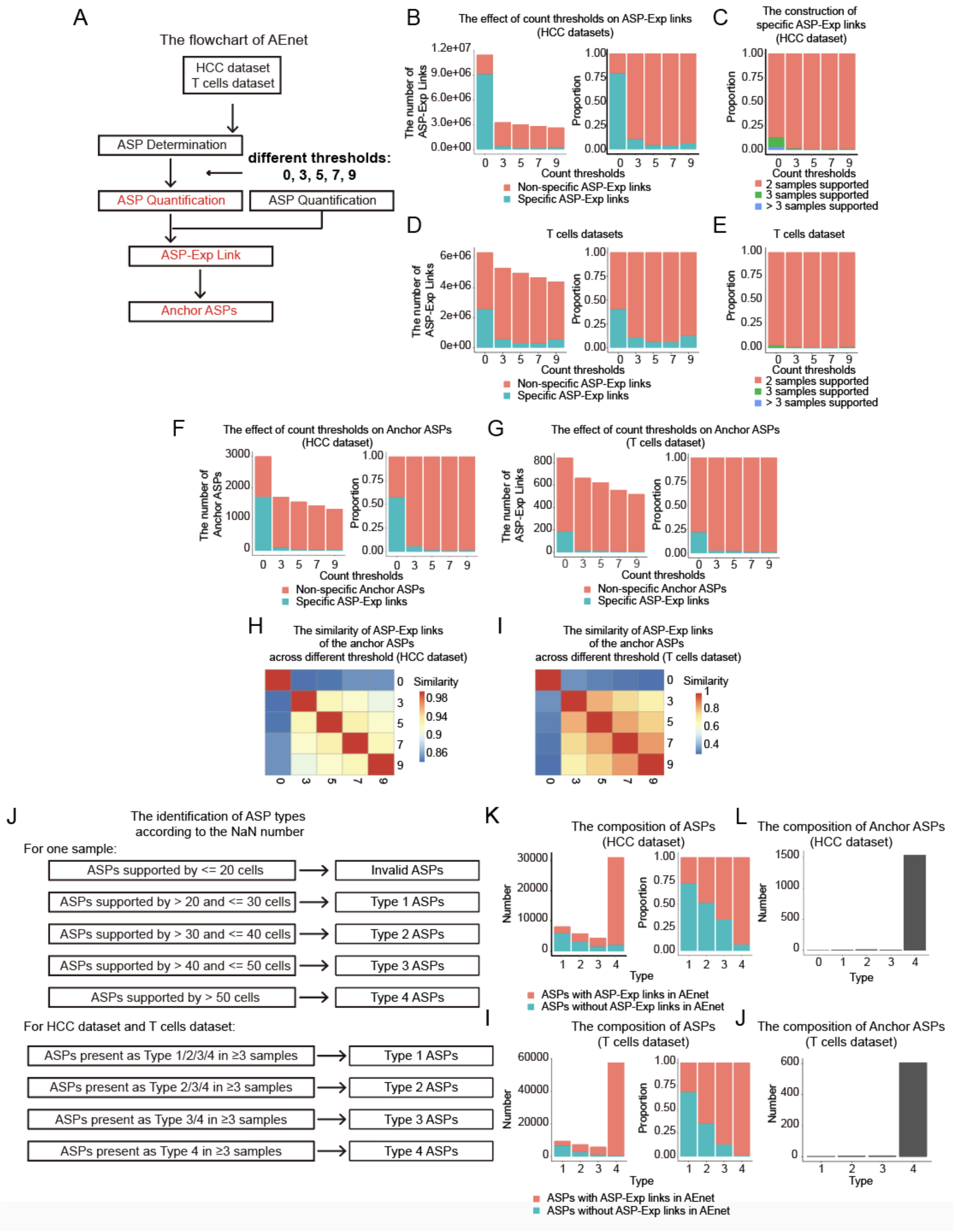

Figure R14. (A) Schematic overview of benchmarking strategy for evaluating different count thresholds. **(B)** Quantification of ASP-EXP links across varying count thresholds, with color coding denoting link specificity. **(C)** Patient-level validation rates for specific ASP-EXP links at different expression count cutoffs. **(D)**

Quantification of anchor ASPs across varying count thresholds, with color coding denoting link specificity. **(E)** The similarity of anchor ASPs across different expression count cutoffs. **(F)** Schematic Diagram of the identification of ASP types for one sample or one dataset. **(G)** Quantitative analysis of ASP subtypes categorized by presence (blue) or absence (gray) of ASP-EXP links. **(H)** Frequency distribution of anchor ASPs stratified by ASP subtype classification.

* The cell populations identified by ASP are demonstrated to be independent of RNA-based clustering. Have you directly compared ASP-only, RNA-only, and joint clustering to assess whether they provide unique biological insights?

Response: We appreciate your insightful comment. To serve the question, three independent datasets—iPSC [2], T cell [6], and HCC [7]—were used for evaluation, and detailed information is provided in **Table R4**. Comprehensive ablation analyses using published cell type annotations as ground truth demonstrated that the joint ASP-EXP model (AEnet) consistently outperformed the standalone AS-only (Anet) and expression-only (Enet) approaches **(Fig. R15A)**. Specifically, the median ARI scores were 0.81 (AEnet), 0.68 (Anet), and 0.77 (Enet) for the iPSC dataset; 0.58 (AEnet), 0.10 (Anet), and 0.39 (Enet) for the HCC dataset; and 0.42 (AEnet), 0.10 (Anet), and 0.32 (Enet) for the T cell dataset **(Fig. R15B–D)**. These results demonstrate that integrating ASP features with gene expression markedly improves clustering resolution and biological interpretability. Furthermore, AEnet generated the most informative low-dimensional embeddings across all datasets **(Fig. R15E–G)**, accurately capturing cellular architecture in iPSCs, delineating major lineages in HCC, and resolving functionally distinct T cell subsets—capabilities that were limited or absent in the AS-only and EXP-only models. Thus, AEnet consistently achieves superior performance by jointly leveraging alternative splicing and gene expression to reveal biologically meaningful cellular heterogeneity. We have incorporated this suggestion into the manuscript at **Page15, Line 311-324** and revised **Figure 2A-D and S7**.

dataset	Sample Number	Cell Number	Average reads per cell	Platform	Cell Types
iPSC dataset2	1	191	11,932,904	Smart-seq2 (PE 100)	iPSCs (62), NPCs (69), and MNs (60)
HCC dataset	19	16,963	12,633,167	Smart-seq2 (PE 100)	Tumor cells (2737), Epi (34), Endo (99), HSC (119), pDC (47), Plasma (83), B cell (819), Myeloid (3293), Tcell (7294), Plasma (83), and NK (1947)
T cels dataset	12	8,530	1,250,000	Smart-seq2 (PE 100)	CD4_C01.CCR7 (472), CD4_C02.ANXA1 (509), CD4_C03.GNLY (170), CD4_C04.TCF7 (331), CD4_C05.CXCR6 (639), CD4_C06.CXCR5 (216), CD4_C07.GZMK (204), CD4_C08.IL23R (229), CD4_C09.CXCL13 (272), CD4_C10.FOXP3 (365), CD4_C11.IL10 (176), CD4_C12.CTLA4 (1319), CD8_C01.LEF1 (164), CD8_C02.GPR183 (155), CD8_C03.CX3CR1 (773), CD8_C04.GZMK (840), CD8_C05.CD6 (431), CD8_C06.CD160 (363), CD8_C07.LAYN (831)

Table R5. Summary of the three datasets

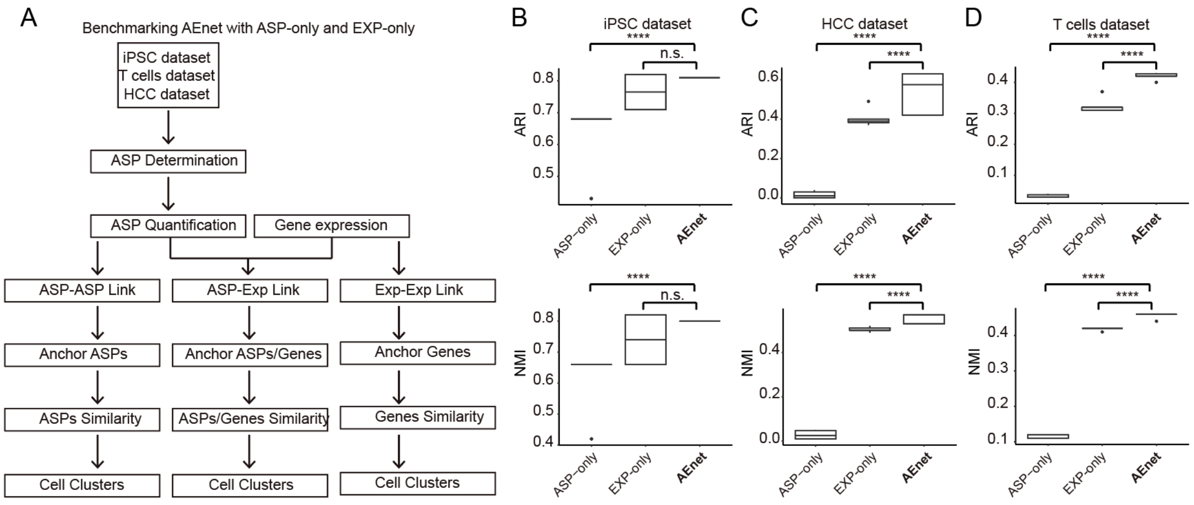

Figure R15. (A) Schematic of the assessment pipeline for ASP-only, RNA-only, and joint clustering analyses. **(B-D)** Quantitative benchmarking of clustering concordance using (upper) Adjusted Rand Index (ARI) and (down) Normalized Mutual Information (NMI) metrics across the three networks. Statistical significance was assessed via one-sided Wilcoxon rank-sum tests. * $P < 0.05$, ** $P < 0.01$, *** $P < 0.001$, **** $P < 0.0001$; n.s., not significant.

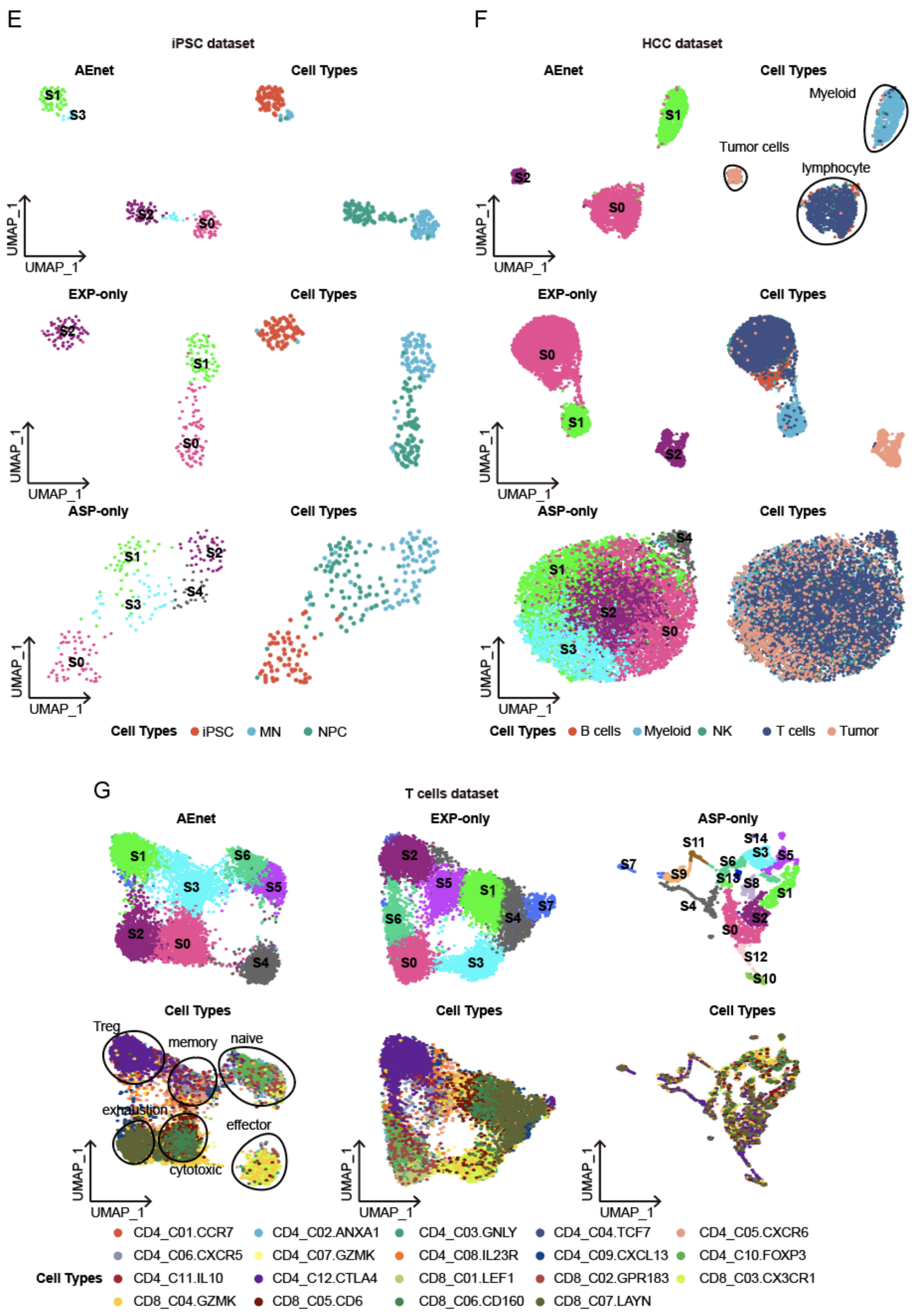

Figure R15. (E–F) Uniform Manifold Approximation and Projection (UMAP) visualizations showing the published cell type annotations (right) and the predicted clusters (left) derived from the ASP-EXP (AEnet) network (top), expression-only (EXP) network (middle), and ASP-only network (bottom) for (E) full-length iPSC and (F) hepatocellular carcinoma (HCC) single-cell RNA-seq datasets. **(G)** UMAP visualizations showing the published cell type annotations (botton) and the predicted

clusters (upper) derived from the ASP-EXP (AEnet) network (left), expression-only (EXP) network (middle), and ASP-only network (right) for the T cells datasets. Clustering solutions shown represent those with the highest adjusted Rand index (ARI).

* The uniform noise model used in evaluations may not reflect biological noise. Have you tested more realistic noise models?

Response: Thank you for highlighting this important point. We acknowledge that the uniform noise model may not adequately capture the complexity of biological variability. To address this limitation, we further evaluated AEnet's performance using more realistic noise models, specifically Gaussian and Poisson noise. We simulated increasing levels of both Gaussian and Poisson noise and generated ASP–ASP similarity matrices (Jaccard index) under each noise condition to assess AEnet's robustness in ASP clustering **(Fig. R16A-B and D-E)**. Using a supervised hierarchical clustering approach, AEnet consistently achieved a high accuracy score—approximately 0.9—between the ground truth and the predicted clusters, even under noise levels as high as 90% for both noise models **(Fig. R15C and F)**. These results demonstrate AEnet's resilience to biologically relevant noise, further supporting its reliability in identifying splicing patterns in realistic, noisy settings. We have incorporated this suggestion into the manuscript at **Page10, Line 256-271 and Figure S5-6.**

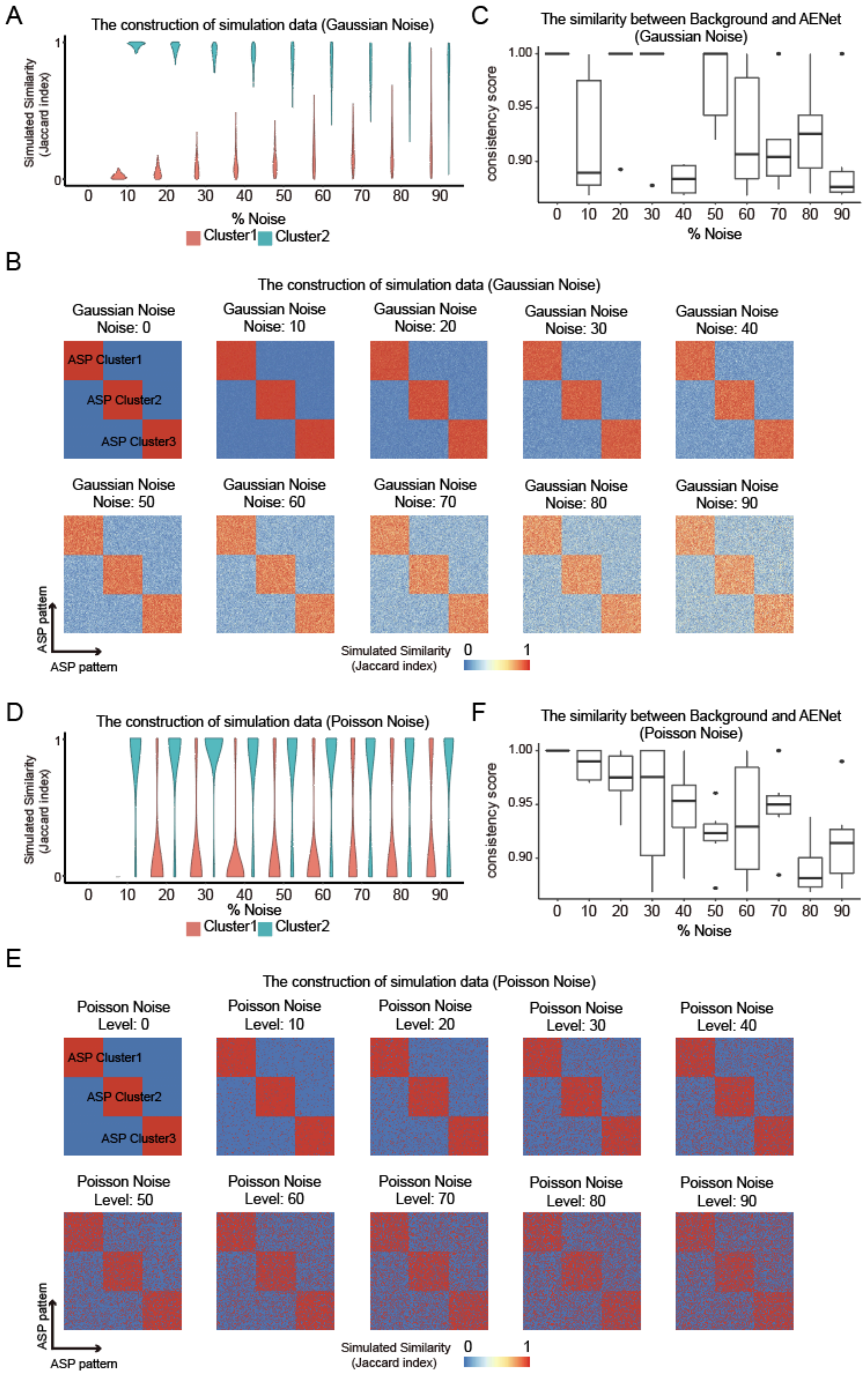

Figure R16. (A-B) Simulated ASPs-ASPs similarity matrix (B) was created with increasing gaussian noise (A). **(C)** Box plots present the jaccard index of ASP clusters between the background and AENet with the increasing gaussian noise.

(D-E) Simulated ASPs-ASPs similarity matrix (E) was created with increasing poisson noise (D). **(F)** Box plots present the jaccard index of ASP clusters between the background and AENet with the poisson noise.

* AEnet claims to bypass the batch effects, but the mechanism should be better explained. Is this achieved because correlations are calculated within each sample and aggregated later?

Response: We appreciate the reviewer's insightful comment regarding batch effects. Our hypothesis posits that if ASP-EXP links are primarily driven by batch effects rather than biological signals, these associations should exhibit sample- or condition-specific patterns. Consequently, we classify ASP-EXP links as batch-vulnerable if they meet either of the following criteria: (i) Supported by only one sample or only one condition, or (ii) Displaying discordant directionality (positive/negative) across different samples and conditions **(Table R6)**. Based on this premise, we established the following operational criteria for identifying batch-vulnerable ASP-EXP links:

1 Associations supported by only a single sample (non-replicable), or

2 Associations showing statistically discordant regulatory effects (positive vs. negative correlations) across different samples/conditions.

We have incorporated this suggestion into the manuscript at **Page4, Line 135-140** and **Figure S1C.**

The filtering critera of AEnet

Multiple Samples in one condition:

ASP	Gene	Supported Samples Numbers(Positive)	Supported Samples Numbers(Negative)	Filtering
Gene10:ASP1	Gene1	>= 2	>= 1	Yes
	Gene2	>= 2	0	No
	Gene3	>= 1	>= 2	Yes
	Gene4	0	>= 2	No
	Gene5	< 2	< 2	No

Multiple conditions:

ASP	Gene	Supported conditions Numbers(Positive)	Supported conditions Numbers(Negative)	Filtering
Gene10:ASP1	Gene1	>= 2	>= 1	Yes
	Gene2	>= 2	0	No
	Gene3	>= 1	>= 2	Yes
	Gene4	0	>= 2	No
	Gene5	< 2	< 2	No

Table R6 Systematic criteria for detecting batch-vulnerable ASP-EXP links across heterogeneous samples and experimental conditions.

* From your results (e.g., Fig. S5C), there seem to be cross-cluster interactions. Are you concerned that rigid clustering may overlook meaningful relationships between clusters?

Response: We appreciate the reviewer's insightful observation regarding cross-cluster interactions observed in our data (Fig. S5C, **Fig. R17A**). While rigid clustering provides discrete groupings, we recognize that this approach may overlook meaningful relationships and transitions between clusters. To address this, we performed a similarity-based regional analysis, organizing ASP clusters based on pairwise similarity and identifying three major regions, each comprising clusters with higher intra-region similarity than inter-region similarity (Fig.R17B).

Region 1 includes clusters C_7 and C_9, which exhibit the highest mutual similarity and are both predominantly enriched in naïve T cells. Interestingly, C_7 also shares

similarity with C_5 and C_10, while C_9 is more closely related to C_4 and C_11. This suggests that C_7 and C_9 may represent bifurcating points leading to two naïve T cell differentiation trajectories—one toward memory T cells (C_5 and C_10), and the other toward effector T cells (C_4 and C_11) **(Fig. R17B-G)**. Supporting this, C_7 is enriched during the naïve-to-memory transition, while C_9 is enriched along the naïve-to-effector axis **(Fig. R17D)**.

Region 2 comprises clusters C_11, C_4, and C_14, which show strong inter-cluster similarity. C_4 acts as a central node, connecting C_11 and C_14, suggesting a potential progression from effector T cells (C_11) to effector memory T cells (C_4), and eventually to exhausted T cells (C_14) **(Fig. R17B-F)**. Notably, C_11 also shares similarity with C_9, and C_14 with C_1—both associated with exhausted states—indicating a continuous exhaustion trajectory **(Fig. R17B-E)**.

Region 3 implies an alternative exhaustion pathway, involving clusters C_5, C_10, and C_12. Here, C_10 links C_5 (memory/naïve-enriched) and C_12 (exhausted-enriched), forming a sequence akin to that in Region 2. Similarity between C_5 and C_7, and between C_12 and C_1, further supports a parallel differentiation path from naïve/memory T cells to exhaustion **(Fig. R17B-G)**.

In summary, while rigid clustering provides discrete groupings, our similarity-based regional analysis reveals underlying transitions and trajectories among ASP-defined clusters. These findings reinforce the reviewer's observation and highlight the importance of complementary methods to uncover dynamic, intermediate cellular states that may be overlooked by strict partitioning. We have incorporated this suggestion into the manuscript at **Page 24, Line 514-538** and **revised Figure S10F-G**.

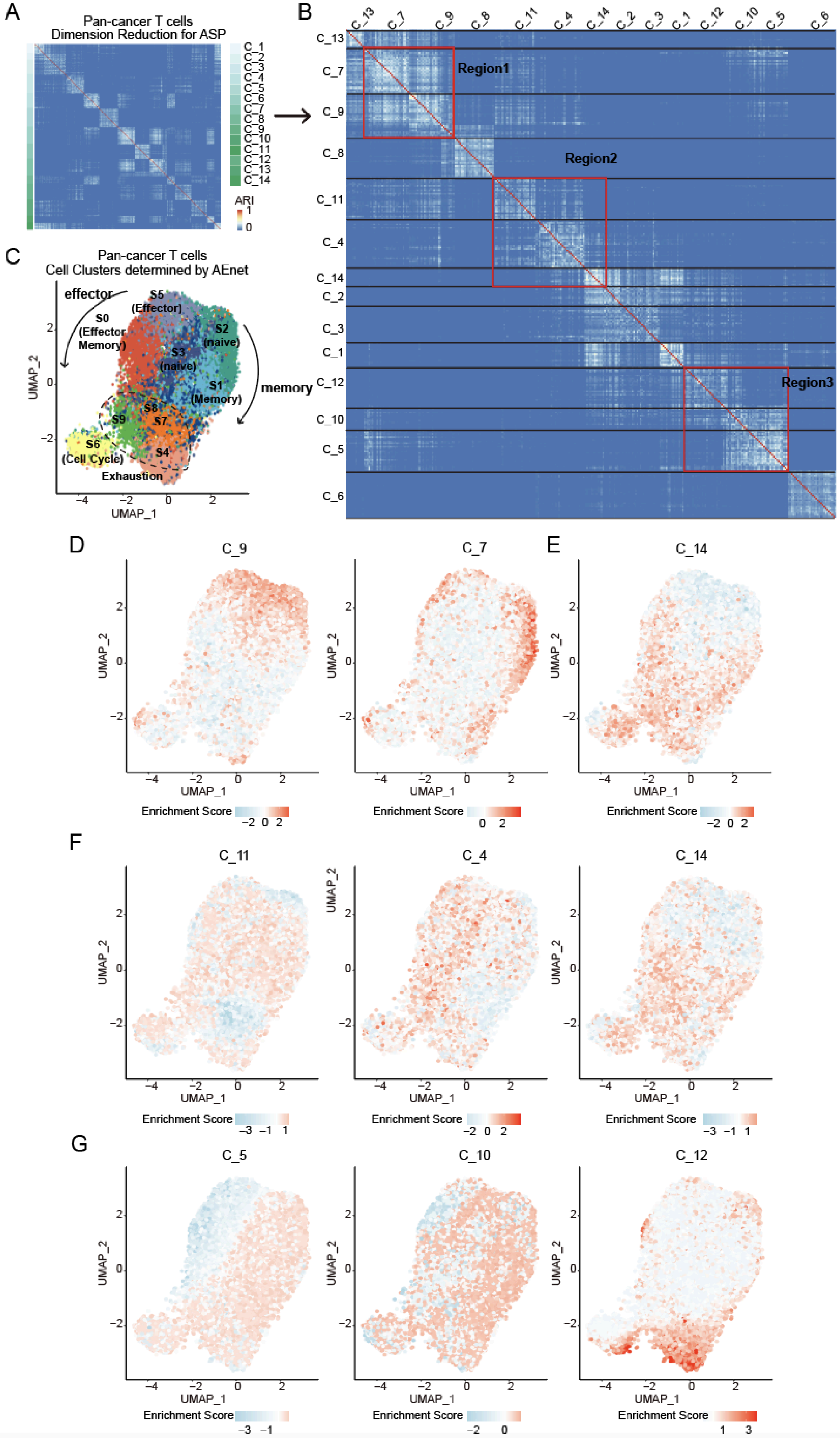

A
Pan-cancer T cells
Dimension Reduction for ASP
C_1
C_2
C_3
C_4
C_5
C_6
C_7
C_8
C_9
C_10
C_11
C_12
C_13
C_14
ARI
1
0
B
Region1
Region2
Region3
C
Pan-cancer T cells
Cell Clusters determined by AEnet
effector
memory
S0 (Effector Memory)
S1 (Memory)
S2 (naive)
S3 (naive)
S4
S5 (Effector)
S6 (Cell Cycle)
S7
S8
S9
Exhaustion
UMAP_1
UMAP_2
D
E
F
G
Enrichment Score

Figure R17. (A) Heatmap showing ASP clusters derived from dimensionality reduction of AEnet on the pan-cancer T cell dataset. **(B)** Heatmap of ASP clusters ordered by similarity, illustrating inter-cluster relationships within the pan-cancer T cell dataset. **(C)** UMAP displays clustering of cell types determined by AEnet. **(D–G)** Distribution of enrichment scores for selected ASP clusters.

* Alternative splicing inherently provides positional information. Have you analyzed whether splicing events are enriched in specific genomic regions?

Response: Yes, we have analyzed whether splicing events are enriched in specific genomic regions and found distinct, dataset-specific enrichment patterns. Splicing (AS) events exhibit positional enrichment, we performed a genome-wide analysis of AS site distribution across three datasets. The genome was partitioned into 10 Mb bins, and AS event frequencies were quantified within each bin. To identify statistically enriched regions, we applied permutation testing (n = 1000), with significance determined by a false discovery rate (FDR) threshold of < 0.05.

This analysis revealed distinct, dataset-specific enrichment patterns **(Fig. R18A-C)**. In the tumor cell dataset, the top three enriched regions were Chr10:70–80 Mb, Chr2:80–90 Mb, and Chr6:30–40 Mb. In the T cell dataset, enrichment was observed in Chr6:30–40 Mb, Chr12:50–60 Mb, and Chr19:30–40 Mb. In the gastrulation dataset, the most enriched bins were Chr1:150–160 Mb, Chr19:40–50 Mb, and Chr11:50–60 Mb. These distinct chromosomal distributions reflect the context-specific nature of AS regulation, likely driven by biological processes such as immune activation, oncogenesis, and early embryonic development.

Importantly, we identified recurrent enrichment in specific genomic regions across datasets, suggesting potential AS hotspots. Notably, Chr6:30–40 Mb was enriched in all three datasets and ranked among the top regions in both the tumor and T cell datasets. This region contains multiple immune-related genes (e.g., HLA-A, HLA-B, HLA-C, PSMB9), supporting the existence of conserved splicing regulation at immune-associated loci, even amid divergent transcriptomic programs **(Fig. R18D-E)**.

We sincerely thank the reviewer for this insightful recommendation. In response, we have performed the suggested comparative analyses. However, to maintain the

conceptual and structural coherence of the manuscript—which is primarily focused on the development and application of AEnet for uncovering splicing-driven regulatory mechanisms—we have decided not to include this analysis in the current version. Nevertheless, we will provide the corresponding analysis scripts alongside our publicly available codebase and plan to incorporate a chromosomal enrichment analysis module in a future update of AEnet.

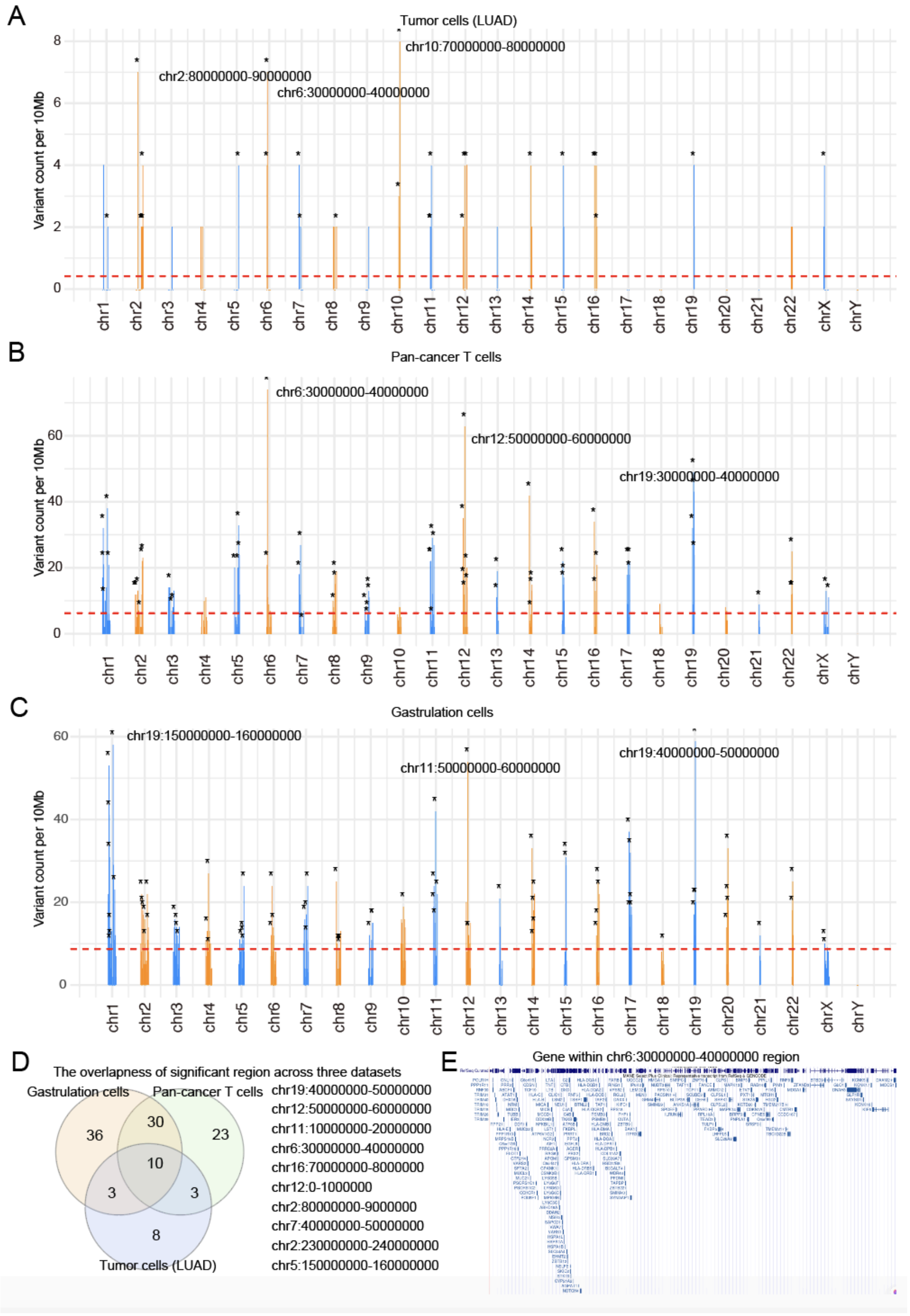

Figure R18. (A-C) Chromosomal distribution of Alternative splicing sites across 24 chromosomes in three datasets: (A) tumor cells, (B) T cells, and (C) gastrulation development. The y-axis represents AS site density (number of sites per 10 Mb

genomic region). The top three enriched regions are annotated for each dataset. Genomic regions with the top three highest AS densities are annotated, with statistically significant regions (hypergeometric test, $P < 0.05$) marked by asterisks.**(D)** Venn diagram showing overlap of statistically significant AS-enriched regions across all three datasets.

Minor points:

* The criteria for defining a cell subpopulation should be stated in the main text.

Response: We appreciate the reviewer's suggestion and have now explicitly defined the criteria for subpopulation identification in the Methods section (Cell Clustering Analysis Part at **Page 35, Line 814-816**). In our study, subpopulations are delineated using a dual-modality approach that integrates alternative splicing patterns (ASPs) with gene expression data. This approach differs from conventional clustering based solely on gene expression.

* Are the same sequencing results used for gene count matrix and ASP detection?

Response: The same sequencing data were used to generate both the gene count matrix and detect alternative splicing (AS) events. Raw reads from single-cell RNA-seq were processed in parallel for gene expression quantification and AS analysis using STAR and RSEM, ensuring consistency in the underlying data for both analyses.

* The first section provides both method description and validation/benchmarking. Consider separating them into distinct sections and expanding each more thoroughly.

Response: We thank the reviewer for highlighting the need for clearer methodological presentation. In response, we have reorganized the content by separating it into two distinct sections: *Methods*, which details the protocols and implementation of AEnet, and *Benchmarking*, which evaluates its performance using public datasets and synthetic data.

* Can AEnet handle larger single-cell datasets? How does it scale computationally?

Response: AEnet is theoretically capable of processing larger-scale datasets due to its inherent computational architecture. The analytical workflow comprises four key steps: (1) sample-specific AEN network construction, (2) cross-sample integration, (3) anchor ASPs clustering, and (4) cell-type clustering with mechanistic prediction. Computational benchmarking identified AEN network construction as the rate-limiting step, prompting our implementation of a parallelized multi-core processing framework to optimize this stage **(Fig. R19)**.

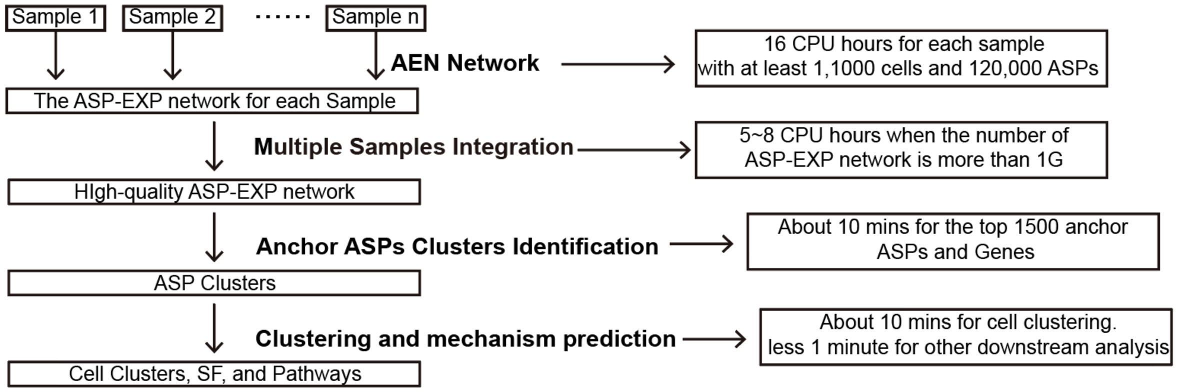

Figure R19. Schematic diagram illustrating the application of AEnet to large-scale single-cell datasets, including computational time estimates for each step.

* Figures in the first section lack self-explanatory captions. Some (e.g., Fig. 1C, S1B) do not provide sufficient information.

Response: We thank the reviewer for this important suggestion. The captions for Fig. 1C and S1B have been rewritten to include: (1) an enhanced description of how dynamic expression of splicing factors influences alternative splicing profiles in Fig. S1B **(Fig. R20A)**; (2) A clarification of the ASP-EXP network constructed through the AEN Network and Multiple Samples Integration steps, as illustrated in Fig. 1B **(Fig. R20B-C)**; (3) an updated explanation of the anchor ASP identification plots shown in Fig. 1B **(Fig. R20B-C)**; and (4) revised annotation of the anchor ASP identification plots in Fig. S1C. We have incorporated this suggestion into the manuscript at **Page 4-5, Line 118-172** and revised **Figure 1B-C** and **S1D**.

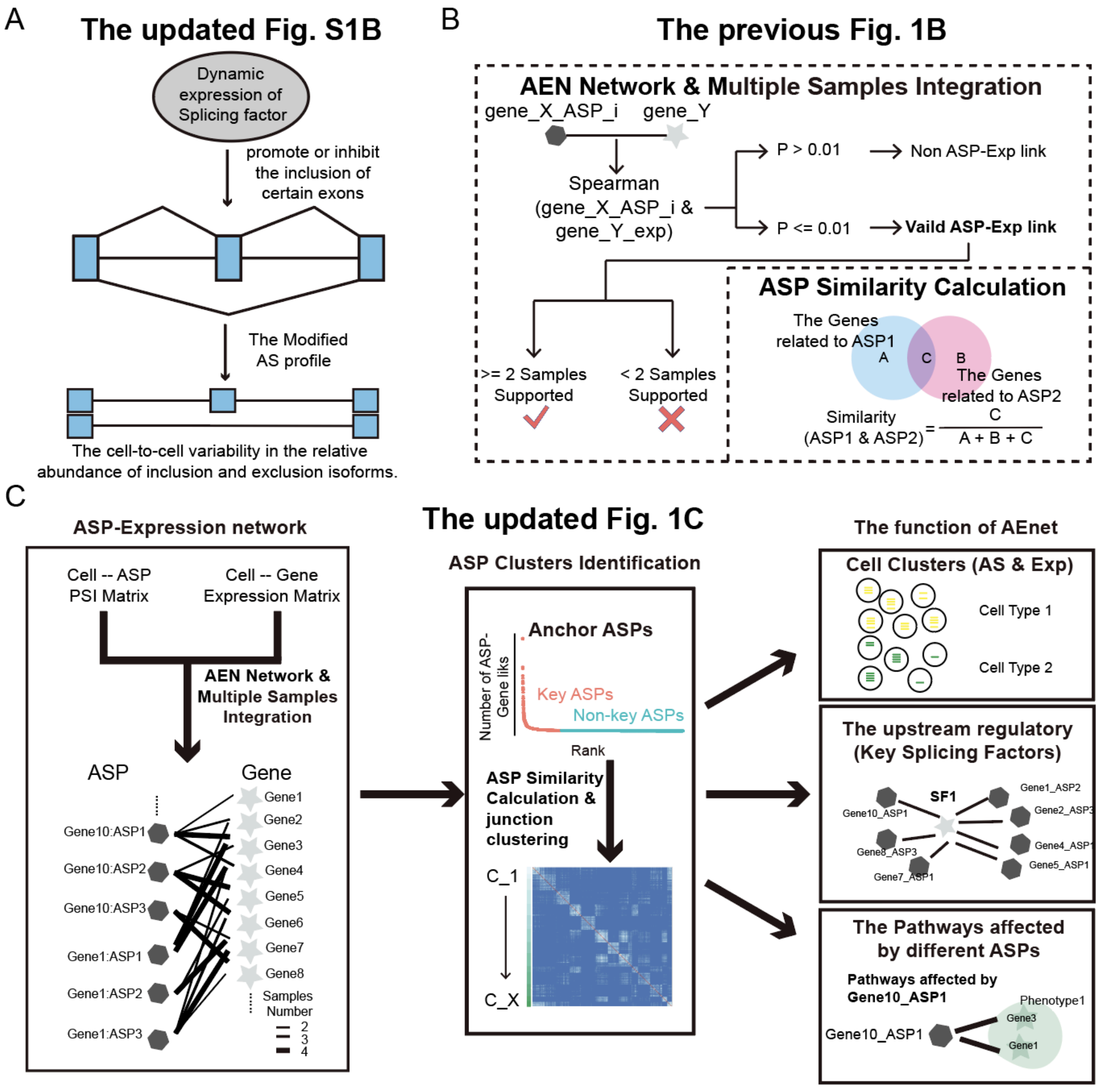

Figure R20. (A) Schematic diagram illustrating the effect of splicing factors on the selection of splicing patterns. **(B)** Schematic Diagram of AEN Network Construction, Multiple Samples Integration, and ASP Similarity Calculation. **(C)** Scheme of AEnet methods.

* The anchor ASP identification plots need x-axes, and the y-axes should be labeled "Number of ASP-Gene Links" instead of just "Degree."

Response: Thank you for this helpful suggestion. In response, we have made the following revisions: (1) added x-axis labels (Rank) to all anchor ASP plots; (2) updated the y-axis label from "Degree" to "Number of ASP–Gene Links" to more accurately represent the data; and (3) ensured consistent formatting. These

modifications enhance the clarity and interpretability of the figures. The corresponding figure legends have also been revised to reflect these updates.

* The cited work from Regev's lab in the introduction is a bit old. Are there more recent references that should be included?

Response: We sincerely appreciate the reviewer's constructive suggestion to update the literature citations. In response, we have incorporated three recent and impactful studies (published between 2019 and 2025) that significantly advance the understanding of cell-type-specific alternative splicing (AS) patterns using single-cell technologies:

1. Clinical relevance: Huang et al. (2023) [22] revealed pervasive alternative splicing dysregulation in pituitary neuroendocrine tumors (PitNETs), uncovering subtype-specific splicing heterogeneity. Their findings improved tumor classification by distinguishing silent corticotroph tumors and identifying a high-risk TPIT lineage subtype driven by ESRP1-mediated splicing abnormalities, thereby enhancing the molecular stratification of PitNETs.

2. Developmental and evolutionary stability of AS: Anoushka et al. (2024) [23]constructed a single-cell full-length isoform atlas across brain regions, cell types, developmental stages, and species. They demonstrated that 72% of genes exhibit isoform diversity driven by alternative splicing, transcription start sites, and polyadenylation. These cell-type-specific isoforms influence protein structure, neurodevelopmental transitions, and disease susceptibility, with conserved splicing patterns observed across mouse and human, alongside human-specific isoforms.

3. Brain-specific splicing programs: David et al. (2019) [24] used single-cell transcriptomics to show that neurexin isoform profiles—essential for synaptic specificity—are established early in neuronal development and remain stable throughout maturation. Their work provides a detailed, cell-type-specific map of cortical neurexin splicing from embryogenesis to adulthood.

These additions support our argument by demonstrating that IRF7's differential AS events, as originally identified by Regev's group, reflect a broader and conserved regulatory mechanism across diverse biological systems. We have revised the introduction accordingly to contextualize these references **(Page 2-3, lines 60–69)**.

Reference

1. Huang Y, Sanguinetti G. BRIE: transcriptome-wide splicing quantification in single cells. *Genome Biol*. 18:1232017;

2. Song Y, Botvinnik OB, Lovci MT, Kakaradov B, Liu P, Xu JL, et al.. Single-cell alternative splicing analysis with Expedition reveals splicing dynamics during neuron differentiation. *Mol Cell*. 67:148–61.e52017;

3. Wen WX, Mead AJ, Thongjuea S. MARVEL: an integrated alternative splicing analysis platform for single-cell RNA sequencing data. *Nucleic Acids Res*. Oxford University Press (OUP); 51:e292023;

4. Linker SM, Urban L, Clark SJ, Chhatriwala M, Amatya S, McCarthy DJ, et al.. Combined single-cell profiling of expression and DNA methylation reveals splicing regulation and heterogeneity. *Genome Biol*. Springer Science and Business Media LLC; 20:302019;

5. Liu S, Zhou B, Wu L, Sun Y, Chen J, Liu S. Single-cell differential splicing analysis reveals high heterogeneity of liver tumor-infiltrating T cells. *Sci Rep*. Springer Science and Business Media LLC; 11:53252021;

6. Zhang L, Yu X, Zheng L, Zhang Y, Li Y, Fang Q, et al.. Lineage tracking reveals dynamic relationships of T cells in colorectal cancer. *Nature*. Springer Science and Business Media LLC; 564:268–722018;

7. Sun Y, Wu L, Zhong Y, Zhou K, Hou Y, Wang Z, et al.. Single-cell landscape of the ecosystem in early-relapse hepatocellular carcinoma. *Cell*. Elsevier BV; 184:404–21.e162021;

8. Xiang X, He Y, Zhang Z, Yang X. Interrogations of single-cell RNA splicing landscapes with SCASL define new cell identities with physiological relevance. *Nat Commun*. Springer Science and Business Media LLC; 2024; doi: 10.1038/s41467-024-46480-9.

9. Grabski IN, Street K, Irizarry RA. Significance analysis for clustering with single-cell RNA-sequencing data. *Nat Methods*. 20:1196–2022023;

10. Stuart T, Butler A, Hoffman P, Hafemeister C, Papalexi E, Mauck WM 3rd, et al.. Comprehensive integration of single-cell data. *Cell*. Elsevier BV; 177:1888–902.e212019;

11. Korsunsky I, Millard N, Fan J, Slowikowski K, Zhang F, Wei K, et al.. Fast, sensitive and accurate integration of single-cell data with Harmony. *Nat Methods*. Springer Science and Business Media LLC; 16:1289–962019;

12. Haghverdi L, Lun ATL, Morgan MD, Marioni JC. Batch effects in single-cell RNA-sequencing data are corrected by matching mutual nearest neighbors. *Nat Biotechnol*. 36:421–72018;

13. Barkas N, Petukhov V, Nikolaeva D, Lozinsky Y, Demharter S, Khodosevich K, et al.. Joint analysis of heterogeneous single-cell RNA-seq dataset collections. *Nat*

Methods. Springer Science and Business Media LLC; 16:695–82019;

14. Xue R, Zhang Q, Cao Q, Kong R, Xiang X, Liu H, et al.. Liver tumour immune microenvironment subtypes and neutrophil heterogeneity. *Nature*. Springer Science and Business Media LLC; 612:141–72022;

15. Maynard A, McCoach CE, Rotow JK, Harris L, Haderk F, Kerr DL, et al.. Therapy-induced evolution of human lung cancer revealed by single-cell RNA sequencing. *Cell*. Elsevier BV; 182:1232–51.e222020;

16. Benegas G, Fischer J, Song YS. Robust and annotation-free analysis of alternative splicing across diverse cell types in mice. *Elife*. eLife Sciences Publications, Ltd; 2022; doi: 10.7554/eLife.73520.

17. Cortés-López M, Chamely P, Hawkins AG, Stanley RF, Swett AD, Ganesan S, et al.. Single-cell multi-omics defines the cell-type-specific impact of splicing aberrations in human hematopoietic clonal outgrowths. *Cell Stem Cell*. Elsevier BV; 30:1262–81.e82023;

18. Fang J, Bolanos LC, Choi K, Liu X, Christie S, Akunuru S, et al.. Ubiquitination of hnRNPA1 by TRAF6 links chronic innate immune signaling with myelodysplasia. *Nat Immunol*. 18:236–452017;

19. Risso D, Perraudeau F, Gribkova S, Dudoit S, Vert J-P. A general and flexible method for signal extraction from single-cell RNA-seq data. *Nat Commun*. 9:2842018;

20. Tran HTN, Ang KS, Chevrier M, Zhang X, Lee NYS, Goh M, et al.. A benchmark of batch-effect correction methods for single-cell RNA sequencing data. *Genome Biol*. Springer Science and Business Media LLC; 21:122020;

21. Wan Y, Larson DR. Splicing heterogeneity: separating signal from noise. *Genome Biol*. 19:862018;

22. Huang Y, Guo J, Han X, Zhao Y, Li X, Xing P, et al.. Splicing diversity enhances the molecular classification of pituitary neuroendocrine tumors. *Nat Commun*. Springer Science and Business Media LLC; 16:15522025;

23. Joglekar A, Hu W, Zhang B, Narykov O, Diekhans M, Marrocco J, et al.. Single-cell long-read sequencing-based mapping reveals specialized splicing patterns in developing and adult mouse and human brain. *Nat Neurosci*. Springer Science and Business Media LLC; 27:1051–632024;

24. Lukacsovich D, Winterer J, Que L, Luo W, Lukacsovich T, Földy C. Single-cell RNA-seq reveals developmental origins and ontogenetic stability of neurexin alternative splicing profiles. *Cell Rep*. Elsevier BV; 27:3752–9.e42019;
