# Supplementary material for: AEnet: a practical tool to construct the splicing-associated phenotype atlas at a single cell level
Source: Gigascience. 2025 Sep 24;14:giaf110. doi: 10.1093/gigascience/giaf110 (PMC12457822; doi:10.1093/gigascience/giaf110)
Supplement: giaf110_GIGA-D-25-00064_Revision_2 [file giaf110_giga-d-25-00064_revision_2.pdf]

GigaScience

AEnet: a practical tool to construct the splicing associated phenotype atlas at single cell level

--Manuscript Draft--

**Manuscript Number:**	GIGA-D-25-00064R2
**Full Title:**	AEnet: a practical tool to construct the splicing associated phenotype atlas at single cell level
**Article Type:**	Technical Note
**Funding Information:**	Shenzhen Science and Technology Program (LCYX20220620105200001) — Miss Liang Wu<br>Shenzhen Science and Technology Program (JCYJ20240813150001003) — Dr Xi Chen<br>Science and Technology Innovation Key R&D Program of Chongqing (CSTB2024TIAD-STX0003) — Miss Liang Wu<br>National Natural Science Foundation of China (32300514) — Miss Liang Wu
**Abstract:**	Alternative splicing (AS), a crucial driver of proteomic diversity, is a fundamental source of cellular heterogeneity alongside gene expression levels. AS is closely linked to various physiological and pathological processes, including tumor progression and embryonic development. Single-cell RNA sequencing (scRNA-seq) technologies capture AS events through junction reads at cellular resolution, enabling the identification of core AS events that regulate specific cell types or states. However, single-cell sequencing technology and its data are plagued by inherent limitations such as shallow sequencing depth, high dropout rates, and batch effects. Furthermore, previous clustering approaches have overlooked the crucial interplay between alternative splicing (AS) and gene expression in defining distinct 'cell types,' posing ongoing challenges in this field. In this study, we present a novel method called the AEnet, which combines gene expression levels with AS patterns to profile cellular heterogeneity and define what we term 'cell subpopulations'. AEnet also identifies key AS events and infers the regulatory mechanisms underlying these events. By applying AEnet to tumor cells, pan-cancer immune cells, and embryonic cells, we demonstrate enhanced cell clustering, the identification of novel AS events with potential functional importance, and the discovery of the key splicing factors involved in cell state transformation. The application of AEnet provides new insights into cellular heterogeneity and its role in both physiological and pathological processes.
**Corresponding Author:**	Liang Wu<br><br>CHINA
**Corresponding Author Secondary Information:**	
**Corresponding Author's Institution:**	
**Corresponding Author's Secondary Institution:**	
**First Author:**	Liang Wu
**First Author Secondary Information:**	
**Order of Authors:**	Liang Wu<br>Shang Liu<br>Xi Chen<br>Yinqi Bai

Powered by Editorial Manager® and ProduXion Manager® from Aries Systems Corporation

	Shiping Liu
	Xiaohu Huang
	Yuhang Wang
	Waidong Huang
	Pengfei Qin
	Rui Li
	Xuanxuan Zou
	Wending Pang
**Order of Authors Secondary Information:**	
**Response to Reviewers:**	Point-by-point response to the referees' comments:<br>Reviewer #1: The authors have addressed my concerns.<br><br>Reviewer #2: The authors have thoroughly addressed my comments. I am pleased to see that the benchmarking analysis is substantially improved and the utility of the software has been demonstrated in wider context, i.e., additional datasets.<br><br>My only remaining comment relates to Comment no. 8: While the authors provided the source code for the R package, and the dataset, I would encourage the authors to provide the R Markdown tutorial. This documentation should demonstrate the pre-processing steps and analysis of at least one example dataset and correspondingly reproduce selected figures in the manuscript. This is not only for the purpose of transparency and reproducibility, but this will in turn encourage prospective users, like myself, to apply this software.<br>Response: We sincerely thank the reviewer for the valuable suggestion. To facilitate reproducibility and help users get started, we also provide demonstration datasets corresponding to two biological systems using the R Markdown tutorial:<br>iPSC dataset: https://liushang17.github.io/ipsc.html<br>T cell dataset: https://liushang17.github.io/tcells.html<br>The corresponding R Markdown source code is also available for both datasets (https://github.com/liushang17/AEnet). These tutorials reproduce key steps and selected figures from the manuscript, offering prospective users a practical guide for applying the AEnet package to their own data.<br><br>Reviewer #3: I appreciate the authors' careful attention to the reviewers' comments. The updated manuscript has addressed many concerns raised in the reviews, especially<br>*Clear separation of model description and benchmarking improves clarity<br>*Benchmarking across multiple additional datasets and platforms enhance the generalizability<br>*Batch effect handling is better explained<br>*Ablation study (A-net vs. E-net) supports the value of joint modeling<br>*Noise modeling and ASP thresholding are better tested<br><br>Overall, this work has been strengthened. A few points should be fixed<br>*Some of the workflow figures (e.g., Fig. 2, S2-S4) are visually confusing and could benefit from clearer visual layout or annotations.<br>Response: We sincerely thank the reviewer for this constructive suggestion. We have revised the schematics and updated the figure descriptions and legends in Figs. 2 and S2–S4, by adjusting the layout for better readability, adding step-wise labels, and ensuring consistent visual elements across all panels.<br><br>Figure S2. Schematic overview of the AEnet pipeline. Inputs (Normal): Cell–Junction Count Matrix, Cell–Gene Expression Matrix, and a predefined list of splicing factors. Key steps are highlighted in bold. Outputs (in bold Italic): Cell clusters, predicted key splicing factors, and signaling pathways regulated by specific alternative splicing patterns (ASPs).<br><br><br>Figure S3. Schematic overview of the pipeline for the comprehensive evaluation of

Powered by Editorial Manager® and ProduXion Manager® from Aries Systems Corporation

	AEnet, organized into four steps: (1) ASP determination and quantification (compared with MARVEL), (2) AEN network construction and multi-sample integration (evaluation of different count thresholds), (3) ASP/gene cluster identification (assessment under varying noise levels), and (4) cell clustering (ablation analysis and comparison with SCASL and scQuint).<br><br>Figure S4. Schematic overview of benchmarking strategy for evaluating different count thresholds on the construction of ASP-EXP Link and Anchor ASPs.<br><br>Figure 2. Schematic diagram of the assessment pipeline for ASP-only, RNA-only, and joint clustering analyses, with all other processing steps remaining identical to those in AEnet.<br><br>*In a few places, the revised manuscript still contains language that seems directly copied from the authors' point-by-point response. The writing would benefit from another editing pass to smooth out tone and narrative flow before final publication.<br><br>Response: Thank you for this astute observation. We fully agree that any residual textual overlap between the manuscript and our point-by-point response is inappropriate for final publication. To address this comprehensively, we have revised the relevant sections of the manuscript. Specifically, the text on Page 10, Lines 248–251 has been updated as follows:<br>"While filtering low-count ASPs improves analytical reliability, we also considered the potential risk of excluding rare but biologically relevant splicing events. To mitigate this, AEnet defines an ASP as 'valid' in a sample only if it is supported by ≥5 reads across ≥20 cells. ASPs failing this threshold are excluded from downstream analysis due to their sparsity, which compromises the reliability of similarity estimates between splicing and expression. To evaluate whether this filtering inadvertently excludes informative, low-abundance ASPs, we stratified all ASPs into five categories based on the number of supporting cells: Invalid (≤20 cells; excluded), Type 1 (>20–30 cells), Type 2 (>30–40 cells), Type 3 (>40–50 cells), and Type 4 (>50 cells). An ASP was assigned to the highest applicable category if it met the criteria in ≥3 samples (Fig. S4F)."<br>Additionally, Page 24, Lines 531–536 has been revised to read:<br>"In summary, while rigid clustering provides discrete groupings, our similarity-based regional analysis reveals underlying transitions and trajectories among ASP-defined clusters. These findings underscore the dynamic continuum of T cell state transitions and highlight the utility of complementary approaches in capturing intermediate cell states that may be overlooked by strict partitioning."<br>Beyond these specific changes, we have thoroughly re-evaluated the entire manuscript and revised the relevant sections (Page 3, Lines 75–79; Page 9, Lines 222–227; Page 10, Lines 248–251 and 253–256; and figure legends for Figs. S1–S4) to ensure consistency, accuracy, and seamless integration with the overall narrative. The manuscript has also undergone a comprehensive editorial revision to enhance clarity, tone, and flow throughout.
**Additional Information:**	
**Question**	**Response**
Are you submitting this manuscript to a special series or article collection?	No
**Experimental design and statistics**<br><br>Full details of the experimental design and statistical methods used should be given in the Methods section, as detailed in our Minimum Standards Reporting Checklist. Information essential to interpreting the data presented should be made available in the figure legends.	Yes

Powered by Editorial Manager® and ProduXion Manager® from Aries Systems Corporation

Have you included all the information requested in your manuscript?	
**Resources**<br><br>A description of all resources used, including antibodies, cell lines, animals and software tools, with enough information to allow them to be uniquely identified, should be included in the Methods section. Authors are strongly encouraged to cite Research Resource Identifiers (RRIDs) for antibodies, model organisms and tools, where possible.<br><br>Have you included the information requested as detailed in our Minimum Standards Reporting Checklist?	Yes
**Availability of data and materials**<br><br>All datasets and code on which the conclusions of the paper rely must be either included in your submission or deposited in publicly available repositories (where available and ethically appropriate), referencing such data using a unique identifier in the references and in the “Availability of Data and Materials” section of your manuscript.<br><br>Have you have met the above requirement as detailed in our Minimum Standards Reporting Checklist?	Yes
GigaScience has policies and guidelines in place for the use of generative AI-writing tools such as ChatGPT. If you have used such writing tools to assist with writing the manuscript this must be declared and cited in the text. Authors should not list AI-writing tools and other AI-assisted technologies as an author or co-author and should acknowledge that they are fully responsible for text generated or refined by AI-writing tools.<p>	No

A summary of use (particularly in the introduction or among methods) needs to be included at the end of the paper, and the outputs should also be included as a supplementary file hosted in GigaDB or other open repositories. Please read our guidelines for more information. <p><br><br>By submitting to GigaScience, you are aware of the journal's AI-writing tools policy, and if you have declared use of such tools below, you have acknowledged this where appropriate in your manuscript and have made a summary of use and outputs available. <p><br><b>AI-assisted writing tools have been used in the preparation of this manuscript?	

Manuscript

Click here to access/download;Manuscript;AEN Manuscript_final.docx

AEnet: a practical tool to construct the splicing associated phenotype atlas at single cell level

Shang Liu[1,13*], Xi Chen[1,2,3,*], Xiaohu Huang[1,4], Yuhang Wang[1,4], Waidong Huang[1,5], Pengfei Qin[1,2,3], Rui Li[12], Xuanxuan Zou[1], Wending Pang[1,4], Xiaoyun Huang[6], Shiping Liu[7,8#], Yinqi Bai[9#], Liang Wu[1,2,3,10,11#]

1. BGI Research, Chongqing 401329, China
2. State Key Laboratory of Genome and Multi-omics Technologies, BGI Research, Shenzhen 518083, China
3. BGI Research, Shenzhen 518083, China
4. School of Biology and Biological Engineering, South China University of Technology, Guangzhou, China
5. College of Life Sciences, University of Chinese Academy of Sciences, Beijing, China
6. JC School of Public Health and Primary Care, Faculty of Medicine, The Chinese University of Hong Kong, Hong Kong SAR, China
7. State Key Laboratory of Genome and Multi-omics Technologies, BGI Research, Hangzhou 310030, China
8. BGI Research, Hangzhou 310030, China
9. BGI Research, Sanya 572025, China
10. Zhongshan-BGI Precision Medical Center, Zhongshan Hospital, Fudan University, Shanghai, China
11. Shanxi Medical University-BGI Collaborative Center for Future Medicine, Shanxi Medical University, Taiyuan 030001, China
12. Institute of Intelligent Medical Research (IIMR), BGI Genomics, Shenzhen 518083, China
13. Ruijin Yangtze River Delta Health Institute, Wuxi Branch of Ruijin Hospital, Ruijin Hospital, Shanghai Jiao Tong University School of Medicine, Shanghai, China

* These authors contributed equally

# Correspondence: Liang Wu (wuliang@genomics.cn); Yingqi Bai

(baiyinqi@genomics.cn); Shiping Liu (liushiping@genomics.cn)

Shang Liu[0000-0001-8050-7968];

Xi Chen [0009-0003-2861-7395];

Yuhang Wang [0000-0001-6804-0445];

Waidong Huang [0009-0001-6125-0148];

Rui Li [0000-0001-5613-4890];

Wending Pang [0009-0002-0095-4843];

Xiaoyun Huang [0000-0002-3389-9759];

Shiping Liu [0000-0003-0019-619X];

Yinqi Bai [0000-0003-1017-5712];

Liang Wu [0000-0001-6259-261X];

Abstract

Alternative splicing (AS), a crucial driver of proteomic diversity, is a fundamental source of cellular heterogeneity alongside gene expression levels. AS is closely linked to various physiological and pathological processes, including tumor progression and embryonic development. Single-cell RNA sequencing (scRNA-seq) technologies capture AS events through junction reads at cellular resolution, enabling the identification of core AS events that regulate specific cell types or states. However, single-cell sequencing technologies and its data are plagued by inherent limitations such as shallow sequencing depth, high dropout rates, and batch effects. Furthermore, previous clustering approaches have overlooked the crucial interplay between alternative splicing (AS) and gene expression in defining distinct 'cell types,' posing ongoing challenges in this field. In this study, we present a novel method called AEnet, which combines gene expression levels with AS patterns to profile cellular heterogeneity and define what we term 'cell subpopulations'. AEnet also identifies key AS events and infers the regulatory mechanisms underlying these events. By applying AEnet to tumor cells, pan-cancer immune cells, and embryonic cells, we demonstrate enhanced cell clustering, the identification of novel AS events with potential functional

importance, and the discovery of the key splicing factors involved in cell state transitions. The application of AEnet provides new insights into cellular heterogeneity and its role in both physiological and pathological processes.

Introduction

The diversity of proteomes is an important manifestation of the complexity of organisms, and alternative splicing (AS) is one of the major factors contributing to this diversity [1,2]. The major types of alternative splicing include exon skipping (SE), mutually exclusive exons (MXE), intron retention (IR), alternative 3′ splice site (A3SS), alternative 5′ splice site (A5SS), alternative last exon (ALE), alternative first exon (AFE), and multiple-exon splicing (MSE) have also been identified[3,4]. AS plays a crucial role in various physiological and pathological processes such as embryonic development [5], aging [6], and tumor progression [7,8]. Several key splicing factors are involved in the regulation of AS during disease progression. For instance, RBFOX2 is a master regulator for mesenchymal tissue-specific splicing [9], playing a significant role in the formation of mesenchymal-like states in tumor cells [10]. Recently, single-cell transcriptomics has become a powerful tool for analyzing profiles of AS at high resolution [11,12]. Several recent studies have expanded our understanding of cell-type-specific splicing programs. For example, Huang et al. revealed that subtype-specific splicing patterns refine the classification of pituitary neuroendocrine tumors (PitNETs) [13]. Anoushka et al. constructed a cross-species isoform atlas, demonstrating conserved and human-specific splicing programs linked to neurodevelopment and disease[14]. Similarly, David et al. showed that neurexin isoforms are cell-type-specific and developmentally stable in the brain, underpinning synaptic identity [15]. Together, these studies underscore the biological relevance and regulatory specificity of AS in health and disease, motivating the development of methods like AEnet for systematic analysis of AS at single-cell resolution.[16].

Although several computational methods have been developed to analyze alternative splicing at the single-cell level, most rely on predefined cell types based on canonical gene expression clustering. These methods typically assess splicing heterogeneity through comparisons between expression-defined groups, in a manner analogous to differential gene expression analysis **(Table S1)**. However, cell types defined by AS can differ substantially from those defined by gene expression, which may result in incomplete detection of splicing heterogeneity. This limitation is evident in tools such as BRIE [17], Outrigger [18], and our earlier method, DESJ-detection [19]. Furthermore, existing single-cell analysis tools lack the extensibility to reveal the regulatory mechanisms of alternative splicing, infer its regulatory factors, and identify functional pathways of specific isoforms—rather than merely those of differentially spliced genes **(Table S1)**. MARVEL [20] addresses this limitation by performing gene ontology (GO) enrichment analysis using the clusterProfiler R package, which can identify enriched pathways among differentially spliced genes. However, this approach only indirectly

infers the potential functional relevance of AS events and does not directly assess the role of individual isoforms produced via alternative splicing, thus limiting an in-depth understanding of how alternative splicing influences cells and cell states [21,22].

Several issues require further consideration in the design of a single-cell AS analytical pipeline. First, unlike RNA expression, which is typically quantified in absolute values, AS events are usually quantified as proportional values. The sparsity of scRNA-seq data often introduces the "not a number (NaN)" challenge during calculations when the denominator (the total number of AS events for a given gene in a single cell) is zero [23]. Second, AS events can also be affected by batch effects, an inherent limitation of single-cell techniques [24]. Third, not all AS events contribute functionally to cellular heterogeneity [25]. Therefore, both upstream and downstream approaches that account for filtering processes should be incorporated.

To address these challenges, we present the Alternative Splicing-Gene Expression Network (AEnet) to explore core AS events and gene co-expression patterns in a network at the single-cell level. Using our algorithm, we find that both splice site preferences and gene expression contribute to cellular heterogeneity during clustering, though they exhibit dynamic interplays and varying weights across different datasets. We refer to the separated clusters as cell (sub)populations to avoid confusion with either cell types or cell states. The software has three major functions: first, to construct AS profiling-based clusters and separate cell subpopulations with distinct AS-gene expression networks; second, to identify key splicing factors for AS clusters (analogous to gene markers); and third, to pinpoint functional pathways involved in the regulatory mechanisms based on core subsets of AS events.

By applying the AEnet method to malignant cells with different immunotherapy responses, T cell analysis in pan-cancer, and cell differentiation during embryonic gastrulation, we demonstrate the power of AEnet in fine-grained clustering of cells by disease or developmental states, seamlessly linking upstream regulatory factors and downstream action pathways, highlighting novel isoforms of functional importance, and constructing alternative splicing landscapes along the AS-based developmental trajectory. These findings will deepen our understanding of the role of alternative splicing in tumorigenesis and embryonic development, providing new strategies and ideas for clinical prognosis prediction, tumor immunotherapy, and congenital disease treatment.

Results

The overview of AEnet

We have developed the AEnet method that integrates alternative splicing with gene expression levels to uncover cellular splicing heterogeneity and underlying regulatory mechanisms. In brief, AEnet begins by quantifying alternative splicing patterns (ASPs)

using junction reads from individual cells during data preprocessing (**Fig. 1A**). The percent spliced-in (PSI) for a specific AS event is defined as the proportion of junction reads curated from all detected junctions that span the same site (**Fig. 1A**). Notably, unlike expression values, which are always nonnegative integers, PSI is assigned as NaN (resulting from division by 0) when no junction reads are detected at a presupposed AS site in a cell (**Fig. S1A**). In scRNA-seq data, the prevalent RNA dropout and shallow sequencing issues make the occurrence of NaN even more challenging within individual cells.

To mitigate this effect, for each ASP and each gene, we limit the calculation of the correlation (Spearman by default) to cells with valid PSI values and the gene's expression level, retaining only those ASP-expression correlation links (referred to as ASP-EXP links) with significant P values (**Fig. 1B**). This filtering step ensures that only statistically robust associations are retained, as these statistically significant links indicate potential relationships between gene expression dynamics and the usage preference of specific ASPs across the cells. When multiple scRNA-seq samples are available under any experimental conditions, we retain only ASP-EXP links that share the same correlation trend and appear at a moderately higher frequency (2 by default) to exclude batch effect-induced artifacts (**Fig. S1B-C**). These steps allow AEnet to overcome technical noise and capture common relationships between AS and gene expression.

We hypothesize that the interactions identified in common ASP-EXP links play either direct or indirect roles in post-transcriptional regulation and gene expression diversity. For instance, increased expression of specific splicing factors may promote or inhibit the inclusion of certain exons, thereby modifying the AS profile of target genes (**Fig. S1D**)[26]. Additionally, distinct isoforms are associated with the expression dynamics of multiple genes and influence specific signaling pathways, ultimately contributing to changes in cell state (**Fig. S1E**). In other cases, both alternative splicing and gene expression levels may be dysregulated, leading to aberrant gene function during stress responses or in diseases[27].

To prioritize splicing events with broad regulatory influence, AEnet ranks ASPs based on the number of associated ASP–EXP links. The top 1,500 ASPs (by default) are selected as anchor ASPs. Pairwise similarities between anchor ASPs are then computed using the Jaccard index of their associated gene sets, resulting in an ASP similarity matrix used for clustering **(Fig. S1F–G)**. In parallel, a gene similarity matrix is constructed to define anchor genes, which are similarly clustered into gene modules **(Fig. S1H).** These clusters represent co-regulated gene sets and splicing programs.

Taken together, the AEnet pipeline begins with the detection of alternative splicing patterns (ASPs) at the cellular level, followed by the construction of ASP (of gene i)–expression (of gene j) links (ASP-EXP links), and generation of the ASP similarity matrix at the sample level. Ultimately, it uncovers specific ASPs and co-

expression/regulatory patterns at the cell population level (**Fig. 1C, S2A**). The output of AEnet focuses on evaluating three main biological events (**Fig. 1C, S2B-D**): first, the separation of cell subpopulations with distinct ASP compositions; second, the key splicing factors that influence specific ASP clusters (analogous to gene markers), identified based on a predefined list of splicing factors **(Table S2)**; and third, the functional pathways activated or inhibited by individual or small subsets of ASPs. These downstream analyses demonstrate AEnet's capability in identifying cellular splicing heterogeneity and regulatory mechanisms.

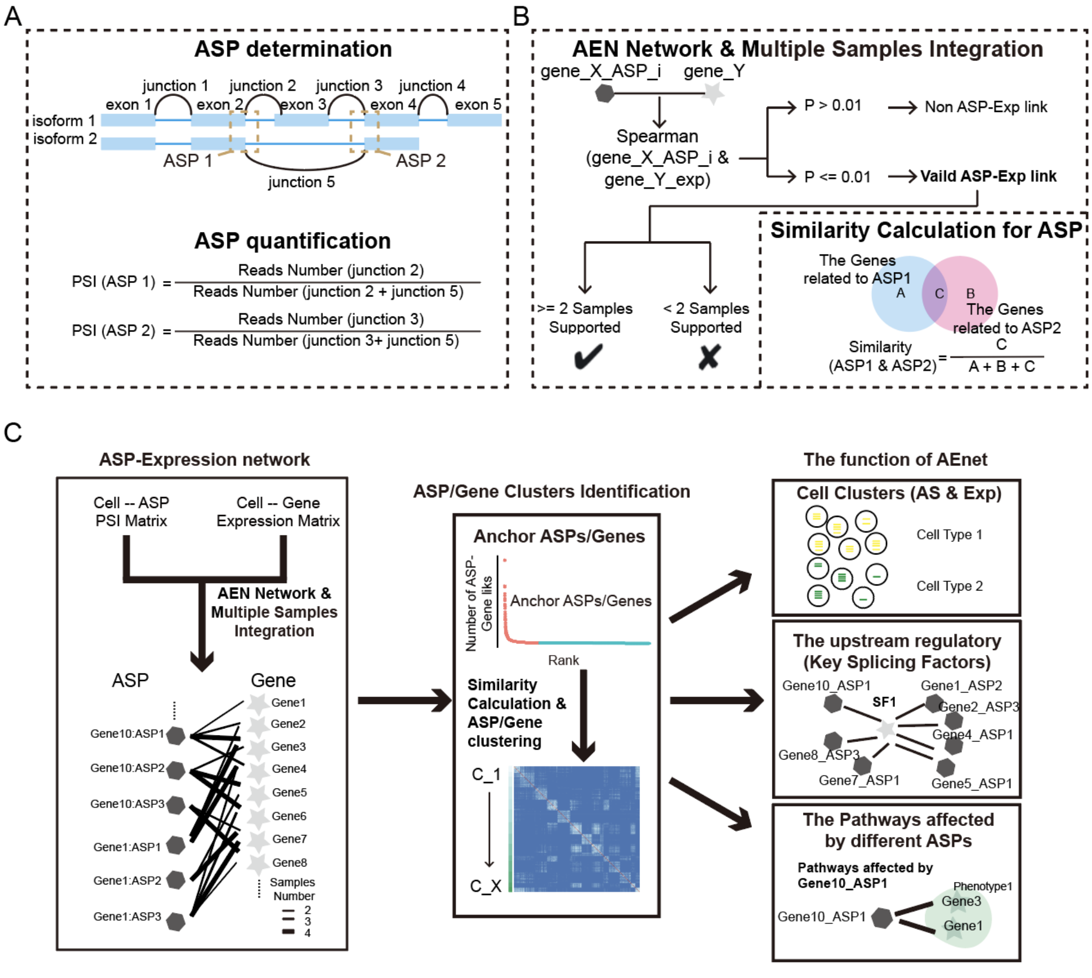

Figure 1. Schematic diagram of the AEnet method. A. Schematic diagram of the identification of alternative splicing pattern (ASP) and the calculation of PSI. **B.** Schematic diagram of AEnet network construction, multiple samples integration, and ASP similarity calculation. **C.** Scheme of AEnet methods.

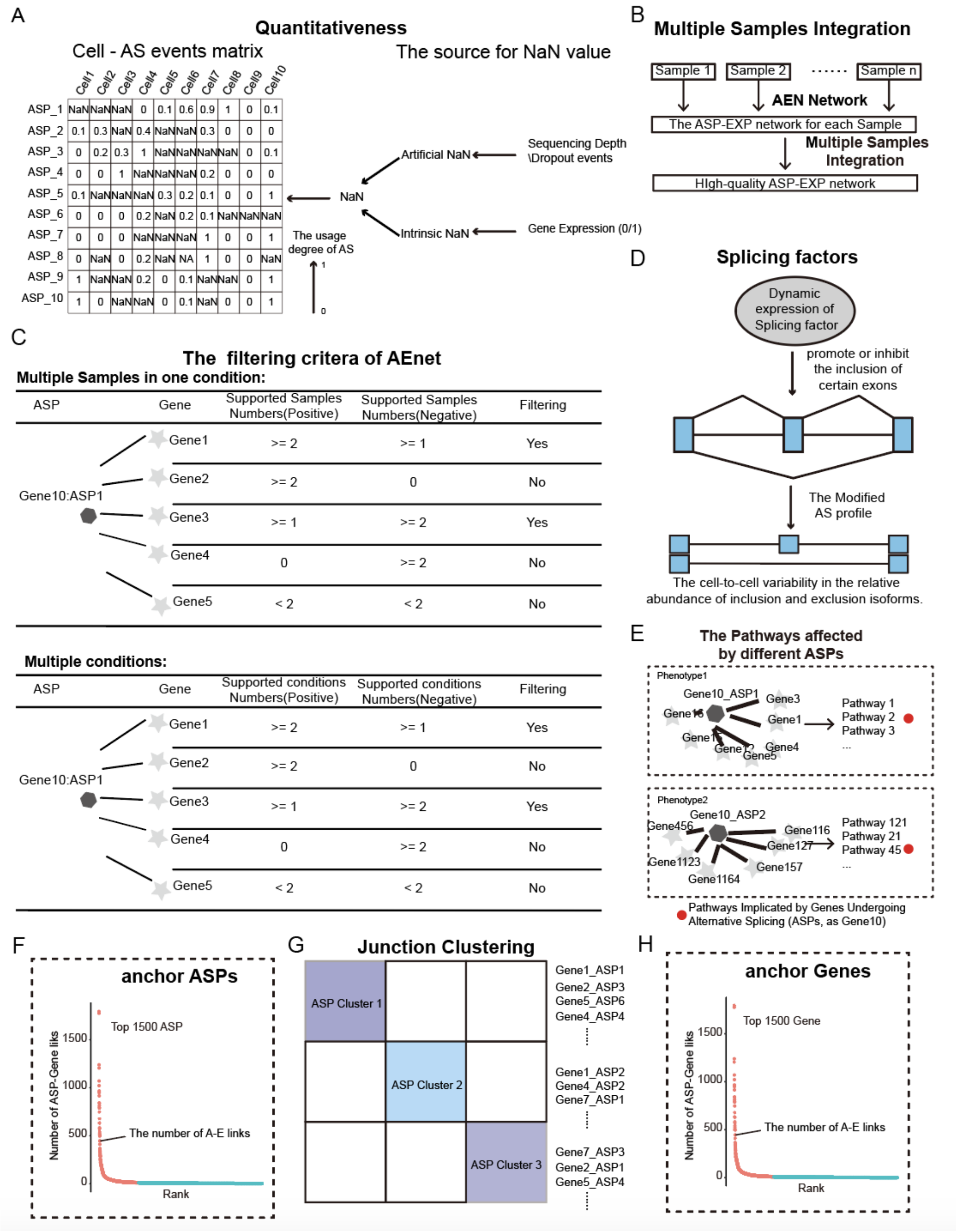

Figure S1. The key difficulties in cell splicing heterogeneity analysis. A. The NaN value problem and its source. **B.** Schematic diagram illustrating the application of AEnet to multiple samples. **C.** Systematic criteria for detecting batch-vulnerable ASP-EXP links across heterogeneous samples and experimental conditions. **D.** Schematic diagram illustrating the effect of splicing factors on the selection of splicing patterns. **E.** Schematic diagram showing how different ASPs of the same gene impact distinct biological pathways. **F.** The selection of anchor ASPs according to the number of ASP-

Gene links of the ASPs. **G.** The identification of ASP clusters. **H.** The selection of anchor Genes according to the number of ASP-Gene links of the genes.

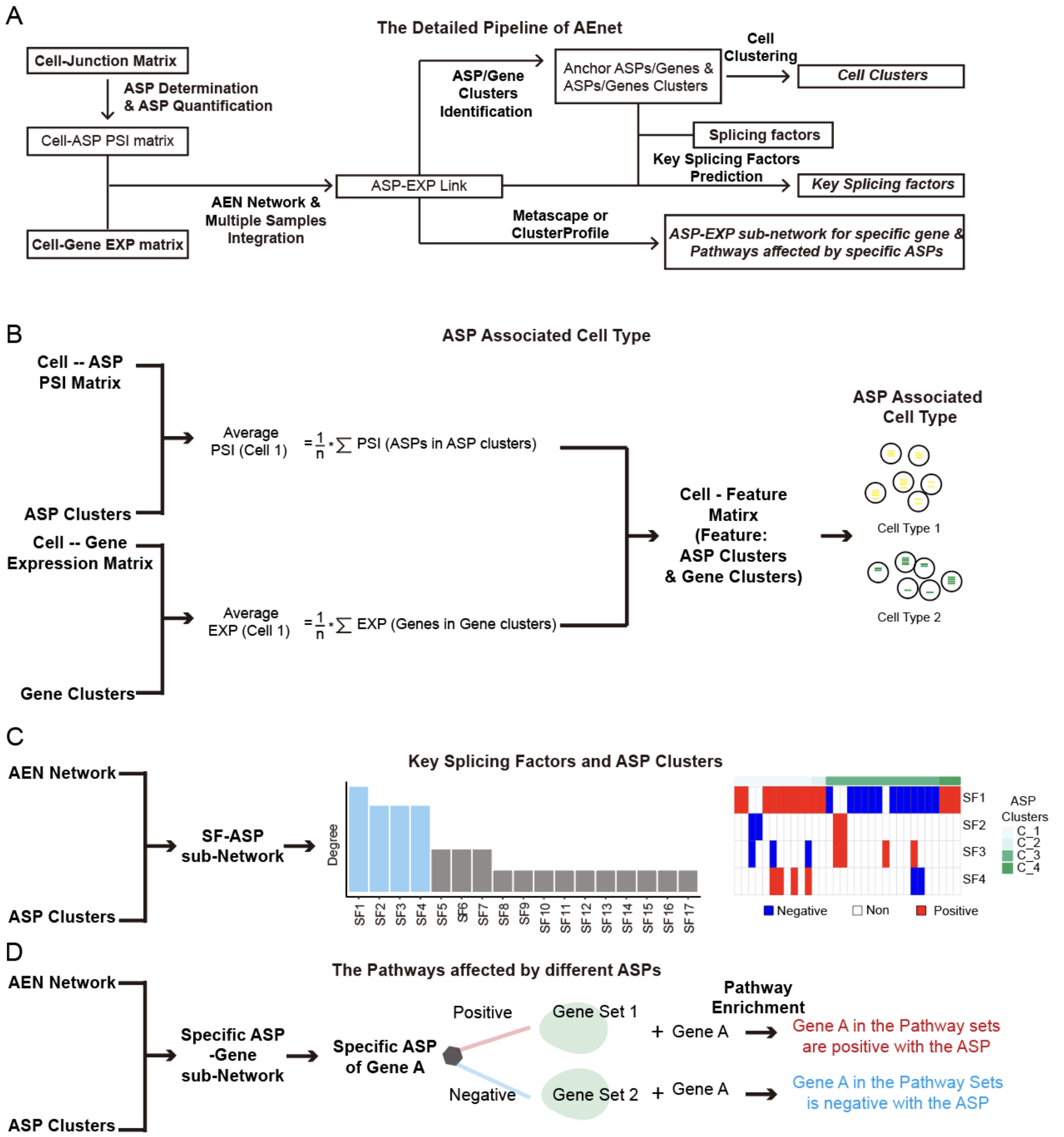

Figure S2. The three major functions of AEnet. A. Schematic overview of the AEnet pipeline. Inputs (Normal): Cell–Junction Count Matrix, Cell–Gene Expression Matrix, and a predefined list of splicing factors. Key steps are highlighted in bold. Outputs (in bold Italic): Cell clusters, predicted key splicing factors, and signaling pathways regulated by specific alternative splicing patterns (ASPs).**B.** Schematic diagram of the analysis of cellular splicing heterogeneity. **C.** Identification of the most critical splicing factors associated with the ASP Set. **D.** Schematic diagram of pathway inference associated with different patterns of ASP.

Evaluation of AEnet for Comprehensive Characterization of Splicing Heterogeneity

We next evaluated AEnet across multiple analytical steps, including ASP–EXP network construction (comprising ASP identification, ASP quantification, and the prediction of ASP and gene clusters), and cell clustering **(Fig. S3A)**. A total of four datasets were used for this evaluation, each accompanied by published annotations serving as ground truth. These datasets, including iPSC[18], HCC[28], and T cell datasets[29], are described in detail in **Table S3**.

We first evaluated the performance of AEnet in the ASP identification **(Fig. S3A-C)**. Current methods, such as BRIE, Outrigger, and MARVEL, are all fundamentally annotation-dependent and therefore unable to detect unannotated AS events. Additionally, these tools are limited in their ability to detect MSE **(Fig. S3B, Table S4)**. Since MARVEL has been compared with other methods and demonstrated to be the optimal one in its category in a previous study, we next compared the AS events detected by AEnet and MARVEL using the demo dataset of MARVEL, which includes induced pluripotent stem cells (iPSCs) and iPSC-derived endoderm cells **(Fig. S3C)**[30]. MARVEL identified a total of 20,509 SE, 1,279 MXE, 8,295 RI, 5,163 A5SS, 5,832 A3SS, 5,818 AFE, 2,072 ALE, and 0 MSE **(Fig. S3D)**. In MARVEL's iPSC dataset, AEnet detected 722,278 additional AS patterns (63% unannotated) across 12,866 genes, with MSE detection 3.2-fold higher than MARVEL **(Fig. S3E-F).** These findings highlight AEnet's comprehensive capability to detect a wide range of AS events—including unannotated and complex patterns—except for intron retention events. Compared to our homologous method DESJ-detection, AEnet overcomes critical limitations in low-depth robustness. DESJ-detection's single-junction PSI calculation leads to 38% error rates in low-coverage scenarios **(Fig. S3G-H)**. In contrast, AEnet mitigates this by focusing on junction reads with shared splice sites (requiring ≥5 supporting reads) and transforming read distributions into transcript usage ratios. This strategy reduced PSI error by 47% ($p<0.001$, **Fig. S3I**). Taken together, AEnet outperforms existing methods in detecting unannotated alternative splicing events, resolving complex AS patterns, and ensuring reliable quantification across varying data depths.

Low-count ASPs may introduce random fluctuations in PSI estimates, potentially leading to inaccurate quantification, which in turn can affect the construction of ASP-Exp links and the identification of anchor ASPs **(Fig. S4A)**. To evaluate the impact of count thresholds on these outcomes, we systematically tested a range of minimum read count thresholds: 0, 3, 5, 7, and 9 **(Fig. S4A)**. Using a threshold of 0 yielded the highest number of ASP-Exp links, with ~40% classified as "specific." However, 99% of these specific links were supported by fewer than two samples, suggesting they were likely artifacts of random fluctuations rather than meaningful biological associations

(Fig. S4B-C). Similarly, the 0-read threshold also led to an inflated number of anchor ASPs, most of which were linked to low-confidence, sample-specific signals **(Fig. S4D)**. In contrast, applying thresholds of ≥3 substantially reduced these spurious associations. Importantly, most biologically meaningful ASP-Exp links were retained even when moderate thresholds (>0) were applied **(Fig. S4E).** Based on these results, AEnet uses a default threshold of five supporting reads to ensure PSI robustness while minimizing noise.

While filtering low-count ASPs improves analytical reliability, we also considered the potential risk of excluding rare but biologically relevant splicing events. To address this, AEnet defines an ASP as "valid" in a sample only if it is supported by ≥5 reads across ≥20 cells. ASPs failing this criterion are excluded from downstream analysis, as their sparsity compromises the reliability of similarity estimates between splicing and expression. To assess whether this filtering excludes informative low-abundance ASPs, we stratified all ASPs into five categories based on their support across cells: Invalid: ≤20 cells (excluded); Type 1: >20–30 cells; Type 2: >30–40 cells; Type 3: >40–50 cells; Type 4: >50 cells. An ASP was assigned to the highest applicable category if it met the criteria in ≥3 samples **(Fig. S4F)**. As expected, higher-support ASPs (fewer NaNs) showed stronger ASP-Exp associations. Nonetheless, ~30% of Type 1 ASPs (i.e., relatively rare but retained) still showed significant correlations with gene expression, and four were identified as anchor ASPs **(Fig. S4G-H)**, indicating their functional relevance. In summary, our results support the use of both read count and sample support thresholds to reduce noise while preserving biological signal. AEnet remains capable of capturing meaningful but infrequent ASPs and provides user-defined thresholding to support flexible analysis tailored to specific research goals.

Finally, to assess the performance of AEnet in the identification of ASP clusters, an ASPs-ASPs similarity (Jaccard index) matrix was generated with increasing levels of noise to evaluate AEnet's effectiveness in ASP clusters prediction, **(Fig. S5A-B)**. Using a supervised hierarchical clustering method, AEnet demonstrated a high accuracy consistency score of approximately 0.9 between the background and the clusters identified, even when noise levels reached 80% **(Fig. S5C-D)**. These results demonstrate AEnet's robustness in identifying ASP clusters despite noise. Furthermore, we evaluated AEnet's performance using more realistic noise models, specifically Gaussian and Poisson noise. We simulated increasing levels of both Gaussian and Poisson noise and generated ASP–ASP similarity matrices (Jaccard index) under each noise condition to assess AEnet's robustness in ASP clustering **(Fig.**

S6A-B, D-E). Using a supervised hierarchical clustering approach, AEnet consistently achieved a high accuracy score—approximately 0.9—between the ground truth and the predicted clusters, even under noise levels as high as 90% for both noise models **(Fig. S6C, F)**. These results demonstrate AEnet's resilience to biologically relevant noise, further supporting its reliability in identifying splicing patterns in realistic, noisy settings.

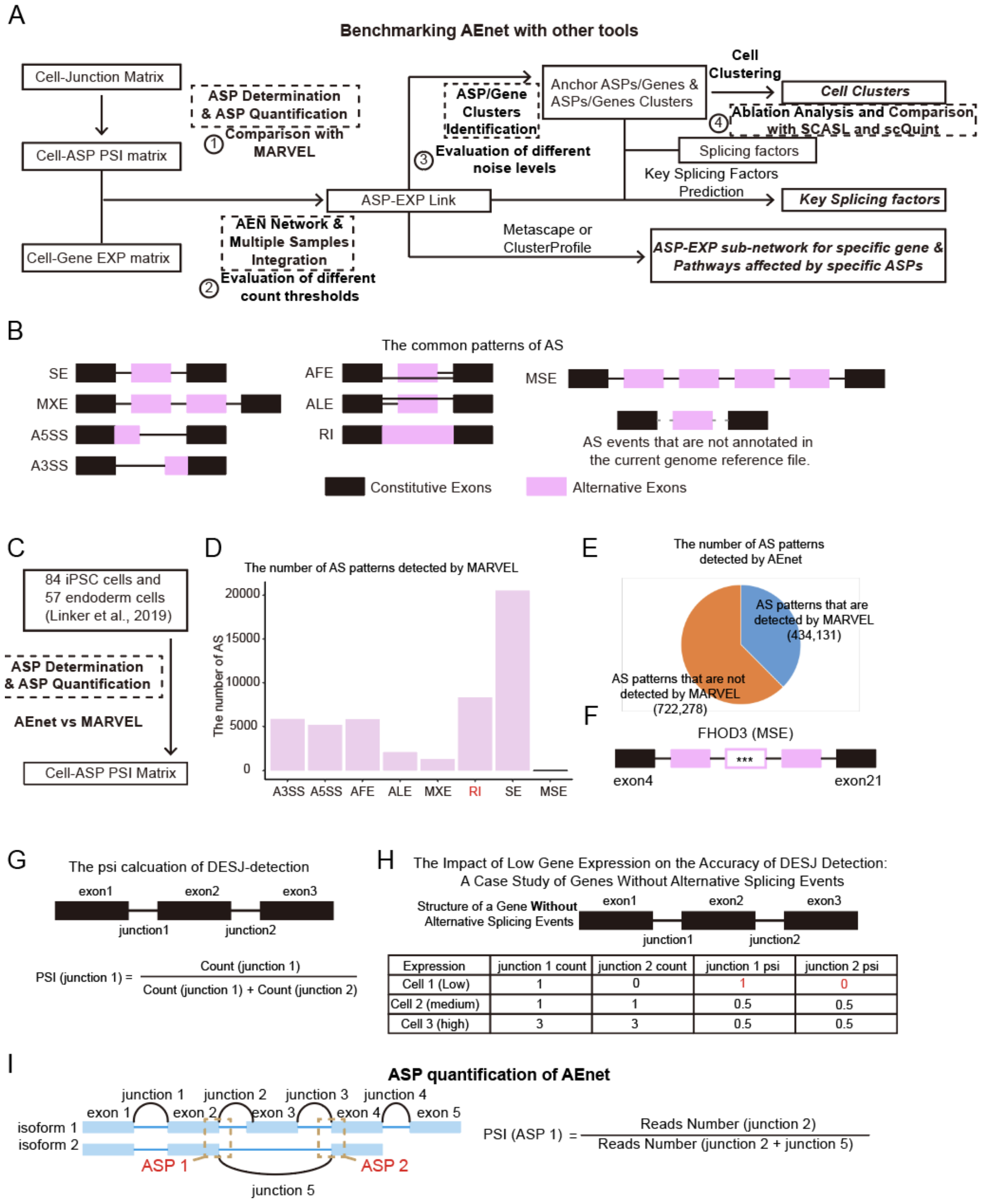

Figure S3. The evaluation of AEnet. A. Schematic overview of the pipeline for the comprehensive evaluation of AEnet, organized into four steps: (1) ASP determination

and quantification (compared with MARVEL), (2) AEN network construction and multi-sample integration (evaluation of different count thresholds), (3) ASP/gene cluster identification (assessment under varying noise levels), and (4) cell clustering (ablation analysis and comparison with SCASL and scQuint). **B.** The common types of Alternative splicing. **C.** The comparison between AEnet and MARVEL in the detection of ASPs. **D.** The number of alternative splicing events detected by MARVEL. **E.** The number of alternative splicing events detected by AEnet as well as the comparison with MARVEL. **F.** The demo example of FHOD3 for the rare patterns detected by AEnet. **G.** Schematic illustration of PSI calculation in DESJ-detection. **H.** Impact of Low Gene Expression on the Accuracy of DESJ Detection: A Case Study Using Genes Without Alternative Splicing Events. **I.** Schematic Diagram of the calculation of PSI for Alternative Splicing Pattern (ASP).

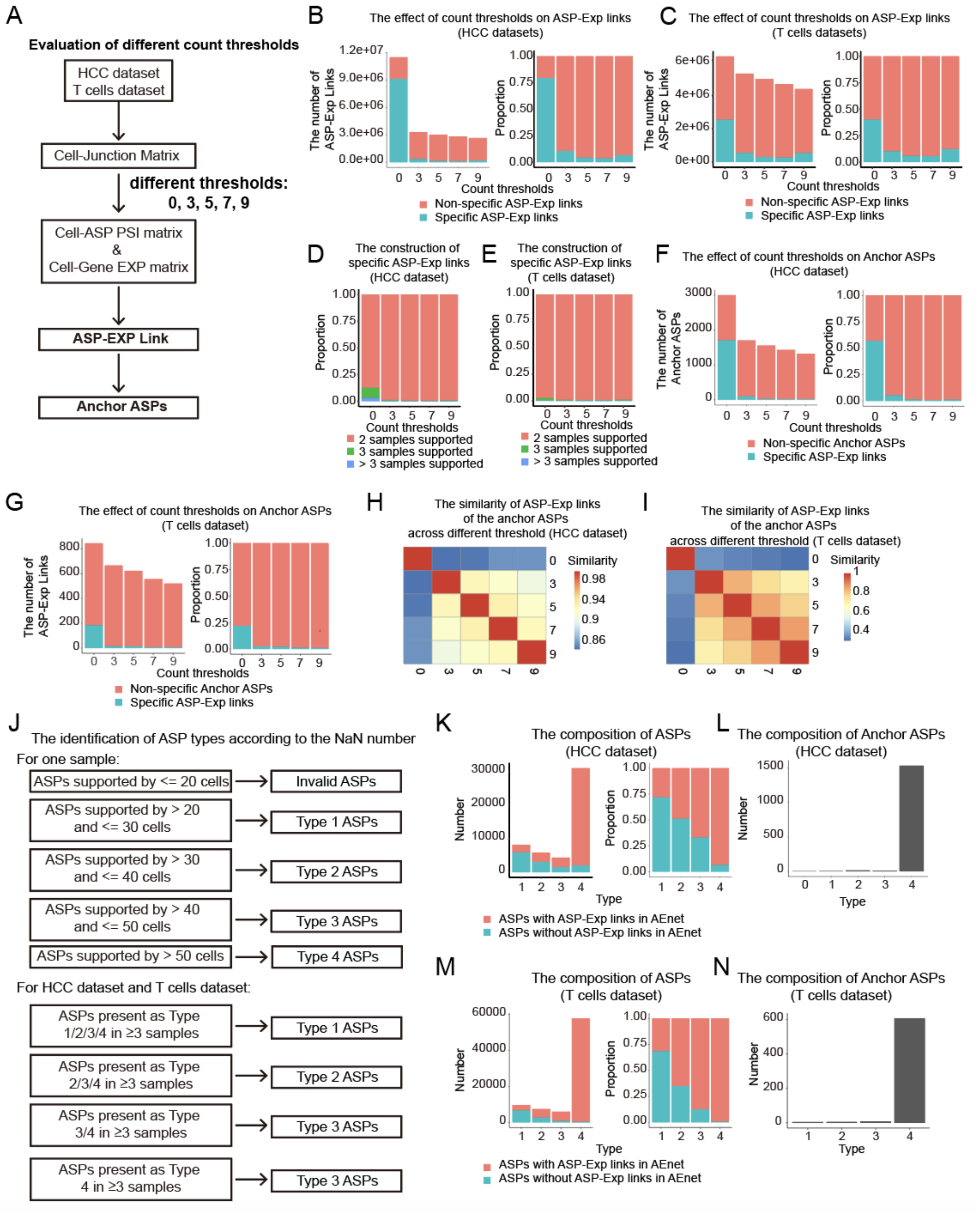

Figure S4. Evaluation of different count thresholds on AEnet performance. A. Schematic overview of benchmarking strategy for evaluating different count thresholds on the construction of ASP-EXP Link and Anchor ASPs. **B-C.** Quantification of ASP-EXP links across varying count thresholds, with color coding denoting link specificity in the HCC dataset (B) and the T cell dataset (C).**D-E.** Patient-level validation rates for specific ASP-EXP links at different expression count cutoffs in the HCC dataset (D) and the T cell dataset (E). **F-G.** Quantification of anchor ASPs across varying count thresholds, with color coding denoting link specificity in the HCC dataset (F) and the T cell dataset (G). **H-I.** The similarity of anchor ASPs across different expression count

cutoffs in the HCC dataset (H) and the T cell dataset (I). **J.** Schematic Diagram of the identification of ASP types for one sample or one dataset. **K.** Quantitative analysis of ASP subtypes categorized by presence (blue) or absence (gray) of ASP-EXP links in the HCC dataset. **L.** Frequency distribution of anchor ASPs stratified by ASP subtype classification in the HCC dataset. **M.** Quantitative analysis of ASP subtypes categorized by presence (blue) or absence (gray) of ASP-EXP links in the T cells dataset. **N.** Frequency distribution of anchor ASPs stratified by ASP subtype classification in the T cells dataset.

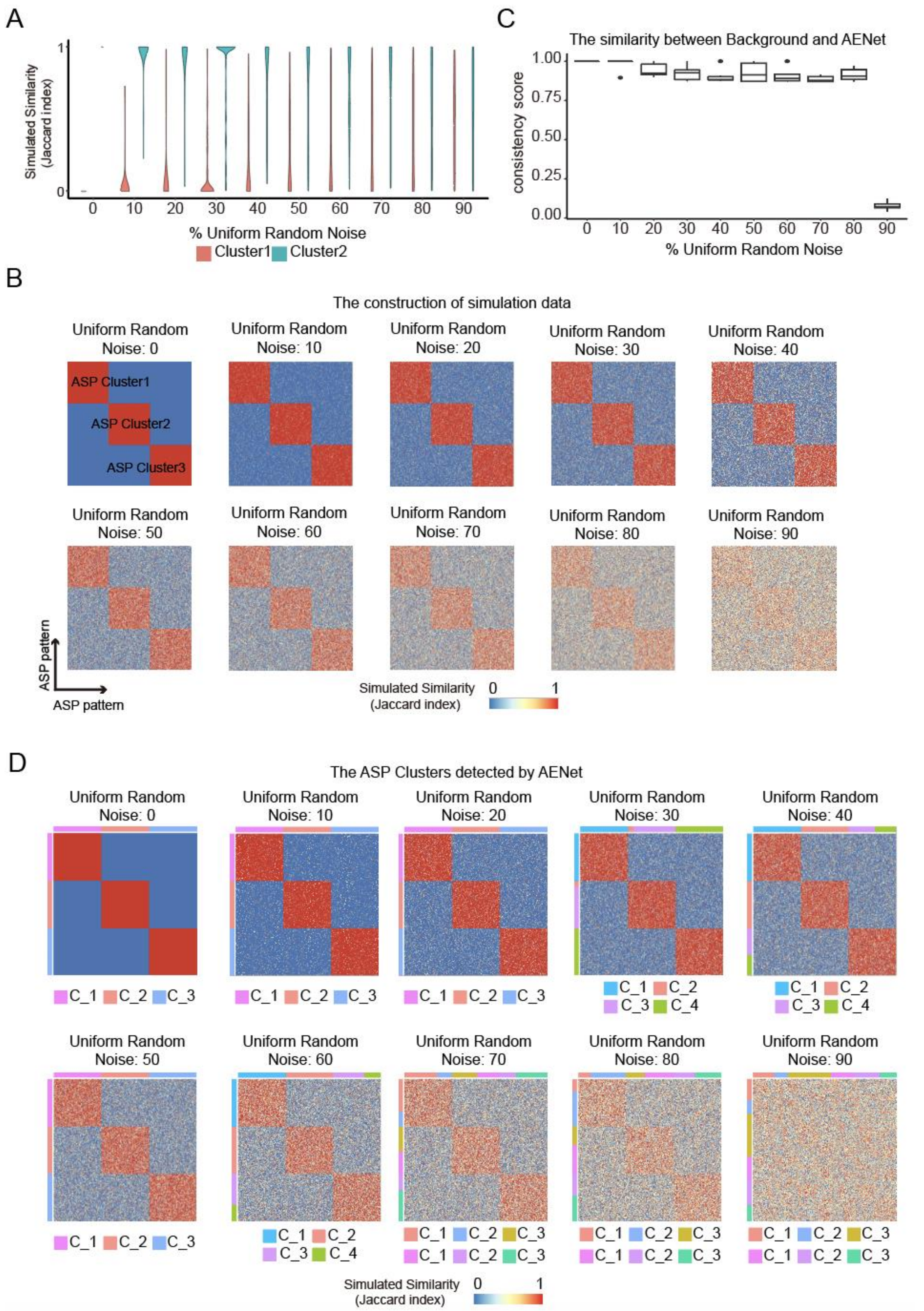

Figure S5. The evaluation of ASP clusters prediction of AEnet with random noise. **A-B.** Simulated ASPs-ASPs similarity matrix (B) was created with increasing noise (A). **C.** Box plots present the Jaccard index of ASP clusters between the background and AENet. **D.** ASP clusters identified by AENet in the simulated datasets.

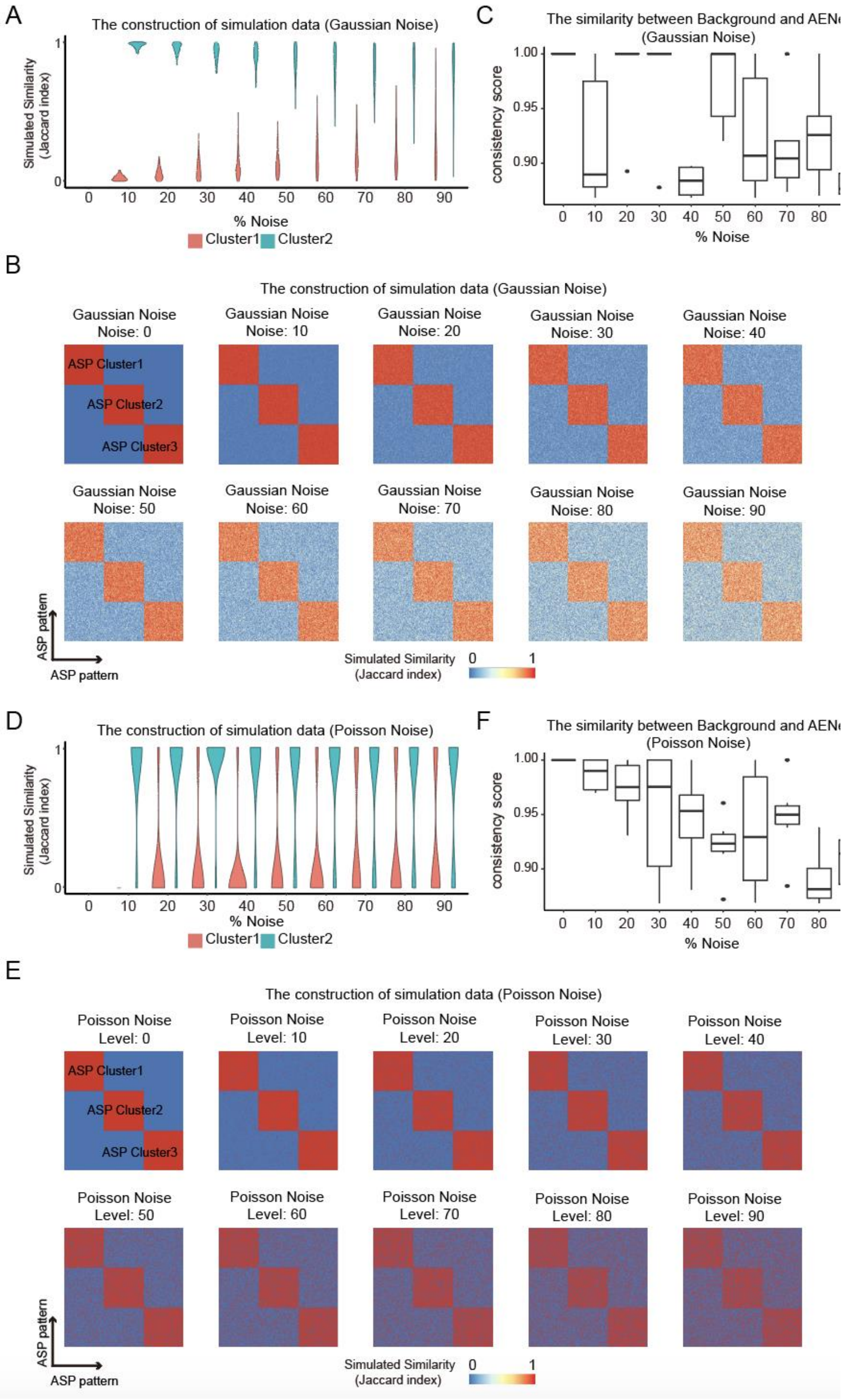

Figure S6. The evaluation of ASP clusters prediction of AEnet with gaussian noise and Poisson noise. A-B. Simulated ASPs-ASPs similarity matrix (B) was created with increasing gaussian noise (A). **C.** Box plots present theJaccard index of

ASP clusters between the background and AENet with the increasing gaussian noise. **D-E.** Simulated ASPs-ASPs similarity matrix (E) was created with increasing poisson noise (D). **F.** Box plots present the Jaccard index of ASP clusters between the background and AENet with the Poisson noise.

Benchmarking AEnet Against Existing Methods

While the above analyses validate AEnet's basic performance in characterizing splicing heterogeneity, its practical value in the field requires comparison with existing state-of-the-art methods. We thus benchmarked AEnet against established tools for single-cell AS analysis. We next evaluated AEnet's performance in capturing cellular heterogeneity using the three benchmarking datasets. Comprehensive ablation analyses, using published cell type annotations as ground truth, demonstrated that the joint ASP-EXP model (AEnet) consistently outperformed the standalone AS-only (Anet) and expression-only (Enet) approaches **(Fig. 2A)**. Specifically, the median ARI scores were 0.81 (AEnet), 0.68 (Anet), and 0.77 (Enet) for the iPSC dataset; 0.58 (AEnet), 0.10 (Anet), and 0.39 (Enet) for the HCC dataset; and 0.42 (AEnet), 0.10 (Anet), and 0.32 (Enet) for the T cell dataset **(Fig. 2B–D)**. These results highlight that integrating ASP features with gene expression significantly enhances clustering resolution and biological interpretability. Moreover, AEnet produced the most informative low-dimensional embeddings across all datasets **(Fig. S7),** accurately reconstructing the cellular architecture in iPSCs, delineating major lineages in HCC, and resolving functionally distinct T cell subsets—tasks in which AS-only or EXP-only models performed suboptimally.

We further compared AEnet with established splicing-aware clustering methods, including SCASL [31] and scQuint [32]. Across all three datasets, AEnet consistently outperformed SCASL in clustering accuracy, with higher ARI scores: iPSC (0.81 vs. 0.37), T cell (0.42 vs. 0.29), and HCC (0.58 vs. 0.35) **(Fig. 2E–G)**. In the iPSC dataset, SCASL failed to distinguish iPSCs from NPCs, while AEnet clearly separated these populations **(Fig. S8A)**. In the HCC dataset, AEnet effectively resolved lymphoid, myeloid, and malignant epithelial lineages, whereas SCASL showed poor separation between immune cell types **(Fig. S8B)**. Similarly, in the T cell dataset, SCASL generated overlapping clusters, failing to delineate functional T cell subsets, in contrast to the well-separated clusters produced by AEnet **(Fig. S8C)**. Since scQuint primarily uses a variational autoencoder (VAE) to generate embeddings, we applied clustering to the scQuint-derived embeddings using default parameters. In the iPSC dataset—characterized by relatively simple cellular composition—AEnet and scQuint showed comparable performance. However, in the more complex T cell and HCC datasets, scQuint produced overlapping clusters and failed to resolve key subpopulations **(Fig. S8D)**. Together, these results confirm that joint modeling of alternative splicing and gene expression enables AEnet to more accurately capture cellular heterogeneity compared to other splicing-based methods.

We next assessed AEnet's performance in mitigating batch effects and identifying AS-driven cell heterogeneity. Compared to SCASL—the current leading single-cell clustering tool based on AS—AEnet showed improved robustness across multiple samples **(Fig. S9)**. In lung cancer datasets[33], AEnet uncovered shared AS heterogeneity across patients, whereas SCASL mainly reflected patient-specific batch effects **(Fig. S9A–B)**. Similarly, in the CRC [29] and RHCC[28] T cell datasets, SCASL failed to detect AS heterogeneity in T cells with minimal batch effects, while AEnet successfully captured these patterns **(Fig. S9C–D)**. These results demonstrate AEnet's ability to detect biologically meaningful AS heterogeneity even in the presence of technical noise or batch variation, making it well-suited for large-scale, multi-sample studies.

To further demonstrate AEnet's versatility, we applied it to a widely used 10x Genomics PBMC dataset **(Fig. S9E–G)** [34]. Due to the limited sequencing depth of 10x platforms, AEnet detected insufficient splicing events for robust AS-based analysis. We therefore focused on alternative polyadenylation (APA), another form of isoform regulation. In this dataset, AEnet identified 190 anchor APA events and defined seven APA-based cell clusters **(Fig. S9E).** These clusters aligned well with known immune cell types, including B cells, $CD4^+$ T cells, $CD8^+$ T cells, dendritic cells, macrophages, mast cells, monocytes, neutrophils, NK cells, and regulatory T cells **(Fig. S9F)**. Clustering performance was quantitatively supported by a median ARI of 0.83 and NMI of 0.78 **(Fig. S9G)**. These results highlight AEnet's ability to uncover isoform-level heterogeneity in shallow-depth datasets, although we note that AS-based analyses remain more challenging in such settings due to limited read coverage.

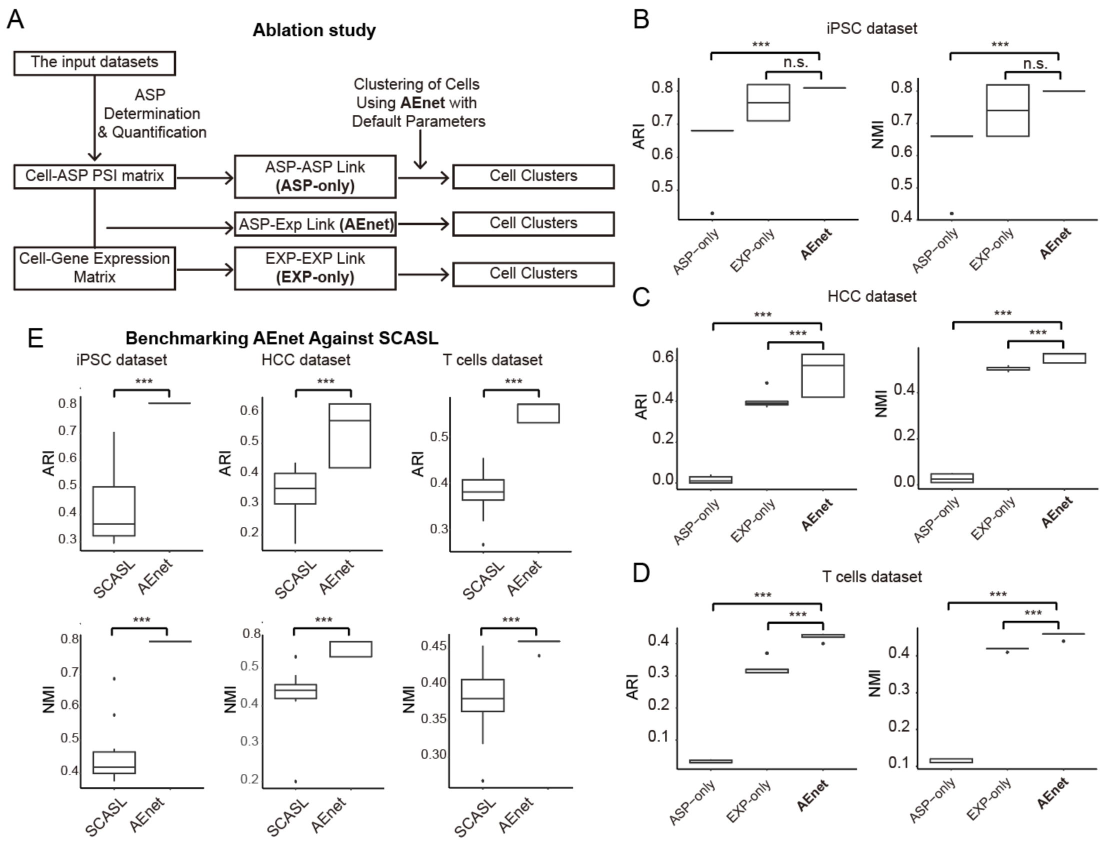

Figure 2. Benchmarking AEnet Against existing methods. A. Schematic diagram of the assessment pipeline for ASP-only, RNA-only, and joint clustering analyses, with all other processing steps remaining identical to those in AEnet. **B-D.** Quantitative benchmarking of clustering concordance using (upper) Adjusted Rand Index (ARI) and (lower) Normalized Mutual Information (NMI) metrics across the three networks. Statistical significance was assessed via one-sided Wilcoxon rank-sum tests. **E-G.** Quantitative benchmarking of clustering concordance using (left) ARI and (right) NMI metrics of AEnet and SCASL. Statistical significance was assessed via one-sided Wilcoxon rank-sum tests. * P < 0.05, ** P < 0.01, *** P < 0.001; n.s., not significant.

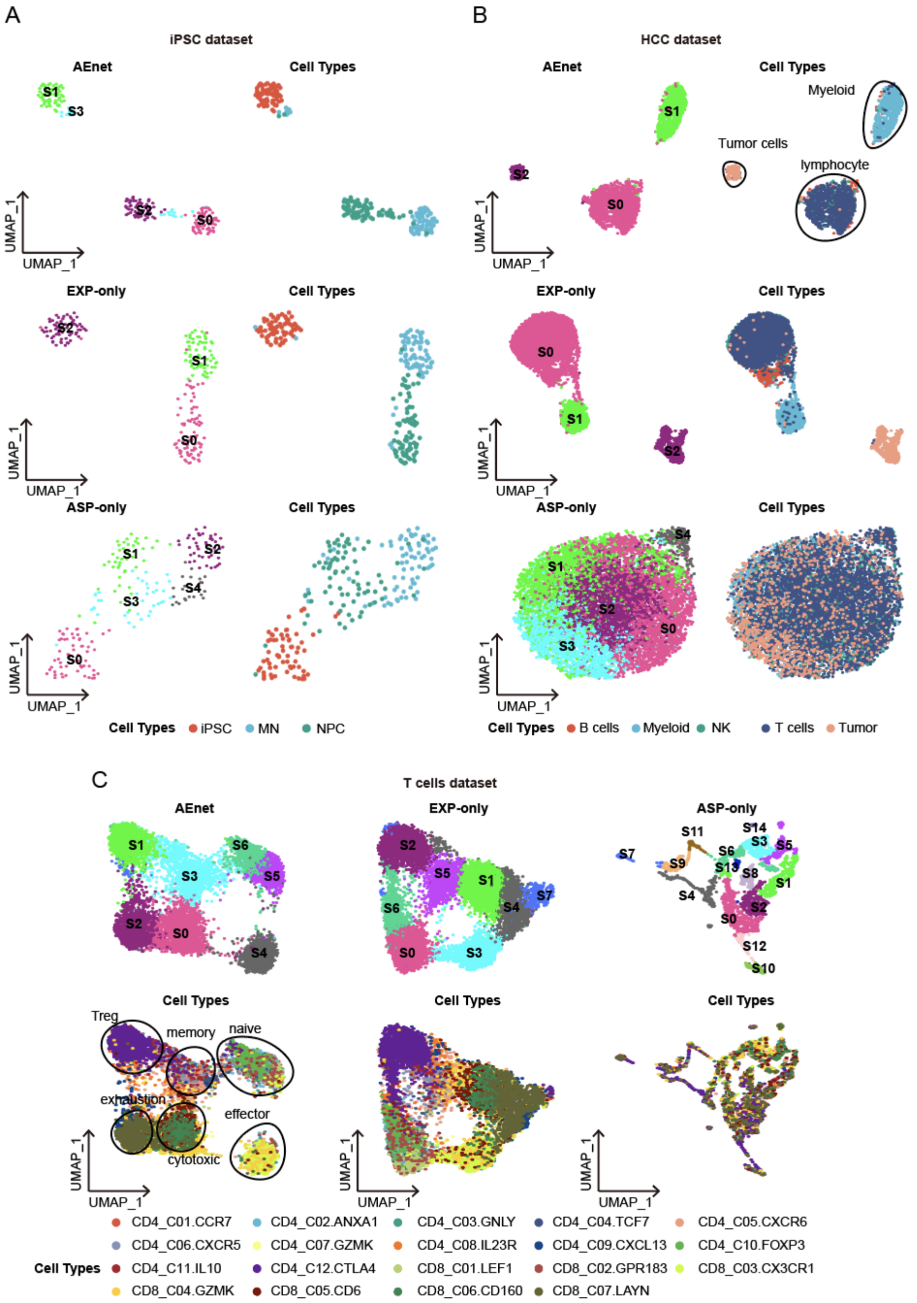

Figure S7. Ablation analysis for AEnet. A-C. Uniform Manifold Approximation and Projection (UMAP) visualizations showing the published cell type annotations (right) and the predicted clusters (left) derived from the ASP-EXP (AEnet) network (top), expression-only (EXP) network (middle), and ASP-only network (bottom) for (A) full-length iPSC and (B) hepatocellular carcinoma (HCC) single-cell RNA-seq datasets. (C) UMAP visualizations showing the published cell type annotations (bottom) and the predicted clusters (upper) derived from the ASP-EXP (AEnet) network (left), expression-only (EXP) network (middle), and ASP-only network (right) for the T cells datasets. Clustering solutions shown represent those with the highest adjusted Rand index (ARI).

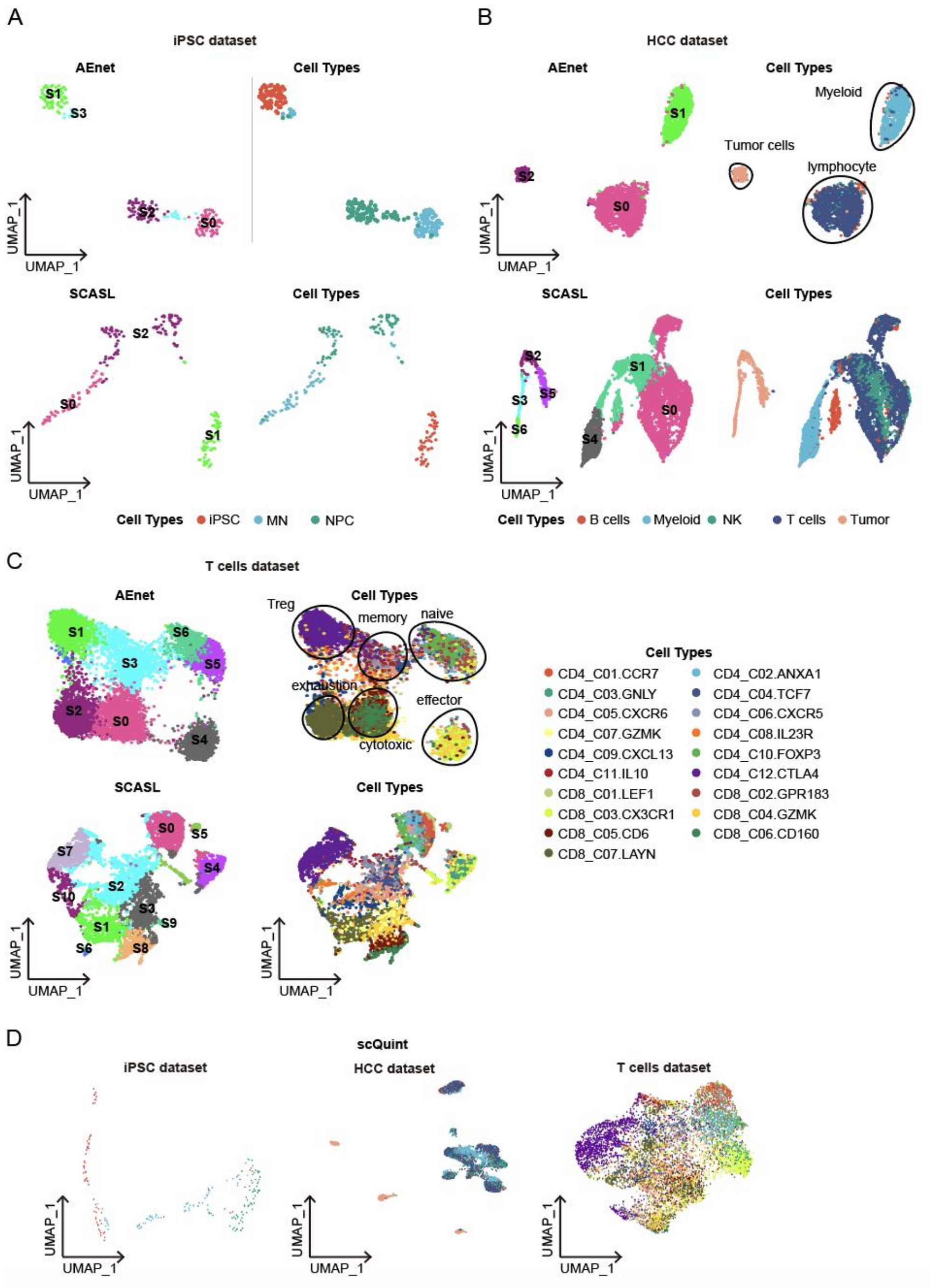

Figure S8. Benchmarking AEnet with SCAL and scQuint. A-C. UMAP visualizations showing the published cell type annotations (right) and the predicted clusters (left) derived from AEnet (upper) and SCASL (bottom) for: (A) full-length iPSC (B) hepatocellular carcinoma (HCC) single-cell RNA-seq datasets, and the (C) T cell dataset. Clustering solutions shown represent those with the highest adjusted Rand index (ARI). **D.** UMAP visualizations demonstrate the relative performance of scQuint (right) in: full-length iPSC dataset, T cells sequencing data, and hepatocellular carcinoma (HCC) single-cell RNA-seq data.

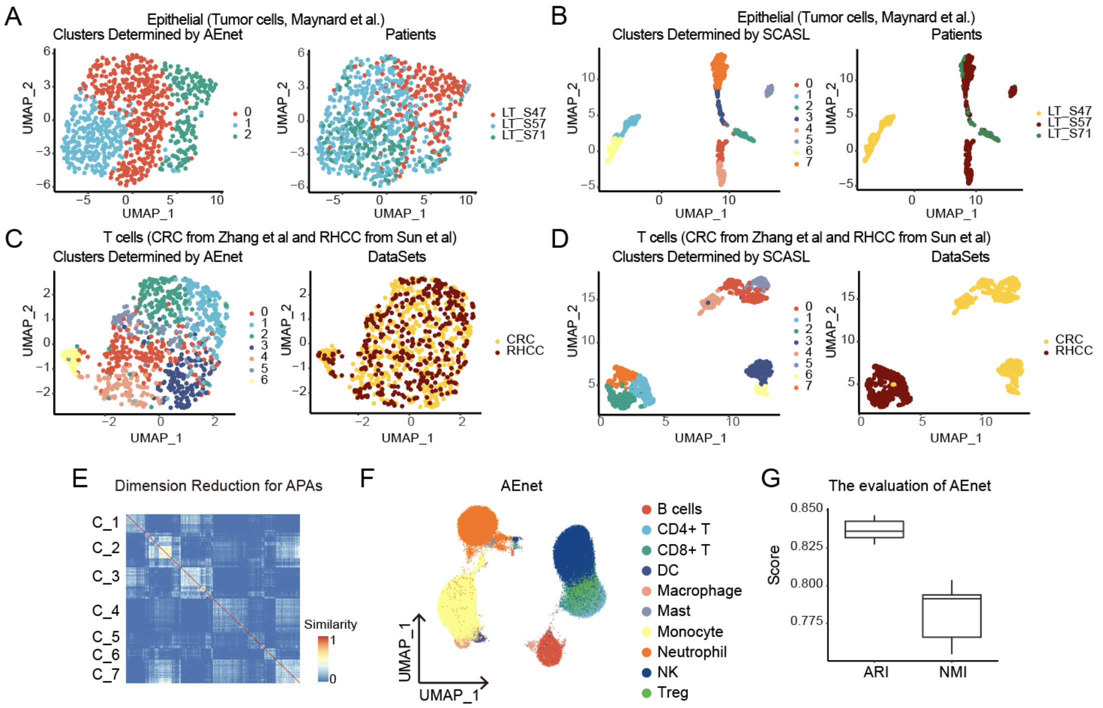

Figure S9. Performance comparison of AEnet and SCASL. A-B. UMAP shows the clustering of cell types determined by AEnet **(A)** and SCASL **(B)** (left panel) and patient clustering (right panel) for epithelial cells from multiple patients. **C-D.** UMAP shows the clustering of cell types determined by AEnet **(C)** and SCASL **(D)** (left panel) and patient clustering (right panel) for T cells from the CRC (Colorectal cancer) and RHCC (Recurrent Hepatocellular carcinoma) datasets. **E.** Heatmap showing APA classes derived from dimensionality reduction using AEnet. **F.** UMAP visualization of cell clustering based on alternative polyadenylation profiles. **G.** Quantitative benchmarking of clustering performance using ARI and NMI, evaluated against the ground truth annotations of the PBMC dataset.

AEnet resolves tumor heterogeneity bias and identifies immunotherapy-nonresponsive tumor subpopulations

Due to inherent intra- and inter-tumor heterogeneity, grouping malignant cells solely based on either gene expression profiles or ASPs is challenging. Here, we showcase the power of AEnet in untangling the intricate ASP-EXP relationships using data from 1,286 tumor cells from six lung cancer patients with varying responses to immunotherapy, classified as normal (N), residual disease (RD), and progressive disease (PD) after therapy [33].

Based on the AEnet algorithm, the ASP similarity matrix revealed a distinct separation into six ASP clusters, which resulted in three cell subpopulations (**Fig. 3A-B**). The AEnet-defined clusters exhibited biased distribution across response groups, with PD dominated by S2, RD primarily comprising S0 cells, and N group containing the

majority of S1 cells (**Fig. 3C**). The marker genes of S1 (N) were enriched in alveolar signatures, including *AQP4*, *SFTPB/C/D*, *NKX2-1*, and *FOXA2 [35,36]*, while S2 (PD) was associated with elevated expression of prothrombin activation genes (*PLAT*, *PLAUR*), gap-junction proteins (*GJB2/3/*5), and the well-known EMT (epithelial-mesenchymal transition) marker *EPCAM* (**Fig. 3D**) [37–39].

From the perspective of ASP clusters, C_4 (ASP cluster 4) was notably co-occurring with S2 while being excluded from S1 (**Fig. 3E**). The top-ranked hub genes in this cluster were IK and CELF2, which exhibited opposite expression trends between normal and PD cells (**Fig. 3F-G**). CELF2 is a crucial splicing factor, and its downregulation has been reported to promote tumor progression in both pancreatic and breast cancers [40,41]. The role of IK remains unclear; however, we found it to be upregulated in a CRC cohort as responses to immunotherapy deteriorated (**Fig. 3H**)[42].

In addition, C_4 was enriched in inflammation-associated pathways, including the response to type II interferon, positive regulation of lymphocyte proliferation, and the adaptive immune system (**Fig. 3I**). Among the key genes related to inflammatory responses, CD74, the HLA-DR antigens-associated invariant chain, is reported to exhibit dual oncogenic and tumor-suppressive roles depending on the cancer type and specific microenvironment. In this lung cancer dataset, we found that ASPs in CD74 were exclusively dominated by the isoforms CD74-201 and CD74-202. CD74-202 is reported as the soluble form, which suppressed melanoma cell growth and induced apoptosis under IFN-γ stimulatory conditions [43,44]. The significantly differentiated ratio of CD74-202 to CD74-201 could directly indicate post-therapy responses in different cell groups (**Fig. 3J**).

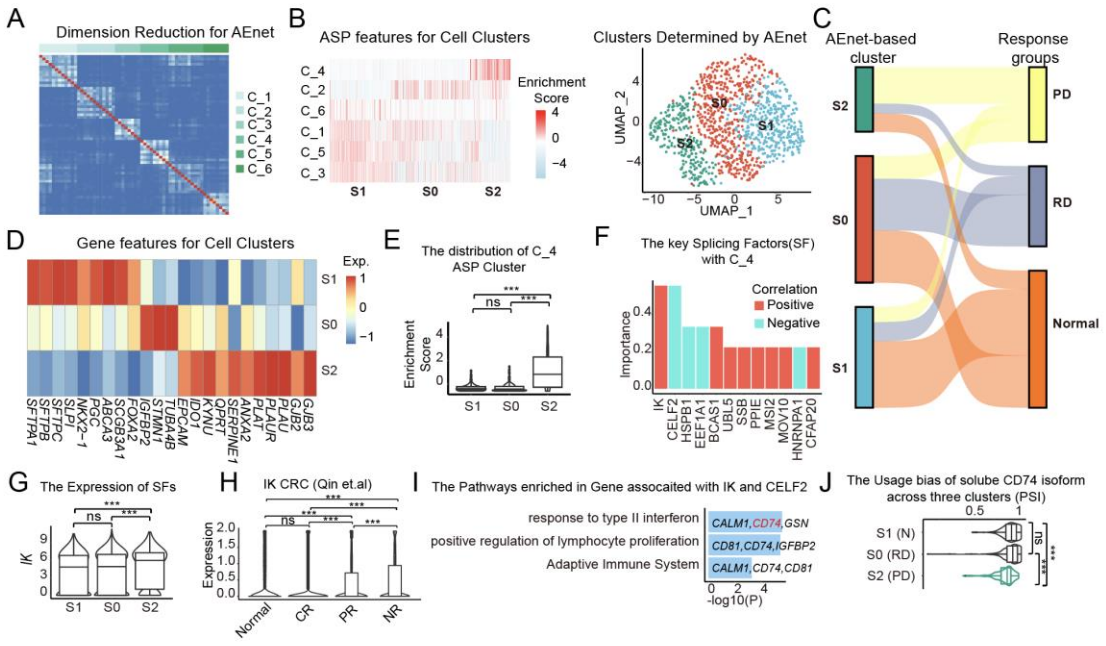

Figure 3. AEnet decreases the bias caused by tumor heterogeneity and uncovers mechanistic insights in immunotherapy response. A. The heatmap shows the ASP clusters from dimension reduction of AEnet. **B.** The heatmap displays the enrichment score of ASP clusters across cell clusters determined by AEnet (left panel). UMAP displays clustering of cell types determined by AEnet (right panel). **C.** The Sankey plot shows the overlapping of cells between response groups and Clusters determined by AEnet. **D.** The heatmap displays the expression of marker genes across cell clusters determined by AEnet. **E.** The distribution of C_4 enrichment scores across cell clusters determined by AEnet. **F.** The barplot displays the importance of splicing factors in the formation of C_4 ASP clusters. The color represents the correlation relationship between splicing factors and C_4 ASP clusters. **G.** The expression of IK across cell clusters. Statistical analysis was performed using the Student's t-test. **H.** The expression of IK within cells at different response groups. Statistical analysis was performed using the Student's t-test. **I.** The top 3 pathways enriched for the genes with alternative splicing patterns in C_4 ASP clusters. **J.** The alternative splicing patterns of CD74 and the PSI distribution across 3 clusters. Statistical analysis was performed using the Student's t-test. * $p < 0.05$, ** $p < 0.01$, *** $p < 0.001$.

AEnet reveals cellular splicing heterogeneity and its key splicing events in tumor-infiltrating T cells across various cancer types

The design of the AEnet algorithm largely bypasses batch effect issues in scRNA-seq analyses, making it particularly suitable for pan-cancer studies. To explore the cross-tissue capacity of this 'AS-Expression Network' concept, we curated tumor-infiltrating lymphocyte T cells from four cancer types: liver cancer (HCC) [45], colorectal cancer (CRC) [29], lung cancer (LUAD) [33], and recurrent liver cancer (RHCC) [28].

By selecting the top 1,223 ASP events from a total of 86,616 valid ASP-EXP links (supported by at least 2 samples across a minimum of 2 datasets), we identified 14 ASP clusters (referred to as C1–C14) (**Fig. S10A-C**). We subsequently generated 10 cell subpopulations (referred to as S0–S9) based on distinct compositions of ASP cluster-wise signatures across the 4 T cells datasets (**Fig. 4A and S10D**). Among these populations, S2 and S3 expressed naïve T cell markers (*IL7R, CCR7, LEF1*), S1 was characterized by memory T cell markers (*CD52, ANXA1, CREM*), S0 and S5 exhibited effector T markers (*NKG7, GZMA/B*), S4, S7, S8, and S9 represented the exhausted state of T cells (*PDCD1, CTLA4, HAVCR2*), while S6 corresponded to the proliferative state (*MKI67, TOP2A*) (**Fig. S10E**) [46]. Strikingly, AEnet did not partition T cells into canonical $CD4^+$ and $CD8^+$ subtypes, suggesting that alternative splicing primarily contributes to the transition of cell states rather than defining cell lineage (**Fig. 4B**).

Cross-cluster interactions observed in our data **(Fig. S10C)**. To further explore these relationships, we organized ASP clusters based on pairwise similarity and identified

three major regions, each comprising clusters with higher intra-region similarity than inter-region similarity **(Fig. S10F)**. Region 1 includes clusters C_7 and C_9, which exhibit the highest mutual similarity and are both predominantly enriched in naïve T cells. Interestingly, C_7 also shares similarity with C_5 and C_10, while C_9 is more closely related to C_4 and C_11. This suggests that C_7 and C_9 may represent bifurcating points leading to two naïve T cell differentiation trajectories—one toward memory T cells (C_5 and C_10), and the other toward effector T cells (C_4 and C_11) **(Fig. S10H-J)**. Supporting this, C_7 is enriched during the naïve-to-memory transition, while C_9 is enriched along the naïve-to-effector axis **(Fig. S10G)**. Region 2 comprises clusters C_11, C_4, and C_14, which show strong inter-cluster similarity. C_4 acts as a central node, connecting C_11 and C_14, suggesting a potential progression from effector T cells (C_11) to effector memory T cells (C_4), and eventually to exhausted T cells (C_14). Notably, C_11 also shares similarity with C_9, and C_14 with C_1—both associated with exhausted states—indicating a continuous exhaustion trajectory **(Fig. S10H)**. Region 3 implies an alternative exhaustion pathway, involving clusters C_5, C_10, and C_12. Here, C_10 links C_5 (memory/naïve-enriched) and C_12 (exhausted-enriched), forming a sequence akin to that in Region 2. Similarity between C_5 and C_7, and between C_12 and C_1, further supports a parallel differentiation path from naïve/memory T cells to exhaustion **(Fig. S10I)**. In summary, while rigid clustering provides discrete groupings, our similarity-based regional analysis reveals underlying transitions and trajectories among ASP-defined clusters. These findings highlight the dynamic continuum of T cell state transitions and underscore the value of complementary methods in capturing intermediate cell states that may be overlooked by strict partitioning.

When focusing on ASP clusters, the exclusive deficiency of C_5 in effector T cells (S0 and S5) and C_8 in proliferating T cells (S6) were two notable signatures demonstrating the correlation between specific ASPs and cell population heterogeneity (**Fig. 4C**). Among the top 10 common splicing factors, the C_5 ASP cluster positively correlated with EEF1A1 and PABPC1, which were partially aligned with ASPs primarily occurring in naïve T cells (clusters C_7, C_9, and C_13) (**Fig. 4D-E**). In contrast, C_8 was negatively correlated with Small Nuclear Ribonucleoprotein 25 (SNRNP25), a splicing factor involved in spliceosome assembly and function,intron excision and exon ligation[47]. The upregulation of SNRNP25 (reflected as a double negative correlation) in proliferating T cells served as a clear marker, identifying this cell type solely through splicing factors **(Fig. S10K)**.

C_5 was also correlated with HSPA1A/B, splicing factors contributing to exhausted T cells (C_1, C_2, C_3, C_12, and C_14). To explore the functional implications of C_5, we performed pathway enrichment analysis on its hub genes (**Fig. 4F**). This revealed a previously unannotated splicing variant of FYB1 (referred to as FYB_new), which utilizes chr5_39217636 as the end of the first exon of FYB1—a site not documented in the GRCh38.p14 reference genome. This splicing variant is associated with

pathways involved in second messenger generation, immune response-regulating signaling, and cell surface receptor signaling, all critical processes for achieving effector cell status (**Fig. 4G-H**). In contrast, FYB1-212 was associated with pathways linked to naïve and memory T cells.

Among the top splicing factors, HNRNPLL ranked third and exhibited a strong positive correlation with ASP clusters associated with exhausted T cells. It was also linked to unfavorable clinical prognosis (**Fig. 4I**). Genes regulated by HNRNPLL were enriched in thymic T cell selection, axon guidance, and leukocyte activation (**Fig. S10L**). Among the hub genes, CD3D, the most canonical T cell marker, exhibited two isoforms with distinct PSI distributions between exhausted and other T cells (**Fig. S10M**). Exhausted T cells preferentially spliced into isoform CD3D-202, which is linked to the PD-1 signaling pathway, CD28 family-mediated costimulation, and TCR signaling, while other T cells primarily utilized CD3D-201 for differentiation (**Fig. S10N**).

Collectively, we demonstrate the capability of the AEnet algorithm for integrated scRNA-seq data analysis without the need for batch corrections. Our algorithm facilitates bioinformatic data mining for isoform usage preferences, and even the discovery of novel isoforms of functional importance, such as new ASPs in the FYB gene for effector T cells.

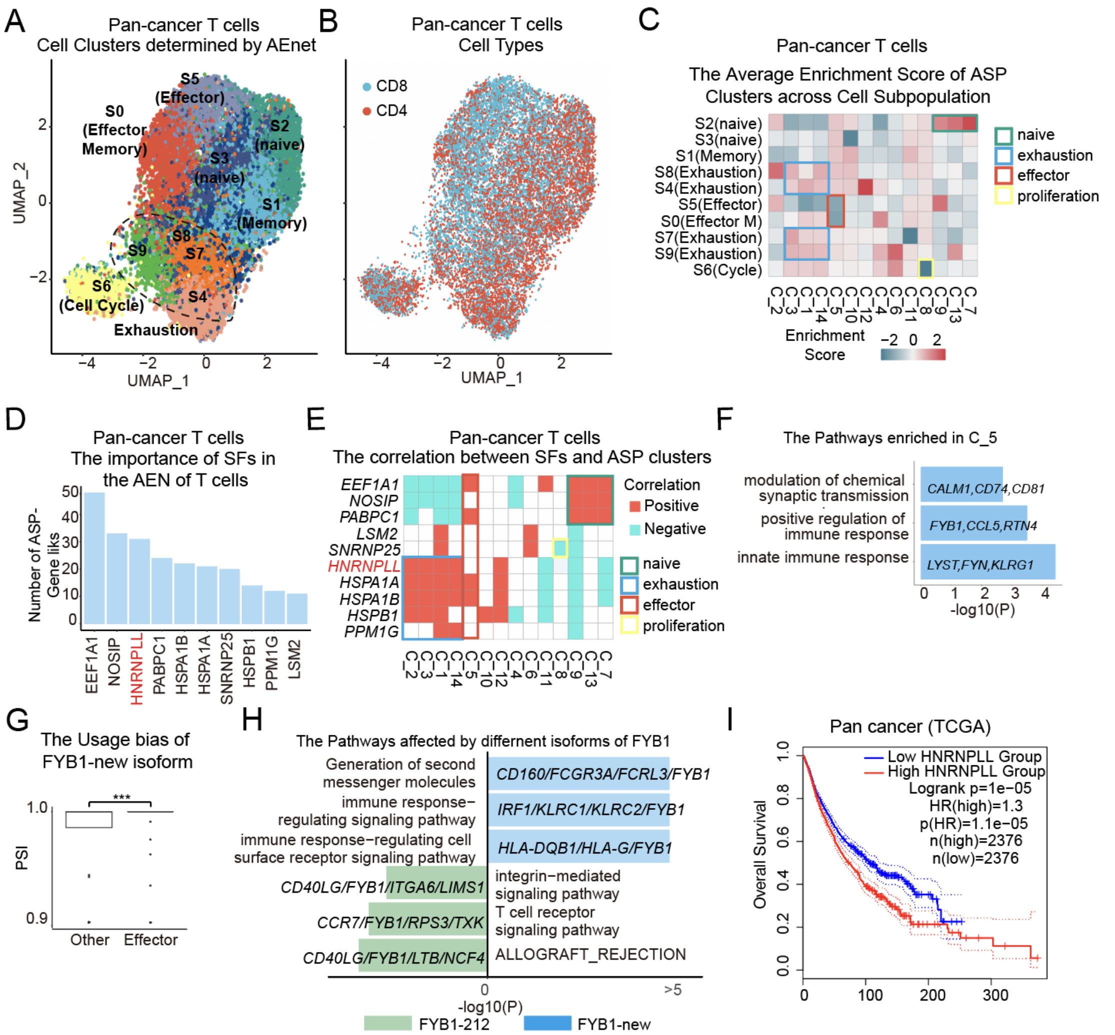

Figure 4. Application of the AEnet to pan-cancer tumor-infiltrating T cell single-cell data. A-B. UMAP displays clustering of cell types determined by AEnet (A) and lineage (B). **C.** The heatmap displays the enrichment score of ASP clusters across cell clusters determined by AS. **D.** The barplot displays the importance of splicing factors in the formation of the AEN network of the pan-cancer T cells. **E.** The relationship between ASP clusters and key splicing factors. **F.** The top 3 pathways enriched for the genes with alternative splicing patterns in C_5 ASP clusters. **G.** The PSI distribution of FYB1-new isoform within FYB1 across effector and other T cells. Statistical analysis was performed using the Student's t-test. **H.** The pathways enriched in the gene sets with different isoforms of FYB1. **I.** Kaplan-Meier analysis shows the overall survival of patients characterized by low (blue) or high (red) HNRNPLL in the TCGA cohort. Statistical analysis was performed using the log-rank test. * $p < 0.05$, ** $p < 0.01$, *** $p < 0.001$.

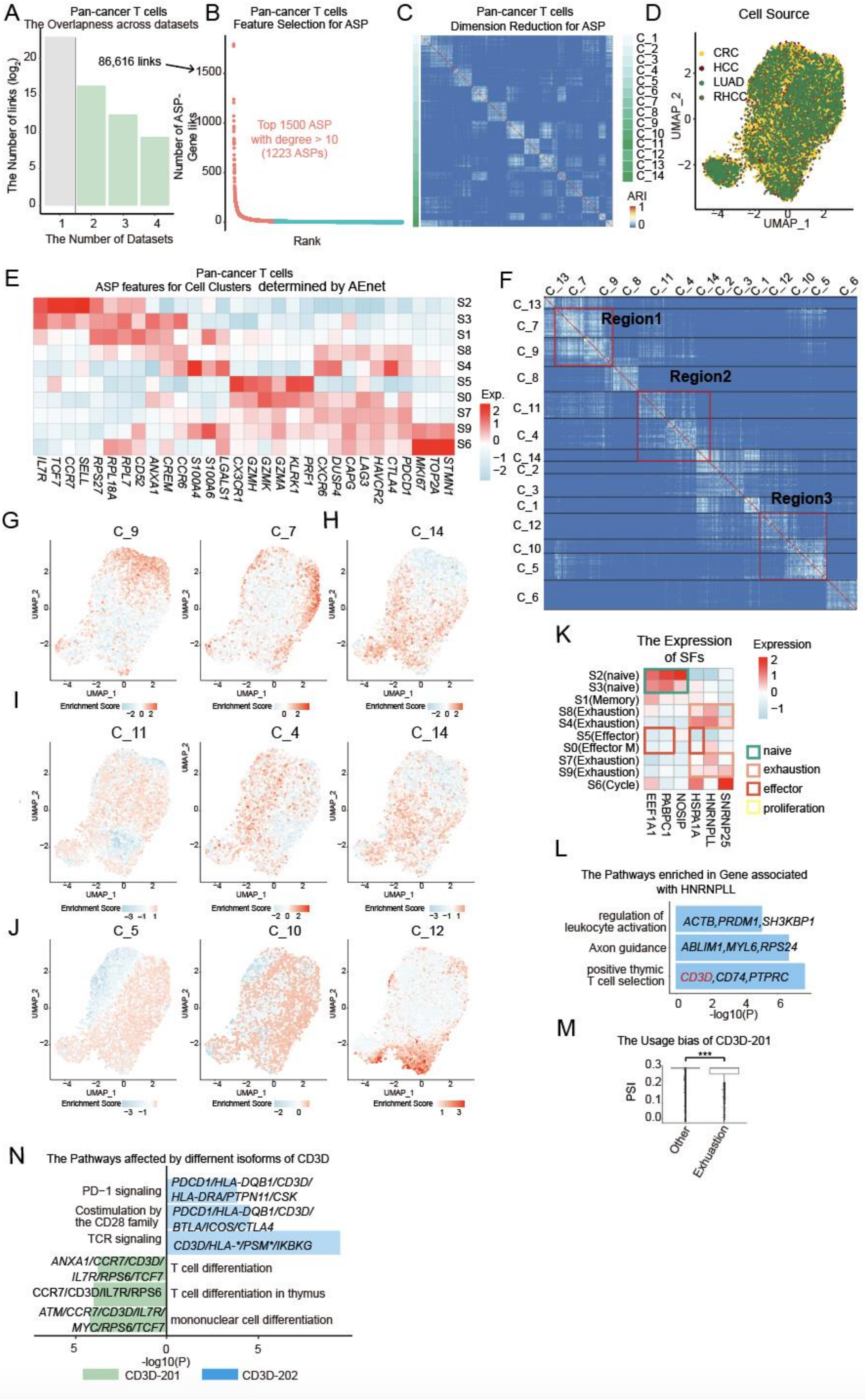
A
Pan-cancer T cells
The Overlapness across datasets
86,616 links
The Number of links (log2)
The Number of Datasets
B
Pan-cancer T cells
Feature Selection for ASP
Top 1500 ASP
with degree > 10
(1223 ASPs)
Number of ASP-Gene liks
Rank
C
Pan-cancer T cells
Dimension Reduction for ASP
ARI
D
Cell Source
CRC
HCC
LUAD
RHCC
UMAP_1
UMAP_2
E
Pan-cancer T cells
ASP features for Cell Clusters determined by AEnet
Exp.
S2
S3
S1
S8
S4
S5
S0
S7
S9
S6
IL7R
TCF7
CCR7
SELL
RPS27
RPL18A
RPL7
CD52
ANXA1
CREM
CCR6
S100A4
S100A6
LGALS1
CX3CR1
GZMH
GZMK
GZMA
KLRK1
PRF1
CXCR6
DUSP4
CAPG
LAG3
HAVCR2
CTLA4
PDCD1
MKI67
TOP2A
STMN1
F
Region1
Region2
Region3
G
H
I
J
Enrichment Score
K
The Expression of SFs
Expression
S2(naive)
S3(naive)
S1(Memory)
S8(Exhaustion)
S4(Exhaustion)
S5(Effector)
S0(Effector M)
S7(Exhaustion)
S9(Exhaustion)
S6(Cycle)
EEF1A1
PABPC1
NOSIP
HSPA1A
HNRNPLL
SNRNP25
naive
exhaustion
effector
proliferation
L
The Pathways enriched in Gene associated with HNRNPLL
regulation of leukocyte activation
ACTB,PRDM1,SH3KBP1
Axon guidance
ABLIM1,MYL6,RPS24
positive thymic T cell selection
CD3D,CD74,PTPRC
-log10(P)
M
The Usage bias of CD3D-201
PSI
Other
Exhuastion
N
The Pathways affected by different isoforms of CD3D
PD−1 signaling
PDCD1/HLA-DQB1/CD3D/
HLA-DRA/PTPN11/CSK
Costimulation by the CD28 family
PDCD1/HLA-DQB1/CD3D/
BTLA/ICOS/CTLA4
TCR signaling
CD3D/HLA-*/PSM*/IKBKG
ANXA1/CCR7/CD3D/
IL7R/RPS6/TCF7
T cell differentiation
CCR7/CD3D/IL7R/RPS6
T cell differentiation in thymus
ATM/CCR7/CD3D/IL7R/
MYC/RPS6/TCF7
mononuclear cell differentiation
-log10(P)
CD3D-201
CD3D-202

Figure S10. AEnet reveals cellular splicing heterogeneity and its key splicing events in tumor-infiltrating T cells across various cancer types. A. The barplot displays the selection of high quality links in the AEnet of Pan-cancer T cells. **B.** The selection of key alternative splicing patterns. **C.** The heatmap shows the ASP cluster from dimension reduction of AEnet. **D.** UMAP displays cell sources. **E.** The heatmap displays the expression of marker genes across cell clusters determined by AS. **F.** Heatmap of ASP clusters ordered by similarity, illustrating inter-cluster relationships within the pan-cancer T cell dataset. **G-J.** Distribution of enrichment scores for selected ASP clusters. **K.** The expression of key splicing factors across clusters determined by alternative splicing. **L.** The pathways enriched in genes with ASP associated with HNRNPLL. **M.** The PSI distribution of CD3D-201 isoform within CD3D within exhaustion and other T cells. Statistical analysis was performed using the Student's t-test. **N.** The pathways enriched in the gene sets with different isoforms of CD3D.

AEnet Uncovers Transitional Cell States and Key Splicing Factors in Embryonic Gastrulation

During embryogenesis, alternative splicing is a key mechanism that fine-tunes developmental pathways and controls cell fate decisions. Here, we apply AEnet to interrogate a scRNA-seq dataset of gastrulation-stage human embryos from the Human Developmental Biology Resource, elucidating how the AS process enables precise regulation of gene expression at this stage. The reference dataset comprises 1,195 cells (665 caudal, 340 rostral, and 190 yolk sac cells), with a median of 4,000 genes detected per cell[48].

Following the standard AEnet workflow, we first identified a total of 1,604 ASP events, 25 ASP clusters, and 11 cell populations (**Fig. 5A and S11A-C**). The sequential differentiation trajectory from epiblast cells (cell subpopulation 5, S5) to the primitive streak (S1), followed by the transition to endodermal cells (S6/S10) or mesoderm (S2/S0), ultimately leading to axial mesoderm (S7), was clearly discernible (**Fig. 5A, right panel**) [49]. Notably, the ASPs are assumed to be highly distinct among different cell types during embryogenesis. As a result, AEnet factorized the cell populations in a manner similar to those clustered based solely on RNA profilings (**Fig. 5B**, ARI = 0.304).

Epiblast cells (S5) are classic pluripotent stem cells, derived from the inner cell mass of the blastocyst and capable of differentiating into the three germ layers. For the highly correlated ASP clusters C_13 and C_17, PSIP1 and SNRPN were identified as key regulatory factors that maintain cell stemness (**Fig. 5C-D, S11D-E**). HNRNPAB and SRSF3 were associated with splicing decisions that resulted in longer junctions spanning genomic loci, observed more frequently in epiblast cells than in cells with reduced stemness (**Fig. 5E**). We verified this intriguing finding in an independent iPSC dataset (**Fig. S11F**)[18].

Some subpopulations defined by AEnet were in a transitional stage. For example, S2 shares RNA profiling similarities with both S1 and S0 (**Fig. 5B**). This cell population expressed relatively lower stemness signatures and higher mesodermal features compared to the primitive streak (S1) (**Fig. 5F**). The enriched pathways also indicated that S2 was an intermediate cell state between the primitive streak (S1) and mesoderm (S0) (**Fig. 5G**). We subsequently compared the ASP cluster compositions between S2 and S0, focusing on two of the most distinct ASP clusters, C_1 and C_18, for downstream analysis (**Fig. 5C**). As part of the routine analysis, we identified the top splicing factors and hub-gene-enriched pathways. SNRPD2, a core component of the spliceosome, emerged as a pivotal factor distinguishing these two mesoderm subtypes (**Fig. 5H and S11G**). Related to the genes in enriched pathways, TNRC6B displayed distinct isoform distributions across cell clusters (**Fig. 5I and S11H**). TNRC6B-205 was enriched in S2 and linked to the classical WNT, Notch, and MAPK signaling pathways. In contrast, TNRC6B-201 was prevalent in other cell types and associated with primary germ layer formation and other processes (**Fig. 5J**).

Moreover, we unveiled that ASP clusters C_2, C_5, C_7, C_16, and C_23 were pivotal for endoderm differentiation, with HSPB1 emerging as a core negative regulator of their formation. Clusters C_12, C_20, and C_24, crucial for yolk sac mesoderm development, were positively regulated by HSPB1 **(Fig. S11I-J)**. Lastly, ASP events in hepatic and erythrocyte development (C_3, C_4, C_6, C_8, C_11, C_14, C_22, and C_25) were governed by MBNL1 and HSPA5, both highly expressed in these cell types (**Fig. S11I-J**).

By exploring the alternative splicing landscapes in this human embryonic data (**Fig. 5K**), we not only clarified phenotypic subtleties along the AS-based developmental trajectory but also illuminated the complexity of AS mechanisms underlying cell differentiation and embryonic development, successfully demonstrating the capabilities of the AEnet algorithm.

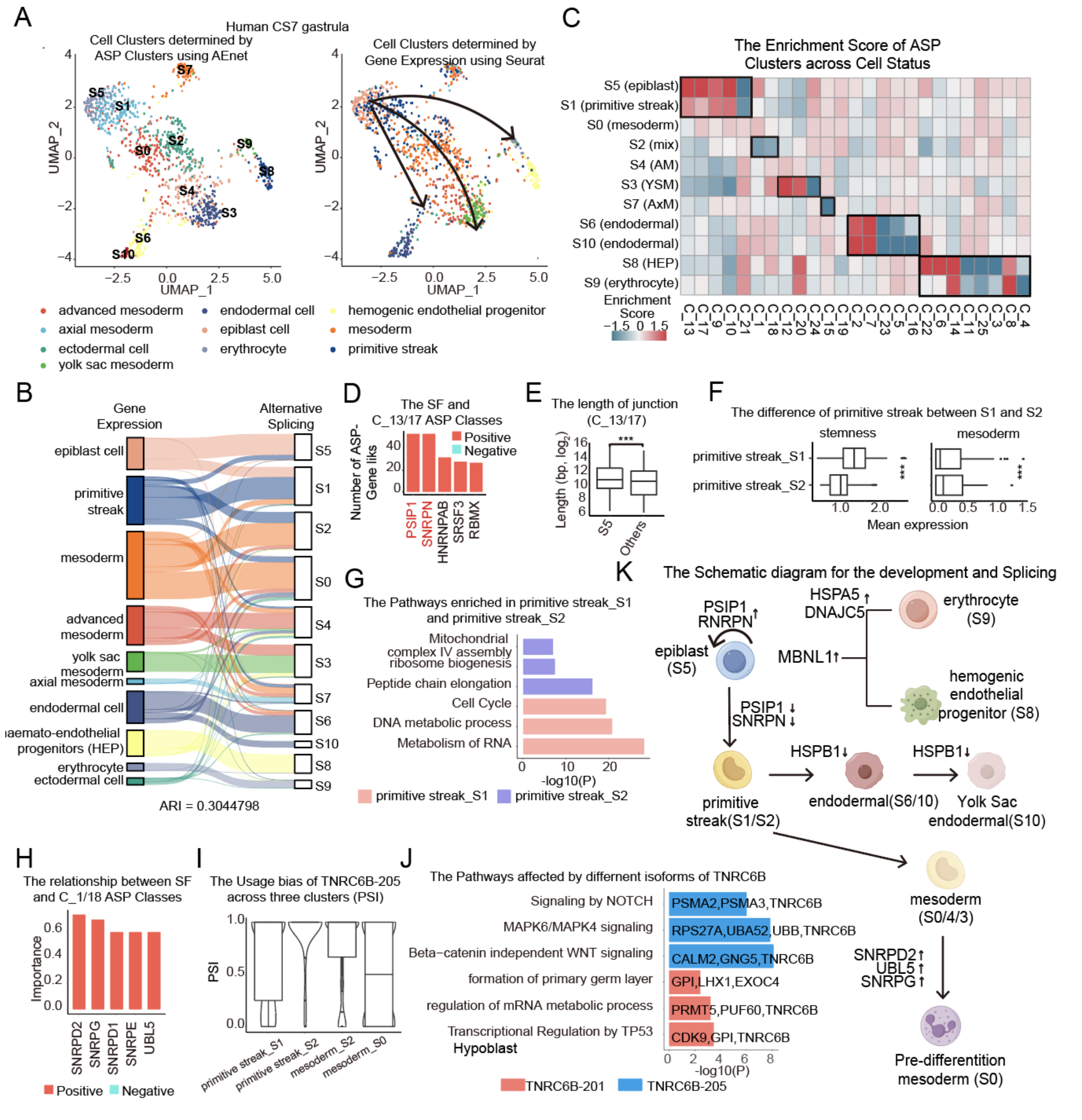

Figure 5. AEnet uncovers transitional cell states and key splicing factors in embryonic gastrulation. A. UMAP displays clustering of cells by cell type determined by Alternative Splicing (left panel), and gene expression (right panel). **B.** The overlapping of cells between clusters determined by alternative splicing and gene expression. **C.** The heatmap displays the enrichment score of ASP clusters across cell clusters determined by AS. **D.** The barplot displays the importance of splicing factors in the formation of C_13/17 ASP clusters. The color indicates the relationship between SF and ASP clusters. **E.** The length of junction that is differentially used in different cell types. Statistical analysis was performed using the Student's t-test. **F.** The boxplot displays the expression of stemness and mesoderm markers across primitive streak cell subsets determined by AS and expression. Statistical analysis was performed using the Student's t-test. **G.** The pathways enriched in the upregulated genes in the primitive streak cell subsets determined by AS and expression. **H.** The barplot displays the importance of splicing factors in the formation of C_1/18 ASP clusters. The color indicates the relationship between SF and ASP clusters. **I.** The alternative splicing

patterns of TNRC6B and the PSI distribution. **J.** The pathways enriched in the gene sets with different isoforms of TNRC6B. **K.** The illustration depicts the developmental trajectory and highlights the top-ranked key splicing factors based on their relative importance. * $p < 0.05$, ** $p < 0.01$, *** $p < 0.001$.

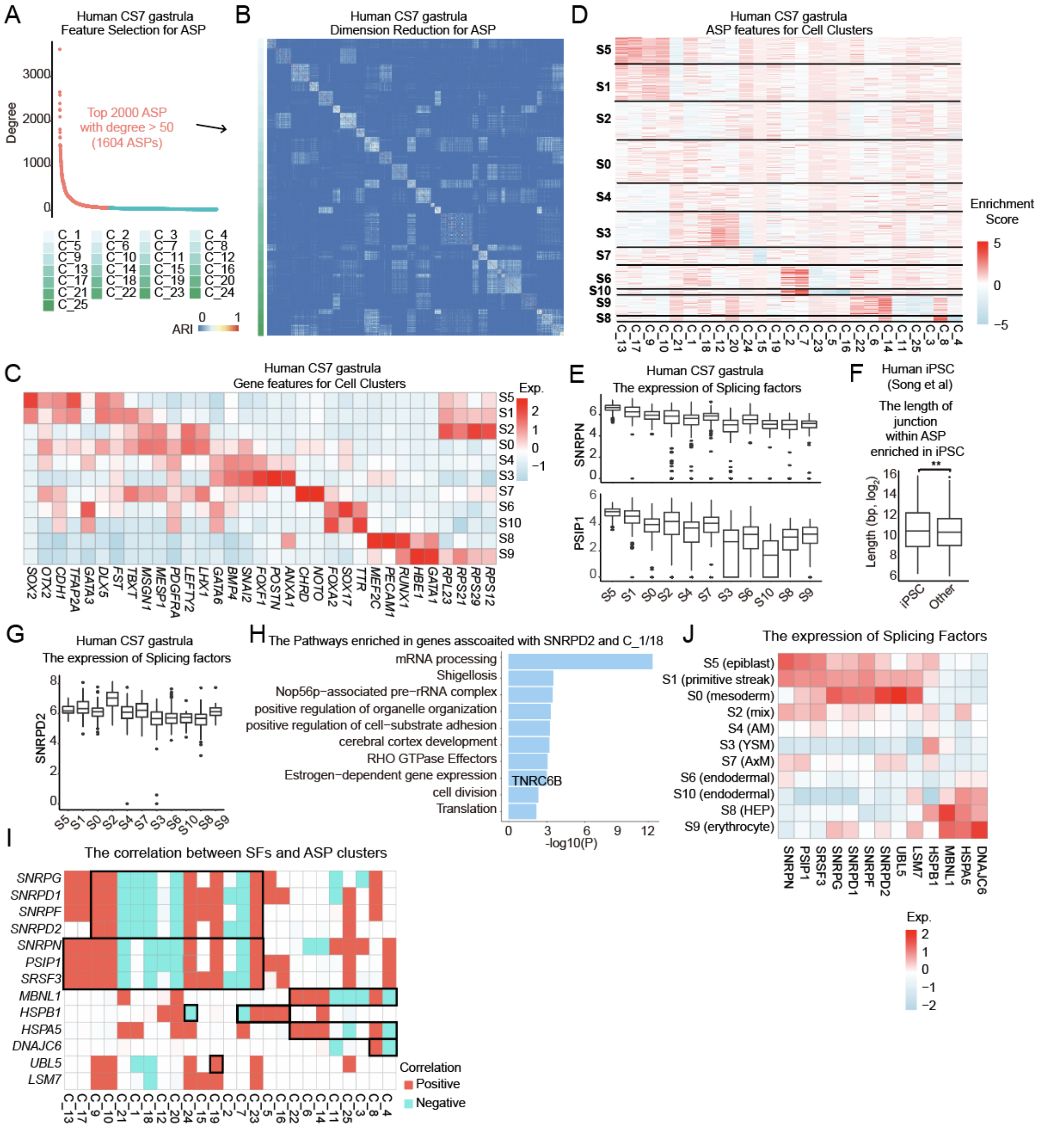

Figure S11. AEnet reveals intermediate cell status during gastrulation. A. The selection of key alternative splicing patterns. **B.** The heatmap shows the ASP clusters from dimension reduction of AEnet. **C.** The heatmap displays the expression of developmental markers across cell clusters determined by AS. **D.** The heatmap displays the enrichment score of ASP clusters across cell clusters determined by AS. **E.** The expression of SNRPN and PSIP1 across the cell clusters determined by AS. **F.** The length of junction that is differentially used in different cell types from Song et al. Statistical analysis was performed using the Student's t-test. **G.** The expression of SNRPD2 across the cell clusters determined by AS. **H.** The pathways enriched in the

genes within C_1 and C_18, which shows correlation with the expression of SNRPD2. **I-J.** The heatmap displays the relationship between ASP clusters and key splicing factors (I), as well as the expression of key splicing factors **(J)**. * $p < 0.05$, ** $p < 0.01$, *** $p < 0.001$.

Discussion

The discovery of cellular heterogeneity can be enhanced by multiple modalities derived from single-cell genomics technologies. For example, SHARE-seq provides insights into cell identity by jointly detecting gene expression and chromatin regulation [50], while CITE-seq simultaneously studies the transcriptome and protein expression, accounting for post-transcriptional and translational modifications to enable fine-grained detection of cell populations [51]. However, generating such data remains laboratory-intensive, involving complex, multi-step workflows that require careful optimization and the use of specialized reagents and equipment. In fact, alternative splicing events represent another inherent modality that is often present in common scRNA-seq data [52,53]. These events can be cost-efficiently detected using plate-based platforms like Fluidigm C1 and Smart-seq [54,55], or droplet-based platforms with higher sequencing depth [22,56,57].

The development of AEnet introduces a novel framework that integrates single-cell alternative splicing dynamics with gene expression profiles to construct regulatory networks reflective of cellular heterogeneity. By applying AEnet to three distinct single-cell datasets, we found that the interplay between AS and gene expression is dynamic in the process of generating cell populations. For tumor patient samples with varying immunotherapy responses and pan-cancer T cell analysis, the integrated clustering results reveal distinct cell states within the RNA-based subtypes. In contrast, during cell differentiation in embryonic gastrulation, the clustering results more closely resemble the major cell types. This suggests that AS events contribute differently across tissues and conditions in distinguishing cell clusters, with a particularly important role during the fine-grained clustering process. Therefore, previous methods that first rely on RNA-based clustering and then apply AS-based differential analysis as a separate downstream step are not optimal for capturing cellular heterogeneity using AS events.

A central advantage of the core advantages of AEnet lies in its ability to "modularize" complex gene expression data by grouping AS patterns, thereby minimizing noise from RNA expression profiling, which is often referred to as batch effects in scRNA-seq analysis. This modular approach also mitigates AS-based biases related to missing values, tumor heterogeneity, and patient-specific factors, while enabling the identification of novel cell subpopulations and intermediate states that may remain obscured using traditional methods.

It is important to note that AEnet is primarily designed for unsupervised discovery of splicing heterogeneity and regulatory mechanisms within a single biological context (e.g., a specific sample or condition), rather than for direct differential splicing comparisons across clusters or conditions (e.g., disease vs. healthy states). Its strengths lie in integrating alternative splicing and gene expression to characterize intra-condition splicing programs, identify regulatory modules, and predict affected pathways—capabilities enabled by its modular approach to minimize noise and capture context-enriched features. Notably, AEnet can also predict key regulatory components such as splicing factors (SFs) that drive observed splicing patterns, providing mechanistic insights into post-transcriptional regulation. For users seeking group-wise differential splicing analysis, AEnet's outputs (e.g., anchor ASPs and regulatory modules) can be integrated with dedicated tools like MARVEL or MAJIQ-SC, which specialize in statistical comparisons across predefined groups. A detailed functionality comparison of these methods is provided in Supplemental Table 1, aiding users in selecting tools aligned with their research objectives.

Looking forward, advances in sequencing technologies, particularly long-read platforms, will further enhance the resolution and accuracy of AS detection at the single cell level [14,58]. This advancement will significantly broaden the use of our method as a conventional analysis tool. However, even with well-designed sequencing, barcode errors in current single-cell long-read sequencing still significantly affect data quality, leading to the misassignment of AS events to unrelated cells [59]. We look forward to the rapid development of high-precision, high-throughput long-read sequencing technologies to advance research in the single-cell AS field and the development of our software in the near future.

Methods

Dataset preprocessing prior to AEnet for alternative splicing

Dataset preprocessing prior to AEnet involved a series of essential steps aimed at constructing the cell-junction count matrix and the annotation file for junctions, using the established DESJ-detection pipeline [19]. Initially, alignment software such as STAR was employed to obtain the coverage information of junctions in each cell [60]. To ensure the reliability of the data, the junctions with a minimum of Rm reads in Cellm cells were retained, with default values set as Cellm = 10 and Rm = 4. Additionally, all junctions were annotated to determine their primary gene sources. Specifically, we selectively retained junctions that were exclusively associated with a single gene to ensure accuracy of the alignment. Furthermore, a count matrix was generated, reflecting the read numbers of junctions in each cell. Subsequently, the count matrix was normalized through dividing the counts by the unique mapped reads number for each cell, resulting in the generation of the CPM (counts per million) matrix. The diligent execution of these preprocessing steps ensured the integrity and reliability of the dataset, allowing for robust analysis within the AEnet framework.

Detailed procedure for AEnet

The AEnet method consists of six major steps: ASP identification and quantification, AEN network and multiple samples integration, anchor ASP identification, ASP clusters identification, cell clustering, and regulatory mechanism prediction.The following sections describe each step in detail.

ASP identification and quantitative

Exon-exon junctions with the same starting point or endpoint were defined as alternative splicing patterns (ASPs). The percent spliced In (PSI) value of the ASPs in cells was measured by comparing the coverage of one junction to the two junctions derived from the ASP. Subsequently, we integrated all cells and all ASPs into a cell-ASP PSI matrix.

AEN Network and multiple samples integration

We integrated the alternative splicing event scoring matrix and gene expression matrix of all cells in each sample, to infer the potential relationship between ASPs and gene expression. For each sample, AEnet calculated the significance of the correlation between ASP PSI values and gene expression using Spearman's rank correlation. Significant ASP PSI - Gene Expression links ($p < 0.01$) were then used to construct an alternative splicing-gene expression association network (AEN) for each sample. The edge width of the links in the network was determined using the Spearman correlation coefficient, which indicates both the strength and the direction (positive or negative) of

the correlation. To ensure robustness, we extracted ASP-EXP links that exhibited consistent trends across multiple samples (defined as more than two samples). These links were then integrated into a multi-sample AEN network. In this pan-sample AEN network, the width of the edges corresponds to the number of samples supporting each ASP-EXP link, while the color indicates the direction of the correlation (positive or negative). This approach effectively mitigated batch effects and strengthened the reliability of the analysis.

Anchor ASPs/Genes identification

We calculated the degree strength of the ASP in the AEN network and selected the top ASPs based on their ranking. The degree of ASPs was determined by the number of links to the ASP in the AEN network. A higher degree indicates a stronger association between the ASP and gene expression dynamics in the dataset. The top 1,500 ASPs (by default) were selected for downstream analyses, provided that each ASP was associated with more than 15 positive and 15 negative links. To reduce data complexity and extract key information, we calculate the Jaccard metric of ASP-EXP links (where genes linked to the ASP are treated as sets) to represent their similarity for each pair of ASPs, thereby constructing a similarity matrix of anchor ASPs. Analogously, anchor genes were defined as those associated with ASPs from more than 30 distinct genes. For these anchor genes, a similar Jaccard similarity metric was calculated based on their associated ASPs, enabling the construction of a gene-level similarity matrix.

ASP/Gene clusters identification

Using hierarchical clustering methods, we divided the ASPs into ASP clusters according to their similarity, ensuring that ASPs in the same clusters were associated with similar gene expression sets. The number of clusters is set to 25 by default. Clusters are filtered by ensuring that at least 10% of ASP pairs within each cluster exhibit a similarity score higher than 0.1. Clusters with fewer than 10 ASPs are merged with the most similar clusters, provided that the similarity between clusters is above 0.1. The similarity between two ASP clusters is defined as the proportion of ASP pairs with a similarity score greater than 0.1, where one ASP in the pair belongs to one cluster and the other ASP belongs to the other cluster. An analogous clustering procedure was applied to anchor genes to identify gene clusters based on shared ASP associations.

Cell clustering analysis

To further understand the cell heterogeneity at the level of alternative splicing, we performed cell clustering analysis based on alternative splicing and gene expression. Specifically, we first calculated the enrichment scores of each ASP cluster and each

gene cluster for individual cells, resulting in a cell-by-cluster enrichment score matrix. The enrichment score of an ASP cluster in a given cell was defined as the average PSI (Percent Spliced-In) value of all ASPs within that cluster. Similarly, the enrichment score of a gene cluster in a given cell was defined as the average expression level of all genes in that cluster. Next, the cell-ASP and Gene cluster enrichment score matrix was normalized using the scale function. Finally, the normalized matrix was used as input for the FindNeighbors and FindClusters functions of Seurat to detect cell clusters [61,62]. Notably, cell clustering can be performed based on either cell–ASP cluster enrichment score matrix or cell–gene cluster matrix alone. Finally, cell subpopulations were delineated using a dual-modality approach that integrates alternative splicing patterns (ASPs) with gene expression data.

Regulatory mechanism inference

To identify key splicing factors, we integrated the AEN network with a predefined list of splicing factors and a specific set of ASP events [63]. This allowed us to construct a sub-network of ASP-splicing factors, facilitating the exploration of the regulatory relationships between alternative splicing events and splicing factors. The higher the degree strength of the splicing factor, the more it indicated a close relationship with the occurrence of these ASP events. The regulatory direction of splicing factors on the given set of ASPs was determined based on the proportion of positive or negative correlations. Specifically, if 75% or more of the splicing factor–ASP pairs were positive (or negative), the splicing factor was classified as positively (or negatively) regulating the ASP sets.

We also identified key pathways associated with different ASP patterns. For each ASP event, gene sets that were positively and negatively correlated with the event were identified based on the AEN network. Functional enrichment analysis was then performed on both the ASP's gene and the positively (or negatively) correlated gene sets. The pathway to which the ASP gene belongs is considered the most likely pathway affected by the ASP change.

Performance evaluation of AEnet

To evaluate AEnet's performance in inferring ASPs relative to MARVEL—the currently most comprehensive method—we applied a demo dataset of MARVEL, comprising induced pluripotent stem cells (iPSCs) and iPSC-derived endoderm cells [30]. ASP patterns were identified using the asp function of AEnet, while MARVEL was run with default parameters for direct comparison. To systematically evaluate the impact of count thresholds on ASP detection, we tested a range of minimum read count cutoffs: 0, 3, 5, 7, and 9. In parallel, to assess potential biases introduced by filtering against rare but biologically relevant splicing events, we stratified all ASPs into five categories based

on the number of supporting cells: Invalid: ≤20 cells (excluded); Type 1: >20–30 cells; Type 2: >30–40 cells; Type 3: >40–50 cells; Type 4: >50 cells. Subsequent analyses evaluated the influence of each ASP type on link robustness and biological relevance.

To evaluate AEnet's ability to identify ASP clusters, a simulated ASP similarity matrix was generated, with ASPs represented along both rows and columns, and similarity values (measured by the Jaccard index) as matrix elements. To assess robustness under varying noise conditions, different types of noise were introduced: uniform noise via the noiseInjector.unif function from the GROAN package, Gaussian noise using the rtruncnorm function, and Poisson noise using the poisson_discrete function from the truncnorm package. The number of ground-truth ASP clusters was set to three. AEnet's clustering performance was quantified by a consistency score, defined as the Jaccard index between the predicted ASP clusters and the simulated ground-truth clusters.

Benchmarking AEnet against other methods

We next focused on evaluating the clustering performance of AEnet using published datasets with established cell type annotations as ground truth, employing Adjusted Rand Index (ARI) and Normalized Mutual Information (NMI) as evaluation metrics. Three independent datasets—iPSC[18], HCC[28], and T cell datasets[29]—were used for benchmarking, with detailed information provided in **Supplementary Table S3**. To assess the contribution of different data modalities, we conducted ablation analyses by providing AEnet with either the expression matrix alone, the ASP PSI matrix alone, or both. These inputs were used to construct the full ASP-EXP network, as well as ASP-only and EXP-only networks, followed by cell clustering based on each configuration.

To compare the performance of AEnet with existing splicing-based clustering methods, we evaluated SCASL and scQuint across three benchmark datasets. The cell–junction count matrix was used as input for both SCASL and scQuint, with all methods run using their respective default parameters. Notably, scQuint was used solely for low-dimensional embedding, whereas SCASL provided both embeddings and clustering outputs. To further assess AEnet's robustness to batch effects, we analyzed data from three patients in the lung cancer dataset and four patients from the CRC and RHCC cohorts. We then examined the distribution of cells in the low-dimensional embedding space to evaluate the extent of batch mixing.

To further demonstrate the versatility of AEnet, we applied it to a widely used 10x Genomics peripheral blood mononuclear cell (PBMC) dataset [34]. Due to the limited sequencing depth of 10X data, AEnet was unable to detect a sufficient number of alternative splicing (AS) events for downstream analysis. Therefore, we focused

instead on alternative polyadenylation (APA) events, which also reflect isoform-level regulatory dynamics. APA usage was quantified using the scAPAtrap [64] and movAPA [65] package, and the resulting cell-level Relative Usage of Distal polyadenylation sites (RUD) values, together with gene expression data, were used as input to AEnet with default parameters to evaluate its performance on 10X datasets.

The lung cancer cells data processing

We downloaded the scRNA-seq raw reads of the human lung cancer dataset from the NCBI database under accession code PRJNA591860[33]. This dataset contained 1,286 normal and cancer cells from six patients before initiating systemic targeted therapy (TKI naive [TN]), at the residual disease (RD) state, which includes samples taken at any response groups during treatment with targeted therapy while the tumor was regressing or stable by clinical imaging (RD), and upon subsequent progressive disease as determined by clinical imaging, at which point the tumors showed acquired drug resistance (progression [PD]). The human genome (version GRCh38) was used as the reference genome for alignment with STAR (v2.5.3)[60]. We used an existing pipeline to create the junction count matrix. We first merged all the output of the SJ.out.tab files from the STAR aligner. Next, we conducted the dataset pre-processing prior to the AEnet step, described as before. Finally, we get the cell-junction CPM matrix, and the junction annotation files for the dataset.

Then, we used the outcome from the above step as the input to the AEnet pipeline. Firstly, the CPM matrix was input to the asp function of AEnet with default parameters, and the ASPs for each gene is identified. Secondly, the correlation network between ASPs and Gene is constructed using the asp function with default parameters based on the expression matrix and the junction CPM matrix. Next, we further refined the AEN network by retaining only the connection pairs with at least 4 samples supported, using the merge_cor function with default parameters. Additionally, the ASPs with positive or negative links all more than 20 are retained and 70 key ASPs were identified. These key ASPs were then clustered with hierarchy clustering, leading to the identification of 6 ASP clusters based on their similarity, using the junction_clustering function with default parameters. Subsequently, we calculated the enrichment score of each ASP cluster for the cells and performed cluster analysis using the enrichment score matrix of the cell-ASP clusters, categorizing the cells into 3 groups using asp_score and cell_clus function with resolution as 0.2. To further identify the key splicing factors involved in the formation of the C_4 ASP clusters, we used the key_sf function to detect the key SF. Additionally, we also group cells into different populations based on gene expression. The gene count matrix was normalized using log1p normalization. Next, the top 3000 highly variable genes were selected to perform principal component analysis. Subsequently, 20 dimensions of principal components were used to perform Louvain clustering and Uniform Manifold Approximation and Projection (UMAP)-based visualization.

The SCASL package was applied to the junction count matrix of tumor cells to perform cell clustering, using default parameters for filtering, normalization, imputation, and clustering [31]. The Seurat [61,62] package was applied to the expression matrix of tumor cells to perform normalization, dimensionality reduction and clustering: (1) the data was normalized using the "LogNormalize" function; (2) the top 2000 highly variable genes were detected with the "FindVariableFeatures" function and selected, the batch effects across different samples were corrected by the "FindIntegrationAnchors" and "IntegrateData" functions; (3) a KNN-based graph in the 20 PCA space was constructed and refined by cell-cell weights using the "FindNeighbors" function; (4) representative results from graph-based clustering were obtained using the "FindClusters" function with a resolution of 0.3; (5) the top 20 PCAs were used to perform UMAP for visualization of the cells.

The pan-cancer T cells data processing

We downloaded the scRNA-seq raw reads of human T cells in the fasta format from the EGA database (EGAS00001002072 for HCC, EGAS00001002791 for CRC), NCBI database (PRJNA591860 for LUAD), and CNSA database (CNP0000650 for RHCC). The corresponding gene expression matrix was downloaded from the GEO database (GSE98638 for HCC, GSE108989 for CRC) and CNSA database (CNP0000650 for RHCC). The human genome (version GRCH38) was used as the reference genome for alignment with STAR (v2.5.3). We used an existing pipeline to create the junction count matrix. We first merged all the output of the SJ.out.tab files from the STAR aligner. Next, we conducted the dataset pre-processing prior to the AEnet step, described as before. Finally, we get the cell-junction CPM matrix, and the junction annotation files for each dataset.

Then, we used the outcome from the above step as the input to the AEnet pipeline. Firstly, the CPM matrix was input to the asp function of AEnet with default parameters, and the ASPs for each gene were identified for each dataset. Secondly, the correlation network between ASPs and Gene is constructed using the asp function with default parameters based on the expression matrix and the junction CPM matrix for each dataset. Next, we further refined the AEN network by retaining only the connection pairs with at least 2 samples supported for each dataset, using the merge_cor function with default parameters. Additionally, The ASP-EXP links supported by at least 2 datasets were retained for the downstream analysis. As a result, 1,223 key ASPs were identified based on their ranking and absolute number of degrees (top 2,000 with a degree number greater than 10). These key ASPs were then clustered, leading to the identification of 14 ASP clusters based on the similarity of their associated phenotypes through junction_clustering function with default parameters. Subsequently, we calculated the enrichment score of each ASP cluster for the cells of the four datasets and performed cluster analysis using the enrichment score matrix of the cell-ASP clusters, categorizing the cells into 10 groups using cell_clus function with resolution

as 0.9. To further identify the key splicing factors involved in the formation of the 14 ASP clusters, we used the key_sf function to detect the key SF for each ASP cluster. AEnet was also used to detect key gene sets affected by ASPs from the same gene. These gene sets were then used to identify enriched pathways associated with different alternative splicing patterns of the same gene, such as CD3D and FYB1. The SCASL package was applied to the junction count matrix of T cells in multiple datasets to perform cell clustering, using default parameters for filtering, normalization, imputation, and clustering [31].

The gastrulating human embryo cells data processing

We downloaded the scRNA-seq raw reads of the human embryo dataset from ArrayExpress under accession code E-MTAB-9388. This dataset contained 1195 cells of the human embryo at embryonic day 16 assigned into 10 clusters. The human genome (version GRCH38) was used as the reference genome for alignment with STAR (v2.5.3). We used an existing pipeline to create the junction count matrix. We first merged all the output of the SJ.out.tab files from the STAR aligner. Next, we conducted the dataset pre-processing prior to the AEnet step, described as before. Finally, we get the cell-junction CPM matrix, and the junction annotation files for the dataset.

Then, we used the result from the above step as the input to the AEnet pipeline. Firstly, the CPM matrix was input to the asp function of AEnet with default parameters, and the ASPs for each gene were identified. Secondly, the correlation network between ASPs and Gene is constructed using the asp function with default parameters based on the expression matrix and the junction CPM matrix. Next, we further refined the AEN network by retaining only the connection pairs with p-values less than 1e-4. As a result, 1,604 key ASPs were identified based on their ranking and absolute number of degrees (top 2,000 with a degree number greater than 50). These key ASPs were then clustered, leading to the identification of 25 ASP clusters based on the similarity of their associated phenotypes. Subsequently, we calculated the enrichment scores of each ASP class within the cells and performed cluster analysis using the enrichment score matrix of the cell-ASP clusters, categorizing the cells into 11 groups using the cell_clus function with resolution as 0.1. To further identify the key splicing factors involved in the formation of the 25 ASP clusters, we used the key_sf function to detect the key SF for each ASP class. AEnet was also used to detect key gene sets affected by ASPs from the same gene. These gene sets were then used to identify enriched pathways associated with different alternative splicing patterns of the same gene, such as TNRC6B.

Pathway enrichment analysis

Metascape (RRID:SCR_016620) was used to characterize the biological functions of the DEGs of cells in different statuses[66]. The differentially expressed genes between

different cell types were uploaded into the Metascape for the pathway analysis with default setting.

Survival Analysis

GEPIA2 (RRID: SCR_026154) was used to analyze the survival status of HNRNPLL[67]. HNRNPLL was uploaded into the GEPIA2 and the reference datasets were the whole TCGA datasets.

Acknowledgements

This study was supported by Science and Technology Innovation Key R&D Program of Chongqing (CSTB2024TIAD-STX0003), National Natural Science Foundation of China (32300514), and Shenzhen Science and Technology Program (LCYX20220620105200001 and JCYJ20240813150001003).

Data availability

The gastrulating human embryo dataset is available in the ArrayExpress database under accession number E-MTAB-9388. scRNA-seq raw reads of human T cells in fastq format from the EGA database (EGAS00001002072 for HCC, EGAS00001002791 for CRC), NCBI database (PRJNA591860 for LUAD), and CNSA database (CNP0000650 for RHCC). The corresponding gene expression matrix was downloaded from the GEO database (GSE98638 for HCC, GSE108989 for CRC) and CNSA database (CNP0000650 for RHCC). The additional data files are hosted in Zenodo [69].

Availability of Source Code and Requirements

Project name: AEnet

Project homepage: https://github.com/liushang17/AEN

Operating system(s): Platform independent

Programming language: R

License: GPL-3.0 license

RRID:SCR_027285

A version of record snapshot of the GitHub repository has been archived in the Software Heritage [70].

Author contributions

Sh.L., X.C, Y.B., L.W. and S.L. conceived the study, designed and performed research, contributed new analytical tools, analyzed data and wrote the manuscript. Sh.L. also developed the software, performed experiments and developed the metrics. X.C, X.H., Y.B., Y.W., W.H., P.Q., R.L., W.P., and X.Z. discussed the results and contributed to the writing. X.C, helped procure and interpret the datasets. L.W., Y.B., X. H, X.C, and S.L. supervised research and contributed to the writing. L.W. supervised the research and the entire project.

Competing interests

The authors declare no competing interests.

Declaration of generative AI and AI-assisted technologies in the writing process

During the preparation of this work, the authors used ChatGPT 4.0 (GPT-4) in order to facilitate the process of proofreading the contents of the draft[68]. After using this tool/service, the authors reviewed and edited the content as needed and took full responsibility for the content of the publication.

Reference

1. Liu Y, Gonzàlez-Porta M, Santos S, Brazma A, Marioni JC, Aebersold R, et al.. Impact of alternative splicing on the human proteome. *Cell Rep*. 20:1229–412017;

2. Öther-Gee Pohl S, Myant KB. Alternative RNA splicing in tumour heterogeneity, plasticity and therapy. *Dis Model Mech*. The Company of Biologists; 2022; doi: 10.1242/dmm.049233.

3. Wright CJ, Smith CWJ, Jiggins CD. Alternative splicing as a source of phenotypic diversity. *Nat Rev Genet*. Springer Science and Business Media LLC; 23:697–7102022;

4. Suzuki H, Aoki Y, Kameyama T, Saito T, Masuda S, Tanihata J, et al.. Endogenous multiple Exon skipping and back-splicing at the DMD mutation hotspot. *Int J Mol Sci*. MDPI AG; 17:17222016;

5. Raj B, Blencowe BJ. Alternative splicing in the mammalian nervous system: Recent insights into mechanisms and functional roles. *Neuron*. Elsevier BV; 87:14–272015;

6. Bhadra M, Howell P, Dutta S, Heintz C, Mair WB. Alternative splicing in aging and longevity. *Hum Genet*. Springer Science and Business Media LLC; 139:357–692020;

7. Martinez NM, Lynch KW. Control of alternative splicing in immune responses: many regulators, many predictions, much still to learn. *Immunol Rev*. Wiley; 253:216–362013;

8. Martín E, Vivori C, Rogalska M, Herrero-Vicente J, Valcárcel J. Alternative splicing regulation of cell-cycle genes by SPF45/SR140/CHERP complex controls cell proliferation. *RNA*. Cold Spring Harbor Laboratory; 27:1557–762021;

9. Braeutigam C, Rago L, Rolke A, Waldmeier L, Christofori G, Winter J. The RNA-binding protein Rbfox2: an essential regulator of EMT-driven alternative splicing and a mediator of cellular invasion. *Oncogene*. Springer Science and Business Media LLC; 33:1082–922014;

10. Jbara A, Lin K-T, Stossel C, Siegfried Z, Shqerat H, Amar-Schwartz A, et al.. RBFOX2 modulates a metastatic signature of alternative splicing in pancreatic cancer. *Nature*. Springer Science and Business Media LLC; 617:147–532023;

11. Olivieri JE, Dehghannasiri R, Salzman J. The SpliZ generalizes “percent spliced in” to reveal regulated splicing at single-cell resolution. *Nat Methods*. Springer Science and Business Media LLC; 19:307–102022;

12. Li Z, Zhang B, Chan JJ, Tabatabaeian H, Tong QY, Chew XH, et al.. An isoform-resolution transcriptomic atlas of colorectal cancer from long-read single-cell sequencing. *Cell Genom*. Elsevier BV; 4:1006412024;

13. Huang Y, Guo J, Han X, Zhao Y, Li X, Xing P, et al.. Splicing diversity enhances the molecular classification of pituitary neuroendocrine tumors. *Nat Commun*. Springer Science and Business Media LLC; 16:15522025;

14. Joglekar A, Hu W, Zhang B, Narykov O, Diekhans M, Marrocco J, et al.. Single-cell long-read sequencing-based mapping reveals specialized splicing patterns in developing and adult mouse and human brain. *Nat Neurosci*. Springer Science and Business Media LLC; 27:1051–632024;

15. Lukacsovich D, Winterer J, Que L, Luo W, Lukacsovich T, Földy C. Single-cell RNA-seq reveals developmental origins and ontogenetic stability of neurexin alternative splicing profiles. *Cell Rep*. Elsevier BV; 27:3752–9.e42019;

16. Shalek AK, Satija R, Adiconis X, Gertner RS, Gaublomme JT, Raychowdhury R, et al.. Single-cell transcriptomics reveals bimodality in expression and splicing in immune cells. *Nature*. Springer Science and Business Media LLC; 498:236–402013;

17. Huang Y, Sanguinetti G. BRIE: transcriptome-wide splicing quantification in single cells. *Genome Biol*. Springer Science and Business Media LLC; 2017; doi: 10.1186/s13059-017-1248-5.

18. Song Y, Botvinnik OB, Lovci MT, Kakaradov B, Liu P, Xu JL, et al.. Single-cell alternative splicing analysis with expedition reveals splicing dynamics during neuron differentiation. *Mol Cell*. Elsevier BV; 67:148–61.e52017;

19. Liu S, Zhou B, Wu L, Sun Y, Chen J, Liu S. Single-cell differential splicing analysis reveals high heterogeneity of liver tumor-infiltrating T cells. *Sci Rep*. Springer Science and Business Media LLC; 11:53252021;

20. Wen WX, Mead AJ, Thongjuea S. MARVEL: an integrated alternative splicing

analysis platform for single-cell RNA sequencing data. *Nucleic Acids Res*. Oxford University Press (OUP); 51:e292023;

21. Zhang Q, Ai Y, Abdel-Wahab O. Molecular impact of mutations in RNA splicing factors in cancer. *Mol Cell*. Elsevier BV; 84:3667–802024;

22. Capitanchik C, Wilkins OG, Wagner N, Gagneur J, Ule J. From computational models of the splicing code to regulatory mechanisms and therapeutic implications. *Nat Rev Genet*. 2024; doi: 10.1038/s41576-024-00774-2.

23. Risso D, Perraudeau F, Gribkova S, Dudoit S, Vert J-P. A general and flexible method for signal extraction from single-cell RNA-seq data. *Nat Commun*. 9:2842018;

24. Tran HTN, Ang KS, Chevrier M, Zhang X, Lee NYS, Goh M, et al.. A benchmark of batch-effect correction methods for single-cell RNA sequencing data. *Genome Biol*. Springer Science and Business Media LLC; 21:122020;

25. Wan Y, Larson DR. Splicing heterogeneity: separating signal from noise. *Genome Biol*. 19:862018;

26. Dvinge H, Kim E, Abdel-Wahab O, Bradley RK. RNA splicing factors as oncoproteins and tumour suppressors. *Nat Rev Cancer*. Springer Science and Business Media LLC; 16:413–302016;

27. Bradley RK, Anczuków O. RNA splicing dysregulation and the hallmarks of cancer. *Nat Rev Cancer*. 23:135–552023;

28. Sun Y, Wu L, Zhong Y, Zhou K, Hou Y, Wang Z, et al.. Single-cell landscape of the ecosystem in early-relapse hepatocellular carcinoma. *Cell*. Elsevier BV; 184:404–21.e162021;

29. Zhang L, Yu X, Zheng L, Zhang Y, Li Y, Fang Q, et al.. Lineage tracking reveals dynamic relationships of T cells in colorectal cancer. *Nature*. Springer Science and Business Media LLC; 564:268–722018;

30. Linker SM, Urban L, Clark SJ, Chhatriwala M, Amatya S, McCarthy DJ, et al.. Combined single-cell profiling of expression and DNA methylation reveals splicing regulation and heterogeneity. *Genome Biol*. Springer Science and Business Media LLC; 20:302019;

31. Xiang X, He Y, Zhang Z, Yang X. Interrogations of single-cell RNA splicing landscapes with SCASL define new cell identities with physiological relevance. *Nat Commun*. Springer Science and Business Media LLC; 2024; doi: 10.1038/s41467-024-46480-9.

32. Benegas G, Fischer J, Song YS. Robust and annotation-free analysis of alternative splicing across diverse cell types in mice. *Elife*. eLife Sciences Publications, Ltd; 2022; doi: 10.7554/eLife.73520.

33. Maynard A, McCoach CE, Rotow JK, Harris L, Haderk F, Kerr DL, et al.. Therapy-induced evolution of human lung cancer revealed by single-cell RNA sequencing. *Cell*. Elsevier BV; 182:1232–51.e222020;

34. Xue R, Zhang Q, Cao Q, Kong R, Xiang X, Liu H, et al.. Liver tumour immune

microenvironment subtypes and neutrophil heterogeneity. *Nature*. Springer Science and Business Media LLC; 612:141–72022;

35. Travaglini KJ, Nabhan AN, Penland L, Sinha R, Gillich A, Sit RV, et al.. A molecular cell atlas of the human lung from single-cell RNA sequencing. *Nature*. Springer Science and Business Media LLC; 587:619–252020;

36. Sikkema L, Ramírez-Suástegui C, Strobl DC, Gillett TE, Zappia L, Madissoon E, et al.. An integrated cell atlas of the lung in health and disease. *Nat Med*. 29:1563–772023;

37. Yang J, Antin P, Berx G, Blanpain C, Brabletz T, Bronner M, et al.. Guidelines and definitions for research on epithelial-mesenchymal transition. *Nat Rev Mol Cell Biol*. Springer Science and Business Media LLC; 21:341–522020;

38. Beyer EC, Berthoud VM. Gap junction gene and protein families: Connexins, innexins, and pannexins. *Biochim Biophys Acta Biomembr*. 1860:5–82018;

39. Narayanaswamy PB, Baral TK, Haller H, Dumler I, Acharya K, Kiyan Y. Transcriptomic pathway analysis of urokinase receptor silenced breast cancer cells: a microarray study. *Oncotarget*. 8:101572–902017;

40. Piqué L, Martinez de Paz A, Piñeyro D, Martínez-Cardús A, Castro de Moura M, Llinàs-Arias P, et al.. Epigenetic inactivation of the splicing RNA-binding protein CELF2 in human breast cancer. *Oncogene*. Springer Science and Business Media LLC; 38:7106–122019;

41. Lai S, Wang Y, Li T, Dong Y, Lin Y, Wang L, et al.. N6-methyladenosine-mediated CELF2 regulates CD44 alternative splicing affecting tumorigenesis via ERAD pathway in pancreatic cancer. *Cell Biosci*. 12:1252022;

42. Qin P, Chen H, Wang Y, Huang L, Huang K, Xiao G, et al.. Cancer-associated fibroblasts undergoing neoadjuvant chemotherapy suppress rectal cancer revealed by single-cell and spatial transcriptomics. *Cell Rep Med*. Elsevier BV; 4:1012312023;

43. Fukuda Y, Bustos MA, Cho S-N, Roszik J, Ryu S, Lopez VM, et al.. Interplay between soluble CD74 and macrophage-migration inhibitory factor drives tumor growth and influences patient survival in melanoma. *Cell Death Dis*. Springer Science and Business Media LLC; 2022; doi: 10.1038/s41419-022-04552-y.

44. Leng L, Metz CN, Fang Y, Xu J, Donnelly S, Baugh J, et al.. MIF signal transduction initiated by binding to CD74. *J Exp Med*. Rockefeller University Press; 197:1467–762003;

45. Zheng C, Zheng L, Yoo J-K, Guo H, Zhang Y, Guo X, et al.. Landscape of infiltrating T cells in liver cancer revealed by single-cell sequencing. *Cell*. 169:1342–56.e162017;

46. Zheng L, Qin S, Si W, Wang A, Xing B, Gao R, et al.. Pan-cancer single-cell landscape of tumor-infiltrating T cells. *Science*. American Association for the Advancement of Science (AAAS); 374:abe64742021;

47. Wilkinson ME, Charenton C, Nagai K. RNA splicing by the spliceosome. *Annu Rev Biochem*. Annual Reviews; 89:359–882020;

48. Tyser RCV, Mahammadov E, Nakanoh S, Vallier L, Scialdone A, Srinivas S. Single-cell transcriptomic characterization of a gastrulating human embryo. *Nature*. Springer Science and Business Media LLC; 600:285–92021;

49. Zhai J, Xiao Z, Wang Y, Wang H. Human embryonic development: from peri-implantation to gastrulation. *Trends Cell Biol*. Elsevier BV; 32:18–292022;

50. Ma S, Zhang B, LaFave LM, Earl AS, Chiang Z, Hu Y, et al.. Chromatin potential identified by shared single-cell profiling of RNA and chromatin. *Cell*. Elsevier BV; 183:1103–16.e202020;

51. Stoeckius M, Hafemeister C, Stephenson W, Houck-Loomis B, Chattopadhyay PK, Swerdlow H, et al.. Simultaneous epitope and transcriptome measurement in single cells. *Nat Methods*. 14:865–82017;

52. Kahles A, Lehmann K-V, Toussaint NC, Hüser M, Stark SG, Sachsenberg T, et al.. Comprehensive analysis of alternative splicing across tumors from 8,705 patients. *Cancer Cell*. 34:211–24.e62018;

53. Cortés-López M, Chamely P, Hawkins AG, Stanley RF, Swett AD, Ganesan S, et al.. Single-cell multi-omics defines the cell-type-specific impact of splicing aberrations in human hematopoietic clonal outgrowths. *Cell Stem Cell*. Elsevier BV; 30:1262–81.e82023;

54. Picelli S, Björklund ÅK, Faridani OR, Sagasser S, Winberg G, Sandberg R. Smart-seq2 for sensitive full-length transcriptome profiling in single cells. *Nat Methods*. Springer Science and Business Media LLC; 10:1096–82013;

55. Picelli S, Faridani OR, Björklund AK, Winberg G, Sagasser S, Sandberg R. Full-length RNA-seq from single cells using Smart-seq2. *Nat Protoc*. Springer Science and Business Media LLC; 9:171–812014;

56. Liao Y, Liu Z, Zhang Y, Lu P, Wen L, Tang F. High-throughput and high-sensitivity full-length single-cell RNA-seq analysis on third-generation sequencing platform. *Cell Discov*. Springer Science and Business Media LLC; 9:52023;

57. Shiau C-K, Lu L, Kieser R, Fukumura K, Pan T, Lin H-Y, et al.. High throughput single cell long-read sequencing analyses of same-cell genotypes and phenotypes in human tumors. *Nat Commun*. 14:41242023;

58. Kumari P, Kaur M, Dindhoria K, Ashford B, Amarasinghe SL, Thind AS. Advances in long-read single-cell transcriptomics. *Hum Genet*. Springer Science and Business Media LLC; 143:1005–202024;

59. Weile J, Ferra G, Boyle G, Pendyala S, Amorosi C, Yeh C-L, et al.. Pacybara: accurate long-read sequencing for barcoded mutagenized allelic libraries. *Bioinformatics*. 2024; doi: 10.1093/bioinformatics/btae182.

60. Dobin A, Davis CA, Schlesinger F, Drenkow J, Zaleski C, Jha S, et al.. STAR: ultrafast universal RNA-seq aligner. *Bioinformatics*. Oxford University Press (OUP); 29:15–212013;

61. Hao Y, Hao S, Andersen-Nissen E, Mauck WM 3rd, Zheng S, Butler A, et al.. Integrated analysis of multimodal single-cell data. *Cell*. Elsevier BV; 184:3573–87.e292021;

62. Stuart T, Butler A, Hoffman P, Hafemeister C, Papalexi E, Mauck WM 3rd, et al.. Comprehensive integration of single-cell data. *Cell*. Elsevier BV; 177:1888–902.e212019;

63. Seiler M, Peng S, Agrawal AA, Palacino J, Teng T, Zhu P, et al.. Somatic mutational landscape of splicing factor genes and their functional consequences across 33 cancer types. *Cell Rep*. 23:282–96.e42018;

64. Wu X, Liu T, Ye C, Ye W, Ji G. scAPAtrap: identification and quantification of alternative polyadenylation sites from single-cell RNA-seq data. *Brief Bioinform*. Oxford University Press (OUP); 2021; doi: 10.1093/bib/bbaa273.

65. Ye W, Liu T, Fu H, Ye C, Ji G, Wu X. movAPA: modeling and visualization of dynamics of alternative polyadenylation across biological samples. *Bioinformatics*. Oxford University Press (OUP); 37:2470–22021;

66. Zhou Y, Zhou B, Pache L, Chang M, Khodabakhshi AH, Tanaseichuk O, et al.. Metascape provides a biologist-oriented resource for the analysis of systems-level datasets. *Nat Commun*. Springer Science and Business Media LLC; 10:15232019;

67. Tang Z, Kang B, Li C, Chen T, Zhang Z. GEPIA2: an enhanced web server for large-scale expression profiling and interactive analysis. *Nucleic Acids Res*. Oxford University Press (OUP); 47:W556–602019;

68. Bai Y, Kosonocky CW, Wang JZ. How our authors are using AI tools in manuscript writing. *Patterns (N Y)*. Elsevier BV; 5:1010752024;

69. Liu S. AEnet. [Data set]. Zenodo. 2025. https://doi.org/10.5281/zenodo.16849845

70. Liu S. AEnet: a practical tool to construct the splicing associated phenotype atlas at single cell level. [Computer software]. Software Heritage. 2025. https://archive.softwareheritage.org/swh:1:snp:0ba5c7b2cec2e259301a1b28ed02642febc5bf95

Click here to access/download
Supplementary Material
Supplementary Tables.xlsx

Personal Cover Click here to access/download;Personal Cover;Cover letter.pdf

Dear Hongfang,

On behalf of all co-authors, we are extremely grateful for the journal's acceptance of our manuscript entitled "AEnet: a practical tool to construct the splicing associated phenotype atlas at single-cell level" for publication in GigaScience. This recognition of our work is deeply encouraging, and we sincerely appreciate the editorial team's guidance throughout the review process.

We also wish to extend our heartfelt thanks to the reviewers for their invaluable and meticulous comments. Their insights have been instrumental in refining our work, and we truly value the time and expertise they have dedicated to enhancing the quality of our manuscript. We have carefully addressed their remaining suggestions, with key revisions including:

- Added R Markdown tutorials for iPSC and T cell datasets (with links provided) to aid reproducibility and user guidance.
- Revised Figs. 2 and S2–S4 for better clarity, with improved layouts, step-wise labels, and consistent visuals.
- Conducted a thorough editorial check to refine language, enhance flow, and ensure consistency across the manuscript.

We believe these revisions have further strengthened the manuscript, making it more robust, accessible, and valuable to the scientific community. AEnet's integration of alternative splicing and gene expression data offers a unique tool for exploring cellular heterogeneity at the single-cell level, and we are excited to share it through GigaScience.

Thank you once again for your support and the opportunity to publish our work. Please feel free to contact us if any additional adjustments are needed.

Yours Sincerely,

Liang Wu, PhD

On behalf of all authors

BGI Research, Shenzhen 518083, China.

Email: wuliang@genomics.cn

Click here to access/download;Personal Cover;Response letter for AEnet.pdf

Point-by-point response to the referees' comments:

Reviewer #1: The authors have addressed my concerns.

Reviewer #2: The authors have thoroughly addressed my comments. I am pleased to see that the benchmarking analysis is substantially improved and the utility of the software has been demonstrated in wider context, i.e., additional datasets.

My only remaining comment relates to Comment no. 8: While the authors provided the source code for the R package, and the dataset, I would encourage the authors to provide the R Markdown tutorial. This documentation should demonstrate the pre-processing steps and analysis of at least one example dataset and correspondingly reproduce selected figures in the manuscript. This is not only for the purpose of transparency and reproducibility, but this will in turn encourage prospective users, like myself, to apply this software.

Response: We sincerely thank the reviewer for the valuable suggestion. To facilitate reproducibility and help users get started, we also provide demonstration datasets corresponding to two biological systems using the R Markdown tutorial:

- iPSC dataset: https://liushang17.github.io/ipsc.html
- T cell dataset: https://liushang17.github.io/tcells.html

The corresponding R Markdown source code is also available for both datasets (https://github.com/liushang17/AEnet). These tutorials reproduce key steps and selected figures from the manuscript, offering prospective users a practical guide for applying the AEnet package to their own data.

Reviewer #3: I appreciate the authors' careful attention to the reviewers' comments. The updated manuscript has addressed many concerns raised in the reviews, especially

* Clear separation of model description and benchmarking improves clarity
* Benchmarking across multiple additional datasets and platforms enhance the generalizability
* Batch effect handling is better explained
* Ablation study (A-net vs. E-net) supports the value of joint modeling
* Noise modeling and ASP thresholding are better tested

Overall, this work has been strengthened. A few points should be fixed

* Some of the workflow figures (e.g., Fig. 2, S2-S4) are visually confusing and could benefit from clearer visual layout or annotations.

Response: We sincerely thank the reviewer for this constructive suggestion. We have revised the schematics and updated the figure descriptions and legends in Fig. 2 and S2–S4, by adjusting the layout for better readability, adding step-wise labels, and ensuring consistent visual elements across all panels.

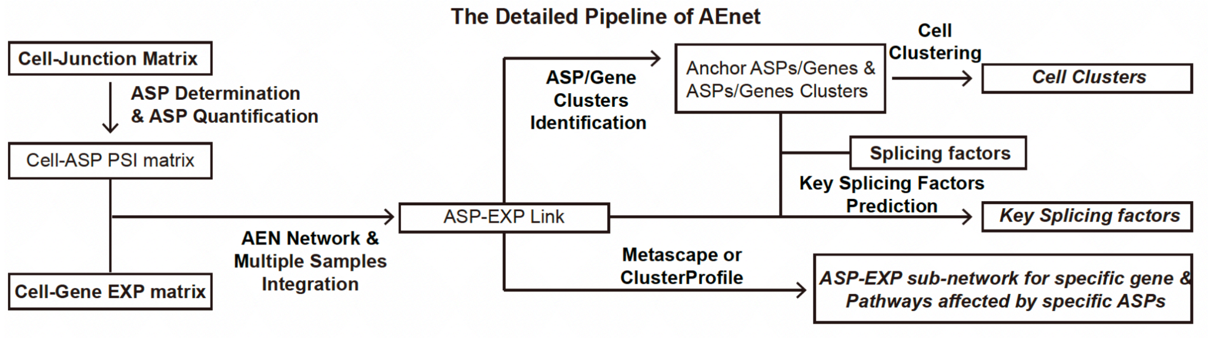

Figure S2. Schematic overview of the AEnet pipeline. Inputs (Normal): Cell–Junction Count Matrix, Cell–Gene Expression Matrix, and a predefined list of splicing factors. Key steps are highlighted in bold. Outputs (in bold Italic): Cell clusters, predicted key splicing factors, and signaling pathways regulated by specific alternative splicing patterns (ASPs).

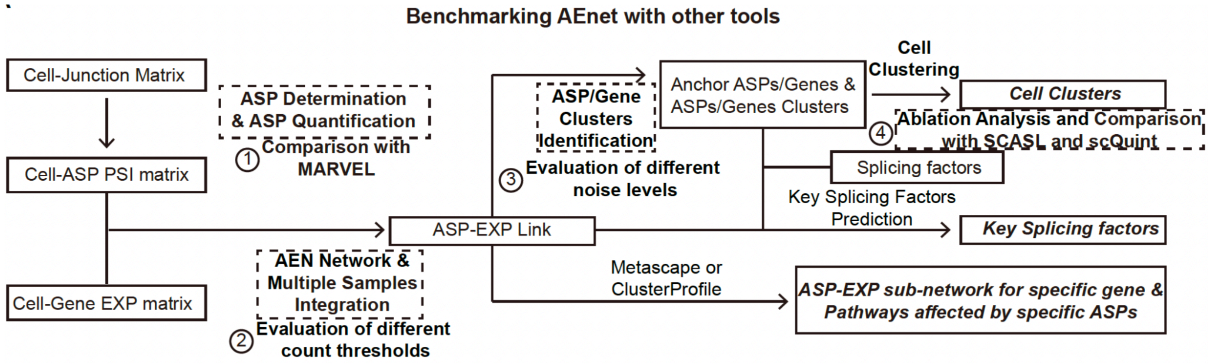

Figure S3. Schematic overview of the pipeline for the comprehensive evaluation of AEnet, organized into four steps: (1) ASP determination and quantification (compared with MARVEL), (2) AEN network construction and multi-sample integration (evaluation of different count thresholds), (3) ASP/gene cluster identification (assessment under varying noise levels), and (4) cell clustering (ablation analysis and comparison with SCASL and scQuint).

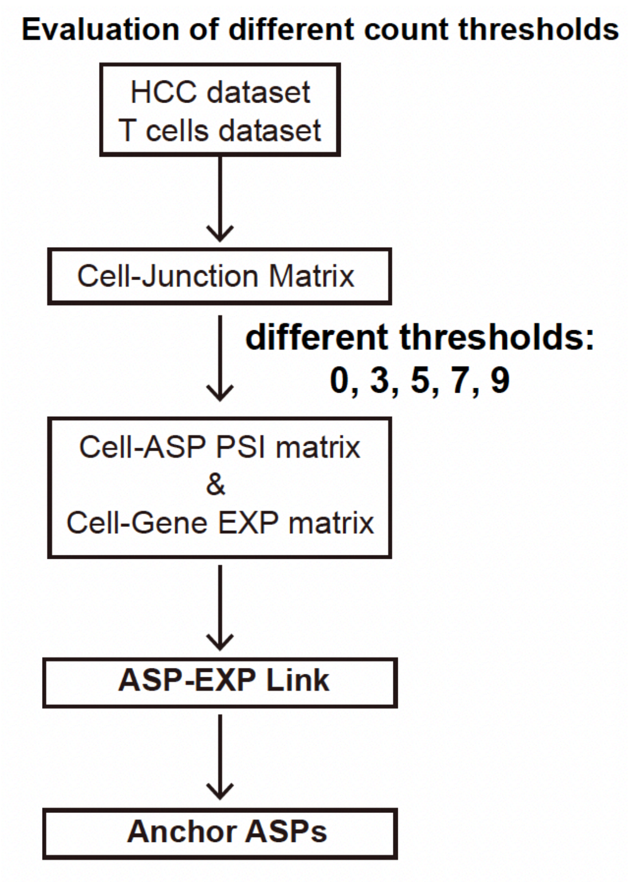

Figure S4. Schematic overview of benchmarking strategy for evaluating different count thresholds on the construction of ASP-EXP Link and Anchor ASPs.

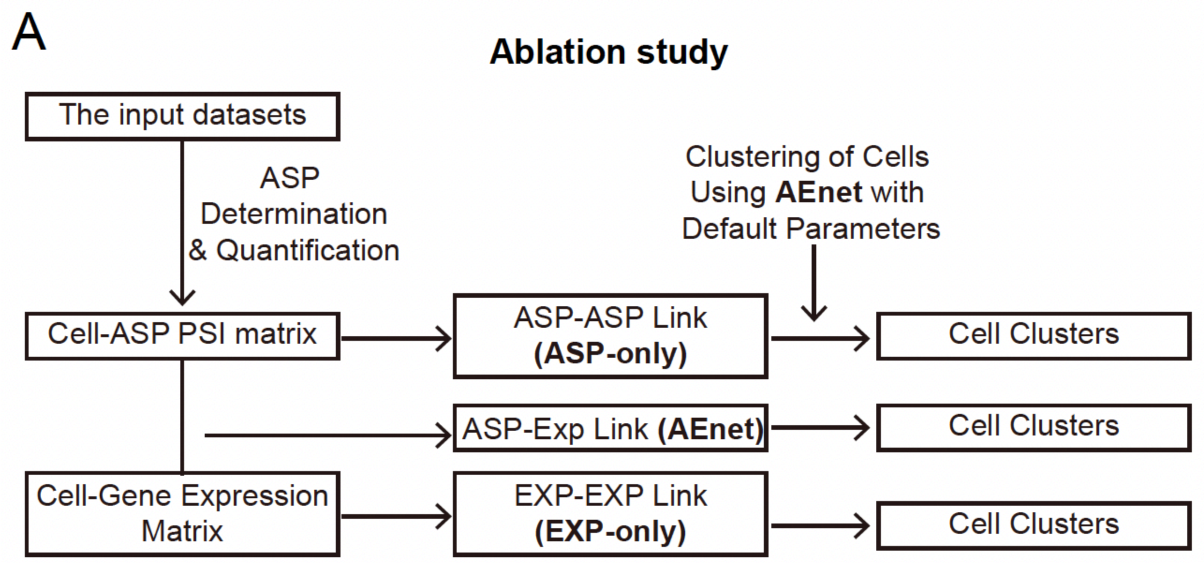

Figure 2. Schematic diagram of the assessment pipeline for ASP-only, RNA-only, and joint clustering analyses, with all other processing steps remaining identical to those in AEnet.

* In a few places, the revised manuscript still contains language that seems directly copied from the authors' point-by-point response. The writing would benefit from another editing pass to smooth out tone and narrative flow before final publication.

Response: Thank you for this astute observation. We fully agree that any residual textual overlap between the manuscript and our point-by-point response is inappropriate for final publication. To address this comprehensively, we have revised the relevant sections of the manuscript. Specifically, the text on **Page 10, Lines 248–251** has been updated as follows:

"While filtering low-count ASPs improves analytical reliability, we also considered the potential risk of excluding rare but biologically relevant splicing events. To mitigate this, AEnet defines an ASP as 'valid' in a sample only if it is supported by ≥5 reads across ≥20 cells. ASPs failing this threshold are excluded from downstream analysis due to their sparsity, which compromises the reliability of similarity estimates between splicing and expression. To evaluate whether this filtering inadvertently excludes informative, low-abundance ASPs, we stratified all ASPs into five categories based on the number of supporting cells: Invalid (≤20 cells; excluded), Type 1 (>20–30 cells), Type 2 (>30–40 cells), Type 3 (>40–50 cells), and Type 4 (>50 cells). An ASP was assigned to the highest applicable category if it met the criteria in ≥3 samples (Fig. S4F)."

Additionally, **Page 24, Lines 531–536** has been revised to read:

"In summary, while rigid clustering provides discrete groupings, our similarity-based regional analysis reveals underlying transitions and trajectories among ASP-defined clusters. These findings underscore the dynamic continuum of T cell state transitions and highlight the utility of complementary approaches in capturing intermediate cell states that may be overlooked by strict partitioning."

Beyond these specific changes, we have thoroughly re-evaluated the entire manuscript and revised the relevant sections (Page 3, Lines 75–79; Page 9, Lines 222–227; Page 10, Lines 248–251 and 253–256; and figure legends for Figs. S1–S4) to ensure consistency, accuracy, and seamless integration with the overall narrative. The manuscript has also undergone a comprehensive editorial revision to enhance clarity, tone, and flow throughout.
