# Supplementary material for: AEnet: a practical tool to construct the splicing-associated phenotype atlas at a single cell level
Source: Gigascience. 2025 Sep 24;14:giaf110. doi: 10.1093/gigascience/giaf110 (PMC12457822; doi:10.1093/gigascience/giaf110)
Supplement: giaf110_Authors_Response_To_Reviewer_Comments_original_submission [file giaf110_authors_response_to_reviewer_comments_original_submission.pdf]

Author's Response To Reviewer Comments

Reviewer reports:

Reviewer #1: This paper presents AEnet, a cell clustering method based on the combined gene expression levels and AS patt
shallow, and not convinced. I have several concerns about the contents of this paper:

(1) Since the inference of AS patterns is the foundation for subsequent analysis and the authors proposed a new approach to
efficiency to quantify the AS patterns.

(2) The authors assumes that the combined gene expression levels and AS patterns is more accurate in identifying the cell cl
(gene expression only) by replacing combined AE matrix with the AS patterns matrix or gene expression matrix and keeping

(3) I observe that the authors compared the performance of AEnet and SCASL (single-cell clustering method based on AS) in
and/or cell-type annotation accuracy based on the ground-truth cell labels, rather than the patient samples.

(4) Beside the ablation study, the comparison with other widely-used single-cell clustering methods should be conducted, e.g

(5) In Fig. 2, the authors mentioned that AEnet can decrease the bias or batch effects. If so, the benchmarked evaluations w

(6) Since the AEnet is a universal method for clustering, more benchmarked datasets are needed, including the datasets obta
(7) Typo "cell cells" in "how alternative splicing influences cell cells and cell states".

Reviewer #2: Liu et al. developed a novel software (AEnet) for analysing alternative splicing (AS) at single-cell resolution. Th
and pathway enrichment analysis. Overall, the software demonstrates sufficient novelty over existing single-cell AS tools to w
improving the already high-quality of the manuscript.

Main comments:

1. In Fig. S3A (left panel) and S3C (left panel), were the cell clusters identified by AEnet biologically meaningful? For example

2. The clustering capability of AEnet has not been benchmarked against datasets in which the cell types are known (ground tr
consists of a wide diversity of confidently-assigned cell types suitable for this benchmarking exercise.

3. The clustering capability of AEnet was compared against SCASL and Seurat by visually inspecting the clustering profile on
normalised mutual information (NMI).

4. The ability of AEnet to mitigate batch effect was compared against Seurat and SCASL. For Seurat specifically, the batch eff
correction, more suitable approaches for adjusting batch effect should be benchmarked against. The canonical correlation ana

5. It would be of particular interest to benchmark AEnet against other splicing-based clustering approaches. The variational a
5474597%2Fv1&data=05%7C02%7Cwuliang%40genomics.cn%7C19524fec513b46a5e91e08dd65e932ef%7C853aa2281adc
nbgoIhPFzDFAkxTMiwh1cM8PlxZo58M7e2jgco%3D&reserved=0) have demonstrated convincing clustering ability.

6. I appreciate the detailed demonstration of AEnet on tumour-infiltrating T cells and embryonic gastrulation using AEnet, and
factors, namely SF3B1, SRSF2, and U2AF1, and therefore are known to have dysregulated splicing profiles. Moreover, severa
exercise. Furthermore, related to comment no. 2 above, the haematopoietic compartment consists of a wide variety of cell ty
demonstrating the application of AEnet. One such example is a dataset that consists of SFB31-mutant MDS samples (PMID: 3

7. The cornerstone of single-cell RNA-sequencing analysis is differential gene and splicing analysis. AEnet excels in revealing
a limitation in itself, this may be detailed in the Discussion to aid users in deciding which single-cell AS tools will be best suite

8. For the purpose of reproducibility and to convince prospective users, like myself, to use AEnet, please may the authors pro

Minor comments:
9. Please may the authors provide the pre-defined list of splicing factors in a Supplementary Table.
10. In paragraph 1 of the Introduction, "… analyzing profilings of AS…" should be "…analyzing profiles of AS…".

11. In paragraph 2 of the Introduction, the authors alluded to the inflated false negatives exemplified by published single-cell

12. In paragraph 2 of the Introduction, the authors also mentioned that current single-cell AS tools do not identify functional

13. In paragraph 3 of the Introduction, the authors listed three challenges of single-cell AS analysis. Please may the authors

Reviewer #3: In this work, Liu & Chen et al. present AEnet, a computational method for analyzing alternative splicing (AS) at
tumor immunotherapy response, tumor-infiltrating T cells, and embryonic development. This work integrates two distinct but
publication.
Major points:
* Retaining NaNs for zero counts is valid, but even low-count ASPs may introduce random fluctuations in PSIs. Have you test

* The cell populations identified by ASP are demonstrated to be independent of RNA-based clustering. Have you directly comp

* The uniform noise model used in evaluations may not reflect biological noise. Have you tested more realistic noise models?

* AEnet claims to bypass the batch effects, but the mechanism should be better explained. Is this achieved because correlati

* From your results (e.g., Fig. S5C), there seem to be cross-cluster interactions. Are you concerned that rigid clustering may

* Alternative splicing inherently provides positional information. Have you analyzed whether splicing events are enriched in sp

Minor points:
* The criteria for defining a cell subpopulation should be stated in the main text.
* Are the same sequencing results used for gene count matrix and ASP detection?
* The first section provides both method description and validation/benchmarking. Consider separating them into distinct sec
* Can AEnet handle larger single-cell datasets? How does it scale computationally?
* Figures in the first section lack self-explanatory captions. Some (e.g., Fig. 1C, S1B) do not provide sufficient information.
* The anchor ASP identification plots need x-axes, and the y-axes should be labeled "Number of ASP-Gene Links" instead of j
* The cited work from Regev's lab in the introduction is a bit old. Are there more recent references that should be included?

Point-by-point response to the referees’ comments

The general response to the reviewers:
We sincerely thank all the reviewers for their time and for providing valuable comments and suggestions, which have significa

Reviewer reports:

Reviewer #1: This paper presents AEnet, a cell clustering method based on the combined gene expression levels and AS patt
shallow, and not convinced. I have several concerns about the contents of this paper:

(1) Since the inference of AS patterns is the foundation for subsequent analysis and the authors proposed a new approach to
efficiency to quantify the AS patterns.

Response: Thank you for highlighting this critical point. We have rigorously benchmarked AEnet against BRIE, Outrigger, MAR
Current methods (BRIE [1], Outrigger [2], and MARVEL [3]) are all fundamentally annotation-dependent and therefore unabl
previous study [3], we next compared the AS events detected by AEnet and MARVEL using the demo dataset of MARVEL, whi
MARVEL's iPSC dataset, AEnet detected 722,278 additional AS patterns (63% unannotated) across 12,866 genes, with MSE d

Type	BRIE	Outrigger	MARVEL	DESJ-detection	AEnet
SE	√	/	√	√	√
MXE	/	√	√	√	√
RI	/	/	√	/	/
A3SS	/	/	√	√	√
A5SS	/	/	√	√	√
AFE	/	/	√	√	√
ALE	/	/	√	√	√
MSE	/	/	√	√	√
Unannotated AS events	/	/	/	√	√

Table R1. Summary of the five methods.

Figure R1. (A) The common types of Alternative splicing. (B) The comparison between AEnet and MARVEL in the detection of
detected by AEnet.
Compared to our homologous method DESJ-detection [5], AEnet overcomes critical limitations in low-depth robustness. DESJ
distributions to transcript usage ratios, reducing PSI error by 47% (p<0.001, Fig. R1H).
Taken together, AEnet outperforms existing methods in detecting unannotated alternative splicing events, resolving complex

Figure R1. (G) Schematic illustration of PSI calculation in DESJ-detection. (H) Impact of Low Gene Expression on the Accurac

(2) The authors assumes that the combined gene expression levels and AS patterns is more accurate in identifying the cell cl
(gene expression only) by replacing combined AE matrix with the AS patterns matrix or gene expression matrix and keeping
Response: We appreciate your insightful comment. To serve the question, three independent datasets—iPSC [2], T cell [6], a
outperformed the standalone AS-only (Anet) and expression-only (Enet) approaches (Fig. R2A). Specifically, the median ARI
demonstrate that integrating ASP features with gene expression markedly improves clustering resolution and biological interp
distinct T cell subsets—capabilities that were limited or absent in the AS-only and EXP-only models. Thus, AEnet consistently
revised Figure 2A-D and S7.

Table R2. Summary of the three datasets

Figure R2. (A) Schematic of the assessment pipeline for ASP-only, RNA-only, and joint clustering analyses. (B-D) Quantitative
tests. * P < 0.05, ** P < 0.01, *** P < 0.001, **** P < 0.0001; n.s., not significant.

Figure R2. (E–F) Uniform Manifold Approximation and Projection (UMAP) visualizations showing the published cell type annota
cell RNA-seq datasets. (G) UMAP visualizations showing the published cell type annotations (botton) and the predicted cluster

(ARI).

(3) I observe that the authors compared the performance of AEnet and SCASL (single-cell clustering method based on AS) in
and/or cell-type annotation accuracy based on the ground-truth cell labels, rather than the patient samples.
Response: We appreciate the reviewer's suggestion to use ARI and NMI metrics for a more objective comparison between AE
clustering. This demonstrates that AEnet not only reduces batch effects but also provides more accurate cell cluster identifica
Comprehensive benchmarking demonstrates that AEnet consistently outperforms SCASL in clustering accuracy, achieving hig
embeddings across all datasets. In the iPSC dataset, SCASL failed to effectively distinguish iPSCs from NPCs, whereas AEnet
lymphoid populations (Fig. R3B, E). Similarly, in the T cell dataset, SCASL generated overlapping clusters and failed to resolv
accurately capture cellular heterogeneity and biologically meaningful substructures, establishing it as a superior tool for single

Figure R3. (A-C) Quantitative benchmarking of clustering concordance using (left) ARI and (right) NMI metrics of AEnet and S
RNA-seq datasets, and the (F) T cell dataset. Clustering solutions shown represent those with the highest adjusted Rand inde

(4) Beside the ablation study, the comparison with other widely-used single-cell clustering methods should be conducted, e.g
Response: We fully appreciate your careful assessment of this issue. We evaluated the clustering performance of AEnet, scSH
yielded a single clustering result. Moreover, scSHC does not provide functionality for generating low-dimensional embeddings
To ensure consistency across methods, the cell–junction count matrix was used as input for both scSHC and Seurat. Compreh
the HCC dataset, 0.58 (AEnet), 0.29 (Seurat), and 0.10 (scSHC); and for the T cell dataset, 0.42 (AEnet), 0.36 (Seurat), and
fragmented clusters. Notably, these mini-clusters were primarily driven by junctions from specific genes—such as ACTG1—an
Seurat and scSHC in clustering accuracy across multiple datasets, offering more biologically meaningful clustering and interpr
We sincerely thank the reviewer for this insightful recommendation. In response, we have conducted the suggested comparat
rather than splicing-based inference—we have decided not to include these results in the main text at this stage. We believe

Figure R4. (A-C) Quantitative benchmarking of clustering concordance using (left) ARI and (right) NMI metrics of AEnet and S
RNA-seq datasets, and the (F) T cell dataset. Clustering solutions shown represent those with the highest adjusted Rand inde

(5) In Fig. 2, the authors mentioned that AEnet can decrease the bias or batch effects. If so, the benchmarked evaluations w
Response: We thank the reviewer for the insightful comment. In response, we conducted benchmarking comparisons to evalu
To ensure a fair comparison, we applied two widely used batch correction methods—Seurat CCA [10] and Harmony[11]—to t
detect key splicing events and fail to capture splicing-driven cellular heterogeneity. Consequently, AEnet outperformed both m
(e.g., ACTG1-associated mini-clusters) rather than true alternative splicing patterns (Fig. R5C-D).
By contrast, AEnet successfully preserves AS-based clustering structure and captures shared splicing variation across multiple
designed to model splicing-specific heterogeneity. In summary, AEnet offers a splicing-aware alternative to general batch cor

Figure R5. (A-B). Quantitative benchmarking of clustering concordance using (A) Adjusted Rand Index (ARI) and (B) Normaliz
for the T cells datasets. Clustering solutions shown represent those with the highest adjusted Rand index (ARI). (E) The differ
shows the clustering of cell types determined by AEnet (G) and SCASL (H) (left panel) and patient clustering (right panel) for
not significant

(6) Since the AEnet is a universal method for clustering, more benchmarked datasets are needed, including the datasets obta
Response: We thank the reviewer for raising this important point regarding the generalizability of AEnet. Owing to the ultra-h
when sequencing depth is adequate. To further demonstrate its versatility, we applied AEnet to a widely used 10X Genomics
Due to the limited sequencing depth and only single-end coverage for transcript (5' end or 3' end) of 10X data, alternative sp
data. In this dataset, AEnet identified 190 anchor alternative polyadenylation (APA) events and delineated 7 APA-defined clus
(Treg) cells (Fig. R6C). Clustering results were consistent with established biological annotations. Quantitative benchmarking
isoform usage to cellular heterogeneity based on 10x Genomics datasets.

Figure R6. (A) Schematic of the assessment pipeline for AEnet applied to the PBMC dataset from 10x Genomics. (B) Heatmap
evaluated against the ground truth annotations of the PBMC dataset.

(7) Typo "cell cells" in "how alternative splicing influences cell cells and cell states".

Response: The phrase "cell cells" was indeed a typographical error, and we have corrected to "how alternative splicing influer

Reviewer #2: Liu et al. developed a novel software (AEnet) for analysing alternative splicing (AS) at single-cell resolution. Th
and pathway enrichment analysis. Overall, the software demonstrates sufficient novelty over existing single-cell AS tools to w
improving the already high-quality of the manuscript.

Main comments:

1. In Fig. S3A (left panel) and S3C (left panel), were the cell clusters identified by AEnet biologically meaningful? For example
Response: We are grateful for this thoughtful comment. The clusters identified by AEnet in Fig. S3A and S3C do exhibit clear
from progressive disease (PD; subpopulation S2), to residual disease (RD; S0), to a normal response state (S1), as visualized
genes (PLAT, PLAUR), gap-junction proteins (GJB2/3/5), and the well-known EMT (epithelial-mesenchymal transition) marker
In the colorectal cancer (CRC) [6] and recurrent hepatocellular carcinoma (RHCC) [7]T cell datasets, AEnet also effectively ca
CREM), S1 displayed effector T cell markers (NKG7, GZMA/B), S2 and S4 represented exhausted T cell states (PDCD1, CTLA4
functional state transitions rather than in defining classical T cell lineages. TThis interpretation is in the revised manuscript at

Figure R7. (A) UMAP shows the clustering of cell types determined by AEnet for epithelial cells from multiple patients. (B) The
clustering of cell types determined by AEnet for T cells from the CRC (Colorectal cancer) and RHCC (Recurrent Hepatocellular

2. The clustering capability of AEnet has not been benchmarked against datasets in which the cell types are known (ground tr
consists of a wide diversity of confidently-assigned cell types suitable for this benchmarking exercise.
Response: Thank you for highlighting this important point. We next focused on evaluating the clustering capability of AEnet u
in Table R3.
Comprehensive ablation analyses using published cell type annotations as ground truth demonstrated that the joint ASP-EXP
(AEnet), 0.10 (Anet), and 0.39 (Enet) for the HCC dataset; and 0.42 (AEnet), 0.10 (Anet), and 0.32 (Enet) for the T cell data
embeddings across all datasets (Fig. R8E–G), accurately capturing cellular architecture in iPSCs, delineating major lineages in
expression to reveal biologically meaningful cellular heterogeneity. We have incorporated this suggestion into the manuscript
As a preliminary attempt, we analyzed a publicly available PBMC dataset (GSE132044), which includes two samples and 528
3,202 alternative splicing patterns were detected, resulting in just 6,524 ASP-EXP links. This led to the identification of only 6
and we plan to incorporate higher-depth datasets in future evaluations to further demonstrate the generalizability of AEnet.

Table R3. Summary of the three datasets

Figure R8. (A) Schematic of the assessment pipeline for ASP-only, RNA-only, and joint clustering analyses. (B-D) Quantitative
tests. * P < 0.05, ** P < 0.01, *** P < 0.001, **** P < 0.0001; n.s., not significant.

Figure R8. (E–F) Uniform Manifold Approximation and Projection (UMAP) visualizations showing the published cell type annota
cell RNA-seq datasets. (G) UMAP visualizations showing the published cell type annotations (botton) and the predicted cluster
(ARI). (H) Detailed summary of the PBMC dataset.

3. The clustering capability of AEnet was compared against SCASL and Seurat by visually inspecting the clustering profile on r
normalised mutual information (NMI).
Response: Thank you for highlighting this important point. We fully agree that clustering performance should be evaluated us
comprehensive benchmarking analyses across three datasets—iPSC, T cell, and HCC—using published cell type annotations a
AEnet consistently outperformed SCASL [8] in clustering accuracy across all datasets: iPSC (AEnet: 0.81 vs. SCASL: 0.37), T
NPCs, while AEnet accurately resolved these subpopulations (Fig. R9A, D). In the HCC dataset, AEnet clearly separated lymph
overlapping and poorly resolved clusters generated by SCASL (Fig. R9C, F). We have incorporated this suggestion into the ma
We also compared AEnet to Seurat [10] using ARI and NMI, applying the same cell–junction count matrix to ensure a fair con
scSHC: 0.26) (Fig. R10A–C). While Seurat performed comparably in the iPSC dataset, it produced overly complex and fragme
heterogeneity (Fig. R10G). This suggests Seurat may be suboptimal for identifying isoform-level variation.
Taken together, these results demonstrate that AEnet not only outperforms SCASL but also provides more accurate, biologica

Figure R9. (A-C) Quantitative benchmarking of clustering concordance using (left) ARI and (right) NMI metrics of AEnet and S
(botton) for: (D) full-length iPSC (E) hepatocellular carcinoma (HCC) single-cell RNA-seq datasets, and the (F) T cell dataset.

Figure R10. (A-C) Quantitative benchmarking of clustering concordance using (left) ARI and (right) NMI metrics of AEnet and
(botton) for: (D) full-length iPSC (E) hepatocellular carcinoma (HCC) single-cell RNA-seq datasets, and the (F) T cell dataset.

4. The ability of AEnet to mitigate batch effect was compared against Seurat and SCASL. For Seurat specifically, the batch eff
correction, more suitable approaches for adjusting batch effect should be benchmarked against. The canonical correlation ana
Response: We thank the reviewer for the insightful comment regarding the need for more suitable batch correction baselines
CCA [10] and Harmony [11]—to the cell–junction count matrix prior to PSI computation, ensuring fair comparison (Fig. R11A
for gene expression data and are not well-suited for alternative splicing analysis. In particular, they lack the sensitivity to det
R11A-B). Moreover, CCA and Harmony tended to overcorrect, resulting in fragmented clusters driven by expression noise (e.
conditions, compared to SCASL (Fig. R5E–H). In summary, AEnet offers a splicing-aware alternative to general batch correcti

Figure R11. (A-B). Quantitative benchmarking of clustering concordance using (A) Adjusted Rand Index (ARI) and (B) Norma
for the T cells datasets. Clustering solutions shown represent those with the highest adjusted Rand index (ARI). (E) The differ
shows the clustering of cell types determined by AEnet (G) and SCASL (H) (left panel) and patient clustering (right panel) for
not significant

5. It would be of particular interest to benchmark AEnet against other splicing-based clustering approaches. The variational a
5474597%2Fv1&data=05%7C02%7Cwuliang%40genomics.cn%7C19524fec513b46a5e91e08dd65e932ef%7C853aa2281adc
nbgoIhPFzDFAkxTMiwh1cM8PlxZo58M7e2jgco%3D&reserved=0) have demonstrated convincing clustering ability.
Response: We thank the reviewer for this important suggestion. To benchmark AEnet against other splicing-based clustering
As scQuint primarily employs a variational autoencoder (VAE) for embedding, we performed clustering on the scQuint-derived
However, in the more complex T cell dataset and HCC datasets, AEnet effectively resolved well-defined functional subsets and
DOLPHIN study, as BAM files required for exon count matrix construction were unavailable for our own datasets (Fig. R12E).
DOLPHIN's performance (Fig. R12F–G). In addition, AEnet produced informative low-dimensional embeddings across all three
Collectively, AEnet outperforms scQuint in resolving fine-grained cellular subtypes and matches DOLPHIN's clustering accurac

Figure R12. (A) Schematic of the assessment pipeline for AEnet and scQuint. (B-D) Uniform Manifold Approximation and Proje
Schematic illustration of the assessment pipeline used to compare AEnet and DOLPHIN. (F–G) Quantitative benchmarking of
the three DOLPHIN benchmark datasets: PBMC (H), PDAC (I), and Colon (J).

6. I appreciate the detailed demonstration of AEnet on tumour-infiltrating T cells and embryonic gastrulation using AEnet, and
factors, namely SF3B1, SRSF2, and U2AF1, and therefore are known to have dysregulated splicing profiles. Moreover, severa
exercise. Furthermore, related to comment no. 2 above, the haematopoietic compartment consists of a wide variety of cell ty
demonstrating the application of AEnet. One such example is a dataset that consists of SF3B1-mutant MDS samples (PMID: 3
Response: We sincerely thank the reviewer for the valuable suggestion regarding the use of SF3B1-mutant myelodysplastic s
(e.g., SF3B1, SRSF2, U2AF1), represent highly relevant biological systems for investigating alternative splicing dysregulation
To preliminarily explore this direction, we applied AEnet to the dataset referenced in PMID: 37582363 [17]. AEnet identified a
megakaryocyte-erythroid progenitors (MEP), megakaryocyte progenitors (MKP), hematopoietic stem and progenitor cells (HS
Information (NMI) of 0.5 (Fig. R13B). These findings support AEnet's ability to resolve complex cellular heterogeneity in clinic
We next examined the distribution of wild-type (WT) and mutant (MUT) cells in MDS patients and found that WT cells were en
The splicing factor most strongly associated with this cluster was HNRNPA1 (Fig. R13E), which regulates the alternative splici
Due to time constraints and in order to maintain the manuscript's conceptual focus, we have not incorporated this analysis in
available alongside the AEnet package to support reproducibility and further exploration by the community.

Figure R13. (A) UMAP visualizations showing the published cell type annotations (right) and the predicted clusters (left) deriv
MDS dataset. (C) The distribution of SF3B1 WT and MUT cells in the MDS patients. (D) The distribution of enrichment scores

7. The cornerstone of single-cell RNA-sequencing analysis is differential gene and splicing analysis. AEnet excels in revealing
a limitation in itself, this may be detailed in the Discussion to aid users in deciding which single-cell AS tools will be best suite
Response: We thank the reviewer for this thoughtful and constructive comment. AEnet is designed to integrate alternative sp
programs, and functional consequences within a single biological context, such as a specific sample or condition.
As correctly noted by the reviewer, AEnet is not currently designed for direct differential splicing comparisons across clusters
Nevertheless, the anchor ASPs and regulatory modules identified by AEnet could serve as informative features for downstrea
We agree that this functional scope should be made explicit for users when choosing the most suitable tool for their research
group-wise differential splicing analysis, users may consider integrating AEnet's outputs with dedicated statistical testing fram
Softwares/Steps SCASL MARVEL DESJ-detection Expenditure (Outrigger) BRIE AEnet
quantitativeness √ √ √ √ √ √
imputation √ / / / √ /
feature selection / / / / / √
dimension reduction √ √ / / / √
cell clustering √ / / / / √
Differentially Splicing events Detection √ √ √ √ √ /
Modality classification / √ / √ / /
RNA velocity / / / / √ /
Splicing Factors prediction / / / / / √
Pathways affected by single splicing event prediction / / / / / √
Pathways enriched in differentially splicing genes / √ / / / /
Nonsense-mediated decay prediction / √ / / / /
RNA-sequencing platform a a&b a a a a
a, Plate-based sequencing; b, Droplet-based
Table R4. Summary of methods

8. For the purpose of reproducibility and to convince prospective users, like myself, to use AEnet, please may the authors pro
Response: We sincerely thank the reviewer for this constructive suggestion. In response, we have updated our R package, wh
●iPSC dataset: https://drive.google.com/file/d/1Qkg4De3DER4Qs5V_vP-M7GgwprwjwkLA/view?usp=drive_link
●T cell dataset: https://drive.google.com/file/d/1zuut5OlYgFeYXytU5kUZsYKbx07CKb56/view?usp=drive_link
We fully agree that providing the code used to generate manuscript figures is essential for transparency and reproducibility. T
future. In the meantime, we are happy to share preliminary versions of the scripts upon request. We appreciate the reviewer

Minor comments:
9. Please may the authors provide the pre-defined list of splicing factors in a Supplementary Table.
Response: Thanks for pointing this out. We have provided the pre-defined list of splicing factors in Supplementary Table S4.

10. In paragraph 1 of the Introduction, "… analyzing profilings of AS…" should be "…analyzing profiles of AS…".
Response: We thank the reviewer for their attentive reading and valuable feedback. The phrase "analyzing profilings of AS" h

11. In paragraph 2 of the Introduction, the authors alluded to the inflated false negatives exemplified by published single-cell
Response: We sincerely thank the reviewer for this correction. Upon re-evaluation, we recognize that our original statement c
'Although there are several bioinformatic approaches to studying single-cell AS profiling, most are based on canonical RNA-ba
expression, which may result in incomplete detection of splicing heterogeneity.'

12. In paragraph 2 of the Introduction, the authors also mentioned that current single-cell AS tools do not identify functional
Response: We sincerely thank the reviewer for pointing out that MARVEL has a functionality to identify enriched functional pa
R package, which can identify enriched pathways among differentially spliced genes. We have incorporated this suggestion in

13. In paragraph 3 of the Introduction, the authors listed three challenges of single-cell AS analysis. Please may the authors
Response: We thank the reviewer for raising this important point. We have added relevant citations [19–21] for each challeng

Reviewer #3: In this work, Liu & Chen et al. present AEnet, a computational method for analyzing alternative splicing (AS) at
tumor immunotherapy response, tumor-infiltrating T cells, and embryonic development. This work integrates two distinct but
publication.
Major points:
* Retaining NaNs for zero counts is valid, but even low-count ASPs may introduce random fluctuations in PSIs. Have you test
Response: We thank the reviewer for this insightful question. We fully agree that low-count ASPs can introduce noise into PS

assessed their influence on ASP-Exp link quantity, specificity, and anchor ASP detection (Fig. R14A).
Setting the threshold to 0 yielded the highest number of ASP-Exp links, with ~40% classified as "specific." However, 99% of
number of anchor ASPs, most of which were linked to low-confidence, sample-specific signals (Fig. R14D). By contrast, thresh
supporting reads to ensure PSI robustness while minimizing noise.
We also appreciate the reviewer's point about potential bias against rare but biologically relevant splicing events. In AEnet, a
and expression. To assess whether this filtering excludes informative low-abundance ASPs, we stratified all ASPs into five cat

- Invalid: ≤20 cells (excluded)
- Type 1: >20–30 cells
- Type 2: >30–40 cells
- Type 3: >40–50 cells
- Type 4: >50 cells

An ASP was assigned to the highest applicable category if it met the criteria in ≥3 samples (Fig. R14F). As expected, higher-s
identified as anchor ASPs (Fig. R14G–H), underscoring their potential functional relevance, albeit at a lower frequency than h
provides user-defined thresholding to support flexible analysis tailored to specific research goals. We have incorporated this s

Figure R14. (A) Schematic overview of benchmarking strategy for evaluating different count thresholds. (B) Quantification of
thresholds, with color coding denoting link specificity. (E) The similarity of anchor ASPs across different expression count cuto
ASPs stratified by ASP subtype classification.

* The cell populations identified by ASP are demonstrated to be independent of RNA-based clustering. Have you directly comp
Response: We appreciate your insightful comment. To serve the question, three independent datasets—iPSC [2], T cell [6], a
outperformed the standalone AS-only (Anet) and expression-only (Enet) approaches (Fig. R15A). Specifically, the median AR
demonstrate that integrating ASP features with gene expression markedly improves clustering resolution and biological interp
distinct T cell subsets—capabilities that were limited or absent in the AS-only and EXP-only models. Thus, AEnet consistently
revised Figure 2A-D and S7.

Table R5. Summary of the three datasets

Figure R15. (A) Schematic of the assessment pipeline for ASP-only, RNA-only, and joint clustering analyses. (B-D) Quantitativ
tests. * P < 0.05, ** P < 0.01, *** P < 0.001, **** P < 0.0001; n.s., not significant.

Figure R15. (E–F) Uniform Manifold Approximation and Projection (UMAP) visualizations showing the published cell type anno
cell RNA-seq datasets. (G) UMAP visualizations showing the published cell type annotations (botton) and the predicted cluster
(ARI).

* The uniform noise model used in evaluations may not reflect biological noise. Have you tested more realistic noise models?
Response: Thank you for highlighting this important point. We acknowledge that the uniform noise model may not adequatel
and Poisson noise and generated ASP–ASP similarity matrices (Jaccard index) under each noise condition to assess AEnet's ro
noise levels as high as 90% for both noise models (Fig. R15C and F). These results demonstrate AEnet's resilience to biologic

Figure R16. (A-B) Simulated ASPs-ASPs similarity matrix (B) was created with increasing gaussian noise (A). (C) Box plots pr
index of ASP clusters between the background and AENet with the poisson noise.

* AEnet claims to bypass the batch effects, but the mechanism should be better explained. Is this achieved because correlati

Response: We appreciate the reviewer's insightful comment regarding batch effects. Our hypothesis posits that if ASP-EXP lin
criteria: (i) Supported by only one sample or only one condition, or (ii) Displaying discordant directionality (positive/negative)
1 Associations supported by only a single sample (non-replicable), or
2 Associations showing statistically discordant regulatory effects (positive vs. negative correlations) across different samples/
We have incorporated this suggestion into the manuscript at Page4, Line 135-140 and Figure S1C.

Table R6 Systematic criteria for detecting batch-vulnerable ASP-EXP links across heterogeneous samples and experimental co

* From your results (e.g., Fig. S5C), there seem to be cross-cluster interactions. Are you concerned that rigid clustering may
Response: We appreciate the reviewer's insightful observation regarding cross-cluster interactions observed in our data (Fig.
organizing ASP clusters based on pairwise similarity and identifying three major regions, each comprising clusters with higher
Region 1 includes clusters C_7 and C_9, which exhibit the highest mutual similarity and are both predominantly enriched in n
trajectories—one toward memory T cells (C_5 and C_10), and the other toward effector T cells (C_4 and C_11) (Fig. R17B-G
Region 2 comprises clusters C_11, C_4, and C_14, which show strong inter-cluster similarity. C_4 acts as a central node, con
C_1—both associated with exhausted states—indicating a continuous exhaustion trajectory (Fig. R17B-E).
Region 3 implies an alternative exhaustion pathway, involving clusters C_5, C_10, and C_12. Here, C_10 links C_5 (memory/
exhaustion (Fig. R17B-G).
In summary, while rigid clustering provides discrete groupings, our similarity-based regional analysis reveals underlying trans
strict partitioning. We have incorporated this suggestion into the manuscript at Page 24, Line 514-538 and revised Figure S1(

Figure R17. (A) Heatmap showing ASP clusters derived from dimensionality reduction of AEnet on the pan-cancer T cell datas
selected ASP clusters.

* Alternative splicing inherently provides positional information. Have you analyzed whether splicing events are enriched in sp

Response: Yes, we have analyzed whether splicing events are enriched in specific genomic regions and found distinct, datase
were quantified within each bin. To identify statistically enriched regions, we applied permutation testing (n = 1000), with sig
This analysis revealed distinct, dataset-specific enrichment patterns (Fig. R18A-C). In the tumor cell dataset, the top three en
Chr1:150–160 Mb, Chr19:40–50 Mb, and Chr11:50–60 Mb. These distinct chromosomal distributions reflect the context-spec
Importantly, we identified recurrent enrichment in specific genomic regions across datasets, suggesting potential AS hotspots
existence of conserved splicing regulation at immune-associated loci, even amid divergent transcriptomic programs (Fig. R18
We sincerely thank the reviewer for this insightful recommendation. In response, we have performed the suggested compara
not to include this analysis in the current version. Nevertheless, we will provide the corresponding analysis scripts alongside c

Figure R18. (A-C) Chromosomal distribution of Alternative splicing sites across 24 chromosomes in three datasets: (A) tumor
highest AS densities are annotated, with statistically significant regions (hypergeometric test, $P < 0.05$) marked by asterisks.

Minor points:
* The criteria for defining a cell subpopulation should be stated in the main text.
Response: We appreciate the reviewer's suggestion and have now explicitly defined the criteria for subpopulation identificatio
approach differs from conventional clustering based solely on gene expression.

* Are the same sequencing results used for gene count matrix and ASP detection?
Response: The same sequencing data were used to generate both the gene count matrix and detect alternative splicing (AS)

* The first section provides both method description and validation/benchmarking. Consider separating them into distinct sec
Response: We thank the reviewer for highlighting the need for clearer methodological presentation. In response, we have rec

* Can AEnet handle larger single-cell datasets? How does it scale computationally?
Response: AEnet is theoretically capable of processing larger-scale datasets due to its inherent computational architecture. Th
identified AEN network construction as the rate-limiting step, prompting our implementation of a parallelized multi-core proce

Figure R19. Schematic diagram illustrating the application of AEnet to large-scale single-cell datasets, including computationa
* Figures in the first section lack self-explanatory captions. Some (e.g., Fig. 1C, S1B) do not provide sufficient information.
Response: We thank the reviewer for this important suggestion. The captions for Fig. 1C and S1B have been rewritten to incl
Samples Integration steps, as illustrated in Fig. 1B (Fig. R20B-C); (3) an updated explanation of the anchor ASP identificatior

Figure R20. (A) Schematic diagram illustrating the effect of splicing factors on the selection of splicing patterns. (B) Schemat

* The anchor ASP identification plots need x-axes, and the y-axes should be labeled "Number of ASP-Gene Links" instead of j
Response: Thank you for this helpful suggestion. In response, we have made the following revisions: (1) added x-axis labels
interpretability of the figures. The corresponding figure legends have also been revised to reflect these updates.

* The cited work from Regev's lab in the introduction is a bit old. Are there more recent references that should be included?
Response: We sincerely appreciate the reviewer's constructive suggestion to update the literature citations. In response, we h

1.Clinical relevance: Huang et al. (2023) [22] revealed pervasive alternative splicing dysregulation in pituitary neuroendocrin
splicing abnormalities, thereby enhancing the molecular stratification of PitNETs.

2.Developmental and evolutionary stability of AS: Anoushka et al. (2024) [23]constructed a single-cell full-length isoform atla
isoforms influence protein structure, neurodevelopmental transitions, and disease susceptibility, with conserved splicing patte

3.Brain-specific splicing programs: David et al. (2019) [24] used single-cell transcriptomics to show that neurexin isoform pro
adulthood.

These additions support our argument by demonstrating that IRF7's differential AS events, as originally identified by Regev's

Reference
1. Huang Y, Sanguinetti G. BRIE: transcriptome-wide splicing quantification in single cells. Genome Biol. 18:1232017;
2. Song Y, Botvinnik OB, Lovci MT, Kakaradov B, Liu P, Xu JL, et al.. Single-cell alternative splicing analysis with Expedition re
3. Wen WX, Mead AJ, Thongjuea S. MARVEL: an integrated alternative splicing analysis platform for single-cell RNA sequencin
4. Linker SM, Urban L, Clark SJ, Chhatriwala M, Amatya S, McCarthy DJ, et al.. Combined single-cell profiling of expression an
5. Liu S, Zhou B, Wu L, Sun Y, Chen J, Liu S. Single-cell differential splicing analysis reveals high heterogeneity of liver tumor
6. Zhang L, Yu X, Zheng L, Zhang Y, Li Y, Fang Q, et al.. Lineage tracking reveals dynamic relationships of T cells in colorecta
7. Sun Y, Wu L, Zhong Y, Zhou K, Hou Y, Wang Z, et al.. Single-cell landscape of the ecosystem in early-relapse hepatocellula
8. Xiang X, He Y, Zhang Z, Yang X. Interrogations of single-cell RNA splicing landscapes with SCASL define new cell identities
9. Grabski IN, Street K, Irizarry RA. Significance analysis for clustering with single-cell RNA-sequencing data. Nat Methods. 2
10. Stuart T, Butler A, Hoffman P, Hafemeister C, Papalexi E, Mauck WM 3rd, et al.. Comprehensive integration of single-cell
11. Korsunsky I, Millard N, Fan J, Slowikowski K, Zhang F, Wei K, et al.. Fast, sensitive and accurate integration of single-cell
12. Haghverdi L, Lun ATL, Morgan MD, Marioni JC. Batch effects in single-cell RNA-sequencing data are corrected by matching
13. Barkas N, Petukhov V, Nikolaeva D, Lozinsky Y, Demharter S, Khodosevich K, et al.. Joint analysis of heterogeneous singl
14. Xue R, Zhang Q, Cao Q, Kong R, Xiang X, Liu H, et al.. Liver tumour immune microenvironment subtypes and neutrophil
15. Maynard A, McCoach CE, Rotow JK, Harris L, Haderk F, Kerr DL, et al.. Therapy-induced evolution of human lung cancer r
16. Benegas G, Fischer J, Song YS. Robust and annotation-free analysis of alternative splicing across diverse cell types in mic
17. Cortés-López M, Chamely P, Hawkins AG, Stanley RF, Swett AD, Ganesan S, et al.. Single-cell multi-omics defines the cel
18. Fang J, Bolanos LC, Choi K, Liu X, Christie S, Akunuru S, et al.. Ubiquitination of hnRNPA1 by TRAF6 links chronic innate i
19. Risso D, Perraudeau F, Gribkova S, Dudoit S, Vert J-P. A general and flexible method for signal extraction from single-cel
20. Tran HTN, Ang KS, Chevrier M, Zhang X, Lee NYS, Goh M, et al.. A benchmark of batch-effect correction methods for sing
21. Wan Y, Larson DR. Splicing heterogeneity: separating signal from noise. Genome Biol. 19:862018;
22. Huang Y, Guo J, Han X, Zhao Y, Li X, Xing P, et al.. Splicing diversity enhances the molecular classification of pituitary ne
23. Joglekar A, Hu W, Zhang B, Narykov O, Diekhans M, Marrocco J, et al.. Single-cell long-read sequencing-based mapping r
24. Lukacsovich D, Winterer J, Que L, Luo W, Lukacsovich T, Földy C. Single-cell RNA-seq reveals developmental origins and

Cookie Preference Center

We use cookies which are necessary to make our site work. We may also use additional cookies to analyze, improve and personalize our content and your digital experience. For more information, see our Cookie Policy.

You may choose not to allow some types of cookies. However, blocking some types may impact your experience of our site and the services we are able to offer. See the different category headings below to find out more or change your settings.

Allow all

Manage Consent Preferences

Strictly Necessary Cookies

Always active

These cookies are necessary for the website to function and cannot be switched off in our systems. They are usually only set in response to actions made by you which amount to a request for services, such as setting your privacy preferences, logging in or filling in forms. You can set your browser to block or alert you about these cookies, but some parts of the site will not then work.

Functional Cookies

These cookies enable the website to provide enhanced functionality and personalisation. They may be set by us or by third party providers whose services we have added to our pages. If you do not allow these cookies then some or all of these services may not function properly.

Performance Cookies

These cookies allow us to count visits and traffic sources so we can measure and improve the performance of our site. They help us to know which pages are the most and least popular and see how visitors move around the site.

Targeting Cookies

These cookies may be set through our site by our advertising partners. They may be used by those companies to build a profile of your interests and show you relevant adverts on other sites. If you do not allow these cookies, you will experience less targeted advertising.

Cookie List

Clear

Apply Cancel
Consent Leg.Interest

Confirm my choices

Powered by onetrus
