# Supplementary material for: AEnet: a practical tool to construct the splicing-associated phenotype atlas at a single cell level
Source: Gigascience. 2025 Sep 24;14:giaf110. doi: 10.1093/gigascience/giaf110 (PMC12457822; doi:10.1093/gigascience/giaf110)
Supplement: giaf110_Authors_Response_To_Reviewer_Comments_Revision_1 [file giaf110_authors_response_to_reviewer_comments_revision_1.pdf]

Author's Response To Reviewer Comments

Point-by-point response to the referees' comments:
Reviewer #1: The authors have addressed my concerns.

Reviewer #2: The authors have thoroughly addressed my comments. I am pleased to see that the benchmarking analysis is substantially improved and the utility of the software has been demonstrated in wider context, i.e., additional datasets.

My only remaining comment relates to Comment no. 8: While the authors provided the source code for the R package, and the dataset, I would encourage the authors to provide the R Markdown tutorial. This documentation should demonstrate the pre-processing steps and analysis of at least one example dataset and correspondingly reproduce selected figures in the manuscript. This is not only for the purpose of transparency and reproducibility, but this will in turn encourage prospective users, like myself, to apply this software.
Response: We sincerely thank the reviewer for the valuable suggestion. To facilitate reproducibility and help users get started, we also provide demonstration datasets corresponding to two biological systems using the R Markdown tutorial:
iPSC dataset: https://liushang17.github.io/ipsc.html
T cell dataset: https://liushang17.github.io/tcells.html
The corresponding R Markdown source code is also available for both datasets (https://github.com/liushang17/AEnet). These tutorials reproduce key steps and selected figures from the manuscript, offering prospective users a practical guide for applying the AEnet package to their own data.

Reviewer #3: I appreciate the authors' careful attention to the reviewers' comments. The updated manuscript has addressed many concerns raised in the reviews, especially
* Clear separation of model description and benchmarking improves clarity
* Benchmarking across multiple additional datasets and platforms enhance the generalizability
* Batch effect handling is better explained
* Ablation study (A-net vs. E-net) supports the value of joint modeling
* Noise modeling and ASP thresholding are better tested

Overall, this work has been strengthened. A few points should be fixed
* Some of the workflow figures (e.g., Fig. 2, S2-S4) are visually confusing and could benefit from clearer visual layout or annotations.
Response: We sincerely thank the reviewer for this constructive suggestion. We have revised the schematics and updated the figure descriptions and legends in Figs. 2 and S2–S4, by adjusting the layout for better readability, adding step-wise labels, and ensuring consistent visual elements across all panels.

Figure S2. Schematic overview of the AEnet pipeline. Inputs (Normal): Cell–Junction Count Matrix, Cell–Gene Expression Matrix, and a predefined list of splicing factors. Key steps are highlighted in bold. Outputs (in bold Italic): Cell clusters, predicted key splicing factors, and signaling pathways regulated by specific alternative splicing patterns (ASPs).

Figure S3. Schematic overview of the pipeline for the comprehensive evaluation of AEnet, organized into four steps: (1) ASP determination and quantification (compared with MARVEL), (2) AEN network construction and multi-sample integration (evaluation of different count thresholds), (3) ASP/gene cluster identification (assessment under varying noise levels), and (4) cell clustering (ablation analysis and comparison with SCASL and scQuint).

Figure S4. Schematic overview of benchmarking strategy for evaluating different count thresholds on the construction of ASP-EXP Link and Anchor ASPs.

Figure 2. Schematic diagram of the assessment pipeline for ASP-only, RNA-only, and joint clustering analyses, with all other processing steps remaining identical to those in AEnet.

* In a few places, the revised manuscript still contains language that seems directly copied from the authors' point-by-point response. The writing would benefit from another editing pass to smooth out tone and narrative flow before final publication.

Response: Thank you for this astute observation. We fully agree that any residual textual overlap between the manuscript and our point-by-point response is inappropriate for final publication. To address this comprehensively, we have revised the relevant sections of the manuscript. Specifically, the text on Page 10, Lines 248–251 has been updated as follows:
"While filtering low-count ASPs improves analytical reliability, we also considered the potential risk of excluding rare but biologically relevant splicing events. To mitigate this, AEnet defines an ASP as 'valid' in a sample only if it is supported by ≥5 reads across ≥20 cells. ASPs failing this threshold are excluded from downstream analysis due to their sparsity, which compromises the reliability of similarity estimates between splicing and expression. To evaluate whether this filtering inadvertently excludes informative, low-abundance ASPs, we stratified all ASPs into five categories based on the number of supporting cells: Invalid (≤20 cells; excluded), Type 1 (>20–30 cells), Type 2 (>30–40 cells), Type 3 (>40–50 cells), and Type 4 (>50 cells). An ASP was assigned to the highest applicable category if it met the criteria in ≥3 samples (Fig. S4F)."
Additionally, Page 24, Lines 531–536 has been revised to read:
"In summary, while rigid clustering provides discrete groupings, our similarity-based regional analysis reveals underlying transitions and trajectories among ASP-defined clusters. These findings underscore the dynamic continuum of T cell state transitions and highlight the utility of complementary approaches in capturing intermediate cell states that may be overlooked by strict partitioning."
Beyond these specific changes, we have thoroughly re-evaluated the entire manuscript and revised the relevant sections (Page 3, Lines 75–79; Page 9, Lines 222–227; Page 10, Lines 248–251 and 253–256; and figure legends for Figs. S1–S4) to ensure consistency, accuracy, and seamless integration with the overall narrative. The manuscript has also undergone a comprehensive editorial revision to enhance clarity, tone, and flow throughout.

Editorial Manager®

Cookie Preference Center

We use cookies which are necessary to make our site work. We may also use additional cookies to analyze, improve and personalize our content and your digital experience. For more information, see our Cookie Policy.

You may choose not to allow some types of cookies. However, blocking some types may impact your experience of our site and the services we are able to offer. See the different category headings below to find out more or change your settings.

Allow all

Manage Consent Preferences

Strictly Necessary Cookies

Always active

These cookies are necessary for the website to function and cannot be switched off in our systems. They are usually only set in response to actions made by you which amount to a request for services, such as setting your privacy preferences, logging in or filling in forms. You can set your browser to block or alert you about these cookies, but some parts of the site will not then work.

Functional Cookies

These cookies enable the website to provide enhanced functionality and personalisation. They may be set by us or by third party providers whose services we have added to our pages. If you do not allow these cookies then some or all of these services may not function properly.

Performance Cookies

These cookies allow us to count visits and traffic sources so we can measure and improve the performance of our site. They help us to know which pages are the most and least popular and see how visitors move around the site.

Targeting Cookies

These cookies may be set through our site by our advertising partners. They may be used by those companies to build a profile of your interests and show you relevant adverts on other sites. If you do not allow these cookies, you will experience less targeted advertising.

Cookie List

Clear

Apply Cancel

Consent Leg.Interest

Confirm my choices

Powered by onetrust
