# Supplementary material for: AEnet: a practical tool to construct the splicing-associated phenotype atlas at a single cell level
Source: Gigascience. 2025 Sep 24;14:giaf110. doi: 10.1093/gigascience/giaf110 (PMC12457822; doi:10.1093/gigascience/giaf110)
Supplement: giaf110_Reviewer_1_Report_Original_Submission — Xin Shao -- 3/12/2025 [file giaf110_reviewer_1_report_original_submission.pdf]

Reviewer Report

Title: AEnet: a practical tool to construct the splicing associated phenotype atlas at single cell level

Version: Original Submission **Date:** 3/12/2025

Reviewer name: Xin Shao

Reviewer Comments to Author:

This paper presents AEnet, a cell clustering method based on the combined gene expression levels and AS patterns for cell-type annotation with scRNA-seq data. This idea is very interesting and I can see the great efforts made by the authors. However, as a method paper, the limited benchmarked datasets and methods make this work too simple, shallow, and not convinced. I have several concerns about the contents of this paper:(1) Since the inference of AS patterns is the foundation for subsequent analysis and the authors proposed a new approach to infer the AS patterns from scRNA-seq data, this approach should be benchmarked with other known methods, such as BRIE, Outrigger, MARVEL, and their own earlier attempt, DESJ-detection, to demonstrate the superior accuracy or efficiency to quantify the AS patterns.(2) The authors assumes that the combined gene expression levels and AS patterns is more accurate in identifying the cell clusters than the traditional strategies. If so, the ablation study should be performed by only considering the AS patterns or gene expression levels, to demonstrate the superior accuracy of AEnet compared to so-called Anet (AS only) or Enet (gene expression only) by replacing combined AE matrix with the AS patterns matrix or gene expression matrix and keeping the other parts of processing totally same as that in AEnet.(3) I observe that the authors compared the performance of AEnet and SCASL (single-cell clustering method based on AS) in identifying the cell clusters (Fig. S3). But the authors focused on the performance of reducing batch effects. This is very unreasonable. Since they are both single-cell clustering methods, the evaluation index should be known ARI, NMI, and/or cell-type annotation accuracy based on the ground-truth cell labels, rather than the patient samples.(4) Beside the ablation study, the comparison with other widely-used single-cell clustering methods should be conducted, e.g., the Seurat clustering method based on the combined AE matrix (ASP & E), and scSHC, with the known ARI and NMI indexes.(5) In Fig. 2, the authors mentioned that AEnet can decrease the bias or batch effects. If so, the benchmarked evaluations with other known methods (such as Seurat CCA, Harmony, fastMNN, Conos, LIGER) should be performed to demonstrate the superior accuracy or efficiency to reduce the bias or batch effects.(6) Since the AEnet is a universal method for clustering, more benchmarked datasets are needed, including the datasets obtained from different scRNA-seq technologies (Smart-seq, Microwell-seq, 10X Genomics, etc.), species, tissues, conditions, diseases (including more non-cancer diseases).(7) Typo "cell cells" in "how alternative splicing influences cell cells and cell states".

Level of Interest

Please indicate how interesting you found the manuscript: Choose an item.

Quality of Written English

Please indicate the quality of language in the manuscript: Choose an item.

Declaration of Competing Interests

Please complete a declaration of competing interests, considering the following questions:

- Have you in the past five years received reimbursements, fees, funding, or salary from an organisation that may in any way gain or lose financially from the publication of this manuscript, either now or in the future?
- Do you hold any stocks or shares in an organisation that may in any way gain or lose financially from the publication of this manuscript, either now or in the future?
- Do you hold or are you currently applying for any patents relating to the content of the manuscript?
- Have you received reimbursements, fees, funding, or salary from an organization that holds or has applied for patents relating to the content of the manuscript?
- Do you have any other financial competing interests?
- Do you have any non-financial competing interests in relation to this paper?

If you can answer no to all of the above, write 'I declare that I have no competing interests' below. If your reply is yes to any, please give details below.

I declare that I have no competing interests

I agree to the open peer review policy of the journal. I understand that my name will be included on my report to the authors and, if the manuscript is accepted for publication, my named report including any attachments I upload will be posted on the website along with the authors' responses. I agree for my report to be made available under an Open Access Creative Commons CC-BY license (http://creativecommons.org/licenses/by/4.0/). I understand that any comments which I do not wish to be included in my named report can be included as confidential comments to the editors, which will not be published.

Choose an item.

To further support our reviewers, we have joined with Publons, where you can gain additional credit to further highlight your hard work (see: https://publons.com/journal/530/gigascience). On publication of this paper, your review will be automatically added to Publons, you can then choose whether or not to claim your Publons credit. I understand this statement.

Yes Choose an item.
