# Supplementary material for: AEnet: a practical tool to construct the splicing-associated phenotype atlas at a single cell level
Source: Gigascience. 2025 Sep 24;14:giaf110. doi: 10.1093/gigascience/giaf110 (PMC12457822; doi:10.1093/gigascience/giaf110)
Supplement: giaf110_Reviewer_2_Report_Original_Submission — Sean Wen -- 3/14/2025 [file giaf110_reviewer_2_report_original_submission.pdf]

Reviewer Report

Title: AEnet: a practical tool to construct the splicing associated phenotype atlas at single cell level

Version: Original Submission **Date:** 3/14/2025

Reviewer name: Sean Wen

Reviewer Comments to Author:

Liu et al. developed a novel software (AEnet) for analysing alternative splicing (AS) at single-cell resolution. This software leverages both alternative splicing and gene expression profiles (ASP and EXP, respectively) to build ASP-EXP links. These links are then utilised for identifying cell clusters, cluster-specific regulatory splicing factors and isoforms, and pathway enrichment analysis. Overall, the software demonstrates sufficient novelty over existing single-cell AS tools to warrant publication. The narrative is also sensible and coherent. My only comments pertain to the benchmarking of this software and application of this software beyond the contexts reported in this manuscript, and they are aimed at improving the already high-quality of the manuscript.Main comments:1. In Fig. S3A (left panel) and S3C (left panel), were the cell clusters identified by AEnet biologically meaningful? For example, do the clusters demonstrate expression of lineage-specific genes that would enable cell type assignment or identification?2. The clustering capability of AEnet has not been benchmarked against datasets in which the cell types are known (ground truth). Is AEnet able to delineate the different cell populations whose identity have been previously assigned? The myriads of published peripheral blood mononuclear cells (PBMCs) and bone marrow mononuclear cells (BMMCs) datasets consists of a wide diversity of confidently-assigned cell types suitable for this benchmarking exercise.3. The clustering capability of AEnet was compared against SCASL and Seurat by visually inspecting the clustering profile on reduced dimension space. While this initial visual-based inspection is sensible, an objective measurement could serve as an additional method of comparison. Two such metrices for clustering assessment are adjusted rand index (ARI) and normalised mutual information (NMI).4. The ability of AEnet to mitigate batch effect was compared against Seurat and SCASL. For Seurat specifically, the batch effect was "corrected" using the "FindIntegrationAnchors" and "IntegrateData" functions. These functions integrate multiple samples, but do not adjust for batch effect per se. To enable a fair comparison with Seurat, or other means of batch correction, more suitable approaches for adjusting batch effect should be benchmarked against. The canonical correlation analysis (CCA) and Harmony are two batch correction approaches implemented by Seurat that may be benchmarked against.5. It would be of particular interest to benchmark AEnet against other splicing-based clustering approaches. The variational autoencoder (VAE) algorithm implemented by scQuint (PMID: 35229721) and DOLPHIN (https://www.researchsquare.com/article/rs-5474597/v1) have demonstrated convincing clustering ability.6. I appreciate the detailed demonstration of AEnet on tumour-infiltrating T cells and embryonic gastrulation using AEnet, and the identification of regulatory splicing factors in these scenarios. In haematological malignancies such as myelodysplastic syndrome (MDS) and myeloproliferative neoplasm (MPN), more than half of the patients have mutations in splicing factors, namely SF3B1, SRSF2, and U2AF1, and therefore are known to have dysregulated splicing profiles. Moreover, several AS events

related to these splicing factors have already been experimentally validated, for example, SEPTIN2 and MAP3K7 are mis-spliced in SFB31-mutant MDS patients. These splicing events may serve as positive controls in benchmarking exercise. Furthermore, related to comment no. 2 above, the haematopoietic compartment consists of a wide variety of cell types suitable for showcasing AEnet functionalities. Therefore, these cancer types are biologically relevant for benchmarking and for demonstrating the application of AEnet. One such example is a dataset that consists of SFB31-mutant MDS samples (PMID: 37582363).7. The cornerstone of single-cell RNA-sequencing analysis is differential gene and splicing analysis. AEnet excels in revealing regulatory splicing factors, isoforms, and pathways within a cluster or condition. However, AEnet does not enable comparison across different clusters or conditions, e.g., disease versus healthy states. While the lack of this functionality is not a limitation in itself, this may be detailed in the Discussion to aid users in deciding which single-cell AS tools will be best suited to meet their research goals.8. For the purpose of reproducibility and to convince prospective users, like myself, to use AEnet, please may the authors provide the codes used to generate AEnet-related figures in the manuscript. A R Markdown format is highly encouraged, if possible.Minor comments:9. Please may the authors provide the pre-defined list of splicing factors in a Supplementary Table.10. In paragraph 1 of the Introduction, "… analyzing profilings of AS…" should be "…analyzing profiles of AS…".11. In paragraph 2 of the Introduction, the authors alluded to the inflated false negatives exemplified by published single-cell AS tools. Has this limitation been demonstrated or investigated for these tools? If not, I recommend the authors to tone down this assertion.12. In paragraph 2 of the Introduction, the authors also mentioned that current single-cell AS tools do not identify functional pathways. But MARVEL has a functionality to identify enriched functional pathways (PMID: 36631981).13. In paragraph 3 of the Introduction, the authors listed three challenges of single-cell AS analysis. Please may the authors provide the relevant citations for each of these challenges.

Level of Interest

Please indicate how interesting you found the manuscript: Choose an item.

Quality of Written English

Please indicate the quality of language in the manuscript: Choose an item.

Declaration of Competing Interests

Please complete a declaration of competing interests, considering the following questions:

- Have you in the past five years received reimbursements, fees, funding, or salary from an organisation that may in any way gain or lose financially from the publication of this manuscript, either now or in the future?
- Do you hold any stocks or shares in an organisation that may in any way gain or lose financially from the publication of this manuscript, either now or in the future?

- Do you hold or are you currently applying for any patents relating to the content of the manuscript?
- Have you received reimbursements, fees, funding, or salary from an organization that holds or has applied for patents relating to the content of the manuscript?
- Do you have any other financial competing interests?
- Do you have any non-financial competing interests in relation to this paper?

If you can answer no to all of the above, write 'I declare that I have no competing interests' below. If your reply is yes to any, please give details below.

I declare that I have no competing interests

I agree to the open peer review policy of the journal. I understand that my name will be included on my report to the authors and, if the manuscript is accepted for publication, my named report including any attachments I upload will be posted on the website along with the authors' responses. I agree for my report to be made available under an Open Access Creative Commons CC-BY license (http://creativecommons.org/licenses/by/4.0/). I understand that any comments which I do not wish to be included in my named report can be included as confidential comments to the editors, which will not be published.

Choose an item.

To further support our reviewers, we have joined with Publons, where you can gain additional credit to further highlight your hard work (see: https://publons.com/journal/530/gigascience). On publication of this paper, your review will be automatically added to Publons, you can then choose whether or not to claim your Publons credit. I understand this statement.

Yes Choose an item.
