# Supplementary material for: AEnet: a practical tool to construct the splicing-associated phenotype atlas at a single cell level
Source: Gigascience. 2025 Sep 24;14:giaf110. doi: 10.1093/gigascience/giaf110 (PMC12457822; doi:10.1093/gigascience/giaf110)
Supplement: giaf110_Reviewer_3_Report_Original_Submission — Cyrillus Tan -- 3/18/2025 [file giaf110_reviewer_3_report_original_submission.pdf]

Reviewer Report

Title: AEnet: a practical tool to construct the splicing associated phenotype atlas at single cell level

Version: Original Submission **Date:** 3/18/2025

Reviewer name: Cyrillus Tan

Reviewer Comments to Author:

In this work, Liu & Chen et al. present AEnet, a computational method for analyzing alternative splicing (AS) at the single-cell level. By integrating AS and scRNA-seq data, they identify alternative splicing pattern (ASP) clusters and use them to define cell subpopulations. The authors demonstrate the utility of their method in three biological contexts: tumor immunotherapy response, tumor-infiltrating T cells, and embryonic development. This work integrates two distinct but complementary data modalities, AS and scRNA-seq, into a single framework, improving the characterization of cellular heterogeneity. The method is well-designed and effectively applied, but several issues should be addressed before publication.Major points:* Retaining NaNs for zero counts is valid, but even low-count ASPs may introduce random fluctuations in PSIs. Have you tested different count thresholds? And how does it affect results? If an ASP is rare and mostly NaN, how do you decide whether to include it? Could this introduce bias against infrequent but biologically relevant splicing events?* The cell populations identified by ASP are demonstrated to be independent of RNA-based clustering. Have you directly compared ASP-only, RNA-only, and joint clustering to assess whether they provide unique biological insights?* The uniform noise model used in evaluations may not reflect biological noise. Have you tested more realistic noise models?* AEnet claims to bypass the batch effects, but the mechanism should be better explained. Is this achieved because correlations are calculated within each sample and aggregated later?* From your results (e.g., Fig. S5C), there seem to be cross-cluster interactions. Are you concerned that rigid clustering may overlook meaningful relationships between clusters?* Alternative splicing inherently provides positional information. Have you analyzed whether splicing events are enriched in specific genomic regions?Minor points:* The criteria for defining a cell subpopulation should be stated in the main text.* Are the same sequencing results used for gene count matrix and ASP detection?* The first section provides both method description and validation/benchmarking. Consider separating them into distinct sections and expanding each more thoroughly.* Can AEnet handle larger single-cell datasets? How does it scale computationally?* Figures in the first section lack self-explanatory captions. Some (e.g., Fig. 1C, S1B) do not provide sufficient information.* The anchor ASP identification plots need x-axes, and the y-axes should be labeled "Number of ASP-Gene Links" instead of just "Degree."* The cited work from Regev's lab in the introduction is a bit old. Are there more recent references that should be included?

Level of Interest

Please indicate how interesting you found the manuscript: Choose an item.

Quality of Written English

Please indicate the quality of language in the manuscript: Choose an item.

Declaration of Competing Interests

Please complete a declaration of competing interests, considering the following questions:

- Have you in the past five years received reimbursements, fees, funding, or salary from an organisation that may in any way gain or lose financially from the publication of this manuscript, either now or in the future?
- Do you hold any stocks or shares in an organisation that may in any way gain or lose financially from the publication of this manuscript, either now or in the future?
- Do you hold or are you currently applying for any patents relating to the content of the manuscript?
- Have you received reimbursements, fees, funding, or salary from an organization that holds or has applied for patents relating to the content of the manuscript?
- Do you have any other financial competing interests?
- Do you have any non-financial competing interests in relation to this paper?

If you can answer no to all of the above, write 'I declare that I have no competing interests' below. If your reply is yes to any, please give details below.

I declare that I have no competing interests.

I agree to the open peer review policy of the journal. I understand that my name will be included on my report to the authors and, if the manuscript is accepted for publication, my named report including any attachments I upload will be posted on the website along with the authors' responses. I agree for my report to be made available under an Open Access Creative Commons CC-BY license (http://creativecommons.org/licenses/by/4.0/). I understand that any comments which I do not wish to be included in my named report can be included as confidential comments to the editors, which will not be published.

Choose an item.

To further support our reviewers, we have joined with Publons, where you can gain additional credit to further highlight your hard work (see: https://publons.com/journal/530/gigascience). On publication of this paper, your review will be automatically added to Publons, you can then choose whether or not to claim your Publons credit. I understand this statement.

Yes Choose an item.
