# Supplementary material for: AEnet: a practical tool to construct the splicing-associated phenotype atlas at a single cell level
Source: Gigascience. 2025 Sep 24;14:giaf110. doi: 10.1093/gigascience/giaf110 (PMC12457822; doi:10.1093/gigascience/giaf110)
Supplement: giaf110_Reviewer_3_Report_Revision_1 — Cyrillus Tan -- 7/14/2025 [file giaf110_reviewer_3_report_revision_1.pdf]

Reviewer Report

Title: AEnet: a practical tool to construct the splicing associated phenotype atlas at single cell level

Version: Revision 1 **Date:** 7/14/2025

Reviewer name: Cyrillus Tan

Reviewer Comments to Author:

I appreciate the authors' careful attention to the reviewers' comments. The updated manuscript has addressed many concerns raised in the reviews, especially* Clear separation of model description and benchmarking improves clarity* Benchmarking across multiple additional datasets and platforms enhance the generalizability* Batch effect handling is better explained* Ablation study (A-net vs. E-net) supports the value of joint modeling* Noise modeling and ASP thresholding are better testedOverall, this work has been strengthened. A few points should be fixed* Some of the workflow figures (e.g., Fig. 2, S2-S4) are visually confusing and could benefit from clearer visual layout or annotations.* In a few places, the revised manuscript still contains language that seems directly copied from the authors' point-by-point response. The writing would benefit from another editing pass to smooth out tone and narrative flow before final publication.

Level of Interest

Please indicate how interesting you found the manuscript: Choose an item.

Quality of Written English

Please indicate the quality of language in the manuscript: Choose an item.

Declaration of Competing Interests

Please complete a declaration of competing interests, considering the following questions:

- Have you in the past five years received reimbursements, fees, funding, or salary from an organisation that may in any way gain or lose financially from the publication of this manuscript, either now or in the future?
- Do you hold any stocks or shares in an organisation that may in any way gain or lose financially from the publication of this manuscript, either now or in the future?
- Do you hold or are you currently applying for any patents relating to the content of the manuscript?
- Have you received reimbursements, fees, funding, or salary from an organization that holds or has applied for patents relating to the content of the manuscript?
- Do you have any other financial competing interests?
- Do you have any non-financial competing interests in relation to this paper?

If you can answer no to all of the above, write 'I declare that I have no competing interests' below. If your reply is yes to any, please give details below.

I declare that I have no competing interests

I agree to the open peer review policy of the journal. I understand that my name will be included on my report to the authors and, if the manuscript is accepted for publication, my named report including any attachments I upload will be posted on the website along with the authors' responses. I agree for my report to be made available under an Open Access Creative Commons CC-BY license (http://creativecommons.org/licenses/by/4.0/). I understand that any comments which I do not wish to be included in my named report can be included as confidential comments to the editors, which will not be published.

Choose an item.

To further support our reviewers, we have joined with Publons, where you can gain additional credit to further highlight your hard work (see: https://publons.com/journal/530/gigascience). On publication of this paper, your review will be automatically added to Publons, you can then choose whether or not to claim your Publons credit. I understand this statement.

Yes Choose an item.
